# Two sets of RNAi components are required for heterochromatin formation *in trans* triggered by truncated transgenes

**DOI:** 10.1093/nar/gkw267

**Published:** 2016-04-16

**Authors:** Ulrike Götz, Simone Marker, Miriam Cheaib, Karsten Andresen, Simon Shrestha, Dilip A. Durai, Karl J. Nordström, Marcel H. Schulz, Martin Simon

**Affiliations:** 1 Molecular Cell Dynamics Saarland University, Centre for Human and Molecular Biology, Campus A2 4, 66123 Saarbrücken, Germany; 2 Department of Biology, University of Kaiserslautern, Erwin-Schrödinger Straße, Building Nr. 14, 67663 Kaiserslautern, Germany; 3 Institute of Biotechnology and Drug Research, Erwin-Schrödinger-Str. 56, 67663 Kaiserslautern, Germany; 4 Cluster of Excellence, Multimodal Computing and Interaction and Max Planck Institute for Informatics Saarland University, Department for Computational Biology and Applied Algorithmics, Campus E1 4, 66123 Saarbrücken, Germany; 5 Department for Genetics, Saarland University, Centre for Human and Molecular Biology, Campus A2 4, 66123 Saarbrücken, Germany

## Abstract

Across kingdoms, RNA interference (RNAi) has been shown to control gene expression at the transcriptional- or the post-transcriptional level. Here, we describe a mechanism which involves both aspects: truncated transgenes, which fail to produce intact mRNA, induce siRNA accumulation and silencing of homologous loci *in trans* in the ciliate *Paramecium*. We show that silencing is achieved by co-transcriptional silencing, associated with repressive histone marks at the endogenous gene. This is accompanied by secondary siRNA accumulation, strictly limited to the open reading frame of the remote locus. Our data shows that in this mechanism, heterochromatic marks depend on a variety of RNAi components. These include RDR3 and PTIWI14 as well as a second set of components, which are also involved in post-transcriptional silencing: RDR2, PTIWI13, DCR1 and CID2. Our data indicates differential processing of nascent un-spliced and long, spliced transcripts thus suggesting a hitherto-unrecognized functional interaction between post-transcriptional and co-transcriptional RNAi. Both sets of RNAi components are required for efficient *trans*-acting RNAi at the chromatin level and our data indicates similar mechanisms contributing to genome wide regulation of gene expression by epigenetic mechanisms.

## INTRODUCTION

RNA interference has been described to be an important epigenetic regulator in almost all eukaryotes. This includes post-transcriptional inactivation by mRNA cleavage or inhibition of translation. Moreover, newest insights describe a highly dynamic cross-talk between transcription, siRNA accumulation, and histone modifications: small RNAs guide heterochromatin formation by recruitment of the histone modifying machinery (1–3).

Three core components are described for RNAi in plants, animals, fungi and protists (4–7): RDR, Dicer and Argonaute. Small interfering RNAs (siRNAs) derive from double stranded RNA (dsRNA) precursors which are generated by bidirectional transcription or by the activity of a RNA-dependent-RNA-polymerase (RDR), converting single-stranded RNA (ssRNA) into dsRNA. The endonuclease Dicer or Dicer-like (Dcl) is required to cleave siRNAs of 20–24nt from dsRNA precursors. These are then bound by proteins of the Argonaute family: siRNAs guide Argonautes to their target in a homology-dependent manner, thus inducing silencing by RNA cleavage (post-transcriptional gene silencing, PTGS) or by recruitment of chromatin modifying components an individual locus (transcriptional gene silencing, TGS). Argonautes divide in the Ago clade which was mainly described to act on miRNAs and siRNAs whereas the Piwi clade appears to be expressed mainly in germline cells interacting with Piwi-interacting small RNAs (piRNAs) (8).

Especially research in *S. pombe* led to our current view of transcriptional silencing: siRNAs guide enzyme complexes to nascent transcripts to establish repressive chromatin marks. Thus, RNAi-induced heterochromatin formation requires active transcription (6). In this mechanism of co-transcriptional silencing (CTGS), *cis*-acting siRNAs are produced from the silenced locus itself.

Another class of small RNAs, piRNAs, were described to act in epigenetic silencing of transposable elements in the germline or germline related cells. The biosynthesis mechanism distinguishes piRNAs from siRNAs as the former are amplified in a Dicer-independent manner in the ping-pong cycle (8). However, recent evidence suggests that piRNAs also have siRNA-like functions because of (i) reports for piRNA-mediated silencing of mRNA producing loci in somatic tissues (9) and (ii) several studies revealing that piRNAs can trigger transcriptional silencing comparable to CTGS rather than pure PTGS: piRNAs direct Piwi to nascent transcripts, thus recruiting histone methyltransferases to silence transposons (reviewed in ref. (10)).

In almost all studied organisms, introduction of constructs producing dsRNA or hairpin RNA induces siRNAs or miRNAs, which silence gene expression of homologous endogenous genes. Introduction of hairpin RNA was also shown to induce efficient TGS in plants (11). However, *trans-*acting siRNAs that mediate TGS in *S.pombe* occur in few cases only: (i) if the target gene position is close to heterochromatic loci, (ii) in knockouts of the exoribonuclease *ERI1*, (iii) or in *HP1* (*Swi6*) over-expression lines at targets showing antisense transcription (12–14). As the classical system in which CTGS was discovered and subsequently characterized are the centromeric repeats in *S. pombe*, only few studies spent attention to CTGS regulation of protein coding genes (15), such as the regulation of stress response genes (16). Interestingly, interaction of RNAi and the exosome has also been shown to control heterochromatin formation at genomic domains of genes and retrotransposons (17). We therefore just started to discover the extent of RNAi induced heterochromatin formation as well as to understand which endogenous trigger mechanisms interact with the RNAi machinery.

Ciliates are unicellular organisms with an equidistant phylogenetic relationship to the kingdoms of plants, animals and fungi. Although being unicellular, they evolved differentiation between germline and soma by presence of generative Micronuclei (Mic) and a somatic Macronucleus (Mac) in a single cell. A developmental-specific RNAi pathway has been described in *Paramecium tetraurelia* and *Tetrahymena thermophila* which produces scnRNAs during meiosis, controlling the DNA content of F1 progeny by RNA-induced DNA eliminations (18). Apart from the germline-specific scnRNA pathway, also vegetative RNAi has been extensively studied in *Paramecium* by PTGS, induced by feeding of dsRNA producing bacteria involving siRNAs (19–22). In *Paramecium*, recent studies demonstrate another RNAi pathway involving the highly divergent RNA-dependent RNA polymerase RDR3 to control endogenous gene expression, suggestive of transcriptional control (22,23). Further, RNAseq analyses suggest that such epigenetic mechanisms contribute to transcriptional robustness of the transcriptomic landscape in *P. tetraurelia*, thus raising the question about the extent and the molecular details of this mechanism (24).

It was therefore the aim of this study to uncover the molecular characteristics of *trans* silencing in *P. tetraurelia*. RNAi against endogenous genes can be triggered by introduction of truncated transgenes (25,26). We have previously shown that transgene-induced silencing depends on RDR3 (22). Analysis of further genetic requirements suggests that this pathway is distinct to post-transcriptional silencing, although individual RNAi components are shared by both pathways (19–22). However, it remains unclear why and how truncated transgenes trigger RNAi. Here, we show that transgene induced silencing works at the chromatin level and is triggered by a variety of transgene derived siRNAs with complex genetic requirements.

## MATERIALS AND METHODS

### Cell culture & RNAi by feeding

*Paramecium tetraurelia* strain 51 was grown in wheat grass powder (WGP, Pines International Co., Lawrence, KS) infusion medium bacterized with *Klebsiella pneumonia* supplemented with 0.8 μg/ml β-sitosterol, unless otherwise stated. RNAi by feeding was induced as described (27,28), by cloning a gene fragment between inversely oriented T7 promoters. Transformation of this plasmid into RNAse III deficient *Escherichia coli* strain HT115DE3 allowed for induction and accumulation of dsRNA by addition of 0.4 mM isopropyl-β-d-thiogalactopyranoside (IPTG), inducing the chromosomal P_lac_ driven T7 polymerase gene followed by growth in LB medium for 2 h. Induced bacteria were washed and resuspended in WGP medium and applied to *Paramecium* cultures. All transgenic cultures were fed with induced bacteria for three days before RNA isolation.

### Microarray statistics and GO enrichment

Microarray data processing was done using R (R Development Core Team (2012), http://www.R-project.org/) and Bioconductor (29). An annotation package was built with pdInfoBuilder using raw data files (.xys) along with Nimblegen microarray design file (.ndf). All microarray data were RMA normalized using the package *oligo* in Bioconductor. The normalized data were fitted to a linear model (30) using the LIMMA package. Differentially expressed genes were chosen based on the Empirical Bayes method and a comparison of contrasts along with adjusted *P*-values for multiple testing using a false discovery rate (31) <0.05 and a log_2_-fold change >1. Statistically significant differentially expressed genes from microarrays were analyzed using the gene enrichment test topGO (32) on GO terms within the GO subontology *biological process* (BP). The method for significance testing was Fisher's exact test with *P*-values <0.05. The analysis is based on previously published microarray data (23) deposited at the Gene Expression Omnibus database (33) under accession number GSE59390.

### Microinjections

Endotoxin free plasmids were isolated using the NucleoBond^®^ Xtra EF Kit (Machery & Nagel, Düren, Germany), linearized at the BglI restriction site in the vector sequence, phenol extracted and filtered with a 0.22 μm Ultrafree-MC filter (Merck-Millipore, Darmstadt, Germany). After dissolving at a concentration of 5 mg/ml, ∼3–8 pl DNA was injected in the macronucleus of cells younger than 6 divisions after autogamy. Before, cells were washed in Dryl′s phosphate buffer (2 mM Na_3_C_6_H_5_O_7_, 1 mM NaH_2_PO_4_, 1 mM Na_2_HPO_4_, 1.5 mM CaCl_2_) supplemented with 0.2% bovine serum albumin and immobilized in an aqueous drop under paraffin oil. The pTI+ and pTI- constructs harbouring a constitutive bidirectional promoter from scaffold 93 position 237470–239396 are described in ref. (21). In the pTI-/- construct, the fragment between position 173–529 relative to the truncated *ND16*9 gene was removed. In the pTI-/-K28 construct, a fragment of the *Saccharyomyces cerevisiae K28* preprotoxin orf (position 541–935 of the cloned gene (34), kind gift of B. Becker & M. Schmitt, Saarbrücken) was cloned into pTI-/- at position 625–1010 relative to the *ND169* ATG.

### RNA-isolation and northern blots

Total RNA from *Paramcium* was isolated using TriReagent^®^ (Sigma–Aldrich, Seelze, Germany). Denaturing gel electrophoresis and northern blotting was carried out as described before (22). The blots were hybridized with radioactively labelled probes in church buffer (0.25 M phosphatebuffer pH 7.2, 7% SDS, 1% BSA). Random primed PCR-templates synthesized with Klenow (exo-) polymerase were used as a probe for the *ND169* specific siRNAs, hybridized at 42°C. Probes used for loading control were generated using T4-PNK and were hybridized at 60°C.

### RNA isolation, library preparation, sequencing, post-processing

Small RNA fractions (17–25nt) were gel purified starting from 50 μg total RNA. Small RNAs (17-25nt) were extracted from 17.5% urea acrylamide gels stained in SYBR^®^-Gold (Life-Technologies, Darmstadt, Germany). Gel slices were smashed and small RNAs were extracted by overnight incubation in 3Vol of 0.3M NaCl. After precipitation with 3 Vol EtOH and 70ng/μl glycogen, the small RNA fraction was cloned using the NEBNext^®^ small RNA library prep Kit (NEB, Frankfurt a.M., Germany), following the manufacturer's instructions (18 h 3′-adapter ligation at 16°C). Sequencing was carried out on a HiSEQ2500 Illumina platform using the default RTA analysis software. Long RNA libraries were prepared as described before in (24). Ribo depletion was done with the Yeast Ribo-Zero™ Magnetic Gold Kit (Illumina, Munich, Germany).

Small RNA sequencing was done on an Illumina HiSEQ2500 Platform using the Rapid mode with a read length of 50 nucleotides. Reads were de-multiplexed and subsequently adapter trimmed (see below) and filtered for lengths between 17 and 25nt. Sequencing data of long RNAs were first checked for adapter contamination. Adapter sequences were trimmed using Trim Galore (http://www.bioinformatics.babraham.ac.uk/projects/trim_galore/) that uses Cutadapt (35) with a stringency cutoff of 10. All sequences longer than 60nt were kept for further analysis. These sequences were aligned to the transgene and to the endogenous ND169 gene using Bowtie2 (36) allowing up to one mismatch. Bowtie2 was used with –local mode to accommodate adapters that were not removed. Aligned reads were then separated into reads mapping to the forward and the reverse strand of the reference sequence. These reads were further analyzed using samtools (37).

### Splice rate analysis

To compute splice rates we calculated the number of reads which were spliced as follows: all reads overlapping the intron are considered as splicing candidates if they align to the adjacent exon region surrounding the intron and have a gap at the intron region. If reads aligned to more than four basepairs of the intron (starting from the boundary of the intron) then the read is said to be un-spliced. From un-spliced and spliced read candidates we computed splice rates.

### Small RNA alignment and processing

Small RNA reads have been aligned using Bowtie2 (36). Alignments were done in two rounds. First reads were aligned against a number of genome sequences that could have generated small RNAs due to contaminations. Second, all reads that did not match in the first round were aligned against the *P. tetraurelia* MAC genome (version 51) and coding sequence annotation (version 1.91) downloaded from ParameciumDB (38). Only read alignments without mismatches were kept. Read overlaps with target regions was done using custom scripts. Visualization and normalization was implemented in the statistical programming language R. Raw data were deposited at the European Nucleotide Archive (ENA, http://www.ebi.ac.uk/ena) under study accession no. PRJEB13116.

### Normalization of small RNA reads

As the sequencing runs produced different numbers of reads, we had to normalize read counts for comparison between samples. Sequencing statistics and read count of Mac mapping reads can be found in Supplementary Figure S8. We are not aware about normalization methods specialized for small RNA-seq data sets. However, many methods have been proposed to normalize mRNA-seq data. There are two prevalent classes of mRNA-seq normalization methods, (i) total count scaling (TCS) methods and (ii) methods that analyze the median log fold change, or a similar quantity, among all genes between mRNA-seq experiments (39). The latter methods assume that gene annotation is available and that most of the genes between samples are not differentially expressed. As little is known about the location, size, and expression variability of endogenous small RNA loci in *P. tetraurelia*, this second class of methods was not applicable.

Therefore we used a variant of TCS as introduced for mRNA-seq data (40), which normalizes by scaling through a factor that estimates the difference in the number of reads mapped between samples. However, the disadvantage of TCS methods is that the used normalization factors were shown to be biased by highly expressed genes in the dataset, because this kind of normalization ignores the fact that the repertoire of expressed RNAs may change between conditions (39). This effect is exacerbated in our knockdown samples compared to wild type samples, because large amounts of siRNAs against the knockdown gene are introduced into the cells. These primary siRNAs are known to trigger the production of other, secondary siRNAs, against the knockdown gene, all of which we are sequencing. Therefore, we removed from the estimated total library size, all small RNA reads that mapped against the knockdown gene(s), this quantity is denoted *K* below. Further, we removed small RNAs from highly abundant structural RNAs that are known to produce small RNAs, e.g. ribosomal RNAs, which are mostly RNA degradation products. In addition, other small RNAs that may have been introduced due to feeding cultures are removed, leaving only small RNA reads mapping to the rest of the *Paramecium* genome, this quantity is denoted *T* below.

In detail, assume read count *R* for a region of interest that we want to compare between samples. Denote as *T* the total number of reads mapping to the genome, as described above, and denote as *K* the number of small RNA reads mapping to the knockdown gene. We compute the normalized read count $\hat R$:

\begin{equation*}\hat R = R \cdot \frac{M}{{T - K}},\end{equation*}

where *M* is the maximum over all values (*T*_1_ – *K*_1_),…,(*T_n_* – *K_n_*) over all *n* samples. The normalization and comparison functions for small RNA seq data are accessible as an analysis pipeline under https://github.com/SchulzLab/RAPID.

### Chromatin-immunoprecipitation (ChIP)

Chromatin isolation, sonication, fixation and immunoprecipitation were carried out as described previously using shearing fragments of 300–500 bp (41). The antibodies used were αH3 and H3K27me3 (07-442 and 07-449; Merck-Millipore, Darmstadt, Germany), and αH3K9ac and αH3K4me3 (ab4441 and ab8580; Abcam, Cambridge, UK). The H3K27me3 antibody has been shown to specifically detect this modification in *Paramecium* histone H3 in spite of one amino acid mismatch to the immunogen peptide (42). Immunogenic peptides of the remaining antibodies fit 100% to *Paramecium* histone H3. DNA quantification of ChIP eluates was carried out by qPCR with the SsoAdvanced™ SYBR^®^Supermix (BioRad, Munich, Germany). All ChIPs were carried out in biological triplices, thus three cultures (wild type or feeding) of the same transgenic line with one ChIP per antibody each and three technical PCR replicates each.

## RESULTS

### Microarray analysis indicates *RDR3* involved in heterochromatin formation

We previously identified RDR3, a highly divergent RDR, involved in transgene-induced silencing of endogenous genes as well as in endogenous regulation of the transcriptionally controlled multigene family of surface antigens (22). Microarray analysis of *RDR3* knockdown cultures revealed that ∼20% of annotated genes are differentially expressed genes (DEGs) including the surface antigen multigene family (23). Interestingly, silencing of RDR3 does not solely activate gene expression, but many genes are down-regulated. This cannot be attributed to direct interaction with RDR3 but may be the result of genome-wide deregulation of gene expression pathways. Table 1 shows GO term enrichment of these DEGs indicating processes of chromatin assembly being down-regulated, and processes associated with transcriptional activity up-regulated, thus suggesting an association of *RDR3* and heterochromatin formation (detailed gene lists of diff. regulated GO terms can be found in Supplementary Data File 2).

**Table 1.** Top 10 GO Terms found by gene enrichment on differentially expressed genes in *RDR3* silenced cultures

GO.ID	Term - Downregulated	Annotated	Significant	*P*-value
GO:0006333	chromatin assembly or disassembly	108	56	1,06E-31
GO:0051276	chromosome organization	127	58	5,47E-29
GO:0006325	chromatin organization	123	57	6,41E-29
GO:0006323	DNA packaging	58	37	1,02E-25
GO:0006334	nucleosome assembly	58	37	1,02E-25
GO:0031497	chromatin assembly	58	37	1,02E-25
GO:0034728	nucleosome organization	58	37	1,02E-25
GO:0065004	protein-DNA complex assembly	58	37	1,02E-25
GO:0071824	protein-DNA complex subunit organization	58	37	1,02E-25
GO:0006996	organelle organization	187	66	5,64E-25
**GO.ID**	**Term - Upregulated**	**Annotated**	**Significant**	***P*-value**
GO:0016310	phosphorylation	2624	294	1,32E-09
GO:0006468	protein phosphorylation	2533	282	8,35E-09
GO:0006793	phosphorus metabolic process	2707	297	1,17E-08
GO:0006796	phosphate-containing compound metabolic process	2707	297	1,17E-08
GO:0006464	protein modification process	2836	299	7,56E-07
GO:0043412	macromolecule modification	2846	299	1,08E-06
GO:0010468	regulation of gene expression	472	64	8,57E-05
GO:0006351	transcription, DNA-dependent	466	63	1,06E-04
GO:0006355	regulation of transcription, DNA-dependendent	466	63	1,06E-04
GO:0051252	regulation of RNA metabolic process	466	63	1,06E-04

The GO term enrichment is based on genome wide microarray expression data of cells undergoing *RDR3* silencing compared to cells undergoing control silencing of an unrelated gene (*ICL7a*). The upper part indicates significantly downregulated-, and the lower part unregulated GO terms (including redundant terms), along with the total number of annotated genes, the number of differentially expressed genes and the individual *P*-value. (For details see Supplementary Figure S1A and SB).

We therefore asked for the silencing trigger, small RNAs, and further RNAi components to be associated with RDR3-mediated silencing. For this approach, the transgene-induced silencing mechanism served as a model system to mimic endogenous silencing *in trans*.

### Different truncated transgenes induce silencing of endogenous genes

**Figure 1.**
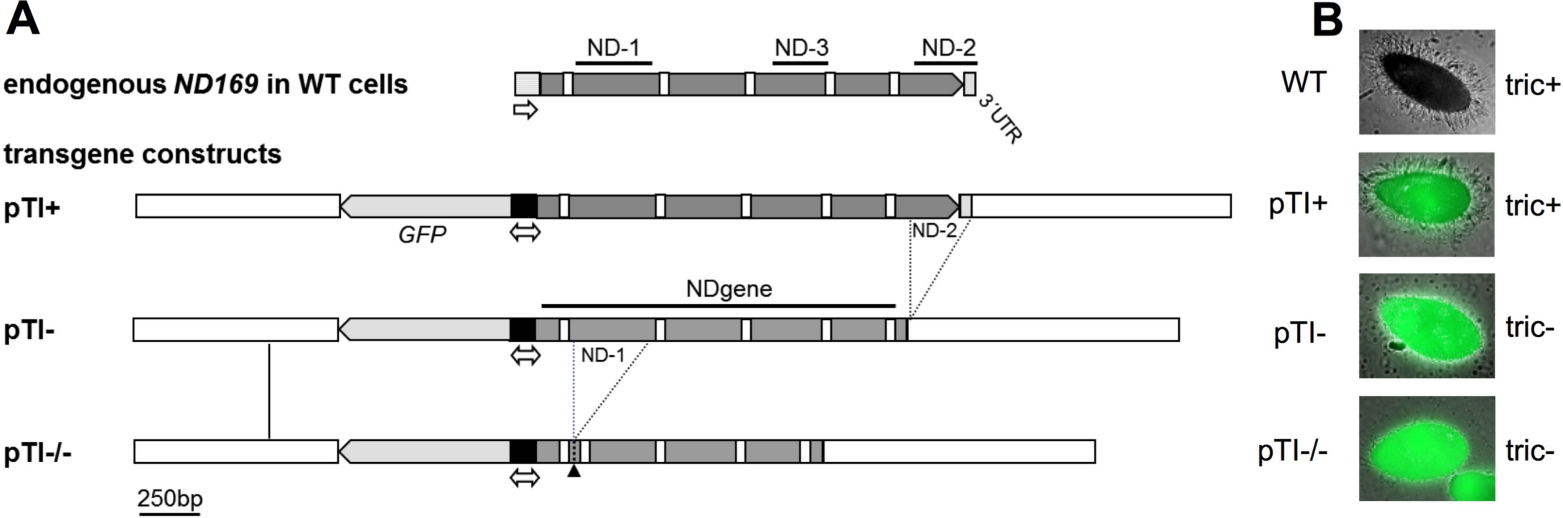
Transgene constructs for silencing of the *ND169* gene. (**A**) Transgene constructs in their linearized form containing a *GFP* orf and different versions of *ND169* required for trichocyst discharge, both driven by the same bidirectional promoter. The control construct pTI+ contains the full length *ND169* gene including the 3′-regulatory untranslated region (3′-UTR). The 3′-part of the coding sequence (CDS) and the 3′-UTR are lacking in the pTI- construct (ND-2). A second fragment of the 5′- coding region was removed in the pTI-/- construct (ND-1). The artificial ND-1 junction is indicated by the black arrowhead. Introns are indicated by white boxes. (**B**) Trichocyst discharge phenotypes of wild type (WT) and transgene-injected cells after stimulation with picric acid. WT and pTI+ cells showed full trichocyst ejection (tric+); cell lines injected with pTI- or pTI-/- constructs were silenced in trichocyst discharge (tric–).

In this study, we used different transgene constructs to trigger silencing of the endogenous *ND169* (endo *ND169*) gene, serving as a reporter gene here. Its gene product is necessary for membrane fusion during trichocyst discharge. The plasmid map of pTI+, which uses a bidirectional promoter for expression of *GFP* and the native version of the *ND169* gene is shown in Figure 1A. Microinjection of the pTI+ construct results in tric+ cells, meaning that trichocyst discharge is not altered compared to wild type (Figure 1B). As reported previously, truncation of the *ND169* transgene induces silencing of the endo *ND169* locus. This effect was described for the 3′-truncated version pTI- which lacks a part of the 3′-CDS and 3′-UTR (22). Here, we found that the newly designed pTI-/- construct, which additionally lacks a part of the 5′-CDS, triggers silencing as well when injected in high copy numbers (Figure 1A and B).

Northern blot analysis showed over-expression of *ND169* mRNA in pTI+ cells, whereas aberrant transcripts are produced in pTI- cells: their size corresponds to the distance between the promoter and the linearization site (Supplementary Figure S3B). Thus, mRNA transcription appears to impede siRNA synthesis, which is in agreement with our previous data showing that pTI+ does not induce siRNA accumulation (22).

### Bidirectional transcription of the transgene accumulates spliced and un-spliced RNA

**Figure 2.**
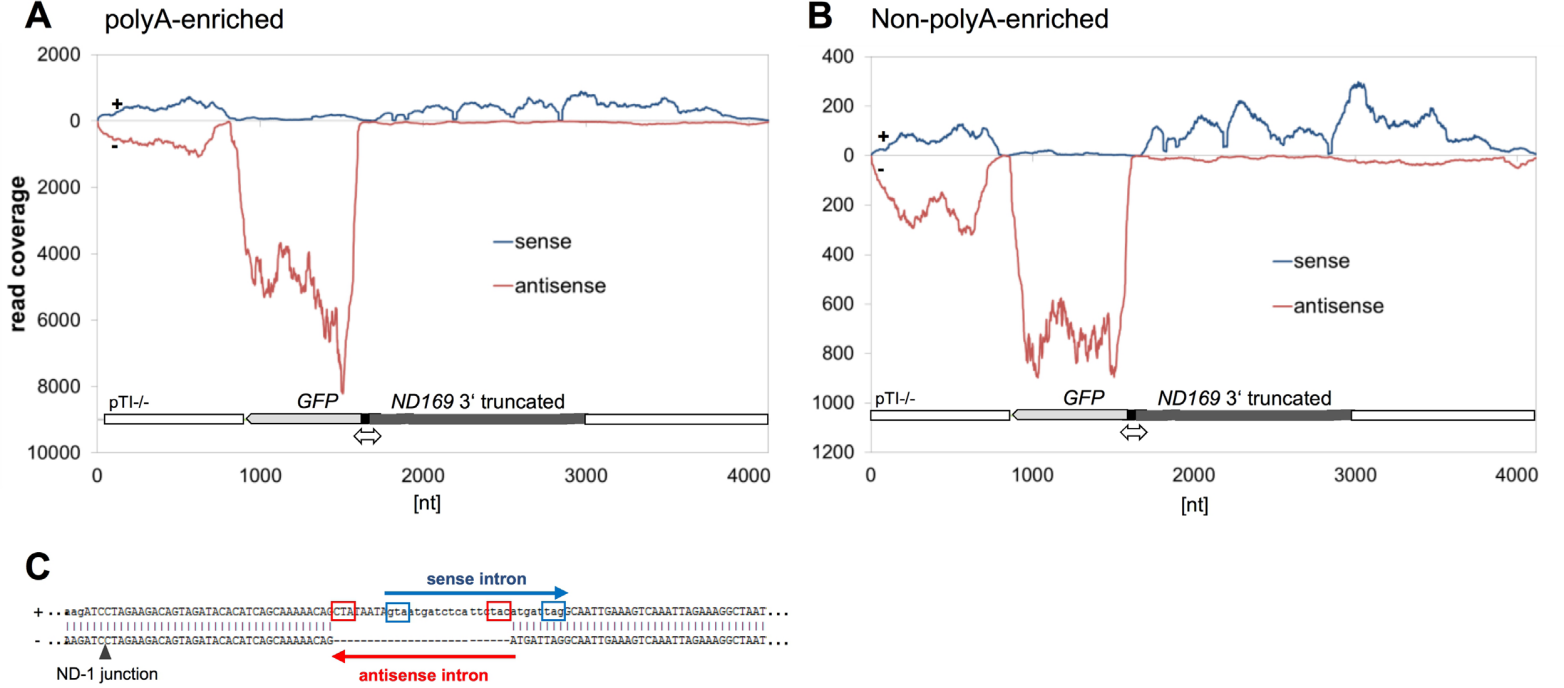
Long RNAs of the pTI-/- transgene. Directional RNA-Seq of polyA-enriched RNA (**A**) and rRNA-depleted/non-poly-A enriched RNA (**B**) prepared from pTI-/- cells. The coverage of each nucleotide position with transcripts from the top (+) strand (blue) and bottom (–) strand (red) is shown. The *GFP* orf (bottom strand) is indicated by the light grey arrow, the truncated *ND169* orf by a dark grey bar, with gaps representing introns. Transcription of both is driven by the same bidirectional promotor (black). (**C**) Schematic representation of antisense intron 1, found to be spliced in pTI-/- injected cells. This intron was identified by Sanger sequencing of strand-specific RT-PCR products (see Supplementary Figure S2), and in the directional RNA-Seq data set described in (B). Sense intron 2 was not spliced in the same transcript.

**Figure 3.**
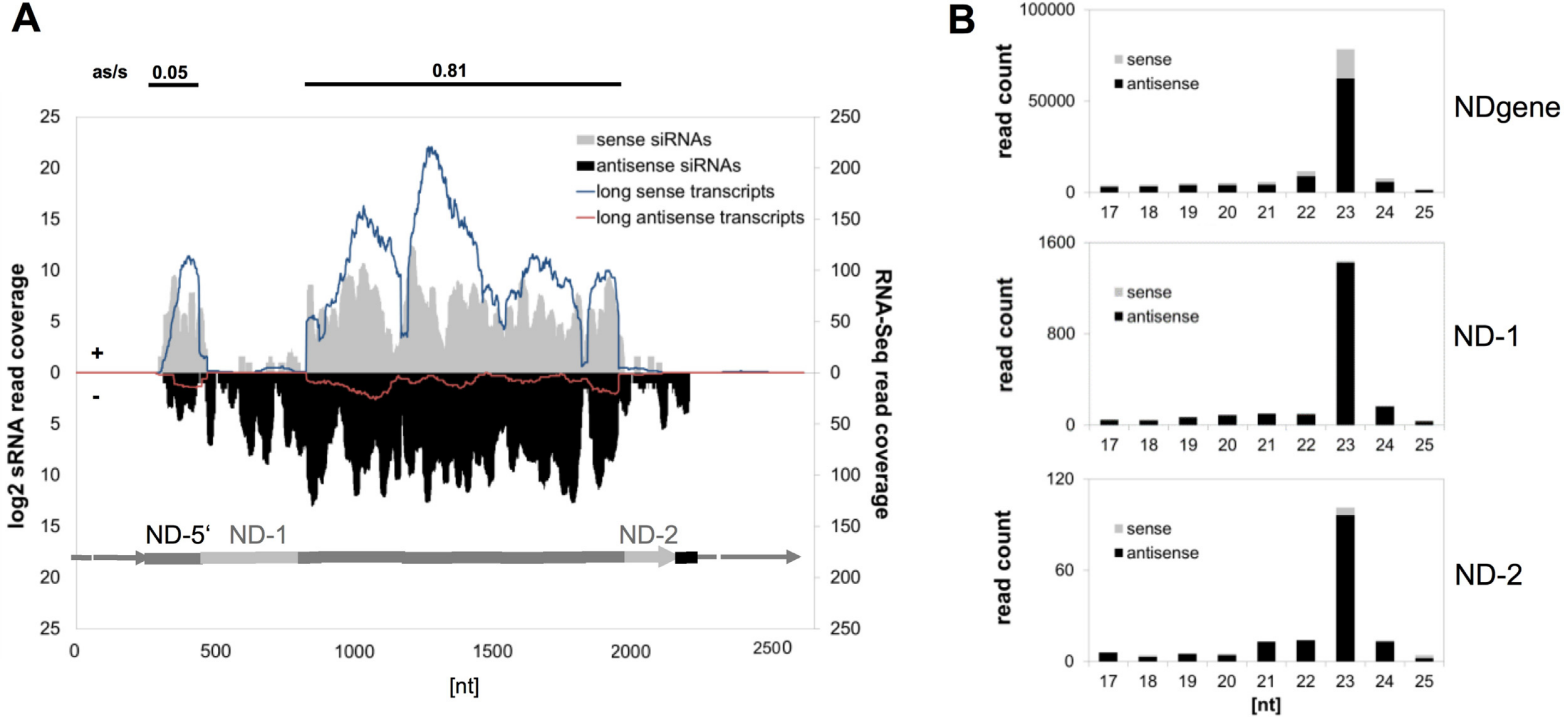
Truncated transgenes induce transitivity at the endogenous locus. (**A**) SiRNA reads from pTI-/- injected cells were mapped to the endogenous *ND169* gene. 23nt sense siRNA levels are shown in grey, antisense in black. *ND169* and parts of the flanking regions are indicated above the x-axis. Introns are represented by gaps. Light grey bars represent regions not present in the transgene, indicating secondary siRNA production from the endogenous *ND169* locus. On top, the ratio of antisense to sense 23nt siRNAs is indicated for the 5′-region of the *ND169* CDS (ND-5′) and for the NDgene region. Long transcripts revealed by RNA-seq (non-polyA enriched, see Figure 2B) are depicted in blue (sense) and red (antisense). Similar results were obtained for the pTI- transgene (see Supplementary Figure S3). (**B**) Secondary siRNAs (ND-1 and ND-2) and primary siRNAs are predominantly 23nt in length. Secondary siRNAs showed a much stricter antisense bias compared to primaries.

We first searched for the silencing trigger molecules produced by the transgene and we sequenced long RNAs from transgene cultures. Figure 2 shows directional long RNA sequencing after polyA enrichment (2A) and after depletion of ribosomal RNA and non-polyA enrichment (2B) of pTI-/- cells. Reads were mapped to the transgene sequence. In both analyses, the intron-less, native *GFP* gene showed the highest read count. The truncated *ND169* fragment, lacking the transcription stop signal, shows transcription of the coding strand towards the downstream linearization site. Five *ND169* introns, with the typical length of *Paramecium* introns of ∼25nt, remained in the double-truncated pTI-/- sequence. We subsequently analysed splice efficiency by counting reads mapping to intron and exon/exon junctions (Supplementary Table S1) revealed only a slight difference of the proportion of spliced transcripts for the five sense introns between the native endogenous gene (average of 90%), the poly-A enriched (78.6%) and in the non-polyA enriched (70%) pTI-/- data sets. Thus, un-spliced transcripts accumulate in the presence of the transgene. As the vast majority of the detected derives from the transgene and not from the endogenous gene (see Figure 3A), we conclude that the transgene RNA shows a lower splice frequency compared to the endogenous gene. Our data does not allow for conclusion whether this is significantly correlated to polyadenlyation.

As RNAi mechanisms usually start with dsRNA triggers, we aimed to analyse antisense RNA (asRNA) for the presence of sense introns or sense intron junctions to distinguish whether this RNA results from bidirectional transcription or RDR activity. However, sequencing of long RNA yielded only few antisense reads. We did not find any long antisense RNAs showing sense exon/exon junctions which would indicate RDR activity on spliced sense RNAs. However, we found two antisense introns in antisense reads. As these reads also contain the transgene junction, they need to derive from the transgene and not from the endogenous locus. Figure 2C shows a schematic representation of an antisense read which contains sense intron no. 2 but shows splicing of an antisense intron with the typical *Paramecium* intron boundaries. Splicing of these antisense introns was verified by Sanger sequencing of strand-specific RT-PCRs (Supplementary Figure S2). Thus, splicing of antisense- but not sense introns in antisense RNAs clearly demonstrates bidirectional transcription at the transgene locus.

### Transgene-siRNAs trigger 5′-monophosporylated secondary siRNAs

To analyse siRNAs of the transgene, deep sequencing of small RNA was carried out and reads were mapped to the endogenous *ND169* locus. In Figure 3A, small RNA reads are combined with the coverage of reads deriving from non-polyA enriched/ribo-depleted long RNAseq. The Figure shows that *ND169* mapping siRNAs were predominantly antisense in almost the entire transgene region (NDgene), except for the ultimate 5′- region (ND-5′). The region between the transcription start site and the first intron showed a clear sense bias, suggesting that siRNAs at the 5′-end have distinct accumulation criteria.

Interestingly, siRNAs were also apparent in regions of the endogenous *ND169*, which are not part of the transgene (ND-1 and ND-2), thus demonstrating that siRNA production and transitivity occurs at the remote locus. In the following, we refer to these siRNAs as secondary siRNAs. For the subsequent analyses we had to consider that reads mapping to the transgene represent a mix of primary (1°) and secondary endo *ND169* (2°) siRNAs. The coverage of 2° siRNAs was drastically lower compared to the transgene sequence (∼5% in ND-1, ∼1% in ND-2), thus we conclude that the majority of transgene-mapping siRNAs represent 1° siRNAs. The read length distribution showed all siRNAs to be predominantly 23nt (Figure 3B), which was also the major length of small RNAs reported in PTGS induced by dsRNA feeding in *Paramecium* (19,43).

A closer look at the 2° siRNA coverage revealed that the ND-1 region adjacent to the 5′-end accumulated more siRNAs than the ND-2 region located at the 3′-end. This unequal distribution of 2° siRNAs is different in PTGS in *Paramecium* showing an even coverage of 2° siRNAs along the mRNA (43). However, at this point, we cannot exclude that different mechanisms trigger 2° siRNAs. 5′- and 3′-transitivity could trigger 2° siRNAs in the ND-1 region. Contrarily, in the ND-2 region only 3′-transitivity occurs, because no 1° siRNA can target downstream of the ND-2 region (see below).

To ensure that we do not miss a large number of small RNAs due to the 5′-monophosphate specific cloning procedure and thus to exclude that another small RNA species such as 5′-triphosphorylated RDR products exist, we treated transgene RNA samples with pyrophosphatase and prepared libraries containing both, 5′-mono- and potential di- and tri-phosphorylated siRNAs (Supplementary Figure S4). Pyrophosphatase treatment did not alter the predominant length of sequenced RNAs of 23nt, nor the ratio between 1° and 2° siRNAs (Supplementary Figure S4B). We previously demonstrated that NDgene mapping siRNAs are 5′-monophosphorylated using northern blots (22). Therefore, we conclude, that 2° siRNAs (of ND-1 and ND-2 regions) are predominantly monophosphorylated. This indicates that a Dicer or a putative phosphatase is involved 2° siRNA biogenesis and that these are no direct and unprocessed *de novo* initiated RDR products. The normalized read count of siRNAs after phosphatase treatment is slightly lower compared to untreated samples (Supplementary Figure S4B).This is likely due to normalization: if more small RNAs map to the *Paramecium* genome after phosphatase treatment, the relative number maps to the transgene becomes smaller. Vice versa, this indicates that other small RNA species exist in *Paramecium*. Whereas we show here that the occurrence of 2° siRNAs is a result of a high copy transgene in the pTI-/- line (Figure 3A), we can demonstrate that 2° siRNA accumulation is not due to over-expression of the transgene. In Supplementary Figure S5 we show siRNA quantifications of pTI-/- lines injected with different copy numbers. Even in a line harbouring a transgene copy number of only ∼2-fold higher than that of the endogenous gene, 2° siRNAs could be detected. In all analysed transgenic lines, the amount of 2° siRNAs is ∼2-5% of the 1° siRNA abundance independent of the transgene copy number (Supplementary Figure S5F). We can conclude that 2° siRNA accumulation is a direct function of 1° siRNA abundance and the occurrence of 2° siRNAs in the low copy number transgenic line (pTI-/-1) indicates that this kind of transitivity can also be triggered by endogenous defective genes such as pseudogenes.

### Genetic requirements of 1° and 2° siRNAs

A series of RNAi components have been described in previous studies to be involved in transgene-induced silencing by phenotypic analysis. These components include RDR3, the Piwi ohnologs PTIWI08 and PTIWI14, and a set of RNAi components which are also involved in PTGS: RDR2, CID2, DCR1 and PTIWI13 (19–22). This raises the question why two sets of RDRs and Piwis should be necessary for efficient silencing. RDR2 is a canonical and essential RDR which does not show phenotypes in pTI- transgene lines but only those showing moderate silencing (21,22) and, in our study, in all pTI-/- transformed clones.

To see whether the involvement of the two sets of RNAi components is due to different genetic requirements of 1° and 2° siRNAs, we induced knockdown of all individual RNAi components and quantified siRNAs of the defined *ND169* regions by deep sequencing. As the importance of PTIWI08 in transgene-induced silencing could not be clearly distinguished from that of its highly similar ohnolog PTIWI14 (20), we induced double knockdowns to ensure proper silencing of both genes. However, northern blot analyses indicated that PTIWI08 contributes only to a minor degree to siRNA accumulation during vegetative growth (Supplementary Figure S6C).

**Figure 4.**
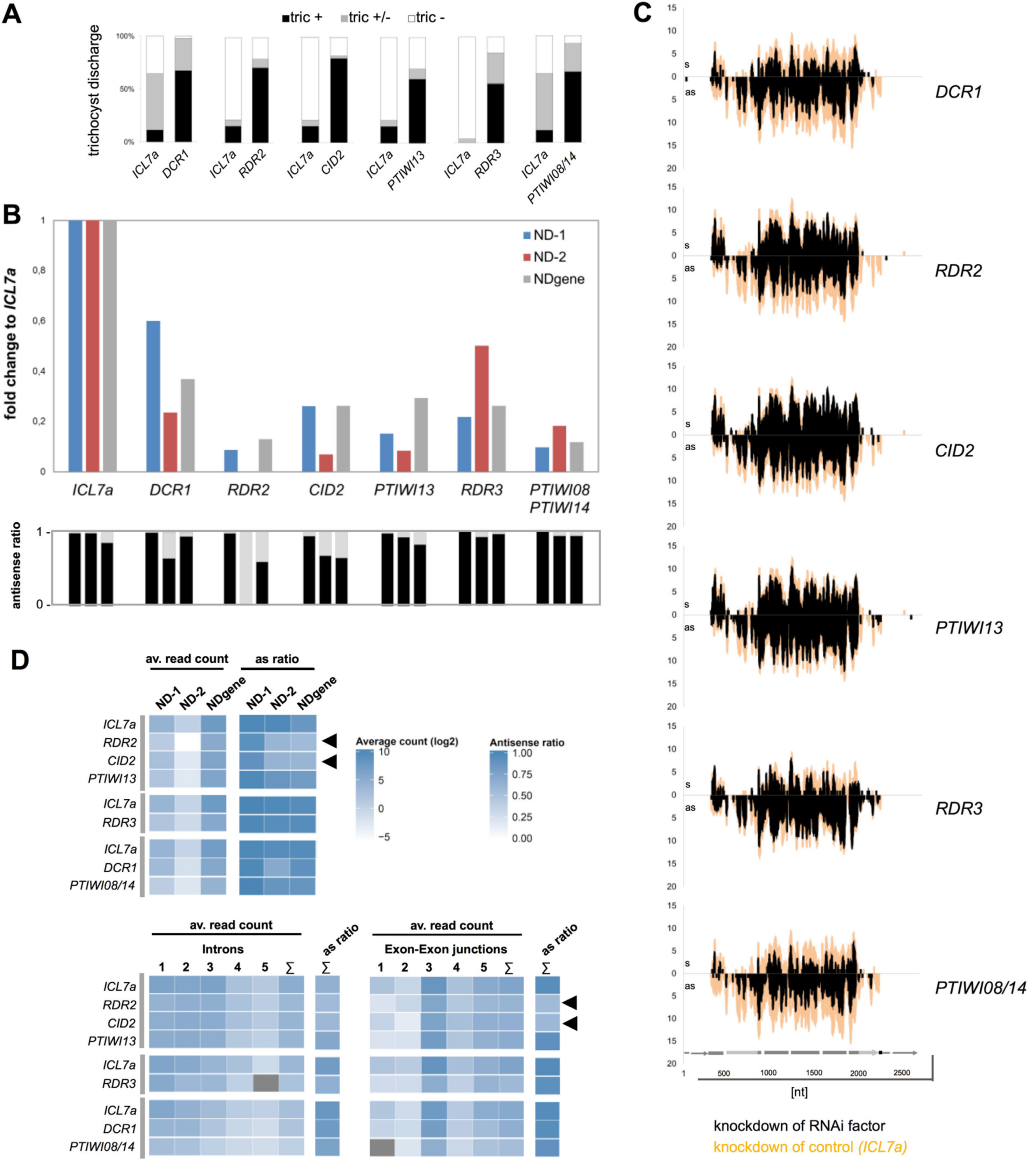
Knockdown of RNAi components reduces primary 23 nt small RNAs. (**A**) Phenotypes of knockdown lines and the respective control (*ICL7a*) were calculated counting cells with no (tric −), partial (tric +/−) or complete (tric +) trichocyst discharge. (**B**) Fold-changes to the control dsRNA feeding (*ICL7a*) are shown for 23nt siRNAs mapping antisense to the *ND169* endogenous regions ND-1 and ND-2, and to the transgene-covered region NDgene. Antisense ratios of 23nt siRNAs are given below. Similar results were obtained in replicate experiments and northern blots (Supplementary Figure S6A/B). (**C**) siRNA coverage of *ND169* with 23nt sense (s) and antisense (as) siRNAs in knockdown conditions. The coverage of the respective control sample (*ICL7a* feeding) is indicated in orange. (**D**) Heat maps showing the average sense and antisense 23nt siRNA read count (top) and the respective antisense ratio (bottom) on introns and exon-exon junctions of *ND169*. Individual injected clones are shown with their respective *ICL7a* control, indicated on the left by grey lines. Arrowheads point to discussed changes in the antisense ration in RDR2/CID2 silenced cultures.

All knockdown experiments were carried out in biological replicates with different transgenic lines using RNAi by dsRNA-feeding against the different components. To guarantee for comparable transgene copy numbers, the different transgenic lines were chosen by fluorescence analysis of GFP which we found to correlate with transgene copy number as well with the phenotype (Supplementary Figure S5). Two samples per RNAi component were chosen from different transgene lines for RNA analysis. Figure 4 shows data of the replicate series with stronger phenotypes (data of the replicate series with weaker phenotypes in Supplementary File S6B) involving phenotypic data in Figure 4A, fold change of normalized siRNA read counts to the individual reference in Figure 4B and coverage plots of 23nt siRNAs are shown in Figure 4C.

Our data shows that no RNAi component exclusively affected 2° siRNAs (ND-1 and ND-2). In contrast, knockdown of any RNAi component reduced siRNAs mapping to the NDgene region. Their reduction cannot be due to a pure loss of 2° siRNAs, because their maximum coverage in the ND-1 region was only ∼5% of that of the NDgene region: therefore, 2° siRNAs appear to have lower abundance and their loss cannot explain the reduction of siRNAs in the NDgene region. The finding that all components are indeed involved in 1° siRNA accumulation is confirmed by the replicate series shown in Supplementary Figure S6B. As mentioned above, these lines show a weaker reduction of transgene siRNAs compared to the first series (Figure 4B). The weaker effect can be explained for most lines (except *RDR3* and *PTIWI08/14*), as the decrease of transgene siRNAs correlates with the number of dsRNA-feeding associated siRNAs, representing the feeding efficiency and thus the knockdown efficiency of the RNAi component (Supplementary Figure S6A/B). It seems tempting to speculate here that RNAi against *RDR3* and *PTIWI08/14* (which our data suggest to be associated in CTGS as discussed below) may involve a self enforcing loop of RNAi stabilization. The reduction of transgene-induced silencing would then not solely represent the function of *RDR3* and *PTIWI08/14* silencing efficiency.

Consequently, this indicates that all RNAi components are involved in 1° siRNA accumulation and we can exclude that a subset is exclusively necessary for 2° siRNAs. In our data, the interpretation of 2° siRNA fold changes appears difficult, as additional to the knockdown of RNAi components also the abundance of 1° siRNAs greatly affects 2° siRNA synthesis, as mentioned above. Although tempting, we cannot attribute any RNAi component to be additionally involved in 2° siRNA accumulation by their stronger effect on 2° siRNAs. However, the data shows that both regions specific for 2° siRNAs do not react in the same manner/uniformly to the knockdown. In both replicate series, ND-1 shows for instance less sensitivity to *DCR1* silencing compared to ND-2, otherwise the ND-2 region shows the greatest fold change in *RDR2* knockdown lines. Therefore, the data indicates different behaviour of 2° siRNAs in the 5′- and the 3′ -region, thus suggesting different accumulation pathways.

### Two classes of 1° siRNAs?

Analysing the strand orientation of 1° siRNAs, we found that only *RDR2* and *CID2* knockdown lines altered the strict antisense ratio of 1° siRNAs (shown in Figure 4B). This raises the question why this effect cannot be seen in all knockdown lines with sufficient reduction of siRNAs. As we described above that the ultimate 5′-region of the transgene shows a sense- rather than an antisense bias, we already hypothesized that the transgene derived 1° siRNAs may have more than one accumulation pathway. To uncover potential differences in the bulk of 1° siRNAs, we focused on intron- and exon/exon junction-mapping siRNAs. Our analysis of long RNAs did not indicate accumulation of RDR-derived precursors, as very few antisense transcripts with spliced sense introns were detected. In small RNA data however, a large number of antisense siRNAs mapping to sense exon-exon junctions were detected (Figure 4D). This clearly demonstrates that RDR activity on a sense template strand occurs. These long precursors appear to become very rapidly and quantitatively diced into siRNAs thus explaining why we cannot find long as RNAs. As *RDR2* and *CID2* knockdown lines showed a loss of the strong antisense bias of these siRNAs (arrowheads in Figure 4D), these components appear to be the prime candidates for synthesis of antisense siRNA from long, spliced sense RNA templates in agreement with the conclusions for RDR2 and CID2 in PTGS associated transitivity (43).

Comparing intron and junction mapping reads, a mirror image becomes apparent: junctions showed a higher coverage at the 3′-end of the *ND169* gene, whereas introns showed higher and constantly decreasing coverage from the 5′- to the 3′-end. Especially junction nos. 1 and 2 showed very weak coverage (Figure 4D). Interestingly, intron mapping reads did not show a clear antisense bias in all analysed cultures which therefore distinguishes them from the total bulk of antisense 1° siRNAs. Contrarily, junction mapping reads showed the clear antisense bias and again here, only silencing of *RDR2* and *CID2* altered the antisense ratio of these junction mapping reads (arrowheads in Figure 4D) indicating that these two components are indeed responsible for antisense siRNA accumulation from spliced sense RNAs. Therefore, the general distribution and antisense bias of intron and junction mapping siRNAs and their different behaviour upon *RDR2* and CID2 silencing suggests differential processing of two classes of long RNA transcripts. Full length and spliced transcripts are processed by the RDR2/CID2 into predominantly antisense siRNAs and un-spliced 5′-nascent transcripts are processed differently, presumably by RDR3.

Thus, transgene siRNA accumulation appears to depend on two different sets of RNAi components, showing preferences for different RNA-templates. Importantly, both classes and both pathways depend on each other, because this is the only explanation why knockdown of any RNAi component results in alterations of all siRNA classes.

### 1° siRNA accumulation depends on two sets of RNAi components

**Figure 5.**
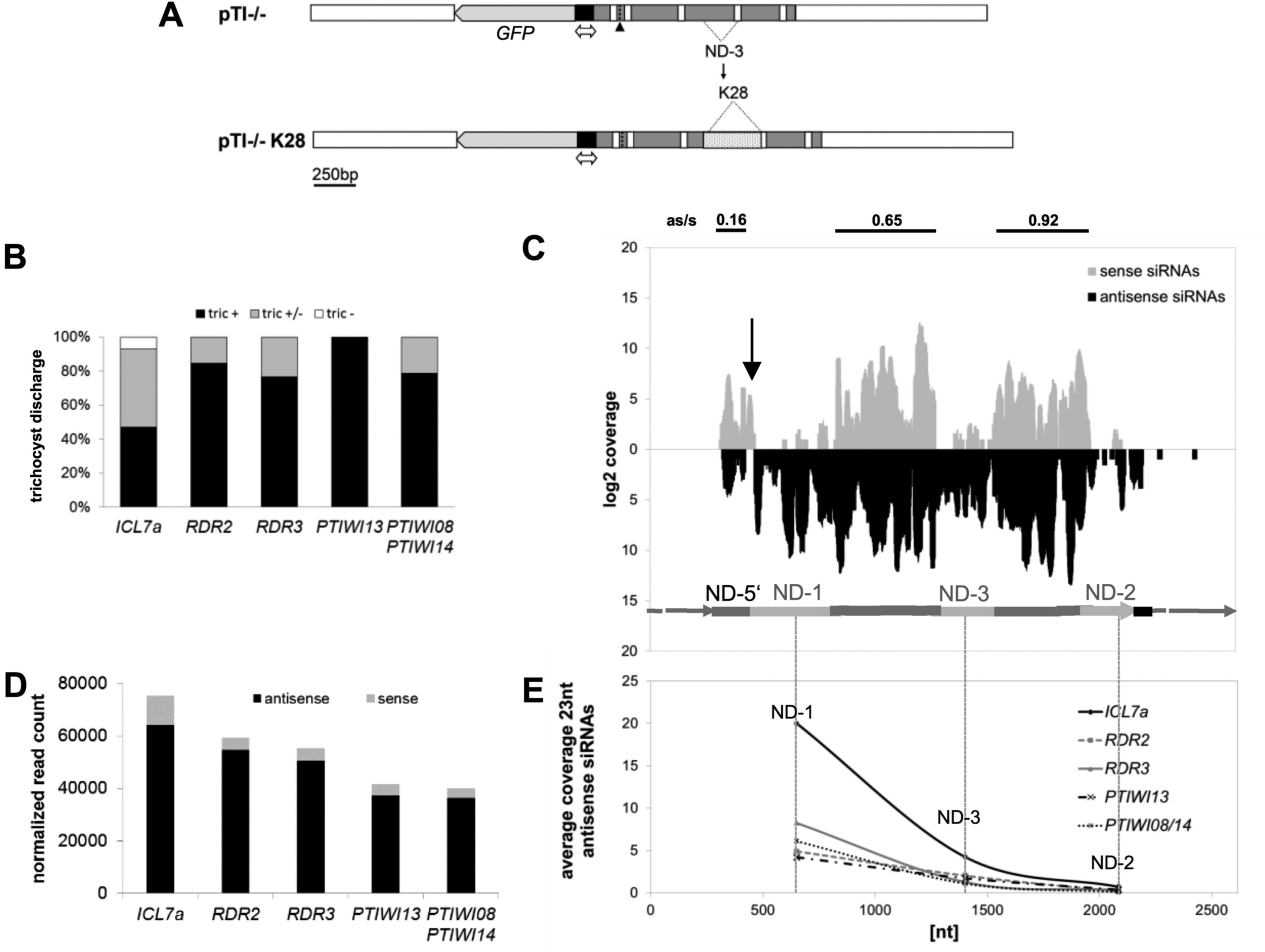
RDR2 & RDR3 and PTIWI13 & PTIWI08/14 are necessary for primary siRNA accumulation. (**A**) A third fragment of the *ND169* central region (ND-3) was removed from the pTI-/- construct and replaced by a fragment of the yeast K28 gene (K28). (**B**) pTI-/- K28 injected clones expressing GFP at similar levels as pTI-/- clones displayed only partial *ND169* silencing, as ∼50% of cells remained tric+. Knockdown of RNAi components lead to almost complete reversion of the transgene-induced phenotype. (**C**) Coverage plot of small RNAs from pTI-/- K28 injected cells (*ICL7a* control dsRNA feeding) revealed accumulation of predominantly 23nt siRNAs mapping to the transgene sequence and to endogenous *ND169*-specific regions (ND-1, ND3, ND-2). (**D**) Average coverage of 2° siRNAs in the three endogenous *ND169* regions in reference to the position in the *ND169* gene. Average coverage of 23nt antisense siRNAs mapping to the respective region are depicted for its mid position. (**E**) SiRNA levels of the K28 transgene region representing 1° siRNAs were reduced upon knockdown of *RDR2, RDR3, PTIWI13* and *PTIWI08/14*.

To foster the latter conclusion that two sets of RNAi components (two PTIWIs and two RDRs) are involved in 1° siRNA accumulation and to get a deeper insight into 2° siRNA synthesis, we constructed another transgene, pTI-/-K28. For this, we eliminated a third region of *ND169* and replaced it with an exogenous sequence (a fragment of the yeast K28 toxin gene). The new ND-3 region is specific for 2° siRNAs and the transgene K28 region can produce 1° siRNAs exclusively (Figure 5A).

The tric- phenotype of the pTI-/- K28 transgene was usually weaker (at approx. equal copy numbers) compared to both other pTI constructs, meaning that only ∼50% of the cells showed decrease of discharged trichocysts (Figure 5B). This is likely due to a lower number of 1° siRNAs, as the length of the region overlapping the *ND169* sequence is reduced in the pTI-/-K28 transgene.

Importantly, the coverage plot of siRNAs mapping to the K28 transgene sequence (Figure 5C) supports our latter conclusion: the 5′-region again showed the characteristic sense bias and the first intron was exclusively covered by sense siRNAs (arrow in Figure 5C), whereas the junction was covered solely by antisense siRNAs. This clearly shows that siRNAs from both strands have different templates and therefore different accumulation pathways.

The K28 region in the transgene allowed for a quantification of unambiguous 1° siRNAs: knockdown of each of the genes within the two sets *PTIWI13/8-14* and *RDR2/3* (Figure 5D) resulted in the reduction of K28 siRNAs, thus supporting our latter conclusions for 1° siRNAs drawn from pTI-/- lines.

### 2° siRNAs show decreasing coverage along the endo *ND169* ORF

Analyzing 2° siRNAs induced by the pTI-/- K28 transgene, Figure 5E shows the normalized read counts of the three regions specific for 2° siRNAs in the context of their position within the endo *ND169* gene. 2° siRNA coverage decreases along the *ND169* ORF, which is different to PTGS-associated 2° siRNAs. Now, we can rule out that the decreasing coverage could be due to different targeting mechanisms as mentioned above: both, the ND-1 and ND-3 region, can theoretically be targeted by 5′- and 3′-transitivity and thus should be targeted to similar extent by 1° siRNAs.

Figure 5E moreover indicates strong reduction of 2° siRNAs in all knockdown lines. Analyzing read length and strand distribution, 1° K28, as well as 2° ND-1 and ND-3 siRNAs showed the typical 23nt peak, and were predominantly antisense (Supplementary Figure S7). However, this was not always the case for 2° siRNAs of the ND-2 region. While *PTIWI08/14* and *RDR3* knockdowns still showed the 23nt peak, although with reduced siRNA abundance, knockdowns of *PTIWI13* and *RDR2* resulted in accumulation of sense reads of various lengths with a loss of the discrete size of 23nt. This specific effect of *RDR2* (as well as *PTIWI13*, which is also involved in PTGS by dsRNA-feeding) on the ND-2 region but not on the ND-1 and ND-3 region let us again hypothesize that RDR2 together with PTIWI13 have a specific role in 3′-transitivity. Although this effect was not obvious in the pTI-/- lines, silencing of both components showed the strongest reduction in the ND-2 region in all samples, together with CID2 (Figure 4B). It seems conceivable that the less efficient silencing of the pTI-/-K28 construct allows for stronger accumulation of full-length mRNAs from the *ND169* locus that are randomly degraded from the 3′-end when RDR2 does not convert them into siRNAs.

### Injection of truncated transgenes induces heterochromatin formation *in trans*

**Figure 6.**
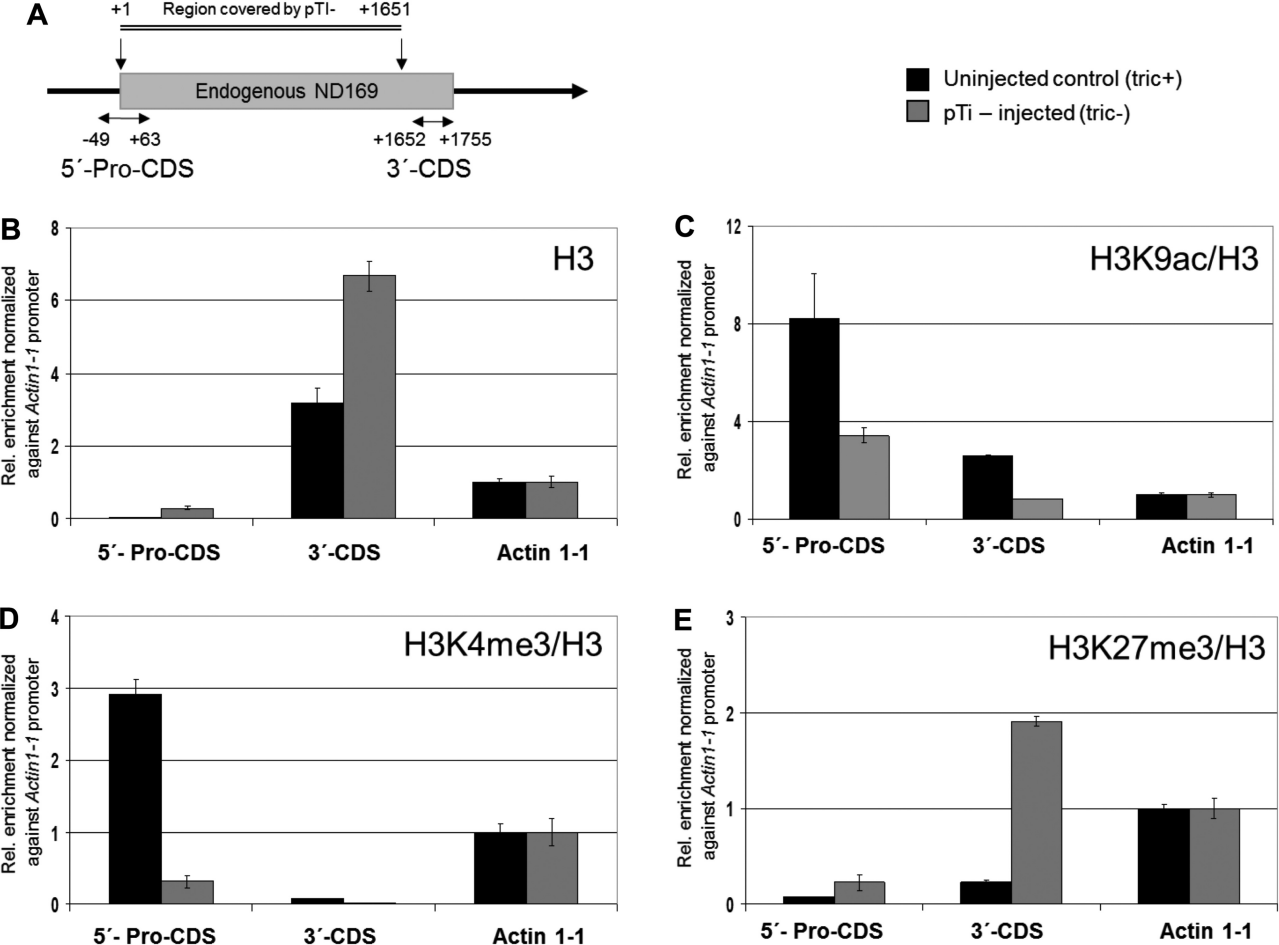
Chromatin remodeling at the endo *ND169* gene locus in transgenic cells. (**A**) Scheme of the two regions probed by ChIP experiments, one covering the promoter and the 5′-part of the CDS and one covering the 3′-part of the CDS corresponding to the ND-2 region. The region covered by the transgene is indicated above. (**B–E**) Chromatin was analyzed from uninjected wild type cells (tric+ phenotype), indicated in black, and pTI- injected cells (tric- phenotype), indicated in grey. All ChIP data are represented as enrichment relative to 10% input DNA and normalized to the native actin 1-1 promoter. Chromatin fragments precipitated with an antibody specific for the unmodified C-terminus of histone H3 were quantified by qPCR to calculate relative enrichment in wild type and transgene-expressing samples (**B**). For detection of histone modifications, chromatin was precipitated with antibodies specific for histone H3 lysine 9 acetylation (**C**), histone H3 lysine 4 trimethylation (**D**), and histone H3 lysine 27 trimethylation (**E**). Relative enrichment to 10% input DNA was normalized to histone H3 occupancy at the specific region and to the native actin 1-1 promoter.

The decreasing coverage of transgene-triggered 2° siRNAs clearly distinguishes these from those occurring in dsRNA-feeding induced PTGS (43). In combination with the involvement of RDR3 with its above-described involvement in TGS of the surface antigen genes it seemed likely that transgene-induced silencing does not act entirely on the post-transcriptional level. To verify this, ChIPs were carried out to identify possible chromatin remodelling going along with silencing of the endogenous *ND169* gene. Figure 6A shows the endogenous *ND169* gene and its overlap with the pTI- construct to show the region chosen for enrichment in ChIP DNA: the promoter including the 5′- CDS and the 3′-CDS. Dynamic chromatin remodelling at the endo *ND169* locus became apparent by an increase of the histone occupancy in the 5′-region and in the 3′-CDS (Figure 6B). Simultaneously, two histone marks which are typically associated with actively transcribed chromatin (H3K9ac and H3K4me3) showed a strong enrichment in promoters when the gene was expressed (un-injected cells) and the pull-down levels were clearly reduced when the gene was silenced (Figure 6C and D). The repressive H3K27me3 mark showed the opposite effect, i.e. a strong enrichment in transgene-injected cultures, especially in the 3′-CDS (Figure 6E). These analyses show the loss of active marks and enrichment of repressive marks at the endogenous locus in transgene-induced silencing.

### Multiple implications for RDR2 mediated siRNA accumulation

RDR2 was indicated to be involved in 2° siRNA accumulation from mRNA templates in PTGS by exogenous dsRNA (43). This function is consistent with our results, suggesting RDR2 to produce small RNAs from full length, spliced transcripts. In addition, another aspect, the independency of efficient pTI- lines to *RDR2* knockdown might suggest, that RDR2 mediated siRNAs represent simply an additional post-transcriptional mechanism, which compensates for inefficient heterochromatin formation and remaining full-length transcription at the transgene and the remote locus.

**Figure 7.**
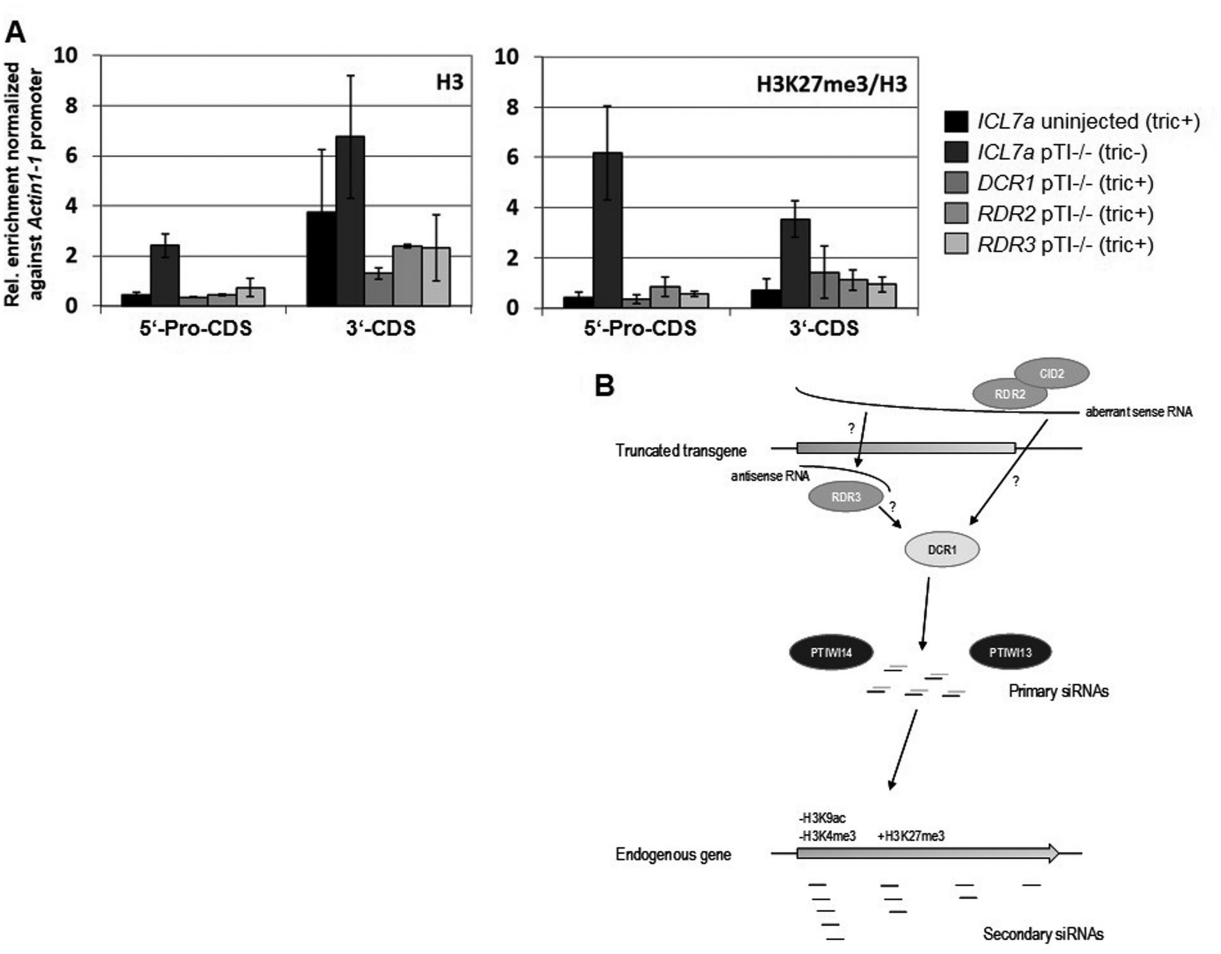
Transgene-induced heterochromatin formation depends on DCR1, RDR2, and RDR3. (**A**) ChIPs were carried out from uninjected wild type cells (subjected to control dsRNA-feeding against *ICL7a*, tric+), pTI-/- injected cells (*ICL7a* feeding, tric-) and the pTi-/- cell line undergoing silencing of *DCR1, RDR2*, or *RDR3* (tric+). All ChIP data represent enrichment relative to 10% input DNA and are normalized to the native actin1-1 promoter. The qPCR primers are the same used for ChIPs in Figure 4. **Left:** Histone occupancy was determined by ChIP with an antibody against the unmodified C-terminus of H3. **Right:** ChIPs with an antibody specific for H3K27 trimethylation. (**B**) A working model of transcription and hypothetical siRNA accumulation pathways. Our data shows that the transgene produces 1° siRNAs which induce dynamic chromatin remodeling *in trans* by loss of H3K9ac/H3K4me3 and increasing levels of H3K27me3 at the endogenous gene. This locus itself produces then 2° siRNAs with clear antisense bias and decreasing levels from the 5′- to the 3′- area of the *ND169* coding region. Although our data does not allow for conclusions about the genetic requirements of 2° siRNAs we can conclude that each of the analyzed RNAi components is required for 1° siRNA production and stabilization. Our data suggest a differential processing of ling and spliced sense transcripts of the transgene by RDR2 and CID2 which may be involved in recognition of such RNA by RNA surveillance mechanisms. Both may also be involved in processing of sense and antisense transcripts in the 5′- area of the transgene together with RDR3.

To test whether RDR2 mediated siRNAs just represent an additional post-transcriptional silencing of transgene- and endogenous transcripts independent of heterochromatin formation, ChIP-experiments were carried out with un-injected and pTI-/- injected lines undergoing control feeding (*ICL7a*) and RNAi against *DCR1, RDR2* and *RDR3*. Figure 7A shows that the pTI-/- construct induces similar effects of increased histone occupancy and increased levels of H3K27me3 at the endo *ND169* locus compared to the pTI- ChIPs in Figure. The only apparent difference between the ChIPs of both transgenes is that pTI-/- ChIPs show a much higher pull-down with the H3K27me3 in the promoter region. These data clearly shows that both, histone occupancy and H3K27me3, depend on RDR2, DCR1, and RDR3, thus indicating that these components are involved in heterochromatin formation. We conclude that RDR2 and associated components do not just contribute additional PTGS, but are also essential for heterochromatin formation *in trans*.

## DISCUSSION

In *Paramecium*, a variety of transgenes (different target genes, truncations, frameshifts) have been used in different studies, demonstrating that non-expressible transgenes injected at high copy numbers cause homology dependent silencing (25–26,44). Our data uncovers the molecular background of siRNA precursors, secondary siRNAs synthesis and heterochromatin formation in this pathway, indicate bidirectional transcription occurring at the transgene locus and, at first glance contradictory, involvement of two RDRs for primary siRNA synthesis.

We demonstrate that transgene-induced silencing caused heterochromatin formation *in trans* at a remote locus. In *S. pombe*, CTGS *in trans* depends on the chromosomal localization of the target genes close to heterochromatic regions (12,13) or requires an additional manipulation of the RNAi system by *ERI1* deletion or over-expression of *SWI6* (13,14). The target gene in our studies (*ND169*) is not located to known heterochromatic loci. However, *trans* silencing requires injection of the triggering transgene construct at high copy numbers (26). It seems reasonable that the abundance of 1° siRNAs is a crucial factor overcoming a potential gene position effect in *Paramecium*. Telomeres are the only constitutive heterochromatic regions in the ciliate acentromeric Mac chromosomes and we reported a telomere position effect as a prerequisite for endogenous RDR3-mediated RNAi of surface antigen genes (23). It is likely that efficient heterochromatin formation requires either a certain abundance of triggering siRNAs or proximity to a heterochromatic region. However, our data also shows that silencing is a quantitative phenomenon, which is linked to transgene copy number and primary siRNA abundance: the comparison of transgene lines with different copy numbers show low numbers of 2° siRNAs even in transgene lines of ∼2x of the endogenous copy number (Supplementary Figure S5). Silencing by truncated transgenes has apparently no threshold and the occurrence of 2° siRNAs in this low copy number transgene line indicates that similar mechanisms could work for endogenous genes.

### Requirements for primary transgene-induced siRNAs

Our results show that all analysed RNAi components are involved in 1° siRNA accumulation. Therefore, 2° siRNAs may have either entirely different genetic requirements, or the same as primaries. Although we cannot dissect individual components of 2° siRNA accumulation, the similarities of 1° and 2° siRNAs let us hypothesize that the very same mechanism acts on the transgene and at the endogenous *ND169* locus. Differences in e.g. antisense bias may be due to different precursor molecules because the remote locus is likely affected by silencing of the transcriptional elongation (see below).

Compared to *S.pombe* which possesses only one Dicer, one RDR and one Ago, the genome of *Paramecium* reveals eight Dicers (19,45), four RDRs (22) and seventeen Piwi (ref. (20) and M. Simon, unpublished) isoforms. In *S. pombe*, at least for Ago, an involvement in CTGS and PTGS was described (46).

The altered antisense ratio of siRNAs in *RDR2, CID2* knockdown lines, suggests that these components are predominantly associated with antisense siRNAs. This is supported by the role of RDR2 in accumulation of PTGS associated 2° siRNAs, which are predominantly antisense to the mRNA (43). CID2 is a nucleotidyltransferase family member involved in RNA surveillance mechanisms: in yeast, CID12 interacts with the RITS (RNA-induced transcriptional silencing complex) and therefore connects RNA surveillance and RNA mediated heterochromatin formation (47). Thus, the borders between clear TGS and PTGS blur indeed in the light of increasing number of studies demonstrating RNA surveillance mechanism associated with heterochromatin formation. These include e.g. the recruitment of RITS and associated RNA surveillance mechanisms to silence meiosis specific genes (48), but it was interestingly also shown, that the exosome can induce heterochromatin in an RNAi independent manner (49).

In *Tetrahymena*, a single RDR associates in complexes (RDRCs) with different nucleotidyltransferase subunits and *in vitro* experiments suggest that uridinylation at the 3′-end of primary transcripts support conversion into dsRNA by RDR (50). Consequently, it seems logic that CID2 recognizes the aberrant transgene transcripts to initiates the silencing cascade and RDR2-mediated synthesis of RNA complementary to spliced transcripts. This would be the same action as reported for RDR2 in exogenously triggered PTGS (43), as well as in our example on the endogenous *ND169*: in the two latter cases, the RDR2 template represents intact mRNA, which is targeted by 1° siRNAs. Further support for our hypothesis comes from the Piwi domain architecture: as PTIWI13 is the only Piwi involved with a potential Slicer activity (20), it makes sense that this Piwi is associated with accumulation of antisense siRNAs to attack sense transcripts. Thus, implication of RNAi components as well as their associated siRNAs let us hypothesize that the set of RDR2, CID2 and PTIWI13 are indeed involved in a PTGS, or PTGS similar mechanism here. With respect to the diversification of *Paramecium* RNAi, it would have been surprising if this set would have an entirely distinct role here compared to their role in PTGS.

In addition, un-spliced transcripts contribute to 1° siRNA accumulation and as the above-described RDR2-mediated mechanism has apparently less influence on siRNAs from nascent transcripts, we cannot directly assign individual RNAi components to their accumulation. We can speculate that RDR3 and PTIWI14, which have until now been exclusively described to be involved in the transgene-induced RNAi, are responsible for their accumulation (Figure 7B). Nascent sense transcripts may directly form dsRNA with antisense transcripts. As RDR3 has a highly divergent domain lacking conservative residues, it may be catalytically dead but could be necessary to mediate hybridization of the two strands rather than synthesizing new RNA. However, in this scenario Dicer activity would need to cut the dsRNA before loading siRNAs into PTIWI14 (see model Figure 7B). In this case, the antisense bias of transgene siRNAs would then be the result preferential loading and stabilization by PTIWI proteins. This hypothesis in fostered by the involvement of RDR3 in transcriptional silencing of the surface antigen gene family (22,23) which is accompanied also by accumulation of sense siRNAs but here from active loci (unpublished data).

Our data shows that a PTGS aspect is involved in transgene-induced heterochromatin formation. A similar hypothesis was made for flies and fission yeast, that RNAi pathways that modify chromatin by targeting nascent RNA need an additional post-transcriptional mechanism to ensure efficient silencing (10). According to our results, involvement of RDR2 does not simply provide additional PTGS, because RDR2 knockdown resulted in deficiency of heterochromatin formation at the endo*ND169* locus (Figure 7A). We conclude that both silencing modes indeed interact with each other rather than to be a consequence of one another, because this is the only explanation why silencing of any component reduced all individual classes of siRNAs (sense/antisense & introns/junctions) to similar extent. Although we cannot exclude that individual components may be involved in both, CTGS and PTGS, it seems more likely that both pathways are functionally linked this may occur on the RNA level if antisense siRNAs are triggered by sense RNAs (and vice versa). In agreement with the above.

### Does the PTGS factor in transgene-induced silencing limit transitivity?

Transitivity occurs in many systems such as siRNA-mediated PTGS in *Arabidopsis* or *C. elegans* and CTGS in fission yeast (12,51). Our results indicate that transitivity is strictly limited to the transcribed region of the *ND169* gene and that the coverage of 2° siRNAs decreases from the 5′- to the 3′-end of the CDS. The decreasing coverage of 2° siRNAs can be explained by the nascent transcript model. PolII would initiate transcription, and then nascent transcripts can be attacked by 1° siRNAs giving rise to 2° siRNAs. As the transgene sequence cannot produce siRNAs that fit to the endo *ND169* promoter, silencing occurs likely at the step of elongation rather than transcriptional initiation.

If we apply the above-hypothesized model to 2° siRNAs assuming that the very same mechanism acts at the remote locus, the association of RDR2, CID2, and PTIWI13 would also be consistent with our findings. The observed 3′-transitivity in the ND-2 region, which cannot be initiated by downstream binding of 1° siRNAs, can only be due to RDR activity. The prime candidate for this would be RDR2, as it is the only catalytic RDR involved in the mechanism, and its knockdown had the strongest influence on the ND2-region. In addition, copying of full-length transcripts by RDR activity is confirmed by the presence of siRNAs antisense to exon-exon junctions of *ND169*. Our hypothesis that RDR2 is responsible for this is based on our finding that its knockdown had a stronger influence to this region compared to all other RNAi components, that this region shows the strongest antisense bias and finally, that RDR2 is responsible for 3′-transitivity in PTGS (43).

Another interesting aspect are the margins for transitivity: as 2° siRNA accumulation is limited to the transcribed region of the *ND169* gene, the native mRNA transcription start and stop sites appear to limit the siRNA precursor transcription. Consequently, this can only be a sense transcript. This is different to recent reports of piRNA-mediated TGS at loci harbouring transposable elements in flies where silencing spreads into flanking protein coding genes (52). Our data indicate that the involvement of PTGS components may be a tool to restrict transitivity to one individual gene. This would be an important mechanism in the highly condensed *Paramecium* Mac genome to avoid side effects of siRNA-mediated heterochromatin formation.

### Implications of transgene-induced silencing to endogenous gene regulation and inheritance

Across kingdoms, most studies investigating *trans* silencing use hairpin constructs to apply directly a dsRNA fragment to induce RNAi. This means that a dsRNA triggering RNAi is directly present. Alternatively, 2° siRNA accumulation can be triggered by piRNAs in *C. elegans*: in a transgene model, these 2° siRNAs were shown to act in trans on another transgene thus triggering heritable silencing and 3° siRNA accumulation in trans at a homologous region in a paramutation like manner (53). In our study, it remains unclear what the direct precursor is. We show that mRNA transcription impairs siRNA synthesis, letting us assume that an RNA surveillance mechanism is involved as discussed above. Similarly, also a pseudogene could trigger siRNA accumulation. On the other hand, the data shows clearly that a mRNA producing locus can also be converted into a siRNA producing locus by *trans* acting siRNAs.

In general, the importance of small RNA-producing pseudogenes emerged earlier for the understanding of the evolutionary impact of gene duplication as well as our current understanding of gene regulation *in trans* and the recent genome wide siRNA annotations across kingdoms reveal more and more siRNA producing pseudogenes which moreover can regulate their parental coding genes (reviewed in ref. (54)). In the unicellular pathogen *Trypanosoma*, triggering dsRNA and siRNA was recently shown to be formed by two pseudogenes with partially complementary sequences as well as by dsRNA hybrids by pseudo- and coding genes, especially the latter possibility was shown to occur on pseudogenes of the VSG-family (variant surface glycoprotein) (55). Also in *Paramecium*, our microarray analysis of *RDR3* knockdown cultures showed that the surface antigen multigene family is affected (23). This multigene family which is responsible for antigenic variation showing some similarity to that of pathogenic protists (56), was also shown to consist of intrachromosomal gene duplicates (23) whose contribution to the gene expression mechanisms has to be clarified. However, a genome wide analysis of pseudogenes and siRNA clusters is right now missing in this organism. One exception is a siRNA cluster on chromosome22 formed by two overlapping orfs and we can show that these siRNAs depend on all components of the transgene RNAi pathway except RDR3 (Supplementary Figure S6C).

With this study, we contribute more knowledge on detailed mechanisms how non-coding genes can produce siRNAs without requiring the formation of hairpin precursors. As indicated by a recent transcriptome analyses, siRNA-mediated heterochromatin formation might not only contribute to the regulation of a large number genes, but also to an epigenetically determined robustness counteracting rapid transcriptome dynamics (24).

It remains to be noted that the siRNAs described here, as well as their endogenous counterparts, may be responsible for transgenerational inheritance of gene expression: transgene-induced silencing as well as PTGS by dsRNA feeding was described to cause deletions of homologous DNA sequences during the development of a new Mac in the sexual progeny (26). However, the authors discuss whether this is a direct effect of the 23nt siRNAs or whether they act indirectly by targeting maternal transcripts. The latter normally specifically hybridize to scnRNAs, which mediate the deletion of sequences not determined to be retained in the in F1 Mac, thus 23nt siRNA may counteract the deletion protecting maternal transcripts (26).

Although we cannot answer this question, we know that at least DCR1 and RDR3 are also involved in accumulation of 23nt siRNAs of surface antigen genes (Simon M, unpublished data). As this multigene family can pass on its expression pattern to sexual progeny (18) without causing a gene deletion effect it seems likely that 23nt siRNAs can transfer gene expression patterns, by determining chromatin states, to the developmental Mac. PTIWI08 may be a good candidate for this process: as it is the ohnolog of PTIWI14 (sharing a high degree of homology) and it is strongly up-regulated during autogamy (20), it may load the same siRNAs as PTIWI14, transferring them to the zygotic nucleus.

## Supplementary Material

SUPPLEMENTARY DATA
