# Supplementary material for: Two sets of RNAi components are required for heterochromatin formation in trans triggered by truncated transgenes
Source: Nucleic Acids Res. 2016 Apr 16;44(12):5908–23. doi: 10.1093/nar/gkw267 (PMC4937312; doi:10.1093/nar/gkw267)
Supplement: SUPPLEMENTARY DATA [file supp_44_12_5908__index.html]

Two sets of RNAi components are required for heterochromatin formation in trans triggered by truncated transgenes — Two sets of RNAi components are required for heterochromatin formation in trans triggered by truncated transgenes — SUPPLEMENTARY DATA 

# Two sets of RNAi components are required for heterochromatin formation *in trans* triggered by truncated transgenes

## SUPPLEMENTARY DATA

- SUPPLEMENTARY DATA
- SUPPLEMENTARY DATA
- SUPPLEMENTARY DATA
