# Supplementary material for: Two sets of RNAi components are required for heterochromatin formation in trans triggered by truncated transgenes
Source: Nucleic Acids Res. 2016 Apr 16;44(12):5908–23. doi: 10.1093/nar/gkw267 (PMC4937312; doi:10.1093/nar/gkw267)
Supplement: SUPPLEMENTARY DATA [file supp_gkw267_nar-00543-y-2016-File010.pdf]

Supplementary data

Two sets of RNAi components are required for heterochromatin formation *in trans* triggered by truncated transgenes

Götz *et al.*

Supplementary Table S1

Supplementary Figures S1A-S1B-S2-S3-S4-S5-S6-S7-S8

Supplementary Table S1

		Sense introns					Antisense Introns	
		1	2	3	4	5	1	2
pTI-/- polyA-enriched	sense	95% (291/306)	81% (149/184)	88% (396/449)	37% (81/220)	92% (253/275)	0% (0/190)	0% (0/449)
	antisense	0.8% (1/120)	1.6% (1/63)	0% (0/25)	6.7% (2/30)	0% (0/10)	4.3 % (2/46)	30% (9/30)
pTI-/- Non-polyA enriched ribo-depleted	sense	83% (97/117)	71% (55/77)	73% (111/153)	40% (34/85)	83% (33/40)	0% (0/76)	0.7% (1/138)
	antisense	0 % (0/21)	0% (0/12)	0% (0/6)	0% (0/2)	11% (1/9)	0% (0/12)	20% (5/25)
Wild type polyA-enriched	sense	98% (108/110)	93% (25/27)	91% (63/69)	78% (51/65)	90% (36/40)	0% (0/29)	0% (0/85)
	antisense	50% (3/6)	0% (0/1)	25% (1/4)	0% (0/8)	100% (2/2)	0% (0/0)	0% (0/2)

Supplementary Table S1: Splice rates of sense and antisense *ND169* introns in sense and antisense RNA-Seq reads. Splicing rates are given as percentage, and raw read numbers below.

Supplementary Figure S1A

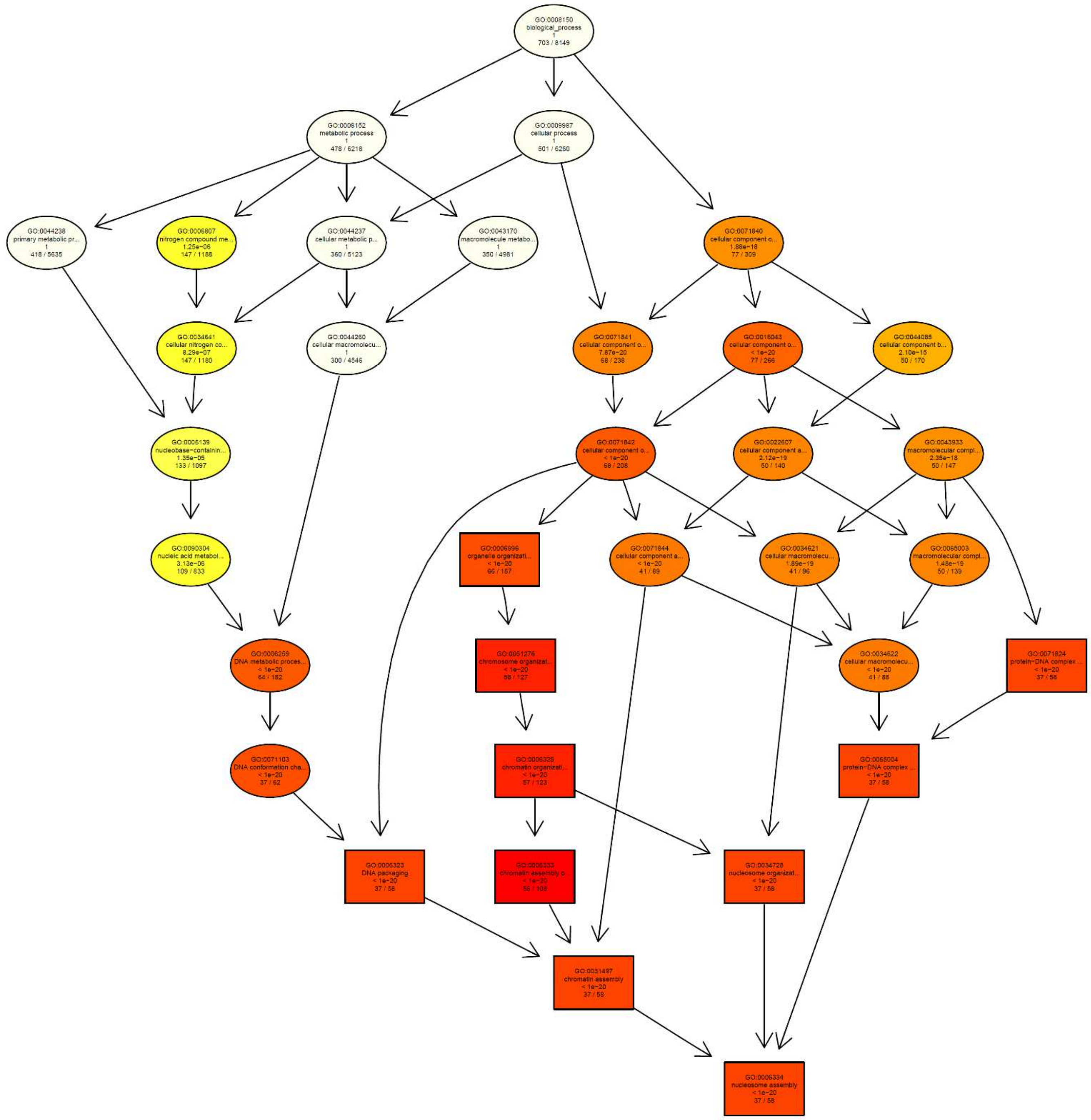

Supplementary Fig. S1A: Hierarchical graph of downregulated GO terms found by gene enrichment on differentially expressed genes in RDR3 silenced cultures. Rectangles indicate the 10 most significant terms (relative significance ranging from red to yellow (most significant to lower significance). Nodes indicate the GO identifier and name, the raw p-value and below the number significantly regulated genes out of the total number of annotated genes per GO term.

Supplementary Figure S1B

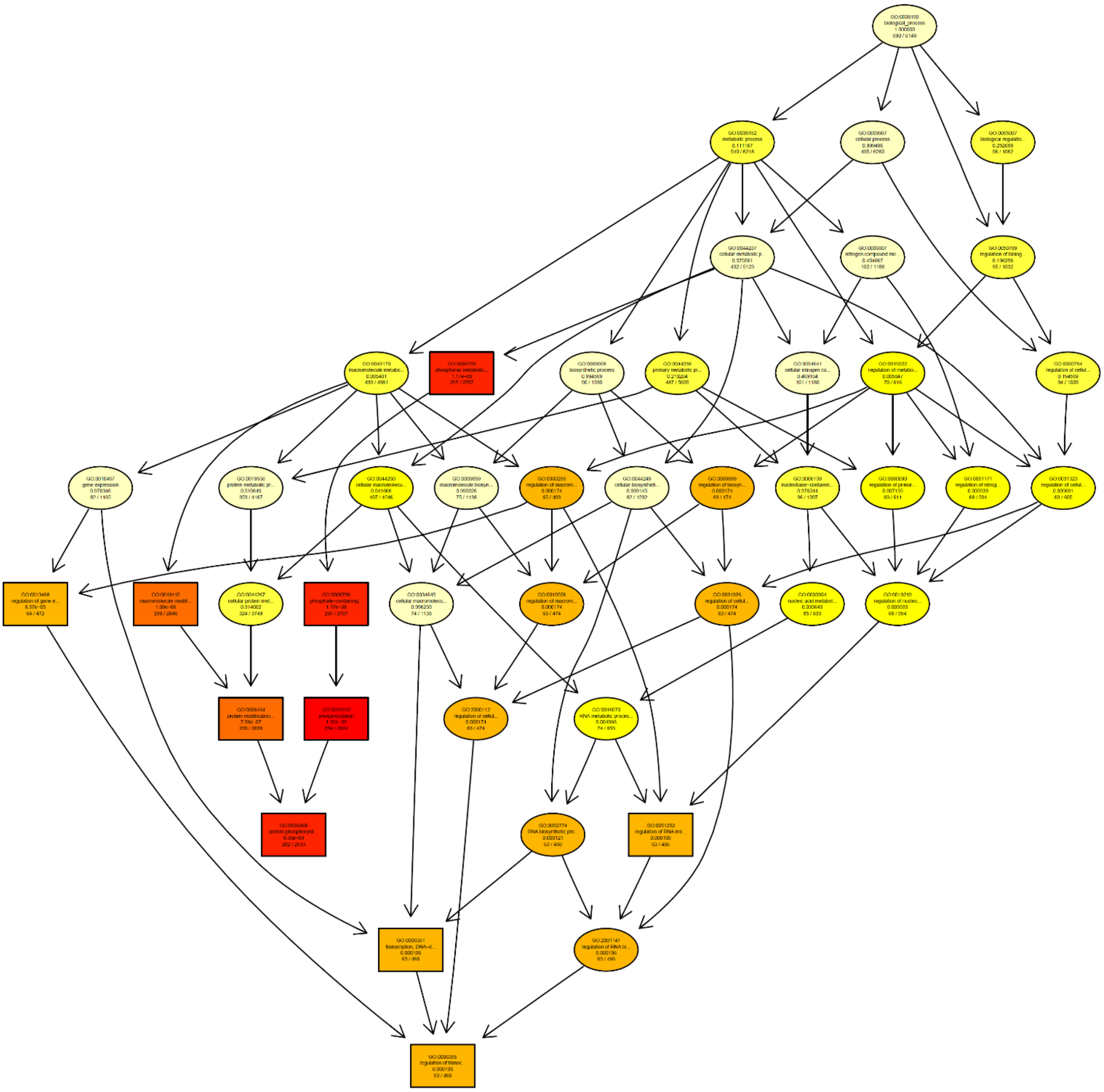

Supplementary Fig. S1B: Hierarchical graph of upregulated GO terms found by gene enrichment on differentially expressed genes in RDR3 silenced cultures. Rectangles indicate the 10 most significant terms (relative significance ranging from red to yellow (most significant to lower significance). Nodes indicate the GO identifier and name, the raw p-value and below the number significantly regulated genes out of the total number of annotated genes per GO term.

Supplementary Figure S2

Götz *et al.*

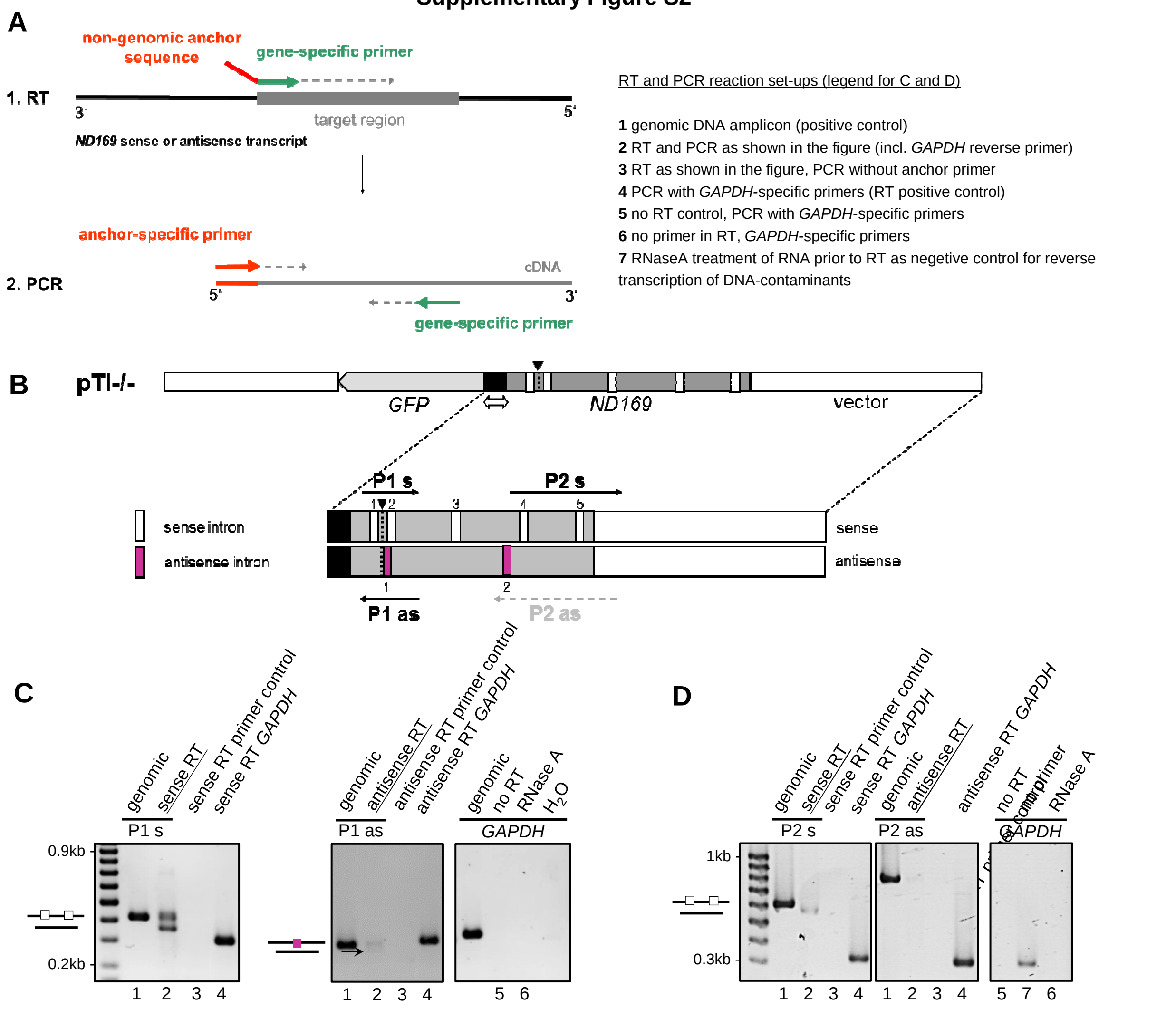

Supplementary Fig. S2: Splicing of antisense introns occurs in transgene-derived antisense transcripts. (A) Strand-specific RT-PCRs were performed to determine splicing events in sense and antisense transcripts of the pTI-/- transgene. Despite using stringent reaction conditions, un-primed cDNA synthesis was detected (see (C)). Therefore, in order to ensure strand-specific reverse transcription (RT), sequence-specific RT primers elongated with an anchor sequence for PCR amplification were used . **(B)** Map of sense and antisense introns in pTI-/- transcripts. Strand-specific RT-PCRs of regions P1 and P2 were performed on pTI-/- RNA samples. Arrows indicate orientation of reverse transcription. To discriminate transcripts of the transgene from those transcribed from the endogenous *ND169*, region P1 was defined to overlap the artificial ND-1 junction (black arrowhead) and P2 to overlap the plasmid vector. Note that the P1 primerset and PCR conditions were designed to allow amplification of the longer endogenous product. However, the endogenous product was not detected in the presence of the transgene (C and D). **(C)** Strand-specific RT-PCR products and controls of region P1. A genomic DNA amplicon containing the anchor sequence was generated by PCR and used as a positive control (1). Both, sense and antisense transcripts of region P1 were detected (2). The antisense product consisted of a upper band representing the unspliced form, and a weak lower band (black arrow), representing the form in which antisense-intron 1 was spliced. Sequencing of bulk and cloned PCR products confirmed splicing of sense intron 1 and 2, and of antisense intron 1. Note that cloning of the antisense product was performed for both bands together and consistently, antisense intron 1 was only found spliced in a minor fraction of the cloned products. False positive amplification driven by the gene-specific part of the RT primer and the oppositely directed PCR primer could be excluded, as no product was obtained when only the latter PCR primer and not the anchor primer was used in the PCR (3). A *GAPDH* gene-specific reverse primer was added to each RT reaction and *GAPDH* transcripts were amplified as a control for successful cDNA synthesis (4). Successful removal of genomic DNA from RNA samples was verified by cDNA synthesis without RT enzyme (5). RNA was treated with RNase A prior to cDNA synthesis to check for false positive amplification due to reverse transcription of traces of genomic DNA, introducing the anchor sequence (6). **(D)** Strand-specific RT-PCR products and controls of region P2. Splicing of both introns in the detected sense product was confirmed by bulk sequencing; no product was obtained from antisense amplification (2). Amplification of *GAPDH* from an RT reaction without primer confirmed a background level of un-primed cDNA synthesis (7). Antisense intron 2 was detected by directional RNA-Seq only.

Supplementary Figure S3

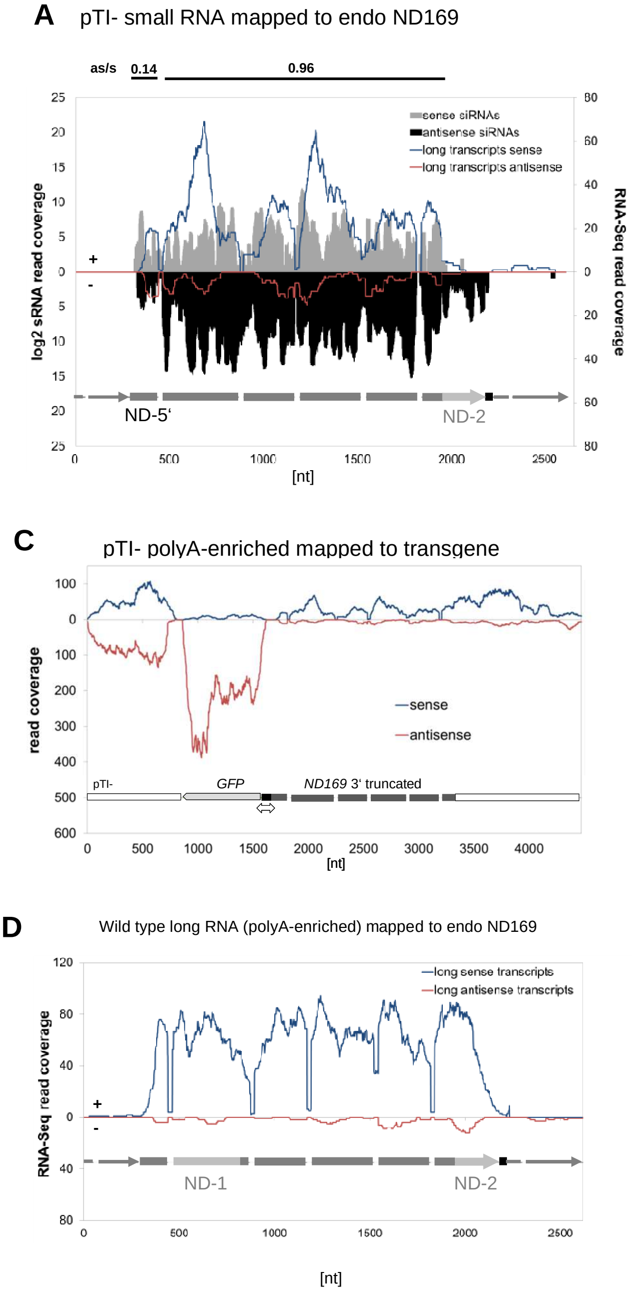

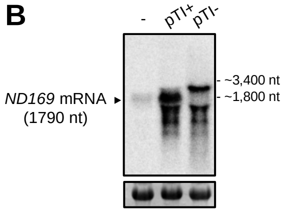

Supplementary Fig. S3: Transcripts and small RNAs induced by the pTI- transgene.
SiRNA reads from pTI- injected cells were mapped to the endogenous *ND169* gene. 23nt sense siRNA levels are shown in grey, antisense in black. The *ND169* gene and parts of the flanking regions are indicated above the X-axis. Introns are represented by gaps. The light grey bar represents the ND-2 region not present in the transgene, indicating secondary siRNA production from the endogenous *ND169* locus. On top, the ratio of antisense to sense 23nt siRNAs is indicated for the 5'-region of the *ND169* CDS (ND-5') and for the NDgene region. Long transcripts revealed by poly-A enriched RNA-seq are depicted in blue (sense) and red (antisense). **(B)** The Northern blot shows *ND169* transcripts in WT and pTI- injected cell lines. pTI+ induced overexpression of *ND169* mRNA (black arrowhead), whereas pTI- lines accumulated transcripts of aberrant size, which fit approx. to the distance of promoter to the linearization site. A double-stranded probe covering 726 nt of the *ND169* 5'-CDS was used for hybridisation. The 17S rRNA is shown as a loading control. **(C)** Directional RNA-Seq of polyA-enriched RNA from pTI- cells. The coverage of each nucleotide position with transcripts from the top (+) strand (blue) and bottom (-) strand (red) is shown. The *GFP* orf (bottom strand) is indicated by the light grey arrow, the truncated *ND169* orf by a dark grey bar, with gaps representing introns. Transcription of both is driven by the same bidirectional promoter (black). **(D)** Coverage plot of polyA-enriched transcripts mapping to the endogenous *ND169* gene in wild type cells (control). The ND-1 and ND-2 regions lacking in the transgenes pTI- and pTI-/- are indicated in light grey.

Supplementary Figure S4

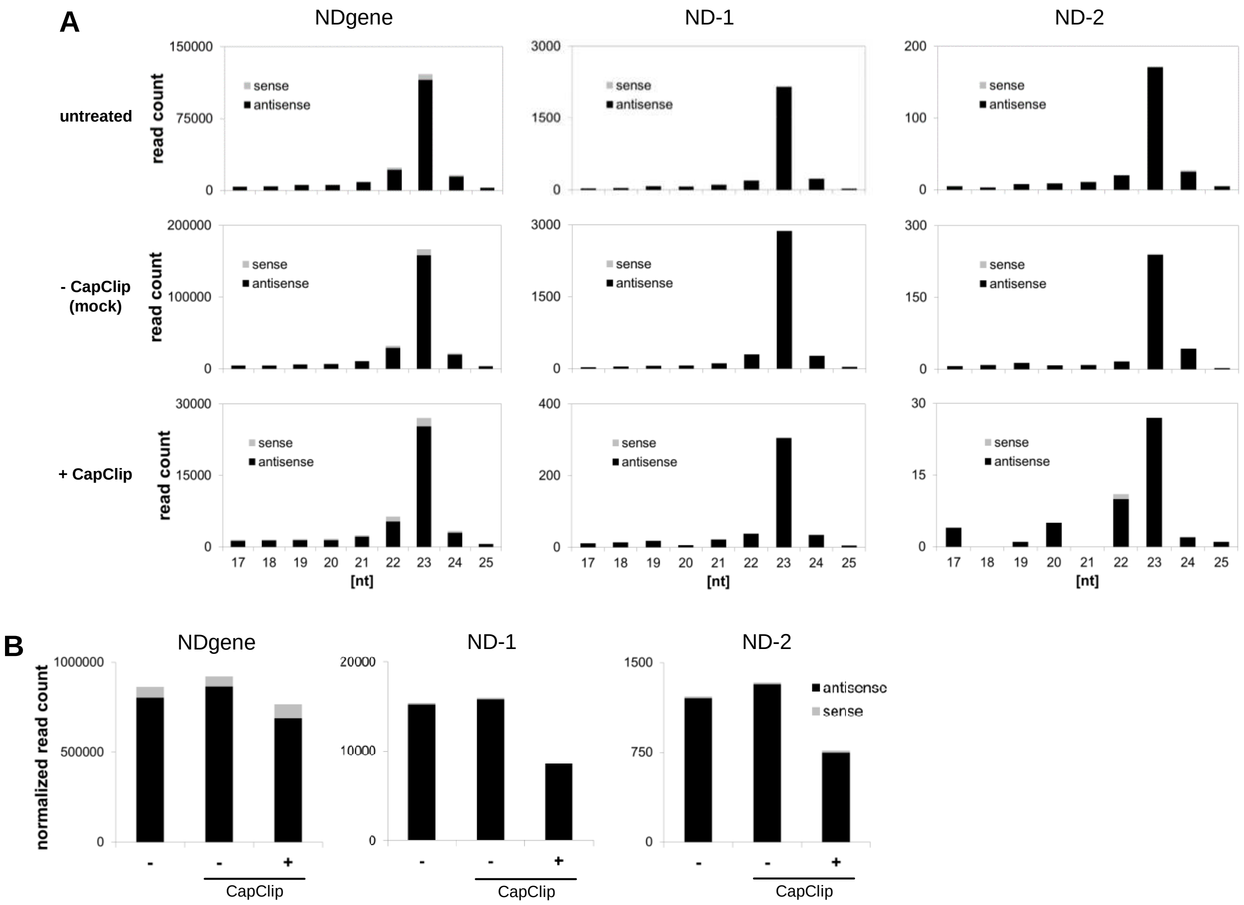

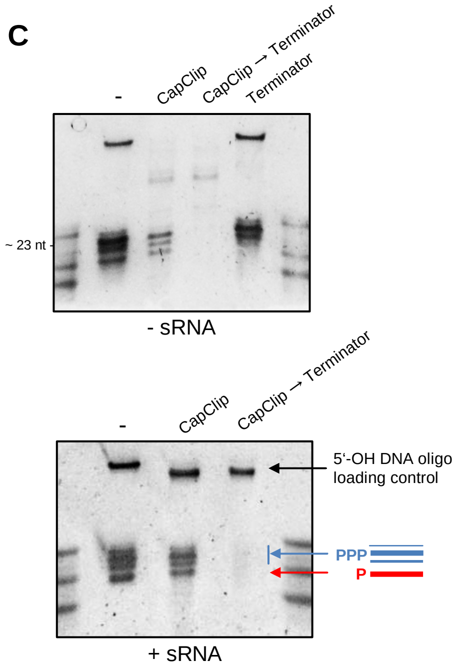

Supplementary Fig. S4 : Transgene-induced, transitive secondary siRNAs are predominantly 5'-monophosphorylated. Small RNA libraries were produced after treatment of pTI-/- sRNA samples with a pyrophosphatase, in order to enrich for siRNA species carrying a 5'-mono- or tri-phosphate, or a 5' cap (m7GpppG or others). (A) Size distribution of *ND169* siRNAs in an untreated RNA sample (top) and after enzymatic treatment with the Acid Pyrophosphatase Cap-Clip™ (CellScript) (bottom). -CapClip mock (middle): same treatment as +CapClip (see Supplementary methods), but without adding enzyme. Enzymatic treatment did neither alter the size distribution, the strand bias, nor result in sRNA degradation. (B) Normalized read count of *ND169* siRNAs mapping to the region covered by the pTI-/- transgene (NDgene), and to the endogenous regions (ND-1 and ND-2) of which only secondary siRNAs are produced. CapClip treatment did not increase the proportion of transitive secondary siRNAs compared to the total, indicating that this species is predominantly 5-monophosphorylated. (C) Verification of CapClip activity and specificity using oligonucleotides. Upper gel: A 5'-tri-phosphorylated RNA oligo (21nt) was synthesized *in vitro*, gel purified and mixed in equal proportions with a 5'-mono-phosphorylated RNA (21nt) and a 5'-OH DNA oligo (42nt). CapClip treatment of the mix, followed by treatment with the Terminator™ 5´-phosphate-dependent exonuclease (Epicentre), but not Terminator treatment alone degraded this RNA oligo, confirming its 5'-tri-phosphate end. Note that the gel-purified 5'-tri-phosphate RNA migrated as three bands; the DNA control oligo was degraded by CapClip in some cases for unknown reasons. Below: Gel-purified sRNAs (18-24nt fraction) of pTI-/- samples was supplemented with the oligo mix and treated with enzymes in parallel to the samples used for sRNA library construction.

Supplementary Figure S5

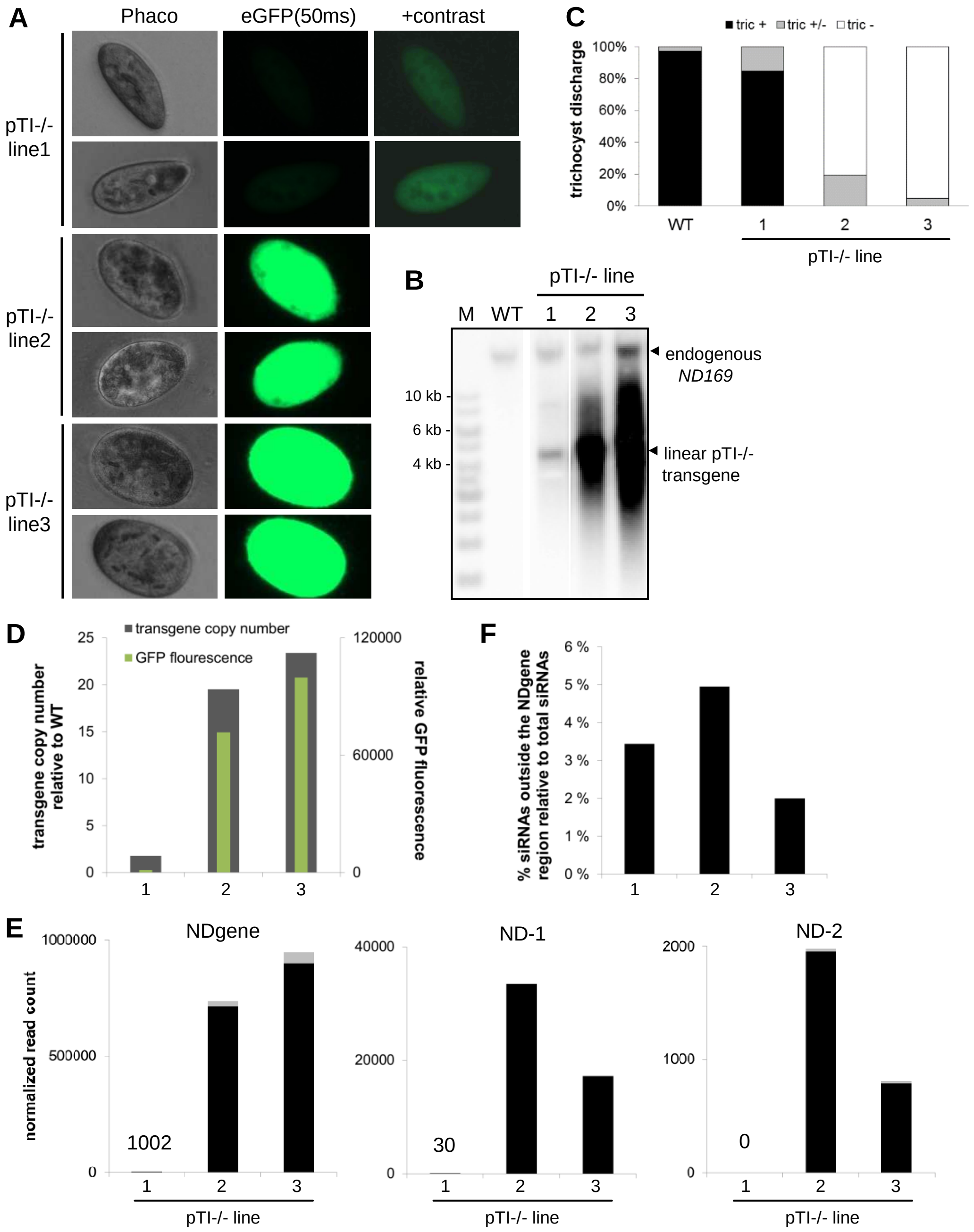

Suppl. Fig. S5 : The abundance of primary siRNAs correlates with the strength of transgene-induced silencing. Three cell lines maintaining different pTI-/- transgene copy numbers were used. **(A)** pTI-/- injected cell lines were selected based GFP expression. **(B)** The relative pTI-/- transgene copy number was determined by Southern blots using a probe mapping to a 700 bp region of the 5´-*ND169* orf. Undigested genomic DNA was used for the blot. In all cell lines the MAC chromosome carrying the endogenous *ND169* gene could be detected. In addition, in injected cell lines the 4120 bp transgene was detected, predominantly maintained as monomeric mini-chromosome. The transgene copy number correlated with the GFP fluorescence (see also (D). **(C)** The two high-copy number pTI-/- injected lines 2 and 3 showed silencing of trichocyst discharge. Non-injected WT cells showed complete discharge. **(D)** Measurement of the green fluorescence intensity of the cells shown in (A) and densitometric quantification of the southern blot shown in (B) revealed direct correlation of the relative transgene copy number with the GFP fluorescence. Fluorescence intensity is given relative to the non-injected WT. Transgene copy numbers are relative to the non-injected WT, normalized to the endogenous gene copy number. **(E)** Small RNA sequencing and mapping to the *ND169* orf showed that the number of siRNAs mapping to the NDgene region (i.e. predominantly primary siRNAs) correlated with the relative transgene copy number. Read numbers are given as numbers for pTI-/- line 1 **(F)** **T**he relative proportion of these secondary siRNAs is given in percentage of NDgene mapping siRNAs.

Supplementary Figure S6

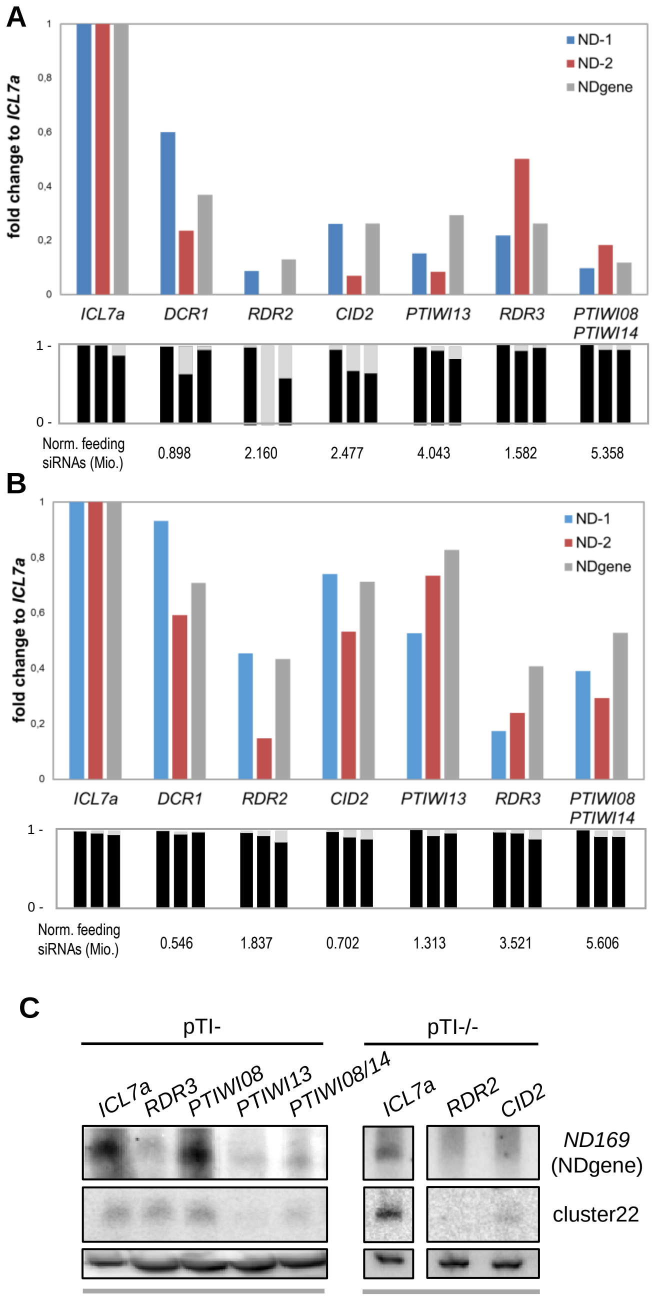

Suppl. Fig. S6 : Comparsion of replicate series of RNAi knock down lines. Foldchanges to the control dsRNA feeding (*ICL7a*) are shown for 23nt siRNAs mapping antisense to the *ND169* endogenous regions ND-1 and ND-2, and to the transgene-covered region NDgene. The replicate with stronger reversion **(A)** of the transgene-induced RNAi phenotype was selected for main Figure 4B. **(B)** The second replicate series with lower fold-changes is shown. Antisense ratios of 23nt siRNAs are given below graphs as well as the normalized number of feeding associated siRNAs (in millions)for the respective gene reflecting silencing efficiency.

(C) Knockdown of specific RNAi components by dsRNA feeding results in reduction or loss of 1° transgene and endogenous cluster22 siRNAs. SiRNAs induced by the transgenes pTI- or pTI-/- are shown on the top panel (NDgene region; probe of the entire *ND169* orf). The same blot was hybridized with single-stranded oligonucleotide probes corresponding to the bottom strand of a transcribed, intergenic region on scaffold 22 (ParameciumDB) producing endogenous siRNAs. SiRNA levels after 72h of dsRNA feeding are shown. Note that silencing of *RDR3* leads to decrease of cluster22 siRNA levels only after prolonged dsRNA feeding (≥ 5 days) 18. Independent knockdown experiments on individual pTI injected clones are shown with their respective *ICL7a* control, as indicated below by grey lines. Glutamine tRNA was probed as a loading control.

Supplementary Figue S7

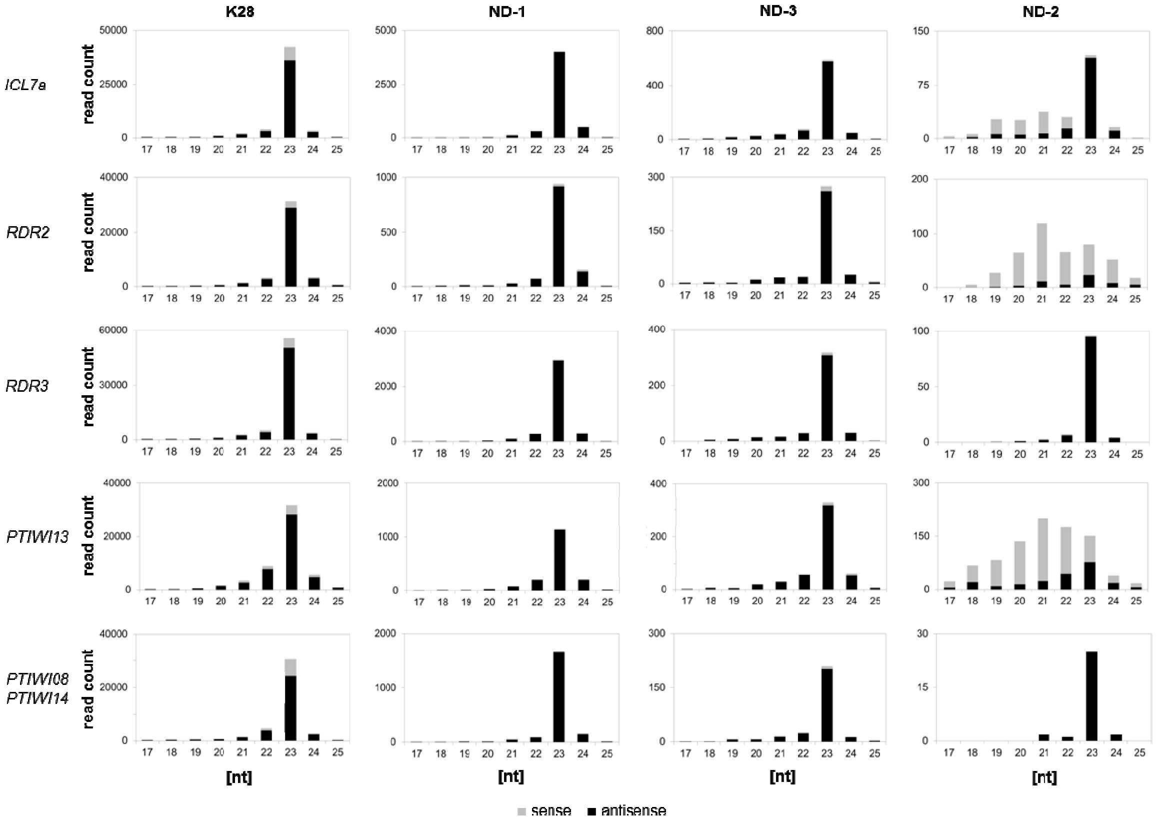

Supplementary Fig. S7: Size distribution of primary siRNAs and secondary trans small RNAs induced by the pTI-/- K28 transgene. Reads mapping to the K28 region (transgene) and to the *ND169* ND-1, ND-3, and ND-2 regions (endogenous) are shown in control (*ICL7a*) and RNAi knockdown samples. Experimental setup see main figure 6.

Supplementary Figue S8

Sample	total reads (17-25nt)	reads aligned to MAC 51 genome (including transgene)
A-pTI-/- ICL	18.695.851	3.406.353
B-pTI-/- ICL	12.484.217	4.857.575
C-pTI-/- ICL	12.350.699	3.253.304
D-pTI-/- ICL	32.913.357	10.216.708
A-DCR 1 (R2)	10.050.388	3.753.640
D-DCR 1 (R1)	10.881.363	1.354.109
A-RDR2 (R1)	12.302.816	2.117.962
B-RDR2 (R2)	14.232.099	4.219.013
A-CID2 (R1)	21.911.056	2.738.905
B-CID2 (R2)	15.478.934	5.973.354
A-PTIWI13 (R2)	14.003.351	4.422.801
B-PTIWI13 (R1)	12.340.151	5.143.494
B-RDR3 (R2)	16.548.891	5.181.674
C-RDR3 (R1)	12.817.110	3.163.539
B-PTIWI814 (R2)	16.700.829	7.742.105
D-PTIWI814 (R1)	15.295.881	4.582.315
K28 ICL	9.927.068	2.478.683
K28 RDR 2	13.717.468	2.389.681
K28 PTIWI 13	14.261.707	3.704.168
K28 RDR 3	14.218.543	4.319.924
K28 PTIWI 8/14	15.787.800	4.246.360
pTI-/- 1	19.219.389	8.024.320
pTI-/- 2	20.756.970	9.595.972
pTI-/- 3	23.744.861	10.589.670

Supplementary Fig. S8: Read statistics and library composition details of siRNA libraries. The correlation of an RNAi knockdown line to its individual ICL control is indicated by large letters (A-D). (R1) and (R2) in brackets indicates the replicate series shown in Suppl. Fig. S6: (R1) represents the replicate series with better silencing efficiency shown in Suppl. Fig. S6A and (R2) the replicate series with lower silencing efficiency in Suppl. Fig. S6B. The total read number represents reads trimmed for quality, adapters and for a read length between 17-25nt.
