# Supplementary material for: Two sets of RNAi components are required for heterochromatin formation in trans triggered by truncated transgenes
Source: Nucleic Acids Res. 2016 Apr 16;44(12):5908–23. doi: 10.1093/nar/gkw267 (PMC4937312; doi:10.1093/nar/gkw267)
Supplement: SUPPLEMENTARY DATA [file supp_gkw267_nar-00543-y-2016-File011.pdf]

Supplementary methods

Two sets of RNAi components are required for heterochromatin formation *in trans* triggered by truncated transgenes

Götz *et al.*

Material:

- **Primers used for strand-specific RT-PCRs of transgene samples**
- **Primers for ChIP**
- **dsRNA fragment positions for RNAi feeding**
- **Primers and oligonucleotides for probes**

Methods:

- **Strand-specific RT-PCR**
- **Genomic DNA Isolation and Southern Blot**
- **Preparing libraries of 5'- tri- and mono-phosphorylated small RNAs**

Primers used for strand-specific RT-PCRs of transgene samples

Position	strand detection	position of amplicon in pTI-/- transgene	primer name	primer used in	primer sequence (5' to 3')
P1	sense	1721-2115	ND169_701_r2-anchor	reverse transcription	GACTGGAGCACGAGGACACTGAGTTA GAAGAGTGGCCCATTCTCATCTC
			ND169_P1_f2 PCR	PCR (forward)	CTCTGCGATAGGGAGGGGATGG
			anchor	PCR (reverse)	GACTGGAGCACGAGGACACTGAGT
P1	antisense	1721-2006	ND169_P1_f2_anchor	reverse transcription	GACTGGAGCACGAGGACACTGACTCT GCGATAGGGAGGGGATGG
			anchor	PCR (forward)	GACTGGAGCACGAGGACACTGA
			ND169_669_r	PCR (reverse)	GTTCTTCCTCATCGGGGATTTGATC
P2	sense	2437-3018	Tr-Vector_r_anchor_2	reverse transcription	GACTGGAGCACGAGGACACTGACAGC GAGTCAGTGAGCGAGGAAG
			1158_f2	PCR (forward)	CTGTGGATTCTGCTATGAGAGCGAAG
			anchor	PCR (reverse)	GACTGGAGCACGAGGACACTGAGT
P2	antisense	2227-3018	ND169_P2_f2_anchor	reverse transcription	GACTGGAGCACGAGGACACTGAAATG TGGATGGTGCTAAGTGGATGAAG
			anchor	PCR (forward)	GACTGGAGCACGAGGACACTGA
			Tr-Vector_r2	PCR (reverse)	CAGCGAGTCAGTGAGCGAGGAAG

Primers for ChIP

Primer name	Sequence (5'-3')	Position rel. to ATG
ND169 pro-5'cds for	TGTATGAGGTTGACATGTAATTGTGAA	-49
ND169 pro-5'cds rev	AATAACATTTGGCCATCCCCTCC	63
ND169 3' cds for	TGGAATATGTGTTTGAGACATCCA	1652
ND169 3' cds rev	TCCTCAATTTCCTCCTTGTCAAC	1755
Actin1_1 for (1)	AAGGTGAAATAATTGTCATCATAATCA	-62
Actin1_1 rev (1)	CAACAACTGCGGGGAAG	108

DsRNA constructs for gene silencing

Gene	accession number (ParameciumDB)	WGD duplicate	orf size ATG-TGA [bp]	dsRNA fragment (position within orf)	length of dsRNA fragment [bp]	Reference
ND169	GSPATG00008337001	-	1879	1450 – 1860	411	Marker et al. 2010 (2)
ICL7a	GSPATG00021610001	*ICL7b* (GSPATG00023293001)	582	1 – 580	580	Galvani & Sperling 2002 (3)
DCR1	GSPATG00021751001	-	5394	3019 - 3987	969	Lepère et al. 2009 (4)
RDR2	GSPATG00036857001	-	4107	1289 - 1559	271	Marker et al. 2010 (2)
RDR3	GSPATG00006401001	-	3374	1789 – 2462	674	Marker et al. 2010 (2)
CID2	PTETG13400003001	-	1020	390 - 883	494	Marker et al. 2014 (5)
PTIWI13	PTETG4800007001	-	2483	105 - 787	683	Bouhouche et al. 2011 (6)
PTIWI08	GSPATG0002128800	*PTIWI14*	2430	269 – 913	645	Bouhouche et al. 2011 (6)
PTIWI14	PTETG16300003001	*PTIWI08*	2428	285 – 1007	723	Bouhouche et al. 2011 (6)

Primers and oligonucleotides for Northern-probes

Primer name	Sequence (5'-3')	Northern blot
ND169_701f	GATGATGTTACTCTGCGATAGGG	PCR Product for internal labelling
ND169_1733r	TCAAAATCTCTAAAATGCTTTTTCCTT	PCR Product for internal labelling
Cluster22-1	TAGAGTGTAAGCCGAACTAGACTTGACCCA AAAGATAAATATAACTAGAT	Oligo for 5´-labeling
Cluster22-2	ACTTGACCACCTTTTGTCAATGAAGAGAACT TTAGCCAAATTAATACCTT	Oligo for 5´-labeling

Preparing libraries of 5'- tri- and mono-phosphorylated small RNAs

For capturing 5'- tri- and mono-phosphorylated RNAs in the same small RNA library, gel-purified small RNA extracts of 50 µg total RNA (see main text) were treated with the Acid Pyrophosphatase Cap-Clip™ (CELLSCRIPT, Madison, Wisconsin) prior to library construction. It removes a pyrophosphate from 5'-triposphorylated and 5'-capped RNAs (m7GpppG or others), leaving a 5'-monophosphate. The reaction was set up with 1x Cap-Clip™ reaction buffer, 20U Murine RNase-Inhibitor (NEB, Frankfurt, Germany) and 15U Cap-Clip™ enzyme, and incubated at 37°C for 2.5 hours. After purification with phenol (pH 4) and precipitation with ethanol-sodium acetate and 10.5 µg glycogen, the small RNA was dissolved in nuclease-free water and used for library construction as described (see main text). Two control libraries were prepared, one with small RNAs treated identically, but without adding the Cap-Clip™ enzyme, and one with untreated small RNAs.

In order to verify successful Cap-Clip™ treatment, another aliquot of small RNA was supplemented with 200ng of an oligonucleotide mix (see below) and treated as described, using 20U Cap-Clip™. After phenol purification and precipitation, the reaction product was dissolved in water, and two third were further treated with 1U of Terminator™ 5´-monophosphate-dependent exonuclease (Epicentre, Madison, Wisconsin), in 1x reaction buffer A and 20U Murine RNase-Inhibitor for 70 minutes at 30°C. Both samples were precipitated, run on a 17.5% denaturing urea-polyacrylamide gel and oligonucleotides were visualized by SybrGold staining.

The oligonucleotide mix was prepared as follows: A 5'-triphosphorylated RNA oligo (21nt) was synthesized *in vitro* using annealed DNA oligonucleotide templates containing a T7 polymerase promoter sequence (Fermentas High Yield Transcript Aid T7 Kit (Thermo Fisher Scientific, Waltham, Massachusetts), according to the manufacturer's instructions). After DNase I treatment the product was gel-purified and mixed in equal proportions with a gel-purified, commercial 5'-monophosphorylated RNA (21nt) and a 5'-OH DNA oligo (42nt).

Chromosomal DNA Isolation and Southern Blot

Isolation and preparation of intact chromosomes of *Paramecium* cells was carried out to determine transgene and endogenous copy number by Southern blots. 200,000 cells were starved in Volvic ® water to digest bacterial DNA. Cells were centrifuged and resuspended in 0.5M EDTA pH 9, 1% Sarcosyl, 1% SDS, 0.25mg/ml proteinase K followed by incubation at 55°C over night. After extraction with Tris-buffered phenol pH 8, the aqueous phase was dialysed against TE buffer for minimum two days. After this, a final concentration of 20ng/µl RNase A was added for 20 min. After additional phenol extraction, DNA was loaded on a 1% agarose gel and Southern blotted according to standard procedures (including depurination to guarantee for efficient blotting of large chromosomes). Labelling of PCR products and hybridisations (at 60°C) were carried out as described for Northern blots using the same probe for the *ND169* gene covering the full orf.

Strand-specific RT-PCR

8 µg of total RNA were treated with 2.5U of RNase-free DNase I (Qiagen, Hilden, Germany) in1x buffer RDD for 20 minutes at room temperature and then purified by acid phenol (pH 4) and precipitation with ethanol-sodium acetate. To ensure strand-specificity of the reverse transcription, cDNA synthesis was performed using primers fused 5' to the artificial anchor sequence GACTGGAGCACGAGGACACTGA (according to (7), modified). 500 ng DNase I-treated total RNA were reverse transcribed as follows: the RNA was denatured for 3 minutes at 95°C in presence of primers and dNTPs and then chilled on ice, in order to separate sense and antisense strands. Target transcripts were reverse transcribed according the supplier's instructions using 10 pmol of each primer (*ND169* sense or antisense- and *GAPDH* (GSPATG00016902001) sense-specific), 200U of Maxima® Reverse Transcriptase (Thermo Fisher Scientific, Waltham, Massachusetts) and 20U of Murine RNase-Inhibitor in 1x RT buffer for 30 minutes at 57°C. The enzyme was heat-denatured for 5 minutes at 85°C and the cDNA was diluted 1:1 with nuclease-free water. 1 µl of cDNA were used in a 25 µl PCR reaction. PCRs were performed with Q5® High-Fidelity DNA Polymerase (NEB, Frankfurt, Germany) according to the supplier's instructions. An anchor-specific forward primer and a *ND169*-specific reverse primer were used for amplification of antisense transcripts; a *ND169*-specific forward primer and an anchor-specific reverse primer were used for amplification of sense transcripts. *GAPDH* transcripts were amplified in a separate reaction using *GAPDH*-specific forward- and reverse primers.

References for Supplementary Methods

1. Ladenburger, E.M., Sehring, I.M., Korn, I. and Plattner, H. (2009) Novel types of Ca^{2+} channels participate in the secretory cycle of Paramecium cells. *Mol Cell Biol* **29**, 3605-3622.

2. Marker, S., Le Mouël, A., Meyer, E. and Simon, M. (2010) Distinct RNA-dependent RNA polymerases are required for RNAi triggered by double-stranded RNA versus truncated transgenes in Paramecium tetraurelia. *Nucleic Acids Res.* **38**, 4092-4107.

3. Galvani, A. and Sperling, L. (2002) RNA interference by feeding in Paramecium. *Trends Genet* **18**, 11-12.

4. Lepère, G., Nowacki, M., Serrano, V., Gout, J.F., Guglielmi, G., Duharcourt, S. and Meyer, E. (2009) Silencing-associated and meiosis-specific small RNA pathways in Paramecium tetraurelia. *Nucleic Acids Res* **37**, 903-915.

6. Marker, S., Carradec, Q., Tanty, V., Arnaiz, O. and Meyer, E. (2014) A forward genetic screen reveals essential and non-essential RNAi factors in Paramecium tetraurelia. *Nucleic Acids Res* **42**, 7268-7280.

6. Bouhouche, K., Gout, J.F., Kapusta, A., Bétermier, M. and Meyer, E. (2011) Functional specialization of Piwi proteins in Paramecium tetraurelia from post-transcriptional gene silencing to genome remodelling. *Nucleic Acids Res* **39**, 4249-4264.

7. Cho, D.H., Thienes, C.P., Mahoney, S.E., Analau, E., Filippova, G.N. and Tapscott, S.J. (2005) Antisense Transcription and Heterochromatin at the DM1 CTG Repeats Are Constrained by CTCF. *Mol Cell* **20**, 483-489.
