# Supplementary material for: Two sets of RNAi components are required for heterochromatin formation in trans triggered by truncated transgenes
Source: Nucleic Acids Res. 2016 Apr 16;44(12):5908–23. doi: 10.1093/nar/gkw267 (PMC4937312; doi:10.1093/nar/gkw267)
Supplement: SUPPLEMENTARY DATA [file supp_gkw267_nar-00543-y-2016-File012.pdf]

GO 0006333 chromatin assembly or disassembly

ID	coeff.	p-value	signif.	ID	coeff.	p-value	signif.
GSPATT00000182001	1,943	0,00003	1	GSPATT00016556001	-1,26	0,00452	-1
GSPATT00000820001	-0,476	0,01317	0	GSPATT00016929001	-1,277	0,00088	-1
GSPATT00000899001	0,556	0,00363	0	GSPATT00018066001	-3,301	0	-1
GSPATT00000918001	0,22	0,33269	0	GSPATT00018242001	-2,859	0	-1
GSPATT00001089001	0,066	0,67994	0	GSPATT00018690001	0,047	0,85487	0
GSPATT00001397001	-2,106	0	-1	GSPATT00018851001	0,322	0,05086	0
GSPATT00001586001	-1,181	0,00007	-1	GSPATT00018909001	-1,504	0,00003	-1
GSPATT00001620001	-1,808	0	-1	GSPATT00019420001	-1,225	0,00003	-1
GSPATT00002351001	-1,356	0,00003	-1	GSPATT00019523001	0,625	0,00224	0
GSPATT00002484001	-1,693	0,00031	-1	GSPATT00019708001	-1,406	0,00017	-1
GSPATT00002646001	-1,823	0,00001	-1	GSPATT00020067001	-0,076	0,65721	0
GSPATT00003214001	-2,123	0	-1	GSPATT00022485001	0,712	0,00082	0
GSPATT00003228001	-0,385	0,10828	0	GSPATT00022544001	-0,08	0,63682	0
GSPATT00003296001	0,012	0,93563	0	GSPATT00023693001	0,56	0,02439	0
GSPATT00003518001	0,775	0,00349	0	GSPATT00023879001	-3,261	0	-1
GSPATT00003624001	-2,399	0,00001	-1	GSPATT00024439001	-2,018	0	-1
GSPATT00004031001	0,849	0,00389	0	GSPATT00024550001	-2,099	0	-1
GSPATT00004884001	-2,504	0	-1	GSPATT00024986001	0,689	0,01199	0
GSPATT00005004001	-1,096	0,00025	-1	GSPATT00025299001	-1,278	0,00008	-1
GSPATT00005139001	-1,388	0,00024	-1	GSPATT00025378001	-2,3	0	-1
GSPATT00005241001	-1,405	0,00006	-1	GSPATT00027070001	-1,623	0,00001	-1
GSPATT00005440001	-0,958	0,0009	0	GSPATT00027423001	-1,583	0,00071	-1
GSPATT00005981001	0,392	0,01996	0	GSPATT00027502001	-0,002	0,99357	0
GSPATT00006389001	-0,86	0,00039	0	GSPATT00027936001	-1,124	0,00004	-1
GSPATT00006813001	-0,83	0,00083	0	GSPATT00027961001	-2,425	0	-1
GSPATT00007112001	-2,201	0,00002	-1	GSPATT00028109001	-0,511	0,04822	0
GSPATT00008642001	-0,297	0,13029	0	GSPATT00028569001	-1,323	0,0002	-1
GSPATT00008712001	-0,15	0,38371	0	GSPATT00028919001	0,135	0,40445	0
GSPATT00008903001	0,308	0,20793	0	GSPATT00029116001	-2,06	0	-1
GSPATT00008926001	-1,307	0,00001	-1	GSPATT00030447001	-2,057	0,00001	-1
GSPATT00009177001	-2,028	0,00001	-1	GSPATT00031217001	-1,256	0,00035	-1
GSPATT00009493001	-1,344	0,00006	-1	GSPATT00031325001	0,342	0,04168	0
GSPATT00009702001	-0,861	0,00134	0	GSPATT00031557001	-2,404	0	-1
GSPATT00010014001	-1,429	0,00007	-1	GSPATT00031771001	-0,175	0,21941	0
GSPATT00010278001	-1,033	0,00006	-1	GSPATT00032045001	1,262	0,00004	1
GSPATT00010510001	-1,098	0,00011	-1	GSPATT00033286001	0,194	0,25838	0
GSPATT00010697001	0,469	0,00759	0	GSPATT00033453001	-0,901	0,00015	0
GSPATT00010840001	0,527	0,00478	0	GSPATT00033657001	-3,351	0	-1
GSPATT00010887001	0,854	0,00098	0	GSPATT00034423001	-2,215	0,00005	-1
GSPATT00011178001	-2,174	0	-1	GSPATT00034503001	-2,304	0	-1
GSPATT00011487001	-1,542	0	-1	GSPATT00034541001	-1,012	0,00017	-1
GSPATT00012003001	0,811	0,04299	0	GSPATT00035155001	0,505	0,01426	0
GSPATT00012503001	0,744	0,00319	0	GSPATT00035465001	-2,322	0	-1
GSPATT00013091001	-3,48	0	-1	GSPATT00035500001	1,219	0,00004	1
GSPATT00013641001	-4,033	0	-1	GSPATT00035836001	0,097	0,73724	0
GSPATT00013838001	0,587	0,00239	0	GSPATT00036346001	0,451	0,00869	0
GSPATT00013999001	-1,01	0,00005	-1	GSPATT00036420001	-3,876	0	-1
GSPATT00014029001	-2,023	0	-1	GSPATT00036625001	0,996	0,02958	0
GSPATT00014331001	1,055	0,00005	1	GSPATT00037481001	-1,322	0,00002	-1
GSPATT00014719001	0,318	0,05814	0	GSPATT00038451001	0,914	0,00012	0
GSPATT00015030001	-0,653	0,00079	0	GSPATT00038687001	-1,029	0,00009	-1
GSPATT00015427001	0,645	0,00824	0	GSPATT00038699001	0,815	0,00085	0
GSPATT00016260001	-1,138	0,00014	-1	GSPATT00039254001	-0,783	0,00071	0
GSPATT00016268001	0,758	0,00403	0	GSPATT00039584001	-1,173	0,00007	-1

GO 0051276 chromosome organization

ID	coeff.	p-value	signif.	ID	coeff.	p-value	signif.
GSPATT00000182001	1,943	0,00003	1	GSPATT00016268001	0,758	0,00403	0
GSPATT00000820001	-0,476	0,01317	0	GSPATT00016556001	-1,26	0,00452	-1
GSPATT00000899001	0,556	0,00363	0	GSPATT00016929001	-1,277	0,00088	-1
GSPATT00000918001	0,22	0,33269	0	GSPATT00017994001	-0,085	0,59009	0
GSPATT00001065001	-0,143	0,69307	0	GSPATT00018066001	-3,301	0	-1
GSPATT00001089001	0,066	0,67994	0	GSPATT00018242001	-2,859	0	-1
GSPATT00001397001	-2,106	0	-1	GSPATT00018690001	0,047	0,85487	0
GSPATT00001586001	-1,181	0,00007	-1	GSPATT00018851001	0,322	0,05086	0

GSPATT00001620001	-1,808	0	-1	GSPATT00018909001	-1,504	0,00003	-1
GSPATT00002202001	-0,633	0,00593	0	GSPATT00019169001	-0,494	0,02586	0
GSPATT00002351001	-1,356	0,00003	-1	GSPATT00019420001	-1,225	0,00003	-1
GSPATT00002484001	-1,693	0,00031	-1	GSPATT00019523001	0,625	0,00224	0
GSPATT00002646001	-1,823	0,00001	-1	GSPATT00019708001	-1,406	0,00017	-1
GSPATT00003214001	-2,123	0	-1	GSPATT00020067001	-0,076	0,65721	0
GSPATT00003228001	-0,385	0,10828	0	GSPATT00022197001	0,191	0,33245	0
GSPATT00003296001	0,012	0,93563	0	GSPATT00022485001	0,712	0,00082	0
GSPATT00003518001	0,775	0,00349	0	GSPATT00022544001	-0,08	0,63682	0
GSPATT00003624001	-2,399	0,00001	-1	GSPATT00023095001	-0,105	0,42062	0
GSPATT00004031001	0,849	0,00389	0	GSPATT00023693001	0,56	0,02439	0
GSPATT00004464001	-0,315	0,08048	0	GSPATT00023879001	-3,261	0	-1
GSPATT00004884001	-2,504	0	-1	GSPATT00024439001	-2,018	0	-1
GSPATT00005004001	-1,096	0,00025	-1	GSPATT00024550001	-2,099	0	-1
GSPATT00005139001	-1,388	0,00024	-1	GSPATT00024986001	0,689	0,01199	0
GSPATT00005241001	-1,405	0,00006	-1	GSPATT00025299001	-1,278	0,00008	-1
GSPATT00005440001	-0,958	0,0009	0	GSPATT00025378001	-2,3	0	-1
GSPATT00005981001	0,392	0,01996	0	GSPATT00025447001	0,033	0,85164	0
GSPATT00006061001	-2,543	0	-1	GSPATT00027070001	-1,623	0,00001	-1
GSPATT00006389001	-0,86	0,00039	0	GSPATT00027423001	-1,583	0,00071	-1
GSPATT00006666001	-0,748	0,0059	0	GSPATT00027502001	-0,002	0,99357	0
GSPATT00006813001	-0,83	0,00083	0	GSPATT00027936001	-1,124	0,00004	-1
GSPATT00007112001	-2,201	0,00002	-1	GSPATT00027961001	-2,425	0	-1
GSPATT00008642001	-0,297	0,13029	0	GSPATT00028109001	-0,511	0,04822	0
GSPATT00008712001	-0,15	0,38371	0	GSPATT00028569001	-1,323	0,0002	-1
GSPATT00008903001	0,308	0,20793	0	GSPATT00028847001	-1,597	0	-1
GSPATT00008926001	-1,307	0,00001	-1	GSPATT00028919001	0,135	0,40445	0
GSPATT00009177001	-2,028	0,00001	-1	GSPATT00029116001	-2,06	0	-1
GSPATT00009403001	-0,631	0,05954	0	GSPATT00030447001	-2,057	0,00001	-1
GSPATT00009493001	-1,344	0,00006	-1	GSPATT00031217001	-1,256	0,00035	-1
GSPATT00009702001	-0,861	0,00134	0	GSPATT00031325001	0,342	0,04168	0
GSPATT00010014001	-1,429	0,00007	-1	GSPATT00031557001	-2,404	0	-1
GSPATT00010278001	-1,033	0,00006	-1	GSPATT00031622001	-0,037	0,78942	0
GSPATT00010510001	-1,098	0,00011	-1	GSPATT00031771001	-0,175	0,21941	0
GSPATT00010697001	0,469	0,00759	0	GSPATT00032045001	1,262	0,00004	1
GSPATT00010840001	0,527	0,00478	0	GSPATT00033286001	0,194	0,25838	0
GSPATT00010887001	0,854	0,00098	0	GSPATT00033453001	-0,901	0,00015	0
GSPATT00010948001	-0,339	0,04081	0	GSPATT00033657001	-3,351	0	-1
GSPATT00011111001	0,784	0,00245	0	GSPATT00034423001	-2,215	0,00005	-1
GSPATT00011178001	-2,174	0	-1	GSPATT00034453001	1,089	0,00014	1
GSPATT00011487001	-1,542	0	-1	GSPATT00034503001	-2,304	0	-1
GSPATT00011565001	0,357	0,06272	0	GSPATT00034541001	-1,012	0,00017	-1
GSPATT00012003001	0,811	0,04299	0	GSPATT00035155001	0,505	0,01426	0
GSPATT00012503001	0,744	0,00319	0	GSPATT00035465001	-2,322	0	-1
GSPATT00013091001	-3,48	0	-1	GSPATT00035500001	1,219	0,00004	1
GSPATT00013641001	-4,033	0	-1	GSPATT00035836001	0,097	0,73724	0
GSPATT00013838001	0,587	0,00239	0	GSPATT00036346001	0,451	0,00869	0
GSPATT00013999001	-1,01	0,00005	-1	GSPATT00036420001	-3,876	0	-1
GSPATT00014029001	-2,023	0	-1	GSPATT00036625001	0,996	0,02958	0
GSPATT00014331001	1,055	0,00005	1	GSPATT00037481001	-1,322	0,00002	-1
GSPATT00014430001	0,022	0,88182	0	GSPATT00038451001	0,914	0,00012	0
GSPATT00014657001	0,857	0,00015	0	GSPATT00038687001	-1,029	0,00009	-1
GSPATT00014719001	0,318	0,05814	0	GSPATT00038699001	0,815	0,00085	0
GSPATT00015030001	-0,653	0,00079	0	GSPATT00039254001	-0,783	0,00071	0
GSPATT00015427001	0,645	0,00824	0	GSPATT00039584001	-1,173	0,00007	-1
GSPATT00016260001	-1,138	0,00014	-1				

GO 0006325 chromatin organization

ID	coeff.	p-value	signif.	ID	coeff.	p-value	signif.
GSPATT00000182001	1,943	0,00003	1	GSPATT00016268001	0,758	0,00403	0
GSPATT00000820001	-0,476	0,01317	0	GSPATT00016556001	-1,26	0,00452	-1
GSPATT00000899001	0,556	0,00363	0	GSPATT00016929001	-1,277	0,00088	-1
GSPATT00000918001	0,22	0,33269	0	GSPATT00018066001	-3,301	0	-1
GSPATT00001089001	0,066	0,67994	0	GSPATT00018242001	-2,859	0	-1
GSPATT00001397001	-2,106	0	-1	GSPATT00018690001	0,047	0,85487	0
GSPATT00001586001	-1,181	0,00007	-1	GSPATT00018851001	0,322	0,05086	0
GSPATT00001620001	-1,808	0	-1	GSPATT00018909001	-1,504	0,00003	-1

GSPATT00002202001	-0,633	0,00593	0	GSPATT00019169001	-0,494	0,02586	0
GSPATT00002351001	-1,356	0,00003	-1	GSPATT00019420001	-1,225	0,00003	-1
GSPATT00002484001	-1,693	0,00031	-1	GSPATT00019523001	0,625	0,00224	0
GSPATT00002646001	-1,823	0,00001	-1	GSPATT00019708001	-1,406	0,00017	-1
GSPATT00003214001	-2,123	0	-1	GSPATT00020067001	-0,076	0,65721	0
GSPATT00003228001	-0,385	0,10828	0	GSPATT00022197001	0,191	0,33245	0
GSPATT00003296001	0,012	0,93563	0	GSPATT00022485001	0,712	0,00082	0
GSPATT00003518001	0,775	0,00349	0	GSPATT00022544001	-0,08	0,63682	0
GSPATT00003624001	-2,399	0,00001	-1	GSPATT00023095001	-0,105	0,42062	0
GSPATT00004031001	0,849	0,00389	0	GSPATT00023693001	0,56	0,02439	0
GSPATT00004464001	-0,315	0,08048	0	GSPATT00023879001	-3,261	0	-1
GSPATT00004884001	-2,504	0	-1	GSPATT00024439001	-2,018	0	-1
GSPATT00005004001	-1,096	0,00025	-1	GSPATT00024550001	-2,099	0	-1
GSPATT00005139001	-1,388	0,00024	-1	GSPATT00024986001	0,689	0,01199	0
GSPATT00005241001	-1,405	0,00006	-1	GSPATT00025299001	-1,278	0,00008	-1
GSPATT00005440001	-0,958	0,0009	0	GSPATT00025378001	-2,3	0	-1
GSPATT00005981001	0,392	0,01996	0	GSPATT00025447001	0,033	0,85164	0
GSPATT00006061001	-2,543	0	-1	GSPATT00027070001	-1,623	0,00001	-1
GSPATT00006389001	-0,86	0,00039	0	GSPATT00027423001	-1,583	0,00071	-1
GSPATT00006666001	-0,748	0,0059	0	GSPATT00027502001	-0,002	0,99357	0
GSPATT00006813001	-0,83	0,00083	0	GSPATT00027936001	-1,124	0,00004	-1
GSPATT00007112001	-2,201	0,00002	-1	GSPATT00027961001	-2,425	0	-1
GSPATT00008642001	-0,297	0,13029	0	GSPATT00028109001	-0,511	0,04822	0
GSPATT00008712001	-0,15	0,38371	0	GSPATT00028569001	-1,323	0,0002	-1
GSPATT00008903001	0,308	0,20793	0	GSPATT00028919001	0,135	0,40445	0
GSPATT00008926001	-1,307	0,00001	-1	GSPATT00029116001	-2,06	0	-1
GSPATT00009177001	-2,028	0,00001	-1	GSPATT00030447001	-2,057	0,00001	-1
GSPATT00009493001	-1,344	0,00006	-1	GSPATT00031217001	-1,256	0,00035	-1
GSPATT00009702001	-0,861	0,00134	0	GSPATT00031325001	0,342	0,04168	0
GSPATT00010014001	-1,429	0,00007	-1	GSPATT00031557001	-2,404	0	-1
GSPATT00010278001	-1,033	0,00006	-1	GSPATT00031622001	-0,037	0,78942	0
GSPATT00010510001	-1,098	0,00011	-1	GSPATT00031771001	-0,175	0,21941	0
GSPATT00010697001	0,469	0,00759	0	GSPATT00032045001	1,262	0,00004	1
GSPATT00010840001	0,527	0,00478	0	GSPATT00033286001	0,194	0,25838	0
GSPATT00010887001	0,854	0,00098	0	GSPATT00033453001	-0,901	0,00015	0
GSPATT00010948001	-0,339	0,04081	0	GSPATT00033657001	-3,351	0	-1
GSPATT00011111001	0,784	0,00245	0	GSPATT00034423001	-2,215	0,00005	-1
GSPATT00011178001	-2,174	0	-1	GSPATT00034453001	1,089	0,00014	1
GSPATT00011487001	-1,542	0	-1	GSPATT00034503001	-2,304	0	-1
GSPATT00011565001	0,357	0,06272	0	GSPATT00034541001	-1,012	0,00017	-1
GSPATT00012003001	0,811	0,04299	0	GSPATT00035155001	0,505	0,01426	0
GSPATT00012503001	0,744	0,00319	0	GSPATT00035465001	-2,322	0	-1
GSPATT00013091001	-3,48	0	-1	GSPATT00035500001	1,219	0,00004	1
GSPATT00013641001	-4,033	0	-1	GSPATT00035836001	0,097	0,73724	0
GSPATT00013838001	0,587	0,00239	0	GSPATT00036346001	0,451	0,00869	0
GSPATT00013999001	-1,01	0,00005	-1	GSPATT00036420001	-3,876	0	-1
GSPATT00014029001	-2,023	0	-1	GSPATT00036625001	0,996	0,02958	0
GSPATT00014331001	1,055	0,00005	1	GSPATT00037481001	-1,322	0,00002	-1
GSPATT00014430001	0,022	0,88182	0	GSPATT00038451001	0,914	0,00012	0
GSPATT00014657001	0,857	0,00015	0	GSPATT00038687001	-1,029	0,00009	-1
GSPATT00014719001	0,318	0,05814	0	GSPATT00038699001	0,815	0,00085	0
GSPATT00015030001	-0,653	0,00079	0	GSPATT00039254001	-0,783	0,00071	0
GSPATT00015427001	0,645	0,00824	0	GSPATT00039584001	-1,173	0,00007	-1
GSPATT00016260001	-1,138	0,00014	-1				

GO 000-6323/-6334/-31497/-34728/-65004/-71824 DNA packaging/nucleosome GSPATTchromatin) assembly nucleosome organization/prot.-DNA complex assembly-/subunit organization

ID	coeff.	p-value	signif.	ID	coeff.	p-value	signif.
GSPATT00001397001	-2,106	0	-1	GSPATT00018690001	0,047	0,85487	0
GSPATT00001620001	-1,808	0	-1	GSPATT00018909001	-1,504	0,00003	-1
GSPATT00002351001	-1,356	0,00003	-1	GSPATT00019420001	-1,225	0,00003	-1
GSPATT00002646001	-1,823	0,00001	-1	GSPATT00019523001	0,625	0,00224	0
GSPATT00003214001	-2,123	0	-1	GSPATT00022485001	0,712	0,00082	0
GSPATT00004884001	-2,504	0	-1	GSPATT00024439001	-2,018	0	-1
GSPATT00005004001	-1,096	0,00025	-1	GSPATT00024550001	-2,099	0	-1
GSPATT00005440001	-0,958	0,0009	0	GSPATT00024986001	0,689	0,01199	0
GSPATT00006389001	-0,86	0,00039	0	GSPATT00025299001	-1,278	0,00008	-1

GSPATT00006813001	-0,83	0,00083	0	GSPATT00025378001	-2,3	0	-1
GSPATT00007112001	-2,201	0,00002	-1	GSPATT00027423001	-1,583	0,00071	-1
GSPATT00008926001	-1,307	0,00001	-1	GSPATT00027502001	-0,002	0,99357	0
GSPATT00009702001	-0,861	0,00134	0	GSPATT00027936001	-1,124	0,00004	-1
GSPATT00010278001	-1,033	0,00006	-1	GSPATT00027961001	-2,425	0	-1
GSPATT00010697001	0,469	0,00759	0	GSPATT00028569001	-1,323	0,0002	-1
GSPATT00010840001	0,527	0,00478	0	GSPATT00029116001	-2,06	0	-1
GSPATT00011178001	-2,174	0	-1	GSPATT00030447001	-2,057	0,00001	-1
GSPATT00011487001	-1,542	0	-1	GSPATT00031217001	-1,256	0,00035	-1
GSPATT00012003001	0,811	0,04299	0	GSPATT00031557001	-2,404	0	-1
GSPATT00012503001	0,744	0,00319	0	GSPATT00033453001	-0,901	0,00015	0
GSPATT00013838001	0,587	0,00239	0	GSPATT00034423001	-2,215	0,00005	-1
GSPATT00013999001	-1,01	0,00005	-1	GSPATT00034503001	-2,304	0	-1
GSPATT00014029001	-2,023	0	-1	GSPATT00035465001	-2,322	0	-1
GSPATT00014331001	1,055	0,00005	1	GSPATT00036346001	0,451	0,00869	0
GSPATT00015427001	0,645	0,00824	0	GSPATT00036420001	-3,876	0	-1
GSPATT00016268001	0,758	0,00403	0	GSPATT00037481001	-1,322	0,00002	-1
GSPATT00016556001	-1,26	0,00452	-1	GSPATT00038451001	0,914	0,00012	0
GSPATT00018066001	-3,301	0	-1	GSPATT00038699001	0,815	0,00085	0
GSPATT00018242001	-2,859	0	-1	GSPATT00039584001	-1,173	0,00007	-1

GO 0006996 organelle organization

ID	coeff.	p-value	signif.	ID	coeff.	p-value	signif.
GSPATT00000154001	-0,063	0,635	0	GSPATT00016929001	-1,277	0,00088	-1
GSPATT00000182001	1,943	0,00003	1	GSPATT00017254001	-0,111	0,58323	0
GSPATT00000820001	-0,476	0,01317	0	GSPATT00017994001	-0,085	0,59009	0
GSPATT00000899001	0,556	0,00363	0	GSPATT00018066001	-3,301	0	-1
GSPATT00000918001	0,22	0,33269	0	GSPATT00018242001	-2,859	0	-1
GSPATT00001065001	-0,143	0,69307	0	GSPATT00018690001	0,047	0,85487	0
GSPATT00001089001	0,066	0,67994	0	GSPATT00018851001	0,322	0,05086	0
GSPATT00001397001	-2,106	0	-1	GSPATT00018909001	-1,504	0,00003	-1
GSPATT00001586001	-1,181	0,00007	-1	GSPATT00019048001	0,13	0,50226	0
GSPATT00001620001	-1,808	0	-1	GSPATT00019169001	-0,494	0,02586	0
GSPATT00002148001	0,727	0,02739	0	GSPATT00019420001	-1,225	0,00003	-1
GSPATT00002202001	-0,633	0,00593	0	GSPATT00019462001	-0,055	0,69698	0
GSPATT00002222001	-0,531	0,0041	0	GSPATT00019523001	0,625	0,00224	0
GSPATT00002351001	-1,356	0,00003	-1	GSPATT00019626001	0,571	0,00725	0
GSPATT00002427001	-0,034	0,80008	0	GSPATT00019708001	-1,406	0,00017	-1
GSPATT00002484001	-1,693	0,00031	-1	GSPATT00020067001	-0,076	0,65721	0
GSPATT00002646001	-1,823	0,00001	-1	GSPATT00022197001	0,191	0,33245	0
GSPATT00003214001	-2,123	0	-1	GSPATT00022485001	0,712	0,00082	0
GSPATT00003228001	-0,385	0,10828	0	GSPATT00022492001	-1,45	0,00012	-1
GSPATT00003296001	0,012	0,93563	0	GSPATT00022544001	-0,08	0,63682	0
GSPATT00003496001	-0,008	0,95309	0	GSPATT00023012001	-0,444	0,01618	0
GSPATT00003518001	0,775	0,00349	0	GSPATT00023095001	-0,105	0,42062	0
GSPATT00003624001	-2,399	0,00001	-1	GSPATT00023229001	-0,503	0,01096	0
GSPATT00004031001	0,849	0,00389	0	GSPATT00023693001	0,56	0,02439	0
GSPATT00004143001	-1,018	0,00011	-1	GSPATT00023809001	0,404	0,0123	0
GSPATT00004357001	0,123	0,4116	0	GSPATT00023879001	-3,261	0	-1
GSPATT00004464001	-0,315	0,08048	0	GSPATT00023912001	-0,072	0,62903	0
GSPATT00004623001	-1,064	0,00004	-1	GSPATT00024085001	-0,567	0,00415	0
GSPATT00004884001	-2,504	0	-1	GSPATT00024163001	-1,371	0,00078	-1
GSPATT00004942001	0,31	0,04371	0	GSPATT00024439001	-2,018	0	-1
GSPATT00005004001	-1,096	0,00025	-1	GSPATT00024550001	-2,099	0	-1
GSPATT00005139001	-1,388	0,00024	-1	GSPATT00024849001	0,6	0,0059	0
GSPATT00005241001	-1,405	0,00006	-1	GSPATT00024986001	0,689	0,01199	0
GSPATT00005440001	-0,958	0,0009	0	GSPATT00025299001	-1,278	0,00008	-1
GSPATT00005448001	0,47	0,00735	0	GSPATT00025378001	-2,3	0	-1
GSPATT00005484001	-1,295	0,00019	-1	GSPATT00025447001	0,033	0,85164	0
GSPATT00005981001	0,392	0,01996	0	GSPATT00025727001	0,078	0,67982	0
GSPATT00006061001	-2,543	0	-1	GSPATT00026494001	-0,143	0,48656	0
GSPATT00006246001	0,364	0,09095	0	GSPATT00027070001	-1,623	0,00001	-1
GSPATT00006338001	0,711	0,00168	0	GSPATT00027423001	-1,583	0,00071	-1
GSPATT00006389001	-0,86	0,00039	0	GSPATT00027502001	-0,002	0,99357	0
GSPATT00006606001	-0,504	0,07047	0	GSPATT00027936001	-1,124	0,00004	-1
GSPATT00006666001	-0,748	0,0059	0	GSPATT00027961001	-2,425	0	-1
GSPATT00006682001	-0,333	0,06949	0	GSPATT00028109001	-0,511	0,04822	0

GSPATT00006813001	-0,83	0,00083	0	GSPATT00028569001	-1,323	0,0002	-1
GSPATT00006878001	-1,167	0,00004	-1	GSPATT00028711001	-0,452	0,07773	0
GSPATT00007112001	-2,201	0,00002	-1	GSPATT00028779001	0,119	0,43952	0
GSPATT00008039001	0,144	0,30322	0	GSPATT00028847001	-1,597	0	-1
GSPATT00008212001	-0,412	0,03176	0	GSPATT00028869001	-1,156	0,00132	-1
GSPATT00008642001	-0,297	0,13029	0	GSPATT00028919001	0,135	0,40445	0
GSPATT00008712001	-0,15	0,38371	0	GSPATT00029116001	-2,06	0	-1
GSPATT00008903001	0,308	0,20793	0	GSPATT00029199001	0,731	0,00445	0
GSPATT00008926001	-1,307	0,00001	-1	GSPATT00029939001	-0,425	0,05707	0
GSPATT00009177001	-2,028	0,00001	-1	GSPATT00030153001	0,054	0,77133	0
GSPATT00009403001	-0,631	0,05954	0	GSPATT00030162001	-0,596	0,00185	0
GSPATT00009493001	-1,344	0,00006	-1	GSPATT00030447001	-2,057	0,00001	-1
GSPATT00009576001	0,413	0,04928	0	GSPATT00030983001	-0,565	0,00782	0
GSPATT00009702001	-0,861	0,00134	0	GSPATT00031217001	-1,256	0,00035	-1
GSPATT00010014001	-1,429	0,00007	-1	GSPATT00031325001	0,342	0,04168	0
GSPATT00010221001	-0,865	0,00345	0	GSPATT00031557001	-2,404	0	-1
GSPATT00010278001	-1,033	0,00006	-1	GSPATT00031622001	-0,037	0,78942	0
GSPATT00010510001	-1,098	0,00011	-1	GSPATT00031771001	-0,175	0,21941	0
GSPATT00010592001	0,944	0,00628	0	GSPATT00032045001	1,262	0,00004	1
GSPATT00010697001	0,469	0,00759	0	GSPATT00033094001	-0,377	0,096	0
GSPATT00010840001	0,527	0,00478	0	GSPATT00033286001	0,194	0,25838	0
GSPATT00010887001	0,854	0,00098	0	GSPATT00033453001	-0,901	0,00015	0
GSPATT00010948001	-0,339	0,04081	0	GSPATT00033489001	0,384	0,03372	0
GSPATT00011111001	0,784	0,00245	0	GSPATT00033588001	0,445	0,00756	0
GSPATT00011178001	-2,174	0	-1	GSPATT00033657001	-3,351	0	-1
GSPATT00011487001	-1,542	0	-1	GSPATT00033688001	0,056	0,75045	0
GSPATT00011541001	0,642	0,00388	0	GSPATT00034423001	-2,215	0,00005	-1
GSPATT00011565001	0,357	0,06272	0	GSPATT00034453001	1,089	0,00014	1
GSPATT00011883001	-1,095	0,00012	-1	GSPATT00034503001	-2,304	0	-1
GSPATT00011984001	-0,077	0,67506	0	GSPATT00034541001	-1,012	0,00017	-1
GSPATT00012003001	0,811	0,04299	0	GSPATT00034610001	1,415	0,00004	1
GSPATT00012023001	-0,74	0,00153	0	GSPATT00035155001	0,505	0,01426	0
GSPATT00012503001	0,744	0,00319	0	GSPATT00035465001	-2,322	0	-1
GSPATT00012623001	-0,113	0,42282	0	GSPATT00035500001	1,219	0,00004	1
GSPATT00013091001	-3,48	0	-1	GSPATT00035836001	0,097	0,73724	0
GSPATT00013641001	-4,033	0	-1	GSPATT00035871001	-0,36	0,02716	0
GSPATT00013838001	0,587	0,00239	0	GSPATT00035960001	0,28	0,09294	0
GSPATT00013999001	-1,01	0,00005	-1	GSPATT00035964001	-0,863	0,00067	0
GSPATT00014029001	-2,023	0	-1	GSPATT00036081001	-0,394	0,01835	0
GSPATT00014331001	1,055	0,00005	1	GSPATT00036346001	0,451	0,00869	0
GSPATT00014430001	0,022	0,88182	0	GSPATT00036356001	-0,795	0,00063	0
GSPATT00014657001	0,857	0,00015	0	GSPATT00036420001	-3,876	0	-1
GSPATT00014719001	0,318	0,05814	0	GSPATT00036625001	0,996	0,02958	0
GSPATT00014848001	0,877	0,00028	0	GSPATT00037481001	-1,322	0,00002	-1
GSPATT00014878001	-0,206	0,35695	0	GSPATT00038451001	0,914	0,00012	0
GSPATT00015030001	-0,653	0,00079	0	GSPATT00038687001	-1,029	0,00009	-1
GSPATT00015427001	0,645	0,00824	0	GSPATT00038699001	0,815	0,00085	0
GSPATT00016260001	-1,138	0,00014	-1	GSPATT00039254001	-0,783	0,00071	0
GSPATT00016268001	0,758	0,00403	0	GSPATT00039584001	-1,173	0,00007	-1
GSPATT00016556001	-1,26	0,00452	-1				

GO 0016310 phosphorylation

ID	coeff.	p-value	signif.	ID	coeff.	p-value	signif.
GSPATT00000029001	-0,656	0,02257	0	GSPATT00019627001	0,267	0,13823	0
GSPATT00000047001	0,686	0,01107	0	GSPATT00019648001	0,266	0,10254	0
GSPATT00000064001	-0,605	0,00365	0	GSPATT00019665001	0,352	0,16137	0
GSPATT00000093001	0,319	0,15526	0	GSPATT00019672001	0,262	0,38511	0
GSPATT00000160001	-0,436	0,09808	0	GSPATT00019684001	0,599	0,03897	0
GSPATT00000190001	0,762	0,00321	0	GSPATT00019701001	0,633	0,00548	0
GSPATT00000282001	-0,179	0,30932	0	GSPATT00019734001	0,538	0,01044	0
GSPATT00000295001	0,583	0,02578	0	GSPATT00019758001	-0,857	0,00047	0
GSPATT00000324001	-0,092	0,63602	0	GSPATT00019770001	0,159	0,3745	0
GSPATT00000365001	0,81	0,00102	0	GSPATT00019834001	0,855	0,00735	0
GSPATT00000366001	0,639	0,01895	0	GSPATT00019835001	0,65	0,03101	0
GSPATT00000384001	0,653	0,02308	0	GSPATT00019840001	0,546	0,02765	0
GSPATT00000394001	-0,275	0,16617	0	GSPATT00019861001	0,155	0,42691	0
GSPATT00000415001	-0,571	0,00652	0	GSPATT00019876001	-0,036	0,9177	0

GSPATT00000434001	0,504	0,02973	0	GSPATT00019885001	1,059	0,00032	1
GSPATT00000482001	-0,044	0,82968	0	GSPATT00019888001	1,012	0,00029	1
GSPATT00000508001	-0,009	0,95625	0	GSPATT00019898001	0,291	0,35823	0
GSPATT00000542001	0,969	0,00094	0	GSPATT00019899001	1,302	0,01819	1
GSPATT00000559001	0,401	0,04709	0	GSPATT00019918001	1,36	0,00661	1
GSPATT00000560001	0,804	0,0092	0	GSPATT00019930001	-0,02	0,89979	0
GSPATT00000594001	-1,714	0,00001	-1	GSPATT00019935001	0,306	0,30175	0
GSPATT00000596001	-1,334	0,00022	-1	GSPATT00019962001	0,101	0,63424	0
GSPATT00000599001	-0,212	0,13935	0	GSPATT00019970001	0,305	0,11022	0
GSPATT00000615001	0,04	0,80664	0	GSPATT00019974001	0,112	0,7056	0
GSPATT00000619001	0,52	0,00518	0	GSPATT00019988001	-0,077	0,59245	0
GSPATT00000630001	0,066	0,80692	0	GSPATT00020006001	0,694	0,0227	0
GSPATT00000664001	0,473	0,02458	0	GSPATT00020016001	-0,284	0,16066	0
GSPATT00000685001	0,283	0,12345	0	GSPATT00020018001	0,567	0,00877	0
GSPATT00000704001	1,505	0,00001	1	GSPATT00020040001	0,426	0,13971	0
GSPATT00000760001	1,018	0,00234	1	GSPATT00020047001	0,719	0,02416	0
GSPATT00000783001	0,956	0,007	0	GSPATT00020051001	0,424	0,02871	0
GSPATT00000795001	0,73	0,00542	0	GSPATT00020056001	-0,263	0,09627	0
GSPATT00000817001	-0,289	0,11652	0	GSPATT00020061001	0,203	0,18858	0
GSPATT00000822001	0,006	0,96587	0	GSPATT00020081001	0,562	0,01428	0
GSPATT00000824001	1,895	0,00001	1	GSPATT00020083001	-0,007	0,96307	0
GSPATT00000926001	-0,44	0,07676	0	GSPATT00020106001	0,617	0,04707	0
GSPATT00000934001	0,7	0,00134	0	GSPATT00020133001	0,915	0,00052	0
GSPATT00000950001	-0,039	0,85119	0	GSPATT00020136001	0,721	0,00761	0
GSPATT00000961001	1,244	0,00014	1	GSPATT00020148001	0,628	0,00191	0
GSPATT00000985001	-0,053	0,78626	0	GSPATT00020151001	0,152	0,25786	0
GSPATT00001013001	0,18	0,3735	0	GSPATT00020164001	0,049	0,79466	0
GSPATT00001029001	0,351	0,04038	0	GSPATT00020171001	0,019	0,89222	0
GSPATT00001071001	-1,676	0,00003	-1	GSPATT00020175001	-0,408	0,07148	0
GSPATT00001077001	-1,834	0	-1	GSPATT00020206001	1,187	0,00008	1
GSPATT00001082001	0,802	0,00363	0	GSPATT00020222001	0,022	0,8861	0
GSPATT00001085001	-0,102	0,55835	0	GSPATT00020225001	0,491	0,02758	0
GSPATT00001088001	-1,002	0,00029	-1	GSPATT00020239001	-0,066	0,6879	0
GSPATT00001107001	1,346	0,00005	1	GSPATT00020280001	0,417	0,00934	0
GSPATT00001118001	0,362	0,12047	0	GSPATT00020292001	0,983	0,00426	0
GSPATT00001133001	-0,118	0,39808	0	GSPATT00020315001	0,421	0,02041	0
GSPATT00001155001	-0,688	0,04688	0	GSPATT00020317001	1,39	0,00016	1
GSPATT00001162001	-0,208	0,17385	0	GSPATT00020328001	0,132	0,45889	0
GSPATT00001183001	-1,126	0,00625	-1	GSPATT00020353001	0,488	0,03007	0
GSPATT00001189001	-0,09	0,61561	0	GSPATT00020354001	0,971	0,00377	0
GSPATT00001207001	0,62	0,00301	0	GSPATT00020357001	0,423	0,11371	0
GSPATT00001216001	-0,859	0,0049	0	GSPATT00020375001	0,159	0,26389	0
GSPATT00001230001	0,185	0,20391	0	GSPATT00020379001	0,528	0,00684	0
GSPATT00001255001	-0,19	0,27269	0	GSPATT00020390001	-1,113	0,00014	-1
GSPATT00001260001	1,21	0,00035	1	GSPATT00020397001	0,42	0,02881	0
GSPATT00001268001	-0,542	0,00397	0	GSPATT00020442001	0,565	0,009	0
GSPATT00001273001	0,742	0,00126	0	GSPATT00020450001	0,494	0,01321	0
GSPATT00001279001	0,193	0,33618	0	GSPATT00020501001	0,141	0,29138	0
GSPATT00001292001	-0,196	0,32171	0	GSPATT00020577001	0,213	0,12758	0
GSPATT00001310001	0,604	0,00221	0	GSPATT00020591001	0,653	0,02277	0
GSPATT00001314001	0,32	0,07355	0	GSPATT00020594001	0,125	0,38102	0
GSPATT00001320001	0,724	0,00683	0	GSPATT00020603001	0,424	0,03461	0
GSPATT00001342001	0,712	0,00317	0	GSPATT00020607001	0,553	0,03187	0
GSPATT00001359001	0,036	0,83426	0	GSPATT00020634001	-0,891	0,00039	0
GSPATT00001361001	0,243	0,21121	0	GSPATT00020656001	0,384	0,09254	0
GSPATT00001369001	0,226	0,16319	0	GSPATT00020671001	0,4	0,06339	0
GSPATT00001370001	0,219	0,1963	0	GSPATT00020675001	0,023	0,86196	0
GSPATT00001413001	-0,893	0,00057	0	GSPATT00020696001	1,227	0,00012	1
GSPATT00001414001	0,294	0,08325	0	GSPATT00020706001	-2,177	0,00005	-1
GSPATT00001427001	0,007	0,96536	0	GSPATT00020718001	0,498	0,0293	0
GSPATT00001430001	0,427	0,01633	0	GSPATT00020723001	1,447	0,00002	1
GSPATT00001441001	-0,411	0,0844	0	GSPATT00020732001	0,346	0,02628	0
GSPATT00001463001	1,174	0,00247	1	GSPATT00020736001	-0,014	0,93682	0
GSPATT00001467001	-0,474	0,02385	0	GSPATT00020738001	-0,47	0,01442	0
GSPATT00001471001	0,442	0,16312	0	GSPATT00020743001	0,121	0,41116	0
GSPATT00001479001	0,193	0,3499	0	GSPATT00020748001	0,638	0,0469	0
GSPATT00001494001	-0,073	0,59749	0	GSPATT00020754001	0,328	0,15362	0

GSPATT00001504001	0,05	0,75727	0
GSPATT00001508001	0,42	0,01888	0
GSPATT00001527001	0,128	0,47689	0
GSPATT00001554001	0,882	0,00038	0
GSPATT00001563001	-0,431	0,02903	0
GSPATT00001592001	1,698	0,00001	1
GSPATT00001610001	0,834	0,00147	0
GSPATT00001650001	0,32	0,08832	0
GSPATT00001662001	0,438	0,17856	0
GSPATT00001682001	-2,067	0,00007	-1
GSPATT00001698001	-0,612	0,00264	0
GSPATT00001741001	0,068	0,70586	0
GSPATT00001746001	1,163	0,00005	1
GSPATT00001753001	0,102	0,57872	0
GSPATT00001779001	0,661	0,00281	0
GSPATT00001789001	0,667	0,00963	0
GSPATT00001794001	-0,276	0,08218	0
GSPATT00001840001	0,602	0,00734	0
GSPATT00001852001	1,949	0	1
GSPATT00001853001	1,087	0,00026	1
GSPATT00001854001	1,3	0,00007	1
GSPATT00001859001	0,31	0,07571	0
GSPATT00001864001	0,864	0,00071	0
GSPATT00001865001	0,55	0,00393	0
GSPATT00001866001	0,294	0,40339	0
GSPATT00001870001	0,431	0,01514	0
GSPATT00001873001	2,117	0,00001	1
GSPATT00001911001	-0,107	0,62983	0
GSPATT00002000001	0,994	0,00542	0
GSPATT00002011001	0,938	0,00081	0
GSPATT00002013001	0,586	0,02656	0
GSPATT00002014001	0,546	0,01206	0
GSPATT00002052001	0,333	0,07203	0
GSPATT00002063001	0,381	0,02118	0
GSPATT00002065001	0,716	0,00677	0
GSPATT00002068001	1,376	0,00007	1
GSPATT00002077001	-0,063	0,72874	0
GSPATT00002091001	0,425	0,05442	0
GSPATT00002093001	1,052	0,00126	1
GSPATT00002094001	-0,116	0,53723	0
GSPATT00002096001	-0,076	0,75421	0
GSPATT00002107001	-0,282	0,23525	0
GSPATT00002108001	0,114	0,44232	0
GSPATT00002114001	0,469	0,04573	0
GSPATT00002115001	0,601	0,02057	0
GSPATT00002129001	1,065	0,00431	1
GSPATT00002132001	0,587	0,01286	0
GSPATT00002156001	0,933	0,0007	0
GSPATT00002182001	1,186	0,00008	1
GSPATT00002186001	0,08	0,52966	0
GSPATT00002191001	1,024	0,00219	1
GSPATT00002195001	-0,541	0,01959	0
GSPATT00002221001	0,238	0,09743	0
GSPATT00002241001	-0,266	0,23687	0
GSPATT00002247001	0,656	0,00298	0
GSPATT00002269001	-2,667	0,00001	-1
GSPATT00002272001	0,482	0,00978	0
GSPATT00002284001	0,447	0,0176	0
GSPATT00002285001	0,846	0,02305	0
GSPATT00002297001	0,322	0,10354	0
GSPATT00002321001	0,196	0,2005	0
GSPATT00002371001	1,279	0,00502	1
GSPATT00002396001	0,89	0,00446	0
GSPATT00002397001	0,65	0,00105	0
GSPATT00002398001	0,608	0,00382	0
GSPATT00002410001	0,268	0,0869	0
GSPATT00002424001	-0,521	0,01826	0
GSPATT00020755001	1,124	0,00008	1
GSPATT00020757001	0,109	0,4162	0
GSPATT00020787001	0,074	0,69474	0
GSPATT00020798001	-0,359	0,06684	0
GSPATT00020807001	0,453	0,03103	0
GSPATT00020812001	0,207	0,32984	0
GSPATT00020816001	-0,014	0,954	0
GSPATT00020817001	0,81	0,00504	0
GSPATT00020820001	0,296	0,15516	0
GSPATT00020837001	-2,103	0	-1
GSPATT00020863001	1,067	0,00014	1
GSPATT00020867001	-0,219	0,23135	0
GSPATT00020886001	0,561	0,00314	0
GSPATT00020887001	0,231	0,17424	0
GSPATT00020897001	0,202	0,34688	0
GSPATT00020921001	0,201	0,27627	0
GSPATT00020942001	-0,24	0,19484	0
GSPATT00020968001	1,383	0,00001	1
GSPATT00021004001	-0,039	0,80561	0
GSPATT00021006001	-0,917	0,00215	0
GSPATT00021015001	-0,087	0,60216	0
GSPATT00021053001	0,674	0,02023	0
GSPATT00021056001	-0,152	0,53854	0
GSPATT00021078001	0,339	0,1892	0
GSPATT00021080001	-0,038	0,88109	0
GSPATT00021083001	1,214	0,00004	1
GSPATT00021104001	1,014	0,00009	1
GSPATT00021108001	0,03	0,85909	0
GSPATT00021127001	0,409	0,03217	0
GSPATT00021152001	0,566	0,00438	0
GSPATT00021175001	0,819	0,00079	0
GSPATT00021195001	1,22	0,0001	1
GSPATT00021201001	0,446	0,02497	0
GSPATT00021207001	0,704	0,01544	0
GSPATT00021213001	-0,327	0,35092	0
GSPATT00021222001	-1,069	0,00073	-1
GSPATT00021227001	0,331	0,04285	0
GSPATT00021248001	-0,222	0,12494	0
GSPATT00021249001	-0,183	0,27756	0
GSPATT00021250001	0,181	0,35664	0
GSPATT00021252001	2,647	0	1
GSPATT00021256001	0,688	0,00291	0
GSPATT00021258001	1,026	0,00012	1
GSPATT00021270001	0,517	0,01282	0
GSPATT00021271001	0,429	0,01704	0
GSPATT00021273001	1,843	0	1
GSPATT00021274001	1,19	0,00002	1
GSPATT00021275001	1,322	0,00075	1
GSPATT00021294001	-0,03	0,86443	0
GSPATT00021304001	-0,938	0,00125	0
GSPATT00021337001	1,12	0,00026	1
GSPATT00021346001	0,927	0,00175	0
GSPATT00021382001	0,681	0,00268	0
GSPATT00021397001	-0,341	0,04105	0
GSPATT00021403001	0,008	0,96802	0
GSPATT00021407001	0,546	0,01042	0
GSPATT00021410001	0,546	0,03562	0
GSPATT00021416001	0,262	0,11135	0
GSPATT00021423001	0,41	0,01616	0
GSPATT00021453001	0,568	0,01115	0
GSPATT00021496001	-0,121	0,56931	0
GSPATT00021511001	0,607	0,01408	0
GSPATT00021521001	0,151	0,3119	0
GSPATT00021522001	0,222	0,20683	0
GSPATT00021531001	1,595	0	1
GSPATT00021532001	0,469	0,01357	0
GSPATT00021537001	0,203	0,36082	0

GSPATT00002429001	0,235	0,11218	0
GSPATT00002438001	0,165	0,29594	0
GSPATT00002465001	0,13	0,62332	0
GSPATT00002480001	1,183	0,00004	1
GSPATT00002495001	0,174	0,40222	0
GSPATT00002529001	1,935	0	1
GSPATT00002540001	0,687	0,01064	0
GSPATT00002550001	-0,042	0,75069	0
GSPATT00002553001	0,322	0,07357	0
GSPATT00002575001	0,758	0,01395	0
GSPATT00002585001	0,436	0,08223	0
GSPATT00002589001	0,394	0,03041	0
GSPATT00002593001	1,076	0,0001	1
GSPATT00002595001	0,321	0,0482	0
GSPATT00002614001	-0,048	0,8212	0
GSPATT00002618001	0,222	0,2786	0
GSPATT00002633001	0,209	0,22321	0
GSPATT00002634001	-0,559	0,00848	0
GSPATT00002648001	-0,019	0,91239	0
GSPATT00002669001	0,347	0,04238	0
GSPATT00002670001	0,301	0,22471	0
GSPATT00002680001	1,006	0,00039	1
GSPATT00002682001	0,464	0,04413	0
GSPATT00002705001	0,629	0,00714	0
GSPATT00002720001	-1,253	0,00068	-1
GSPATT00002743001	-0,488	0,03306	0
GSPATT00002752001	0,495	0,06805	0
GSPATT00002760001	0,788	0,00112	0
GSPATT00002771001	0,859	0,0012	0
GSPATT00002776001	-0,859	0,00025	0
GSPATT00002799001	-0,188	0,27826	0
GSPATT00002813001	0,189	0,28818	0
GSPATT00002833001	-0,306	0,26468	0
GSPATT00002839001	-0,591	0,0576	0
GSPATT00002865001	-0,375	0,08216	0
GSPATT00002890001	-1,023	0,00005	-1
GSPATT00002908001	1,161	0,00015	1
GSPATT00002917001	-0,703	0,00355	0
GSPATT00002933001	-2,484	0	-1
GSPATT00002954001	-0,433	0,0165	0
GSPATT00002960001	-0,423	0,06926	0
GSPATT00002969001	-0,585	0,02587	0
GSPATT00002999001	1,266	0,00041	1
GSPATT00003037001	0,383	0,02776	0
GSPATT00003054001	0,076	0,56297	0
GSPATT00003075001	0,629	0,02375	0
GSPATT00003077001	-0,196	0,20764	0
GSPATT00003089001	-0,441	0,11477	0
GSPATT00003090001	-0,149	0,32277	0
GSPATT00003094001	0,49	0,01226	0
GSPATT00003105001	1,596	0,00013	1
GSPATT00003123001	0,138	0,52706	0
GSPATT00003132001	0,679	0,00156	0
GSPATT00003154001	0,332	0,08672	0
GSPATT00003156001	0,696	0,00226	0
GSPATT00003167001	0,084	0,63843	0
GSPATT00003168001	1,73	0,00037	1
GSPATT00003185001	0,267	0,31747	0
GSPATT00003191001	-0,442	0,0623	0
GSPATT00003192001	-0,143	0,32291	0
GSPATT00003194001	1,519	0,00002	1
GSPATT00003198001	0,506	0,02601	0
GSPATT00003200001	0,421	0,03094	0
GSPATT00003201001	0,867	0,00568	0
GSPATT00003224001	-0,237	0,12454	0
GSPATT00003229001	0,103	0,49036	0
GSPATT00003230001	0,337	0,26038	0
GSPATT00021547001	0,33	0,05213	0
GSPATT00021550001	0,69	0,00115	0
GSPATT00021566001	0,15	0,38869	0
GSPATT00021592001	0,574	0,00334	0
GSPATT00021605001	-0,285	0,07981	0
GSPATT00021623001	-0,006	0,97564	0
GSPATT00021625001	-0,609	0,00663	0
GSPATT00021637001	0,858	0,0012	0
GSPATT00021639001	1,696	0,00085	1
GSPATT00021643001	0,057	0,70009	0
GSPATT00021645001	0,312	0,06296	0
GSPATT00021666001	-0,927	0,0001	0
GSPATT00021705001	0,593	0,01376	0
GSPATT00021709001	1,146	0,00013	1
GSPATT00021737001	2,607	0,00001	1
GSPATT00021745001	-0,479	0,00827	0
GSPATT00021748001	0,772	0,0086	0
GSPATT00021749001	1,147	0,01618	1
GSPATT00021752001	0,314	0,06862	0
GSPATT00021753001	0,418	0,07228	0
GSPATT00021754001	0,243	0,14094	0
GSPATT00021761001	0,639	0,00204	0
GSPATT00021763001	1,74	0,00004	1
GSPATT00021786001	0,144	0,35607	0
GSPATT00021789001	1,156	0,00096	1
GSPATT00021797001	0,664	0,0087	0
GSPATT00021815001	0,094	0,7025	0
GSPATT00021818001	0,452	0,0106	0
GSPATT00021839001	-0,624	0,00196	0
GSPATT00021843001	-0,573	0,01408	0
GSPATT00021861001	-2,393	0,00001	-1
GSPATT00021876001	0,606	0,05246	0
GSPATT00021880001	0,418	0,06147	0
GSPATT00021882001	-0,509	0,02829	0
GSPATT00021885001	0,508	0,01902	0
GSPATT00021893001	1,556	0,00001	1
GSPATT00021905001	0,176	0,26244	0
GSPATT00021907001	-0,742	0,0007	0
GSPATT00021930001	-0,548	0,007	0
GSPATT00021935001	0,444	0,0187	0
GSPATT00021944001	-1,181	0,00069	-1
GSPATT00021946001	0,249	0,20733	0
GSPATT00021955001	1,15	0,00027	1
GSPATT00021964001	0,822	0,01015	0
GSPATT00021978001	-0,527	0,00915	0
GSPATT00021986001	1,819	0,00001	1
GSPATT00022016001	0,205	0,15716	0
GSPATT00022037001	0,79	0,00181	0
GSPATT00022042001	0,34	0,05228	0
GSPATT00022061001	-0,298	0,11655	0
GSPATT00022128001	-0,88	0,0016	0
GSPATT00022131001	-0,083	0,72879	0
GSPATT00022141001	1,027	0,00014	1
GSPATT00022154001	0,346	0,07096	0
GSPATT00022155001	0,511	0,00951	0
GSPATT00022167001	0,329	0,07157	0
GSPATT00022172001	0,608	0,01725	0
GSPATT00022183001	-0,244	0,23912	0
GSPATT00022205001	0,366	0,11152	0
GSPATT00022214001	0,659	0,00671	0
GSPATT00022218001	-0,466	0,01328	0
GSPATT00022236001	1,845	0,00007	1
GSPATT00022240001	0,698	0,0019	0
GSPATT00022249001	0,454	0,00814	0
GSPATT00022251001	-0,514	0,08066	0
GSPATT00022256001	0,89	0,0088	0
GSPATT00022278001	0,113	0,6617	0

GSPATT00003236001	1,111	0,00005	1	GSPATT00022287001	0,434	0,03622	0
GSPATT00003248001	-0,663	0,0009	0	GSPATT00022290001	1,407	0,00034	1
GSPATT00003253001	0,103	0,43872	0	GSPATT00022296001	1,798	0,00001	1
GSPATT00003279001	0,867	0,0006	0	GSPATT00022304001	0,901	0,00027	0
GSPATT00003280001	-0,04	0,80148	0	GSPATT00022316001	0,995	0,0013	0
GSPATT00003283001	0,264	0,25078	0	GSPATT00022319001	-0,149	0,47401	0
GSPATT00003292001	0,915	0,00467	0	GSPATT00022364001	0,012	0,94018	0
GSPATT00003309001	-0,973	0,0002	0	GSPATT00022375001	0,335	0,05369	0
GSPATT00003326001	-0,051	0,69934	0	GSPATT00022376001	0,282	0,36231	0
GSPATT00003335001	-0,087	0,60586	0	GSPATT00022389001	0,267	0,0861	0
GSPATT00003339001	0,266	0,15758	0	GSPATT00022398001	-0,303	0,11489	0
GSPATT00003344001	0,549	0,00644	0	GSPATT00022415001	0,64	0,00116	0
GSPATT00003348001	-0,128	0,5085	0	GSPATT00022426001	0,639	0,00648	0
GSPATT00003366001	0,829	0,00229	0	GSPATT00022436001	-0,065	0,75967	0
GSPATT00003407001	0,787	0,00042	0	GSPATT00022478001	-0,264	0,12121	0
GSPATT00003425001	-0,517	0,01811	0	GSPATT00022479001	-0,83	0,0106	0
GSPATT00003486001	0,113	0,47243	0	GSPATT00022490001	0,953	0,00048	0
GSPATT00003501001	0,158	0,34108	0	GSPATT00022560001	0,407	0,01304	0
GSPATT00003507001	-1,565	0,00001	-1	GSPATT00022581001	0,197	0,34705	0
GSPATT00003528001	0,505	0,01812	0	GSPATT00022585001	-0,137	0,38681	0
GSPATT00003535001	0,273	0,06967	0	GSPATT00022588001	0,519	0,01581	0
GSPATT00003620001	0,88	0,00103	0	GSPATT00022625001	-0,631	0,00225	0
GSPATT00003647001	0,327	0,21564	0	GSPATT00022648001	1,169	0,00006	1
GSPATT00003651001	0,62	0,00494	0	GSPATT00022652001	0,216	0,20385	0
GSPATT00003653001	-0,158	0,36535	0	GSPATT00022660001	0,792	0,00748	0
GSPATT00003657001	-0,746	0,00329	0	GSPATT00022665001	0,156	0,24856	0
GSPATT00003674001	0,666	0,18862	0	GSPATT00022666001	1,059	0,00175	1
GSPATT00003681001	-0,103	0,4827	0	GSPATT00022671001	0,453	0,06689	0
GSPATT00003682001	1,134	0,00047	1	GSPATT00022672001	0,392	0,0156	0
GSPATT00003692001	0,249	0,08305	0	GSPATT00022676001	0,956	0,00118	0
GSPATT00003699001	-0,208	0,19088	0	GSPATT00022680001	0,88	0,01013	0
GSPATT00003701001	-1,324	0,00003	-1	GSPATT00022747001	-0,042	0,84234	0
GSPATT00003711001	-0,057	0,78591	0	GSPATT00022758001	-0,264	0,37366	0
GSPATT00003719001	-2,965	0	-1	GSPATT00022760001	-0,332	0,06229	0
GSPATT00003722001	-0,214	0,14285	0	GSPATT00022772001	0,587	0,00225	0
GSPATT00003724001	-1,06	0,00023	-1	GSPATT00022792001	0,634	0,00443	0
GSPATT00003735001	0,938	0,00036	0	GSPATT00022794001	-0,642	0,00449	0
GSPATT00003784001	-0,762	0,00155	0	GSPATT00022804001	0,571	0,02711	0
GSPATT00003800001	0,707	0,00325	0	GSPATT00022805001	0,721	0,00384	0
GSPATT00003805001	-0,458	0,01837	0	GSPATT00022813001	-0,287	0,25917	0
GSPATT00003808001	-1,655	0,00001	-1	GSPATT00022862001	0,196	0,23874	0
GSPATT00003833001	0,314	0,13153	0	GSPATT00022877001	0,756	0,0172	0
GSPATT00003834001	0,118	0,39846	0	GSPATT00022923001	0,087	0,54996	0
GSPATT00003866001	0,17	0,22326	0	GSPATT00022959001	1,261	0,00015	1
GSPATT00003883001	1,07	0,00005	1	GSPATT00022960001	-0,158	0,24845	0
GSPATT00003889001	1,021	0,00011	1	GSPATT00022980001	0,614	0,0183	0
GSPATT00003892001	-0,172	0,33783	0	GSPATT00022982001	0,79	0,04014	0
GSPATT00003941001	-0,015	0,90753	0	GSPATT00023007001	0,391	0,03401	0
GSPATT00003946001	0,029	0,8323	0	GSPATT00023011001	0,775	0,00132	0
GSPATT00003963001	0,533	0,01029	0	GSPATT00023034001	-0,277	0,07151	0
GSPATT00003987001	0,889	0,00018	0	GSPATT00023053001	0,174	0,19072	0
GSPATT00004011001	0,459	0,0181	0	GSPATT00023054001	0,175	0,21904	0
GSPATT00004041001	-2,104	0,00001	-1	GSPATT00023076001	0,772	0,00846	0
GSPATT00004054001	0,449	0,01063	0	GSPATT00023086001	0,21	0,20719	0
GSPATT00004057001	-0,473	0,03309	0	GSPATT00023101001	0,252	0,21939	0
GSPATT00004059001	0,026	0,88876	0	GSPATT00023116001	0,243	0,2874	0
GSPATT00004060001	0,495	0,02651	0	GSPATT00023168001	-0,346	0,10457	0
GSPATT00004091001	0,299	0,07171	0	GSPATT00023209001	0,505	0,02572	0
GSPATT00004095001	-0,095	0,61368	0	GSPATT00023226001	-0,09	0,6793	0
GSPATT00004114001	0,269	0,09228	0	GSPATT00023238001	-0,52	0,00932	0
GSPATT00004132001	-0,103	0,69251	0	GSPATT00023249001	0,903	0,00029	0
GSPATT00004152001	0,206	0,29595	0	GSPATT00023269001	-0,248	0,34866	0
GSPATT00004205001	-0,31	0,17947	0	GSPATT00023282001	-0,358	0,04566	0
GSPATT00004210001	0,416	0,03288	0	GSPATT00023287001	-0,508	0,01292	0
GSPATT00004223001	0,612	0,00738	0	GSPATT00023291001	-0,368	0,10871	0
GSPATT00004225001	0,089	0,74057	0	GSPATT00023309001	-0,41	0,01263	0
GSPATT00004229001	-0,224	0,29312	0	GSPATT00023311001	0,38	0,03477	0

GSPATT00004247001	0,159	0,57122	0	GSPATT00023324001	0,902	0,00067	0
GSPATT00004268001	1,307	0,00011	1	GSPATT00023325001	0,851	0,00031	0
GSPATT00004273001	-0,717	0,00174	0	GSPATT00023332001	0,059	0,7739	0
GSPATT00004298001	0,13	0,50582	0	GSPATT00023391001	0,729	0,00128	0
GSPATT00004321001	0,151	0,6363	0	GSPATT00023409001	-0,413	0,17182	0
GSPATT00004366001	0,068	0,69186	0	GSPATT00023417001	0,386	0,08109	0
GSPATT00004368001	0,323	0,16554	0	GSPATT00023422001	-0,559	0,02664	0
GSPATT00004376001	0,343	0,08104	0	GSPATT00023426001	-0,016	0,92617	0
GSPATT00004396001	0,315	0,11278	0	GSPATT00023427001	0,645	0,027	0
GSPATT00004405001	0,153	0,39785	0	GSPATT00023429001	0,253	0,18096	0
GSPATT00004412001	-1,59	0,00001	-1	GSPATT00023436001	0,755	0,04632	0
GSPATT00004442001	-0,201	0,48124	0	GSPATT00023438001	0,035	0,83414	0
GSPATT00004447001	0,383	0,05161	0	GSPATT00023469001	-0,011	0,95408	0
GSPATT00004496001	0,955	0,00187	0	GSPATT00023488001	1,084	0,00116	1
GSPATT00004514001	0,476	0,02673	0	GSPATT00023514001	0,507	0,00757	0
GSPATT00004528001	0,66	0,00397	0	GSPATT00023531001	0,22	0,46511	0
GSPATT00004559001	0,711	0,00505	0	GSPATT00023540001	0,273	0,10052	0
GSPATT00004570001	0,195	0,22266	0	GSPATT00023549001	-1,224	0,00167	-1
GSPATT00004575001	-0,004	0,97788	0	GSPATT00023569001	-0,979	0,00048	0
GSPATT00004580001	1,242	0,00014	1	GSPATT00023571001	-0,22	0,33947	0
GSPATT00004590001	0,499	0,01576	0	GSPATT00023576001	-0,089	0,51115	0
GSPATT00004598001	0,48	0,02633	0	GSPATT00023613001	0,192	0,1796	0
GSPATT00004600001	0,083	0,57831	0	GSPATT00023618001	-0,03	0,8305	0
GSPATT00004601001	1,962	0,0001	1	GSPATT00023627001	0,203	0,23093	0
GSPATT00004610001	0,945	0,00014	0	GSPATT00023631001	0,239	0,10083	0
GSPATT00004630001	-0,933	0,00013	0	GSPATT00023632001	0,122	0,36366	0
GSPATT00004644001	-2,245	0,00001	-1	GSPATT00023667001	-0,421	0,04545	0
GSPATT00004661001	-0,088	0,65447	0	GSPATT00023676001	0,145	0,31782	0
GSPATT00004664001	0,401	0,16244	0	GSPATT00023690001	-1,078	0,0043	-1
GSPATT00004681001	-0,284	0,11177	0	GSPATT00023719001	-0,472	0,04584	0
GSPATT00004693001	0,654	0,00623	0	GSPATT00023728001	0,102	0,45091	0
GSPATT00004716001	-0,265	0,1283	0	GSPATT00023729001	0,5	0,071	0
GSPATT00004725001	-0,044	0,79332	0	GSPATT00023735001	0,573	0,00261	0
GSPATT00004760001	0,235	0,3263	0	GSPATT00023790001	-0,309	0,06093	0
GSPATT00004761001	0,808	0,01968	0	GSPATT00023792001	0,035	0,82314	0
GSPATT00004764001	-0,15	0,28228	0	GSPATT00023807001	-0,378	0,04035	0
GSPATT00004777001	1,749	0,00006	1	GSPATT00023823001	-0,725	0,00571	0
GSPATT00004785001	0,782	0,00521	0	GSPATT00023843001	0,248	0,16073	0
GSPATT00004807001	0,124	0,60039	0	GSPATT00023861001	1,352	0,00004	1
GSPATT00004813001	0,679	0,01555	0	GSPATT00023871001	-0,181	0,31269	0
GSPATT00004832001	-0,277	0,07921	0	GSPATT00023901001	0,661	0,00244	0
GSPATT00004834001	0,789	0,00098	0	GSPATT00023907001	0,749	0,00073	0
GSPATT00004846001	2,635	0	1	GSPATT00023937001	-0,496	0,07124	0
GSPATT00004860001	-0,019	0,91882	0	GSPATT00023947001	0,21	0,19097	0
GSPATT00004865001	-0,645	0,01547	0	GSPATT00023951001	0,462	0,01336	0
GSPATT00004869001	0,682	0,00693	0	GSPATT00023962001	-0,49	0,01746	0
GSPATT00004874001	-0,141	0,55683	0	GSPATT00023981001	0,056	0,75794	0
GSPATT00004901001	0,489	0,04492	0	GSPATT00024046001	0,488	0,06203	0
GSPATT00004905001	0,24	0,28818	0	GSPATT00024078001	0,879	0,0027	0
GSPATT00004906001	0,041	0,80854	0	GSPATT00024080001	0,962	0,00084	0
GSPATT00004925001	-0,765	0,01316	0	GSPATT00024093001	0,589	0,00369	0
GSPATT00004954001	-0,94	0,02171	0	GSPATT00024120001	0,414	0,05081	0
GSPATT00004968001	0,709	0,00121	0	GSPATT00024181001	0,193	0,36401	0
GSPATT00004976001	-0,023	0,86985	0	GSPATT00024182001	-0,819	0,01549	0
GSPATT00004988001	-0,207	0,1401	0	GSPATT00024183001	-0,176	0,34939	0
GSPATT00004998001	0,877	0,0004	0	GSPATT00024185001	1,039	0,00005	1
GSPATT00005003001	0,289	0,06439	0	GSPATT00024203001	0,349	0,11648	0
GSPATT00005007001	0,56	0,00815	0	GSPATT00024205001	0,66	0,00203	0
GSPATT00005009001	0,54	0,00742	0	GSPATT00024206001	-0,177	0,2138	0
GSPATT00005077001	0,271	0,1489	0	GSPATT00024209001	1,188	0,00003	1
GSPATT00005093001	0,879	0,00087	0	GSPATT00024218001	-0,321	0,04683	0
GSPATT00005095001	0,782	0,00596	0	GSPATT00024233001	0,949	0,0003	0
GSPATT00005100001	-0,27	0,06839	0	GSPATT00024234001	0,863	0,00016	0
GSPATT00005117001	0,136	0,40124	0	GSPATT00024244001	0,136	0,47521	0
GSPATT00005120001	0,8	0,00223	0	GSPATT00024253001	0,327	0,12765	0
GSPATT00005130001	-0,006	0,97738	0	GSPATT00024274001	0,022	0,87189	0
GSPATT00005209001	1,585	0,00011	1	GSPATT00024309001	-0,157	0,28831	0

GSPATT00005243001	1,145	0,0018	1	GSPATT00024335001	-0,34	0,04551	0
GSPATT00005245001	0,407	0,07978	0	GSPATT00024337001	0,437	0,02363	0
GSPATT00005247001	-0,102	0,43462	0	GSPATT00024348001	-0,075	0,56089	0
GSPATT00005248001	-1,692	0,00037	-1	GSPATT00024349001	0,096	0,597	0
GSPATT00005269001	0,265	0,06576	0	GSPATT00024352001	0,614	0,00859	0
GSPATT00005271001	-0,185	0,35608	0	GSPATT00024357001	0,148	0,45854	0
GSPATT00005274001	0,558	0,00601	0	GSPATT00024361001	0,391	0,03397	0
GSPATT00005284001	0,206	0,14532	0	GSPATT00024377001	1,043	0,00342	1
GSPATT00005295001	0,7	0,00631	0	GSPATT00024390001	-0,282	0,13976	0
GSPATT00005301001	0,612	0,02995	0	GSPATT00024398001	0,796	0,01758	0
GSPATT00005326001	1,016	0,00028	1	GSPATT00024410001	0,428	0,02489	0
GSPATT00005329001	0,523	0,01314	0	GSPATT00024418001	0,221	0,13987	0
GSPATT00005343001	-0,049	0,73244	0	GSPATT00024424001	-0,29	0,0846	0
GSPATT00005348001	0,239	0,13893	0	GSPATT00024429001	0,278	0,07692	0
GSPATT00005359001	-0,071	0,70866	0	GSPATT00024448001	-0,278	0,17393	0
GSPATT00005382001	0,315	0,08827	0	GSPATT00024451001	0,393	0,03105	0
GSPATT00005394001	-2,476	0	-1	GSPATT00024479001	-0,903	0,00303	0
GSPATT00005416001	-0,283	0,0906	0	GSPATT00024481001	0,435	0,1006	0
GSPATT00005443001	1,201	0,01436	1	GSPATT00024498001	0,272	0,35558	0
GSPATT00005458001	-0,786	0,00076	0	GSPATT00024499001	0,56	0,00549	0
GSPATT00005498001	0,586	0,00534	0	GSPATT00024504001	-0,557	0,06748	0
GSPATT00005504001	-0,323	0,07461	0	GSPATT00024512001	1,298	0,00001	1
GSPATT00005531001	0,024	0,88617	0	GSPATT00024514001	0,748	0,01065	0
GSPATT00005533001	-0,809	0,00171	0	GSPATT00024536001	0,01	0,96698	0
GSPATT00005536001	0,887	0,00254	0	GSPATT00024539001	-2,028	0	-1
GSPATT00005555001	0,146	0,36762	0	GSPATT00024546001	-0,401	0,04144	0
GSPATT00005556001	0,344	0,07104	0	GSPATT00024564001	0,433	0,05933	0
GSPATT00005557001	0,873	0,00334	0	GSPATT00024565001	1,115	0,00008	1
GSPATT00005568001	0,44	0,03873	0	GSPATT00024566001	0,629	0,00565	0
GSPATT00005581001	0,156	0,32591	0	GSPATT00024591001	0,498	0,00429	0
GSPATT00005587001	0,343	0,06896	0	GSPATT00024618001	0,097	0,49798	0
GSPATT00005611001	0,102	0,61679	0	GSPATT00024644001	0,654	0,00896	0
GSPATT00005614001	0,401	0,02429	0	GSPATT00024673001	-0,186	0,23745	0
GSPATT00005647001	-0,353	0,0216	0	GSPATT00024691001	1,052	0,00127	1
GSPATT00005692001	-0,035	0,79461	0	GSPATT00024731001	0,285	0,24363	0
GSPATT00005703001	0,426	0,05301	0	GSPATT00024737001	0,421	0,14567	0
GSPATT00005726001	0,372	0,0779	0	GSPATT00024766001	0,352	0,11206	0
GSPATT00005733001	0,017	0,92434	0	GSPATT00024788001	0,153	0,34111	0
GSPATT00005734001	0,285	0,09036	0	GSPATT00024803001	0,179	0,50259	0
GSPATT00005738001	0,978	0,00023	0	GSPATT00024859001	1,158	0,00272	1
GSPATT00005752001	1,537	0,00004	1	GSPATT00024862001	0,232	0,28545	0
GSPATT00005757001	0,742	0,00204	0	GSPATT00024885001	-1,562	0,00004	-1
GSPATT00005760001	-0,591	0,07206	0	GSPATT00024889001	0,107	0,58905	0
GSPATT00005782001	0,537	0,00527	0	GSPATT00024895001	-0,166	0,43815	0
GSPATT00005787001	0,145	0,47364	0	GSPATT00024913001	-0,542	0,01406	0
GSPATT00005804001	-2,483	0,00045	-1	GSPATT00024916001	1,07	0,00006	1
GSPATT00005811001	0,498	0,03959	0	GSPATT00024917001	1,027	0,0002	1
GSPATT00005814001	-0,041	0,82667	0	GSPATT00024942001	-0,918	0,00043	0
GSPATT00005845001	0,153	0,32908	0	GSPATT00024976001	0,84	0,00119	0
GSPATT00005881001	0,784	0,01296	0	GSPATT00024990001	0,807	0,0027	0
GSPATT00005902001	1,463	0,00021	1	GSPATT00025030001	-0,48	0,01979	0
GSPATT00005904001	0,174	0,28531	0	GSPATT00025035001	1,087	0,00028	1
GSPATT00005934001	0,631	0,00222	0	GSPATT00025044001	0,229	0,26111	0
GSPATT00005943001	1,376	0,00047	1	GSPATT00025071001	-0,063	0,7592	0
GSPATT00005951001	-0,034	0,81726	0	GSPATT00025076001	0,189	0,30656	0
GSPATT00005960001	1,544	0,00007	1	GSPATT00025088001	0,087	0,7199	0
GSPATT00005995001	0,249	0,17873	0	GSPATT00025089001	0,396	0,07801	0
GSPATT00006007001	0,509	0,01301	0	GSPATT00025098001	0,473	0,02724	0
GSPATT00006011001	0,426	0,04714	0	GSPATT00025106001	0,702	0,01194	0
GSPATT00006030001	0,381	0,05068	0	GSPATT00025128001	0,313	0,11606	0
GSPATT00006033001	0,654	0,0075	0	GSPATT00025133001	0,871	0,00255	0
GSPATT00006057001	-0,917	0,00035	0	GSPATT00025144001	1,216	0,00018	1
GSPATT00006075001	0,152	0,28079	0	GSPATT00025176001	0,858	0,01028	0
GSPATT00006080001	0,359	0,11534	0	GSPATT00025177001	0,141	0,61521	0
GSPATT00006094001	0,938	0,00214	0	GSPATT00025184001	0,545	0,03088	0
GSPATT00006105001	0,219	0,41076	0	GSPATT00025243001	1,014	0,00008	1
GSPATT00006125001	0,447	0,0539	0	GSPATT00025254001	0,909	0,00035	0

GSPATT00006174001	-1,189	0,00097	-1	GSPATT00025257001	0,582	0,02537	0
GSPATT00006199001	-0,122	0,43015	0	GSPATT00025262001	-0,617	0,0118	0
GSPATT00006226001	-0,062	0,65778	0	GSPATT00025273001	1,277	0,00074	1
GSPATT00006272001	0,621	0,03899	0	GSPATT00025348001	0,917	0,00082	0
GSPATT00006280001	0,726	0,00083	0	GSPATT00025382001	-0,664	0,00239	0
GSPATT00006296001	0,129	0,52836	0	GSPATT00025431001	0,45	0,04878	0
GSPATT00006297001	0,413	0,01659	0	GSPATT00025434001	2,076	0	1
GSPATT00006313001	0,809	0,01243	0	GSPATT00025443001	0,397	0,05816	0
GSPATT00006314001	0,607	0,00237	0	GSPATT00025450001	0,01	0,9566	0
GSPATT00006315001	0,211	0,21775	0	GSPATT00025459001	0,443	0,0356	0
GSPATT00006331001	0,84	0,00169	0	GSPATT00025471001	-1,355	0,00017	-1
GSPATT00006334001	-0,044	0,80576	0	GSPATT00025473001	1,303	0,00076	1
GSPATT00006378001	0,677	0,00317	0	GSPATT00025481001	-0,394	0,02039	0
GSPATT00006379001	0,429	0,03699	0	GSPATT00025487001	0,527	0,00492	0
GSPATT00006390001	-3,443	0	-1	GSPATT00025488001	0,117	0,44687	0
GSPATT00006408001	-0,255	0,28163	0	GSPATT00025542001	0,154	0,32238	0
GSPATT00006415001	-0,218	0,29617	0	GSPATT00025564001	0,335	0,03391	0
GSPATT00006446001	-0,06	0,78799	0	GSPATT00025565001	0,102	0,44835	0
GSPATT00006468001	1,644	0,00007	1	GSPATT00025585001	0,189	0,32528	0
GSPATT00006472001	-0,821	0,00056	0	GSPATT00025621001	0,549	0,02246	0
GSPATT00006519001	1,503	0,00026	1	GSPATT00025624001	1,872	0,00011	1
GSPATT00006522001	0,067	0,61368	0	GSPATT00025626001	0,186	0,22873	0
GSPATT00006526001	0,784	0,00476	0	GSPATT00025634001	-0,572	0,0037	0
GSPATT00006544001	0,253	0,22418	0	GSPATT00025641001	-0,136	0,41303	0
GSPATT00006575001	-0,075	0,7515	0	GSPATT00025658001	0,311	0,08053	0
GSPATT00006576001	0,119	0,43904	0	GSPATT00025660001	0,423	0,01588	0
GSPATT00006580001	0,511	0,15256	0	GSPATT00025674001	-0,467	0,00665	0
GSPATT00006581001	-0,025	0,86132	0	GSPATT00025677001	0,628	0,0033	0
GSPATT00006592001	0,443	0,14222	0	GSPATT00025683001	1,103	0,00903	1
GSPATT00006594001	2,357	0	1	GSPATT00025688001	0,986	0,00101	0
GSPATT00006615001	0,212	0,32587	0	GSPATT00025714001	-0,285	0,11387	0
GSPATT00006642001	1,159	0,00005	1	GSPATT00025724001	-0,118	0,54796	0
GSPATT00006653001	0,808	0,00058	0	GSPATT00025742001	0,095	0,63776	0
GSPATT00006659001	-0,504	0,0224	0	GSPATT00025754001	1,693	0	1
GSPATT00006681001	1,225	0,00002	1	GSPATT00025790001	0,722	0,01137	0
GSPATT00006690001	0,625	0,00374	0	GSPATT00025829001	-0,303	0,13502	0
GSPATT00006702001	1,003	0,00049	1	GSPATT00025830001	-0,219	0,47777	0
GSPATT00006707001	0,096	0,55283	0	GSPATT00025833001	0,318	0,12793	0
GSPATT00006718001	-1,206	0,0008	-1	GSPATT00025835001	0,577	0,00646	0
GSPATT00006721001	0,259	0,28365	0	GSPATT00025843001	-0,234	0,1386	0
GSPATT00006725001	1,26	0,00008	1	GSPATT00025874001	0,078	0,61912	0
GSPATT00006735001	1,208	0,00017	1	GSPATT00025891001	0,036	0,78724	0
GSPATT00006746001	0,845	0,00197	0	GSPATT00025892001	0,813	0,00112	0
GSPATT00006769001	0,213	0,32339	0	GSPATT00025893001	0,184	0,26595	0
GSPATT00006775001	0,248	0,09259	0	GSPATT00025925001	-2,389	0,00001	-1
GSPATT00006782001	0,148	0,27067	0	GSPATT00025938001	0,244	0,21258	0
GSPATT00006852001	0,834	0,00426	0	GSPATT00025977001	0,266	0,20896	0
GSPATT00006853001	0,531	0,04301	0	GSPATT00025987001	0,777	0,00304	0
GSPATT00006854001	0,961	0,00034	0	GSPATT00025999001	0,214	0,18023	0
GSPATT00006864001	0,6	0,00924	0	GSPATT00026028001	-0,597	0,02174	0
GSPATT00006884001	0,278	0,14598	0	GSPATT00026044001	1,043	0,00011	1
GSPATT00006904001	0,29	0,11816	0	GSPATT00026048001	0,131	0,45105	0
GSPATT00006927001	0,501	0,00669	0	GSPATT00026052001	0,22	0,26869	0
GSPATT00006928001	0,334	0,0274	0	GSPATT00026100001	-0,727	0,05157	0
GSPATT00006948001	0,353	0,03894	0	GSPATT00026117001	0,711	0,00252	0
GSPATT00006949001	0,568	0,03228	0	GSPATT00026130001	0,041	0,84926	0
GSPATT00006953001	0,189	0,35123	0	GSPATT00026158001	-0,019	0,89138	0
GSPATT00006969001	0,405	0,11317	0	GSPATT00026167001	0,538	0,00916	0
GSPATT00006974001	0,738	0,04036	0	GSPATT00026169001	0,593	0,0211	0
GSPATT00007015001	-0,04	0,8039	0	GSPATT00026182001	0,405	0,01463	0
GSPATT00007020001	0,699	0,0179	0	GSPATT00026208001	-0,305	0,05561	0
GSPATT00007023001	0,031	0,83742	0	GSPATT00026214001	0,126	0,51347	0
GSPATT00007028001	1,888	0,00035	1	GSPATT00026231001	-0,721	0,00236	0
GSPATT00007036001	-1,628	0,00205	-1	GSPATT00026239001	0,204	0,24495	0
GSPATT00007037001	0,21	0,21843	0	GSPATT00026269001	-0,827	0,00039	0
GSPATT00007044001	-0,041	0,75732	0	GSPATT00026274001	0,081	0,55749	0
GSPATT00007068001	-0,215	0,22845	0	GSPATT00026292001	0,175	0,24434	0

GSPATT00007081001	-0,453	0,09098	0	GSPATT00026323001	-0,937	0,00078	0
GSPATT00007084001	1,343	0,0001	1	GSPATT00026336001	-0,538	0,01977	0
GSPATT00007085001	0,898	0,00027	0	GSPATT00026360001	1,582	0,00001	1
GSPATT00007086001	-0,397	0,12088	0	GSPATT00026362001	0,039	0,7821	0
GSPATT00007091001	1,465	0,00001	1	GSPATT00026366001	0,255	0,08585	0
GSPATT00007092001	0,27	0,19194	0	GSPATT00026371001	0,968	0,00219	0
GSPATT00007095001	0,892	0,00182	0	GSPATT00026384001	0,13	0,49373	0
GSPATT00007110001	0,57	0,00537	0	GSPATT00026401001	0,404	0,01576	0
GSPATT00007113001	0,035	0,79386	0	GSPATT00026410001	-1,354	0,00004	-1
GSPATT00007130001	0,976	0,0003	0	GSPATT00026424001	-0,195	0,41235	0
GSPATT00007151001	0,29	0,08359	0	GSPATT00026435001	0,978	0,0003	0
GSPATT00007162001	-0,046	0,76877	0	GSPATT00026456001	-0,112	0,49348	0
GSPATT00007168001	2,307	0	1	GSPATT00026471001	0,689	0,0725	0
GSPATT00007170001	0,256	0,39221	0	GSPATT00026484001	0,101	0,56577	0
GSPATT00007176001	0,545	0,0517	0	GSPATT00026498001	0,273	0,11056	0
GSPATT00007193001	-0,121	0,39214	0	GSPATT00026501001	0,573	0,02563	0
GSPATT00007196001	0,471	0,05051	0	GSPATT00026528001	0,335	0,1504	0
GSPATT00007206001	-0,243	0,15376	0	GSPATT00026582001	-0,61	0,00172	0
GSPATT00007221001	-0,047	0,73213	0	GSPATT00026598001	-0,409	0,02421	0
GSPATT00007228001	-0,424	0,07621	0	GSPATT00026607001	0,518	0,00768	0
GSPATT00007243001	0,258	0,11177	0	GSPATT00026611001	0,416	0,09592	0
GSPATT00007288001	-0,479	0,02516	0	GSPATT00026620001	-0,023	0,89953	0
GSPATT00007295001	1,711	0	1	GSPATT00026642001	0,375	0,12827	0
GSPATT00007306001	0,194	0,49349	0	GSPATT00026656001	0,014	0,92729	0
GSPATT00007307001	0,539	0,00329	0	GSPATT00026672001	0,656	0,00939	0
GSPATT00007337001	0,377	0,05996	0	GSPATT00026673001	1,475	0,00074	1
GSPATT00007343001	0,329	0,28753	0	GSPATT00026685001	-0,162	0,49003	0
GSPATT00007357001	-0,154	0,51852	0	GSPATT00026711001	-1,004	0,00014	-1
GSPATT00007369001	-0,3	0,09662	0	GSPATT00026728001	0,421	0,03621	0
GSPATT00007385001	0,031	0,87634	0	GSPATT00026733001	-0,183	0,2048	0
GSPATT00007396001	0,747	0,00516	0	GSPATT00026736001	0,014	0,9335	0
GSPATT00007424001	0,679	0,0528	0	GSPATT00026737001	-0,589	0,01656	0
GSPATT00007458001	-0,178	0,41913	0	GSPATT00026742001	1,681	0,00062	1
GSPATT00007469001	0,74	0,00278	0	GSPATT00026751001	0,217	0,23484	0
GSPATT00007491001	0,204	0,26477	0	GSPATT00026762001	0,081	0,72109	0
GSPATT00007506001	3,323	0	1	GSPATT00026768001	1,3	0,0016	1
GSPATT00007552001	-0,289	0,16464	0	GSPATT00026770001	0,781	0,00036	0
GSPATT00007558001	0,513	0,05289	0	GSPATT00026784001	0,447	0,02348	0
GSPATT00007569001	-0,059	0,72729	0	GSPATT00026817001	-0,512	0,01273	0
GSPATT00007633001	0,585	0,00705	0	GSPATT00026853001	-0,021	0,90542	0
GSPATT00007636001	0,951	0,00013	0	GSPATT00026855001	-0,189	0,23205	0
GSPATT00007651001	1,374	0,00001	1	GSPATT00026879001	0,638	0,0089	0
GSPATT00007652001	0,604	0,00281	0	GSPATT00026882001	0,229	0,14149	0
GSPATT00007655001	1,115	0,00032	1	GSPATT00026903001	0,385	0,04693	0
GSPATT00007660001	-2,547	0	-1	GSPATT00026918001	0,426	0,01186	0
GSPATT00007679001	-0,046	0,77776	0	GSPATT00026931001	0,327	0,03994	0
GSPATT00007689001	0,756	0,0049	0	GSPATT00026977001	0,418	0,01985	0
GSPATT00007699001	0,698	0,00069	0	GSPATT00027082001	-0,14	0,42006	0
GSPATT00007760001	0,871	0,00045	0	GSPATT00027084001	-2,917	0	-1
GSPATT00007792001	0,379	0,04772	0	GSPATT00027094001	-0,238	0,23457	0
GSPATT00007797001	0,346	0,07773	0	GSPATT00027100001	0,499	0,06172	0
GSPATT00007801001	0,358	0,05841	0	GSPATT00027119001	0,768	0,00101	0
GSPATT00007808001	0,931	0,00109	0	GSPATT00027122001	-0,502	0,01341	0
GSPATT00007826001	0,485	0,01651	0	GSPATT00027138001	0,18	0,19509	0
GSPATT00007852001	0,616	0,00193	0	GSPATT00027147001	-0,082	0,6483	0
GSPATT00007857001	1,41	0,00014	1	GSPATT00027149001	0,147	0,32384	0
GSPATT00007860001	0,07	0,62078	0	GSPATT00027156001	0,039	0,76929	0
GSPATT00007861001	0,494	0,03195	0	GSPATT00027189001	0,049	0,79446	0
GSPATT00007899001	0,019	0,90526	0	GSPATT00027203001	0,063	0,64969	0
GSPATT00007911001	-0,929	0,02523	0	GSPATT00027205001	-0,221	0,24032	0
GSPATT00007917001	0,096	0,67711	0	GSPATT00027222001	0,074	0,59482	0
GSPATT00007919001	0,035	0,87791	0	GSPATT00027241001	0,321	0,05768	0
GSPATT00007922001	0,835	0,01888	0	GSPATT00027257001	0,806	0,00465	0
GSPATT00007930001	-0,448	0,05141	0	GSPATT00027270001	0,745	0,00887	0
GSPATT00007946001	1,164	0,00002	1	GSPATT00027312001	-1,048	0,00047	-1
GSPATT00007947001	0,256	0,17572	0	GSPATT00027355001	-0,308	0,10498	0
GSPATT00007949001	1,323	0,00004	1	GSPATT00027372001	1,25	0,00004	1

GSPATT00007953001	0,179	0,38295	0
GSPATT00007957001	0,151	0,34529	0
GSPATT00007961001	-0,294	0,05705	0
GSPATT00007964001	-2,161	0,00001	-1
GSPATT00007966001	0,728	0,03307	0
GSPATT00007975001	0,396	0,04129	0
GSPATT00007989001	0,044	0,84104	0
GSPATT00007997001	0,533	0,00985	0
GSPATT00007998001	0,219	0,19699	0
GSPATT00008011001	0,371	0,20891	0
GSPATT00008014001	0,284	0,11593	0
GSPATT00008031001	-0,45	0,02946	0
GSPATT00008074001	-0,457	0,03281	0
GSPATT00008104001	1,297	0,00002	1
GSPATT00008134001	0,932	0,00055	0
GSPATT00008150001	0,063	0,64187	0
GSPATT00008151001	0,2	0,21222	0
GSPATT00008165001	0,565	0,00488	0
GSPATT00008178001	0,755	0,00688	0
GSPATT00008186001	-0,324	0,05436	0
GSPATT00008216001	0,518	0,06627	0
GSPATT00008228001	1,643	0,00002	1
GSPATT00008231001	-0,275	0,07499	0
GSPATT00008235001	-0,069	0,63056	0
GSPATT00008243001	0,87	0,00126	0
GSPATT00008313001	-3,997	0	-1
GSPATT00008318001	0,769	0,02343	0
GSPATT00008324001	0,184	0,37151	0
GSPATT00008331001	0,617	0,11575	0
GSPATT00008341001	0,816	0,00047	0
GSPATT00008356001	0,219	0,14265	0
GSPATT00008371001	0,43	0,01968	0
GSPATT00008373001	0,504	0,0091	0
GSPATT00008385001	0,841	0,01833	0
GSPATT00008408001	-0,744	0,00043	0
GSPATT00008410001	0,894	0,00015	0
GSPATT00008418001	-0,272	0,07464	0
GSPATT00008447001	-0,614	0,00381	0
GSPATT00008463001	1,266	0,00003	1
GSPATT00008472001	0,504	0,00647	0
GSPATT00008497001	0,172	0,39591	0
GSPATT00008503001	0,019	0,92472	0
GSPATT00008512001	0,357	0,06391	0
GSPATT00008527001	0,18	0,35417	0
GSPATT00008573001	-0,003	0,98576	0
GSPATT00008596001	-1,074	0,00006	-1
GSPATT00008602001	0,371	0,02113	0
GSPATT00008611001	0,235	0,3889	0
GSPATT00008619001	-0,021	0,87621	0
GSPATT00008639001	-0,663	0,00927	0
GSPATT00008645001	0,599	0,05259	0
GSPATT00008738001	0,821	0,00201	0
GSPATT00008749001	-0,136	0,49358	0
GSPATT00008764001	0,257	0,19576	0
GSPATT00008778001	0,342	0,04202	0
GSPATT00008805001	0,579	0,00784	0
GSPATT00008830001	0,301	0,18862	0
GSPATT00008831001	0,097	0,54219	0
GSPATT00008850001	0,887	0,00048	0
GSPATT00008866001	0,383	0,07319	0
GSPATT00008880001	-0,665	0,00165	0
GSPATT00008893001	0,303	0,07004	0
GSPATT00008897001	0,334	0,19577	0
GSPATT00008901001	-1,237	0,00007	-1
GSPATT00008924001	0,154	0,39308	0
GSPATT00008975001	0,694	0,00205	0
GSPATT00008986001	0,293	0,16667	0
GSPATT00027401001	0,365	0,07066	0
GSPATT00027404001	-0,378	0,02671	0
GSPATT00027408001	0,032	0,82286	0
GSPATT00027410001	0,523	0,0081	0
GSPATT00027442001	-0,097	0,65217	0
GSPATT00027453001	-0,38	0,0322	0
GSPATT00027465001	-1,097	0,00007	-1
GSPATT00027479001	0,392	0,10569	0
GSPATT00027483001	0,725	0,00101	0
GSPATT00027492001	1,241	0,00093	1
GSPATT00027506001	0,183	0,46769	0
GSPATT00027520001	-0,336	0,16994	0
GSPATT00027535001	0,825	0,01036	0
GSPATT00027546001	-0,383	0,03735	0
GSPATT00027549001	0,322	0,09184	0
GSPATT00027607001	0,43	0,06985	0
GSPATT00027613001	-0,299	0,09538	0
GSPATT00027617001	-0,374	0,02379	0
GSPATT00027623001	0,439	0,01037	0
GSPATT00027637001	0,397	0,02895	0
GSPATT00027641001	1,114	0,00049	1
GSPATT00027648001	-0,304	0,04309	0
GSPATT00027665001	0,667	0,04913	0
GSPATT00027672001	-0,668	0,01828	0
GSPATT00027677001	0,078	0,65055	0
GSPATT00027685001	0,812	0,00085	0
GSPATT00027690001	0,646	0,00592	0
GSPATT00027700001	0,396	0,14503	0
GSPATT00027727001	0,419	0,06587	0
GSPATT00027731001	-0,101	0,74111	0
GSPATT00027740001	0,305	0,04752	0
GSPATT00027741001	0,59	0,00929	0
GSPATT00027763001	0,328	0,03342	0
GSPATT00027787001	0,369	0,04914	0
GSPATT00027796001	-1,461	0,0001	-1
GSPATT00027807001	-2	0	-1
GSPATT00027831001	1,076	0,00008	1
GSPATT00027838001	0,488	0,03197	0
GSPATT00027852001	-1,233	0,00015	-1
GSPATT00027857001	0,522	0,0062	0
GSPATT00027870001	-0,002	0,9924	0
GSPATT00027898001	0,118	0,48905	0
GSPATT00027906001	0,065	0,67546	0
GSPATT00027913001	0,404	0,09183	0
GSPATT00027933001	-0,886	0,00125	0
GSPATT00027938001	0,112	0,47766	0
GSPATT00027978001	-0,102	0,58439	0
GSPATT00027980001	-0,117	0,44254	0
GSPATT00028006001	0,033	0,84856	0
GSPATT00028020001	1,049	0,00011	1
GSPATT00028029001	1,144	0,00006	1
GSPATT00028033001	0,014	0,94972	0
GSPATT00028035001	1,108	0,00038	1
GSPATT00028058001	-1,581	0,00253	-1
GSPATT00028068001	0,819	0,00613	0
GSPATT00028082001	0,851	0,00023	0
GSPATT00028088001	0,595	0,00642	0
GSPATT00028112001	0,016	0,92503	0
GSPATT00028130001	0,382	0,0628	0
GSPATT00028137001	-0,328	0,07792	0
GSPATT00028156001	0,053	0,70232	0
GSPATT00028162001	0,316	0,14219	0
GSPATT00028174001	-0,017	0,90512	0
GSPATT00028181001	0,307	0,0498	0
GSPATT00028199001	1,181	0,00015	1
GSPATT00028210001	-0,239	0,19183	0
GSPATT00028223001	0,071	0,6272	0

GSPATT00009001001	0,558	0,04588	0	GSPATT00028224001	-0,142	0,45435	0
GSPATT00009009001	-0,53	0,00588	0	GSPATT00028226001	-0,224	0,18937	0
GSPATT00009013001	1,033	0,00013	1	GSPATT00028254001	0,074	0,68566	0
GSPATT00009076001	0,364	0,0251	0	GSPATT00028260001	0,201	0,33982	0
GSPATT00009086001	0,586	0,00945	0	GSPATT00028261001	0,648	0,00753	0
GSPATT00009091001	-2,067	0,00001	-1	GSPATT00028264001	-0,912	0,00051	0
GSPATT00009102001	0,179	0,23283	0	GSPATT00028265001	-0,664	0,00186	0
GSPATT00009110001	0,013	0,95996	0	GSPATT00028266001	-0,643	0,01703	0
GSPATT00009123001	-0,164	0,60057	0	GSPATT00028267001	1,612	0,00001	1
GSPATT00009138001	1,521	0,00028	1	GSPATT00028269001	0,88	0,00108	0
GSPATT00009146001	-0,481	0,05125	0	GSPATT00028305001	0,264	0,18486	0
GSPATT00009149001	0,025	0,87197	0	GSPATT00028307001	-0,082	0,66338	0
GSPATT00009155001	-0,238	0,12312	0	GSPATT00028312001	0,636	0,00684	0
GSPATT00009172001	-1,485	0,00015	-1	GSPATT00028368001	0,41	0,03475	0
GSPATT00009173001	0,244	0,23213	0	GSPATT00028374001	0,68	0,00629	0
GSPATT00009174001	0,724	0,00457	0	GSPATT00028387001	-0,687	0,00975	0
GSPATT00009176001	0,333	0,1816	0	GSPATT00028405001	0,115	0,6059	0
GSPATT00009191001	0,221	0,13878	0	GSPATT00028413001	0,051	0,72041	0
GSPATT00009207001	1,429	0,00002	1	GSPATT00028458001	-1,195	0,00003	-1
GSPATT00009211001	1,961	0	1	GSPATT00028463001	0,348	0,16251	0
GSPATT00009267001	-1,116	0,00261	-1	GSPATT00028502001	0,619	0,01367	0
GSPATT00009271001	-0,931	0,00059	0	GSPATT00028515001	0,356	0,07284	0
GSPATT00009344001	-0,02	0,89901	0	GSPATT00028521001	-0,347	0,06932	0
GSPATT00009346001	0,505	0,00751	0	GSPATT00028522001	0,518	0,02311	0
GSPATT00009355001	-0,135	0,39547	0	GSPATT00028523001	-0,194	0,30675	0
GSPATT00009389001	0,729	0,00072	0	GSPATT00028553001	-1,461	0,00001	-1
GSPATT00009399001	0,067	0,85641	0	GSPATT00028591001	-0,079	0,66532	0
GSPATT00009411001	0,367	0,14277	0	GSPATT00028640001	-0,009	0,94942	0
GSPATT00009414001	1,265	0,00079	1	GSPATT00028664001	1,165	0,00037	1
GSPATT00009428001	0,129	0,62268	0	GSPATT00028690001	0,455	0,03584	0
GSPATT00009429001	-0,821	0,00035	0	GSPATT00028737001	1,259	0,00014	1
GSPATT00009449001	0,122	0,41636	0	GSPATT00028739001	0,281	0,07289	0
GSPATT00009461001	-1,129	0,00293	-1	GSPATT00028760001	-0,016	0,95354	0
GSPATT00009466001	0,566	0,00692	0	GSPATT00028761001	-0,584	0,00278	0
GSPATT00009502001	-0,497	0,00886	0	GSPATT00028762001	0,71	0,00514	0
GSPATT00009512001	0,817	0,00579	0	GSPATT00028795001	-0,564	0,00507	0
GSPATT00009524001	0,752	0,00072	0	GSPATT00028814001	0,718	0,00439	0
GSPATT00009529001	-0,262	0,0797	0	GSPATT00028854001	0,383	0,0558	0
GSPATT00009531001	0,045	0,73938	0	GSPATT00028862001	0,155	0,44362	0
GSPATT00009539001	-0,64	0,00235	0	GSPATT00028879001	-0,101	0,52631	0
GSPATT00009552001	-0,641	0,01566	0	GSPATT00028902001	0,115	0,49398	0
GSPATT00009559001	0,264	0,18251	0	GSPATT00028907001	0,31	0,07543	0
GSPATT00009580001	0,2	0,21252	0	GSPATT00028913001	0,627	0,00447	0
GSPATT00009588001	0,994	0,04353	0	GSPATT00028920001	0,862	0,00104	0
GSPATT00009601001	-0,457	0,11601	0	GSPATT00028924001	0,504	0,00498	0
GSPATT00009602001	0,93	0,0008	0	GSPATT00028949001	0,858	0,01717	0
GSPATT00009607001	0,735	0,01843	0	GSPATT00028981001	0,411	0,07642	0
GSPATT00009626001	0,037	0,77284	0	GSPATT00029004001	-0,194	0,28836	0
GSPATT00009643001	0,405	0,01362	0	GSPATT00029007001	0,785	0,00492	0
GSPATT00009645001	0,973	0,00151	0	GSPATT00029023001	0,516	0,03057	0
GSPATT00009646001	0,589	0,00229	0	GSPATT00029041001	0,57	0,00776	0
GSPATT00009693001	0,347	0,0372	0	GSPATT00029053001	-0,652	0,01479	0
GSPATT00009703001	-2,787	0	-1	GSPATT00029067001	-0,083	0,53645	0
GSPATT00009718001	-0,819	0,00093	0	GSPATT00029101001	-0,869	0,00393	0
GSPATT00009748001	0,253	0,1151	0	GSPATT00029164001	0,564	0,12006	0
GSPATT00009760001	-2,631	0	-1	GSPATT00029173001	0,138	0,34159	0
GSPATT00009772001	-0,026	0,84037	0	GSPATT00029175001	-0,791	0,00029	0
GSPATT00009782001	-0,119	0,55451	0	GSPATT00029187001	0,682	0,01155	0
GSPATT00009828001	0,419	0,03475	0	GSPATT00029196001	2,188	0,00004	1
GSPATT00009848001	-0,228	0,27077	0	GSPATT00029198001	0,416	0,04007	0
GSPATT00009873001	1,405	0,00002	1	GSPATT00029217001	0,222	0,14787	0
GSPATT00009883001	0,58	0,00174	0	GSPATT00029249001	-0,351	0,02712	0
GSPATT00009888001	0,679	0,00449	0	GSPATT00029273001	-2,046	0,00011	-1
GSPATT00009892001	-0,164	0,50097	0	GSPATT00029295001	1,454	0,00404	1
GSPATT00009932001	-0,127	0,37952	0	GSPATT00029341001	0,262	0,14262	0
GSPATT00009937001	0,505	0,12425	0	GSPATT00029366001	0,262	0,1057	0
GSPATT00009951001	-0,279	0,21687	0	GSPATT00029406001	0,843	0,00341	0

GSPATT00009964001	0,607	0,03286	0	GSPATT00029408001	0,85	0,0034	0
GSPATT00009969001	0,018	0,89309	0	GSPATT00029420001	1,305	0,00003	1
GSPATT00009980001	0,361	0,01933	0	GSPATT00029433001	0,663	0,01729	0
GSPATT00009997001	0,66	0,0026	0	GSPATT00029443001	-0,601	0,00289	0
GSPATT00010022001	-0,69	0,00171	0	GSPATT00029444001	1,26	0,00076	1
GSPATT00010023001	0,56	0,00691	0	GSPATT00029470001	0,093	0,49861	0
GSPATT00010038001	0,125	0,38308	0	GSPATT00029472001	0,184	0,20615	0
GSPATT00010040001	-0,369	0,07302	0	GSPATT00029484001	0,665	0,00137	0
GSPATT00010043001	0,211	0,26138	0	GSPATT00029530001	0,081	0,70204	0
GSPATT00010044001	0,443	0,07115	0	GSPATT00029552001	0,56	0,01662	0
GSPATT00010047001	0,376	0,04978	0	GSPATT00029556001	-0,933	0,00515	0
GSPATT00010069001	0,096	0,49349	0	GSPATT00029558001	1,707	0,00001	1
GSPATT00010072001	0,597	0,04414	0	GSPATT00029574001	-1,108	0,00039	-1
GSPATT00010088001	0,474	0,01739	0	GSPATT00029583001	0,198	0,35094	0
GSPATT00010089001	-0,02	0,89788	0	GSPATT00029601001	1,502	0,00004	1
GSPATT00010120001	0,439	0,05835	0	GSPATT00029614001	-0,616	0,00563	0
GSPATT00010165001	1,057	0,05472	0	GSPATT00029618001	0,584	0,00422	0
GSPATT00010186001	-0,625	0,00879	0	GSPATT00029627001	-0,199	0,15139	0
GSPATT00010187001	0,077	0,70848	0	GSPATT00029631001	0,75	0,00323	0
GSPATT00010189001	0,274	0,09078	0	GSPATT00029713001	0,817	0,00169	0
GSPATT00010192001	-0,341	0,04434	0	GSPATT00029723001	0,372	0,11982	0
GSPATT00010201001	-1,112	0,00403	-1	GSPATT00029731001	0,613	0,0095	0
GSPATT00010217001	0,055	0,68694	0	GSPATT00029750001	0,907	0,00106	0
GSPATT00010238001	0,721	0,00063	0	GSPATT00029761001	0,264	0,06412	0
GSPATT00010240001	1,641	0,00002	1	GSPATT00029771001	-0,737	0,0067	0
GSPATT00010241001	-0,059	0,67929	0	GSPATT00029778001	-0,608	0,00307	0
GSPATT00010256001	0,667	0,16041	0	GSPATT00029822001	0,665	0,00641	0
GSPATT00010264001	1,126	0,00022	1	GSPATT00029823001	-0,53	0,02834	0
GSPATT00010267001	0,363	0,04122	0	GSPATT00029824001	0,784	0,05706	0
GSPATT00010305001	0,583	0,02695	0	GSPATT00029844001	0,11	0,52769	0
GSPATT00010342001	-0,027	0,88113	0	GSPATT00029848001	0,411	0,08951	0
GSPATT00010360001	0,009	0,9481	0	GSPATT00029901001	0,748	0,00121	0
GSPATT00010361001	1,344	0,00002	1	GSPATT00029929001	1,561	0,00012	1
GSPATT00010434001	0,716	0,00843	0	GSPATT00029968001	0,19	0,2579	0
GSPATT00010455001	2,068	0,00001	1	GSPATT00029971001	-0,01	0,95787	0
GSPATT00010478001	0,755	0,0015	0	GSPATT00030006001	0,333	0,17202	0
GSPATT00010489001	0,856	0,01707	0	GSPATT00030011001	0,244	0,31877	0
GSPATT00010520001	1,675	0,00001	1	GSPATT00030052001	-0,257	0,11428	0
GSPATT00010535001	0,291	0,15153	0	GSPATT00030056001	0,096	0,60981	0
GSPATT00010550001	-0,166	0,3917	0	GSPATT00030064001	1,558	0,00015	1
GSPATT00010557001	0,305	0,0402	0	GSPATT00030135001	0,579	0,06809	0
GSPATT00010591001	-0,327	0,06596	0	GSPATT00030142001	0,973	0,0012	0
GSPATT00010597001	0,042	0,75702	0	GSPATT00030146001	-0,644	0,00333	0
GSPATT00010599001	-0,541	0,00596	0	GSPATT00030175001	-0,442	0,01694	0
GSPATT00010606001	-0,148	0,37763	0	GSPATT00030183001	0,09	0,72161	0
GSPATT00010615001	0,827	0,00035	0	GSPATT00030220001	-0,307	0,04731	0
GSPATT00010622001	0,288	0,09844	0	GSPATT00030230001	0,126	0,43629	0
GSPATT00010631001	-0,002	0,99061	0	GSPATT00030249001	1,907	0	1
GSPATT00010634001	-0,099	0,64063	0	GSPATT00030250001	0,03	0,86055	0
GSPATT00010700001	1,693	0	1	GSPATT00030251001	0,536	0,02526	0
GSPATT00010735001	0,406	0,06251	0	GSPATT00030268001	0,167	0,24397	0
GSPATT00010751001	0,385	0,03146	0	GSPATT00030295001	0,775	0,00158	0
GSPATT00010794001	0,388	0,14585	0	GSPATT00030300001	0,307	0,11242	0
GSPATT00010803001	0,375	0,05126	0	GSPATT00030312001	0,159	0,34993	0
GSPATT00010807001	0,842	0,00505	0	GSPATT00030338001	0,37	0,02763	0
GSPATT00010846001	0,562	0,04885	0	GSPATT00030339001	0,929	0,00014	0
GSPATT00010852001	0,595	0,00207	0	GSPATT00030340001	-0,629	0,00193	0
GSPATT00010926001	-0,295	0,08518	0	GSPATT00030347001	0,305	0,05631	0
GSPATT00010927001	0,963	0,00093	0	GSPATT00030360001	1,11	0,00034	1
GSPATT00010930001	0,326	0,08283	0	GSPATT00030377001	0,608	0,03137	0
GSPATT00010931001	0,755	0,00409	0	GSPATT00030395001	0,632	0,00363	0
GSPATT00010951001	-0,4	0,08547	0	GSPATT00030433001	0,649	0,01931	0
GSPATT00010982001	1,537	0,00037	1	GSPATT00030441001	-1,166	0,00076	-1
GSPATT00010994001	-2,479	0	-1	GSPATT00030461001	0,787	0,01769	0
GSPATT00011004001	0,073	0,58776	0	GSPATT00030462001	2,069	0	1
GSPATT00011094001	-0,248	0,11654	0	GSPATT00030463001	0,18	0,23178	0
GSPATT00011096001	0,27	0,14138	0	GSPATT00030525001	0,296	0,14492	0

GSPATT00011110001	0,439	0,02922	0	GSPATT00030559001	1,24	0,00015	1
GSPATT00011112001	0,611	0,00704	0	GSPATT00030602001	0,266	0,22578	0
GSPATT00011130001	0,933	0,00615	0	GSPATT00030614001	-0,182	0,32497	0
GSPATT00011132001	-1,165	0,00035	-1	GSPATT00030636001	0,66	0,0019	0
GSPATT00011167001	-0,396	0,02351	0	GSPATT00030654001	-0,73	0,00085	0
GSPATT00011173001	-3,857	0	-1	GSPATT00030663001	0,245	0,35912	0
GSPATT00011179001	0,334	0,11641	0	GSPATT00030666001	0,411	0,06139	0
GSPATT00011220001	0,315	0,15075	0	GSPATT00030669001	0,057	0,6915	0
GSPATT00011221001	1,038	0,00031	1	GSPATT00030671001	-0,295	0,30814	0
GSPATT00011227001	0,852	0,00196	0	GSPATT00030687001	0,812	0,01438	0
GSPATT00011241001	0,626	0,02469	0	GSPATT00030688001	-0,02	0,90163	0
GSPATT00011251001	0,282	0,09853	0	GSPATT00030698001	0,071	0,70437	0
GSPATT00011290001	1,791	0	1	GSPATT00030723001	-0,18	0,43523	0
GSPATT00011305001	-0,492	0,0392	0	GSPATT00030733001	-1,617	0,00013	-1
GSPATT00011340001	0,056	0,74423	0	GSPATT00030745001	-0,1	0,48372	0
GSPATT00011369001	1,426	0,00002	1	GSPATT00030749001	-0,359	0,06814	0
GSPATT00011371001	-0,116	0,58125	0	GSPATT00030782001	0,235	0,17113	0
GSPATT00011372001	-0,323	0,11988	0	GSPATT00030803001	1,123	0,0001	1
GSPATT00011413001	0,314	0,1297	0	GSPATT00030806001	0,727	0,0015	0
GSPATT00011422001	-1,076	0,00614	-1	GSPATT00030818001	-0,245	0,23455	0
GSPATT00011430001	0,323	0,07271	0	GSPATT00030848001	0,031	0,86428	0
GSPATT00011436001	0,608	0,01574	0	GSPATT00030851001	0,263	0,1287	0
GSPATT00011440001	0,123	0,57717	0	GSPATT00030853001	0,549	0,03624	0
GSPATT00011454001	0,507	0,07282	0	GSPATT00030855001	1,1	0,00013	1
GSPATT00011455001	-0,483	0,15659	0	GSPATT00030860001	1,314	0,00014	1
GSPATT00011486001	-0,104	0,60315	0	GSPATT00030871001	-0,434	0,01131	0
GSPATT00011499001	-0,148	0,29042	0	GSPATT00030877001	-0,793	0,0011	0
GSPATT00011546001	-0,929	0,00313	0	GSPATT00030894001	0,443	0,01543	0
GSPATT00011547001	0,964	0,00026	0	GSPATT00030895001	0,613	0,00519	0
GSPATT00011564001	0,676	0,00108	0	GSPATT00030907001	0,89	0,00402	0
GSPATT00011566001	0,262	0,07738	0	GSPATT00030912001	1,631	0,00007	1
GSPATT00011580001	-0,02	0,90743	0	GSPATT00030921001	0,509	0,01961	0
GSPATT00011584001	0,283	0,12359	0	GSPATT00030945001	0,658	0,01733	0
GSPATT00011622001	0,355	0,17874	0	GSPATT00030952001	-0,016	0,91391	0
GSPATT00011666001	-0,488	0,00499	0	GSPATT00030956001	-0,545	0,00308	0
GSPATT00011685001	-0,399	0,05343	0	GSPATT00030971001	0,707	0,00637	0
GSPATT00011700001	-0,131	0,51052	0	GSPATT00030979001	0,011	0,94131	0
GSPATT00011714001	0,124	0,49457	0	GSPATT00030994001	0,373	0,06118	0
GSPATT00011716001	0,632	0,00185	0	GSPATT00031006001	1,218	0,00004	1
GSPATT00011729001	-0,066	0,66441	0	GSPATT00031028001	-0,522	0,00869	0
GSPATT00011731001	0,353	0,05227	0	GSPATT00031029001	-0,147	0,30646	0
GSPATT00011735001	1,538	0,00053	1	GSPATT00031066001	-3,25	0	-1
GSPATT00011739001	-0,466	0,04961	0	GSPATT00031082001	0,807	0,00177	0
GSPATT00011742001	1,365	0,00005	1	GSPATT00031097001	-0,034	0,81174	0
GSPATT00011764001	0,296	0,13873	0	GSPATT00031104001	-1,662	0,00021	-1
GSPATT00011816001	-0,319	0,13633	0	GSPATT00031107001	0,915	0,00188	0
GSPATT00011825001	-0,485	0,08169	0	GSPATT00031110001	1,016	0,0098	1
GSPATT00011826001	1,565	0,00133	1	GSPATT00031155001	-0,356	0,05023	0
GSPATT00011840001	-0,798	0,00327	0	GSPATT00031161001	2,492	0	1
GSPATT00011854001	0,553	0,02078	0	GSPATT00031166001	0,279	0,06965	0
GSPATT00011856001	0,65	0,07665	0	GSPATT00031167001	0,272	0,08891	0
GSPATT00011864001	-0,091	0,49677	0	GSPATT00031168001	0,836	0,01523	0
GSPATT00011887001	0,175	0,46958	0	GSPATT00031178001	0,551	0,0218	0
GSPATT00011897001	0,61	0,00299	0	GSPATT00031182001	-0,535	0,01622	0
GSPATT00011903001	0,087	0,58179	0	GSPATT00031235001	0,643	0,00762	0
GSPATT00011917001	0,417	0,15463	0	GSPATT00031285001	-0,236	0,21081	0
GSPATT00011929001	-1,564	0,00004	-1	GSPATT00031287001	0,155	0,35654	0
GSPATT00011952001	0,118	0,38041	0	GSPATT00031336001	0,795	0,00047	0
GSPATT00011968001	0,326	0,1663	0	GSPATT00031426001	-1,033	0,00006	-1
GSPATT00011981001	-0,032	0,83233	0	GSPATT00031441001	-0,048	0,82385	0
GSPATT00011982001	0,265	0,10595	0	GSPATT00031487001	0,99	0,002	0
GSPATT00012043001	0,391	0,11161	0	GSPATT00031493001	0,611	0,02344	0
GSPATT00012044001	-0,04	0,80131	0	GSPATT00031509001	-0,798	0,00098	0
GSPATT00012047001	-0,114	0,39986	0	GSPATT00031532001	1,665	0,00021	1
GSPATT00012141001	0,915	0,00115	0	GSPATT00031569001	-0,125	0,54879	0
GSPATT00012162001	0,3	0,06106	0	GSPATT00031612001	2,01	0,00001	1
GSPATT00012163001	-0,072	0,57986	0	GSPATT00031618001	0,323	0,2332	0

GSPATT00012176001	0,545	0,02424	0	GSPATT00031623001	-0,002	0,9946	0
GSPATT00012210001	-0,372	0,02535	0	GSPATT00031630001	1,142	0,00103	1
GSPATT00012216001	1,175	0,00014	1	GSPATT00031642001	-1,737	0,00014	-1
GSPATT00012223001	0,404	0,08406	0	GSPATT00031645001	0,862	0,00026	0
GSPATT00012241001	0,159	0,5354	0	GSPATT00031653001	-0,225	0,14582	0
GSPATT00012255001	0,104	0,64586	0	GSPATT00031661001	0,548	0,02007	0
GSPATT00012262001	-1,15	0,00006	-1	GSPATT00031665001	-0,001	0,99648	0
GSPATT00012269001	-0,185	0,39154	0	GSPATT00031698001	-0,689	0,03351	0
GSPATT00012278001	1,263	0,00039	1	GSPATT00031701001	-0,914	0,00444	0
GSPATT00012282001	0,837	0,01461	0	GSPATT00031705001	1,81	0,00002	1
GSPATT00012286001	-0,79	0,00041	0	GSPATT00031706001	-0,775	0,0011	0
GSPATT00012322001	1,362	0,00005	1	GSPATT00031738001	-1,034	0,00008	-1
GSPATT00012339001	1,278	0,00002	1	GSPATT00031739001	0,382	0,19043	0
GSPATT00012348001	-1,134	0,00014	-1	GSPATT00031740001	0,355	0,05225	0
GSPATT00012351001	0,299	0,06088	0	GSPATT00031754001	-0,461	0,03297	0
GSPATT00012371001	-0,352	0,29514	0	GSPATT00031756001	-0,463	0,03455	0
GSPATT00012372001	0,15	0,484	0	GSPATT00031757001	0,716	0,03439	0
GSPATT00012374001	0,203	0,24495	0	GSPATT00031762001	-1,768	0,00003	-1
GSPATT00012376001	0,959	0,00045	0	GSPATT00031768001	-0,037	0,82459	0
GSPATT00012379001	0,369	0,07668	0	GSPATT00031781001	-0,507	0,02156	0
GSPATT00012395001	0,809	0,0075	0	GSPATT00031782001	-0,24	0,42749	0
GSPATT00012396001	0,319	0,11515	0	GSPATT00031802001	0,907	0,00053	0
GSPATT00012400001	0,852	0,00032	0	GSPATT00031822001	0,463	0,05116	0
GSPATT00012442001	0,124	0,39221	0	GSPATT00031828001	-0,487	0,01743	0
GSPATT00012445001	0,429	0,03783	0	GSPATT00031846001	-0,183	0,21789	0
GSPATT00012460001	0,238	0,14276	0	GSPATT00031865001	0,272	0,16899	0
GSPATT00012470001	-0,034	0,86348	0	GSPATT00031924001	0,545	0,00883	0
GSPATT00012488001	-0,04	0,75807	0	GSPATT00031948001	-0,37	0,18789	0
GSPATT00012520001	-2,03	0	-1	GSPATT00031955001	-0,129	0,4888	0
GSPATT00012525001	0,13	0,44838	0	GSPATT00031957001	0,03	0,87201	0
GSPATT00012543001	0,86	0,00059	0	GSPATT00032021001	0,096	0,48485	0
GSPATT00012570001	0,319	0,06017	0	GSPATT00032022001	-0,54	0,01533	0
GSPATT00012593001	-0,057	0,67906	0	GSPATT00032029001	0,283	0,22374	0
GSPATT00012610001	0,358	0,06157	0	GSPATT00032030001	1,339	0,00008	1
GSPATT00012628001	0,326	0,03065	0	GSPATT00032037001	0,772	0,00865	0
GSPATT00012631001	0,442	0,02953	0	GSPATT00032092001	0,508	0,09947	0
GSPATT00012633001	0,282	0,10356	0	GSPATT00032094001	-2,851	0	-1
GSPATT00012634001	0,634	0,00459	0	GSPATT00032095001	0,112	0,41059	0
GSPATT00012649001	-0,517	0,01272	0	GSPATT00032099001	0,878	0,00102	0
GSPATT00012656001	0,231	0,16634	0	GSPATT00032100001	0,509	0,00574	0
GSPATT00012666001	0,427	0,02248	0	GSPATT00032104001	-0,024	0,9428	0
GSPATT00012678001	-0,029	0,84403	0	GSPATT00032110001	-1,089	0,00427	-1
GSPATT00012684001	-0,036	0,83675	0	GSPATT00032111001	-1,233	0,00541	-1
GSPATT00012692001	0,375	0,08064	0	GSPATT00032114001	0,44	0,01977	0
GSPATT00012702001	0,193	0,34721	0	GSPATT00032136001	-0,671	0,00209	0
GSPATT00012722001	0,574	0,01609	0	GSPATT00032166001	0,482	0,04639	0
GSPATT00012732001	0,363	0,12773	0	GSPATT00032169001	0,497	0,00936	0
GSPATT00012735001	0,149	0,25751	0	GSPATT00032224001	0,067	0,66182	0
GSPATT00012742001	1,227	0,00011	1	GSPATT00032226001	0,466	0,04696	0
GSPATT00012746001	0,143	0,51196	0	GSPATT00032238001	0,364	0,06778	0
GSPATT00012753001	0,157	0,26935	0	GSPATT00032288001	0,859	0,00547	0
GSPATT00012754001	-0,119	0,5919	0	GSPATT00032289001	-0,097	0,49171	0
GSPATT00012755001	1,516	0,00005	1	GSPATT00032301001	-0,947	0,00029	0
GSPATT00012756001	0,835	0,00358	0	GSPATT00032341001	0,829	0,00058	0
GSPATT00012822001	0,123	0,4374	0	GSPATT00032372001	-0,241	0,09642	0
GSPATT00012835001	0,596	0,00563	0	GSPATT00032392001	0,313	0,05107	0
GSPATT00012838001	0,001	0,99699	0	GSPATT00032421001	-1,221	0,00022	-1
GSPATT00012852001	1,275	0,00003	1	GSPATT00032429001	0,493	0,03216	0
GSPATT00012853001	0,823	0,00197	0	GSPATT00032432001	-0,292	0,20952	0
GSPATT00012859001	-0,198	0,15153	0	GSPATT00032442001	0,198	0,2398	0
GSPATT00012868001	0,163	0,31281	0	GSPATT00032444001	-0,967	0,00044	0
GSPATT00012875001	0,348	0,03129	0	GSPATT00032458001	0,703	0,02409	0
GSPATT00012913001	0,495	0,01652	0	GSPATT00032459001	0,275	0,11268	0
GSPATT00012914001	0,157	0,26322	0	GSPATT00032460001	-0,526	0,00918	0
GSPATT00012916001	-2,396	0	-1	GSPATT00032522001	-0,127	0,45119	0
GSPATT00012931001	0,812	0,00089	0	GSPATT00032523001	0,812	0,00089	0
GSPATT00012973001	-0,403	0,04733	0	GSPATT00032532001	0,103	0,45294	0

GSPATT00012998001	-0,105	0,43684	0	GSPATT00032533001	-0,17	0,53723	0
GSPATT00013005001	-0,158	0,4174	0	GSPATT00032559001	0,124	0,37615	0
GSPATT00013045001	0,257	0,10688	0	GSPATT00032564001	1,204	0,00066	1
GSPATT00013048001	0,033	0,83799	0	GSPATT00032574001	1,413	0,00228	1
GSPATT00013062001	-0,119	0,40756	0	GSPATT00032597001	0,884	0,00108	0
GSPATT00013085001	1,595	0,00002	1	GSPATT00032618001	0,457	0,06613	0
GSPATT00013094001	0,81	0,00674	0	GSPATT00032619001	0,741	0,02826	0
GSPATT00013102001	0,769	0,00426	0	GSPATT00032623001	-0,162	0,37167	0
GSPATT00013110001	-1,076	0,00259	-1	GSPATT00032667001	1,812	0,00041	1
GSPATT00013157001	0,235	0,17548	0	GSPATT00032670001	0,786	0,00243	0
GSPATT00013159001	0,37	0,09003	0	GSPATT00032697001	0,477	0,0138	0
GSPATT00013164001	-0,51	0,05354	0	GSPATT00032706001	0,443	0,01419	0
GSPATT00013165001	1,021	0,00106	1	GSPATT00032739001	0,096	0,63648	0
GSPATT00013173001	-0,295	0,08555	0	GSPATT00032755001	-0,448	0,01909	0
GSPATT00013174001	0,011	0,93101	0	GSPATT00032763001	-0,489	0,02531	0
GSPATT00013205001	0,52	0,00824	0	GSPATT00032783001	-0,567	0,00797	0
GSPATT00013243001	-0,027	0,84517	0	GSPATT00032791001	0,581	0,0063	0
GSPATT00013253001	1,885	0,00001	1	GSPATT00032810001	0,795	0,00874	0
GSPATT00013260001	0,645	0,00484	0	GSPATT00032894001	0,722	0,00361	0
GSPATT00013263001	0,563	0,01505	0	GSPATT00032910001	1,39	0,00003	1
GSPATT00013271001	0,175	0,21491	0	GSPATT00032920001	0,74	0,00144	0
GSPATT00013272001	1,673	0,00001	1	GSPATT00032929001	0,137	0,64968	0
GSPATT00013281001	0,128	0,50204	0	GSPATT00032930001	1,256	0,00033	1
GSPATT00013308001	0,015	0,90603	0	GSPATT00032934001	0,256	0,2624	0
GSPATT00013315001	-0,188	0,20496	0	GSPATT00032999001	0,68	0,00704	0
GSPATT00013327001	-0,009	0,94794	0	GSPATT00033009001	-0,785	0,00143	0
GSPATT00013343001	0,267	0,15492	0	GSPATT00033016001	0,224	0,20102	0
GSPATT00013356001	-0,722	0,00162	0	GSPATT00033027001	0,378	0,03882	0
GSPATT00013360001	-0,83	0,00139	0	GSPATT00033067001	0,968	0,00046	0
GSPATT00013364001	-0,11	0,50767	0	GSPATT00033072001	0,525	0,02731	0
GSPATT00013383001	-1,314	0,0001	-1	GSPATT00033076001	0,219	0,41915	0
GSPATT00013453001	0,604	0,02056	0	GSPATT00033095001	-1,102	0,00064	-1
GSPATT00013470001	0,177	0,28718	0	GSPATT00033106001	0,765	0,00138	0
GSPATT00013490001	0,322	0,07055	0	GSPATT00033119001	-0,51	0,03205	0
GSPATT00013494001	0,052	0,75665	0	GSPATT00033139001	0,432	0,01158	0
GSPATT00013502001	-0,243	0,19365	0	GSPATT00033154001	1,026	0,00696	1
GSPATT00013552001	0,741	0,00042	0	GSPATT00033161001	-0,302	0,08205	0
GSPATT00013561001	0,271	0,16205	0	GSPATT00033165001	-0,046	0,8569	0
GSPATT00013578001	1,991	0,00001	1	GSPATT00033167001	-0,34	0,05638	0
GSPATT00013652001	0,191	0,18598	0	GSPATT00033198001	0,629	0,09101	0
GSPATT00013656001	0,171	0,2115	0	GSPATT00033205001	0,673	0,00702	0
GSPATT00013680001	-0,238	0,41631	0	GSPATT00033223001	-0,423	0,03978	0
GSPATT00013693001	0,493	0,01038	0	GSPATT00033237001	-0,283	0,16249	0
GSPATT00013695001	0,474	0,01357	0	GSPATT00033250001	-0,453	0,0188	0
GSPATT00013702001	-0,192	0,32187	0	GSPATT00033258001	0,335	0,03868	0
GSPATT00013754001	-3,054	0	-1	GSPATT00033260001	-0,16	0,55088	0
GSPATT00013770001	-0,187	0,4332	0	GSPATT00033306001	-0,043	0,85073	0
GSPATT00013783001	1,026	0,01651	1	GSPATT00033333001	0,576	0,00241	0
GSPATT00013790001	0,074	0,5863	0	GSPATT00033334001	0,4	0,0313	0
GSPATT00013801001	0,361	0,0755	0	GSPATT00033358001	-0,167	0,26712	0
GSPATT00013825001	-0,769	0,00205	0	GSPATT00033399001	-0,126	0,62052	0
GSPATT00013827001	0,481	0,00732	0	GSPATT00033466001	-1,107	0,00106	-1
GSPATT00013828001	0,419	0,03295	0	GSPATT00033469001	0,884	0,00069	0
GSPATT00013847001	0,139	0,40055	0	GSPATT00033477001	0,439	0,04845	0
GSPATT00013852001	-0,368	0,03397	0	GSPATT00033494001	0,481	0,13408	0
GSPATT00013855001	0,438	0,04631	0	GSPATT00033495001	1,143	0,00006	1
GSPATT00013876001	1,319	0,00047	1	GSPATT00033505001	0,875	0,00023	0
GSPATT00013891001	0,176	0,26584	0	GSPATT00033512001	-3,055	0	-1
GSPATT00013892001	-1,136	0,00035	-1	GSPATT00033523001	-1,294	0,00005	-1
GSPATT00013898001	0,162	0,43243	0	GSPATT00033534001	0,523	0,02677	0
GSPATT00013900001	-0,003	0,98838	0	GSPATT00033537001	0,391	0,03807	0
GSPATT00013901001	0,247	0,35648	0	GSPATT00033539001	0,246	0,40993	0
GSPATT00013913001	1,633	0,00001	1	GSPATT00033568001	-0,204	0,16501	0
GSPATT00013928001	0,8	0,00105	0	GSPATT00033573001	0,114	0,43633	0
GSPATT00013961001	2,283	0,00001	1	GSPATT00033586001	0,277	0,16433	0
GSPATT00013977001	0,458	0,01806	0	GSPATT00033606001	-0,328	0,25723	0
GSPATT00013994001	1,578	0,00006	1	GSPATT00033617001	0,273	0,25396	0

GSPATT00014001001	0,554	0,00382	0	GSPATT00033636001	0,336	0,04963	0
GSPATT00014008001	0,723	0,00107	0	GSPATT00033646001	-0,077	0,64536	0
GSPATT00014044001	0,335	0,03981	0	GSPATT00033676001	0,609	0,03214	0
GSPATT00014072001	0,024	0,90147	0	GSPATT00033682001	-0,181	0,30005	0
GSPATT00014088001	0,517	0,03193	0	GSPATT00033727001	-0,264	0,20849	0
GSPATT00014093001	0,192	0,19217	0	GSPATT00033743001	0,187	0,34652	0
GSPATT00014104001	0,566	0,03625	0	GSPATT00033764001	1,303	0,00004	1
GSPATT00014124001	-1,671	0,00007	-1	GSPATT00033786001	0,824	0,01346	0
GSPATT00014132001	2,015	0	1	GSPATT00033798001	0,882	0,00143	0
GSPATT00014139001	0,906	0,02173	0	GSPATT00033801001	0,274	0,11977	0
GSPATT00014152001	0,095	0,60893	0	GSPATT00033810001	-0,178	0,21774	0
GSPATT00014158001	1,1	0,00099	1	GSPATT00033826001	0,54	0,01676	0
GSPATT00014196001	-0,265	0,26029	0	GSPATT00033832001	1,004	0,0005	1
GSPATT00014197001	0,509	0,02553	0	GSPATT00033839001	0,232	0,20924	0
GSPATT00014202001	0,571	0,00343	0	GSPATT00033861001	0,201	0,23013	0
GSPATT00014213001	-0,445	0,00944	0	GSPATT00033863001	0,989	0,00013	0
GSPATT00014216001	0,08	0,66083	0	GSPATT00033871001	0,751	0,00036	0
GSPATT00014218001	0,813	0,0031	0	GSPATT00033879001	0,892	0,00128	0
GSPATT00014219001	0,639	0,00371	0	GSPATT00033881001	0,29	0,19304	0
GSPATT00014235001	0,358	0,02401	0	GSPATT00033904001	0,322	0,04932	0
GSPATT00014239001	0,559	0,04551	0	GSPATT00033912001	0,771	0,0063	0
GSPATT00014253001	-0,142	0,35399	0	GSPATT00033917001	0,213	0,23333	0
GSPATT00014299001	0,536	0,03039	0	GSPATT00033938001	1,074	0,00021	1
GSPATT00014310001	0,41	0,03511	0	GSPATT00033979001	-0,385	0,04696	0
GSPATT00014335001	-0,025	0,84538	0	GSPATT00033980001	0,087	0,60132	0
GSPATT00014350001	-0,099	0,44714	0	GSPATT00034005001	1,03	0,00044	1
GSPATT00014391001	0,443	0,007	0	GSPATT00034018001	-1,92	0	-1
GSPATT00014411001	-0,004	0,97822	0	GSPATT00034024001	1,786	0,00003	1
GSPATT00014414001	0,567	0,00623	0	GSPATT00034063001	-0,183	0,27172	0
GSPATT00014433001	-0,31	0,10884	0	GSPATT00034071001	0,388	0,02595	0
GSPATT00014461001	0,95	0,00019	0	GSPATT00034075001	-0,137	0,30288	0
GSPATT00014474001	-1,949	0	-1	GSPATT00034090001	0,737	0,00209	0
GSPATT00014478001	0,152	0,53003	0	GSPATT00034103001	-0,258	0,10215	0
GSPATT00014523001	1,029	0,0076	1	GSPATT00034104001	0,705	0,00489	0
GSPATT00014525001	1,28	0,0002	1	GSPATT00034124001	-1,017	0,0022	-1
GSPATT00014529001	1,182	0,00018	1	GSPATT00034141001	0,708	0,00161	0
GSPATT00014568001	-0,37	0,05496	0	GSPATT00034156001	-0,057	0,72295	0
GSPATT00014606001	0,487	0,04116	0	GSPATT00034171001	0,663	0,00966	0
GSPATT00014629001	0,582	0,00301	0	GSPATT00034175001	-0,329	0,08396	0
GSPATT00014645001	0,006	0,97144	0	GSPATT00034216001	0,528	0,01503	0
GSPATT00014660001	0,098	0,64509	0	GSPATT00034230001	0,16	0,26608	0
GSPATT00014673001	-0,235	0,2283	0	GSPATT00034233001	0,369	0,08874	0
GSPATT00014674001	0,791	0,00595	0	GSPATT00034270001	1,032	0,00088	1
GSPATT00014676001	0,429	0,07671	0	GSPATT00034308001	-0,353	0,08333	0
GSPATT00014685001	0,887	0,0008	0	GSPATT00034314001	-1,355	0,00021	-1
GSPATT00014692001	-0,278	0,14785	0	GSPATT00034329001	1,249	0,00003	1
GSPATT00014705001	0,351	0,08889	0	GSPATT00034393001	-1,986	0,00002	-1
GSPATT00014745001	0,289	0,08436	0	GSPATT00034405001	-0,584	0,00941	0
GSPATT00014759001	1,203	0,00129	1	GSPATT00034420001	-0,659	0,00959	0
GSPATT00014766001	0,379	0,03636	0	GSPATT00034421001	0,663	0,12721	0
GSPATT00014793001	-0,201	0,47536	0	GSPATT00034463001	0,197	0,40106	0
GSPATT00014796001	1,387	0,00001	1	GSPATT00034473001	0,036	0,77796	0
GSPATT00014810001	0,609	0,01875	0	GSPATT00034552001	1,257	0,00015	1
GSPATT00014841001	0,356	0,10216	0	GSPATT00034577001	-0,839	0,00058	0
GSPATT00014872001	0,244	0,12625	0	GSPATT00034596001	0,345	0,26159	0
GSPATT00014873001	0,194	0,50101	0	GSPATT00034603001	0,885	0,0034	0
GSPATT00014895001	0,711	0,00204	0	GSPATT00034615001	0,724	0,00057	0
GSPATT00014900001	0,37	0,05706	0	GSPATT00034623001	0,426	0,02613	0
GSPATT00014918001	-0,327	0,04948	0	GSPATT00034631001	0,527	0,0108	0
GSPATT00014958001	-0,462	0,20687	0	GSPATT00034632001	-0,156	0,30353	0
GSPATT00014962001	-0,017	0,92935	0	GSPATT00034637001	0,89	0,00286	0
GSPATT00014966001	-0,912	0,0003	0	GSPATT00034640001	1,246	0,00004	1
GSPATT00014999001	0,415	0,07919	0	GSPATT00034647001	0,189	0,18304	0
GSPATT00015015001	0,687	0,00414	0	GSPATT00034648001	0,251	0,12628	0
GSPATT00015017001	-1,086	0,00027	-1	GSPATT00034657001	0,421	0,01754	0
GSPATT00015018001	-0,08	0,70257	0	GSPATT00034667001	-0,213	0,27669	0
GSPATT00015021001	-1,722	0,00006	-1	GSPATT00034770001	0,27	0,13463	0

GSPATT00015033001	-0,23	0,21296	0	GSPATT00034806001	-0,248	0,17157	0
GSPATT00015042001	-0,895	0,00041	0	GSPATT00034819001	0,638	0,01374	0
GSPATT00015043001	0,268	0,46979	0	GSPATT00034862001	0,911	0,00088	0
GSPATT00015055001	0,271	0,36469	0	GSPATT00034863001	-2,801	0	-1
GSPATT00015064001	0,376	0,08595	0	GSPATT00034885001	0,254	0,14736	0
GSPATT00015109001	-0,005	0,9692	0	GSPATT00034888001	0,387	0,09919	0
GSPATT00015151001	0,296	0,07883	0	GSPATT00034893001	0,489	0,0257	0
GSPATT00015163001	-0,077	0,79332	0	GSPATT00034900001	0,251	0,34442	0
GSPATT00015169001	0,432	0,17161	0	GSPATT00034902001	-0,054	0,71793	0
GSPATT00015210001	1,87	0,00003	1	GSPATT00034911001	0,966	0,00178	0
GSPATT00015238001	1,322	0,00004	1	GSPATT00034927001	0,954	0,00128	0
GSPATT00015246001	0,577	0,07312	0	GSPATT00034954001	-0,254	0,17321	0
GSPATT00015257001	-0,315	0,32799	0	GSPATT00034968001	0,919	0,00041	0
GSPATT00015261001	0,509	0,06899	0	GSPATT00034975001	-0,694	0,00453	0
GSPATT00015299001	0,213	0,19618	0	GSPATT00034994001	0,228	0,11464	0
GSPATT00015301001	0,024	0,90708	0	GSPATT00034997001	0,566	0,00681	0
GSPATT00015311001	0,153	0,48604	0	GSPATT00035001001	0,72	0,00113	0
GSPATT00015312001	-0,675	0,00141	0	GSPATT00035014001	0,215	0,22573	0
GSPATT00015325001	0,179	0,42392	0	GSPATT00035015001	-0,804	0,00518	0
GSPATT00015335001	0,857	0,00098	0	GSPATT00035041001	0,7	0,01007	0
GSPATT00015344001	0,355	0,08801	0	GSPATT00035046001	-0,126	0,63021	0
GSPATT00015351001	0,593	0,00674	0	GSPATT00035048001	-0,034	0,8154	0
GSPATT00015356001	-0,33	0,11053	0	GSPATT00035049001	1,06	0,00012	1
GSPATT00015363001	-0,898	0,00028	0	GSPATT00035050001	1,458	0,0075	1
GSPATT00015373001	0,163	0,24261	0	GSPATT00035051001	1,15	0,00008	1
GSPATT00015374001	0,211	0,22091	0	GSPATT00035055001	-2,179	0	-1
GSPATT00015382001	1,396	0,00009	1	GSPATT00035058001	0,603	0,00388	0
GSPATT00015396001	0,853	0,02037	0	GSPATT00035098001	-0,426	0,01033	0
GSPATT00015413001	1,181	0,00207	1	GSPATT00035101001	0,274	0,13516	0
GSPATT00015428001	-0,166	0,38106	0	GSPATT00035109001	0,17	0,21947	0
GSPATT00015448001	1,213	0,00004	1	GSPATT00035110001	0,776	0,02913	0
GSPATT00015474001	0,646	0,00966	0	GSPATT00035113001	-3,146	0	-1
GSPATT00015475001	0,366	0,0282	0	GSPATT00035115001	0,815	0,01116	0
GSPATT00015479001	0,147	0,2715	0	GSPATT00035116001	0,615	0,00474	0
GSPATT00015586001	-0,146	0,30933	0	GSPATT00035132001	0,541	0,03772	0
GSPATT00015587001	-0,173	0,20164	0	GSPATT00035156001	0,909	0,02167	0
GSPATT00015588001	0,437	0,0437	0	GSPATT00035162001	0,462	0,02625	0
GSPATT00015597001	0,345	0,07945	0	GSPATT00035168001	-0,967	0,00127	0
GSPATT00015625001	-1,091	0,00006	-1	GSPATT00035170001	0,275	0,20192	0
GSPATT00015632001	1,616	0,00334	1	GSPATT00035215001	-0,514	0,05916	0
GSPATT00015635001	0,054	0,76188	0	GSPATT00035221001	1,144	0,00247	1
GSPATT00015640001	0,853	0,00185	0	GSPATT00035230001	0,202	0,239	0
GSPATT00015666001	-0,623	0,00761	0	GSPATT00035231001	0,708	0,00161	0
GSPATT00015671001	-0,147	0,30793	0	GSPATT00035242001	0,289	0,32037	0
GSPATT00015678001	0,121	0,52246	0	GSPATT00035265001	-0,98	0,00136	0
GSPATT00015687001	0,716	0,00115	0	GSPATT00035273001	0,212	0,15905	0
GSPATT00015690001	0,723	0,00416	0	GSPATT00035283001	0,586	0,00587	0
GSPATT00015693001	-0,397	0,09858	0	GSPATT00035344001	1,515	0,00002	1
GSPATT00015702001	0,703	0,00191	0	GSPATT00035345001	0,075	0,59803	0
GSPATT00015703001	0,498	0,0388	0	GSPATT00035369001	1,788	0	1
GSPATT00015729001	0,223	0,18041	0	GSPATT00035370001	0,774	0,00252	0
GSPATT00015750001	1,122	0,00018	1	GSPATT00035435001	0,252	0,14704	0
GSPATT00015756001	0,261	0,13282	0	GSPATT00035439001	0,245	0,18477	0
GSPATT00015783001	0,994	0,00013	0	GSPATT00035512001	0,145	0,51374	0
GSPATT00015795001	1,253	0,00003	1	GSPATT00035537001	-0,316	0,13551	0
GSPATT00015804001	-0,028	0,89234	0	GSPATT00035553001	0,19	0,42969	0
GSPATT00015821001	-0,236	0,16594	0	GSPATT00035558001	0,435	0,06307	0
GSPATT00015826001	-1,025	0,00242	-1	GSPATT00035563001	0,506	0,20376	0
GSPATT00015830001	2,01	0,00006	1	GSPATT00035570001	0,438	0,14386	0
GSPATT00015852001	0,793	0,01264	0	GSPATT00035586001	0,216	0,24577	0
GSPATT00015854001	-0,969	0,00154	0	GSPATT00035588001	1,375	0,00072	1
GSPATT00015864001	0,541	0,09018	0	GSPATT00035590001	-0,098	0,61368	0
GSPATT00015881001	0,435	0,06228	0	GSPATT00035599001	0,523	0,00967	0
GSPATT00015890001	0,521	0,0327	0	GSPATT00035613001	0,93	0,00031	0
GSPATT00015896001	0,187	0,19576	0	GSPATT00035630001	0,599	0,00235	0
GSPATT00015906001	-0,376	0,07667	0	GSPATT00035632001	1,647	0,00001	1
GSPATT00015913001	-3,714	0	-1	GSPATT00035666001	0,319	0,04691	0

GSPATT00015946001	-0,176	0,47867	0	GSPATT00035684001	0,079	0,61168	0
GSPATT00015989001	0,756	0,0358	0	GSPATT00035689001	-0,122	0,45197	0
GSPATT00015997001	0,616	0,05511	0	GSPATT00035711001	-0,148	0,32112	0
GSPATT00015998001	0,057	0,70953	0	GSPATT00035742001	-0,345	0,03219	0
GSPATT00016007001	-1,006	0,00099	-1	GSPATT00035743001	-0,119	0,44406	0
GSPATT00016014001	0,361	0,04847	0	GSPATT00035748001	0,686	0,00459	0
GSPATT00016022001	0,297	0,04559	0	GSPATT00035791001	-0,365	0,03388	0
GSPATT00016031001	1,983	0	1	GSPATT00035808001	-0,036	0,8091	0
GSPATT00016058001	0,403	0,0261	0	GSPATT00035812001	0,025	0,89409	0
GSPATT00016068001	0,584	0,00865	0	GSPATT00035830001	0,647	0,0186	0
GSPATT00016075001	0,476	0,01419	0	GSPATT00035834001	0,834	0,00201	0
GSPATT00016078001	-0,46	0,01198	0	GSPATT00035841001	-0,305	0,05898	0
GSPATT00016099001	-0,376	0,0569	0	GSPATT00035846001	-0,018	0,91105	0
GSPATT00016118001	0,681	0,00081	0	GSPATT00035878001	-0,682	0,01003	0
GSPATT00016163001	0,936	0,00075	0	GSPATT00035892001	0,871	0,00133	0
GSPATT00016186001	0,651	0,00328	0	GSPATT00035967001	-1,028	0,00178	-1
GSPATT00016201001	0,264	0,12078	0	GSPATT00035984001	0,033	0,81622	0
GSPATT00016202001	0,942	0,0002	0	GSPATT00035987001	0,061	0,629	0
GSPATT00016224001	0,577	0,00541	0	GSPATT00035988001	0,121	0,62093	0
GSPATT00016246001	1,272	0,00554	1	GSPATT00035996001	1,583	0,00009	1
GSPATT00016252001	-1,875	0,00002	-1	GSPATT00036001001	0,324	0,09202	0
GSPATT00016286001	0,878	0,0129	0	GSPATT00036011001	0,418	0,0183	0
GSPATT00016304001	0,003	0,98948	0	GSPATT00036020001	0,633	0,01487	0
GSPATT00016323001	0,645	0,02514	0	GSPATT00036021001	-0,784	0,08263	0
GSPATT00016324001	0,417	0,15142	0	GSPATT00036025001	-0,396	0,01622	0
GSPATT00016327001	0,649	0,00694	0	GSPATT00036029001	0,388	0,03177	0
GSPATT00016370001	0,574	0,02089	0	GSPATT00036032001	-0,747	0,00511	0
GSPATT00016374001	0,711	0,02701	0	GSPATT00036035001	1,064	0,00084	1
GSPATT00016390001	-0,025	0,88764	0	GSPATT00036037001	0,29	0,15131	0
GSPATT00016393001	0,939	0,0007	0	GSPATT00036059001	-2,602	0,00001	-1
GSPATT00016396001	-0,359	0,34319	0	GSPATT00036080001	-0,995	0,00114	0
GSPATT00016433001	0,343	0,0523	0	GSPATT00036130001	1,008	0,00009	1
GSPATT00016451001	0,509	0,00351	0	GSPATT00036133001	0,425	0,08763	0
GSPATT00016461001	2,349	0	1	GSPATT00036142001	0,347	0,06142	0
GSPATT00016467001	0,07	0,6374	0	GSPATT00036146001	-0,276	0,13155	0
GSPATT00016473001	-0,231	0,19236	0	GSPATT00036182001	0,139	0,41765	0
GSPATT00016474001	-0,225	0,18313	0	GSPATT00036198001	0,154	0,39188	0
GSPATT00016484001	0,021	0,92665	0	GSPATT00036212001	0,53	0,00883	0
GSPATT00016487001	0,393	0,10186	0	GSPATT00036217001	-0,481	0,07457	0
GSPATT00016492001	1,714	0,00007	1	GSPATT00036238001	0,234	0,24062	0
GSPATT00016497001	0,83	0,00243	0	GSPATT00036250001	0,42	0,01334	0
GSPATT00016499001	2,913	0	1	GSPATT00036252001	-0,676	0,01753	0
GSPATT00016501001	0,408	0,05581	0	GSPATT00036267001	1,177	0,00036	1
GSPATT00016508001	-0,185	0,31276	0	GSPATT00036286001	-0,683	0,00156	0
GSPATT00016522001	1,486	0,00001	1	GSPATT00036336001	0,222	0,12243	0
GSPATT00016536001	0,338	0,11356	0	GSPATT00036337001	0,687	0,00442	0
GSPATT00016555001	0,08	0,62165	0	GSPATT00036353001	0,737	0,00133	0
GSPATT00016572001	0,158	0,27397	0	GSPATT00036407001	0,356	0,04968	0
GSPATT00016579001	-0,042	0,75571	0	GSPATT00036409001	0,316	0,04048	0
GSPATT00016580001	1,975	0	1	GSPATT00036434001	0,218	0,17014	0
GSPATT00016581001	0,583	0,00498	0	GSPATT00036445001	0,343	0,02548	0
GSPATT00016584001	0,48	0,00759	0	GSPATT00036459001	1,229	0,0001	1
GSPATT00016598001	-0,315	0,17406	0	GSPATT00036469001	0,587	0,01017	0
GSPATT00016621001	-0,635	0,00167	0	GSPATT00036471001	0,051	0,77388	0
GSPATT00016625001	0,578	0,01915	0	GSPATT00036474001	0,054	0,83167	0
GSPATT00016626001	-1,01	0,01795	-1	GSPATT00036479001	-0,35	0,11614	0
GSPATT00016635001	1,276	0,00002	1	GSPATT00036483001	-0,011	0,94317	0
GSPATT00016642001	0,83	0,00038	0	GSPATT00036486001	-0,301	0,19234	0
GSPATT00016644001	-0,085	0,60496	0	GSPATT00036520001	1,333	0,00049	1
GSPATT00016649001	0,549	0,0028	0	GSPATT00036521001	-1,453	0,00724	-1
GSPATT00016655001	-0,315	0,1207	0	GSPATT00036542001	2,353	0	1
GSPATT00016684001	0,371	0,03571	0	GSPATT00036543001	3,458	0	1
GSPATT00016686001	-0,649	0,0057	0	GSPATT00036578001	0,012	0,94123	0
GSPATT00016692001	0,905	0,00153	0	GSPATT00036602001	-0,016	0,91827	0
GSPATT00016707001	0,035	0,81827	0	GSPATT00036604001	0,849	0,00077	0
GSPATT00016715001	-0,125	0,40578	0	GSPATT00036632001	0,404	0,0432	0
GSPATT00016728001	0,796	0,00369	0	GSPATT00036666001	-0,42	0,03381	0

GSPATT00016739001	0,05	0,81698	0	GSPATT00036671001	-0,905	0,00646	0
GSPATT00016753001	0,303	0,03871	0	GSPATT00036678001	0,014	0,9207	0
GSPATT00016756001	0,551	0,01981	0	GSPATT00036688001	0,31	0,04238	0
GSPATT00016834001	-0,177	0,2236	0	GSPATT00036690001	0,725	0,00628	0
GSPATT00016841001	0,199	0,27702	0	GSPATT00036705001	1,156	0,00008	1
GSPATT00016868001	-0,668	0,00534	0	GSPATT00036706001	0,883	0,00746	0
GSPATT00016887001	-0,259	0,12658	0	GSPATT00036730001	-0,04	0,79056	0
GSPATT00016943001	-0,089	0,60758	0	GSPATT00036752001	0,349	0,08868	0
GSPATT00016964001	0,04	0,87452	0	GSPATT00036770001	0,189	0,34256	0
GSPATT00016974001	-0,017	0,9121	0	GSPATT00036776001	-0,3	0,06534	0
GSPATT00016976001	1,509	0,00004	1	GSPATT00036800001	-0,04	0,79823	0
GSPATT00016985001	0,32	0,1398	0	GSPATT00036806001	-0,03	0,88012	0
GSPATT00017038001	2	0	1	GSPATT00036817001	-0,834	0,01188	0
GSPATT00017050001	0,473	0,00736	0	GSPATT00036847001	-0,022	0,88003	0
GSPATT00017063001	0,061	0,69591	0	GSPATT00036862001	-1,618	0,00012	-1
GSPATT00017094001	0,37	0,12392	0	GSPATT00036876001	0,655	0,01693	0
GSPATT00017107001	0,512	0,01311	0	GSPATT00036879001	0,685	0,00059	0
GSPATT00017117001	1,483	0,00007	1	GSPATT00036888001	0,452	0,00944	0
GSPATT00017122001	-0,89	0,00112	0	GSPATT00036914001	0,754	0,10066	0
GSPATT00017148001	0,445	0,01161	0	GSPATT00036919001	-0,269	0,14769	0
GSPATT00017157001	0,228	0,29231	0	GSPATT00036954001	-0,592	0,0063	0
GSPATT00017193001	0,423	0,02362	0	GSPATT00036971001	-0,218	0,16152	0
GSPATT00017227001	0,713	0,01736	0	GSPATT00037000001	0,283	0,14601	0
GSPATT00017228001	-1,143	0,00016	-1	GSPATT00037053001	-0,135	0,29881	0
GSPATT00017248001	0,004	0,98061	0	GSPATT00037058001	0,084	0,66195	0
GSPATT00017265001	0,224	0,27585	0	GSPATT00037066001	-0,414	0,01584	0
GSPATT00017287001	0,722	0,00261	0	GSPATT00037088001	0,441	0,01862	0
GSPATT00017341001	-0,483	0,07425	0	GSPATT00037094001	-0,008	0,9585	0
GSPATT00017350001	-0,237	0,16156	0	GSPATT00037113001	-0,126	0,51661	0
GSPATT00017354001	-0,088	0,60425	0	GSPATT00037123001	0,774	0,01287	0
GSPATT00017356001	1,623	0,00011	1	GSPATT00037129001	0,656	0,00104	0
GSPATT00017361001	0,54	0,0029	0	GSPATT00037143001	-0,027	0,85661	0
GSPATT00017388001	-0,64	0,00112	0	GSPATT00037145001	0,616	0,02922	0
GSPATT00017455001	0,77	0,0007	0	GSPATT00037148001	-0,358	0,09184	0
GSPATT00017492001	0,873	0,0003	0	GSPATT00037154001	-0,557	0,01543	0
GSPATT00017522001	0,536	0,05133	0	GSPATT00037160001	1,034	0,00084	1
GSPATT00017543001	1,645	0,00001	1	GSPATT00037166001	1	0,00065	0
GSPATT00017557001	0,172	0,32619	0	GSPATT00037168001	0,397	0,02983	0
GSPATT00017576001	1,079	0,00068	1	GSPATT00037172001	0,145	0,33707	0
GSPATT00017630001	-0,188	0,2095	0	GSPATT00037211001	0,931	0,00079	0
GSPATT00017662001	0,243	0,10339	0	GSPATT00037212001	0,689	0,00673	0
GSPATT00017666001	0,164	0,29665	0	GSPATT00037226001	0,202	0,30966	0
GSPATT00017671001	1,215	0,00163	1	GSPATT00037232001	0,667	0,00174	0
GSPATT00017672001	0,666	0,00486	0	GSPATT00037285001	0,579	0,00719	0
GSPATT00017676001	0,897	0,00014	0	GSPATT00037291001	-2,613	0,00006	-1
GSPATT00017680001	1,263	0,0002	1	GSPATT00037311001	-0,268	0,09834	0
GSPATT00017681001	1,015	0,00199	1	GSPATT00037328001	0,772	0,00301	0
GSPATT00017692001	0,757	0,02894	0	GSPATT00037330001	0,474	0,01266	0
GSPATT00017712001	-0,359	0,17781	0	GSPATT00037368001	1,031	0,0019	1
GSPATT00017715001	1,019	0,00017	1	GSPATT00037376001	0,236	0,20339	0
GSPATT00017746001	0,26	0,0958	0	GSPATT00037398001	0,097	0,63269	0
GSPATT00017766001	-1,364	0,00026	-1	GSPATT00037444001	-0,662	0,00643	0
GSPATT00017771001	0,257	0,1542	0	GSPATT00037451001	0,414	0,01229	0
GSPATT00017791001	-1,005	0,00013	-1	GSPATT00037471001	0,104	0,58331	0
GSPATT00017820001	0,089	0,63426	0	GSPATT00037488001	1,394	0,00067	1
GSPATT00017833001	1,804	0,00001	1	GSPATT00037526001	0,897	0,00043	0
GSPATT00017845001	-0,337	0,04954	0	GSPATT00037566001	-0,05	0,76612	0
GSPATT00017866001	-0,949	0,00081	0	GSPATT00037597001	-0,102	0,55503	0
GSPATT00017868001	-0,188	0,4063	0	GSPATT00037660001	0,186	0,17268	0
GSPATT00017882001	-0,011	0,94767	0	GSPATT00037661001	0,066	0,71158	0
GSPATT00017902001	-0,048	0,73859	0	GSPATT00037688001	0,303	0,04893	0
GSPATT00017904001	0,196	0,20412	0	GSPATT00037699001	0,741	0,00075	0
GSPATT00017915001	0,763	0,00829	0	GSPATT00037711001	-0,444	0,05547	0
GSPATT00017926001	-0,085	0,54936	0	GSPATT00037715001	0,109	0,42522	0
GSPATT00017952001	0,822	0,00042	0	GSPATT00037722001	0,821	0,0005	0
GSPATT00017953001	0,93	0,00178	0	GSPATT00037727001	0,543	0,04234	0
GSPATT00017960001	-0,674	0,00313	0	GSPATT00037737001	1,364	0,0001	1

GSPATT00017963001	-0,275	0,06466	0	GSPATT00037754001	-0,856	0,0023	0
GSPATT00017966001	0,227	0,14352	0	GSPATT00037781001	-1,255	0,00102	-1
GSPATT00017972001	-0,131	0,38508	0	GSPATT00037784001	0,072	0,57853	0
GSPATT00017992001	-0,11	0,50579	0	GSPATT00037789001	2,068	0	1
GSPATT00018004001	0,463	0,03497	0	GSPATT00037792001	-0,369	0,18448	0
GSPATT00018101001	0,898	0,00614	0	GSPATT00037795001	0,362	0,01982	0
GSPATT00018127001	0,153	0,36392	0	GSPATT00037796001	1,094	0,0001	1
GSPATT00018132001	0,741	0,02921	0	GSPATT00037798001	0,035	0,81328	0
GSPATT00018133001	-0,081	0,66594	0	GSPATT00037811001	0,21	0,2921	0
GSPATT00018143001	0,268	0,2351	0	GSPATT00037815001	0,925	0,00331	0
GSPATT00018153001	-0,086	0,72806	0	GSPATT00037818001	0,928	0,00072	0
GSPATT00018163001	0,342	0,02849	0	GSPATT00037856001	0,303	0,06934	0
GSPATT00018188001	0,221	0,21776	0	GSPATT00037875001	-0,066	0,66305	0
GSPATT00018202001	0,66	0,01285	0	GSPATT00037876001	0,446	0,01048	0
GSPATT00018223001	-0,409	0,02988	0	GSPATT00037939001	0,394	0,02006	0
GSPATT00018263001	0,814	0,00153	0	GSPATT00037945001	-0,437	0,02189	0
GSPATT00018280001	0,316	0,0745	0	GSPATT00037967001	-0,06	0,6776	0
GSPATT00018290001	-0,359	0,04447	0	GSPATT00037978001	0,461	0,08571	0
GSPATT00018291001	0,141	0,45858	0	GSPATT00038025001	-0,104	0,48304	0
GSPATT00018293001	0,169	0,30306	0	GSPATT00038027001	0,502	0,05619	0
GSPATT00018324001	0,468	0,06312	0	GSPATT00038035001	0,888	0,00025	0
GSPATT00018389001	-0,094	0,58557	0	GSPATT00038045001	-0,396	0,05929	0
GSPATT00018398001	0,481	0,09586	0	GSPATT00038090001	1,233	0,00044	1
GSPATT00018431001	0,56	0,04386	0	GSPATT00038149001	0,258	0,07307	0
GSPATT00018438001	0,545	0,01322	0	GSPATT00038155001	0,75	0,00768	0
GSPATT00018449001	0,58	0,0723	0	GSPATT00038156001	0,107	0,61347	0
GSPATT00018471001	-1,113	0,00027	-1	GSPATT00038162001	-0,205	0,24742	0
GSPATT00018476001	0,992	0,00041	0	GSPATT00038171001	-1,199	0,00032	-1
GSPATT00018515001	0,206	0,16244	0	GSPATT00038175001	-0,718	0,00151	0
GSPATT00018529001	0,92	0,01084	0	GSPATT00038196001	0,025	0,86468	0
GSPATT00018559001	0,48	0,01489	0	GSPATT00038198001	1,751	0,00019	1
GSPATT00018563001	0,407	0,05649	0	GSPATT00038202001	1,049	0,00014	1
GSPATT00018600001	0,51	0,01239	0	GSPATT00038203001	1,279	0,00007	1
GSPATT00018603001	-0,883	0,002	0	GSPATT00038212001	0,313	0,14446	0
GSPATT00018608001	0,219	0,14898	0	GSPATT00038214001	2,959	0	1
GSPATT00018634001	-0,253	0,0798	0	GSPATT00038217001	1,138	0,00032	1
GSPATT00018635001	-4,269	0	-1	GSPATT00038232001	-1,557	0,00002	-1
GSPATT00018645001	-0,232	0,15845	0	GSPATT00038245001	0,014	0,92514	0
GSPATT00018666001	0,125	0,44695	0	GSPATT00038377001	1,241	0,00049	1
GSPATT00018671001	0,675	0,00088	0	GSPATT00038439001	0,034	0,86173	0
GSPATT00018681001	-0,683	0,00496	0	GSPATT00038442001	0,573	0,00219	0
GSPATT00018695001	-0,076	0,62948	0	GSPATT00038453001	0,958	0,00056	0
GSPATT00018702001	-0,494	0,02855	0	GSPATT00038457001	0,034	0,80944	0
GSPATT00018706001	0,301	0,2524	0	GSPATT00038462001	0,25	0,17848	0
GSPATT00018710001	-0,217	0,14626	0	GSPATT00038469001	1,122	0,00009	1
GSPATT00018727001	-0,029	0,84109	0	GSPATT00038489001	0,49	0,00846	0
GSPATT00018728001	-0,429	0,04057	0	GSPATT00038497001	0,218	0,14041	0
GSPATT00018737001	0,112	0,42429	0	GSPATT00038500001	-0,057	0,70837	0
GSPATT00018780001	0,691	0,01868	0	GSPATT00038501001	0,115	0,39982	0
GSPATT00018806001	-0,089	0,50827	0	GSPATT00038509001	0,207	0,13138	0
GSPATT00018878001	-0,526	0,01436	0	GSPATT00038517001	0,388	0,06212	0
GSPATT00018891001	-0,275	0,12204	0	GSPATT00038525001	0,766	0,00172	0
GSPATT00018918001	0,815	0,00108	0	GSPATT00038565001	-0,503	0,00816	0
GSPATT00018950001	0,098	0,47385	0	GSPATT00038594001	-0,531	0,01391	0
GSPATT00018952001	0,199	0,36354	0	GSPATT00038759001	0,112	0,58404	0
GSPATT00018964001	0,792	0,02949	0	GSPATT00038767001	0,906	0,00064	0
GSPATT00018965001	0,578	0,00897	0	GSPATT00038772001	0,189	0,25284	0
GSPATT00018969001	-0,459	0,03222	0	GSPATT00038778001	0,45	0,0114	0
GSPATT00018978001	1,009	0,00294	1	GSPATT00038811001	0,163	0,23056	0
GSPATT00019012001	0,241	0,25273	0	GSPATT00038843001	0,394	0,08578	0
GSPATT00019047001	-0,313	0,12451	0	GSPATT00038849001	0,132	0,34446	0
GSPATT00019068001	0,352	0,08916	0	GSPATT00038851001	-0,622	0,0036	0
GSPATT00019089001	-0,092	0,50555	0	GSPATT00038853001	0,357	0,03403	0
GSPATT00019098001	-0,396	0,01375	0	GSPATT00038854001	0,067	0,67394	0
GSPATT00019105001	-0,564	0,03875	0	GSPATT00038872001	-0,347	0,10873	0
GSPATT00019121001	-0,238	0,13986	0	GSPATT00038895001	-2,984	0,00003	-1
GSPATT00019142001	0,119	0,67944	0	GSPATT00038896001	-0,151	0,65702	0

GSPATT00019147001	1,334	0,00037	1	GSPATT00038914001	1,241	0,00033	1
GSPATT00019217001	0,697	0,00662	0	GSPATT00038932001	0,144	0,35304	0
GSPATT00019232001	1,248	0,0003	1	GSPATT00038938001	0,67	0,06631	0
GSPATT00019272001	0,626	0,00167	0	GSPATT00039020001	1,009	0,00006	1
GSPATT00019281001	0,242	0,15235	0	GSPATT00039053001	0,586	0,00254	0
GSPATT00019357001	-0,269	0,11687	0	GSPATT00039058001	0,227	0,19888	0
GSPATT00019361001	1,018	0,00339	1	GSPATT00039143001	0,942	0,00145	0
GSPATT00019366001	0,029	0,8276	0	GSPATT00039147001	-0,456	0,10233	0
GSPATT00019371001	-1,072	0,00005	-1	GSPATT00039305001	0,494	0,01965	0
GSPATT00019421001	0,515	0,01534	0	GSPATT00039325001	0,34	0,08666	0
GSPATT00019447001	1,328	0,00111	1	GSPATT00039337001	0,067	0,62405	0
GSPATT00019448001	1,274	0,00003	1	GSPATT00039369001	-0,01	0,94511	0
GSPATT00019460001	0,582	0,01671	0	GSPATT00039403001	1,013	0,00053	1
GSPATT00019477001	-0,546	0,02587	0	GSPATT00039502001	-0,434	0,08958	0
GSPATT00019493001	-0,409	0,16665	0	GSPATT00039519001	0,599	0,01071	0
GSPATT00019504001	0,507	0,03568	0	GSPATT00039578001	1,071	0,0029	1
GSPATT00019520001	1,19	0,00006	1	GSPATT00039596001	-0,065	0,63218	0
GSPATT00019546001	0,939	0,00501	0	GSPATT00039617001	-0,125	0,38541	0
GSPATT00019584001	0,197	0,24399	0	GSPATT00039623001	-0,179	0,37866	0
GSPATT00019589001	-0,23	0,14791	0	GSPATT00039672001	0,81	0,01218	0
GSPATT00019597001	0,628	0,00396	0	GSPATT00039701001	0,824	0,06378	0
GSPATT00019603001	0,551	0,01253	0	GSPATT00039733001	0,707	0,00233	0
GSPATT00019614001	-0,092	0,48818	0	GSPATT00039825001	0,881	0,00181	0
GSPATT00019621001	1,048	0,0001	1	PTETT10500005001	0,689	0,01507	0
GSPATT00019623001	0,397	0,02171	0	PTETT10800002001	-0,131	0,51208	0

GO 0006468 protein phosphorylation

ID	coeff.	p-value	signif.	ID	coeff.	p-value	signif.
GSPATT00000029001	-0,656	0,02257	0	GSPATT00019734001	0,538	0,01044	0
GSPATT00000047001	0,686	0,01107	0	GSPATT00019758001	-0,857	0,00047	0
GSPATT00000064001	-0,605	0,00365	0	GSPATT00019770001	0,159	0,3745	0
GSPATT00000093001	0,319	0,15526	0	GSPATT00019834001	0,855	0,00735	0
GSPATT00000160001	-0,436	0,09808	0	GSPATT00019835001	0,65	0,03101	0
GSPATT00000190001	0,762	0,00321	0	GSPATT00019840001	0,546	0,02765	0
GSPATT00000282001	-0,179	0,30932	0	GSPATT00019861001	0,155	0,42691	0
GSPATT00000295001	0,583	0,02578	0	GSPATT00019885001	1,059	0,00032	1
GSPATT00000324001	-0,092	0,63602	0	GSPATT00019888001	1,012	0,00029	1
GSPATT00000365001	0,81	0,00102	0	GSPATT00019898001	0,291	0,35823	0
GSPATT00000366001	0,639	0,01895	0	GSPATT00019899001	1,302	0,01819	1
GSPATT00000384001	0,653	0,02308	0	GSPATT00019918001	1,36	0,00661	1
GSPATT00000394001	-0,275	0,16617	0	GSPATT00019930001	-0,02	0,89979	0
GSPATT00000415001	-0,571	0,00652	0	GSPATT00019935001	0,306	0,30175	0
GSPATT00000434001	0,504	0,02973	0	GSPATT00019962001	0,101	0,63424	0
GSPATT00000482001	-0,044	0,82968	0	GSPATT00019970001	0,305	0,11022	0
GSPATT00000508001	-0,009	0,95625	0	GSPATT00019974001	0,112	0,7056	0
GSPATT00000542001	0,969	0,00094	0	GSPATT00019988001	-0,077	0,59245	0
GSPATT00000559001	0,401	0,04709	0	GSPATT00020006001	0,694	0,0227	0
GSPATT00000560001	0,804	0,0092	0	GSPATT00020016001	-0,284	0,16066	0
GSPATT00000594001	-1,714	0,00001	-1	GSPATT00020018001	0,567	0,00877	0
GSPATT00000596001	-1,334	0,00022	-1	GSPATT00020040001	0,426	0,13971	0
GSPATT00000599001	-0,212	0,13935	0	GSPATT00020051001	0,424	0,02871	0
GSPATT00000615001	0,04	0,80664	0	GSPATT00020056001	-0,263	0,09627	0
GSPATT00000619001	0,52	0,00518	0	GSPATT00020061001	0,203	0,18858	0
GSPATT00000630001	0,066	0,80692	0	GSPATT00020081001	0,562	0,01428	0
GSPATT00000664001	0,473	0,02458	0	GSPATT00020083001	-0,007	0,96307	0
GSPATT00000685001	0,283	0,12345	0	GSPATT00020106001	0,617	0,04707	0
GSPATT00000704001	1,505	0,00001	1	GSPATT00020133001	0,915	0,00052	0
GSPATT00000760001	1,018	0,00234	1	GSPATT00020136001	0,721	0,00761	0
GSPATT00000783001	0,956	0,007	0	GSPATT00020148001	0,628	0,00191	0
GSPATT00000795001	0,73	0,00542	0	GSPATT00020151001	0,152	0,25786	0
GSPATT00000817001	-0,289	0,11652	0	GSPATT00020164001	0,049	0,79466	0
GSPATT00000822001	0,006	0,96587	0	GSPATT00020171001	0,019	0,89222	0
GSPATT00000824001	1,895	0,00001	1	GSPATT00020175001	-0,408	0,07148	0
GSPATT00000926001	-0,44	0,07676	0	GSPATT00020206001	1,187	0,00008	1
GSPATT00000934001	0,7	0,00134	0	GSPATT00020222001	0,022	0,8861	0
GSPATT00000950001	-0,039	0,85119	0	GSPATT00020225001	0,491	0,02758	0
GSPATT00000961001	1,244	0,00014	1	GSPATT00020239001	-0,066	0,6879	0

GSPATT00000985001	-0,053	0,78626	0	GSPATT00020280001	0,417	0,00934	0
GSPATT00001013001	0,18	0,3735	0	GSPATT00020292001	0,983	0,00426	0
GSPATT00001029001	0,351	0,04038	0	GSPATT00020315001	0,421	0,02041	0
GSPATT00001071001	-1,676	0,00003	-1	GSPATT00020317001	1,39	0,00016	1
GSPATT00001077001	-1,834	0	-1	GSPATT00020328001	0,132	0,45889	0
GSPATT00001082001	0,802	0,00363	0	GSPATT00020354001	0,971	0,00377	0
GSPATT00001085001	-0,102	0,55835	0	GSPATT00020357001	0,423	0,11371	0
GSPATT00001088001	-1,002	0,00029	-1	GSPATT00020375001	0,159	0,26389	0
GSPATT00001107001	1,346	0,00005	1	GSPATT00020379001	0,528	0,00684	0
GSPATT00001118001	0,362	0,12047	0	GSPATT00020390001	-1,113	0,00014	-1
GSPATT00001133001	-0,118	0,39808	0	GSPATT00020397001	0,42	0,02881	0
GSPATT00001155001	-0,688	0,04688	0	GSPATT00020450001	0,494	0,01321	0
GSPATT00001162001	-0,208	0,17385	0	GSPATT00020501001	0,141	0,29138	0
GSPATT00001183001	-1,126	0,00625	-1	GSPATT00020577001	0,213	0,12758	0
GSPATT00001189001	-0,09	0,61561	0	GSPATT00020591001	0,653	0,02277	0
GSPATT00001207001	0,62	0,00301	0	GSPATT00020594001	0,125	0,38102	0
GSPATT00001216001	-0,859	0,0049	0	GSPATT00020603001	0,424	0,03461	0
GSPATT00001230001	0,185	0,20391	0	GSPATT00020607001	0,553	0,03187	0
GSPATT00001255001	-0,19	0,27269	0	GSPATT00020634001	-0,891	0,00039	0
GSPATT00001260001	1,21	0,00035	1	GSPATT00020656001	0,384	0,09254	0
GSPATT00001268001	-0,542	0,00397	0	GSPATT00020671001	0,4	0,06339	0
GSPATT00001273001	0,742	0,00126	0	GSPATT00020675001	0,023	0,86196	0
GSPATT00001279001	0,193	0,33618	0	GSPATT00020696001	1,227	0,00012	1
GSPATT00001292001	-0,196	0,32171	0	GSPATT00020706001	-2,177	0,00005	-1
GSPATT00001310001	0,604	0,00221	0	GSPATT00020718001	0,498	0,0293	0
GSPATT00001314001	0,32	0,07355	0	GSPATT00020723001	1,447	0,00002	1
GSPATT00001320001	0,724	0,00683	0	GSPATT00020732001	0,346	0,02628	0
GSPATT00001342001	0,712	0,00317	0	GSPATT00020736001	-0,014	0,93682	0
GSPATT00001359001	0,036	0,83426	0	GSPATT00020738001	-0,47	0,01442	0
GSPATT00001361001	0,243	0,21121	0	GSPATT00020743001	0,121	0,41116	0
GSPATT00001369001	0,226	0,16319	0	GSPATT00020748001	0,638	0,0469	0
GSPATT00001413001	-0,893	0,00057	0	GSPATT00020754001	0,328	0,15362	0
GSPATT00001414001	0,294	0,08325	0	GSPATT00020755001	1,124	0,00008	1
GSPATT00001427001	0,007	0,96536	0	GSPATT00020757001	0,109	0,4162	0
GSPATT00001430001	0,427	0,01633	0	GSPATT00020787001	0,074	0,69474	0
GSPATT00001441001	-0,411	0,0844	0	GSPATT00020798001	-0,359	0,06684	0
GSPATT00001463001	1,174	0,00247	1	GSPATT00020807001	0,453	0,03103	0
GSPATT00001467001	-0,474	0,02385	0	GSPATT00020812001	0,207	0,32984	0
GSPATT00001471001	0,442	0,16312	0	GSPATT00020816001	-0,014	0,954	0
GSPATT00001479001	0,193	0,3499	0	GSPATT00020817001	0,81	0,00504	0
GSPATT00001494001	-0,073	0,59749	0	GSPATT00020820001	0,296	0,15516	0
GSPATT00001504001	0,05	0,75727	0	GSPATT00020837001	-2,103	0	-1
GSPATT00001508001	0,42	0,01888	0	GSPATT00020863001	1,067	0,00014	1
GSPATT00001527001	0,128	0,47689	0	GSPATT00020867001	-0,219	0,23135	0
GSPATT00001554001	0,882	0,00038	0	GSPATT00020886001	0,561	0,00314	0
GSPATT00001563001	-0,431	0,02903	0	GSPATT00020887001	0,231	0,17424	0
GSPATT00001592001	1,698	0,00001	1	GSPATT00020897001	0,202	0,34688	0
GSPATT00001610001	0,834	0,00147	0	GSPATT00020921001	0,201	0,27627	0
GSPATT00001650001	0,32	0,08832	0	GSPATT00020942001	-0,24	0,19484	0
GSPATT00001662001	0,438	0,17856	0	GSPATT00021004001	-0,039	0,80561	0
GSPATT00001682001	-2,067	0,00007	-1	GSPATT00021006001	-0,917	0,00215	0
GSPATT00001698001	-0,612	0,00264	0	GSPATT00021015001	-0,087	0,60216	0
GSPATT00001746001	1,163	0,00005	1	GSPATT00021056001	-0,152	0,53854	0
GSPATT00001753001	0,102	0,57872	0	GSPATT00021080001	-0,038	0,88109	0
GSPATT00001779001	0,661	0,00281	0	GSPATT00021083001	1,214	0,00004	1
GSPATT00001789001	0,667	0,00963	0	GSPATT00021104001	1,014	0,00009	1
GSPATT00001794001	-0,276	0,08218	0	GSPATT00021108001	0,03	0,85909	0
GSPATT00001840001	0,602	0,00734	0	GSPATT00021127001	0,409	0,03217	0
GSPATT00001852001	1,949	0	1	GSPATT00021152001	0,566	0,00438	0
GSPATT00001853001	1,087	0,00026	1	GSPATT00021175001	0,819	0,00079	0
GSPATT00001854001	1,3	0,00007	1	GSPATT00021195001	1,22	0,0001	1
GSPATT00001859001	0,31	0,07571	0	GSPATT00021201001	0,446	0,02497	0
GSPATT00001864001	0,864	0,00071	0	GSPATT00021207001	0,704	0,01544	0
GSPATT00001865001	0,55	0,00393	0	GSPATT00021213001	-0,327	0,35092	0
GSPATT00001866001	0,294	0,40339	0	GSPATT00021222001	-1,069	0,00073	-1
GSPATT00001870001	0,431	0,01514	0	GSPATT00021227001	0,331	0,04285	0
GSPATT00001873001	2,117	0,00001	1	GSPATT00021248001	-0,222	0,12494	0

GSPATT00001911001	-0,107	0,62983	0	GSPATT00021249001	-0,183	0,27756	0
GSPATT00002000001	0,994	0,00542	0	GSPATT00021250001	0,181	0,35664	0
GSPATT00002011001	0,938	0,00081	0	GSPATT00021252001	2,647	0	1
GSPATT00002013001	0,586	0,02656	0	GSPATT00021256001	0,688	0,00291	0
GSPATT00002014001	0,546	0,01206	0	GSPATT00021258001	1,026	0,00012	1
GSPATT00002063001	0,381	0,02118	0	GSPATT00021270001	0,517	0,01282	0
GSPATT00002065001	0,716	0,00677	0	GSPATT00021271001	0,429	0,01704	0
GSPATT00002068001	1,376	0,00007	1	GSPATT00021273001	1,843	0	1
GSPATT00002077001	-0,063	0,72874	0	GSPATT00021274001	1,19	0,00002	1
GSPATT00002091001	0,425	0,05442	0	GSPATT00021275001	1,322	0,00075	1
GSPATT00002093001	1,052	0,00126	1	GSPATT00021294001	-0,03	0,86443	0
GSPATT00002094001	-0,116	0,53723	0	GSPATT00021304001	-0,938	0,00125	0
GSPATT00002107001	-0,282	0,23525	0	GSPATT00021337001	1,12	0,00026	1
GSPATT00002108001	0,114	0,44232	0	GSPATT00021346001	0,927	0,00175	0
GSPATT00002114001	0,469	0,04573	0	GSPATT00021382001	0,681	0,00268	0
GSPATT00002115001	0,601	0,02057	0	GSPATT00021397001	-0,341	0,04105	0
GSPATT00002129001	1,065	0,00431	1	GSPATT00021403001	0,008	0,96802	0
GSPATT00002132001	0,587	0,01286	0	GSPATT00021407001	0,546	0,01042	0
GSPATT00002156001	0,933	0,0007	0	GSPATT00021410001	0,546	0,03562	0
GSPATT00002182001	1,186	0,00008	1	GSPATT00021416001	0,262	0,11135	0
GSPATT00002186001	0,08	0,52966	0	GSPATT00021423001	0,41	0,01616	0
GSPATT00002191001	1,024	0,00219	1	GSPATT00021496001	-0,121	0,56931	0
GSPATT00002195001	-0,541	0,01959	0	GSPATT00021511001	0,607	0,01408	0
GSPATT00002221001	0,238	0,09743	0	GSPATT00021522001	0,222	0,20683	0
GSPATT00002241001	-0,266	0,23687	0	GSPATT00021531001	1,595	0	1
GSPATT00002247001	0,656	0,00298	0	GSPATT00021532001	0,469	0,01357	0
GSPATT00002269001	-2,667	0,00001	-1	GSPATT00021547001	0,33	0,05213	0
GSPATT00002272001	0,482	0,00978	0	GSPATT00021550001	0,69	0,00115	0
GSPATT00002284001	0,447	0,0176	0	GSPATT00021566001	0,15	0,38869	0
GSPATT00002285001	0,846	0,02305	0	GSPATT00021592001	0,574	0,00334	0
GSPATT00002297001	0,322	0,10354	0	GSPATT00021605001	-0,285	0,07981	0
GSPATT00002321001	0,196	0,2005	0	GSPATT00021623001	-0,006	0,97564	0
GSPATT00002371001	1,279	0,00502	1	GSPATT00021625001	-0,609	0,00663	0
GSPATT00002396001	0,89	0,00446	0	GSPATT00021637001	0,858	0,0012	0
GSPATT00002397001	0,65	0,00105	0	GSPATT00021639001	1,696	0,00085	1
GSPATT00002398001	0,608	0,00382	0	GSPATT00021643001	0,057	0,70009	0
GSPATT00002410001	0,268	0,0869	0	GSPATT00021645001	0,312	0,06296	0
GSPATT00002424001	-0,521	0,01826	0	GSPATT00021666001	-0,927	0,0001	0
GSPATT00002429001	0,235	0,11218	0	GSPATT00021705001	0,593	0,01376	0
GSPATT00002438001	0,165	0,29594	0	GSPATT00021709001	1,146	0,00013	1
GSPATT00002465001	0,13	0,62332	0	GSPATT00021737001	2,607	0,00001	1
GSPATT00002480001	1,183	0,00004	1	GSPATT00021745001	-0,479	0,00827	0
GSPATT00002495001	0,174	0,40222	0	GSPATT00021748001	0,772	0,0086	0
GSPATT00002529001	1,935	0	1	GSPATT00021749001	1,147	0,01618	1
GSPATT00002550001	-0,042	0,75069	0	GSPATT00021752001	0,314	0,06862	0
GSPATT00002553001	0,322	0,07357	0	GSPATT00021753001	0,418	0,07228	0
GSPATT00002575001	0,758	0,01395	0	GSPATT00021754001	0,243	0,14094	0
GSPATT00002585001	0,436	0,08223	0	GSPATT00021761001	0,639	0,00204	0
GSPATT00002589001	0,394	0,03041	0	GSPATT00021763001	1,74	0,00004	1
GSPATT00002593001	1,076	0,0001	1	GSPATT00021786001	0,144	0,35607	0
GSPATT00002595001	0,321	0,0482	0	GSPATT00021789001	1,156	0,00096	1
GSPATT00002614001	-0,048	0,8212	0	GSPATT00021797001	0,664	0,0087	0
GSPATT00002618001	0,222	0,2786	0	GSPATT00021815001	0,094	0,7025	0
GSPATT00002633001	0,209	0,22321	0	GSPATT00021818001	0,452	0,0106	0
GSPATT00002634001	-0,559	0,00848	0	GSPATT00021839001	-0,624	0,00196	0
GSPATT00002648001	-0,019	0,91239	0	GSPATT00021843001	-0,573	0,01408	0
GSPATT00002670001	0,301	0,22471	0	GSPATT00021861001	-2,393	0,00001	-1
GSPATT00002680001	1,006	0,00039	1	GSPATT00021876001	0,606	0,05246	0
GSPATT00002682001	0,464	0,04413	0	GSPATT00021880001	0,418	0,06147	0
GSPATT00002705001	0,629	0,00714	0	GSPATT00021882001	-0,509	0,02829	0
GSPATT00002720001	-1,253	0,00068	-1	GSPATT00021885001	0,508	0,01902	0
GSPATT00002743001	-0,488	0,03306	0	GSPATT00021893001	1,556	0,00001	1
GSPATT00002752001	0,495	0,06805	0	GSPATT00021905001	0,176	0,26244	0
GSPATT00002760001	0,788	0,00112	0	GSPATT00021907001	-0,742	0,0007	0
GSPATT00002771001	0,859	0,0012	0	GSPATT00021930001	-0,548	0,007	0
GSPATT00002776001	-0,859	0,00025	0	GSPATT00021935001	0,444	0,0187	0
GSPATT00002799001	-0,188	0,27826	0	GSPATT00021944001	-1,181	0,00069	-1

GSPATT00002813001	0,189	0,28818	0	GSPATT00021946001	0,249	0,20733	0
GSPATT00002833001	-0,306	0,26468	0	GSPATT00021955001	1,15	0,00027	1
GSPATT00002839001	-0,591	0,0576	0	GSPATT00021964001	0,822	0,01015	0
GSPATT00002865001	-0,375	0,08216	0	GSPATT00021978001	-0,527	0,00915	0
GSPATT00002890001	-1,023	0,00005	-1	GSPATT00021986001	1,819	0,00001	1
GSPATT00002908001	1,161	0,00015	1	GSPATT00022016001	0,205	0,15716	0
GSPATT00002917001	-0,703	0,00355	0	GSPATT00022037001	0,79	0,00181	0
GSPATT00002933001	-2,484	0	-1	GSPATT00022042001	0,34	0,05228	0
GSPATT00002954001	-0,433	0,0165	0	GSPATT00022061001	-0,298	0,11655	0
GSPATT00002960001	-0,423	0,06926	0	GSPATT00022128001	-0,88	0,0016	0
GSPATT00002969001	-0,585	0,02587	0	GSPATT00022131001	-0,083	0,72879	0
GSPATT00002999001	1,266	0,00041	1	GSPATT00022141001	1,027	0,00014	1
GSPATT00003037001	0,383	0,02776	0	GSPATT00022154001	0,346	0,07096	0
GSPATT00003054001	0,076	0,56297	0	GSPATT00022155001	0,511	0,00951	0
GSPATT00003075001	0,629	0,02375	0	GSPATT00022167001	0,329	0,07157	0
GSPATT00003077001	-0,196	0,20764	0	GSPATT00022172001	0,608	0,01725	0
GSPATT00003089001	-0,441	0,11477	0	GSPATT00022183001	-0,244	0,23912	0
GSPATT00003090001	-0,149	0,32277	0	GSPATT00022205001	0,366	0,11152	0
GSPATT00003094001	0,49	0,01226	0	GSPATT00022214001	0,659	0,00671	0
GSPATT00003105001	1,596	0,00013	1	GSPATT00022218001	-0,466	0,01328	0
GSPATT00003123001	0,138	0,52706	0	GSPATT00022236001	1,845	0,00007	1
GSPATT00003132001	0,679	0,00156	0	GSPATT00022240001	0,698	0,0019	0
GSPATT00003154001	0,332	0,08672	0	GSPATT00022249001	0,454	0,00814	0
GSPATT00003156001	0,696	0,00226	0	GSPATT00022251001	-0,514	0,08066	0
GSPATT00003167001	0,084	0,63843	0	GSPATT00022256001	0,89	0,0088	0
GSPATT00003168001	1,73	0,00037	1	GSPATT00022278001	0,113	0,6617	0
GSPATT00003185001	0,267	0,31747	0	GSPATT00022290001	1,407	0,00034	1
GSPATT00003191001	-0,442	0,0623	0	GSPATT00022296001	1,798	0,00001	1
GSPATT00003192001	-0,143	0,32291	0	GSPATT00022304001	0,901	0,00027	0
GSPATT00003194001	1,519	0,00002	1	GSPATT00022316001	0,995	0,0013	0
GSPATT00003198001	0,506	0,02601	0	GSPATT00022319001	-0,149	0,47401	0
GSPATT00003200001	0,421	0,03094	0	GSPATT00022364001	0,012	0,94018	0
GSPATT00003224001	-0,237	0,12454	0	GSPATT00022375001	0,335	0,05369	0
GSPATT00003229001	0,103	0,49036	0	GSPATT00022376001	0,282	0,36231	0
GSPATT00003230001	0,337	0,26038	0	GSPATT00022389001	0,267	0,0861	0
GSPATT00003236001	1,111	0,00005	1	GSPATT00022398001	-0,303	0,11489	0
GSPATT00003248001	-0,663	0,0009	0	GSPATT00022415001	0,64	0,00116	0
GSPATT00003253001	0,103	0,43872	0	GSPATT00022426001	0,639	0,00648	0
GSPATT00003279001	0,867	0,0006	0	GSPATT00022436001	-0,065	0,75967	0
GSPATT00003280001	-0,04	0,80148	0	GSPATT00022478001	-0,264	0,12121	0
GSPATT00003283001	0,264	0,25078	0	GSPATT00022479001	-0,83	0,0106	0
GSPATT00003292001	0,915	0,00467	0	GSPATT00022490001	0,953	0,00048	0
GSPATT00003309001	-0,973	0,0002	0	GSPATT00022560001	0,407	0,01304	0
GSPATT00003326001	-0,051	0,69934	0	GSPATT00022581001	0,197	0,34705	0
GSPATT00003335001	-0,087	0,60586	0	GSPATT00022585001	-0,137	0,38681	0
GSPATT00003339001	0,266	0,15758	0	GSPATT00022588001	0,519	0,01581	0
GSPATT00003344001	0,549	0,00644	0	GSPATT00022625001	-0,631	0,00225	0
GSPATT00003348001	-0,128	0,5085	0	GSPATT00022648001	1,169	0,00006	1
GSPATT00003366001	0,829	0,00229	0	GSPATT00022652001	0,216	0,20385	0
GSPATT00003407001	0,787	0,00042	0	GSPATT00022660001	0,792	0,00748	0
GSPATT00003425001	-0,517	0,01811	0	GSPATT00022665001	0,156	0,24856	0
GSPATT00003486001	0,113	0,47243	0	GSPATT00022666001	1,059	0,00175	1
GSPATT00003501001	0,158	0,34108	0	GSPATT00022671001	0,453	0,06689	0
GSPATT00003507001	-1,565	0,00001	-1	GSPATT00022672001	0,392	0,0156	0
GSPATT00003528001	0,505	0,01812	0	GSPATT00022676001	0,956	0,00118	0
GSPATT00003535001	0,273	0,06967	0	GSPATT00022680001	0,88	0,01013	0
GSPATT00003620001	0,88	0,00103	0	GSPATT00022747001	-0,042	0,84234	0
GSPATT00003647001	0,327	0,21564	0	GSPATT00022758001	-0,264	0,37366	0
GSPATT00003651001	0,62	0,00494	0	GSPATT00022760001	-0,332	0,06229	0
GSPATT00003657001	-0,746	0,00329	0	GSPATT00022772001	0,587	0,00225	0
GSPATT00003674001	0,666	0,18862	0	GSPATT00022794001	-0,642	0,00449	0
GSPATT00003681001	-0,103	0,4827	0	GSPATT00022804001	0,571	0,02711	0
GSPATT00003682001	1,134	0,00047	1	GSPATT00022805001	0,721	0,00384	0
GSPATT00003692001	0,249	0,08305	0	GSPATT00022813001	-0,287	0,25917	0
GSPATT00003699001	-0,208	0,19088	0	GSPATT00022862001	0,196	0,23874	0
GSPATT00003701001	-1,324	0,00003	-1	GSPATT00022923001	0,087	0,54996	0
GSPATT00003711001	-0,057	0,78591	0	GSPATT00022960001	-0,158	0,24845	0

GSPATT00003719001	-2,965	0	-1	GSPATT00022982001	0,79	0,04014	0
GSPATT00003722001	-0,214	0,14285	0	GSPATT00023007001	0,391	0,03401	0
GSPATT00003724001	-1,06	0,00023	-1	GSPATT00023011001	0,775	0,00132	0
GSPATT00003735001	0,938	0,00036	0	GSPATT00023034001	-0,277	0,07151	0
GSPATT00003784001	-0,762	0,00155	0	GSPATT00023053001	0,174	0,19072	0
GSPATT00003800001	0,707	0,00325	0	GSPATT00023054001	0,175	0,21904	0
GSPATT00003805001	-0,458	0,01837	0	GSPATT00023076001	0,772	0,00846	0
GSPATT00003808001	-1,655	0,00001	-1	GSPATT00023086001	0,21	0,20719	0
GSPATT00003833001	0,314	0,13153	0	GSPATT00023101001	0,252	0,21939	0
GSPATT00003834001	0,118	0,39846	0	GSPATT00023116001	0,243	0,2874	0
GSPATT00003866001	0,17	0,22326	0	GSPATT00023168001	-0,346	0,10457	0
GSPATT00003883001	1,07	0,00005	1	GSPATT00023209001	0,505	0,02572	0
GSPATT00003889001	1,021	0,00011	1	GSPATT00023226001	-0,09	0,6793	0
GSPATT00003892001	-0,172	0,33783	0	GSPATT00023238001	-0,52	0,00932	0
GSPATT00003941001	-0,015	0,90753	0	GSPATT00023249001	0,903	0,00029	0
GSPATT00003946001	0,029	0,8323	0	GSPATT00023269001	-0,248	0,34866	0
GSPATT00003963001	0,533	0,01029	0	GSPATT00023282001	-0,358	0,04566	0
GSPATT00003987001	0,889	0,00018	0	GSPATT00023287001	-0,508	0,01292	0
GSPATT00004011001	0,459	0,0181	0	GSPATT00023291001	-0,368	0,10871	0
GSPATT00004041001	-2,104	0,00001	-1	GSPATT00023309001	-0,41	0,01263	0
GSPATT00004054001	0,449	0,01063	0	GSPATT00023311001	0,38	0,03477	0
GSPATT00004057001	-0,473	0,03309	0	GSPATT00023324001	0,902	0,00067	0
GSPATT00004059001	0,026	0,88876	0	GSPATT00023325001	0,851	0,00031	0
GSPATT00004060001	0,495	0,02651	0	GSPATT00023332001	0,059	0,7739	0
GSPATT00004091001	0,299	0,07171	0	GSPATT00023391001	0,729	0,00128	0
GSPATT00004095001	-0,095	0,61368	0	GSPATT00023409001	-0,413	0,17182	0
GSPATT00004114001	0,269	0,09228	0	GSPATT00023417001	0,386	0,08109	0
GSPATT00004132001	-0,103	0,69251	0	GSPATT00023422001	-0,559	0,02664	0
GSPATT00004152001	0,206	0,29595	0	GSPATT00023426001	-0,016	0,92617	0
GSPATT00004205001	-0,31	0,17947	0	GSPATT00023427001	0,645	0,027	0
GSPATT00004210001	0,416	0,03288	0	GSPATT00023429001	0,253	0,18096	0
GSPATT00004223001	0,612	0,00738	0	GSPATT00023436001	0,755	0,04632	0
GSPATT00004225001	0,089	0,74057	0	GSPATT00023438001	0,035	0,83414	0
GSPATT00004229001	-0,224	0,29312	0	GSPATT00023469001	-0,011	0,95408	0
GSPATT00004247001	0,159	0,57122	0	GSPATT00023488001	1,084	0,00116	1
GSPATT00004268001	1,307	0,00011	1	GSPATT00023514001	0,507	0,00757	0
GSPATT00004273001	-0,717	0,00174	0	GSPATT00023531001	0,22	0,46511	0
GSPATT00004298001	0,13	0,50582	0	GSPATT00023540001	0,273	0,10052	0
GSPATT00004321001	0,151	0,6363	0	GSPATT00023549001	-1,224	0,00167	-1
GSPATT00004366001	0,068	0,69186	0	GSPATT00023569001	-0,979	0,00048	0
GSPATT00004368001	0,323	0,16554	0	GSPATT00023571001	-0,22	0,33947	0
GSPATT00004376001	0,343	0,08104	0	GSPATT00023576001	-0,089	0,51115	0
GSPATT00004396001	0,315	0,11278	0	GSPATT00023613001	0,192	0,1796	0
GSPATT00004405001	0,153	0,39785	0	GSPATT00023618001	-0,03	0,8305	0
GSPATT00004412001	-1,59	0,00001	-1	GSPATT00023627001	0,203	0,23093	0
GSPATT00004442001	-0,201	0,48124	0	GSPATT00023631001	0,239	0,10083	0
GSPATT00004447001	0,383	0,05161	0	GSPATT00023632001	0,122	0,36366	0
GSPATT00004496001	0,955	0,00187	0	GSPATT00023676001	0,145	0,31782	0
GSPATT00004514001	0,476	0,02673	0	GSPATT00023690001	-1,078	0,0043	-1
GSPATT00004528001	0,66	0,00397	0	GSPATT00023719001	-0,472	0,04584	0
GSPATT00004559001	0,711	0,00505	0	GSPATT00023728001	0,102	0,45091	0
GSPATT00004570001	0,195	0,22266	0	GSPATT00023729001	0,5	0,071	0
GSPATT00004575001	-0,004	0,97788	0	GSPATT00023735001	0,573	0,00261	0
GSPATT00004580001	1,242	0,00014	1	GSPATT00023790001	-0,309	0,06093	0
GSPATT00004590001	0,499	0,01576	0	GSPATT00023792001	0,035	0,82314	0
GSPATT00004598001	0,48	0,02633	0	GSPATT00023807001	-0,378	0,04035	0
GSPATT00004600001	0,083	0,57831	0	GSPATT00023823001	-0,725	0,00571	0
GSPATT00004601001	1,962	0,0001	1	GSPATT00023843001	0,248	0,16073	0
GSPATT00004610001	0,945	0,00014	0	GSPATT00023861001	1,352	0,00004	1
GSPATT00004630001	-0,933	0,00013	0	GSPATT00023871001	-0,181	0,31269	0
GSPATT00004644001	-2,245	0,00001	-1	GSPATT00023901001	0,661	0,00244	0
GSPATT00004661001	-0,088	0,65447	0	GSPATT00023907001	0,749	0,00073	0
GSPATT00004664001	0,401	0,16244	0	GSPATT00023937001	-0,496	0,07124	0
GSPATT00004681001	-0,284	0,11177	0	GSPATT00023947001	0,21	0,19097	0
GSPATT00004693001	0,654	0,00623	0	GSPATT00023951001	0,462	0,01336	0
GSPATT00004716001	-0,265	0,1283	0	GSPATT00023962001	-0,49	0,01746	0
GSPATT00004725001	-0,044	0,79332	0	GSPATT00023981001	0,056	0,75794	0

GSPATT00004760001	0,235	0,3263	0	GSPATT00024046001	0,488	0,06203	0
GSPATT00004761001	0,808	0,01968	0	GSPATT00024078001	0,879	0,0027	0
GSPATT00004764001	-0,15	0,28228	0	GSPATT00024080001	0,962	0,00084	0
GSPATT00004777001	1,749	0,00006	1	GSPATT00024093001	0,589	0,00369	0
GSPATT00004785001	0,782	0,00521	0	GSPATT00024120001	0,414	0,05081	0
GSPATT00004807001	0,124	0,60039	0	GSPATT00024181001	0,193	0,36401	0
GSPATT00004813001	0,679	0,01555	0	GSPATT00024182001	-0,819	0,01549	0
GSPATT00004832001	-0,277	0,07921	0	GSPATT00024183001	-0,176	0,34939	0
GSPATT00004834001	0,789	0,00098	0	GSPATT00024185001	1,039	0,00005	1
GSPATT00004846001	2,635	0	1	GSPATT00024203001	0,349	0,11648	0
GSPATT00004860001	-0,019	0,91882	0	GSPATT00024205001	0,66	0,00203	0
GSPATT00004865001	-0,645	0,01547	0	GSPATT00024206001	-0,177	0,2138	0
GSPATT00004869001	0,682	0,00693	0	GSPATT00024209001	1,188	0,00003	1
GSPATT00004874001	-0,141	0,55683	0	GSPATT00024218001	-0,321	0,04683	0
GSPATT00004905001	0,24	0,28818	0	GSPATT00024233001	0,949	0,0003	0
GSPATT00004906001	0,041	0,80854	0	GSPATT00024234001	0,863	0,00016	0
GSPATT00004925001	-0,765	0,01316	0	GSPATT00024244001	0,136	0,47521	0
GSPATT00004954001	-0,94	0,02171	0	GSPATT00024253001	0,327	0,12765	0
GSPATT00004968001	0,709	0,00121	0	GSPATT00024274001	0,022	0,87189	0
GSPATT00004976001	-0,023	0,86985	0	GSPATT00024309001	-0,157	0,28831	0
GSPATT00004988001	-0,207	0,1401	0	GSPATT00024335001	-0,34	0,04551	0
GSPATT00004998001	0,877	0,0004	0	GSPATT00024337001	0,437	0,02363	0
GSPATT00005003001	0,289	0,06439	0	GSPATT00024348001	-0,075	0,56089	0
GSPATT00005007001	0,56	0,00815	0	GSPATT00024349001	0,096	0,597	0
GSPATT00005009001	0,54	0,00742	0	GSPATT00024352001	0,614	0,00859	0
GSPATT00005077001	0,271	0,1489	0	GSPATT00024357001	0,148	0,45854	0
GSPATT00005093001	0,879	0,00087	0	GSPATT00024377001	1,043	0,00342	1
GSPATT00005095001	0,782	0,00596	0	GSPATT00024390001	-0,282	0,13976	0
GSPATT00005100001	-0,27	0,06839	0	GSPATT00024398001	0,796	0,01758	0
GSPATT00005117001	0,136	0,40124	0	GSPATT00024410001	0,428	0,02489	0
GSPATT00005120001	0,8	0,00223	0	GSPATT00024418001	0,221	0,13987	0
GSPATT00005130001	-0,006	0,97738	0	GSPATT00024424001	-0,29	0,0846	0
GSPATT00005209001	1,585	0,00011	1	GSPATT00024429001	0,278	0,07692	0
GSPATT00005243001	1,145	0,0018	1	GSPATT00024448001	-0,278	0,17393	0
GSPATT00005245001	0,407	0,07978	0	GSPATT00024451001	0,393	0,03105	0
GSPATT00005247001	-0,102	0,43462	0	GSPATT00024479001	-0,903	0,00303	0
GSPATT00005248001	-1,692	0,00037	-1	GSPATT00024481001	0,435	0,1006	0
GSPATT00005269001	0,265	0,06576	0	GSPATT00024498001	0,272	0,35558	0
GSPATT00005271001	-0,185	0,35608	0	GSPATT00024499001	0,56	0,00549	0
GSPATT00005274001	0,558	0,00601	0	GSPATT00024504001	-0,557	0,06748	0
GSPATT00005284001	0,206	0,14532	0	GSPATT00024512001	1,298	0,00001	1
GSPATT00005295001	0,7	0,00631	0	GSPATT00024514001	0,748	0,01065	0
GSPATT00005301001	0,612	0,02995	0	GSPATT00024536001	0,01	0,96698	0
GSPATT00005326001	1,016	0,00028	1	GSPATT00024539001	-2,028	0	-1
GSPATT00005329001	0,523	0,01314	0	GSPATT00024546001	-0,401	0,04144	0
GSPATT00005343001	-0,049	0,73244	0	GSPATT00024564001	0,433	0,05933	0
GSPATT00005359001	-0,071	0,70866	0	GSPATT00024565001	1,115	0,00008	1
GSPATT00005382001	0,315	0,08827	0	GSPATT00024566001	0,629	0,00565	0
GSPATT00005394001	-2,476	0	-1	GSPATT00024591001	0,498	0,00429	0
GSPATT00005416001	-0,283	0,0906	0	GSPATT00024618001	0,097	0,49798	0
GSPATT00005443001	1,201	0,01436	1	GSPATT00024644001	0,654	0,00896	0
GSPATT00005458001	-0,786	0,00076	0	GSPATT00024673001	-0,186	0,23745	0
GSPATT00005498001	0,586	0,00534	0	GSPATT00024691001	1,052	0,00127	1
GSPATT00005504001	-0,323	0,07461	0	GSPATT00024731001	0,285	0,24363	0
GSPATT00005531001	0,024	0,88617	0	GSPATT00024737001	0,421	0,14567	0
GSPATT00005533001	-0,809	0,00171	0	GSPATT00024766001	0,352	0,11206	0
GSPATT00005536001	0,887	0,00254	0	GSPATT00024788001	0,153	0,34111	0
GSPATT00005555001	0,146	0,36762	0	GSPATT00024859001	1,158	0,00272	1
GSPATT00005556001	0,344	0,07104	0	GSPATT00024862001	0,232	0,28545	0
GSPATT00005557001	0,873	0,00334	0	GSPATT00024885001	-1,562	0,00004	-1
GSPATT00005568001	0,44	0,03873	0	GSPATT00024889001	0,107	0,58905	0
GSPATT00005581001	0,156	0,32591	0	GSPATT00024895001	-0,166	0,43815	0
GSPATT00005587001	0,343	0,06896	0	GSPATT00024913001	-0,542	0,01406	0
GSPATT00005611001	0,102	0,61679	0	GSPATT00024916001	1,07	0,00006	1
GSPATT00005647001	-0,353	0,0216	0	GSPATT00024917001	1,027	0,0002	1
GSPATT00005692001	-0,035	0,79461	0	GSPATT00024942001	-0,918	0,00043	0
GSPATT00005703001	0,426	0,05301	0	GSPATT00024976001	0,84	0,00119	0

GSPATT00005726001	0,372	0,0779	0	GSPATT00024990001	0,807	0,0027	0
GSPATT00005733001	0,017	0,92434	0	GSPATT00025030001	-0,48	0,01979	0
GSPATT00005734001	0,285	0,09036	0	GSPATT00025035001	1,087	0,00028	1
GSPATT00005738001	0,978	0,00023	0	GSPATT00025044001	0,229	0,26111	0
GSPATT00005752001	1,537	0,00004	1	GSPATT00025071001	-0,063	0,7592	0
GSPATT00005757001	0,742	0,00204	0	GSPATT00025076001	0,189	0,30656	0
GSPATT00005760001	-0,591	0,07206	0	GSPATT00025088001	0,087	0,7199	0
GSPATT00005782001	0,537	0,00527	0	GSPATT00025089001	0,396	0,07801	0
GSPATT00005804001	-2,483	0,00045	-1	GSPATT00025098001	0,473	0,02724	0
GSPATT00005811001	0,498	0,03959	0	GSPATT00025106001	0,702	0,01194	0
GSPATT00005814001	-0,041	0,82667	0	GSPATT00025128001	0,313	0,11606	0
GSPATT00005845001	0,153	0,32908	0	GSPATT00025133001	0,871	0,00255	0
GSPATT00005881001	0,784	0,01296	0	GSPATT00025144001	1,216	0,00018	1
GSPATT00005902001	1,463	0,00021	1	GSPATT00025176001	0,858	0,01028	0
GSPATT00005904001	0,174	0,28531	0	GSPATT00025177001	0,141	0,61521	0
GSPATT00005943001	1,376	0,00047	1	GSPATT00025184001	0,545	0,03088	0
GSPATT00005960001	1,544	0,00007	1	GSPATT00025243001	1,014	0,00008	1
GSPATT00005995001	0,249	0,17873	0	GSPATT00025254001	0,909	0,00035	0
GSPATT00006007001	0,509	0,01301	0	GSPATT00025257001	0,582	0,02537	0
GSPATT00006011001	0,426	0,04714	0	GSPATT00025262001	-0,617	0,0118	0
GSPATT00006030001	0,381	0,05068	0	GSPATT00025273001	1,277	0,00074	1
GSPATT00006033001	0,654	0,0075	0	GSPATT00025348001	0,917	0,00082	0
GSPATT00006057001	-0,917	0,00035	0	GSPATT00025382001	-0,664	0,00239	0
GSPATT00006075001	0,152	0,28079	0	GSPATT00025431001	0,45	0,04878	0
GSPATT00006080001	0,359	0,11534	0	GSPATT00025443001	0,397	0,05816	0
GSPATT00006094001	0,938	0,00214	0	GSPATT00025450001	0,01	0,9566	0
GSPATT00006125001	0,447	0,0539	0	GSPATT00025459001	0,443	0,0356	0
GSPATT00006174001	-1,189	0,00097	-1	GSPATT00025471001	-1,355	0,00017	-1
GSPATT00006199001	-0,122	0,43015	0	GSPATT00025473001	1,303	0,00076	1
GSPATT00006226001	-0,062	0,65778	0	GSPATT00025481001	-0,394	0,02039	0
GSPATT00006272001	0,621	0,03899	0	GSPATT00025487001	0,527	0,00492	0
GSPATT00006280001	0,726	0,00083	0	GSPATT00025488001	0,117	0,44687	0
GSPATT00006296001	0,129	0,52836	0	GSPATT00025542001	0,154	0,32238	0
GSPATT00006297001	0,413	0,01659	0	GSPATT00025564001	0,335	0,03391	0
GSPATT00006313001	0,809	0,01243	0	GSPATT00025565001	0,102	0,44835	0
GSPATT00006314001	0,607	0,00237	0	GSPATT00025585001	0,189	0,32528	0
GSPATT00006315001	0,211	0,21775	0	GSPATT00025621001	0,549	0,02246	0
GSPATT00006331001	0,84	0,00169	0	GSPATT00025624001	1,872	0,00011	1
GSPATT00006334001	-0,044	0,80576	0	GSPATT00025626001	0,186	0,22873	0
GSPATT00006378001	0,677	0,00317	0	GSPATT00025634001	-0,572	0,0037	0
GSPATT00006379001	0,429	0,03699	0	GSPATT00025641001	-0,136	0,41303	0
GSPATT00006390001	-3,443	0	-1	GSPATT00025658001	0,311	0,08053	0
GSPATT00006408001	-0,255	0,28163	0	GSPATT00025660001	0,423	0,01588	0
GSPATT00006415001	-0,218	0,29617	0	GSPATT00025674001	-0,467	0,00665	0
GSPATT00006446001	-0,06	0,78799	0	GSPATT00025677001	0,628	0,0033	0
GSPATT00006468001	1,644	0,00007	1	GSPATT00025683001	1,103	0,00903	1
GSPATT00006472001	-0,821	0,00056	0	GSPATT00025688001	0,986	0,00101	0
GSPATT00006519001	1,503	0,00026	1	GSPATT00025714001	-0,285	0,11387	0
GSPATT00006522001	0,067	0,61368	0	GSPATT00025724001	-0,118	0,54796	0
GSPATT00006526001	0,784	0,00476	0	GSPATT00025742001	0,095	0,63776	0
GSPATT00006544001	0,253	0,22418	0	GSPATT00025754001	1,693	0	1
GSPATT00006575001	-0,075	0,7515	0	GSPATT00025790001	0,722	0,01137	0
GSPATT00006576001	0,119	0,43904	0	GSPATT00025829001	-0,303	0,13502	0
GSPATT00006580001	0,511	0,15256	0	GSPATT00025830001	-0,219	0,47777	0
GSPATT00006581001	-0,025	0,86132	0	GSPATT00025833001	0,318	0,12793	0
GSPATT00006592001	0,443	0,14222	0	GSPATT00025835001	0,577	0,00646	0
GSPATT00006594001	2,357	0	1	GSPATT00025843001	-0,234	0,1386	0
GSPATT00006615001	0,212	0,32587	0	GSPATT00025874001	0,078	0,61912	0
GSPATT00006642001	1,159	0,00005	1	GSPATT00025891001	0,036	0,78724	0
GSPATT00006653001	0,808	0,00058	0	GSPATT00025892001	0,813	0,00112	0
GSPATT00006659001	-0,504	0,0224	0	GSPATT00025893001	0,184	0,26595	0
GSPATT00006681001	1,225	0,00002	1	GSPATT00025925001	-2,389	0,00001	-1
GSPATT00006690001	0,625	0,00374	0	GSPATT00025938001	0,244	0,21258	0
GSPATT00006702001	1,003	0,00049	1	GSPATT00025977001	0,266	0,20896	0
GSPATT00006707001	0,096	0,55283	0	GSPATT00025987001	0,777	0,00304	0
GSPATT00006718001	-1,206	0,0008	-1	GSPATT00025999001	0,214	0,18023	0
GSPATT00006721001	0,259	0,28365	0	GSPATT00026028001	-0,597	0,02174	0

GSPATT00006725001	1,26	0,00008	1	GSPATT00026044001	1,043	0,00011	1
GSPATT00006735001	1,208	0,00017	1	GSPATT00026048001	0,131	0,45105	0
GSPATT00006746001	0,845	0,00197	0	GSPATT00026052001	0,22	0,26869	0
GSPATT00006769001	0,213	0,32339	0	GSPATT00026100001	-0,727	0,05157	0
GSPATT00006775001	0,248	0,09259	0	GSPATT00026117001	0,711	0,00252	0
GSPATT00006782001	0,148	0,27067	0	GSPATT00026130001	0,041	0,84926	0
GSPATT00006852001	0,834	0,00426	0	GSPATT00026158001	-0,019	0,89138	0
GSPATT00006853001	0,531	0,04301	0	GSPATT00026167001	0,538	0,00916	0
GSPATT00006854001	0,961	0,00034	0	GSPATT00026169001	0,593	0,0211	0
GSPATT00006864001	0,6	0,00924	0	GSPATT00026182001	0,405	0,01463	0
GSPATT00006884001	0,278	0,14598	0	GSPATT00026208001	-0,305	0,05561	0
GSPATT00006904001	0,29	0,11816	0	GSPATT00026214001	0,126	0,51347	0
GSPATT00006927001	0,501	0,00669	0	GSPATT00026231001	-0,721	0,00236	0
GSPATT00006928001	0,334	0,0274	0	GSPATT00026239001	0,204	0,24495	0
GSPATT00006948001	0,353	0,03894	0	GSPATT00026269001	-0,827	0,00039	0
GSPATT00006949001	0,568	0,03228	0	GSPATT00026274001	0,081	0,55749	0
GSPATT00006953001	0,189	0,35123	0	GSPATT00026292001	0,175	0,24434	0
GSPATT00006974001	0,738	0,04036	0	GSPATT00026323001	-0,937	0,00078	0
GSPATT00007015001	-0,04	0,8039	0	GSPATT00026336001	-0,538	0,01977	0
GSPATT00007020001	0,699	0,0179	0	GSPATT00026360001	1,582	0,00001	1
GSPATT00007023001	0,031	0,83742	0	GSPATT00026362001	0,039	0,7821	0
GSPATT00007028001	1,888	0,00035	1	GSPATT00026366001	0,255	0,08585	0
GSPATT00007036001	-1,628	0,00205	-1	GSPATT00026371001	0,968	0,00219	0
GSPATT00007037001	0,21	0,21843	0	GSPATT00026384001	0,13	0,49373	0
GSPATT00007044001	-0,041	0,75732	0	GSPATT00026401001	0,404	0,01576	0
GSPATT00007068001	-0,215	0,22845	0	GSPATT00026410001	-1,354	0,00004	-1
GSPATT00007081001	-0,453	0,09098	0	GSPATT00026424001	-0,195	0,41235	0
GSPATT00007084001	1,343	0,0001	1	GSPATT00026435001	0,978	0,0003	0
GSPATT00007085001	0,898	0,00027	0	GSPATT00026456001	-0,112	0,49348	0
GSPATT00007086001	-0,397	0,12088	0	GSPATT00026471001	0,689	0,0725	0
GSPATT00007091001	1,465	0,00001	1	GSPATT00026484001	0,101	0,56577	0
GSPATT00007092001	0,27	0,19194	0	GSPATT00026498001	0,273	0,11056	0
GSPATT00007095001	0,892	0,00182	0	GSPATT00026501001	0,573	0,02563	0
GSPATT00007110001	0,57	0,00537	0	GSPATT00026528001	0,335	0,1504	0
GSPATT00007113001	0,035	0,79386	0	GSPATT00026582001	-0,61	0,00172	0
GSPATT00007130001	0,976	0,0003	0	GSPATT00026598001	-0,409	0,02421	0
GSPATT00007151001	0,29	0,08359	0	GSPATT00026607001	0,518	0,00768	0
GSPATT00007162001	-0,046	0,76877	0	GSPATT00026611001	0,416	0,09592	0
GSPATT00007168001	2,307	0	1	GSPATT00026620001	-0,023	0,89953	0
GSPATT00007170001	0,256	0,39221	0	GSPATT00026642001	0,375	0,12827	0
GSPATT00007176001	0,545	0,0517	0	GSPATT00026656001	0,014	0,92729	0
GSPATT00007193001	-0,121	0,39214	0	GSPATT00026672001	0,656	0,00939	0
GSPATT00007196001	0,471	0,05051	0	GSPATT00026673001	1,475	0,00074	1
GSPATT00007206001	-0,243	0,15376	0	GSPATT00026685001	-0,162	0,49003	0
GSPATT00007221001	-0,047	0,73213	0	GSPATT00026711001	-1,004	0,00014	-1
GSPATT00007228001	-0,424	0,07621	0	GSPATT00026728001	0,421	0,03621	0
GSPATT00007288001	-0,479	0,02516	0	GSPATT00026733001	-0,183	0,2048	0
GSPATT00007295001	1,711	0	1	GSPATT00026736001	0,014	0,9335	0
GSPATT00007306001	0,194	0,49349	0	GSPATT00026737001	-0,589	0,01656	0
GSPATT00007307001	0,539	0,00329	0	GSPATT00026742001	1,681	0,00062	1
GSPATT00007337001	0,377	0,05996	0	GSPATT00026751001	0,217	0,23484	0
GSPATT00007343001	0,329	0,28753	0	GSPATT00026762001	0,081	0,72109	0
GSPATT00007357001	-0,154	0,51852	0	GSPATT00026768001	1,3	0,0016	1
GSPATT00007369001	-0,3	0,09662	0	GSPATT00026770001	0,781	0,00036	0
GSPATT00007385001	0,031	0,87634	0	GSPATT00026784001	0,447	0,02348	0
GSPATT00007396001	0,747	0,00516	0	GSPATT00026817001	-0,512	0,01273	0
GSPATT00007424001	0,679	0,0528	0	GSPATT00026853001	-0,021	0,90542	0
GSPATT00007458001	-0,178	0,41913	0	GSPATT00026855001	-0,189	0,23205	0
GSPATT00007469001	0,74	0,00278	0	GSPATT00026879001	0,638	0,0089	0
GSPATT00007491001	0,204	0,26477	0	GSPATT00026882001	0,229	0,14149	0
GSPATT00007506001	3,323	0	1	GSPATT00026903001	0,385	0,04693	0
GSPATT00007552001	-0,289	0,16464	0	GSPATT00026918001	0,426	0,01186	0
GSPATT00007569001	-0,059	0,72729	0	GSPATT00026931001	0,327	0,03994	0
GSPATT00007633001	0,585	0,00705	0	GSPATT00026977001	0,418	0,01985	0
GSPATT00007636001	0,951	0,00013	0	GSPATT00027082001	-0,14	0,42006	0
GSPATT00007651001	1,374	0,00001	1	GSPATT00027084001	-2,917	0	-1
GSPATT00007652001	0,604	0,00281	0	GSPATT00027094001	-0,238	0,23457	0

GSPATT00007655001	1,115	0,00032	1
GSPATT00007660001	-2,547	0	-1
GSPATT00007679001	-0,046	0,77776	0
GSPATT00007689001	0,756	0,0049	0
GSPATT00007699001	0,698	0,00069	0
GSPATT00007792001	0,379	0,04772	0
GSPATT00007797001	0,346	0,07773	0
GSPATT00007801001	0,358	0,05841	0
GSPATT00007808001	0,931	0,00109	0
GSPATT00007826001	0,485	0,01651	0
GSPATT00007852001	0,616	0,00193	0
GSPATT00007857001	1,41	0,00014	1
GSPATT00007860001	0,07	0,62078	0
GSPATT00007861001	0,494	0,03195	0
GSPATT00007899001	0,019	0,90526	0
GSPATT00007911001	-0,929	0,02523	0
GSPATT00007917001	0,096	0,67711	0
GSPATT00007919001	0,035	0,87791	0
GSPATT00007922001	0,835	0,01888	0
GSPATT00007930001	-0,448	0,05141	0
GSPATT00007946001	1,164	0,00002	1
GSPATT00007947001	0,256	0,17572	0
GSPATT00007949001	1,323	0,00004	1
GSPATT00007953001	0,179	0,38295	0
GSPATT00007957001	0,151	0,34529	0
GSPATT00007961001	-0,294	0,05705	0
GSPATT00007964001	-2,161	0,00001	-1
GSPATT00007966001	0,728	0,03307	0
GSPATT00007989001	0,044	0,84104	0
GSPATT00007997001	0,533	0,00985	0
GSPATT00007998001	0,219	0,19699	0
GSPATT00008011001	0,371	0,20891	0
GSPATT00008014001	0,284	0,11593	0
GSPATT00008031001	-0,45	0,02946	0
GSPATT00008074001	-0,457	0,03281	0
GSPATT00008104001	1,297	0,00002	1
GSPATT00008134001	0,932	0,00055	0
GSPATT00008150001	0,063	0,64187	0
GSPATT00008151001	0,2	0,21222	0
GSPATT00008165001	0,565	0,00488	0
GSPATT00008178001	0,755	0,00688	0
GSPATT00008186001	-0,324	0,05436	0
GSPATT00008216001	0,518	0,06627	0
GSPATT00008228001	1,643	0,00002	1
GSPATT00008231001	-0,275	0,07499	0
GSPATT00008235001	-0,069	0,63056	0
GSPATT00008243001	0,87	0,00126	0
GSPATT00008313001	-3,997	0	-1
GSPATT00008318001	0,769	0,02343	0
GSPATT00008324001	0,184	0,37151	0
GSPATT00008331001	0,617	0,11575	0
GSPATT00008341001	0,816	0,00047	0
GSPATT00008356001	0,219	0,14265	0
GSPATT00008371001	0,43	0,01968	0
GSPATT00008373001	0,504	0,0091	0
GSPATT00008385001	0,841	0,01833	0
GSPATT00008408001	-0,744	0,00043	0
GSPATT00008410001	0,894	0,00015	0
GSPATT00008418001	-0,272	0,07464	0
GSPATT00008447001	-0,614	0,00381	0
GSPATT00008463001	1,266	0,00003	1
GSPATT00008472001	0,504	0,00647	0
GSPATT00008497001	0,172	0,39591	0
GSPATT00008503001	0,019	0,92472	0
GSPATT00008527001	0,18	0,35417	0
GSPATT00008573001	-0,003	0,98576	0
GSPATT00008596001	-1,074	0,00006	-1
GSPATT00027100001	0,499	0,06172	0
GSPATT00027119001	0,768	0,00101	0
GSPATT00027122001	-0,502	0,01341	0
GSPATT00027138001	0,18	0,19509	0
GSPATT00027147001	-0,082	0,6483	0
GSPATT00027149001	0,147	0,32384	0
GSPATT00027156001	0,039	0,76929	0
GSPATT00027189001	0,049	0,79446	0
GSPATT00027203001	0,063	0,64969	0
GSPATT00027205001	-0,221	0,24032	0
GSPATT00027222001	0,074	0,59482	0
GSPATT00027241001	0,321	0,05768	0
GSPATT00027257001	0,806	0,00465	0
GSPATT00027270001	0,745	0,00887	0
GSPATT00027312001	-1,048	0,00047	-1
GSPATT00027355001	-0,308	0,10498	0
GSPATT00027372001	1,25	0,00004	1
GSPATT00027401001	0,365	0,07066	0
GSPATT00027404001	-0,378	0,02671	0
GSPATT00027408001	0,032	0,82286	0
GSPATT00027410001	0,523	0,0081	0
GSPATT00027442001	-0,097	0,65217	0
GSPATT00027453001	-0,38	0,0322	0
GSPATT00027465001	-1,097	0,00007	-1
GSPATT00027479001	0,392	0,10569	0
GSPATT00027483001	0,725	0,00101	0
GSPATT00027492001	1,241	0,00093	1
GSPATT00027506001	0,183	0,46769	0
GSPATT00027520001	-0,336	0,16994	0
GSPATT00027535001	0,825	0,01036	0
GSPATT00027546001	-0,383	0,03735	0
GSPATT00027549001	0,322	0,09184	0
GSPATT00027607001	0,43	0,06985	0
GSPATT00027613001	-0,299	0,09538	0
GSPATT00027617001	-0,374	0,02379	0
GSPATT00027623001	0,439	0,01037	0
GSPATT00027637001	0,397	0,02895	0
GSPATT00027641001	1,114	0,00049	1
GSPATT00027648001	-0,304	0,04309	0
GSPATT00027665001	0,667	0,04913	0
GSPATT00027672001	-0,668	0,01828	0
GSPATT00027677001	0,078	0,65055	0
GSPATT00027685001	0,812	0,00085	0
GSPATT00027690001	0,646	0,00592	0
GSPATT00027700001	0,396	0,14503	0
GSPATT00027727001	0,419	0,06587	0
GSPATT00027731001	-0,101	0,74111	0
GSPATT00027740001	0,305	0,04752	0
GSPATT00027741001	0,59	0,00929	0
GSPATT00027763001	0,328	0,03342	0
GSPATT00027787001	0,369	0,04914	0
GSPATT00027796001	-1,461	0,0001	-1
GSPATT00027807001	-2	0	-1
GSPATT00027838001	0,488	0,03197	0
GSPATT00027852001	-1,233	0,00015	-1
GSPATT00027857001	0,522	0,0062	0
GSPATT00027870001	-0,002	0,9924	0
GSPATT00027898001	0,118	0,48905	0
GSPATT00027906001	0,065	0,67546	0
GSPATT00027913001	0,404	0,09183	0
GSPATT00027933001	-0,886	0,00125	0
GSPATT00027938001	0,112	0,47766	0
GSPATT00027978001	-0,102	0,58439	0
GSPATT00027980001	-0,117	0,44254	0
GSPATT00028006001	0,033	0,84856	0
GSPATT00028029001	1,144	0,00006	1
GSPATT00028033001	0,014	0,94972	0

GSPATT00008602001	0,371	0,02113	0	GSPATT00028035001	1,108	0,00038	1
GSPATT00008611001	0,235	0,3889	0	GSPATT00028058001	-1,581	0,00253	-1
GSPATT00008619001	-0,021	0,87621	0	GSPATT00028068001	0,819	0,00613	0
GSPATT00008639001	-0,663	0,00927	0	GSPATT00028082001	0,851	0,00023	0
GSPATT00008645001	0,599	0,05259	0	GSPATT00028088001	0,595	0,00642	0
GSPATT00008738001	0,821	0,00201	0	GSPATT00028112001	0,016	0,92503	0
GSPATT00008749001	-0,136	0,49358	0	GSPATT00028130001	0,382	0,0628	0
GSPATT00008764001	0,257	0,19576	0	GSPATT00028137001	-0,328	0,07792	0
GSPATT00008778001	0,342	0,04202	0	GSPATT00028156001	0,053	0,70232	0
GSPATT00008805001	0,579	0,00784	0	GSPATT00028162001	0,316	0,14219	0
GSPATT00008830001	0,301	0,18862	0	GSPATT00028174001	-0,017	0,90512	0
GSPATT00008831001	0,097	0,54219	0	GSPATT00028181001	0,307	0,0498	0
GSPATT00008850001	0,887	0,00048	0	GSPATT00028199001	1,181	0,00015	1
GSPATT00008866001	0,383	0,07319	0	GSPATT00028210001	-0,239	0,19183	0
GSPATT00008880001	-0,665	0,00165	0	GSPATT00028223001	0,071	0,6272	0
GSPATT00008893001	0,303	0,07004	0	GSPATT00028224001	-0,142	0,45435	0
GSPATT00008897001	0,334	0,19577	0	GSPATT00028226001	-0,224	0,18937	0
GSPATT00008901001	-1,237	0,00007	-1	GSPATT00028254001	0,074	0,68566	0
GSPATT00008924001	0,154	0,39308	0	GSPATT00028260001	0,201	0,33982	0
GSPATT00008975001	0,694	0,00205	0	GSPATT00028261001	0,648	0,00753	0
GSPATT00008986001	0,293	0,16667	0	GSPATT00028264001	-0,912	0,00051	0
GSPATT00009001001	0,558	0,04588	0	GSPATT00028265001	-0,664	0,00186	0
GSPATT00009009001	-0,53	0,00588	0	GSPATT00028266001	-0,643	0,01703	0
GSPATT00009013001	1,033	0,00013	1	GSPATT00028267001	1,612	0,00001	1
GSPATT00009076001	0,364	0,0251	0	GSPATT00028269001	0,88	0,00108	0
GSPATT00009086001	0,586	0,00945	0	GSPATT00028305001	0,264	0,18486	0
GSPATT00009091001	-2,067	0,00001	-1	GSPATT00028307001	-0,082	0,66338	0
GSPATT00009102001	0,179	0,23283	0	GSPATT00028312001	0,636	0,00684	0
GSPATT00009110001	0,013	0,95996	0	GSPATT00028368001	0,41	0,03475	0
GSPATT00009123001	-0,164	0,60057	0	GSPATT00028374001	0,68	0,00629	0
GSPATT00009138001	1,521	0,00028	1	GSPATT00028387001	-0,687	0,00975	0
GSPATT00009146001	-0,481	0,05125	0	GSPATT00028405001	0,115	0,6059	0
GSPATT00009149001	0,025	0,87197	0	GSPATT00028413001	0,051	0,72041	0
GSPATT00009155001	-0,238	0,12312	0	GSPATT00028458001	-1,195	0,00003	-1
GSPATT00009172001	-1,485	0,00015	-1	GSPATT00028463001	0,348	0,16251	0
GSPATT00009173001	0,244	0,23213	0	GSPATT00028502001	0,619	0,01367	0
GSPATT00009174001	0,724	0,00457	0	GSPATT00028521001	-0,347	0,06932	0
GSPATT00009176001	0,333	0,1816	0	GSPATT00028522001	0,518	0,02311	0
GSPATT00009191001	0,221	0,13878	0	GSPATT00028523001	-0,194	0,30675	0
GSPATT00009207001	1,429	0,00002	1	GSPATT00028553001	-1,461	0,00001	-1
GSPATT00009211001	1,961	0	1	GSPATT00028591001	-0,079	0,66532	0
GSPATT00009267001	-1,116	0,00261	-1	GSPATT00028640001	-0,009	0,94942	0
GSPATT00009271001	-0,931	0,00059	0	GSPATT00028664001	1,165	0,00037	1
GSPATT00009344001	-0,02	0,89901	0	GSPATT00028690001	0,455	0,03584	0
GSPATT00009346001	0,505	0,00751	0	GSPATT00028737001	1,259	0,00014	1
GSPATT00009355001	-0,135	0,39547	0	GSPATT00028739001	0,281	0,07289	0
GSPATT00009389001	0,729	0,00072	0	GSPATT00028760001	-0,016	0,95354	0
GSPATT00009411001	0,367	0,14277	0	GSPATT00028761001	-0,584	0,00278	0
GSPATT00009414001	1,265	0,00079	1	GSPATT00028762001	0,71	0,00514	0
GSPATT00009428001	0,129	0,62268	0	GSPATT00028795001	-0,564	0,00507	0
GSPATT00009429001	-0,821	0,00035	0	GSPATT00028814001	0,718	0,00439	0
GSPATT00009449001	0,122	0,41636	0	GSPATT00028854001	0,383	0,0558	0
GSPATT00009461001	-1,129	0,00293	-1	GSPATT00028862001	0,155	0,44362	0
GSPATT00009466001	0,566	0,00692	0	GSPATT00028879001	-0,101	0,52631	0
GSPATT00009502001	-0,497	0,00886	0	GSPATT00028902001	0,115	0,49398	0
GSPATT00009512001	0,817	0,00579	0	GSPATT00028907001	0,31	0,07543	0
GSPATT00009529001	-0,262	0,0797	0	GSPATT00028913001	0,627	0,00447	0
GSPATT00009531001	0,045	0,73938	0	GSPATT00028920001	0,862	0,00104	0
GSPATT00009539001	-0,64	0,00235	0	GSPATT00028924001	0,504	0,00498	0
GSPATT00009552001	-0,641	0,01566	0	GSPATT00028949001	0,858	0,01717	0
GSPATT00009559001	0,264	0,18251	0	GSPATT00028981001	0,411	0,07642	0
GSPATT00009580001	0,2	0,21252	0	GSPATT00029004001	-0,194	0,28836	0
GSPATT00009588001	0,994	0,04353	0	GSPATT00029007001	0,785	0,00492	0
GSPATT00009601001	-0,457	0,11601	0	GSPATT00029023001	0,516	0,03057	0
GSPATT00009602001	0,93	0,0008	0	GSPATT00029041001	0,57	0,00776	0
GSPATT00009607001	0,735	0,01843	0	GSPATT00029053001	-0,652	0,01479	0
GSPATT00009626001	0,037	0,77284	0	GSPATT00029067001	-0,083	0,53645	0

GSPATT00009643001	0,405	0,01362	0	GSPATT00029101001	-0,869	0,00393	0
GSPATT00009645001	0,973	0,00151	0	GSPATT00029164001	0,564	0,12006	0
GSPATT00009646001	0,589	0,00229	0	GSPATT00029175001	-0,791	0,00029	0
GSPATT00009693001	0,347	0,0372	0	GSPATT00029187001	0,682	0,01155	0
GSPATT00009703001	-2,787	0	-1	GSPATT00029196001	2,188	0,00004	1
GSPATT00009718001	-0,819	0,00093	0	GSPATT00029198001	0,416	0,04007	0
GSPATT00009748001	0,253	0,1151	0	GSPATT00029217001	0,222	0,14787	0
GSPATT00009760001	-2,631	0	-1	GSPATT00029249001	-0,351	0,02712	0
GSPATT00009772001	-0,026	0,84037	0	GSPATT00029273001	-2,046	0,00011	-1
GSPATT00009782001	-0,119	0,55451	0	GSPATT00029295001	1,454	0,00404	1
GSPATT00009828001	0,419	0,03475	0	GSPATT00029341001	0,262	0,14262	0
GSPATT00009848001	-0,228	0,27077	0	GSPATT00029366001	0,262	0,1057	0
GSPATT00009873001	1,405	0,00002	1	GSPATT00029406001	0,843	0,00341	0
GSPATT00009883001	0,58	0,00174	0	GSPATT00029408001	0,85	0,0034	0
GSPATT00009888001	0,679	0,00449	0	GSPATT00029420001	1,305	0,00003	1
GSPATT00009892001	-0,164	0,50097	0	GSPATT00029443001	-0,601	0,00289	0
GSPATT00009932001	-0,127	0,37952	0	GSPATT00029444001	1,26	0,00076	1
GSPATT00009937001	0,505	0,12425	0	GSPATT00029470001	0,093	0,49861	0
GSPATT00009951001	-0,279	0,21687	0	GSPATT00029472001	0,184	0,20615	0
GSPATT00009964001	0,607	0,03286	0	GSPATT00029530001	0,081	0,70204	0
GSPATT00009969001	0,018	0,89309	0	GSPATT00029552001	0,56	0,01662	0
GSPATT00009980001	0,361	0,01933	0	GSPATT00029556001	-0,933	0,00515	0
GSPATT00009997001	0,66	0,0026	0	GSPATT00029558001	1,707	0,00001	1
GSPATT00010022001	-0,69	0,00171	0	GSPATT00029574001	-1,108	0,00039	-1
GSPATT00010023001	0,56	0,00691	0	GSPATT00029583001	0,198	0,35094	0
GSPATT00010038001	0,125	0,38308	0	GSPATT00029601001	1,502	0,00004	1
GSPATT00010040001	-0,369	0,07302	0	GSPATT00029614001	-0,616	0,00563	0
GSPATT00010043001	0,211	0,26138	0	GSPATT00029618001	0,584	0,00422	0
GSPATT00010044001	0,443	0,07115	0	GSPATT00029627001	-0,199	0,15139	0
GSPATT00010047001	0,376	0,04978	0	GSPATT00029631001	0,75	0,00323	0
GSPATT00010069001	0,096	0,49349	0	GSPATT00029713001	0,817	0,00169	0
GSPATT00010072001	0,597	0,04414	0	GSPATT00029723001	0,372	0,11982	0
GSPATT00010088001	0,474	0,01739	0	GSPATT00029731001	0,613	0,0095	0
GSPATT00010089001	-0,02	0,89788	0	GSPATT00029761001	0,264	0,06412	0
GSPATT00010120001	0,439	0,05835	0	GSPATT00029771001	-0,737	0,0067	0
GSPATT00010165001	1,057	0,05472	0	GSPATT00029778001	-0,608	0,00307	0
GSPATT00010186001	-0,625	0,00879	0	GSPATT00029822001	0,665	0,00641	0
GSPATT00010187001	0,077	0,70848	0	GSPATT00029823001	-0,53	0,02834	0
GSPATT00010189001	0,274	0,09078	0	GSPATT00029824001	0,784	0,05706	0
GSPATT00010192001	-0,341	0,04434	0	GSPATT00029844001	0,11	0,52769	0
GSPATT00010201001	-1,112	0,00403	-1	GSPATT00029848001	0,411	0,08951	0
GSPATT00010217001	0,055	0,68694	0	GSPATT00029901001	0,748	0,00121	0
GSPATT00010238001	0,721	0,00063	0	GSPATT00029929001	1,561	0,00012	1
GSPATT00010240001	1,641	0,00002	1	GSPATT00029968001	0,19	0,2579	0
GSPATT00010241001	-0,059	0,67929	0	GSPATT00029971001	-0,01	0,95787	0
GSPATT00010256001	0,667	0,16041	0	GSPATT00030006001	0,333	0,17202	0
GSPATT00010264001	1,126	0,00022	1	GSPATT00030011001	0,244	0,31877	0
GSPATT00010267001	0,363	0,04122	0	GSPATT00030052001	-0,257	0,11428	0
GSPATT00010305001	0,583	0,02695	0	GSPATT00030056001	0,096	0,60981	0
GSPATT00010342001	-0,027	0,88113	0	GSPATT00030064001	1,558	0,00015	1
GSPATT00010360001	0,009	0,9481	0	GSPATT00030135001	0,579	0,06809	0
GSPATT00010361001	1,344	0,00002	1	GSPATT00030142001	0,973	0,0012	0
GSPATT00010434001	0,716	0,00843	0	GSPATT00030146001	-0,644	0,00333	0
GSPATT00010455001	2,068	0,00001	1	GSPATT00030175001	-0,442	0,01694	0
GSPATT00010478001	0,755	0,0015	0	GSPATT00030183001	0,09	0,72161	0
GSPATT00010489001	0,856	0,01707	0	GSPATT00030220001	-0,307	0,04731	0
GSPATT00010520001	1,675	0,00001	1	GSPATT00030230001	0,126	0,43629	0
GSPATT00010535001	0,291	0,15153	0	GSPATT00030249001	1,907	0	1
GSPATT00010550001	-0,166	0,3917	0	GSPATT00030250001	0,03	0,86055	0
GSPATT00010557001	0,305	0,0402	0	GSPATT00030251001	0,536	0,02526	0
GSPATT00010591001	-0,327	0,06596	0	GSPATT00030268001	0,167	0,24397	0
GSPATT00010597001	0,042	0,75702	0	GSPATT00030295001	0,775	0,00158	0
GSPATT00010599001	-0,541	0,00596	0	GSPATT00030300001	0,307	0,11242	0
GSPATT00010606001	-0,148	0,37763	0	GSPATT00030312001	0,159	0,34993	0
GSPATT00010622001	0,288	0,09844	0	GSPATT00030338001	0,37	0,02763	0
GSPATT00010631001	-0,002	0,99061	0	GSPATT00030339001	0,929	0,00014	0
GSPATT00010634001	-0,099	0,64063	0	GSPATT00030340001	-0,629	0,00193	0

GSPATT00010700001	1,693	0	1	GSPATT00030347001	0,305	0,05631	0
GSPATT00010735001	0,406	0,06251	0	GSPATT00030360001	1,11	0,00034	1
GSPATT00010794001	0,388	0,14585	0	GSPATT00030377001	0,608	0,03137	0
GSPATT00010803001	0,375	0,05126	0	GSPATT00030395001	0,632	0,00363	0
GSPATT00010807001	0,842	0,00505	0	GSPATT00030433001	0,649	0,01931	0
GSPATT00010852001	0,595	0,00207	0	GSPATT00030441001	-1,166	0,00076	-1
GSPATT00010926001	-0,295	0,08518	0	GSPATT00030461001	0,787	0,01769	0
GSPATT00010927001	0,963	0,00093	0	GSPATT00030462001	2,069	0	1
GSPATT00010930001	0,326	0,08283	0	GSPATT00030463001	0,18	0,23178	0
GSPATT00010931001	0,755	0,00409	0	GSPATT00030525001	0,296	0,14492	0
GSPATT00010951001	-0,4	0,08547	0	GSPATT00030559001	1,24	0,00015	1
GSPATT00010982001	1,537	0,00037	1	GSPATT00030602001	0,266	0,22578	0
GSPATT00010994001	-2,479	0	-1	GSPATT00030614001	-0,182	0,32497	0
GSPATT00011004001	0,073	0,58776	0	GSPATT00030636001	0,66	0,0019	0
GSPATT00011094001	-0,248	0,11654	0	GSPATT00030654001	-0,73	0,00085	0
GSPATT00011096001	0,27	0,14138	0	GSPATT00030663001	0,245	0,35912	0
GSPATT00011110001	0,439	0,02922	0	GSPATT00030666001	0,411	0,06139	0
GSPATT00011112001	0,611	0,00704	0	GSPATT00030669001	0,057	0,6915	0
GSPATT00011130001	0,933	0,00615	0	GSPATT00030671001	-0,295	0,30814	0
GSPATT00011132001	-1,165	0,00035	-1	GSPATT00030687001	0,812	0,01438	0
GSPATT00011167001	-0,396	0,02351	0	GSPATT00030688001	-0,02	0,90163	0
GSPATT00011173001	-3,857	0	-1	GSPATT00030698001	0,071	0,70437	0
GSPATT00011179001	0,334	0,11641	0	GSPATT00030723001	-0,18	0,43523	0
GSPATT00011220001	0,315	0,15075	0	GSPATT00030733001	-1,617	0,00013	-1
GSPATT00011221001	1,038	0,00031	1	GSPATT00030745001	-0,1	0,48372	0
GSPATT00011227001	0,852	0,00196	0	GSPATT00030749001	-0,359	0,06814	0
GSPATT00011241001	0,626	0,02469	0	GSPATT00030782001	0,235	0,17113	0
GSPATT00011251001	0,282	0,09853	0	GSPATT00030803001	1,123	0,0001	1
GSPATT00011290001	1,791	0	1	GSPATT00030806001	0,727	0,0015	0
GSPATT00011305001	-0,492	0,0392	0	GSPATT00030818001	-0,245	0,23455	0
GSPATT00011340001	0,056	0,74423	0	GSPATT00030848001	0,031	0,86428	0
GSPATT00011369001	1,426	0,00002	1	GSPATT00030851001	0,263	0,1287	0
GSPATT00011371001	-0,116	0,58125	0	GSPATT00030853001	0,549	0,03624	0
GSPATT00011372001	-0,323	0,11988	0	GSPATT00030855001	1,1	0,00013	1
GSPATT00011413001	0,314	0,1297	0	GSPATT00030860001	1,314	0,00014	1
GSPATT00011422001	-1,076	0,00614	-1	GSPATT00030871001	-0,434	0,01131	0
GSPATT00011430001	0,323	0,07271	0	GSPATT00030877001	-0,793	0,0011	0
GSPATT00011436001	0,608	0,01574	0	GSPATT00030894001	0,443	0,01543	0
GSPATT00011440001	0,123	0,57717	0	GSPATT00030895001	0,613	0,00519	0
GSPATT00011454001	0,507	0,07282	0	GSPATT00030907001	0,89	0,00402	0
GSPATT00011455001	-0,483	0,15659	0	GSPATT00030912001	1,631	0,00007	1
GSPATT00011486001	-0,104	0,60315	0	GSPATT00030921001	0,509	0,01961	0
GSPATT00011499001	-0,148	0,29042	0	GSPATT00030945001	0,658	0,01733	0
GSPATT00011546001	-0,929	0,00313	0	GSPATT00030956001	-0,545	0,00308	0
GSPATT00011547001	0,964	0,00026	0	GSPATT00030971001	0,707	0,00637	0
GSPATT00011564001	0,676	0,00108	0	GSPATT00030979001	0,011	0,94131	0
GSPATT00011566001	0,262	0,07738	0	GSPATT00030994001	0,373	0,06118	0
GSPATT00011580001	-0,02	0,90743	0	GSPATT00031006001	1,218	0,00004	1
GSPATT00011584001	0,283	0,12359	0	GSPATT00031028001	-0,522	0,00869	0
GSPATT00011622001	0,355	0,17874	0	GSPATT00031029001	-0,147	0,30646	0
GSPATT00011666001	-0,488	0,00499	0	GSPATT00031066001	-3,25	0	-1
GSPATT00011685001	-0,399	0,05343	0	GSPATT00031082001	0,807	0,00177	0
GSPATT00011700001	-0,131	0,51052	0	GSPATT00031097001	-0,034	0,81174	0
GSPATT00011714001	0,124	0,49457	0	GSPATT00031104001	-1,662	0,00021	-1
GSPATT00011716001	0,632	0,00185	0	GSPATT00031107001	0,915	0,00188	0
GSPATT00011729001	-0,066	0,66441	0	GSPATT00031110001	1,016	0,0098	1
GSPATT00011731001	0,353	0,05227	0	GSPATT00031155001	-0,356	0,05023	0
GSPATT00011735001	1,538	0,00053	1	GSPATT00031166001	0,279	0,06965	0
GSPATT00011739001	-0,466	0,04961	0	GSPATT00031167001	0,272	0,08891	0
GSPATT00011742001	1,365	0,00005	1	GSPATT00031168001	0,836	0,01523	0
GSPATT00011764001	0,296	0,13873	0	GSPATT00031178001	0,551	0,0218	0
GSPATT00011816001	-0,319	0,13633	0	GSPATT00031182001	-0,535	0,01622	0
GSPATT00011825001	-0,485	0,08169	0	GSPATT00031235001	0,643	0,00762	0
GSPATT00011826001	1,565	0,00133	1	GSPATT00031285001	-0,236	0,21081	0
GSPATT00011840001	-0,798	0,00327	0	GSPATT00031287001	0,155	0,35654	0
GSPATT00011854001	0,553	0,02078	0	GSPATT00031336001	0,795	0,00047	0
GSPATT00011856001	0,65	0,07665	0	GSPATT00031426001	-1,033	0,00006	-1

GSPATT00011864001	-0,091	0,49677	0	GSPATT00031441001	-0,048	0,82385	0
GSPATT00011887001	0,175	0,46958	0	GSPATT00031487001	0,99	0,002	0
GSPATT00011897001	0,61	0,00299	0	GSPATT00031493001	0,611	0,02344	0
GSPATT00011903001	0,087	0,58179	0	GSPATT00031509001	-0,798	0,00098	0
GSPATT00011917001	0,417	0,15463	0	GSPATT00031532001	1,665	0,00021	1
GSPATT00011929001	-1,564	0,00004	-1	GSPATT00031569001	-0,125	0,54879	0
GSPATT00011952001	0,118	0,38041	0	GSPATT00031618001	0,323	0,2332	0
GSPATT00011968001	0,326	0,1663	0	GSPATT00031623001	-0,002	0,9946	0
GSPATT00011981001	-0,032	0,83233	0	GSPATT00031630001	1,142	0,00103	1
GSPATT00011982001	0,265	0,10595	0	GSPATT00031642001	-1,737	0,00014	-1
GSPATT00012043001	0,391	0,11161	0	GSPATT00031645001	0,862	0,00026	0
GSPATT00012044001	-0,04	0,80131	0	GSPATT00031653001	-0,225	0,14582	0
GSPATT00012047001	-0,114	0,39986	0	GSPATT00031661001	0,548	0,02007	0
GSPATT00012141001	0,915	0,00115	0	GSPATT00031665001	-0,001	0,99648	0
GSPATT00012176001	0,545	0,02424	0	GSPATT00031698001	-0,689	0,03351	0
GSPATT00012210001	-0,372	0,02535	0	GSPATT00031701001	-0,914	0,00444	0
GSPATT00012216001	1,175	0,00014	1	GSPATT00031705001	1,81	0,00002	1
GSPATT00012223001	0,404	0,08406	0	GSPATT00031706001	-0,775	0,0011	0
GSPATT00012241001	0,159	0,5354	0	GSPATT00031738001	-1,034	0,00008	-1
GSPATT00012255001	0,104	0,64586	0	GSPATT00031739001	0,382	0,19043	0
GSPATT00012262001	-1,15	0,00006	-1	GSPATT00031740001	0,355	0,05225	0
GSPATT00012269001	-0,185	0,39154	0	GSPATT00031754001	-0,461	0,03297	0
GSPATT00012278001	1,263	0,00039	1	GSPATT00031756001	-0,463	0,03455	0
GSPATT00012282001	0,837	0,01461	0	GSPATT00031757001	0,716	0,03439	0
GSPATT00012286001	-0,79	0,00041	0	GSPATT00031762001	-1,768	0,00003	-1
GSPATT00012322001	1,362	0,00005	1	GSPATT00031768001	-0,037	0,82459	0
GSPATT00012339001	1,278	0,00002	1	GSPATT00031781001	-0,507	0,02156	0
GSPATT00012348001	-1,134	0,00014	-1	GSPATT00031782001	-0,24	0,42749	0
GSPATT00012351001	0,299	0,06088	0	GSPATT00031802001	0,907	0,00053	0
GSPATT00012371001	-0,352	0,29514	0	GSPATT00031822001	0,463	0,05116	0
GSPATT00012372001	0,15	0,484	0	GSPATT00031828001	-0,487	0,01743	0
GSPATT00012374001	0,203	0,24495	0	GSPATT00031846001	-0,183	0,21789	0
GSPATT00012376001	0,959	0,00045	0	GSPATT00031865001	0,272	0,16899	0
GSPATT00012379001	0,369	0,07668	0	GSPATT00031924001	0,545	0,00883	0
GSPATT00012395001	0,809	0,0075	0	GSPATT00031948001	-0,37	0,18789	0
GSPATT00012396001	0,319	0,11515	0	GSPATT00031955001	-0,129	0,4888	0
GSPATT00012400001	0,852	0,00032	0	GSPATT00031957001	0,03	0,87201	0
GSPATT00012445001	0,429	0,03783	0	GSPATT00032021001	0,096	0,48485	0
GSPATT00012460001	0,238	0,14276	0	GSPATT00032022001	-0,54	0,01533	0
GSPATT00012470001	-0,034	0,86348	0	GSPATT00032029001	0,283	0,22374	0
GSPATT00012488001	-0,04	0,75807	0	GSPATT00032030001	1,339	0,00008	1
GSPATT00012520001	-2,03	0	-1	GSPATT00032037001	0,772	0,00865	0
GSPATT00012525001	0,13	0,44838	0	GSPATT00032092001	0,508	0,09947	0
GSPATT00012543001	0,86	0,00059	0	GSPATT00032094001	-2,851	0	-1
GSPATT00012570001	0,319	0,06017	0	GSPATT00032095001	0,112	0,41059	0
GSPATT00012610001	0,358	0,06157	0	GSPATT00032100001	0,509	0,00574	0
GSPATT00012628001	0,326	0,03065	0	GSPATT00032104001	-0,024	0,9428	0
GSPATT00012631001	0,442	0,02953	0	GSPATT00032110001	-1,089	0,00427	-1
GSPATT00012633001	0,282	0,10356	0	GSPATT00032111001	-1,233	0,00541	-1
GSPATT00012634001	0,634	0,00459	0	GSPATT00032114001	0,44	0,01977	0
GSPATT00012649001	-0,517	0,01272	0	GSPATT00032136001	-0,671	0,00209	0
GSPATT00012656001	0,231	0,16634	0	GSPATT00032166001	0,482	0,04639	0
GSPATT00012666001	0,427	0,02248	0	GSPATT00032169001	0,497	0,00936	0
GSPATT00012684001	-0,036	0,83675	0	GSPATT00032224001	0,067	0,66182	0
GSPATT00012692001	0,375	0,08064	0	GSPATT00032226001	0,466	0,04696	0
GSPATT00012702001	0,193	0,34721	0	GSPATT00032238001	0,364	0,06778	0
GSPATT00012722001	0,574	0,01609	0	GSPATT00032288001	0,859	0,00547	0
GSPATT00012732001	0,363	0,12773	0	GSPATT00032289001	-0,097	0,49171	0
GSPATT00012735001	0,149	0,25751	0	GSPATT00032301001	-0,947	0,00029	0
GSPATT00012742001	1,227	0,00011	1	GSPATT00032341001	0,829	0,00058	0
GSPATT00012746001	0,143	0,51196	0	GSPATT00032372001	-0,241	0,09642	0
GSPATT00012753001	0,157	0,26935	0	GSPATT00032392001	0,313	0,05107	0
GSPATT00012754001	-0,119	0,5919	0	GSPATT00032421001	-1,221	0,00022	-1
GSPATT00012755001	1,516	0,00005	1	GSPATT00032429001	0,493	0,03216	0
GSPATT00012756001	0,835	0,00358	0	GSPATT00032432001	-0,292	0,20952	0
GSPATT00012822001	0,123	0,4374	0	GSPATT00032442001	0,198	0,2398	0
GSPATT00012835001	0,596	0,00563	0	GSPATT00032444001	-0,967	0,00044	0

GSPATT00012838001	0,001	0,99699	0	GSPATT00032458001	0,703	0,02409	0
GSPATT00012852001	1,275	0,00003	1	GSPATT00032459001	0,275	0,11268	0
GSPATT00012853001	0,823	0,00197	0	GSPATT00032460001	-0,526	0,00918	0
GSPATT00012859001	-0,198	0,15153	0	GSPATT00032522001	-0,127	0,45119	0
GSPATT00012868001	0,163	0,31281	0	GSPATT00032523001	0,812	0,00089	0
GSPATT00012875001	0,348	0,03129	0	GSPATT00032532001	0,103	0,45294	0
GSPATT00012914001	0,157	0,26322	0	GSPATT00032533001	-0,17	0,53723	0
GSPATT00012916001	-2,396	0	-1	GSPATT00032559001	0,124	0,37615	0
GSPATT00012931001	0,812	0,00089	0	GSPATT00032564001	1,204	0,00066	1
GSPATT00012973001	-0,403	0,04733	0	GSPATT00032574001	1,413	0,00228	1
GSPATT00012998001	-0,105	0,43684	0	GSPATT00032597001	0,884	0,00108	0
GSPATT00013005001	-0,158	0,4174	0	GSPATT00032618001	0,457	0,06613	0
GSPATT00013045001	0,257	0,10688	0	GSPATT00032619001	0,741	0,02826	0
GSPATT00013048001	0,033	0,83799	0	GSPATT00032623001	-0,162	0,37167	0
GSPATT00013062001	-0,119	0,40756	0	GSPATT00032667001	1,812	0,00041	1
GSPATT00013085001	1,595	0,00002	1	GSPATT00032670001	0,786	0,00243	0
GSPATT00013094001	0,81	0,00674	0	GSPATT00032697001	0,477	0,0138	0
GSPATT00013102001	0,769	0,00426	0	GSPATT00032706001	0,443	0,01419	0
GSPATT00013110001	-1,076	0,00259	-1	GSPATT00032739001	0,096	0,63648	0
GSPATT00013157001	0,235	0,17548	0	GSPATT00032755001	-0,448	0,01909	0
GSPATT00013159001	0,37	0,09003	0	GSPATT00032763001	-0,489	0,02531	0
GSPATT00013164001	-0,51	0,05354	0	GSPATT00032783001	-0,567	0,00797	0
GSPATT00013165001	1,021	0,00106	1	GSPATT00032791001	0,581	0,0063	0
GSPATT00013173001	-0,295	0,08555	0	GSPATT00032810001	0,795	0,00874	0
GSPATT00013174001	0,011	0,93101	0	GSPATT00032894001	0,722	0,00361	0
GSPATT00013205001	0,52	0,00824	0	GSPATT00032910001	1,39	0,00003	1
GSPATT00013243001	-0,027	0,84517	0	GSPATT00032920001	0,74	0,00144	0
GSPATT00013253001	1,885	0,00001	1	GSPATT00032929001	0,137	0,64968	0
GSPATT00013260001	0,645	0,00484	0	GSPATT00032930001	1,256	0,00033	1
GSPATT00013263001	0,563	0,01505	0	GSPATT00032934001	0,256	0,2624	0
GSPATT00013271001	0,175	0,21491	0	GSPATT00032999001	0,68	0,00704	0
GSPATT00013272001	1,673	0,00001	1	GSPATT00033009001	-0,785	0,00143	0
GSPATT00013281001	0,128	0,50204	0	GSPATT00033016001	0,224	0,20102	0
GSPATT00013308001	0,015	0,90603	0	GSPATT00033027001	0,378	0,03882	0
GSPATT00013315001	-0,188	0,20496	0	GSPATT00033067001	0,968	0,00046	0
GSPATT00013327001	-0,009	0,94794	0	GSPATT00033072001	0,525	0,02731	0
GSPATT00013343001	0,267	0,15492	0	GSPATT00033076001	0,219	0,41915	0
GSPATT00013356001	-0,722	0,00162	0	GSPATT00033095001	-1,102	0,00064	-1
GSPATT00013360001	-0,83	0,00139	0	GSPATT00033106001	0,765	0,00138	0
GSPATT00013364001	-0,11	0,50767	0	GSPATT00033119001	-0,51	0,03205	0
GSPATT00013383001	-1,314	0,0001	-1	GSPATT00033139001	0,432	0,01158	0
GSPATT00013453001	0,604	0,02056	0	GSPATT00033154001	1,026	0,00696	1
GSPATT00013470001	0,177	0,28718	0	GSPATT00033161001	-0,302	0,08205	0
GSPATT00013490001	0,322	0,07055	0	GSPATT00033165001	-0,046	0,8569	0
GSPATT00013494001	0,052	0,75665	0	GSPATT00033167001	-0,34	0,05638	0
GSPATT00013502001	-0,243	0,19365	0	GSPATT00033198001	0,629	0,09101	0
GSPATT00013552001	0,741	0,00042	0	GSPATT00033205001	0,673	0,00702	0
GSPATT00013561001	0,271	0,16205	0	GSPATT00033223001	-0,423	0,03978	0
GSPATT00013578001	1,991	0,00001	1	GSPATT00033237001	-0,283	0,16249	0
GSPATT00013652001	0,191	0,18598	0	GSPATT00033250001	-0,453	0,0188	0
GSPATT00013656001	0,171	0,2115	0	GSPATT00033258001	0,335	0,03868	0
GSPATT00013680001	-0,238	0,41631	0	GSPATT00033260001	-0,16	0,55088	0
GSPATT00013693001	0,493	0,01038	0	GSPATT00033306001	-0,043	0,85073	0
GSPATT00013695001	0,474	0,01357	0	GSPATT00033333001	0,576	0,00241	0
GSPATT00013702001	-0,192	0,32187	0	GSPATT00033334001	0,4	0,0313	0
GSPATT00013754001	-3,054	0	-1	GSPATT00033358001	-0,167	0,26712	0
GSPATT00013770001	-0,187	0,4332	0	GSPATT00033399001	-0,126	0,62052	0
GSPATT00013783001	1,026	0,01651	1	GSPATT00033466001	-1,107	0,00106	-1
GSPATT00013790001	0,074	0,5863	0	GSPATT00033469001	0,884	0,00069	0
GSPATT00013801001	0,361	0,0755	0	GSPATT00033477001	0,439	0,04845	0
GSPATT00013825001	-0,769	0,00205	0	GSPATT00033494001	0,481	0,13408	0
GSPATT00013827001	0,481	0,00732	0	GSPATT00033495001	1,143	0,00006	1
GSPATT00013828001	0,419	0,03295	0	GSPATT00033505001	0,875	0,00023	0
GSPATT00013847001	0,139	0,40055	0	GSPATT00033512001	-3,055	0	-1
GSPATT00013852001	-0,368	0,03397	0	GSPATT00033523001	-1,294	0,00005	-1
GSPATT00013855001	0,438	0,04631	0	GSPATT00033534001	0,523	0,02677	0
GSPATT00013876001	1,319	0,00047	1	GSPATT00033537001	0,391	0,03807	0

GSPATT00013891001	0,176	0,26584	0	GSPATT00033539001	0,246	0,40993	0
GSPATT00013892001	-1,136	0,00035	-1	GSPATT00033568001	-0,204	0,16501	0
GSPATT00013898001	0,162	0,43243	0	GSPATT00033573001	0,114	0,43633	0
GSPATT00013900001	-0,003	0,98838	0	GSPATT00033586001	0,277	0,16433	0
GSPATT00013901001	0,247	0,35648	0	GSPATT00033606001	-0,328	0,25723	0
GSPATT00013913001	1,633	0,00001	1	GSPATT00033617001	0,273	0,25396	0
GSPATT00013928001	0,8	0,00105	0	GSPATT00033636001	0,336	0,04963	0
GSPATT00013961001	2,283	0,00001	1	GSPATT00033646001	-0,077	0,64536	0
GSPATT00013977001	0,458	0,01806	0	GSPATT00033676001	0,609	0,03214	0
GSPATT00013994001	1,578	0,00006	1	GSPATT00033682001	-0,181	0,30005	0
GSPATT00014001001	0,554	0,00382	0	GSPATT00033727001	-0,264	0,20849	0
GSPATT00014008001	0,723	0,00107	0	GSPATT00033743001	0,187	0,34652	0
GSPATT00014044001	0,335	0,03981	0	GSPATT00033764001	1,303	0,00004	1
GSPATT00014072001	0,024	0,90147	0	GSPATT00033786001	0,824	0,01346	0
GSPATT00014093001	0,192	0,19217	0	GSPATT00033798001	0,882	0,00143	0
GSPATT00014104001	0,566	0,03625	0	GSPATT00033801001	0,274	0,11977	0
GSPATT00014124001	-1,671	0,00007	-1	GSPATT00033810001	-0,178	0,21774	0
GSPATT00014132001	2,015	0	1	GSPATT00033826001	0,54	0,01676	0
GSPATT00014139001	0,906	0,02173	0	GSPATT00033832001	1,004	0,0005	1
GSPATT00014152001	0,095	0,60893	0	GSPATT00033839001	0,232	0,20924	0
GSPATT00014158001	1,1	0,00099	1	GSPATT00033861001	0,201	0,23013	0
GSPATT00014196001	-0,265	0,26029	0	GSPATT00033863001	0,989	0,00013	0
GSPATT00014197001	0,509	0,02553	0	GSPATT00033871001	0,751	0,00036	0
GSPATT00014213001	-0,445	0,00944	0	GSPATT00033881001	0,29	0,19304	0
GSPATT00014216001	0,08	0,66083	0	GSPATT00033904001	0,322	0,04932	0
GSPATT00014218001	0,813	0,0031	0	GSPATT00033912001	0,771	0,0063	0
GSPATT00014219001	0,639	0,00371	0	GSPATT00033917001	0,213	0,23333	0
GSPATT00014235001	0,358	0,02401	0	GSPATT00033938001	1,074	0,00021	1
GSPATT00014239001	0,559	0,04551	0	GSPATT00033979001	-0,385	0,04696	0
GSPATT00014299001	0,536	0,03039	0	GSPATT00033980001	0,087	0,60132	0
GSPATT00014310001	0,41	0,03511	0	GSPATT00034005001	1,03	0,00044	1
GSPATT00014391001	0,443	0,007	0	GSPATT00034018001	-1,92	0	-1
GSPATT00014411001	-0,004	0,97822	0	GSPATT00034024001	1,786	0,00003	1
GSPATT00014414001	0,567	0,00623	0	GSPATT00034063001	-0,183	0,27172	0
GSPATT00014433001	-0,31	0,10884	0	GSPATT00034071001	0,388	0,02595	0
GSPATT00014461001	0,95	0,00019	0	GSPATT00034075001	-0,137	0,30288	0
GSPATT00014474001	-1,949	0	-1	GSPATT00034090001	0,737	0,00209	0
GSPATT00014478001	0,152	0,53003	0	GSPATT00034103001	-0,258	0,10215	0
GSPATT00014529001	1,182	0,00018	1	GSPATT00034104001	0,705	0,00489	0
GSPATT00014568001	-0,37	0,05496	0	GSPATT00034124001	-1,017	0,0022	-1
GSPATT00014606001	0,487	0,04116	0	GSPATT00034141001	0,708	0,00161	0
GSPATT00014629001	0,582	0,00301	0	GSPATT00034156001	-0,057	0,72295	0
GSPATT00014645001	0,006	0,97144	0	GSPATT00034171001	0,663	0,00966	0
GSPATT00014660001	0,098	0,64509	0	GSPATT00034175001	-0,329	0,08396	0
GSPATT00014673001	-0,235	0,2283	0	GSPATT00034216001	0,528	0,01503	0
GSPATT00014674001	0,791	0,00595	0	GSPATT00034230001	0,16	0,26608	0
GSPATT00014676001	0,429	0,07671	0	GSPATT00034233001	0,369	0,08874	0
GSPATT00014685001	0,887	0,0008	0	GSPATT00034270001	1,032	0,00088	1
GSPATT00014692001	-0,278	0,14785	0	GSPATT00034308001	-0,353	0,08333	0
GSPATT00014705001	0,351	0,08889	0	GSPATT00034314001	-1,355	0,00021	-1
GSPATT00014745001	0,289	0,08436	0	GSPATT00034329001	1,249	0,00003	1
GSPATT00014759001	1,203	0,00129	1	GSPATT00034393001	-1,986	0,00002	-1
GSPATT00014793001	-0,201	0,47536	0	GSPATT00034405001	-0,584	0,00941	0
GSPATT00014796001	1,387	0,00001	1	GSPATT00034420001	-0,659	0,00959	0
GSPATT00014810001	0,609	0,01875	0	GSPATT00034421001	0,663	0,12721	0
GSPATT00014841001	0,356	0,10216	0	GSPATT00034463001	0,197	0,40106	0
GSPATT00014872001	0,244	0,12625	0	GSPATT00034473001	0,036	0,77796	0
GSPATT00014873001	0,194	0,50101	0	GSPATT00034552001	1,257	0,00015	1
GSPATT00014895001	0,711	0,00204	0	GSPATT00034577001	-0,839	0,00058	0
GSPATT00014900001	0,37	0,05706	0	GSPATT00034596001	0,345	0,26159	0
GSPATT00014918001	-0,327	0,04948	0	GSPATT00034603001	0,885	0,0034	0
GSPATT00014958001	-0,462	0,20687	0	GSPATT00034615001	0,724	0,00057	0
GSPATT00014962001	-0,017	0,92935	0	GSPATT00034623001	0,426	0,02613	0
GSPATT00014966001	-0,912	0,0003	0	GSPATT00034631001	0,527	0,0108	0
GSPATT00014999001	0,415	0,07919	0	GSPATT00034632001	-0,156	0,30353	0
GSPATT00015015001	0,687	0,00414	0	GSPATT00034637001	0,89	0,00286	0
GSPATT00015017001	-1,086	0,00027	-1	GSPATT00034640001	1,246	0,00004	1

GSPATT00015018001	-0,08	0,70257	0	GSPATT00034647001	0,189	0,18304	0
GSPATT00015021001	-1,722	0,00006	-1	GSPATT00034648001	0,251	0,12628	0
GSPATT00015033001	-0,23	0,21296	0	GSPATT00034657001	0,421	0,01754	0
GSPATT00015042001	-0,895	0,00041	0	GSPATT00034667001	-0,213	0,27669	0
GSPATT00015043001	0,268	0,46979	0	GSPATT00034770001	0,27	0,13463	0
GSPATT00015055001	0,271	0,36469	0	GSPATT00034806001	-0,248	0,17157	0
GSPATT00015064001	0,376	0,08595	0	GSPATT00034819001	0,638	0,01374	0
GSPATT00015109001	-0,005	0,9692	0	GSPATT00034862001	0,911	0,00088	0
GSPATT00015151001	0,296	0,07883	0	GSPATT00034863001	-2,801	0	-1
GSPATT00015169001	0,432	0,17161	0	GSPATT00034885001	0,254	0,14736	0
GSPATT00015210001	1,87	0,00003	1	GSPATT00034888001	0,387	0,09919	0
GSPATT00015238001	1,322	0,00004	1	GSPATT00034893001	0,489	0,0257	0
GSPATT00015246001	0,577	0,07312	0	GSPATT00034900001	0,251	0,34442	0
GSPATT00015257001	-0,315	0,32799	0	GSPATT00034902001	-0,054	0,71793	0
GSPATT00015261001	0,509	0,06899	0	GSPATT00034911001	0,966	0,00178	0
GSPATT00015299001	0,213	0,19618	0	GSPATT00034927001	0,954	0,00128	0
GSPATT00015301001	0,024	0,90708	0	GSPATT00034954001	-0,254	0,17321	0
GSPATT00015311001	0,153	0,48604	0	GSPATT00034968001	0,919	0,00041	0
GSPATT00015312001	-0,675	0,00141	0	GSPATT00034975001	-0,694	0,00453	0
GSPATT00015325001	0,179	0,42392	0	GSPATT00034994001	0,228	0,11464	0
GSPATT00015335001	0,857	0,00098	0	GSPATT00034997001	0,566	0,00681	0
GSPATT00015344001	0,355	0,08801	0	GSPATT00035001001	0,72	0,00113	0
GSPATT00015351001	0,593	0,00674	0	GSPATT00035014001	0,215	0,22573	0
GSPATT00015356001	-0,33	0,11053	0	GSPATT00035015001	-0,804	0,00518	0
GSPATT00015363001	-0,898	0,00028	0	GSPATT00035041001	0,7	0,01007	0
GSPATT00015373001	0,163	0,24261	0	GSPATT00035046001	-0,126	0,63021	0
GSPATT00015374001	0,211	0,22091	0	GSPATT00035048001	-0,034	0,8154	0
GSPATT00015382001	1,396	0,00009	1	GSPATT00035049001	1,06	0,00012	1
GSPATT00015396001	0,853	0,02037	0	GSPATT00035050001	1,458	0,0075	1
GSPATT00015413001	1,181	0,00207	1	GSPATT00035051001	1,15	0,00008	1
GSPATT00015428001	-0,166	0,38106	0	GSPATT00035055001	-2,179	0	-1
GSPATT00015448001	1,213	0,00004	1	GSPATT00035058001	0,603	0,00388	0
GSPATT00015474001	0,646	0,00966	0	GSPATT00035098001	-0,426	0,01033	0
GSPATT00015475001	0,366	0,0282	0	GSPATT00035101001	0,274	0,13516	0
GSPATT00015479001	0,147	0,2715	0	GSPATT00035109001	0,17	0,21947	0
GSPATT00015588001	0,437	0,0437	0	GSPATT00035113001	-3,146	0	-1
GSPATT00015597001	0,345	0,07945	0	GSPATT00035115001	0,815	0,01116	0
GSPATT00015625001	-1,091	0,00006	-1	GSPATT00035116001	0,615	0,00474	0
GSPATT00015632001	1,616	0,00334	1	GSPATT00035132001	0,541	0,03772	0
GSPATT00015635001	0,054	0,76188	0	GSPATT00035156001	0,909	0,02167	0
GSPATT00015640001	0,853	0,00185	0	GSPATT00035162001	0,462	0,02625	0
GSPATT00015666001	-0,623	0,00761	0	GSPATT00035168001	-0,967	0,00127	0
GSPATT00015671001	-0,147	0,30793	0	GSPATT00035170001	0,275	0,20192	0
GSPATT00015678001	0,121	0,52246	0	GSPATT00035215001	-0,514	0,05916	0
GSPATT00015687001	0,716	0,00115	0	GSPATT00035221001	1,144	0,00247	1
GSPATT00015690001	0,723	0,00416	0	GSPATT00035230001	0,202	0,239	0
GSPATT00015693001	-0,397	0,09858	0	GSPATT00035231001	0,708	0,00161	0
GSPATT00015702001	0,703	0,00191	0	GSPATT00035242001	0,289	0,32037	0
GSPATT00015703001	0,498	0,0388	0	GSPATT00035265001	-0,98	0,00136	0
GSPATT00015729001	0,223	0,18041	0	GSPATT00035273001	0,212	0,15905	0
GSPATT00015750001	1,122	0,00018	1	GSPATT00035283001	0,586	0,00587	0
GSPATT00015756001	0,261	0,13282	0	GSPATT00035344001	1,515	0,00002	1
GSPATT00015783001	0,994	0,00013	0	GSPATT00035345001	0,075	0,59803	0
GSPATT00015804001	-0,028	0,89234	0	GSPATT00035369001	1,788	0	1
GSPATT00015821001	-0,236	0,16594	0	GSPATT00035370001	0,774	0,00252	0
GSPATT00015826001	-1,025	0,00242	-1	GSPATT00035435001	0,252	0,14704	0
GSPATT00015830001	2,01	0,00006	1	GSPATT00035439001	0,245	0,18477	0
GSPATT00015852001	0,793	0,01264	0	GSPATT00035512001	0,145	0,51374	0
GSPATT00015854001	-0,969	0,00154	0	GSPATT00035537001	-0,316	0,13551	0
GSPATT00015864001	0,541	0,09018	0	GSPATT00035553001	0,19	0,42969	0
GSPATT00015881001	0,435	0,06228	0	GSPATT00035558001	0,435	0,06307	0
GSPATT00015890001	0,521	0,0327	0	GSPATT00035563001	0,506	0,20376	0
GSPATT00015896001	0,187	0,19576	0	GSPATT00035570001	0,438	0,14386	0
GSPATT00015906001	-0,376	0,07667	0	GSPATT00035586001	0,216	0,24577	0
GSPATT00015913001	-3,714	0	-1	GSPATT00035588001	1,375	0,00072	1
GSPATT00015946001	-0,176	0,47867	0	GSPATT00035590001	-0,098	0,61368	0
GSPATT00015989001	0,756	0,0358	0	GSPATT00035613001	0,93	0,00031	0

GSPATT00015997001	0,616	0,05511	0	GSPATT00035630001	0,599	0,00235	0
GSPATT00015998001	0,057	0,70953	0	GSPATT00035632001	1,647	0,00001	1
GSPATT00016007001	-1,006	0,00099	-1	GSPATT00035666001	0,319	0,04691	0
GSPATT00016022001	0,297	0,04559	0	GSPATT00035684001	0,079	0,61168	0
GSPATT00016031001	1,983	0	1	GSPATT00035689001	-0,122	0,45197	0
GSPATT00016058001	0,403	0,0261	0	GSPATT00035711001	-0,148	0,32112	0
GSPATT00016068001	0,584	0,00865	0	GSPATT00035742001	-0,345	0,03219	0
GSPATT00016075001	0,476	0,01419	0	GSPATT00035743001	-0,119	0,44406	0
GSPATT00016078001	-0,46	0,01198	0	GSPATT00035748001	0,686	0,00459	0
GSPATT00016099001	-0,376	0,0569	0	GSPATT00035791001	-0,365	0,03388	0
GSPATT00016118001	0,681	0,00081	0	GSPATT00035808001	-0,036	0,8091	0
GSPATT00016163001	0,936	0,00075	0	GSPATT00035812001	0,025	0,89409	0
GSPATT00016186001	0,651	0,00328	0	GSPATT00035830001	0,647	0,0186	0
GSPATT00016201001	0,264	0,12078	0	GSPATT00035834001	0,834	0,00201	0
GSPATT00016202001	0,942	0,0002	0	GSPATT00035841001	-0,305	0,05898	0
GSPATT00016224001	0,577	0,00541	0	GSPATT00035846001	-0,018	0,91105	0
GSPATT00016246001	1,272	0,00554	1	GSPATT00035878001	-0,682	0,01003	0
GSPATT00016252001	-1,875	0,00002	-1	GSPATT00035892001	0,871	0,00133	0
GSPATT00016286001	0,878	0,0129	0	GSPATT00035967001	-1,028	0,00178	-1
GSPATT00016304001	0,003	0,98948	0	GSPATT00035984001	0,033	0,81622	0
GSPATT00016323001	0,645	0,02514	0	GSPATT00035987001	0,061	0,629	0
GSPATT00016324001	0,417	0,15142	0	GSPATT00035988001	0,121	0,62093	0
GSPATT00016370001	0,574	0,02089	0	GSPATT00035996001	1,583	0,00009	1
GSPATT00016374001	0,711	0,02701	0	GSPATT00036001001	0,324	0,09202	0
GSPATT00016390001	-0,025	0,88764	0	GSPATT00036020001	0,633	0,01487	0
GSPATT00016393001	0,939	0,0007	0	GSPATT00036021001	-0,784	0,08263	0
GSPATT00016396001	-0,359	0,34319	0	GSPATT00036025001	-0,396	0,01622	0
GSPATT00016433001	0,343	0,0523	0	GSPATT00036029001	0,388	0,03177	0
GSPATT00016451001	0,509	0,00351	0	GSPATT00036032001	-0,747	0,00511	0
GSPATT00016461001	2,349	0	1	GSPATT00036035001	1,064	0,00084	1
GSPATT00016467001	0,07	0,6374	0	GSPATT00036037001	0,29	0,15131	0
GSPATT00016473001	-0,231	0,19236	0	GSPATT00036059001	-2,602	0,00001	-1
GSPATT00016474001	-0,225	0,18313	0	GSPATT00036080001	-0,995	0,00114	0
GSPATT00016484001	0,021	0,92665	0	GSPATT00036130001	1,008	0,00009	1
GSPATT00016487001	0,393	0,10186	0	GSPATT00036133001	0,425	0,08763	0
GSPATT00016492001	1,714	0,00007	1	GSPATT00036142001	0,347	0,06142	0
GSPATT00016497001	0,83	0,00243	0	GSPATT00036146001	-0,276	0,13155	0
GSPATT00016499001	2,913	0	1	GSPATT00036182001	0,139	0,41765	0
GSPATT00016501001	0,408	0,05581	0	GSPATT00036198001	0,154	0,39188	0
GSPATT00016508001	-0,185	0,31276	0	GSPATT00036212001	0,53	0,00883	0
GSPATT00016522001	1,486	0,00001	1	GSPATT00036217001	-0,481	0,07457	0
GSPATT00016536001	0,338	0,11356	0	GSPATT00036238001	0,234	0,24062	0
GSPATT00016555001	0,08	0,62165	0	GSPATT00036250001	0,42	0,01334	0
GSPATT00016572001	0,158	0,27397	0	GSPATT00036252001	-0,676	0,01753	0
GSPATT00016579001	-0,042	0,75571	0	GSPATT00036286001	-0,683	0,00156	0
GSPATT00016580001	1,975	0	1	GSPATT00036336001	0,222	0,12243	0
GSPATT00016581001	0,583	0,00498	0	GSPATT00036337001	0,687	0,00442	0
GSPATT00016584001	0,48	0,00759	0	GSPATT00036353001	0,737	0,00133	0
GSPATT00016598001	-0,315	0,17406	0	GSPATT00036407001	0,356	0,04968	0
GSPATT00016621001	-0,635	0,00167	0	GSPATT00036409001	0,316	0,04048	0
GSPATT00016625001	0,578	0,01915	0	GSPATT00036434001	0,218	0,17014	0
GSPATT00016626001	-1,01	0,01795	-1	GSPATT00036445001	0,343	0,02548	0
GSPATT00016635001	1,276	0,00002	1	GSPATT00036459001	1,229	0,0001	1
GSPATT00016642001	0,83	0,00038	0	GSPATT00036469001	0,587	0,01017	0
GSPATT00016644001	-0,085	0,60496	0	GSPATT00036471001	0,051	0,77388	0
GSPATT00016649001	0,549	0,0028	0	GSPATT00036474001	0,054	0,83167	0
GSPATT00016655001	-0,315	0,1207	0	GSPATT00036479001	-0,35	0,11614	0
GSPATT00016684001	0,371	0,03571	0	GSPATT00036483001	-0,011	0,94317	0
GSPATT00016686001	-0,649	0,0057	0	GSPATT00036486001	-0,301	0,19234	0
GSPATT00016707001	0,035	0,81827	0	GSPATT00036520001	1,333	0,00049	1
GSPATT00016715001	-0,125	0,40578	0	GSPATT00036521001	-1,453	0,00724	-1
GSPATT00016728001	0,796	0,00369	0	GSPATT00036542001	2,353	0	1
GSPATT00016739001	0,05	0,81698	0	GSPATT00036543001	3,458	0	1
GSPATT00016753001	0,303	0,03871	0	GSPATT00036578001	0,012	0,94123	0
GSPATT00016756001	0,551	0,01981	0	GSPATT00036602001	-0,016	0,91827	0
GSPATT00016834001	-0,177	0,2236	0	GSPATT00036604001	0,849	0,00077	0
GSPATT00016841001	0,199	0,27702	0	GSPATT00036632001	0,404	0,0432	0

GSPATT00016868001	-0,668	0,00534	0	GSPATT00036666001	-0,42	0,03381	0
GSPATT00016887001	-0,259	0,12658	0	GSPATT00036671001	-0,905	0,00646	0
GSPATT00016943001	-0,089	0,60758	0	GSPATT00036678001	0,014	0,9207	0
GSPATT00016964001	0,04	0,87452	0	GSPATT00036688001	0,31	0,04238	0
GSPATT00016974001	-0,017	0,9121	0	GSPATT00036690001	0,725	0,00628	0
GSPATT00016976001	1,509	0,00004	1	GSPATT00036705001	1,156	0,00008	1
GSPATT00016985001	0,32	0,1398	0	GSPATT00036706001	0,883	0,00746	0
GSPATT00017038001	2	0	1	GSPATT00036730001	-0,04	0,79056	0
GSPATT00017050001	0,473	0,00736	0	GSPATT00036752001	0,349	0,08868	0
GSPATT00017063001	0,061	0,69591	0	GSPATT00036770001	0,189	0,34256	0
GSPATT00017094001	0,37	0,12392	0	GSPATT00036776001	-0,3	0,06534	0
GSPATT00017107001	0,512	0,01311	0	GSPATT00036800001	-0,04	0,79823	0
GSPATT00017117001	1,483	0,00007	1	GSPATT00036806001	-0,03	0,88012	0
GSPATT00017122001	-0,89	0,00112	0	GSPATT00036817001	-0,834	0,01188	0
GSPATT00017148001	0,445	0,01161	0	GSPATT00036862001	-1,618	0,00012	-1
GSPATT00017157001	0,228	0,29231	0	GSPATT00036876001	0,655	0,01693	0
GSPATT00017193001	0,423	0,02362	0	GSPATT00036879001	0,685	0,00059	0
GSPATT00017227001	0,713	0,01736	0	GSPATT00036888001	0,452	0,00944	0
GSPATT00017228001	-1,143	0,00016	-1	GSPATT00036914001	0,754	0,10066	0
GSPATT00017248001	0,004	0,98061	0	GSPATT00036919001	-0,269	0,14769	0
GSPATT00017265001	0,224	0,27585	0	GSPATT00036954001	-0,592	0,0063	0
GSPATT00017287001	0,722	0,00261	0	GSPATT00036971001	-0,218	0,16152	0
GSPATT00017341001	-0,483	0,07425	0	GSPATT00037000001	0,283	0,14601	0
GSPATT00017350001	-0,237	0,16156	0	GSPATT00037053001	-0,135	0,29881	0
GSPATT00017354001	-0,088	0,60425	0	GSPATT00037058001	0,084	0,66195	0
GSPATT00017356001	1,623	0,00011	1	GSPATT00037066001	-0,414	0,01584	0
GSPATT00017361001	0,54	0,0029	0	GSPATT00037088001	0,441	0,01862	0
GSPATT00017388001	-0,64	0,00112	0	GSPATT00037094001	-0,008	0,9585	0
GSPATT00017455001	0,77	0,0007	0	GSPATT00037123001	0,774	0,01287	0
GSPATT00017492001	0,873	0,0003	0	GSPATT00037129001	0,656	0,00104	0
GSPATT00017543001	1,645	0,00001	1	GSPATT00037143001	-0,027	0,85661	0
GSPATT00017557001	0,172	0,32619	0	GSPATT00037145001	0,616	0,02922	0
GSPATT00017576001	1,079	0,00068	1	GSPATT00037148001	-0,358	0,09184	0
GSPATT00017630001	-0,188	0,2095	0	GSPATT00037154001	-0,557	0,01543	0
GSPATT00017662001	0,243	0,10339	0	GSPATT00037166001	1	0,00065	0
GSPATT00017666001	0,164	0,29665	0	GSPATT00037168001	0,397	0,02983	0
GSPATT00017671001	1,215	0,00163	1	GSPATT00037172001	0,145	0,33707	0
GSPATT00017672001	0,666	0,00486	0	GSPATT00037211001	0,931	0,00079	0
GSPATT00017676001	0,897	0,00014	0	GSPATT00037212001	0,689	0,00673	0
GSPATT00017680001	1,263	0,0002	1	GSPATT00037226001	0,202	0,30966	0
GSPATT00017681001	1,015	0,00199	1	GSPATT00037232001	0,667	0,00174	0
GSPATT00017692001	0,757	0,02894	0	GSPATT00037285001	0,579	0,00719	0
GSPATT00017712001	-0,359	0,17781	0	GSPATT00037291001	-2,613	0,00006	-1
GSPATT00017715001	1,019	0,00017	1	GSPATT00037311001	-0,268	0,09834	0
GSPATT00017746001	0,26	0,0958	0	GSPATT00037328001	0,772	0,00301	0
GSPATT00017766001	-1,364	0,00026	-1	GSPATT00037330001	0,474	0,01266	0
GSPATT00017771001	0,257	0,1542	0	GSPATT00037368001	1,031	0,0019	1
GSPATT00017791001	-1,005	0,00013	-1	GSPATT00037376001	0,236	0,20339	0
GSPATT00017820001	0,089	0,63426	0	GSPATT00037398001	0,097	0,63269	0
GSPATT00017833001	1,804	0,00001	1	GSPATT00037444001	-0,662	0,00643	0
GSPATT00017845001	-0,337	0,04954	0	GSPATT00037451001	0,414	0,01229	0
GSPATT00017866001	-0,949	0,00081	0	GSPATT00037471001	0,104	0,58331	0
GSPATT00017868001	-0,188	0,4063	0	GSPATT00037488001	1,394	0,00067	1
GSPATT00017882001	-0,011	0,94767	0	GSPATT00037526001	0,897	0,00043	0
GSPATT00017902001	-0,048	0,73859	0	GSPATT00037566001	-0,05	0,76612	0
GSPATT00017904001	0,196	0,20412	0	GSPATT00037597001	-0,102	0,55503	0
GSPATT00017915001	0,763	0,00829	0	GSPATT00037660001	0,186	0,17268	0
GSPATT00017926001	-0,085	0,54936	0	GSPATT00037661001	0,066	0,71158	0
GSPATT00017952001	0,822	0,00042	0	GSPATT00037688001	0,303	0,04893	0
GSPATT00017953001	0,93	0,00178	0	GSPATT00037699001	0,741	0,00075	0
GSPATT00017960001	-0,674	0,00313	0	GSPATT00037711001	-0,444	0,05547	0
GSPATT00017963001	-0,275	0,06466	0	GSPATT00037715001	0,109	0,42522	0
GSPATT00017966001	0,227	0,14352	0	GSPATT00037722001	0,821	0,0005	0
GSPATT00017972001	-0,131	0,38508	0	GSPATT00037727001	0,543	0,04234	0
GSPATT00017992001	-0,11	0,50579	0	GSPATT00037737001	1,364	0,0001	1
GSPATT00018004001	0,463	0,03497	0	GSPATT00037754001	-0,856	0,0023	0
GSPATT00018101001	0,898	0,00614	0	GSPATT00037781001	-1,255	0,00102	-1

GSPATT00018127001	0,153	0,36392	0	GSPATT00037784001	0,072	0,57853	0
GSPATT00018132001	0,741	0,02921	0	GSPATT00037789001	2,068	0	1
GSPATT00018133001	-0,081	0,66594	0	GSPATT00037792001	-0,369	0,18448	0
GSPATT00018143001	0,268	0,2351	0	GSPATT00037795001	0,362	0,01982	0
GSPATT00018153001	-0,086	0,72806	0	GSPATT00037796001	1,094	0,0001	1
GSPATT00018163001	0,342	0,02849	0	GSPATT00037798001	0,035	0,81328	0
GSPATT00018188001	0,221	0,21776	0	GSPATT00037811001	0,21	0,2921	0
GSPATT00018202001	0,66	0,01285	0	GSPATT00037815001	0,925	0,00331	0
GSPATT00018223001	-0,409	0,02988	0	GSPATT00037818001	0,928	0,00072	0
GSPATT00018263001	0,814	0,00153	0	GSPATT00037856001	0,303	0,06934	0
GSPATT00018280001	0,316	0,0745	0	GSPATT00037875001	-0,066	0,66305	0
GSPATT00018290001	-0,359	0,04447	0	GSPATT00037876001	0,446	0,01048	0
GSPATT00018291001	0,141	0,45858	0	GSPATT00037939001	0,394	0,02006	0
GSPATT00018293001	0,169	0,30306	0	GSPATT00037945001	-0,437	0,02189	0
GSPATT00018389001	-0,094	0,58557	0	GSPATT00037967001	-0,06	0,6776	0
GSPATT00018398001	0,481	0,09586	0	GSPATT00037978001	0,461	0,08571	0
GSPATT00018431001	0,56	0,04386	0	GSPATT00038025001	-0,104	0,48304	0
GSPATT00018438001	0,545	0,01322	0	GSPATT00038027001	0,502	0,05619	0
GSPATT00018449001	0,58	0,0723	0	GSPATT00038035001	0,888	0,00025	0
GSPATT00018471001	-1,113	0,00027	-1	GSPATT00038045001	-0,396	0,05929	0
GSPATT00018476001	0,992	0,00041	0	GSPATT00038090001	1,233	0,00044	1
GSPATT00018515001	0,206	0,16244	0	GSPATT00038149001	0,258	0,07307	0
GSPATT00018529001	0,92	0,01084	0	GSPATT00038155001	0,75	0,00768	0
GSPATT00018559001	0,48	0,01489	0	GSPATT00038156001	0,107	0,61347	0
GSPATT00018563001	0,407	0,05649	0	GSPATT00038162001	-0,205	0,24742	0
GSPATT00018600001	0,51	0,01239	0	GSPATT00038171001	-1,199	0,00032	-1
GSPATT00018603001	-0,883	0,002	0	GSPATT00038175001	-0,718	0,00151	0
GSPATT00018608001	0,219	0,14898	0	GSPATT00038196001	0,025	0,86468	0
GSPATT00018634001	-0,253	0,0798	0	GSPATT00038198001	1,751	0,00019	1
GSPATT00018635001	-4,269	0	-1	GSPATT00038202001	1,049	0,00014	1
GSPATT00018645001	-0,232	0,15845	0	GSPATT00038203001	1,279	0,00007	1
GSPATT00018666001	0,125	0,44695	0	GSPATT00038212001	0,313	0,14446	0
GSPATT00018671001	0,675	0,00088	0	GSPATT00038214001	2,959	0	1
GSPATT00018681001	-0,683	0,00496	0	GSPATT00038217001	1,138	0,00032	1
GSPATT00018695001	-0,076	0,62948	0	GSPATT00038232001	-1,557	0,00002	-1
GSPATT00018702001	-0,494	0,02855	0	GSPATT00038377001	1,241	0,00049	1
GSPATT00018706001	0,301	0,2524	0	GSPATT00038439001	0,034	0,86173	0
GSPATT00018710001	-0,217	0,14626	0	GSPATT00038442001	0,573	0,00219	0
GSPATT00018727001	-0,029	0,84109	0	GSPATT00038453001	0,958	0,00056	0
GSPATT00018728001	-0,429	0,04057	0	GSPATT00038457001	0,034	0,80944	0
GSPATT00018737001	0,112	0,42429	0	GSPATT00038462001	0,25	0,17848	0
GSPATT00018806001	-0,089	0,50827	0	GSPATT00038469001	1,122	0,00009	1
GSPATT00018878001	-0,526	0,01436	0	GSPATT00038489001	0,49	0,00846	0
GSPATT00018891001	-0,275	0,12204	0	GSPATT00038497001	0,218	0,14041	0
GSPATT00018918001	0,815	0,00108	0	GSPATT00038500001	-0,057	0,70837	0
GSPATT00018950001	0,098	0,47385	0	GSPATT00038501001	0,115	0,39982	0
GSPATT00018952001	0,199	0,36354	0	GSPATT00038509001	0,207	0,13138	0
GSPATT00018964001	0,792	0,02949	0	GSPATT00038517001	0,388	0,06212	0
GSPATT00018965001	0,578	0,00897	0	GSPATT00038525001	0,766	0,00172	0
GSPATT00018969001	-0,459	0,03222	0	GSPATT00038565001	-0,503	0,00816	0
GSPATT00018978001	1,009	0,00294	1	GSPATT00038594001	-0,531	0,01391	0
GSPATT00019012001	0,241	0,25273	0	GSPATT00038759001	0,112	0,58404	0
GSPATT00019047001	-0,313	0,12451	0	GSPATT00038767001	0,906	0,00064	0
GSPATT00019068001	0,352	0,08916	0	GSPATT00038772001	0,189	0,25284	0
GSPATT00019089001	-0,092	0,50555	0	GSPATT00038778001	0,45	0,0114	0
GSPATT00019098001	-0,396	0,01375	0	GSPATT00038811001	0,163	0,23056	0
GSPATT00019105001	-0,564	0,03875	0	GSPATT00038843001	0,394	0,08578	0
GSPATT00019121001	-0,238	0,13986	0	GSPATT00038849001	0,132	0,34446	0
GSPATT00019142001	0,119	0,67944	0	GSPATT00038851001	-0,622	0,0036	0
GSPATT00019147001	1,334	0,00037	1	GSPATT00038853001	0,357	0,03403	0
GSPATT00019217001	0,697	0,00662	0	GSPATT00038854001	0,067	0,67394	0
GSPATT00019232001	1,248	0,0003	1	GSPATT00038872001	-0,347	0,10873	0
GSPATT00019272001	0,626	0,00167	0	GSPATT00038895001	-2,984	0,00003	-1
GSPATT00019281001	0,242	0,15235	0	GSPATT00038896001	-0,151	0,65702	0
GSPATT00019357001	-0,269	0,11687	0	GSPATT00038914001	1,241	0,00033	1
GSPATT00019361001	1,018	0,00339	1	GSPATT00038932001	0,144	0,35304	0
GSPATT00019366001	0,029	0,8276	0	GSPATT00038938001	0,67	0,06631	0

GSPATT00019371001	-1,072	0,00005	-1	GSPATT00039020001	1,009	0,00006	1
GSPATT00019421001	0,515	0,01534	0	GSPATT00039058001	0,227	0,19888	0
GSPATT00019447001	1,328	0,00111	1	GSPATT00039143001	0,942	0,00145	0
GSPATT00019448001	1,274	0,00003	1	GSPATT00039147001	-0,456	0,10233	0
GSPATT00019460001	0,582	0,01671	0	GSPATT00039305001	0,494	0,01965	0
GSPATT00019477001	-0,546	0,02587	0	GSPATT00039325001	0,34	0,08666	0
GSPATT00019493001	-0,409	0,16665	0	GSPATT00039337001	0,067	0,62405	0
GSPATT00019504001	0,507	0,03568	0	GSPATT00039369001	-0,01	0,94511	0
GSPATT00019520001	1,19	0,00006	1	GSPATT00039403001	1,013	0,00053	1
GSPATT00019546001	0,939	0,00501	0	GSPATT00039502001	-0,434	0,08958	0
GSPATT00019584001	0,197	0,24399	0	GSPATT00039519001	0,599	0,01071	0
GSPATT00019589001	-0,23	0,14791	0	GSPATT00039578001	1,071	0,0029	1
GSPATT00019597001	0,628	0,00396	0	GSPATT00039596001	-0,065	0,63218	0
GSPATT00019614001	-0,092	0,48818	0	GSPATT00039617001	-0,125	0,38541	0
GSPATT00019621001	1,048	0,0001	1	GSPATT00039623001	-0,179	0,37866	0
GSPATT00019623001	0,397	0,02171	0	GSPATT00039672001	0,81	0,01218	0
GSPATT00019627001	0,267	0,13823	0	GSPATT00039701001	0,824	0,06378	0
GSPATT00019648001	0,266	0,10254	0	GSPATT00039733001	0,707	0,00233	0
GSPATT00019665001	0,352	0,16137	0	GSPATT00039825001	0,881	0,00181	0
GSPATT00019672001	0,262	0,38511	0	PTETT10500005001	0,689	0,01507	0
GSPATT00019684001	0,599	0,03897	0	PTETT10800002001	-0,131	0,51208	0
GSPATT00019701001	0,633	0,00548	0				

GO 0006793 phosphorus metabolic process / GO 0006796 phosphate-containing compound metabolic process

ID	coeff.	p-value	signif.	ID	coeff.	p-value	signif.
GSPATT00000029001	-0,656	0,02257	0	GSPATT00019603001	0,551	0,01253	0
GSPATT00000047001	0,686	0,01107	0	GSPATT00019614001	-0,092	0,48818	0
GSPATT00000064001	-0,605	0,00365	0	GSPATT00019621001	1,048	0,0001	1
GSPATT00000093001	0,319	0,15526	0	GSPATT00019622001	0,562	0,00554	0
GSPATT00000160001	-0,436	0,09808	0	GSPATT00019623001	0,397	0,02171	0
GSPATT00000190001	0,762	0,00321	0	GSPATT00019627001	0,267	0,13823	0
GSPATT00000282001	-0,179	0,30932	0	GSPATT00019634001	-0,335	0,03168	0
GSPATT00000295001	0,583	0,02578	0	GSPATT00019648001	0,266	0,10254	0
GSPATT00000324001	-0,092	0,63602	0	GSPATT00019665001	0,352	0,16137	0
GSPATT00000365001	0,81	0,00102	0	GSPATT00019672001	0,262	0,38511	0
GSPATT00000366001	0,639	0,01895	0	GSPATT00019684001	0,599	0,03897	0
GSPATT00000384001	0,653	0,02308	0	GSPATT00019701001	0,633	0,00548	0
GSPATT00000394001	-0,275	0,16617	0	GSPATT00019734001	0,538	0,01044	0
GSPATT00000415001	-0,571	0,00652	0	GSPATT00019758001	-0,857	0,00047	0
GSPATT00000434001	0,504	0,02973	0	GSPATT00019770001	0,159	0,3745	0
GSPATT00000482001	-0,044	0,82968	0	GSPATT00019793001	0,172	0,36914	0
GSPATT00000508001	-0,009	0,95625	0	GSPATT00019834001	0,855	0,00735	0
GSPATT00000542001	0,969	0,00094	0	GSPATT00019835001	0,65	0,03101	0
GSPATT00000559001	0,401	0,04709	0	GSPATT00019840001	0,546	0,02765	0
GSPATT00000560001	0,804	0,0092	0	GSPATT00019861001	0,155	0,42691	0
GSPATT00000594001	-1,714	0,00001	-1	GSPATT00019876001	-0,036	0,9177	0
GSPATT00000596001	-1,334	0,00022	-1	GSPATT00019885001	1,059	0,00032	1
GSPATT00000599001	-0,212	0,13935	0	GSPATT00019888001	1,012	0,00029	1
GSPATT00000615001	0,04	0,80664	0	GSPATT00019898001	0,291	0,35823	0
GSPATT00000619001	0,52	0,00518	0	GSPATT00019899001	1,302	0,01819	1
GSPATT00000630001	0,066	0,80692	0	GSPATT00019918001	1,36	0,00661	1
GSPATT00000664001	0,473	0,02458	0	GSPATT00019930001	-0,02	0,89979	0
GSPATT00000685001	0,283	0,12345	0	GSPATT00019935001	0,306	0,30175	0
GSPATT00000704001	1,505	0,00001	1	GSPATT00019962001	0,101	0,63424	0
GSPATT00000760001	1,018	0,00234	1	GSPATT00019970001	0,305	0,11022	0
GSPATT00000783001	0,956	0,007	0	GSPATT00019974001	0,112	0,7056	0
GSPATT00000795001	0,73	0,00542	0	GSPATT00019988001	-0,077	0,59245	0
GSPATT00000817001	-0,289	0,11652	0	GSPATT00020006001	0,694	0,0227	0
GSPATT00000822001	0,006	0,96587	0	GSPATT00020016001	-0,284	0,16066	0
GSPATT00000824001	1,895	0,00001	1	GSPATT00020018001	0,567	0,00877	0
GSPATT00000926001	-0,44	0,07676	0	GSPATT00020040001	0,426	0,13971	0
GSPATT00000934001	0,7	0,00134	0	GSPATT00020047001	0,719	0,02416	0
GSPATT00000950001	-0,039	0,85119	0	GSPATT00020051001	0,424	0,02871	0
GSPATT00000961001	1,244	0,00014	1	GSPATT00020056001	-0,263	0,09627	0
GSPATT00000985001	-0,053	0,78626	0	GSPATT00020061001	0,203	0,18858	0
GSPATT00001013001	0,18	0,3735	0	GSPATT00020081001	0,562	0,01428	0
GSPATT00001029001	0,351	0,04038	0	GSPATT00020083001	-0,007	0,96307	0

GSPATT00001071001	-1,676	0,00003	-1	GSPATT00020106001	0,617	0,04707	0
GSPATT00001077001	-1,834	0	-1	GSPATT00020133001	0,915	0,00052	0
GSPATT00001082001	0,802	0,00363	0	GSPATT00020136001	0,721	0,00761	0
GSPATT00001085001	-0,102	0,55835	0	GSPATT00020148001	0,628	0,00191	0
GSPATT00001088001	-1,002	0,00029	-1	GSPATT00020151001	0,152	0,25786	0
GSPATT00001107001	1,346	0,00005	1	GSPATT00020164001	0,049	0,79466	0
GSPATT00001118001	0,362	0,12047	0	GSPATT00020171001	0,019	0,89222	0
GSPATT00001133001	-0,118	0,39808	0	GSPATT00020175001	-0,408	0,07148	0
GSPATT00001155001	-0,688	0,04688	0	GSPATT00020181001	-0,28	0,08622	0
GSPATT00001162001	-0,208	0,17385	0	GSPATT00020183001	0,379	0,08436	0
GSPATT00001183001	-1,126	0,00625	-1	GSPATT00020206001	1,187	0,00008	1
GSPATT00001189001	-0,09	0,61561	0	GSPATT00020213001	-1,901	0	-1
GSPATT00001201001	-1,126	0,00014	-1	GSPATT00020222001	0,022	0,8861	0
GSPATT00001207001	0,62	0,00301	0	GSPATT00020225001	0,491	0,02758	0
GSPATT00001216001	-0,859	0,0049	0	GSPATT00020239001	-0,066	0,6879	0
GSPATT00001230001	0,185	0,20391	0	GSPATT00020280001	0,417	0,00934	0
GSPATT00001255001	-0,19	0,27269	0	GSPATT00020292001	0,983	0,00426	0
GSPATT00001260001	1,21	0,00035	1	GSPATT00020315001	0,421	0,02041	0
GSPATT00001268001	-0,542	0,00397	0	GSPATT00020317001	1,39	0,00016	1
GSPATT00001273001	0,742	0,00126	0	GSPATT00020328001	0,132	0,45889	0
GSPATT00001279001	0,193	0,33618	0	GSPATT00020353001	0,488	0,03007	0
GSPATT00001292001	-0,196	0,32171	0	GSPATT00020354001	0,971	0,00377	0
GSPATT00001310001	0,604	0,00221	0	GSPATT00020357001	0,423	0,11371	0
GSPATT00001314001	0,32	0,07355	0	GSPATT00020375001	0,159	0,26389	0
GSPATT00001320001	0,724	0,00683	0	GSPATT00020379001	0,528	0,00684	0
GSPATT00001342001	0,712	0,00317	0	GSPATT00020390001	-1,113	0,00014	-1
GSPATT00001359001	0,036	0,83426	0	GSPATT00020397001	0,42	0,02881	0
GSPATT00001361001	0,243	0,21121	0	GSPATT00020442001	0,565	0,009	0
GSPATT00001369001	0,226	0,16319	0	GSPATT00020450001	0,494	0,01321	0
GSPATT00001370001	0,219	0,1963	0	GSPATT00020501001	0,141	0,29138	0
GSPATT00001413001	-0,893	0,00057	0	GSPATT00020577001	0,213	0,12758	0
GSPATT00001414001	0,294	0,08325	0	GSPATT00020591001	0,653	0,02277	0
GSPATT00001427001	0,007	0,96536	0	GSPATT00020594001	0,125	0,38102	0
GSPATT00001430001	0,427	0,01633	0	GSPATT00020603001	0,424	0,03461	0
GSPATT00001441001	-0,411	0,0844	0	GSPATT00020607001	0,553	0,03187	0
GSPATT00001463001	1,174	0,00247	1	GSPATT00020634001	-0,891	0,00039	0
GSPATT00001467001	-0,474	0,02385	0	GSPATT00020656001	0,384	0,09254	0
GSPATT00001471001	0,442	0,16312	0	GSPATT00020671001	0,4	0,06339	0
GSPATT00001479001	0,193	0,3499	0	GSPATT00020675001	0,023	0,86196	0
GSPATT00001494001	-0,073	0,59749	0	GSPATT00020696001	1,227	0,00012	1
GSPATT00001504001	0,05	0,75727	0	GSPATT00020706001	-2,177	0,00005	-1
GSPATT00001508001	0,42	0,01888	0	GSPATT00020718001	0,498	0,0293	0
GSPATT00001520001	-0,24	0,08374	0	GSPATT00020723001	1,447	0,00002	1
GSPATT00001527001	0,128	0,47689	0	GSPATT00020732001	0,346	0,02628	0
GSPATT00001554001	0,882	0,00038	0	GSPATT00020736001	-0,014	0,93682	0
GSPATT00001563001	-0,431	0,02903	0	GSPATT00020738001	-0,47	0,01442	0
GSPATT00001592001	1,698	0,00001	1	GSPATT00020743001	0,121	0,41116	0
GSPATT00001610001	0,834	0,00147	0	GSPATT00020748001	0,638	0,0469	0
GSPATT00001650001	0,32	0,08832	0	GSPATT00020754001	0,328	0,15362	0
GSPATT00001662001	0,438	0,17856	0	GSPATT00020755001	1,124	0,00008	1
GSPATT00001682001	-2,067	0,00007	-1	GSPATT00020757001	0,109	0,4162	0
GSPATT00001698001	-0,612	0,00264	0	GSPATT00020787001	0,074	0,69474	0
GSPATT00001741001	0,068	0,70586	0	GSPATT00020798001	-0,359	0,06684	0
GSPATT00001746001	1,163	0,00005	1	GSPATT00020807001	0,453	0,03103	0
GSPATT00001753001	0,102	0,57872	0	GSPATT00020812001	0,207	0,32984	0
GSPATT00001779001	0,661	0,00281	0	GSPATT00020816001	-0,014	0,954	0
GSPATT00001789001	0,667	0,00963	0	GSPATT00020817001	0,81	0,00504	0
GSPATT00001794001	-0,276	0,08218	0	GSPATT00020820001	0,296	0,15516	0
GSPATT00001840001	0,602	0,00734	0	GSPATT00020837001	-2,103	0	-1
GSPATT00001852001	1,949	0	1	GSPATT00020856001	0,607	0,00205	0
GSPATT00001853001	1,087	0,00026	1	GSPATT00020863001	1,067	0,00014	1
GSPATT00001854001	1,3	0,00007	1	GSPATT00020867001	-0,219	0,23135	0
GSPATT00001859001	0,31	0,07571	0	GSPATT00020886001	0,561	0,00314	0
GSPATT00001864001	0,864	0,00071	0	GSPATT00020887001	0,231	0,17424	0
GSPATT00001865001	0,55	0,00393	0	GSPATT00020897001	0,202	0,34688	0
GSPATT00001866001	0,294	0,40339	0	GSPATT00020921001	0,201	0,27627	0
GSPATT00001870001	0,431	0,01514	0	GSPATT00020942001	-0,24	0,19484	0

GSPATT00001873001	2,117	0,00001	1
GSPATT00001911001	-0,107	0,62983	0
GSPATT00001997001	-0,215	0,43497	0
GSPATT00002000001	0,994	0,00542	0
GSPATT00002008001	0,799	0,00219	0
GSPATT00002011001	0,938	0,00081	0
GSPATT00002013001	0,586	0,02656	0
GSPATT00002014001	0,546	0,01206	0
GSPATT00002052001	0,333	0,07203	0
GSPATT00002063001	0,381	0,02118	0
GSPATT00002065001	0,716	0,00677	0
GSPATT00002068001	1,376	0,00007	1
GSPATT00002077001	-0,063	0,72874	0
GSPATT00002091001	0,425	0,05442	0
GSPATT00002093001	1,052	0,00126	1
GSPATT00002094001	-0,116	0,53723	0
GSPATT00002096001	-0,076	0,75421	0
GSPATT00002107001	-0,282	0,23525	0
GSPATT00002108001	0,114	0,44232	0
GSPATT00002114001	0,469	0,04573	0
GSPATT00002115001	0,601	0,02057	0
GSPATT00002129001	1,065	0,00431	1
GSPATT00002132001	0,587	0,01286	0
GSPATT00002156001	0,933	0,0007	0
GSPATT00002182001	1,186	0,00008	1
GSPATT00002186001	0,08	0,52966	0
GSPATT00002191001	1,024	0,00219	1
GSPATT00002195001	-0,541	0,01959	0
GSPATT00002221001	0,238	0,09743	0
GSPATT00002241001	-0,266	0,23687	0
GSPATT00002247001	0,656	0,00298	0
GSPATT00002269001	-2,667	0,00001	-1
GSPATT00002272001	0,482	0,00978	0
GSPATT00002284001	0,447	0,0176	0
GSPATT00002285001	0,846	0,02305	0
GSPATT00002295001	0,216	0,24021	0
GSPATT00002297001	0,322	0,10354	0
GSPATT00002321001	0,196	0,2005	0
GSPATT00002371001	1,279	0,00502	1
GSPATT00002396001	0,89	0,00446	0
GSPATT00002397001	0,65	0,00105	0
GSPATT00002398001	0,608	0,00382	0
GSPATT00002410001	0,268	0,0869	0
GSPATT00002424001	-0,521	0,01826	0
GSPATT00002429001	0,235	0,11218	0
GSPATT00002438001	0,165	0,29594	0
GSPATT00002465001	0,13	0,62332	0
GSPATT00002480001	1,183	0,00004	1
GSPATT00002495001	0,174	0,40222	0
GSPATT00002529001	1,935	0	1
GSPATT00002540001	0,687	0,01064	0
GSPATT00002550001	-0,042	0,75069	0
GSPATT00002553001	0,322	0,07357	0
GSPATT00002575001	0,758	0,01395	0
GSPATT00002585001	0,436	0,08223	0
GSPATT00002589001	0,394	0,03041	0
GSPATT00002593001	1,076	0,0001	1
GSPATT00002595001	0,321	0,0482	0
GSPATT00002614001	-0,048	0,8212	0
GSPATT00002618001	0,222	0,2786	0
GSPATT00002633001	0,209	0,22321	0
GSPATT00002634001	-0,559	0,00848	0
GSPATT00002648001	-0,019	0,91239	0
GSPATT00002669001	0,347	0,04238	0
GSPATT00002670001	0,301	0,22471	0
GSPATT00002680001	1,006	0,00039	1
GSPATT00002682001	0,464	0,04413	0
GSPATT00020968001	1,383	0,00001	1
GSPATT00021004001	-0,039	0,80561	0
GSPATT00021006001	-0,917	0,00215	0
GSPATT00021015001	-0,087	0,60216	0
GSPATT00021053001	0,674	0,02023	0
GSPATT00021056001	-0,152	0,53854	0
GSPATT00021078001	0,339	0,1892	0
GSPATT00021080001	-0,038	0,88109	0
GSPATT00021083001	1,214	0,00004	1
GSPATT00021094001	0,597	0,01141	0
GSPATT00021104001	1,014	0,00009	1
GSPATT00021108001	0,03	0,85909	0
GSPATT00021127001	0,409	0,03217	0
GSPATT00021152001	0,566	0,00438	0
GSPATT00021175001	0,819	0,00079	0
GSPATT00021195001	1,22	0,0001	1
GSPATT00021201001	0,446	0,02497	0
GSPATT00021207001	0,704	0,01544	0
GSPATT00021213001	-0,327	0,35092	0
GSPATT00021222001	-1,069	0,00073	-1
GSPATT00021227001	0,331	0,04285	0
GSPATT00021248001	-0,222	0,12494	0
GSPATT00021249001	-0,183	0,27756	0
GSPATT00021250001	0,181	0,35664	0
GSPATT00021252001	2,647	0	1
GSPATT00021256001	0,688	0,00291	0
GSPATT00021258001	1,026	0,00012	1
GSPATT00021270001	0,517	0,01282	0
GSPATT00021271001	0,429	0,01704	0
GSPATT00021273001	1,843	0	1
GSPATT00021274001	1,19	0,00002	1
GSPATT00021275001	1,322	0,00075	1
GSPATT00021294001	-0,03	0,86443	0
GSPATT00021304001	-0,938	0,00125	0
GSPATT00021337001	1,12	0,00026	1
GSPATT00021346001	0,927	0,00175	0
GSPATT00021382001	0,681	0,00268	0
GSPATT00021397001	-0,341	0,04105	0
GSPATT00021403001	0,008	0,96802	0
GSPATT00021407001	0,546	0,01042	0
GSPATT00021410001	0,546	0,03562	0
GSPATT00021416001	0,262	0,11135	0
GSPATT00021423001	0,41	0,01616	0
GSPATT00021453001	0,568	0,01115	0
GSPATT00021496001	-0,121	0,56931	0
GSPATT00021511001	0,607	0,01408	0
GSPATT00021521001	0,151	0,3119	0
GSPATT00021522001	0,222	0,20683	0
GSPATT00021531001	1,595	0	1
GSPATT00021532001	0,469	0,01357	0
GSPATT00021537001	0,203	0,36082	0
GSPATT00021547001	0,33	0,05213	0
GSPATT00021550001	0,69	0,00115	0
GSPATT00021566001	0,15	0,38869	0
GSPATT00021592001	0,574	0,00334	0
GSPATT00021605001	-0,285	0,07981	0
GSPATT00021623001	-0,006	0,97564	0
GSPATT00021625001	-0,609	0,00663	0
GSPATT00021637001	0,858	0,0012	0
GSPATT00021639001	1,696	0,00085	1
GSPATT00021643001	0,057	0,70009	0
GSPATT00021645001	0,312	0,06296	0
GSPATT00021666001	-0,927	0,0001	0
GSPATT00021705001	0,593	0,01376	0
GSPATT00021709001	1,146	0,00013	1
GSPATT00021737001	2,607	0,00001	1
GSPATT00021745001	-0,479	0,00827	0

GSPATT00002705001	0,629	0,00714	0	GSPATT00021748001	0,772	0,0086	0
GSPATT00002720001	-1,253	0,00068	-1	GSPATT00021749001	1,147	0,01618	1
GSPATT00002743001	-0,488	0,03306	0	GSPATT00021752001	0,314	0,06862	0
GSPATT00002752001	0,495	0,06805	0	GSPATT00021753001	0,418	0,07228	0
GSPATT00002760001	0,788	0,00112	0	GSPATT00021754001	0,243	0,14094	0
GSPATT00002771001	0,859	0,0012	0	GSPATT00021761001	0,639	0,00204	0
GSPATT00002776001	-0,859	0,00025	0	GSPATT00021763001	1,74	0,00004	1
GSPATT00002799001	-0,188	0,27826	0	GSPATT00021786001	0,144	0,35607	0
GSPATT00002813001	0,189	0,28818	0	GSPATT00021789001	1,156	0,00096	1
GSPATT00002824001	-0,251	0,09825	0	GSPATT00021797001	0,664	0,0087	0
GSPATT00002833001	-0,306	0,26468	0	GSPATT00021815001	0,094	0,7025	0
GSPATT00002839001	-0,591	0,0576	0	GSPATT00021818001	0,452	0,0106	0
GSPATT00002865001	-0,375	0,08216	0	GSPATT00021839001	-0,624	0,00196	0
GSPATT00002890001	-1,023	0,00005	-1	GSPATT00021843001	-0,573	0,01408	0
GSPATT00002908001	1,161	0,00015	1	GSPATT00021861001	-2,393	0,00001	-1
GSPATT00002917001	-0,703	0,00355	0	GSPATT00021876001	0,606	0,05246	0
GSPATT00002933001	-2,484	0	-1	GSPATT00021880001	0,418	0,06147	0
GSPATT00002954001	-0,433	0,0165	0	GSPATT00021882001	-0,509	0,02829	0
GSPATT00002960001	-0,423	0,06926	0	GSPATT00021885001	0,508	0,01902	0
GSPATT00002969001	-0,585	0,02587	0	GSPATT00021893001	1,556	0,00001	1
GSPATT00002999001	1,266	0,00041	1	GSPATT00021905001	0,176	0,26244	0
GSPATT00003037001	0,383	0,02776	0	GSPATT00021907001	-0,742	0,0007	0
GSPATT00003054001	0,076	0,56297	0	GSPATT00021930001	-0,548	0,007	0
GSPATT00003075001	0,629	0,02375	0	GSPATT00021935001	0,444	0,0187	0
GSPATT00003077001	-0,196	0,20764	0	GSPATT00021944001	-1,181	0,00069	-1
GSPATT00003089001	-0,441	0,11477	0	GSPATT00021946001	0,249	0,20733	0
GSPATT00003090001	-0,149	0,32277	0	GSPATT00021955001	1,15	0,00027	1
GSPATT00003094001	0,49	0,01226	0	GSPATT00021964001	0,822	0,01015	0
GSPATT00003105001	1,596	0,00013	1	GSPATT00021978001	-0,527	0,00915	0
GSPATT00003123001	0,138	0,52706	0	GSPATT00021986001	1,819	0,00001	1
GSPATT00003132001	0,679	0,00156	0	GSPATT00022016001	0,205	0,15716	0
GSPATT00003154001	0,332	0,08672	0	GSPATT00022037001	0,79	0,00181	0
GSPATT00003156001	0,696	0,00226	0	GSPATT00022042001	0,34	0,05228	0
GSPATT00003167001	0,084	0,63843	0	GSPATT00022061001	-0,298	0,11655	0
GSPATT00003168001	1,73	0,00037	1	GSPATT00022128001	-0,88	0,0016	0
GSPATT00003185001	0,267	0,31747	0	GSPATT00022131001	-0,083	0,72879	0
GSPATT00003191001	-0,442	0,0623	0	GSPATT00022141001	1,027	0,00014	1
GSPATT00003192001	-0,143	0,32291	0	GSPATT00022154001	0,346	0,07096	0
GSPATT00003194001	1,519	0,00002	1	GSPATT00022155001	0,511	0,00951	0
GSPATT00003198001	0,506	0,02601	0	GSPATT00022167001	0,329	0,07157	0
GSPATT00003200001	0,421	0,03094	0	GSPATT00022172001	0,608	0,01725	0
GSPATT00003201001	0,867	0,00568	0	GSPATT00022183001	-0,244	0,23912	0
GSPATT00003224001	-0,237	0,12454	0	GSPATT00022205001	0,366	0,11152	0
GSPATT00003229001	0,103	0,49036	0	GSPATT00022214001	0,659	0,00671	0
GSPATT00003230001	0,337	0,26038	0	GSPATT00022218001	-0,466	0,01328	0
GSPATT00003236001	1,111	0,00005	1	GSPATT00022236001	1,845	0,00007	1
GSPATT00003248001	-0,663	0,0009	0	GSPATT00022240001	0,698	0,0019	0
GSPATT00003253001	0,103	0,43872	0	GSPATT00022249001	0,454	0,00814	0
GSPATT00003279001	0,867	0,0006	0	GSPATT00022251001	-0,514	0,08066	0
GSPATT00003280001	-0,04	0,80148	0	GSPATT00022256001	0,89	0,0088	0
GSPATT00003283001	0,264	0,25078	0	GSPATT00022278001	0,113	0,6617	0
GSPATT00003292001	0,915	0,00467	0	GSPATT00022287001	0,434	0,03622	0
GSPATT00003309001	-0,973	0,0002	0	GSPATT00022290001	1,407	0,00034	1
GSPATT00003326001	-0,051	0,69934	0	GSPATT00022296001	1,798	0,00001	1
GSPATT00003335001	-0,087	0,60586	0	GSPATT00022304001	0,901	0,00027	0
GSPATT00003339001	0,266	0,15758	0	GSPATT00022316001	0,995	0,0013	0
GSPATT00003344001	0,549	0,00644	0	GSPATT00022319001	-0,149	0,47401	0
GSPATT00003348001	-0,128	0,5085	0	GSPATT00022364001	0,012	0,94018	0
GSPATT00003366001	0,829	0,00229	0	GSPATT00022375001	0,335	0,05369	0
GSPATT00003371001	0,268	0,11218	0	GSPATT00022376001	0,282	0,36231	0
GSPATT00003407001	0,787	0,00042	0	GSPATT00022389001	0,267	0,0861	0
GSPATT00003425001	-0,517	0,01811	0	GSPATT00022398001	-0,303	0,11489	0
GSPATT00003486001	0,113	0,47243	0	GSPATT00022403001	0,733	0,00613	0
GSPATT00003501001	0,158	0,34108	0	GSPATT00022415001	0,64	0,00116	0
GSPATT00003507001	-1,565	0,00001	-1	GSPATT00022426001	0,639	0,00648	0
GSPATT00003528001	0,505	0,01812	0	GSPATT00022436001	-0,065	0,75967	0
GSPATT00003535001	0,273	0,06967	0	GSPATT00022478001	-0,264	0,12121	0

GSPATT00003620001	0,88	0,00103	0	GSPATT00022479001	-0,83	0,0106	0
GSPATT00003647001	0,327	0,21564	0	GSPATT00022490001	0,953	0,00048	0
GSPATT00003651001	0,62	0,00494	0	GSPATT00022560001	0,407	0,01304	0
GSPATT00003653001	-0,158	0,36535	0	GSPATT00022581001	0,197	0,34705	0
GSPATT00003657001	-0,746	0,00329	0	GSPATT00022585001	-0,137	0,38681	0
GSPATT00003674001	0,666	0,18862	0	GSPATT00022588001	0,519	0,01581	0
GSPATT00003681001	-0,103	0,4827	0	GSPATT00022625001	-0,631	0,00225	0
GSPATT00003682001	1,134	0,00047	1	GSPATT00022648001	1,169	0,00006	1
GSPATT00003692001	0,249	0,08305	0	GSPATT00022652001	0,216	0,20385	0
GSPATT00003699001	-0,208	0,19088	0	GSPATT00022660001	0,792	0,00748	0
GSPATT00003701001	-1,324	0,00003	-1	GSPATT00022665001	0,156	0,24856	0
GSPATT00003711001	-0,057	0,78591	0	GSPATT00022666001	1,059	0,00175	1
GSPATT00003719001	-2,965	0	-1	GSPATT00022671001	0,453	0,06689	0
GSPATT00003722001	-0,214	0,14285	0	GSPATT00022672001	0,392	0,0156	0
GSPATT00003724001	-1,06	0,00023	-1	GSPATT00022676001	0,956	0,00118	0
GSPATT00003735001	0,938	0,00036	0	GSPATT00022680001	0,88	0,01013	0
GSPATT00003784001	-0,762	0,00155	0	GSPATT00022747001	-0,042	0,84234	0
GSPATT00003800001	0,707	0,00325	0	GSPATT00022758001	-0,264	0,37366	0
GSPATT00003805001	-0,458	0,01837	0	GSPATT00022760001	-0,332	0,06229	0
GSPATT00003808001	-1,655	0,00001	-1	GSPATT00022772001	0,587	0,00225	0
GSPATT00003833001	0,314	0,13153	0	GSPATT00022792001	0,634	0,00443	0
GSPATT00003834001	0,118	0,39846	0	GSPATT00022794001	-0,642	0,00449	0
GSPATT00003866001	0,17	0,22326	0	GSPATT00022804001	0,571	0,02711	0
GSPATT00003883001	1,07	0,00005	1	GSPATT00022805001	0,721	0,00384	0
GSPATT00003889001	1,021	0,00011	1	GSPATT00022813001	-0,287	0,25917	0
GSPATT00003892001	-0,172	0,33783	0	GSPATT00022862001	0,196	0,23874	0
GSPATT00003941001	-0,015	0,90753	0	GSPATT00022877001	0,756	0,0172	0
GSPATT00003946001	0,029	0,8323	0	GSPATT00022923001	0,087	0,54996	0
GSPATT00003963001	0,533	0,01029	0	GSPATT00022959001	1,261	0,00015	1
GSPATT00003987001	0,889	0,00018	0	GSPATT00022960001	-0,158	0,24845	0
GSPATT00004011001	0,459	0,0181	0	GSPATT00022980001	0,614	0,0183	0
GSPATT00004041001	-2,104	0,00001	-1	GSPATT00022982001	0,79	0,04014	0
GSPATT00004054001	0,449	0,01063	0	GSPATT00022998001	0,587	0,0075	0
GSPATT00004057001	-0,473	0,03309	0	GSPATT00023007001	0,391	0,03401	0
GSPATT00004059001	0,026	0,88876	0	GSPATT00023011001	0,775	0,00132	0
GSPATT00004060001	0,495	0,02651	0	GSPATT00023034001	-0,277	0,07151	0
GSPATT00004091001	0,299	0,07171	0	GSPATT00023053001	0,174	0,19072	0
GSPATT00004095001	-0,095	0,61368	0	GSPATT00023054001	0,175	0,21904	0
GSPATT00004114001	0,269	0,09228	0	GSPATT00023076001	0,772	0,00846	0
GSPATT00004132001	-0,103	0,69251	0	GSPATT00023086001	0,21	0,20719	0
GSPATT00004152001	0,206	0,29595	0	GSPATT00023101001	0,252	0,21939	0
GSPATT00004205001	-0,31	0,17947	0	GSPATT00023116001	0,243	0,2874	0
GSPATT00004210001	0,416	0,03288	0	GSPATT00023168001	-0,346	0,10457	0
GSPATT00004223001	0,612	0,00738	0	GSPATT00023209001	0,505	0,02572	0
GSPATT00004225001	0,089	0,74057	0	GSPATT00023226001	-0,09	0,6793	0
GSPATT00004229001	-0,224	0,29312	0	GSPATT00023238001	-0,52	0,00932	0
GSPATT00004247001	0,159	0,57122	0	GSPATT00023249001	0,903	0,00029	0
GSPATT00004268001	1,307	0,00011	1	GSPATT00023269001	-0,248	0,34866	0
GSPATT00004273001	-0,717	0,00174	0	GSPATT00023282001	-0,358	0,04566	0
GSPATT00004298001	0,13	0,50582	0	GSPATT00023287001	-0,508	0,01292	0
GSPATT00004311001	0,568	0,00775	0	GSPATT00023291001	-0,368	0,10871	0
GSPATT00004321001	0,151	0,6363	0	GSPATT00023309001	-0,41	0,01263	0
GSPATT00004366001	0,068	0,69186	0	GSPATT00023311001	0,38	0,03477	0
GSPATT00004368001	0,323	0,16554	0	GSPATT00023324001	0,902	0,00067	0
GSPATT00004374001	1,795	0	1	GSPATT00023325001	0,851	0,00031	0
GSPATT00004376001	0,343	0,08104	0	GSPATT00023332001	0,059	0,7739	0
GSPATT00004396001	0,315	0,11278	0	GSPATT00023391001	0,729	0,00128	0
GSPATT00004405001	0,153	0,39785	0	GSPATT00023409001	-0,413	0,17182	0
GSPATT00004412001	-1,59	0,00001	-1	GSPATT00023417001	0,386	0,08109	0
GSPATT00004442001	-0,201	0,48124	0	GSPATT00023422001	-0,559	0,02664	0
GSPATT00004447001	0,383	0,05161	0	GSPATT00023426001	-0,016	0,92617	0
GSPATT00004496001	0,955	0,00187	0	GSPATT00023427001	0,645	0,027	0
GSPATT00004514001	0,476	0,02673	0	GSPATT00023429001	0,253	0,18096	0
GSPATT00004528001	0,66	0,00397	0	GSPATT00023436001	0,755	0,04632	0
GSPATT00004559001	0,711	0,00505	0	GSPATT00023438001	0,035	0,83414	0
GSPATT00004570001	0,195	0,22266	0	GSPATT00023469001	-0,011	0,95408	0
GSPATT00004575001	-0,004	0,97788	0	GSPATT00023488001	1,084	0,00116	1

GSPATT00004580001	1,242	0,00014	1	GSPATT00023514001	0,507	0,00757	0
GSPATT00004590001	0,499	0,01576	0	GSPATT00023531001	0,22	0,46511	0
GSPATT00004598001	0,48	0,02633	0	GSPATT00023540001	0,273	0,10052	0
GSPATT00004600001	0,083	0,57831	0	GSPATT00023549001	-1,224	0,00167	-1
GSPATT00004601001	1,962	0,0001	1	GSPATT00023569001	-0,979	0,00048	0
GSPATT00004610001	0,945	0,00014	0	GSPATT00023571001	-0,22	0,33947	0
GSPATT00004630001	-0,933	0,00013	0	GSPATT00023576001	-0,089	0,51115	0
GSPATT00004644001	-2,245	0,00001	-1	GSPATT00023613001	0,192	0,1796	0
GSPATT00004661001	-0,088	0,65447	0	GSPATT00023618001	-0,03	0,8305	0
GSPATT00004664001	0,401	0,16244	0	GSPATT00023627001	0,203	0,23093	0
GSPATT00004681001	-0,284	0,11177	0	GSPATT00023631001	0,239	0,10083	0
GSPATT00004693001	0,654	0,00623	0	GSPATT00023632001	0,122	0,36366	0
GSPATT00004708001	-0,936	0,00106	0	GSPATT00023667001	-0,421	0,04545	0
GSPATT00004716001	-0,265	0,1283	0	GSPATT00023676001	0,145	0,31782	0
GSPATT00004725001	-0,044	0,79332	0	GSPATT00023690001	-1,078	0,0043	-1
GSPATT00004760001	0,235	0,3263	0	GSPATT00023719001	-0,472	0,04584	0
GSPATT00004761001	0,808	0,01968	0	GSPATT00023728001	0,102	0,45091	0
GSPATT00004764001	-0,15	0,28228	0	GSPATT00023729001	0,5	0,071	0
GSPATT00004777001	1,749	0,00006	1	GSPATT00023735001	0,573	0,00261	0
GSPATT00004785001	0,782	0,00521	0	GSPATT00023790001	-0,309	0,06093	0
GSPATT00004807001	0,124	0,60039	0	GSPATT00023792001	0,035	0,82314	0
GSPATT00004813001	0,679	0,01555	0	GSPATT00023807001	-0,378	0,04035	0
GSPATT00004832001	-0,277	0,07921	0	GSPATT00023823001	-0,725	0,00571	0
GSPATT00004834001	0,789	0,00098	0	GSPATT00023843001	0,248	0,16073	0
GSPATT00004846001	2,635	0	1	GSPATT00023861001	1,352	0,00004	1
GSPATT00004860001	-0,019	0,91882	0	GSPATT00023871001	-0,181	0,31269	0
GSPATT00004865001	-0,645	0,01547	0	GSPATT00023901001	0,661	0,00244	0
GSPATT00004869001	0,682	0,00693	0	GSPATT00023907001	0,749	0,00073	0
GSPATT00004874001	-0,141	0,55683	0	GSPATT00023937001	-0,496	0,07124	0
GSPATT00004901001	0,489	0,04492	0	GSPATT00023947001	0,21	0,19097	0
GSPATT00004905001	0,24	0,28818	0	GSPATT00023951001	0,462	0,01336	0
GSPATT00004906001	0,041	0,80854	0	GSPATT00023962001	-0,49	0,01746	0
GSPATT00004925001	-0,765	0,01316	0	GSPATT00023981001	0,056	0,75794	0
GSPATT00004954001	-0,94	0,02171	0	GSPATT00024046001	0,488	0,06203	0
GSPATT00004968001	0,709	0,00121	0	GSPATT00024078001	0,879	0,0027	0
GSPATT00004976001	-0,023	0,86985	0	GSPATT00024080001	0,962	0,00084	0
GSPATT00004988001	-0,207	0,1401	0	GSPATT00024093001	0,589	0,00369	0
GSPATT00004998001	0,877	0,0004	0	GSPATT00024120001	0,414	0,05081	0
GSPATT00005003001	0,289	0,06439	0	GSPATT00024158001	0,676	0,00939	0
GSPATT00005007001	0,56	0,00815	0	GSPATT00024181001	0,193	0,36401	0
GSPATT00005009001	0,54	0,00742	0	GSPATT00024182001	-0,819	0,01549	0
GSPATT00005029001	0,213	0,14145	0	GSPATT00024183001	-0,176	0,34939	0
GSPATT00005077001	0,271	0,1489	0	GSPATT00024185001	1,039	0,00005	1
GSPATT00005093001	0,879	0,00087	0	GSPATT00024203001	0,349	0,11648	0
GSPATT00005095001	0,782	0,00596	0	GSPATT00024205001	0,66	0,00203	0
GSPATT00005098001	0,976	0,00298	0	GSPATT00024206001	-0,177	0,2138	0
GSPATT00005100001	-0,27	0,06839	0	GSPATT00024209001	1,188	0,00003	1
GSPATT00005117001	0,136	0,40124	0	GSPATT00024218001	-0,321	0,04683	0
GSPATT00005120001	0,8	0,00223	0	GSPATT00024233001	0,949	0,0003	0
GSPATT00005130001	-0,006	0,97738	0	GSPATT00024234001	0,863	0,00016	0
GSPATT00005209001	1,585	0,00011	1	GSPATT00024244001	0,136	0,47521	0
GSPATT00005243001	1,145	0,0018	1	GSPATT00024253001	0,327	0,12765	0
GSPATT00005245001	0,407	0,07978	0	GSPATT00024274001	0,022	0,87189	0
GSPATT00005247001	-0,102	0,43462	0	GSPATT00024309001	-0,157	0,28831	0
GSPATT00005248001	-1,692	0,00037	-1	GSPATT00024335001	-0,34	0,04551	0
GSPATT00005269001	0,265	0,06576	0	GSPATT00024337001	0,437	0,02363	0
GSPATT00005271001	-0,185	0,35608	0	GSPATT00024348001	-0,075	0,56089	0
GSPATT00005274001	0,558	0,00601	0	GSPATT00024349001	0,096	0,597	0
GSPATT00005284001	0,206	0,14532	0	GSPATT00024352001	0,614	0,00859	0
GSPATT00005295001	0,7	0,00631	0	GSPATT00024357001	0,148	0,45854	0
GSPATT00005301001	0,612	0,02995	0	GSPATT00024361001	0,391	0,03397	0
GSPATT00005326001	1,016	0,00028	1	GSPATT00024377001	1,043	0,00342	1
GSPATT00005329001	0,523	0,01314	0	GSPATT00024390001	-0,282	0,13976	0
GSPATT00005343001	-0,049	0,73244	0	GSPATT00024398001	0,796	0,01758	0
GSPATT00005348001	0,239	0,13893	0	GSPATT00024407001	-0,816	0,004	0
GSPATT00005359001	-0,071	0,70866	0	GSPATT00024410001	0,428	0,02489	0
GSPATT00005382001	0,315	0,08827	0	GSPATT00024418001	0,221	0,13987	0

GSPATT00005394001	-2,476	0	-1	GSPATT00024424001	-0,29	0,0846	0
GSPATT00005416001	-0,283	0,0906	0	GSPATT00024429001	0,278	0,07692	0
GSPATT00005443001	1,201	0,01436	1	GSPATT00024448001	-0,278	0,17393	0
GSPATT00005458001	-0,786	0,00076	0	GSPATT00024450001	-0,469	0,00704	0
GSPATT00005479001	0,453	0,01604	0	GSPATT00024451001	0,393	0,03105	0
GSPATT00005498001	0,586	0,00534	0	GSPATT00024479001	-0,903	0,00303	0
GSPATT00005504001	-0,323	0,07461	0	GSPATT00024481001	0,435	0,1006	0
GSPATT00005531001	0,024	0,88617	0	GSPATT00024485001	-0,462	0,15535	0
GSPATT00005533001	-0,809	0,00171	0	GSPATT00024498001	0,272	0,35558	0
GSPATT00005536001	0,887	0,00254	0	GSPATT00024499001	0,56	0,00549	0
GSPATT00005555001	0,146	0,36762	0	GSPATT00024504001	-0,557	0,06748	0
GSPATT00005556001	0,344	0,07104	0	GSPATT00024512001	1,298	0,00001	1
GSPATT00005557001	0,873	0,00334	0	GSPATT00024514001	0,748	0,01065	0
GSPATT00005568001	0,44	0,03873	0	GSPATT00024536001	0,01	0,96698	0
GSPATT00005581001	0,156	0,32591	0	GSPATT00024539001	-2,028	0	-1
GSPATT00005587001	0,343	0,06896	0	GSPATT00024546001	-0,401	0,04144	0
GSPATT00005611001	0,102	0,61679	0	GSPATT00024564001	0,433	0,05933	0
GSPATT00005614001	0,401	0,02429	0	GSPATT00024565001	1,115	0,00008	1
GSPATT00005647001	-0,353	0,0216	0	GSPATT00024566001	0,629	0,00565	0
GSPATT00005692001	-0,035	0,79461	0	GSPATT00024591001	0,498	0,00429	0
GSPATT00005703001	0,426	0,05301	0	GSPATT00024598001	-0,675	0,01914	0
GSPATT00005726001	0,372	0,0779	0	GSPATT00024618001	0,097	0,49798	0
GSPATT00005733001	0,017	0,92434	0	GSPATT00024644001	0,654	0,00896	0
GSPATT00005734001	0,285	0,09036	0	GSPATT00024673001	-0,186	0,23745	0
GSPATT00005738001	0,978	0,00023	0	GSPATT00024691001	1,052	0,00127	1
GSPATT00005752001	1,537	0,00004	1	GSPATT00024731001	0,285	0,24363	0
GSPATT00005757001	0,742	0,00204	0	GSPATT00024737001	0,421	0,14567	0
GSPATT00005760001	-0,591	0,07206	0	GSPATT00024766001	0,352	0,11206	0
GSPATT00005782001	0,537	0,00527	0	GSPATT00024788001	0,153	0,34111	0
GSPATT00005787001	0,145	0,47364	0	GSPATT00024803001	0,179	0,50259	0
GSPATT00005804001	-2,483	0,00045	-1	GSPATT00024859001	1,158	0,00272	1
GSPATT00005811001	0,498	0,03959	0	GSPATT00024862001	0,232	0,28545	0
GSPATT00005814001	-0,041	0,82667	0	GSPATT00024885001	-1,562	0,00004	-1
GSPATT00005845001	0,153	0,32908	0	GSPATT00024889001	0,107	0,58905	0
GSPATT00005881001	0,784	0,01296	0	GSPATT00024895001	-0,166	0,43815	0
GSPATT00005902001	1,463	0,00021	1	GSPATT00024913001	-0,542	0,01406	0
GSPATT00005904001	0,174	0,28531	0	GSPATT00024916001	1,07	0,00006	1
GSPATT00005911001	0,788	0,00064	0	GSPATT00024917001	1,027	0,0002	1
GSPATT00005934001	0,631	0,00222	0	GSPATT00024942001	-0,918	0,00043	0
GSPATT00005943001	1,376	0,00047	1	GSPATT00024976001	0,84	0,00119	0
GSPATT00005951001	-0,034	0,81726	0	GSPATT00024990001	0,807	0,0027	0
GSPATT00005960001	1,544	0,00007	1	GSPATT00025030001	-0,48	0,01979	0
GSPATT00005995001	0,249	0,17873	0	GSPATT00025035001	1,087	0,00028	1
GSPATT00006007001	0,509	0,01301	0	GSPATT00025044001	0,229	0,26111	0
GSPATT00006011001	0,426	0,04714	0	GSPATT00025071001	-0,063	0,7592	0
GSPATT00006030001	0,381	0,05068	0	GSPATT00025076001	0,189	0,30656	0
GSPATT00006033001	0,654	0,0075	0	GSPATT00025088001	0,087	0,7199	0
GSPATT00006057001	-0,917	0,00035	0	GSPATT00025089001	0,396	0,07801	0
GSPATT00006075001	0,152	0,28079	0	GSPATT00025098001	0,473	0,02724	0
GSPATT00006080001	0,359	0,11534	0	GSPATT00025106001	0,702	0,01194	0
GSPATT00006094001	0,938	0,00214	0	GSPATT00025128001	0,313	0,11606	0
GSPATT00006105001	0,219	0,41076	0	GSPATT00025133001	0,871	0,00255	0
GSPATT00006125001	0,447	0,0539	0	GSPATT00025144001	1,216	0,00018	1
GSPATT00006174001	-1,189	0,00097	-1	GSPATT00025176001	0,858	0,01028	0
GSPATT00006199001	-0,122	0,43015	0	GSPATT00025177001	0,141	0,61521	0
GSPATT00006226001	-0,062	0,65778	0	GSPATT00025184001	0,545	0,03088	0
GSPATT00006272001	0,621	0,03899	0	GSPATT00025243001	1,014	0,00008	1
GSPATT00006280001	0,726	0,00083	0	GSPATT00025254001	0,909	0,00035	0
GSPATT00006296001	0,129	0,52836	0	GSPATT00025257001	0,582	0,02537	0
GSPATT00006297001	0,413	0,01659	0	GSPATT00025262001	-0,617	0,0118	0
GSPATT00006313001	0,809	0,01243	0	GSPATT00025273001	1,277	0,00074	1
GSPATT00006314001	0,607	0,00237	0	GSPATT00025348001	0,917	0,00082	0
GSPATT00006315001	0,211	0,21775	0	GSPATT00025382001	-0,664	0,00239	0
GSPATT00006331001	0,84	0,00169	0	GSPATT00025431001	0,45	0,04878	0
GSPATT00006334001	-0,044	0,80576	0	GSPATT00025434001	2,076	0	1
GSPATT00006378001	0,677	0,00317	0	GSPATT00025443001	0,397	0,05816	0
GSPATT00006379001	0,429	0,03699	0	GSPATT00025450001	0,01	0,9566	0

GSPATT00006390001	-3,443	0	-1	GSPATT00025459001	0,443	0,0356	0
GSPATT00006408001	-0,255	0,28163	0	GSPATT00025471001	-1,355	0,00017	-1
GSPATT00006415001	-0,218	0,29617	0	GSPATT00025473001	1,303	0,00076	1
GSPATT00006446001	-0,06	0,78799	0	GSPATT00025481001	-0,394	0,02039	0
GSPATT00006468001	1,644	0,00007	1	GSPATT00025487001	0,527	0,00492	0
GSPATT00006472001	-0,821	0,00056	0	GSPATT00025488001	0,117	0,44687	0
GSPATT00006519001	1,503	0,00026	1	GSPATT00025542001	0,154	0,32238	0
GSPATT00006522001	0,067	0,61368	0	GSPATT00025564001	0,335	0,03391	0
GSPATT00006526001	0,784	0,00476	0	GSPATT00025565001	0,102	0,44835	0
GSPATT00006544001	0,253	0,22418	0	GSPATT00025585001	0,189	0,32528	0
GSPATT00006575001	-0,075	0,7515	0	GSPATT00025594001	-3,032	0	-1
GSPATT00006576001	0,119	0,43904	0	GSPATT00025621001	0,549	0,02246	0
GSPATT00006580001	0,511	0,15256	0	GSPATT00025624001	1,872	0,00011	1
GSPATT00006581001	-0,025	0,86132	0	GSPATT00025626001	0,186	0,22873	0
GSPATT00006592001	0,443	0,14222	0	GSPATT00025634001	-0,572	0,0037	0
GSPATT00006594001	2,357	0	1	GSPATT00025641001	-0,136	0,41303	0
GSPATT00006615001	0,212	0,32587	0	GSPATT00025658001	0,311	0,08053	0
GSPATT00006642001	1,159	0,00005	1	GSPATT00025660001	0,423	0,01588	0
GSPATT00006653001	0,808	0,00058	0	GSPATT00025674001	-0,467	0,00665	0
GSPATT00006659001	-0,504	0,0224	0	GSPATT00025677001	0,628	0,0033	0
GSPATT00006681001	1,225	0,00002	1	GSPATT00025683001	1,103	0,00903	1
GSPATT00006690001	0,625	0,00374	0	GSPATT00025688001	0,986	0,00101	0
GSPATT00006702001	1,003	0,00049	1	GSPATT00025714001	-0,285	0,11387	0
GSPATT00006707001	0,096	0,55283	0	GSPATT00025724001	-0,118	0,54796	0
GSPATT00006718001	-1,206	0,0008	-1	GSPATT00025742001	0,095	0,63776	0
GSPATT00006721001	0,259	0,28365	0	GSPATT00025754001	1,693	0	1
GSPATT00006725001	1,26	0,00008	1	GSPATT00025790001	0,722	0,01137	0
GSPATT00006735001	1,208	0,00017	1	GSPATT00025829001	-0,303	0,13502	0
GSPATT00006746001	0,845	0,00197	0	GSPATT00025830001	-0,219	0,47777	0
GSPATT00006769001	0,213	0,32339	0	GSPATT00025833001	0,318	0,12793	0
GSPATT00006775001	0,248	0,09259	0	GSPATT00025835001	0,577	0,00646	0
GSPATT00006782001	0,148	0,27067	0	GSPATT00025843001	-0,234	0,1386	0
GSPATT00006852001	0,834	0,00426	0	GSPATT00025874001	0,078	0,61912	0
GSPATT00006853001	0,531	0,04301	0	GSPATT00025891001	0,036	0,78724	0
GSPATT00006854001	0,961	0,00034	0	GSPATT00025892001	0,813	0,00112	0
GSPATT00006864001	0,6	0,00924	0	GSPATT00025893001	0,184	0,26595	0
GSPATT00006884001	0,278	0,14598	0	GSPATT00025925001	-2,389	0,00001	-1
GSPATT00006904001	0,29	0,11816	0	GSPATT00025938001	0,244	0,21258	0
GSPATT00006927001	0,501	0,00669	0	GSPATT00025977001	0,266	0,20896	0
GSPATT00006928001	0,334	0,0274	0	GSPATT00025987001	0,777	0,00304	0
GSPATT00006948001	0,353	0,03894	0	GSPATT00025999001	0,214	0,18023	0
GSPATT00006949001	0,568	0,03228	0	GSPATT00026028001	-0,597	0,02174	0
GSPATT00006953001	0,189	0,35123	0	GSPATT00026044001	1,043	0,00011	1
GSPATT00006969001	0,405	0,11317	0	GSPATT00026048001	0,131	0,45105	0
GSPATT00006973001	-1,451	0,00032	-1	GSPATT00026052001	0,22	0,26869	0
GSPATT00006974001	0,738	0,04036	0	GSPATT00026100001	-0,727	0,05157	0
GSPATT00007015001	-0,04	0,8039	0	GSPATT00026117001	0,711	0,00252	0
GSPATT00007020001	0,699	0,0179	0	GSPATT00026130001	0,041	0,84926	0
GSPATT00007023001	0,031	0,83742	0	GSPATT00026158001	-0,019	0,89138	0
GSPATT00007028001	1,888	0,00035	1	GSPATT00026167001	0,538	0,00916	0
GSPATT00007036001	-1,628	0,00205	-1	GSPATT00026169001	0,593	0,0211	0
GSPATT00007037001	0,21	0,21843	0	GSPATT00026182001	0,405	0,01463	0
GSPATT00007044001	-0,041	0,75732	0	GSPATT00026208001	-0,305	0,05561	0
GSPATT00007068001	-0,215	0,22845	0	GSPATT00026214001	0,126	0,51347	0
GSPATT00007081001	-0,453	0,09098	0	GSPATT00026231001	-0,721	0,00236	0
GSPATT00007084001	1,343	0,0001	1	GSPATT00026239001	0,204	0,24495	0
GSPATT00007085001	0,898	0,00027	0	GSPATT00026269001	-0,827	0,00039	0
GSPATT00007086001	-0,397	0,12088	0	GSPATT00026274001	0,081	0,55749	0
GSPATT00007091001	1,465	0,00001	1	GSPATT00026292001	0,175	0,24434	0
GSPATT00007092001	0,27	0,19194	0	GSPATT00026323001	-0,937	0,00078	0
GSPATT00007095001	0,892	0,00182	0	GSPATT00026336001	-0,538	0,01977	0
GSPATT00007110001	0,57	0,00537	0	GSPATT00026341001	0,56	0,00614	0
GSPATT00007113001	0,035	0,79386	0	GSPATT00026346001	-0,833	0,00425	0
GSPATT00007130001	0,976	0,0003	0	GSPATT00026360001	1,582	0,00001	1
GSPATT00007151001	0,29	0,08359	0	GSPATT00026362001	0,039	0,7821	0
GSPATT00007162001	-0,046	0,76877	0	GSPATT00026366001	0,255	0,08585	0
GSPATT00007168001	2,307	0	1	GSPATT00026371001	0,968	0,00219	0

GSPATT00007170001	0,256	0,39221	0	GSPATT00026384001	0,13	0,49373	0
GSPATT00007176001	0,545	0,0517	0	GSPATT00026401001	0,404	0,01576	0
GSPATT00007193001	-0,121	0,39214	0	GSPATT00026410001	-1,354	0,00004	-1
GSPATT00007196001	0,471	0,05051	0	GSPATT00026424001	-0,195	0,41235	0
GSPATT00007206001	-0,243	0,15376	0	GSPATT00026435001	0,978	0,0003	0
GSPATT00007221001	-0,047	0,73213	0	GSPATT00026456001	-0,112	0,49348	0
GSPATT00007228001	-0,424	0,07621	0	GSPATT00026471001	0,689	0,0725	0
GSPATT00007243001	0,258	0,11177	0	GSPATT00026484001	0,101	0,56577	0
GSPATT00007288001	-0,479	0,02516	0	GSPATT00026498001	0,273	0,11056	0
GSPATT00007295001	1,711	0	1	GSPATT00026501001	0,573	0,02563	0
GSPATT00007306001	0,194	0,49349	0	GSPATT00026528001	0,335	0,1504	0
GSPATT00007307001	0,539	0,00329	0	GSPATT00026582001	-0,61	0,00172	0
GSPATT00007337001	0,377	0,05996	0	GSPATT00026598001	-0,409	0,02421	0
GSPATT00007343001	0,329	0,28753	0	GSPATT00026607001	0,518	0,00768	0
GSPATT00007357001	-0,154	0,51852	0	GSPATT00026611001	0,416	0,09592	0
GSPATT00007369001	-0,3	0,09662	0	GSPATT00026620001	-0,023	0,89953	0
GSPATT00007385001	0,031	0,87634	0	GSPATT00026642001	0,375	0,12827	0
GSPATT00007396001	0,747	0,00516	0	GSPATT00026656001	0,014	0,92729	0
GSPATT00007424001	0,679	0,0528	0	GSPATT00026672001	0,656	0,00939	0
GSPATT00007458001	-0,178	0,41913	0	GSPATT00026673001	1,475	0,00074	1
GSPATT00007469001	0,74	0,00278	0	GSPATT00026685001	-0,162	0,49003	0
GSPATT00007491001	0,204	0,26477	0	GSPATT00026711001	-1,004	0,00014	-1
GSPATT00007506001	3,323	0	1	GSPATT00026728001	0,421	0,03621	0
GSPATT00007552001	-0,289	0,16464	0	GSPATT00026733001	-0,183	0,2048	0
GSPATT00007558001	0,513	0,05289	0	GSPATT00026736001	0,014	0,9335	0
GSPATT00007569001	-0,059	0,72729	0	GSPATT00026737001	-0,589	0,01656	0
GSPATT00007633001	0,585	0,00705	0	GSPATT00026742001	1,681	0,00062	1
GSPATT00007636001	0,951	0,00013	0	GSPATT00026750001	0,269	0,07138	0
GSPATT00007651001	1,374	0,00001	1	GSPATT00026751001	0,217	0,23484	0
GSPATT00007652001	0,604	0,00281	0	GSPATT00026762001	0,081	0,72109	0
GSPATT00007655001	1,115	0,00032	1	GSPATT00026768001	1,3	0,0016	1
GSPATT00007660001	-2,547	0	-1	GSPATT00026770001	0,781	0,00036	0
GSPATT00007679001	-0,046	0,77776	0	GSPATT00026784001	0,447	0,02348	0
GSPATT00007689001	0,756	0,0049	0	GSPATT00026817001	-0,512	0,01273	0
GSPATT00007699001	0,698	0,00069	0	GSPATT00026853001	-0,021	0,90542	0
GSPATT00007760001	0,871	0,00045	0	GSPATT00026855001	-0,189	0,23205	0
GSPATT00007792001	0,379	0,04772	0	GSPATT00026879001	0,638	0,0089	0
GSPATT00007794001	-1,257	0,00002	-1	GSPATT00026882001	0,229	0,14149	0
GSPATT00007797001	0,346	0,07773	0	GSPATT00026883001	0,746	0,01254	0
GSPATT00007801001	0,358	0,05841	0	GSPATT00026903001	0,385	0,04693	0
GSPATT00007808001	0,931	0,00109	0	GSPATT00026918001	0,426	0,01186	0
GSPATT00007826001	0,485	0,01651	0	GSPATT00026931001	0,327	0,03994	0
GSPATT00007852001	0,616	0,00193	0	GSPATT00026977001	0,418	0,01985	0
GSPATT00007857001	1,41	0,00014	1	GSPATT00027082001	-0,14	0,42006	0
GSPATT00007860001	0,07	0,62078	0	GSPATT00027084001	-2,917	0	-1
GSPATT00007861001	0,494	0,03195	0	GSPATT00027094001	-0,238	0,23457	0
GSPATT00007899001	0,019	0,90526	0	GSPATT00027100001	0,499	0,06172	0
GSPATT00007911001	-0,929	0,02523	0	GSPATT00027115001	-0,008	0,97395	0
GSPATT00007917001	0,096	0,67711	0	GSPATT00027119001	0,768	0,00101	0
GSPATT00007919001	0,035	0,87791	0	GSPATT00027122001	-0,502	0,01341	0
GSPATT00007922001	0,835	0,01888	0	GSPATT00027138001	0,18	0,19509	0
GSPATT00007930001	-0,448	0,05141	0	GSPATT00027147001	-0,082	0,6483	0
GSPATT00007946001	1,164	0,00002	1	GSPATT00027149001	0,147	0,32384	0
GSPATT00007947001	0,256	0,17572	0	GSPATT00027156001	0,039	0,76929	0
GSPATT00007949001	1,323	0,00004	1	GSPATT00027189001	0,049	0,79446	0
GSPATT00007953001	0,179	0,38295	0	GSPATT00027203001	0,063	0,64969	0
GSPATT00007957001	0,151	0,34529	0	GSPATT00027205001	-0,221	0,24032	0
GSPATT00007961001	-0,294	0,05705	0	GSPATT00027222001	0,074	0,59482	0
GSPATT00007964001	-2,161	0,00001	-1	GSPATT00027241001	0,321	0,05768	0
GSPATT00007966001	0,728	0,03307	0	GSPATT00027257001	0,806	0,00465	0
GSPATT00007975001	0,396	0,04129	0	GSPATT00027270001	0,745	0,00887	0
GSPATT00007989001	0,044	0,84104	0	GSPATT00027312001	-1,048	0,00047	-1
GSPATT00007997001	0,533	0,00985	0	GSPATT00027355001	-0,308	0,10498	0
GSPATT00007998001	0,219	0,19699	0	GSPATT00027372001	1,25	0,00004	1
GSPATT00008011001	0,371	0,20891	0	GSPATT00027401001	0,365	0,07066	0
GSPATT00008014001	0,284	0,11593	0	GSPATT00027404001	-0,378	0,02671	0
GSPATT00008031001	-0,45	0,02946	0	GSPATT00027408001	0,032	0,82286	0

GSPATT00008074001	-0,457	0,03281	0	GSPATT00027410001	0,523	0,0081	0
GSPATT00008104001	1,297	0,00002	1	GSPATT00027442001	-0,097	0,65217	0
GSPATT00008134001	0,932	0,00055	0	GSPATT00027453001	-0,38	0,0322	0
GSPATT00008150001	0,063	0,64187	0	GSPATT00027465001	-1,097	0,00007	-1
GSPATT00008151001	0,2	0,21222	0	GSPATT00027470001	0,073	0,75047	0
GSPATT00008165001	0,565	0,00488	0	GSPATT00027479001	0,392	0,10569	0
GSPATT00008178001	0,755	0,00688	0	GSPATT00027483001	0,725	0,00101	0
GSPATT00008186001	-0,324	0,05436	0	GSPATT00027492001	1,241	0,00093	1
GSPATT00008216001	0,518	0,06627	0	GSPATT00027506001	0,183	0,46769	0
GSPATT00008228001	1,643	0,00002	1	GSPATT00027520001	-0,336	0,16994	0
GSPATT00008231001	-0,275	0,07499	0	GSPATT00027535001	0,825	0,01036	0
GSPATT00008235001	-0,069	0,63056	0	GSPATT00027546001	-0,383	0,03735	0
GSPATT00008243001	0,87	0,00126	0	GSPATT00027549001	0,322	0,09184	0
GSPATT00008313001	-3,997	0	-1	GSPATT00027607001	0,43	0,06985	0
GSPATT00008318001	0,769	0,02343	0	GSPATT00027613001	-0,299	0,09538	0
GSPATT00008324001	0,184	0,37151	0	GSPATT00027617001	-0,374	0,02379	0
GSPATT00008331001	0,617	0,11575	0	GSPATT00027623001	0,439	0,01037	0
GSPATT00008341001	0,816	0,00047	0	GSPATT00027637001	0,397	0,02895	0
GSPATT00008356001	0,219	0,14265	0	GSPATT00027641001	1,114	0,00049	1
GSPATT00008371001	0,43	0,01968	0	GSPATT00027648001	-0,304	0,04309	0
GSPATT00008373001	0,504	0,0091	0	GSPATT00027665001	0,667	0,04913	0
GSPATT00008385001	0,841	0,01833	0	GSPATT00027672001	-0,668	0,01828	0
GSPATT00008408001	-0,744	0,00043	0	GSPATT00027677001	0,078	0,65055	0
GSPATT00008410001	0,894	0,00015	0	GSPATT00027685001	0,812	0,00085	0
GSPATT00008418001	-0,272	0,07464	0	GSPATT00027690001	0,646	0,00592	0
GSPATT00008426001	-0,189	0,19536	0	GSPATT00027700001	0,396	0,14503	0
GSPATT00008447001	-0,614	0,00381	0	GSPATT00027727001	0,419	0,06587	0
GSPATT00008463001	1,266	0,00003	1	GSPATT00027731001	-0,101	0,74111	0
GSPATT00008472001	0,504	0,00647	0	GSPATT00027740001	0,305	0,04752	0
GSPATT00008497001	0,172	0,39591	0	GSPATT00027741001	0,59	0,00929	0
GSPATT00008503001	0,019	0,92472	0	GSPATT00027763001	0,328	0,03342	0
GSPATT00008512001	0,357	0,06391	0	GSPATT00027787001	0,369	0,04914	0
GSPATT00008527001	0,18	0,35417	0	GSPATT00027796001	-1,461	0,0001	-1
GSPATT00008573001	-0,003	0,98576	0	GSPATT00027807001	-2	0	-1
GSPATT00008596001	-1,074	0,00006	-1	GSPATT00027831001	1,076	0,00008	1
GSPATT00008602001	0,371	0,02113	0	GSPATT00027838001	0,488	0,03197	0
GSPATT00008611001	0,235	0,3889	0	GSPATT00027852001	-1,233	0,00015	-1
GSPATT00008619001	-0,021	0,87621	0	GSPATT00027857001	0,522	0,0062	0
GSPATT00008639001	-0,663	0,00927	0	GSPATT00027870001	-0,002	0,9924	0
GSPATT00008645001	0,599	0,05259	0	GSPATT00027898001	0,118	0,48905	0
GSPATT00008738001	0,821	0,00201	0	GSPATT00027906001	0,065	0,67546	0
GSPATT00008749001	-0,136	0,49358	0	GSPATT00027913001	0,404	0,09183	0
GSPATT00008764001	0,257	0,19576	0	GSPATT00027933001	-0,886	0,00125	0
GSPATT00008778001	0,342	0,04202	0	GSPATT00027938001	0,112	0,47766	0
GSPATT00008805001	0,579	0,00784	0	GSPATT00027978001	-0,102	0,58439	0
GSPATT00008830001	0,301	0,18862	0	GSPATT00027980001	-0,117	0,44254	0
GSPATT00008831001	0,097	0,54219	0	GSPATT00027986001	0,288	0,08477	0
GSPATT00008850001	0,887	0,00048	0	GSPATT00028006001	0,033	0,84856	0
GSPATT00008866001	0,383	0,07319	0	GSPATT00028020001	1,049	0,00011	1
GSPATT00008880001	-0,665	0,00165	0	GSPATT00028029001	1,144	0,00006	1
GSPATT00008893001	0,303	0,07004	0	GSPATT00028033001	0,014	0,94972	0
GSPATT00008897001	0,334	0,19577	0	GSPATT00028035001	1,108	0,00038	1
GSPATT00008901001	-1,237	0,00007	-1	GSPATT00028058001	-1,581	0,00253	-1
GSPATT00008924001	0,154	0,39308	0	GSPATT00028068001	0,819	0,00613	0
GSPATT00008975001	0,694	0,00205	0	GSPATT00028082001	0,851	0,00023	0
GSPATT00008986001	0,293	0,16667	0	GSPATT00028088001	0,595	0,00642	0
GSPATT00009001001	0,558	0,04588	0	GSPATT00028112001	0,016	0,92503	0
GSPATT00009009001	-0,53	0,00588	0	GSPATT00028130001	0,382	0,0628	0
GSPATT00009013001	1,033	0,00013	1	GSPATT00028137001	-0,328	0,07792	0
GSPATT00009076001	0,364	0,0251	0	GSPATT00028156001	0,053	0,70232	0
GSPATT00009086001	0,586	0,00945	0	GSPATT00028162001	0,316	0,14219	0
GSPATT00009091001	-2,067	0,00001	-1	GSPATT00028174001	-0,017	0,90512	0
GSPATT00009102001	0,179	0,23283	0	GSPATT00028181001	0,307	0,0498	0
GSPATT00009110001	0,013	0,95996	0	GSPATT00028199001	1,181	0,00015	1
GSPATT00009123001	-0,164	0,60057	0	GSPATT00028210001	-0,239	0,19183	0
GSPATT00009138001	1,521	0,00028	1	GSPATT00028223001	0,071	0,6272	0
GSPATT00009146001	-0,481	0,05125	0	GSPATT00028224001	-0,142	0,45435	0

GSPATT00009149001	0,025	0,87197	0	GSPATT00028226001	-0,224	0,18937	0
GSPATT00009155001	-0,238	0,12312	0	GSPATT00028254001	0,074	0,68566	0
GSPATT00009172001	-1,485	0,00015	-1	GSPATT00028260001	0,201	0,33982	0
GSPATT00009173001	0,244	0,23213	0	GSPATT00028261001	0,648	0,00753	0
GSPATT00009174001	0,724	0,00457	0	GSPATT00028264001	-0,912	0,00051	0
GSPATT00009176001	0,333	0,1816	0	GSPATT00028265001	-0,664	0,00186	0
GSPATT00009191001	0,221	0,13878	0	GSPATT00028266001	-0,643	0,01703	0
GSPATT00009207001	1,429	0,00002	1	GSPATT00028267001	1,612	0,00001	1
GSPATT00009211001	1,961	0	1	GSPATT00028269001	0,88	0,00108	0
GSPATT00009267001	-1,116	0,00261	-1	GSPATT00028305001	0,264	0,18486	0
GSPATT00009271001	-0,931	0,00059	0	GSPATT00028307001	-0,082	0,66338	0
GSPATT00009291001	0,067	0,70438	0	GSPATT00028312001	0,636	0,00684	0
GSPATT00009344001	-0,02	0,89901	0	GSPATT00028347001	0,779	0,00108	0
GSPATT00009346001	0,505	0,00751	0	GSPATT00028368001	0,41	0,03475	0
GSPATT00009355001	-0,135	0,39547	0	GSPATT00028374001	0,68	0,00629	0
GSPATT00009389001	0,729	0,00072	0	GSPATT00028387001	-0,687	0,00975	0
GSPATT00009399001	0,067	0,85641	0	GSPATT00028405001	0,115	0,6059	0
GSPATT00009411001	0,367	0,14277	0	GSPATT00028413001	0,051	0,72041	0
GSPATT00009414001	1,265	0,00079	1	GSPATT00028458001	-1,195	0,00003	-1
GSPATT00009428001	0,129	0,62268	0	GSPATT00028463001	0,348	0,16251	0
GSPATT00009429001	-0,821	0,00035	0	GSPATT00028502001	0,619	0,01367	0
GSPATT00009449001	0,122	0,41636	0	GSPATT00028515001	0,356	0,07284	0
GSPATT00009461001	-1,129	0,00293	-1	GSPATT00028521001	-0,347	0,06932	0
GSPATT00009466001	0,566	0,00692	0	GSPATT00028522001	0,518	0,02311	0
GSPATT00009502001	-0,497	0,00886	0	GSPATT00028523001	-0,194	0,30675	0
GSPATT00009512001	0,817	0,00579	0	GSPATT00028553001	-1,461	0,00001	-1
GSPATT00009524001	0,752	0,00072	0	GSPATT00028591001	-0,079	0,66532	0
GSPATT00009529001	-0,262	0,0797	0	GSPATT00028640001	-0,009	0,94942	0
GSPATT00009531001	0,045	0,73938	0	GSPATT00028664001	1,165	0,00037	1
GSPATT00009539001	-0,64	0,00235	0	GSPATT00028690001	0,455	0,03584	0
GSPATT00009552001	-0,641	0,01566	0	GSPATT00028736001	0,809	0,00541	0
GSPATT00009559001	0,264	0,18251	0	GSPATT00028737001	1,259	0,00014	1
GSPATT00009580001	0,2	0,21252	0	GSPATT00028739001	0,281	0,07289	0
GSPATT00009588001	0,994	0,04353	0	GSPATT00028760001	-0,016	0,95354	0
GSPATT00009601001	-0,457	0,11601	0	GSPATT00028761001	-0,584	0,00278	0
GSPATT00009602001	0,93	0,0008	0	GSPATT00028762001	0,71	0,00514	0
GSPATT00009607001	0,735	0,01843	0	GSPATT00028795001	-0,564	0,00507	0
GSPATT00009626001	0,037	0,77284	0	GSPATT00028814001	0,718	0,00439	0
GSPATT00009643001	0,405	0,01362	0	GSPATT00028854001	0,383	0,0558	0
GSPATT00009645001	0,973	0,00151	0	GSPATT00028862001	0,155	0,44362	0
GSPATT00009646001	0,589	0,00229	0	GSPATT00028879001	-0,101	0,52631	0
GSPATT00009693001	0,347	0,0372	0	GSPATT00028902001	0,115	0,49398	0
GSPATT00009703001	-2,787	0	-1	GSPATT00028903001	0,487	0,0098	0
GSPATT00009718001	-0,819	0,00093	0	GSPATT00028907001	0,31	0,07543	0
GSPATT00009748001	0,253	0,1151	0	GSPATT00028913001	0,627	0,00447	0
GSPATT00009760001	-2,631	0	-1	GSPATT00028920001	0,862	0,00104	0
GSPATT00009772001	-0,026	0,84037	0	GSPATT00028924001	0,504	0,00498	0
GSPATT00009782001	-0,119	0,55451	0	GSPATT00028949001	0,858	0,01717	0
GSPATT00009828001	0,419	0,03475	0	GSPATT00028981001	0,411	0,07642	0
GSPATT00009848001	-0,228	0,27077	0	GSPATT00029004001	-0,194	0,28836	0
GSPATT00009873001	1,405	0,00002	1	GSPATT00029007001	0,785	0,00492	0
GSPATT00009883001	0,58	0,00174	0	GSPATT00029023001	0,516	0,03057	0
GSPATT00009888001	0,679	0,00449	0	GSPATT00029041001	0,57	0,00776	0
GSPATT00009892001	-0,164	0,50097	0	GSPATT00029053001	-0,652	0,01479	0
GSPATT00009932001	-0,127	0,37952	0	GSPATT00029067001	-0,083	0,53645	0
GSPATT00009937001	0,505	0,12425	0	GSPATT00029101001	-0,869	0,00393	0
GSPATT00009951001	-0,279	0,21687	0	GSPATT00029164001	0,564	0,12006	0
GSPATT00009964001	0,607	0,03286	0	GSPATT00029173001	0,138	0,34159	0
GSPATT00009969001	0,018	0,89309	0	GSPATT00029175001	-0,791	0,00029	0
GSPATT00009980001	0,361	0,01933	0	GSPATT00029187001	0,682	0,01155	0
GSPATT00009997001	0,66	0,0026	0	GSPATT00029196001	2,188	0,00004	1
GSPATT00010022001	-0,69	0,00171	0	GSPATT00029198001	0,416	0,04007	0
GSPATT00010023001	0,56	0,00691	0	GSPATT00029217001	0,222	0,14787	0
GSPATT00010038001	0,125	0,38308	0	GSPATT00029249001	-0,351	0,02712	0
GSPATT00010040001	-0,369	0,07302	0	GSPATT00029273001	-2,046	0,00011	-1
GSPATT00010043001	0,211	0,26138	0	GSPATT00029295001	1,454	0,00404	1
GSPATT00010044001	0,443	0,07115	0	GSPATT00029341001	0,262	0,14262	0

GSPATT00010047001	0,376	0,04978	0	GSPATT00029366001	0,262	0,1057	0
GSPATT00010069001	0,096	0,49349	0	GSPATT00029406001	0,843	0,00341	0
GSPATT00010072001	0,597	0,04414	0	GSPATT00029408001	0,85	0,0034	0
GSPATT00010088001	0,474	0,01739	0	GSPATT00029420001	1,305	0,00003	1
GSPATT00010089001	-0,02	0,89788	0	GSPATT00029433001	0,663	0,01729	0
GSPATT00010120001	0,439	0,05835	0	GSPATT00029443001	-0,601	0,00289	0
GSPATT00010165001	1,057	0,05472	0	GSPATT00029444001	1,26	0,00076	1
GSPATT00010186001	-0,625	0,00879	0	GSPATT00029470001	0,093	0,49861	0
GSPATT00010187001	0,077	0,70848	0	GSPATT00029472001	0,184	0,20615	0
GSPATT00010189001	0,274	0,09078	0	GSPATT00029484001	0,665	0,00137	0
GSPATT00010192001	-0,341	0,04434	0	GSPATT00029530001	0,081	0,70204	0
GSPATT00010201001	-1,112	0,00403	-1	GSPATT00029552001	0,56	0,01662	0
GSPATT00010217001	0,055	0,68694	0	GSPATT00029556001	-0,933	0,00515	0
GSPATT00010238001	0,721	0,00063	0	GSPATT00029558001	1,707	0,00001	1
GSPATT00010240001	1,641	0,00002	1	GSPATT00029574001	-1,108	0,00039	-1
GSPATT00010241001	-0,059	0,67929	0	GSPATT00029583001	0,198	0,35094	0
GSPATT00010256001	0,667	0,16041	0	GSPATT00029601001	1,502	0,00004	1
GSPATT00010264001	1,126	0,00022	1	GSPATT00029614001	-0,616	0,00563	0
GSPATT00010267001	0,363	0,04122	0	GSPATT00029618001	0,584	0,00422	0
GSPATT00010305001	0,583	0,02695	0	GSPATT00029627001	-0,199	0,15139	0
GSPATT00010342001	-0,027	0,88113	0	GSPATT00029631001	0,75	0,00323	0
GSPATT00010360001	0,009	0,9481	0	GSPATT00029713001	0,817	0,00169	0
GSPATT00010361001	1,344	0,00002	1	GSPATT00029723001	0,372	0,11982	0
GSPATT00010434001	0,716	0,00843	0	GSPATT00029731001	0,613	0,0095	0
GSPATT00010455001	2,068	0,00001	1	GSPATT00029750001	0,907	0,00106	0
GSPATT00010478001	0,755	0,0015	0	GSPATT00029761001	0,264	0,06412	0
GSPATT00010489001	0,856	0,01707	0	GSPATT00029771001	-0,737	0,0067	0
GSPATT00010520001	1,675	0,00001	1	GSPATT00029778001	-0,608	0,00307	0
GSPATT00010535001	0,291	0,15153	0	GSPATT00029822001	0,665	0,00641	0
GSPATT00010550001	-0,166	0,3917	0	GSPATT00029823001	-0,53	0,02834	0
GSPATT00010557001	0,305	0,0402	0	GSPATT00029824001	0,784	0,05706	0
GSPATT00010582001	-0,301	0,06581	0	GSPATT00029844001	0,11	0,52769	0
GSPATT00010591001	-0,327	0,06596	0	GSPATT00029848001	0,411	0,08951	0
GSPATT00010597001	0,042	0,75702	0	GSPATT00029901001	0,748	0,00121	0
GSPATT00010598001	0,186	0,32536	0	GSPATT00029929001	1,561	0,00012	1
GSPATT00010599001	-0,541	0,00596	0	GSPATT00029968001	0,19	0,2579	0
GSPATT00010606001	-0,148	0,37763	0	GSPATT00029971001	-0,01	0,95787	0
GSPATT00010615001	0,827	0,00035	0	GSPATT00030006001	0,333	0,17202	0
GSPATT00010622001	0,288	0,09844	0	GSPATT00030011001	0,244	0,31877	0
GSPATT00010631001	-0,002	0,99061	0	GSPATT00030052001	-0,257	0,11428	0
GSPATT00010634001	-0,099	0,64063	0	GSPATT00030056001	0,096	0,60981	0
GSPATT00010700001	1,693	0	1	GSPATT00030064001	1,558	0,00015	1
GSPATT00010735001	0,406	0,06251	0	GSPATT00030135001	0,579	0,06809	0
GSPATT00010751001	0,385	0,03146	0	GSPATT00030142001	0,973	0,0012	0
GSPATT00010794001	0,388	0,14585	0	GSPATT00030146001	-0,644	0,00333	0
GSPATT00010803001	0,375	0,05126	0	GSPATT00030175001	-0,442	0,01694	0
GSPATT00010807001	0,842	0,00505	0	GSPATT00030183001	0,09	0,72161	0
GSPATT00010846001	0,562	0,04885	0	GSPATT00030220001	-0,307	0,04731	0
GSPATT00010852001	0,595	0,00207	0	GSPATT00030230001	0,126	0,43629	0
GSPATT00010926001	-0,295	0,08518	0	GSPATT00030249001	1,907	0	1
GSPATT00010927001	0,963	0,00093	0	GSPATT00030250001	0,03	0,86055	0
GSPATT00010930001	0,326	0,08283	0	GSPATT00030251001	0,536	0,02526	0
GSPATT00010931001	0,755	0,00409	0	GSPATT00030268001	0,167	0,24397	0
GSPATT00010951001	-0,4	0,08547	0	GSPATT00030295001	0,775	0,00158	0
GSPATT00010956001	-0,78	0,00146	0	GSPATT00030300001	0,307	0,11242	0
GSPATT00010982001	1,537	0,00037	1	GSPATT00030312001	0,159	0,34993	0
GSPATT00010994001	-2,479	0	-1	GSPATT00030338001	0,37	0,02763	0
GSPATT00011004001	0,073	0,58776	0	GSPATT00030339001	0,929	0,00014	0
GSPATT00011094001	-0,248	0,11654	0	GSPATT00030340001	-0,629	0,00193	0
GSPATT00011096001	0,27	0,14138	0	GSPATT00030347001	0,305	0,05631	0
GSPATT00011110001	0,439	0,02922	0	GSPATT00030360001	1,11	0,00034	1
GSPATT00011112001	0,611	0,00704	0	GSPATT00030377001	0,608	0,03137	0
GSPATT00011130001	0,933	0,00615	0	GSPATT00030395001	0,632	0,00363	0
GSPATT00011132001	-1,165	0,00035	-1	GSPATT00030433001	0,649	0,01931	0
GSPATT00011155001	0,816	0,00288	0	GSPATT00030441001	-1,166	0,00076	-1
GSPATT00011167001	-0,396	0,02351	0	GSPATT00030461001	0,787	0,01769	0
GSPATT00011173001	-3,857	0	-1	GSPATT00030462001	2,069	0	1

GSPATT00011179001	0,334	0,11641	0	GSPATT00030463001	0,18	0,23178	0
GSPATT00011220001	0,315	0,15075	0	GSPATT00030486001	-1,516	0,00003	-1
GSPATT00011221001	1,038	0,00031	1	GSPATT00030525001	0,296	0,14492	0
GSPATT00011227001	0,852	0,00196	0	GSPATT00030559001	1,24	0,00015	1
GSPATT00011241001	0,626	0,02469	0	GSPATT00030602001	0,266	0,22578	0
GSPATT00011251001	0,282	0,09853	0	GSPATT00030614001	-0,182	0,32497	0
GSPATT00011290001	1,791	0	1	GSPATT00030636001	0,66	0,0019	0
GSPATT00011305001	-0,492	0,0392	0	GSPATT00030653001	0,411	0,01804	0
GSPATT00011340001	0,056	0,74423	0	GSPATT00030654001	-0,73	0,00085	0
GSPATT00011369001	1,426	0,00002	1	GSPATT00030663001	0,245	0,35912	0
GSPATT00011371001	-0,116	0,58125	0	GSPATT00030666001	0,411	0,06139	0
GSPATT00011372001	-0,323	0,11988	0	GSPATT00030669001	0,057	0,6915	0
GSPATT00011413001	0,314	0,1297	0	GSPATT00030671001	-0,295	0,30814	0
GSPATT00011422001	-1,076	0,00614	-1	GSPATT00030682001	-0,162	0,29594	0
GSPATT00011430001	0,323	0,07271	0	GSPATT00030687001	0,812	0,01438	0
GSPATT00011436001	0,608	0,01574	0	GSPATT00030688001	-0,02	0,90163	0
GSPATT00011440001	0,123	0,57717	0	GSPATT00030698001	0,071	0,70437	0
GSPATT00011454001	0,507	0,07282	0	GSPATT00030723001	-0,18	0,43523	0
GSPATT00011455001	-0,483	0,15659	0	GSPATT00030733001	-1,617	0,00013	-1
GSPATT00011486001	-0,104	0,60315	0	GSPATT00030745001	-0,1	0,48372	0
GSPATT00011499001	-0,148	0,29042	0	GSPATT00030749001	-0,359	0,06814	0
GSPATT00011515001	0,879	0,00666	0	GSPATT00030782001	0,235	0,17113	0
GSPATT00011546001	-0,929	0,00313	0	GSPATT00030803001	1,123	0,0001	1
GSPATT00011547001	0,964	0,00026	0	GSPATT00030806001	0,727	0,0015	0
GSPATT00011564001	0,676	0,00108	0	GSPATT00030818001	-0,245	0,23455	0
GSPATT00011566001	0,262	0,07738	0	GSPATT00030826001	-2,147	0,00001	-1
GSPATT00011580001	-0,02	0,90743	0	GSPATT00030848001	0,031	0,86428	0
GSPATT00011584001	0,283	0,12359	0	GSPATT00030851001	0,263	0,1287	0
GSPATT00011622001	0,355	0,17874	0	GSPATT00030853001	0,549	0,03624	0
GSPATT00011666001	-0,488	0,00499	0	GSPATT00030855001	1,1	0,00013	1
GSPATT00011685001	-0,399	0,05343	0	GSPATT00030860001	1,314	0,00014	1
GSPATT00011700001	-0,131	0,51052	0	GSPATT00030871001	-0,434	0,01131	0
GSPATT00011714001	0,124	0,49457	0	GSPATT00030877001	-0,793	0,0011	0
GSPATT00011716001	0,632	0,00185	0	GSPATT00030894001	0,443	0,01543	0
GSPATT00011729001	-0,066	0,66441	0	GSPATT00030895001	0,613	0,00519	0
GSPATT00011731001	0,353	0,05227	0	GSPATT00030907001	0,89	0,00402	0
GSPATT00011735001	1,538	0,00053	1	GSPATT00030912001	1,631	0,00007	1
GSPATT00011739001	-0,466	0,04961	0	GSPATT00030921001	0,509	0,01961	0
GSPATT00011742001	1,365	0,00005	1	GSPATT00030945001	0,658	0,01733	0
GSPATT00011764001	0,296	0,13873	0	GSPATT00030952001	-0,016	0,91391	0
GSPATT00011816001	-0,319	0,13633	0	GSPATT00030956001	-0,545	0,00308	0
GSPATT00011825001	-0,485	0,08169	0	GSPATT00030971001	0,707	0,00637	0
GSPATT00011826001	1,565	0,00133	1	GSPATT00030979001	0,011	0,94131	0
GSPATT00011840001	-0,798	0,00327	0	GSPATT00030994001	0,373	0,06118	0
GSPATT00011854001	0,553	0,02078	0	GSPATT00031006001	1,218	0,00004	1
GSPATT00011856001	0,65	0,07665	0	GSPATT00031028001	-0,522	0,00869	0
GSPATT00011864001	-0,091	0,49677	0	GSPATT00031029001	-0,147	0,30646	0
GSPATT00011887001	0,175	0,46958	0	GSPATT00031066001	-3,25	0	-1
GSPATT00011897001	0,61	0,00299	0	GSPATT00031082001	0,807	0,00177	0
GSPATT00011903001	0,087	0,58179	0	GSPATT00031097001	-0,034	0,81174	0
GSPATT00011917001	0,417	0,15463	0	GSPATT00031104001	-1,662	0,00021	-1
GSPATT00011929001	-1,564	0,00004	-1	GSPATT00031107001	0,915	0,00188	0
GSPATT00011952001	0,118	0,38041	0	GSPATT00031110001	1,016	0,0098	1
GSPATT00011968001	0,326	0,1663	0	GSPATT00031155001	-0,356	0,05023	0
GSPATT00011981001	-0,032	0,83233	0	GSPATT00031161001	2,492	0	1
GSPATT00011982001	0,265	0,10595	0	GSPATT00031166001	0,279	0,06965	0
GSPATT00012043001	0,391	0,11161	0	GSPATT00031167001	0,272	0,08891	0
GSPATT00012044001	-0,04	0,80131	0	GSPATT00031168001	0,836	0,01523	0
GSPATT00012047001	-0,114	0,39986	0	GSPATT00031178001	0,551	0,0218	0
GSPATT00012141001	0,915	0,00115	0	GSPATT00031182001	-0,535	0,01622	0
GSPATT00012162001	0,3	0,06106	0	GSPATT00031235001	0,643	0,00762	0
GSPATT00012163001	-0,072	0,57986	0	GSPATT00031285001	-0,236	0,21081	0
GSPATT00012176001	0,545	0,02424	0	GSPATT00031287001	0,155	0,35654	0
GSPATT00012210001	-0,372	0,02535	0	GSPATT00031336001	0,795	0,00047	0
GSPATT00012216001	1,175	0,00014	1	GSPATT00031426001	-1,033	0,00006	-1
GSPATT00012223001	0,404	0,08406	0	GSPATT00031441001	-0,048	0,82385	0
GSPATT00012241001	0,159	0,5354	0	GSPATT00031487001	0,99	0,002	0

GSPATT00012255001	0,104	0,64586	0	GSPATT00031493001	0,611	0,02344	0
GSPATT00012262001	-1,15	0,00006	-1	GSPATT00031509001	-0,798	0,00098	0
GSPATT00012269001	-0,185	0,39154	0	GSPATT00031532001	1,665	0,00021	1
GSPATT00012278001	1,263	0,00039	1	GSPATT00031569001	-0,125	0,54879	0
GSPATT00012282001	0,837	0,01461	0	GSPATT00031612001	2,01	0,00001	1
GSPATT00012286001	-0,79	0,00041	0	GSPATT00031618001	0,323	0,2332	0
GSPATT00012322001	1,362	0,00005	1	GSPATT00031623001	-0,002	0,9946	0
GSPATT00012339001	1,278	0,00002	1	GSPATT00031630001	1,142	0,00103	1
GSPATT00012348001	-1,134	0,00014	-1	GSPATT00031642001	-1,737	0,00014	-1
GSPATT00012351001	0,299	0,06088	0	GSPATT00031645001	0,862	0,00026	0
GSPATT00012371001	-0,352	0,29514	0	GSPATT00031653001	-0,225	0,14582	0
GSPATT00012372001	0,15	0,484	0	GSPATT00031661001	0,548	0,02007	0
GSPATT00012374001	0,203	0,24495	0	GSPATT00031665001	-0,001	0,99648	0
GSPATT00012376001	0,959	0,00045	0	GSPATT00031698001	-0,689	0,03351	0
GSPATT00012379001	0,369	0,07668	0	GSPATT00031701001	-0,914	0,00444	0
GSPATT00012395001	0,809	0,0075	0	GSPATT00031705001	1,81	0,00002	1
GSPATT00012396001	0,319	0,11515	0	GSPATT00031706001	-0,775	0,0011	0
GSPATT00012400001	0,852	0,00032	0	GSPATT00031738001	-1,034	0,00008	-1
GSPATT00012413001	0,837	0,00048	0	GSPATT00031739001	0,382	0,19043	0
GSPATT00012442001	0,124	0,39221	0	GSPATT00031740001	0,355	0,05225	0
GSPATT00012445001	0,429	0,03783	0	GSPATT00031754001	-0,461	0,03297	0
GSPATT00012460001	0,238	0,14276	0	GSPATT00031756001	-0,463	0,03455	0
GSPATT00012467001	0,278	0,19931	0	GSPATT00031757001	0,716	0,03439	0
GSPATT00012470001	-0,034	0,86348	0	GSPATT00031762001	-1,768	0,00003	-1
GSPATT00012488001	-0,04	0,75807	0	GSPATT00031768001	-0,037	0,82459	0
GSPATT00012520001	-2,03	0	-1	GSPATT00031781001	-0,507	0,02156	0
GSPATT00012525001	0,13	0,44838	0	GSPATT00031782001	-0,24	0,42749	0
GSPATT00012543001	0,86	0,00059	0	GSPATT00031802001	0,907	0,00053	0
GSPATT00012570001	0,319	0,06017	0	GSPATT00031822001	0,463	0,05116	0
GSPATT00012593001	-0,057	0,67906	0	GSPATT00031828001	-0,487	0,01743	0
GSPATT00012610001	0,358	0,06157	0	GSPATT00031846001	-0,183	0,21789	0
GSPATT00012628001	0,326	0,03065	0	GSPATT00031865001	0,272	0,16899	0
GSPATT00012631001	0,442	0,02953	0	GSPATT00031924001	0,545	0,00883	0
GSPATT00012633001	0,282	0,10356	0	GSPATT00031948001	-0,37	0,18789	0
GSPATT00012634001	0,634	0,00459	0	GSPATT00031955001	-0,129	0,4888	0
GSPATT00012649001	-0,517	0,01272	0	GSPATT00031957001	0,03	0,87201	0
GSPATT00012656001	0,231	0,16634	0	GSPATT00032021001	0,096	0,48485	0
GSPATT00012666001	0,427	0,02248	0	GSPATT00032022001	-0,54	0,01533	0
GSPATT00012678001	-0,029	0,84403	0	GSPATT00032029001	0,283	0,22374	0
GSPATT00012684001	-0,036	0,83675	0	GSPATT00032030001	1,339	0,00008	1
GSPATT00012692001	0,375	0,08064	0	GSPATT00032037001	0,772	0,00865	0
GSPATT00012702001	0,193	0,34721	0	GSPATT00032092001	0,508	0,09947	0
GSPATT00012722001	0,574	0,01609	0	GSPATT00032094001	-2,851	0	-1
GSPATT00012732001	0,363	0,12773	0	GSPATT00032095001	0,112	0,41059	0
GSPATT00012735001	0,149	0,25751	0	GSPATT00032099001	0,878	0,00102	0
GSPATT00012742001	1,227	0,00011	1	GSPATT00032100001	0,509	0,00574	0
GSPATT00012746001	0,143	0,51196	0	GSPATT00032104001	-0,024	0,9428	0
GSPATT00012753001	0,157	0,26935	0	GSPATT00032110001	-1,089	0,00427	-1
GSPATT00012754001	-0,119	0,5919	0	GSPATT00032111001	-1,233	0,00541	-1
GSPATT00012755001	1,516	0,00005	1	GSPATT00032114001	0,44	0,01977	0
GSPATT00012756001	0,835	0,00358	0	GSPATT00032136001	-0,671	0,00209	0
GSPATT00012822001	0,123	0,4374	0	GSPATT00032166001	0,482	0,04639	0
GSPATT00012835001	0,596	0,00563	0	GSPATT00032169001	0,497	0,00936	0
GSPATT00012838001	0,001	0,99699	0	GSPATT00032224001	0,067	0,66182	0
GSPATT00012852001	1,275	0,00003	1	GSPATT00032226001	0,466	0,04696	0
GSPATT00012853001	0,823	0,00197	0	GSPATT00032238001	0,364	0,06778	0
GSPATT00012859001	-0,198	0,15153	0	GSPATT00032247001	-0,428	0,012	0
GSPATT00012868001	0,163	0,31281	0	GSPATT00032288001	0,859	0,00547	0
GSPATT00012875001	0,348	0,03129	0	GSPATT00032289001	-0,097	0,49171	0
GSPATT00012913001	0,495	0,01652	0	GSPATT00032301001	-0,947	0,00029	0
GSPATT00012914001	0,157	0,26322	0	GSPATT00032341001	0,829	0,00058	0
GSPATT00012916001	-2,396	0	-1	GSPATT00032372001	-0,241	0,09642	0
GSPATT00012931001	0,812	0,00089	0	GSPATT00032392001	0,313	0,05107	0
GSPATT00012973001	-0,403	0,04733	0	GSPATT00032421001	-1,221	0,00022	-1
GSPATT00012989001	0,026	0,8519	0	GSPATT00032429001	0,493	0,03216	0
GSPATT00012998001	-0,105	0,43684	0	GSPATT00032432001	-0,292	0,20952	0
GSPATT00013005001	-0,158	0,4174	0	GSPATT00032442001	0,198	0,2398	0

GSPATT00013045001	0,257	0,10688	0	GSPATT00032444001	-0,967	0,00044	0
GSPATT00013048001	0,033	0,83799	0	GSPATT00032458001	0,703	0,02409	0
GSPATT00013062001	-0,119	0,40756	0	GSPATT00032459001	0,275	0,11268	0
GSPATT00013085001	1,595	0,00002	1	GSPATT00032460001	-0,526	0,00918	0
GSPATT00013094001	0,81	0,00674	0	GSPATT00032522001	-0,127	0,45119	0
GSPATT00013102001	0,769	0,00426	0	GSPATT00032523001	0,812	0,00089	0
GSPATT00013110001	-1,076	0,00259	-1	GSPATT00032532001	0,103	0,45294	0
GSPATT00013157001	0,235	0,17548	0	GSPATT00032533001	-0,17	0,53723	0
GSPATT00013159001	0,37	0,09003	0	GSPATT00032559001	0,124	0,37615	0
GSPATT00013164001	-0,51	0,05354	0	GSPATT00032564001	1,204	0,00066	1
GSPATT00013165001	1,021	0,00106	1	GSPATT00032574001	1,413	0,00228	1
GSPATT00013173001	-0,295	0,08555	0	GSPATT00032597001	0,884	0,00108	0
GSPATT00013174001	0,011	0,93101	0	GSPATT00032618001	0,457	0,06613	0
GSPATT00013205001	0,52	0,00824	0	GSPATT00032619001	0,741	0,02826	0
GSPATT00013243001	-0,027	0,84517	0	GSPATT00032623001	-0,162	0,37167	0
GSPATT00013253001	1,885	0,00001	1	GSPATT00032649001	0,305	0,06511	0
GSPATT00013260001	0,645	0,00484	0	GSPATT00032667001	1,812	0,00041	1
GSPATT00013263001	0,563	0,01505	0	GSPATT00032670001	0,786	0,00243	0
GSPATT00013271001	0,175	0,21491	0	GSPATT00032697001	0,477	0,0138	0
GSPATT00013272001	1,673	0,00001	1	GSPATT00032706001	0,443	0,01419	0
GSPATT00013281001	0,128	0,50204	0	GSPATT00032739001	0,096	0,63648	0
GSPATT00013308001	0,015	0,90603	0	GSPATT00032755001	-0,448	0,01909	0
GSPATT00013315001	-0,188	0,20496	0	GSPATT00032763001	-0,489	0,02531	0
GSPATT00013327001	-0,009	0,94794	0	GSPATT00032783001	-0,567	0,00797	0
GSPATT00013343001	0,267	0,15492	0	GSPATT00032791001	0,581	0,0063	0
GSPATT00013356001	-0,722	0,00162	0	GSPATT00032810001	0,795	0,00874	0
GSPATT00013360001	-0,83	0,00139	0	GSPATT00032894001	0,722	0,00361	0
GSPATT00013364001	-0,11	0,50767	0	GSPATT00032910001	1,39	0,00003	1
GSPATT00013383001	-1,314	0,0001	-1	GSPATT00032920001	0,74	0,00144	0
GSPATT00013453001	0,604	0,02056	0	GSPATT00032929001	0,137	0,64968	0
GSPATT00013470001	0,177	0,28718	0	GSPATT00032930001	1,256	0,00033	1
GSPATT00013490001	0,322	0,07055	0	GSPATT00032934001	0,256	0,2624	0
GSPATT00013494001	0,052	0,75665	0	GSPATT00032999001	0,68	0,00704	0
GSPATT00013502001	-0,243	0,19365	0	GSPATT00033009001	-0,785	0,00143	0
GSPATT00013552001	0,741	0,00042	0	GSPATT00033016001	0,224	0,20102	0
GSPATT00013561001	0,271	0,16205	0	GSPATT00033027001	0,378	0,03882	0
GSPATT00013578001	1,991	0,00001	1	GSPATT00033067001	0,968	0,00046	0
GSPATT00013652001	0,191	0,18598	0	GSPATT00033072001	0,525	0,02731	0
GSPATT00013656001	0,171	0,2115	0	GSPATT00033076001	0,219	0,41915	0
GSPATT00013680001	-0,238	0,41631	0	GSPATT00033095001	-1,102	0,00064	-1
GSPATT00013693001	0,493	0,01038	0	GSPATT00033106001	0,765	0,00138	0
GSPATT00013695001	0,474	0,01357	0	GSPATT00033119001	-0,51	0,03205	0
GSPATT00013702001	-0,192	0,32187	0	GSPATT00033139001	0,432	0,01158	0
GSPATT00013754001	-3,054	0	-1	GSPATT00033154001	1,026	0,00696	1
GSPATT00013770001	-0,187	0,4332	0	GSPATT00033161001	-0,302	0,08205	0
GSPATT00013783001	1,026	0,01651	1	GSPATT00033165001	-0,046	0,8569	0
GSPATT00013790001	0,074	0,5863	0	GSPATT00033167001	-0,34	0,05638	0
GSPATT00013801001	0,361	0,0755	0	GSPATT00033198001	0,629	0,09101	0
GSPATT00013825001	-0,769	0,00205	0	GSPATT00033205001	0,673	0,00702	0
GSPATT00013827001	0,481	0,00732	0	GSPATT00033223001	-0,423	0,03978	0
GSPATT00013828001	0,419	0,03295	0	GSPATT00033237001	-0,283	0,16249	0
GSPATT00013847001	0,139	0,40055	0	GSPATT00033250001	-0,453	0,0188	0
GSPATT00013852001	-0,368	0,03397	0	GSPATT00033258001	0,335	0,03868	0
GSPATT00013855001	0,438	0,04631	0	GSPATT00033260001	-0,16	0,55088	0
GSPATT00013876001	1,319	0,00047	1	GSPATT00033274001	0,713	0,00057	0
GSPATT00013891001	0,176	0,26584	0	GSPATT00033306001	-0,043	0,85073	0
GSPATT00013892001	-1,136	0,00035	-1	GSPATT00033333001	0,576	0,00241	0
GSPATT00013898001	0,162	0,43243	0	GSPATT00033334001	0,4	0,0313	0
GSPATT00013900001	-0,003	0,98838	0	GSPATT00033358001	-0,167	0,26712	0
GSPATT00013901001	0,247	0,35648	0	GSPATT00033399001	-0,126	0,62052	0
GSPATT00013913001	1,633	0,00001	1	GSPATT00033466001	-1,107	0,00106	-1
GSPATT00013928001	0,8	0,00105	0	GSPATT00033469001	0,884	0,00069	0
GSPATT00013961001	2,283	0,00001	1	GSPATT00033477001	0,439	0,04845	0
GSPATT00013977001	0,458	0,01806	0	GSPATT00033494001	0,481	0,13408	0
GSPATT00013994001	1,578	0,00006	1	GSPATT00033495001	1,143	0,00006	1
GSPATT00014001001	0,554	0,00382	0	GSPATT00033505001	0,875	0,00023	0
GSPATT00014008001	0,723	0,00107	0	GSPATT00033512001	-3,055	0	-1

GSPATT00014044001	0,335	0,03981	0	GSPATT00033523001	-1,294	0,00005	-1
GSPATT00014047001	0,165	0,26272	0	GSPATT00033534001	0,523	0,02677	0
GSPATT00014072001	0,024	0,90147	0	GSPATT00033537001	0,391	0,03807	0
GSPATT00014088001	0,517	0,03193	0	GSPATT00033539001	0,246	0,40993	0
GSPATT00014093001	0,192	0,19217	0	GSPATT00033568001	-0,204	0,16501	0
GSPATT00014104001	0,566	0,03625	0	GSPATT00033573001	0,114	0,43633	0
GSPATT00014124001	-1,671	0,00007	-1	GSPATT00033586001	0,277	0,16433	0
GSPATT00014132001	2,015	0	1	GSPATT00033606001	-0,328	0,25723	0
GSPATT00014139001	0,906	0,02173	0	GSPATT00033617001	0,273	0,25396	0
GSPATT00014152001	0,095	0,60893	0	GSPATT00033636001	0,336	0,04963	0
GSPATT00014158001	1,1	0,00099	1	GSPATT00033646001	-0,077	0,64536	0
GSPATT00014196001	-0,265	0,26029	0	GSPATT00033676001	0,609	0,03214	0
GSPATT00014197001	0,509	0,02553	0	GSPATT00033682001	-0,181	0,30005	0
GSPATT00014202001	0,571	0,00343	0	GSPATT00033727001	-0,264	0,20849	0
GSPATT00014213001	-0,445	0,00944	0	GSPATT00033743001	0,187	0,34652	0
GSPATT00014216001	0,08	0,66083	0	GSPATT00033764001	1,303	0,00004	1
GSPATT00014218001	0,813	0,0031	0	GSPATT00033786001	0,824	0,01346	0
GSPATT00014219001	0,639	0,00371	0	GSPATT00033798001	0,882	0,00143	0
GSPATT00014235001	0,358	0,02401	0	GSPATT00033801001	0,274	0,11977	0
GSPATT00014239001	0,559	0,04551	0	GSPATT00033810001	-0,178	0,21774	0
GSPATT00014253001	-0,142	0,35399	0	GSPATT00033826001	0,54	0,01676	0
GSPATT00014299001	0,536	0,03039	0	GSPATT00033832001	1,004	0,0005	1
GSPATT00014310001	0,41	0,03511	0	GSPATT00033839001	0,232	0,20924	0
GSPATT00014335001	-0,025	0,84538	0	GSPATT00033861001	0,201	0,23013	0
GSPATT00014350001	-0,099	0,44714	0	GSPATT00033863001	0,989	0,00013	0
GSPATT00014391001	0,443	0,007	0	GSPATT00033871001	0,751	0,00036	0
GSPATT00014411001	-0,004	0,97822	0	GSPATT00033879001	0,892	0,00128	0
GSPATT00014414001	0,567	0,00623	0	GSPATT00033881001	0,29	0,19304	0
GSPATT00014433001	-0,31	0,10884	0	GSPATT00033904001	0,322	0,04932	0
GSPATT00014438001	0,123	0,39312	0	GSPATT00033912001	0,771	0,0063	0
GSPATT00014461001	0,95	0,00019	0	GSPATT00033917001	0,213	0,23333	0
GSPATT00014474001	-1,949	0	-1	GSPATT00033938001	1,074	0,00021	1
GSPATT00014478001	0,152	0,53003	0	GSPATT00033979001	-0,385	0,04696	0
GSPATT00014523001	1,029	0,0076	1	GSPATT00033980001	0,087	0,60132	0
GSPATT00014525001	1,28	0,0002	1	GSPATT00034005001	1,03	0,00044	1
GSPATT00014529001	1,182	0,00018	1	GSPATT00034018001	-1,92	0	-1
GSPATT00014568001	-0,37	0,05496	0	GSPATT00034024001	1,786	0,00003	1
GSPATT00014587001	-0,019	0,88031	0	GSPATT00034063001	-0,183	0,27172	0
GSPATT00014606001	0,487	0,04116	0	GSPATT00034071001	0,388	0,02595	0
GSPATT00014629001	0,582	0,00301	0	GSPATT00034075001	-0,137	0,30288	0
GSPATT00014645001	0,006	0,97144	0	GSPATT00034090001	0,737	0,00209	0
GSPATT00014660001	0,098	0,64509	0	GSPATT00034103001	-0,258	0,10215	0
GSPATT00014673001	-0,235	0,2283	0	GSPATT00034104001	0,705	0,00489	0
GSPATT00014674001	0,791	0,00595	0	GSPATT00034124001	-1,017	0,0022	-1
GSPATT00014676001	0,429	0,07671	0	GSPATT00034141001	0,708	0,00161	0
GSPATT00014685001	0,887	0,0008	0	GSPATT00034156001	-0,057	0,72295	0
GSPATT00014692001	-0,278	0,14785	0	GSPATT00034171001	0,663	0,00966	0
GSPATT00014705001	0,351	0,08889	0	GSPATT00034175001	-0,329	0,08396	0
GSPATT00014745001	0,289	0,08436	0	GSPATT00034216001	0,528	0,01503	0
GSPATT00014759001	1,203	0,00129	1	GSPATT00034230001	0,16	0,26608	0
GSPATT00014766001	0,379	0,03636	0	GSPATT00034233001	0,369	0,08874	0
GSPATT00014793001	-0,201	0,47536	0	GSPATT00034270001	1,032	0,00088	1
GSPATT00014796001	1,387	0,00001	1	GSPATT00034305001	0,927	0,0006	0
GSPATT00014810001	0,609	0,01875	0	GSPATT00034308001	-0,353	0,08333	0
GSPATT00014841001	0,356	0,10216	0	GSPATT00034314001	-1,355	0,00021	-1
GSPATT00014872001	0,244	0,12625	0	GSPATT00034329001	1,249	0,00003	1
GSPATT00014873001	0,194	0,50101	0	GSPATT00034393001	-1,986	0,00002	-1
GSPATT00014895001	0,711	0,00204	0	GSPATT00034405001	-0,584	0,00941	0
GSPATT00014900001	0,37	0,05706	0	GSPATT00034420001	-0,659	0,00959	0
GSPATT00014918001	-0,327	0,04948	0	GSPATT00034421001	0,663	0,12721	0
GSPATT00014958001	-0,462	0,20687	0	GSPATT00034463001	0,197	0,40106	0
GSPATT00014962001	-0,017	0,92935	0	GSPATT00034473001	0,036	0,77796	0
GSPATT00014966001	-0,912	0,0003	0	GSPATT00034552001	1,257	0,00015	1
GSPATT00014999001	0,415	0,07919	0	GSPATT00034577001	-0,839	0,00058	0
GSPATT00015015001	0,687	0,00414	0	GSPATT00034596001	0,345	0,26159	0
GSPATT00015017001	-1,086	0,00027	-1	GSPATT00034603001	0,885	0,0034	0
GSPATT00015018001	-0,08	0,70257	0	GSPATT00034615001	0,724	0,00057	0

GSPATT00015021001	-1,722	0,00006	-1	GSPATT00034623001	0,426	0,02613	0
GSPATT00015033001	-0,23	0,21296	0	GSPATT00034631001	0,527	0,0108	0
GSPATT00015042001	-0,895	0,00041	0	GSPATT00034632001	-0,156	0,30353	0
GSPATT00015043001	0,268	0,46979	0	GSPATT00034637001	0,89	0,00286	0
GSPATT00015055001	0,271	0,36469	0	GSPATT00034640001	1,246	0,00004	1
GSPATT00015064001	0,376	0,08595	0	GSPATT00034647001	0,189	0,18304	0
GSPATT00015109001	-0,005	0,9692	0	GSPATT00034648001	0,251	0,12628	0
GSPATT00015151001	0,296	0,07883	0	GSPATT00034657001	0,421	0,01754	0
GSPATT00015163001	-0,077	0,79332	0	GSPATT00034667001	-0,213	0,27669	0
GSPATT00015169001	0,432	0,17161	0	GSPATT00034770001	0,27	0,13463	0
GSPATT00015210001	1,87	0,00003	1	GSPATT00034806001	-0,248	0,17157	0
GSPATT00015238001	1,322	0,00004	1	GSPATT00034819001	0,638	0,01374	0
GSPATT00015246001	0,577	0,07312	0	GSPATT00034827001	-0,31	0,07036	0
GSPATT00015257001	-0,315	0,32799	0	GSPATT00034862001	0,911	0,00088	0
GSPATT00015261001	0,509	0,06899	0	GSPATT00034863001	-2,801	0	-1
GSPATT00015299001	0,213	0,19618	0	GSPATT00034885001	0,254	0,14736	0
GSPATT00015301001	0,024	0,90708	0	GSPATT00034888001	0,387	0,09919	0
GSPATT00015311001	0,153	0,48604	0	GSPATT00034893001	0,489	0,0257	0
GSPATT00015312001	-0,675	0,00141	0	GSPATT00034900001	0,251	0,34442	0
GSPATT00015325001	0,179	0,42392	0	GSPATT00034902001	-0,054	0,71793	0
GSPATT00015335001	0,857	0,00098	0	GSPATT00034911001	0,966	0,00178	0
GSPATT00015344001	0,355	0,08801	0	GSPATT00034927001	0,954	0,00128	0
GSPATT00015351001	0,593	0,00674	0	GSPATT00034954001	-0,254	0,17321	0
GSPATT00015356001	-0,33	0,11053	0	GSPATT00034968001	0,919	0,00041	0
GSPATT00015363001	-0,898	0,00028	0	GSPATT00034975001	-0,694	0,00453	0
GSPATT00015373001	0,163	0,24261	0	GSPATT00034994001	0,228	0,11464	0
GSPATT00015374001	0,211	0,22091	0	GSPATT00034997001	0,566	0,00681	0
GSPATT00015382001	1,396	0,00009	1	GSPATT00035001001	0,72	0,00113	0
GSPATT00015396001	0,853	0,02037	0	GSPATT00035014001	0,215	0,22573	0
GSPATT00015413001	1,181	0,00207	1	GSPATT00035015001	-0,804	0,00518	0
GSPATT00015428001	-0,166	0,38106	0	GSPATT00035041001	0,7	0,01007	0
GSPATT00015448001	1,213	0,00004	1	GSPATT00035046001	-0,126	0,63021	0
GSPATT00015474001	0,646	0,00966	0	GSPATT00035048001	-0,034	0,8154	0
GSPATT00015475001	0,366	0,0282	0	GSPATT00035049001	1,06	0,00012	1
GSPATT00015479001	0,147	0,2715	0	GSPATT00035050001	1,458	0,0075	1
GSPATT00015586001	-0,146	0,30933	0	GSPATT00035051001	1,15	0,00008	1
GSPATT00015587001	-0,173	0,20164	0	GSPATT00035055001	-2,179	0	-1
GSPATT00015588001	0,437	0,0437	0	GSPATT00035058001	0,603	0,00388	0
GSPATT00015597001	0,345	0,07945	0	GSPATT00035098001	-0,426	0,01033	0
GSPATT00015625001	-1,091	0,00006	-1	GSPATT00035101001	0,274	0,13516	0
GSPATT00015632001	1,616	0,00334	1	GSPATT00035109001	0,17	0,21947	0
GSPATT00015635001	0,054	0,76188	0	GSPATT00035110001	0,776	0,02913	0
GSPATT00015640001	0,853	0,00185	0	GSPATT00035113001	-3,146	0	-1
GSPATT00015666001	-0,623	0,00761	0	GSPATT00035115001	0,815	0,01116	0
GSPATT00015671001	-0,147	0,30793	0	GSPATT00035116001	0,615	0,00474	0
GSPATT00015678001	0,121	0,52246	0	GSPATT00035132001	0,541	0,03772	0
GSPATT00015687001	0,716	0,00115	0	GSPATT00035156001	0,909	0,02167	0
GSPATT00015690001	0,723	0,00416	0	GSPATT00035162001	0,462	0,02625	0
GSPATT00015693001	-0,397	0,09858	0	GSPATT00035168001	-0,967	0,00127	0
GSPATT00015702001	0,703	0,00191	0	GSPATT00035170001	0,275	0,20192	0
GSPATT00015703001	0,498	0,0388	0	GSPATT00035215001	-0,514	0,05916	0
GSPATT00015729001	0,223	0,18041	0	GSPATT00035221001	1,144	0,00247	1
GSPATT00015750001	1,122	0,00018	1	GSPATT00035230001	0,202	0,239	0
GSPATT00015756001	0,261	0,13282	0	GSPATT00035231001	0,708	0,00161	0
GSPATT00015783001	0,994	0,00013	0	GSPATT00035242001	0,289	0,32037	0
GSPATT00015795001	1,253	0,00003	1	GSPATT00035265001	-0,98	0,00136	0
GSPATT00015804001	-0,028	0,89234	0	GSPATT00035273001	0,212	0,15905	0
GSPATT00015821001	-0,236	0,16594	0	GSPATT00035283001	0,586	0,00587	0
GSPATT00015826001	-1,025	0,00242	-1	GSPATT00035344001	1,515	0,00002	1
GSPATT00015830001	2,01	0,00006	1	GSPATT00035345001	0,075	0,59803	0
GSPATT00015852001	0,793	0,01264	0	GSPATT00035369001	1,788	0	1
GSPATT00015854001	-0,969	0,00154	0	GSPATT00035370001	0,774	0,00252	0
GSPATT00015864001	0,541	0,09018	0	GSPATT00035435001	0,252	0,14704	0
GSPATT00015881001	0,435	0,06228	0	GSPATT00035439001	0,245	0,18477	0
GSPATT00015890001	0,521	0,0327	0	GSPATT00035512001	0,145	0,51374	0
GSPATT00015896001	0,187	0,19576	0	GSPATT00035537001	-0,316	0,13551	0
GSPATT00015906001	-0,376	0,07667	0	GSPATT00035553001	0,19	0,42969	0

GSPATT00015913001	-3,714	0	-1	GSPATT00035558001	0,435	0,06307	0
GSPATT00015946001	-0,176	0,47867	0	GSPATT00035563001	0,506	0,20376	0
GSPATT00015989001	0,756	0,0358	0	GSPATT00035570001	0,438	0,14386	0
GSPATT00015997001	0,616	0,05511	0	GSPATT00035586001	0,216	0,24577	0
GSPATT00015998001	0,057	0,70953	0	GSPATT00035588001	1,375	0,00072	1
GSPATT00016007001	-1,006	0,00099	-1	GSPATT00035590001	-0,098	0,61368	0
GSPATT00016014001	0,361	0,04847	0	GSPATT00035599001	0,523	0,00967	0
GSPATT00016022001	0,297	0,04559	0	GSPATT00035613001	0,93	0,00031	0
GSPATT00016031001	1,983	0	1	GSPATT00035630001	0,599	0,00235	0
GSPATT00016054001	-0,06	0,68875	0	GSPATT00035632001	1,647	0,00001	1
GSPATT00016058001	0,403	0,0261	0	GSPATT00035666001	0,319	0,04691	0
GSPATT00016068001	0,584	0,00865	0	GSPATT00035684001	0,079	0,61168	0
GSPATT00016075001	0,476	0,01419	0	GSPATT00035689001	-0,122	0,45197	0
GSPATT00016078001	-0,46	0,01198	0	GSPATT00035711001	-0,148	0,32112	0
GSPATT00016099001	-0,376	0,0569	0	GSPATT00035742001	-0,345	0,03219	0
GSPATT00016118001	0,681	0,00081	0	GSPATT00035743001	-0,119	0,44406	0
GSPATT00016152001	0,24	0,11484	0	GSPATT00035748001	0,686	0,00459	0
GSPATT00016163001	0,936	0,00075	0	GSPATT00035791001	-0,365	0,03388	0
GSPATT00016186001	0,651	0,00328	0	GSPATT00035808001	-0,036	0,8091	0
GSPATT00016201001	0,264	0,12078	0	GSPATT00035812001	0,025	0,89409	0
GSPATT00016202001	0,942	0,0002	0	GSPATT00035830001	0,647	0,0186	0
GSPATT00016222001	-0,197	0,20303	0	GSPATT00035834001	0,834	0,00201	0
GSPATT00016224001	0,577	0,00541	0	GSPATT00035841001	-0,305	0,05898	0
GSPATT00016246001	1,272	0,00554	1	GSPATT00035845001	0,903	0,0002	0
GSPATT00016252001	-1,875	0,00002	-1	GSPATT00035846001	-0,018	0,91105	0
GSPATT00016286001	0,878	0,0129	0	GSPATT00035878001	-0,682	0,01003	0
GSPATT00016304001	0,003	0,98948	0	GSPATT00035892001	0,871	0,00133	0
GSPATT00016323001	0,645	0,02514	0	GSPATT00035967001	-1,028	0,00178	-1
GSPATT00016324001	0,417	0,15142	0	GSPATT00035984001	0,033	0,81622	0
GSPATT00016327001	0,649	0,00694	0	GSPATT00035987001	0,061	0,629	0
GSPATT00016354001	1,499	0,00002	1	GSPATT00035988001	0,121	0,62093	0
GSPATT00016370001	0,574	0,02089	0	GSPATT00035996001	1,583	0,00009	1
GSPATT00016374001	0,711	0,02701	0	GSPATT00036001001	0,324	0,09202	0
GSPATT00016390001	-0,025	0,88764	0	GSPATT00036011001	0,418	0,0183	0
GSPATT00016393001	0,939	0,0007	0	GSPATT00036020001	0,633	0,01487	0
GSPATT00016396001	-0,359	0,34319	0	GSPATT00036021001	-0,784	0,08263	0
GSPATT00016407001	-0,858	0,00048	0	GSPATT00036025001	-0,396	0,01622	0
GSPATT00016433001	0,343	0,0523	0	GSPATT00036029001	0,388	0,03177	0
GSPATT00016451001	0,509	0,00351	0	GSPATT00036032001	-0,747	0,00511	0
GSPATT00016461001	2,349	0	1	GSPATT00036035001	1,064	0,00084	1
GSPATT00016467001	0,07	0,6374	0	GSPATT00036037001	0,29	0,15131	0
GSPATT00016473001	-0,231	0,19236	0	GSPATT00036059001	-2,602	0,00001	-1
GSPATT00016474001	-0,225	0,18313	0	GSPATT00036080001	-0,995	0,00114	0
GSPATT00016484001	0,021	0,92665	0	GSPATT00036130001	1,008	0,00009	1
GSPATT00016487001	0,393	0,10186	0	GSPATT00036133001	0,425	0,08763	0
GSPATT00016492001	1,714	0,00007	1	GSPATT00036142001	0,347	0,06142	0
GSPATT00016497001	0,83	0,00243	0	GSPATT00036146001	-0,276	0,13155	0
GSPATT00016499001	2,913	0	1	GSPATT00036182001	0,139	0,41765	0
GSPATT00016501001	0,408	0,05581	0	GSPATT00036198001	0,154	0,39188	0
GSPATT00016508001	-0,185	0,31276	0	GSPATT00036212001	0,53	0,00883	0
GSPATT00016522001	1,486	0,00001	1	GSPATT00036217001	-0,481	0,07457	0
GSPATT00016536001	0,338	0,11356	0	GSPATT00036238001	0,234	0,24062	0
GSPATT00016547001	-0,209	0,18719	0	GSPATT00036250001	0,42	0,01334	0
GSPATT00016555001	0,08	0,62165	0	GSPATT00036252001	-0,676	0,01753	0
GSPATT00016572001	0,158	0,27397	0	GSPATT00036267001	1,177	0,00036	1
GSPATT00016579001	-0,042	0,75571	0	GSPATT00036286001	-0,683	0,00156	0
GSPATT00016580001	1,975	0	1	GSPATT00036336001	0,222	0,12243	0
GSPATT00016581001	0,583	0,00498	0	GSPATT00036337001	0,687	0,00442	0
GSPATT00016584001	0,48	0,00759	0	GSPATT00036353001	0,737	0,00133	0
GSPATT00016598001	-0,315	0,17406	0	GSPATT00036372001	-0,154	0,42691	0
GSPATT00016621001	-0,635	0,00167	0	GSPATT00036407001	0,356	0,04968	0
GSPATT00016625001	0,578	0,01915	0	GSPATT00036409001	0,316	0,04048	0
GSPATT00016626001	-1,01	0,01795	-1	GSPATT00036434001	0,218	0,17014	0
GSPATT00016635001	1,276	0,00002	1	GSPATT00036445001	0,343	0,02548	0
GSPATT00016642001	0,83	0,00038	0	GSPATT00036459001	1,229	0,0001	1
GSPATT00016644001	-0,085	0,60496	0	GSPATT00036469001	0,587	0,01017	0
GSPATT00016649001	0,549	0,0028	0	GSPATT00036471001	0,051	0,77388	0

GSPATT00016655001	-0,315	0,1207	0	GSPATT00036474001	0,054	0,83167	0
GSPATT00016684001	0,371	0,03571	0	GSPATT00036479001	-0,35	0,11614	0
GSPATT00016686001	-0,649	0,0057	0	GSPATT00036483001	-0,011	0,94317	0
GSPATT00016687001	-1,172	0,00022	-1	GSPATT00036486001	-0,301	0,19234	0
GSPATT00016692001	0,905	0,00153	0	GSPATT00036520001	1,333	0,00049	1
GSPATT00016707001	0,035	0,81827	0	GSPATT00036521001	-1,453	0,00724	-1
GSPATT00016715001	-0,125	0,40578	0	GSPATT00036542001	2,353	0	1
GSPATT00016728001	0,796	0,00369	0	GSPATT00036543001	3,458	0	1
GSPATT00016739001	0,05	0,81698	0	GSPATT00036578001	0,012	0,94123	0
GSPATT00016753001	0,303	0,03871	0	GSPATT00036602001	-0,016	0,91827	0
GSPATT00016756001	0,551	0,01981	0	GSPATT00036604001	0,849	0,00077	0
GSPATT00016834001	-0,177	0,2236	0	GSPATT00036632001	0,404	0,0432	0
GSPATT00016841001	0,199	0,27702	0	GSPATT00036666001	-0,42	0,03381	0
GSPATT00016868001	-0,668	0,00534	0	GSPATT00036671001	-0,905	0,00646	0
GSPATT00016887001	-0,259	0,12658	0	GSPATT00036678001	0,014	0,9207	0
GSPATT00016943001	-0,089	0,60758	0	GSPATT00036688001	0,31	0,04238	0
GSPATT00016964001	0,04	0,87452	0	GSPATT00036690001	0,725	0,00628	0
GSPATT00016974001	-0,017	0,9121	0	GSPATT00036705001	1,156	0,00008	1
GSPATT00016976001	1,509	0,00004	1	GSPATT00036706001	0,883	0,00746	0
GSPATT00016985001	0,32	0,1398	0	GSPATT00036730001	-0,04	0,79056	0
GSPATT00017038001	2	0	1	GSPATT00036752001	0,349	0,08868	0
GSPATT00017050001	0,473	0,00736	0	GSPATT00036770001	0,189	0,34256	0
GSPATT00017063001	0,061	0,69591	0	GSPATT00036776001	-0,3	0,06534	0
GSPATT00017094001	0,37	0,12392	0	GSPATT00036800001	-0,04	0,79823	0
GSPATT00017107001	0,512	0,01311	0	GSPATT00036806001	-0,03	0,88012	0
GSPATT00017117001	1,483	0,00007	1	GSPATT00036817001	-0,834	0,01188	0
GSPATT00017122001	-0,89	0,00112	0	GSPATT00036847001	-0,022	0,88003	0
GSPATT00017148001	0,445	0,01161	0	GSPATT00036862001	-1,618	0,00012	-1
GSPATT00017157001	0,228	0,29231	0	GSPATT00036876001	0,655	0,01693	0
GSPATT00017193001	0,423	0,02362	0	GSPATT00036879001	0,685	0,00059	0
GSPATT00017227001	0,713	0,01736	0	GSPATT00036888001	0,452	0,00944	0
GSPATT00017228001	-1,143	0,00016	-1	GSPATT00036914001	0,754	0,10066	0
GSPATT00017248001	0,004	0,98061	0	GSPATT00036919001	-0,269	0,14769	0
GSPATT00017265001	0,224	0,27585	0	GSPATT00036954001	-0,592	0,0063	0
GSPATT00017287001	0,722	0,00261	0	GSPATT00036971001	-0,218	0,16152	0
GSPATT00017341001	-0,483	0,07425	0	GSPATT00037000001	0,283	0,14601	0
GSPATT00017350001	-0,237	0,16156	0	GSPATT00037053001	-0,135	0,29881	0
GSPATT00017354001	-0,088	0,60425	0	GSPATT00037058001	0,084	0,66195	0
GSPATT00017356001	1,623	0,00011	1	GSPATT00037066001	-0,414	0,01584	0
GSPATT00017361001	0,54	0,0029	0	GSPATT00037088001	0,441	0,01862	0
GSPATT00017388001	-0,64	0,00112	0	GSPATT00037094001	-0,008	0,9585	0
GSPATT00017455001	0,77	0,0007	0	GSPATT00037113001	-0,126	0,51661	0
GSPATT00017456001	0,739	0,00754	0	GSPATT00037123001	0,774	0,01287	0
GSPATT00017492001	0,873	0,0003	0	GSPATT00037129001	0,656	0,00104	0
GSPATT00017522001	0,536	0,05133	0	GSPATT00037143001	-0,027	0,85661	0
GSPATT00017543001	1,645	0,00001	1	GSPATT00037145001	0,616	0,02922	0
GSPATT00017557001	0,172	0,32619	0	GSPATT00037148001	-0,358	0,09184	0
GSPATT00017560001	1,038	0,00731	1	GSPATT00037154001	-0,557	0,01543	0
GSPATT00017569001	-0,582	0,01474	0	GSPATT00037160001	1,034	0,00084	1
GSPATT00017576001	1,079	0,00068	1	GSPATT00037166001	1	0,00065	0
GSPATT00017630001	-0,188	0,2095	0	GSPATT00037168001	0,397	0,02983	0
GSPATT00017662001	0,243	0,10339	0	GSPATT00037172001	0,145	0,33707	0
GSPATT00017666001	0,164	0,29665	0	GSPATT00037211001	0,931	0,00079	0
GSPATT00017671001	1,215	0,00163	1	GSPATT00037212001	0,689	0,00673	0
GSPATT00017672001	0,666	0,00486	0	GSPATT00037226001	0,202	0,30966	0
GSPATT00017676001	0,897	0,00014	0	GSPATT00037232001	0,667	0,00174	0
GSPATT00017680001	1,263	0,0002	1	GSPATT00037285001	0,579	0,00719	0
GSPATT00017681001	1,015	0,00199	1	GSPATT00037291001	-2,613	0,00006	-1
GSPATT00017692001	0,757	0,02894	0	GSPATT00037311001	-0,268	0,09834	0
GSPATT00017712001	-0,359	0,17781	0	GSPATT00037328001	0,772	0,00301	0
GSPATT00017715001	1,019	0,00017	1	GSPATT00037330001	0,474	0,01266	0
GSPATT00017746001	0,26	0,0958	0	GSPATT00037368001	1,031	0,0019	1
GSPATT00017766001	-1,364	0,00026	-1	GSPATT00037376001	0,236	0,20339	0
GSPATT00017771001	0,257	0,1542	0	GSPATT00037398001	0,097	0,63269	0
GSPATT00017791001	-1,005	0,00013	-1	GSPATT00037444001	-0,662	0,00643	0
GSPATT00017820001	0,089	0,63426	0	GSPATT00037451001	0,414	0,01229	0
GSPATT00017833001	1,804	0,00001	1	GSPATT00037471001	0,104	0,58331	0

GSPATT00017845001	-0,337	0,04954	0	GSPATT00037488001	1,394	0,00067	1
GSPATT00017866001	-0,949	0,00081	0	GSPATT00037526001	0,897	0,00043	0
GSPATT00017868001	-0,188	0,4063	0	GSPATT00037566001	-0,05	0,76612	0
GSPATT00017882001	-0,011	0,94767	0	GSPATT00037597001	-0,102	0,55503	0
GSPATT00017902001	-0,048	0,73859	0	GSPATT00037660001	0,186	0,17268	0
GSPATT00017904001	0,196	0,20412	0	GSPATT00037661001	0,066	0,71158	0
GSPATT00017915001	0,763	0,00829	0	GSPATT00037688001	0,303	0,04893	0
GSPATT00017926001	-0,085	0,54936	0	GSPATT00037699001	0,741	0,00075	0
GSPATT00017952001	0,822	0,00042	0	GSPATT00037711001	-0,444	0,05547	0
GSPATT00017953001	0,93	0,00178	0	GSPATT00037715001	0,109	0,42522	0
GSPATT00017960001	-0,674	0,00313	0	GSPATT00037722001	0,821	0,0005	0
GSPATT00017963001	-0,275	0,06466	0	GSPATT00037727001	0,543	0,04234	0
GSPATT00017966001	0,227	0,14352	0	GSPATT00037737001	1,364	0,0001	1
GSPATT00017972001	-0,131	0,38508	0	GSPATT00037754001	-0,856	0,0023	0
GSPATT00017992001	-0,11	0,50579	0	GSPATT00037781001	-1,255	0,00102	-1
GSPATT00018004001	0,463	0,03497	0	GSPATT00037784001	0,072	0,57853	0
GSPATT00018101001	0,898	0,00614	0	GSPATT00037789001	2,068	0	1
GSPATT00018127001	0,153	0,36392	0	GSPATT00037792001	-0,369	0,18448	0
GSPATT00018132001	0,741	0,02921	0	GSPATT00037795001	0,362	0,01982	0
GSPATT00018133001	-0,081	0,66594	0	GSPATT00037796001	1,094	0,0001	1
GSPATT00018143001	0,268	0,2351	0	GSPATT00037798001	0,035	0,81328	0
GSPATT00018153001	-0,086	0,72806	0	GSPATT00037811001	0,21	0,2921	0
GSPATT00018163001	0,342	0,02849	0	GSPATT00037815001	0,925	0,00331	0
GSPATT00018188001	0,221	0,21776	0	GSPATT00037818001	0,928	0,00072	0
GSPATT00018202001	0,66	0,01285	0	GSPATT00037856001	0,303	0,06934	0
GSPATT00018223001	-0,409	0,02988	0	GSPATT00037875001	-0,066	0,66305	0
GSPATT00018263001	0,814	0,00153	0	GSPATT00037876001	0,446	0,01048	0
GSPATT00018280001	0,316	0,0745	0	GSPATT00037939001	0,394	0,02006	0
GSPATT00018290001	-0,359	0,04447	0	GSPATT00037945001	-0,437	0,02189	0
GSPATT00018291001	0,141	0,45858	0	GSPATT00037967001	-0,06	0,6776	0
GSPATT00018293001	0,169	0,30306	0	GSPATT00037978001	0,461	0,08571	0
GSPATT00018324001	0,468	0,06312	0	GSPATT00038025001	-0,104	0,48304	0
GSPATT00018389001	-0,094	0,58557	0	GSPATT00038027001	0,502	0,05619	0
GSPATT00018393001	-0,203	0,16787	0	GSPATT00038035001	0,888	0,00025	0
GSPATT00018398001	0,481	0,09586	0	GSPATT00038045001	-0,396	0,05929	0
GSPATT00018431001	0,56	0,04386	0	GSPATT00038090001	1,233	0,00044	1
GSPATT00018438001	0,545	0,01322	0	GSPATT00038149001	0,258	0,07307	0
GSPATT00018449001	0,58	0,0723	0	GSPATT00038155001	0,75	0,00768	0
GSPATT00018471001	-1,113	0,00027	-1	GSPATT00038156001	0,107	0,61347	0
GSPATT00018476001	0,992	0,00041	0	GSPATT00038162001	-0,205	0,24742	0
GSPATT00018515001	0,206	0,16244	0	GSPATT00038171001	-1,199	0,00032	-1
GSPATT00018529001	0,92	0,01084	0	GSPATT00038175001	-0,718	0,00151	0
GSPATT00018559001	0,48	0,01489	0	GSPATT00038196001	0,025	0,86468	0
GSPATT00018563001	0,407	0,05649	0	GSPATT00038198001	1,751	0,00019	1
GSPATT00018600001	0,51	0,01239	0	GSPATT00038202001	1,049	0,00014	1
GSPATT00018603001	-0,883	0,002	0	GSPATT00038203001	1,279	0,00007	1
GSPATT00018608001	0,219	0,14898	0	GSPATT00038212001	0,313	0,14446	0
GSPATT00018634001	-0,253	0,0798	0	GSPATT00038214001	2,959	0	1
GSPATT00018635001	-4,269	0	-1	GSPATT00038217001	1,138	0,00032	1
GSPATT00018645001	-0,232	0,15845	0	GSPATT00038232001	-1,557	0,00002	-1
GSPATT00018660001	-0,45	0,12247	0	GSPATT00038245001	0,014	0,92514	0
GSPATT00018666001	0,125	0,44695	0	GSPATT00038377001	1,241	0,00049	1
GSPATT00018671001	0,675	0,00088	0	GSPATT00038439001	0,034	0,86173	0
GSPATT00018681001	-0,683	0,00496	0	GSPATT00038442001	0,573	0,00219	0
GSPATT00018695001	-0,076	0,62948	0	GSPATT00038453001	0,958	0,00056	0
GSPATT00018702001	-0,494	0,02855	0	GSPATT00038457001	0,034	0,80944	0
GSPATT00018706001	0,301	0,2524	0	GSPATT00038462001	0,25	0,17848	0
GSPATT00018710001	-0,217	0,14626	0	GSPATT00038469001	1,122	0,00009	1
GSPATT00018727001	-0,029	0,84109	0	GSPATT00038489001	0,49	0,00846	0
GSPATT00018728001	-0,429	0,04057	0	GSPATT00038497001	0,218	0,14041	0
GSPATT00018737001	0,112	0,42429	0	GSPATT00038500001	-0,057	0,70837	0
GSPATT00018780001	0,691	0,01868	0	GSPATT00038501001	0,115	0,39982	0
GSPATT00018806001	-0,089	0,50827	0	GSPATT00038509001	0,207	0,13138	0
GSPATT00018878001	-0,526	0,01436	0	GSPATT00038517001	0,388	0,06212	0
GSPATT00018891001	-0,275	0,12204	0	GSPATT00038525001	0,766	0,00172	0
GSPATT00018918001	0,815	0,00108	0	GSPATT00038565001	-0,503	0,00816	0
GSPATT00018920001	-0,177	0,49925	0	GSPATT00038594001	-0,531	0,01391	0

GSPATT00018950001	0,098	0,47385	0
GSPATT00018952001	0,199	0,36354	0
GSPATT00018955001	-1,697	0,00048	-1
GSPATT00018964001	0,792	0,02949	0
GSPATT00018965001	0,578	0,00897	0
GSPATT00018969001	-0,459	0,03222	0
GSPATT00018978001	1,009	0,00294	1
GSPATT00019012001	0,241	0,25273	0
GSPATT00019047001	-0,313	0,12451	0
GSPATT00019066001	0,738	0,00884	0
GSPATT00019068001	0,352	0,08916	0
GSPATT00019089001	-0,092	0,50555	0
GSPATT00019098001	-0,396	0,01375	0
GSPATT00019105001	-0,564	0,03875	0
GSPATT00019121001	-0,238	0,13986	0
GSPATT00019142001	0,119	0,67944	0
GSPATT00019147001	1,334	0,00037	1
GSPATT00019217001	0,697	0,00662	0
GSPATT00019232001	1,248	0,0003	1
GSPATT00019271001	0,448	0,01195	0
GSPATT00019272001	0,626	0,00167	0
GSPATT00019281001	0,242	0,15235	0
GSPATT00019357001	-0,269	0,11687	0
GSPATT00019361001	1,018	0,00339	1
GSPATT00019366001	0,029	0,8276	0
GSPATT00019371001	-1,072	0,00005	-1
GSPATT00019421001	0,515	0,01534	0
GSPATT00019447001	1,328	0,00111	1
GSPATT00019448001	1,274	0,00003	1
GSPATT00019460001	0,582	0,01671	0
GSPATT00019477001	-0,546	0,02587	0
GSPATT00019493001	-0,409	0,16665	0
GSPATT00019504001	0,507	0,03568	0
GSPATT00019520001	1,19	0,00006	1
GSPATT00019546001	0,939	0,00501	0
GSPATT00019584001	0,197	0,24399	0
GSPATT00019589001	-0,23	0,14791	0
GSPATT00019597001	0,628	0,00396	0
GSPATT00038759001	0,112	0,58404	0
GSPATT00038767001	0,906	0,00064	0
GSPATT00038772001	0,189	0,25284	0
GSPATT00038778001	0,45	0,0114	0
GSPATT00038811001	0,163	0,23056	0
GSPATT00038843001	0,394	0,08578	0
GSPATT00038849001	0,132	0,34446	0
GSPATT00038851001	-0,622	0,0036	0
GSPATT00038853001	0,357	0,03403	0
GSPATT00038854001	0,067	0,67394	0
GSPATT00038872001	-0,347	0,10873	0
GSPATT00038895001	-2,984	0,00003	-1
GSPATT00038896001	-0,151	0,65702	0
GSPATT00038914001	1,241	0,00033	1
GSPATT00038921001	-0,019	0,91788	0
GSPATT00038932001	0,144	0,35304	0
GSPATT00038938001	0,67	0,06631	0
GSPATT00039020001	1,009	0,00006	1
GSPATT00039053001	0,586	0,00254	0
GSPATT00039058001	0,227	0,19888	0
GSPATT00039143001	0,942	0,00145	0
GSPATT00039147001	-0,456	0,10233	0
GSPATT00039305001	0,494	0,01965	0
GSPATT00039325001	0,34	0,08666	0
GSPATT00039337001	0,067	0,62405	0
GSPATT00039369001	-0,01	0,94511	0
GSPATT00039403001	1,013	0,00053	1
GSPATT00039502001	-0,434	0,08958	0
GSPATT00039519001	0,599	0,01071	0
GSPATT00039578001	1,071	0,0029	1
GSPATT00039596001	-0,065	0,63218	0
GSPATT00039617001	-0,125	0,38541	0
GSPATT00039623001	-0,179	0,37866	0
GSPATT00039672001	0,81	0,01218	0
GSPATT00039701001	0,824	0,06378	0
GSPATT00039733001	0,707	0,00233	0
GSPATT00039825001	0,881	0,00181	0
PTETT10500005001	0,689	0,01507	0
PTETT10800002001	-0,131	0,51208	0

GO 0006464 protein modification process

ID	coeff.	p-value	signif.
GSPATT00000029001	-0,656	0,02257	0
GSPATT00000047001	0,686	0,01107	0
GSPATT00000064001	-0,605	0,00365	0
GSPATT00000091001	0,592	0,02302	0
GSPATT00000093001	0,319	0,15526	0
GSPATT00000160001	-0,436	0,09808	0
GSPATT00000190001	0,762	0,00321	0
GSPATT00000248001	0,46	0,00681	0
GSPATT00000282001	-0,179	0,30932	0
GSPATT00000295001	0,583	0,02578	0
GSPATT00000324001	-0,092	0,63602	0
GSPATT00000365001	0,81	0,00102	0
GSPATT00000366001	0,639	0,01895	0
GSPATT00000384001	0,653	0,02308	0
GSPATT00000394001	-0,275	0,16617	0
GSPATT00000415001	-0,571	0,00652	0
GSPATT00000434001	0,504	0,02973	0
GSPATT00000482001	-0,044	0,82968	0
GSPATT00000508001	-0,009	0,95625	0
GSPATT00000542001	0,969	0,00094	0
GSPATT00000559001	0,401	0,04709	0
GSPATT00000560001	0,804	0,0092	0
GSPATT00000594001	-1,714	0,00001	-1
GSPATT00000596001	-1,334	0,00022	-1
GSPATT00000599001	-0,212	0,13935	0
GSPATT00000615001	0,04	0,80664	0
GSPATT00019584001	0,197	0,24399	0
GSPATT00019589001	-0,23	0,14791	0
GSPATT00019597001	0,628	0,00396	0
GSPATT00019614001	-0,092	0,48818	0
GSPATT00019621001	1,048	0,0001	1
GSPATT00019622001	0,562	0,00554	0
GSPATT00019623001	0,397	0,02171	0
GSPATT00019627001	0,267	0,13823	0
GSPATT00019634001	-0,335	0,03168	0
GSPATT00019648001	0,266	0,10254	0
GSPATT00019665001	0,352	0,16137	0
GSPATT00019672001	0,262	0,38511	0
GSPATT00019684001	0,599	0,03897	0
GSPATT00019701001	0,633	0,00548	0
GSPATT00019734001	0,538	0,01044	0
GSPATT00019758001	-0,857	0,00047	0
GSPATT00019770001	0,159	0,3745	0
GSPATT00019793001	0,172	0,36914	0
GSPATT00019834001	0,855	0,00735	0
GSPATT00019835001	0,65	0,03101	0
GSPATT00019840001	0,546	0,02765	0
GSPATT00019849001	1,004	0,00239	1
GSPATT00019861001	0,155	0,42691	0
GSPATT00019885001	1,059	0,00032	1
GSPATT00019888001	1,012	0,00029	1
GSPATT00019897001	0,216	0,1244	0

GSPATT00000619001	0,52	0,00518	0
GSPATT00000630001	0,066	0,80692	0
GSPATT00000664001	0,473	0,02458	0
GSPATT00000685001	0,283	0,12345	0
GSPATT00000704001	1,505	0,00001	1
GSPATT00000760001	1,018	0,00234	1
GSPATT00000783001	0,956	0,007	0
GSPATT00000795001	0,73	0,00542	0
GSPATT00000811001	0,048	0,81575	0
GSPATT00000817001	-0,289	0,11652	0
GSPATT00000822001	0,006	0,96587	0
GSPATT00000824001	1,895	0,00001	1
GSPATT00000926001	-0,44	0,07676	0
GSPATT00000934001	0,7	0,00134	0
GSPATT00000950001	-0,039	0,85119	0
GSPATT00000961001	1,244	0,00014	1
GSPATT00000975001	0,767	0,00162	0
GSPATT00000978001	0,54	0,00309	0
GSPATT00000985001	-0,053	0,78626	0
GSPATT00000988001	0,476	0,00614	0
GSPATT00001013001	0,18	0,3735	0
GSPATT00001029001	0,351	0,04038	0
GSPATT00001071001	-1,676	0,00003	-1
GSPATT00001077001	-1,834	0	-1
GSPATT00001082001	0,802	0,00363	0
GSPATT00001085001	-0,102	0,55835	0
GSPATT00001088001	-1,002	0,00029	-1
GSPATT00001107001	1,346	0,00005	1
GSPATT00001118001	0,362	0,12047	0
GSPATT00001133001	-0,118	0,39808	0
GSPATT00001155001	-0,688	0,04688	0
GSPATT00001162001	-0,208	0,17385	0
GSPATT00001174001	-0,137	0,61349	0
GSPATT00001183001	-1,126	0,00625	-1
GSPATT00001189001	-0,09	0,61561	0
GSPATT00001201001	-1,126	0,00014	-1
GSPATT00001205001	0,174	0,23944	0
GSPATT00001207001	0,62	0,00301	0
GSPATT00001216001	-0,859	0,0049	0
GSPATT00001230001	0,185	0,20391	0
GSPATT00001255001	-0,19	0,27269	0
GSPATT00001260001	1,21	0,00035	1
GSPATT00001268001	-0,542	0,00397	0
GSPATT00001273001	0,742	0,00126	0
GSPATT00001279001	0,193	0,33618	0
GSPATT00001292001	-0,196	0,32171	0
GSPATT00001310001	0,604	0,00221	0
GSPATT00001314001	0,32	0,07355	0
GSPATT00001320001	0,724	0,00683	0
GSPATT00001342001	0,712	0,00317	0
GSPATT00001359001	0,036	0,83426	0
GSPATT00001361001	0,243	0,21121	0
GSPATT00001369001	0,226	0,16319	0
GSPATT00001373001	0,174	0,38899	0
GSPATT00001413001	-0,893	0,00057	0
GSPATT00001414001	0,294	0,08325	0
GSPATT00001427001	0,007	0,96536	0
GSPATT00001430001	0,427	0,01633	0
GSPATT00001441001	-0,411	0,0844	0
GSPATT00001463001	1,174	0,00247	1
GSPATT00001467001	-0,474	0,02385	0
GSPATT00001471001	0,442	0,16312	0
GSPATT00001479001	0,193	0,3499	0
GSPATT00001494001	-0,073	0,59749	0
GSPATT00001504001	0,05	0,75727	0
GSPATT00001508001	0,42	0,01888	0
GSPATT00001527001	0,128	0,47689	0

GSPATT00019898001	0,291	0,35823	0
GSPATT00019899001	1,302	0,01819	1
GSPATT00019918001	1,36	0,00661	1
GSPATT00019930001	-0,02	0,89979	0
GSPATT00019935001	0,306	0,30175	0
GSPATT00019962001	0,101	0,63424	0
GSPATT00019970001	0,305	0,11022	0
GSPATT00019974001	0,112	0,7056	0
GSPATT00019988001	-0,077	0,59245	0
GSPATT00020006001	0,694	0,0227	0
GSPATT00020016001	-0,284	0,16066	0
GSPATT00020018001	0,567	0,00877	0
GSPATT00020040001	0,426	0,13971	0
GSPATT00020051001	0,424	0,02871	0
GSPATT00020056001	-0,263	0,09627	0
GSPATT00020061001	0,203	0,18858	0
GSPATT00020081001	0,562	0,01428	0
GSPATT00020083001	-0,007	0,96307	0
GSPATT00020106001	0,617	0,04707	0
GSPATT00020133001	0,915	0,00052	0
GSPATT00020136001	0,721	0,00761	0
GSPATT00020148001	0,628	0,00191	0
GSPATT00020151001	0,152	0,25786	0
GSPATT00020164001	0,049	0,79466	0
GSPATT00020171001	0,019	0,89222	0
GSPATT00020175001	-0,408	0,07148	0
GSPATT00020181001	-0,28	0,08622	0
GSPATT00020183001	0,379	0,08436	0
GSPATT00020206001	1,187	0,00008	1
GSPATT00020213001	-1,901	0	-1
GSPATT00020222001	0,022	0,8861	0
GSPATT00020225001	0,491	0,02758	0
GSPATT00020239001	-0,066	0,6879	0
GSPATT00020280001	0,417	0,00934	0
GSPATT00020292001	0,983	0,00426	0
GSPATT00020315001	0,421	0,02041	0
GSPATT00020317001	1,39	0,00016	1
GSPATT00020328001	0,132	0,45889	0
GSPATT00020354001	0,971	0,00377	0
GSPATT00020357001	0,423	0,11371	0
GSPATT00020367001	0,66	0,05352	0
GSPATT00020375001	0,159	0,26389	0
GSPATT00020379001	0,528	0,00684	0
GSPATT00020390001	-1,113	0,00014	-1
GSPATT00020397001	0,42	0,02881	0
GSPATT00020419001	-0,186	0,31238	0
GSPATT00020450001	0,494	0,01321	0
GSPATT00020501001	0,141	0,29138	0
GSPATT00020577001	0,213	0,12758	0
GSPATT00020591001	0,653	0,02277	0
GSPATT00020594001	0,125	0,38102	0
GSPATT00020603001	0,424	0,03461	0
GSPATT00020607001	0,553	0,03187	0
GSPATT00020634001	-0,891	0,00039	0
GSPATT00020656001	0,384	0,09254	0
GSPATT00020671001	0,4	0,06339	0
GSPATT00020675001	0,023	0,86196	0
GSPATT00020696001	1,227	0,00012	1
GSPATT00020706001	-2,177	0,00005	-1
GSPATT00020718001	0,498	0,0293	0
GSPATT00020723001	1,447	0,00002	1
GSPATT00020732001	0,346	0,02628	0
GSPATT00020736001	-0,014	0,93682	0
GSPATT00020738001	-0,47	0,01442	0
GSPATT00020743001	0,121	0,41116	0
GSPATT00020748001	0,638	0,0469	0
GSPATT00020754001	0,328	0,15362	0

GSPATT00001554001	0,882	0,00038	0	GSPATT00020755001	1,124	0,00008	1
GSPATT00001563001	-0,431	0,02903	0	GSPATT00020757001	0,109	0,4162	0
GSPATT00001592001	1,698	0,00001	1	GSPATT00020787001	0,074	0,69474	0
GSPATT00001610001	0,834	0,00147	0	GSPATT00020798001	-0,359	0,06684	0
GSPATT00001642001	-1,843	0,00008	-1	GSPATT00020807001	0,453	0,03103	0
GSPATT00001650001	0,32	0,08832	0	GSPATT00020812001	0,207	0,32984	0
GSPATT00001662001	0,438	0,17856	0	GSPATT00020816001	-0,014	0,954	0
GSPATT00001682001	-2,067	0,00007	-1	GSPATT00020817001	0,81	0,00504	0
GSPATT00001698001	-0,612	0,00264	0	GSPATT00020820001	0,296	0,15516	0
GSPATT00001746001	1,163	0,00005	1	GSPATT00020837001	-2,103	0	-1
GSPATT00001753001	0,102	0,57872	0	GSPATT00020856001	0,607	0,00205	0
GSPATT00001779001	0,661	0,00281	0	GSPATT00020863001	1,067	0,00014	1
GSPATT00001789001	0,667	0,00963	0	GSPATT00020867001	-0,219	0,23135	0
GSPATT00001794001	-0,276	0,08218	0	GSPATT00020886001	0,561	0,00314	0
GSPATT00001840001	0,602	0,00734	0	GSPATT00020887001	0,231	0,17424	0
GSPATT00001845001	-1,549	0,00003	-1	GSPATT00020897001	0,202	0,34688	0
GSPATT00001852001	1,949	0	1	GSPATT00020921001	0,201	0,27627	0
GSPATT00001853001	1,087	0,00026	1	GSPATT00020942001	-0,24	0,19484	0
GSPATT00001854001	1,3	0,00007	1	GSPATT00021004001	-0,039	0,80561	0
GSPATT00001859001	0,31	0,07571	0	GSPATT00021006001	-0,917	0,00215	0
GSPATT00001864001	0,864	0,00071	0	GSPATT00021015001	-0,087	0,60216	0
GSPATT00001865001	0,55	0,00393	0	GSPATT00021056001	-0,152	0,53854	0
GSPATT00001866001	0,294	0,40339	0	GSPATT00021080001	-0,038	0,88109	0
GSPATT00001870001	0,431	0,01514	0	GSPATT00021083001	1,214	0,00004	1
GSPATT00001871001	0,839	0,00105	0	GSPATT00021086001	0,414	0,02053	0
GSPATT00001873001	2,117	0,00001	1	GSPATT00021094001	0,597	0,01141	0
GSPATT00001911001	-0,107	0,62983	0	GSPATT00021104001	1,014	0,00009	1
GSPATT00001920001	0,423	0,0194	0	GSPATT00021108001	0,03	0,85909	0
GSPATT00002000001	0,994	0,00542	0	GSPATT00021127001	0,409	0,03217	0
GSPATT00002008001	0,799	0,00219	0	GSPATT00021152001	0,566	0,00438	0
GSPATT00002011001	0,938	0,00081	0	GSPATT00021161001	0,405	0,05228	0
GSPATT00002013001	0,586	0,02656	0	GSPATT00021175001	0,819	0,00079	0
GSPATT00002014001	0,546	0,01206	0	GSPATT00021195001	1,22	0,0001	1
GSPATT00002063001	0,381	0,02118	0	GSPATT00021201001	0,446	0,02497	0
GSPATT00002065001	0,716	0,00677	0	GSPATT00021207001	0,704	0,01544	0
GSPATT00002068001	1,376	0,00007	1	GSPATT00021213001	-0,327	0,35092	0
GSPATT00002077001	-0,063	0,72874	0	GSPATT00021222001	-1,069	0,00073	-1
GSPATT00002091001	0,425	0,05442	0	GSPATT00021227001	0,331	0,04285	0
GSPATT00002093001	1,052	0,00126	1	GSPATT00021248001	-0,222	0,12494	0
GSPATT00002094001	-0,116	0,53723	0	GSPATT00021249001	-0,183	0,27756	0
GSPATT00002107001	-0,282	0,23525	0	GSPATT00021250001	0,181	0,35664	0
GSPATT00002108001	0,114	0,44232	0	GSPATT00021252001	2,647	0	1
GSPATT00002114001	0,469	0,04573	0	GSPATT00021256001	0,688	0,00291	0
GSPATT00002115001	0,601	0,02057	0	GSPATT00021258001	1,026	0,00012	1
GSPATT00002129001	1,065	0,00431	1	GSPATT00021270001	0,517	0,01282	0
GSPATT00002132001	0,587	0,01286	0	GSPATT00021271001	0,429	0,01704	0
GSPATT00002156001	0,933	0,0007	0	GSPATT00021273001	1,843	0	1
GSPATT00002182001	1,186	0,00008	1	GSPATT00021274001	1,19	0,00002	1
GSPATT00002186001	0,08	0,52966	0	GSPATT00021275001	1,322	0,00075	1
GSPATT00002191001	1,024	0,00219	1	GSPATT00021294001	-0,03	0,86443	0
GSPATT00002195001	-0,541	0,01959	0	GSPATT00021304001	-0,938	0,00125	0
GSPATT00002196001	-0,094	0,51362	0	GSPATT00021337001	1,12	0,00026	1
GSPATT00002202001	-0,633	0,00593	0	GSPATT00021346001	0,927	0,00175	0
GSPATT00002221001	0,238	0,09743	0	GSPATT00021382001	0,681	0,00268	0
GSPATT00002233001	0,107	0,42961	0	GSPATT00021397001	-0,341	0,04105	0
GSPATT00002241001	-0,266	0,23687	0	GSPATT00021403001	0,008	0,96802	0
GSPATT00002247001	0,656	0,00298	0	GSPATT00021407001	0,546	0,01042	0
GSPATT00002269001	-2,667	0,00001	-1	GSPATT00021410001	0,546	0,03562	0
GSPATT00002272001	0,482	0,00978	0	GSPATT00021416001	0,262	0,11135	0
GSPATT00002284001	0,447	0,0176	0	GSPATT00021423001	0,41	0,01616	0
GSPATT00002285001	0,846	0,02305	0	GSPATT00021473001	-0,067	0,62289	0
GSPATT00002295001	0,216	0,24021	0	GSPATT00021496001	-0,121	0,56931	0
GSPATT00002297001	0,322	0,10354	0	GSPATT00021511001	0,607	0,01408	0
GSPATT00002321001	0,196	0,2005	0	GSPATT00021522001	0,222	0,20683	0
GSPATT00002371001	1,279	0,00502	1	GSPATT00021531001	1,595	0	1
GSPATT00002396001	0,89	0,00446	0	GSPATT00021532001	0,469	0,01357	0
GSPATT00002397001	0,65	0,00105	0	GSPATT00021547001	0,33	0,05213	0

GSPATT00002398001	0,608	0,00382	0	GSPATT00021550001	0,69	0,00115	0
GSPATT00002410001	0,268	0,0869	0	GSPATT00021566001	0,15	0,38869	0
GSPATT00002422001	0,51	0,00603	0	GSPATT00021592001	0,574	0,00334	0
GSPATT00002424001	-0,521	0,01826	0	GSPATT00021605001	-0,285	0,07981	0
GSPATT00002429001	0,235	0,11218	0	GSPATT00021623001	-0,006	0,97564	0
GSPATT00002438001	0,165	0,29594	0	GSPATT00021625001	-0,609	0,00663	0
GSPATT00002465001	0,13	0,62332	0	GSPATT00021626001	0,481	0,08266	0
GSPATT00002480001	1,183	0,00004	1	GSPATT00021637001	0,858	0,0012	0
GSPATT00002495001	0,174	0,40222	0	GSPATT00021639001	1,696	0,00085	1
GSPATT00002529001	1,935	0	1	GSPATT00021643001	0,057	0,70009	0
GSPATT00002550001	-0,042	0,75069	0	GSPATT00021645001	0,312	0,06296	0
GSPATT00002553001	0,322	0,07357	0	GSPATT00021666001	-0,927	0,0001	0
GSPATT00002557001	0,873	0,00059	0	GSPATT00021705001	0,593	0,01376	0
GSPATT00002575001	0,758	0,01395	0	GSPATT00021709001	1,146	0,00013	1
GSPATT00002585001	0,436	0,08223	0	GSPATT00021737001	2,607	0,00001	1
GSPATT00002589001	0,394	0,03041	0	GSPATT00021745001	-0,479	0,00827	0
GSPATT00002593001	1,076	0,0001	1	GSPATT00021748001	0,772	0,0086	0
GSPATT00002595001	0,321	0,0482	0	GSPATT00021749001	1,147	0,01618	1
GSPATT00002614001	-0,048	0,8212	0	GSPATT00021752001	0,314	0,06862	0
GSPATT00002618001	0,222	0,2786	0	GSPATT00021753001	0,418	0,07228	0
GSPATT00002633001	0,209	0,22321	0	GSPATT00021754001	0,243	0,14094	0
GSPATT00002634001	-0,559	0,00848	0	GSPATT00021761001	0,639	0,00204	0
GSPATT00002648001	-0,019	0,91239	0	GSPATT00021763001	1,74	0,00004	1
GSPATT00002670001	0,301	0,22471	0	GSPATT00021786001	0,144	0,35607	0
GSPATT00002680001	1,006	0,00039	1	GSPATT00021789001	1,156	0,00096	1
GSPATT00002682001	0,464	0,04413	0	GSPATT00021797001	0,664	0,0087	0
GSPATT00002705001	0,629	0,00714	0	GSPATT00021815001	0,094	0,7025	0
GSPATT00002720001	-1,253	0,00068	-1	GSPATT00021818001	0,452	0,0106	0
GSPATT00002743001	-0,488	0,03306	0	GSPATT00021839001	-0,624	0,00196	0
GSPATT00002752001	0,495	0,06805	0	GSPATT00021843001	-0,573	0,01408	0
GSPATT00002760001	0,788	0,00112	0	GSPATT00021861001	-2,393	0,00001	-1
GSPATT00002771001	0,859	0,0012	0	GSPATT00021876001	0,606	0,05246	0
GSPATT00002776001	-0,859	0,00025	0	GSPATT00021880001	0,418	0,06147	0
GSPATT00002799001	-0,188	0,27826	0	GSPATT00021882001	-0,509	0,02829	0
GSPATT00002813001	0,189	0,28818	0	GSPATT00021885001	0,508	0,01902	0
GSPATT00002822001	0,211	0,21934	0	GSPATT00021893001	1,556	0,00001	1
GSPATT00002824001	-0,251	0,09825	0	GSPATT00021905001	0,176	0,26244	0
GSPATT00002833001	-0,306	0,26468	0	GSPATT00021907001	-0,742	0,0007	0
GSPATT00002839001	-0,591	0,0576	0	GSPATT00021930001	-0,548	0,007	0
GSPATT00002846001	-0,271	0,31085	0	GSPATT00021935001	0,444	0,0187	0
GSPATT00002865001	-0,375	0,08216	0	GSPATT00021944001	-1,181	0,00069	-1
GSPATT00002890001	-1,023	0,00005	-1	GSPATT00021946001	0,249	0,20733	0
GSPATT00002908001	1,161	0,00015	1	GSPATT00021955001	1,15	0,00027	1
GSPATT00002917001	-0,703	0,00355	0	GSPATT00021964001	0,822	0,01015	0
GSPATT00002933001	-2,484	0	-1	GSPATT00021971001	-0,255	0,12333	0
GSPATT00002937001	-0,313	0,07411	0	GSPATT00021978001	-0,527	0,00915	0
GSPATT00002954001	-0,433	0,0165	0	GSPATT00021986001	1,819	0,00001	1
GSPATT00002960001	-0,423	0,06926	0	GSPATT00022016001	0,205	0,15716	0
GSPATT00002969001	-0,585	0,02587	0	GSPATT00022037001	0,79	0,00181	0
GSPATT00002999001	1,266	0,00041	1	GSPATT00022042001	0,34	0,05228	0
GSPATT00003022001	0,072	0,67211	0	GSPATT00022061001	-0,298	0,11655	0
GSPATT00003037001	0,383	0,02776	0	GSPATT00022128001	-0,88	0,0016	0
GSPATT00003054001	0,076	0,56297	0	GSPATT00022131001	-0,083	0,72879	0
GSPATT00003075001	0,629	0,02375	0	GSPATT00022141001	1,027	0,00014	1
GSPATT00003077001	-0,196	0,20764	0	GSPATT00022151001	-0,741	0,00371	0
GSPATT00003089001	-0,441	0,11477	0	GSPATT00022154001	0,346	0,07096	0
GSPATT00003090001	-0,149	0,32277	0	GSPATT00022155001	0,511	0,00951	0
GSPATT00003094001	0,49	0,01226	0	GSPATT00022167001	0,329	0,07157	0
GSPATT00003105001	1,596	0,00013	1	GSPATT00022172001	0,608	0,01725	0
GSPATT00003123001	0,138	0,52706	0	GSPATT00022183001	-0,244	0,23912	0
GSPATT00003132001	0,679	0,00156	0	GSPATT00022205001	0,366	0,11152	0
GSPATT00003154001	0,332	0,08672	0	GSPATT00022214001	0,659	0,00671	0
GSPATT00003156001	0,696	0,00226	0	GSPATT00022218001	-0,466	0,01328	0
GSPATT00003167001	0,084	0,63843	0	GSPATT00022236001	1,845	0,00007	1
GSPATT00003168001	1,73	0,00037	1	GSPATT00022240001	0,698	0,0019	0
GSPATT00003185001	0,267	0,31747	0	GSPATT00022249001	0,454	0,00814	0
GSPATT00003191001	-0,442	0,0623	0	GSPATT00022251001	-0,514	0,08066	0

GSPATT00003192001	-0,143	0,32291	0	GSPATT00022256001	0,89	0,0088	0
GSPATT00003194001	1,519	0,00002	1	GSPATT00022278001	0,113	0,6617	0
GSPATT00003198001	0,506	0,02601	0	GSPATT00022290001	1,407	0,00034	1
GSPATT00003200001	0,421	0,03094	0	GSPATT00022296001	1,798	0,00001	1
GSPATT00003205001	-0,564	0,00697	0	GSPATT00022304001	0,901	0,00027	0
GSPATT00003224001	-0,237	0,12454	0	GSPATT00022316001	0,995	0,0013	0
GSPATT00003229001	0,103	0,49036	0	GSPATT00022319001	-0,149	0,47401	0
GSPATT00003230001	0,337	0,26038	0	GSPATT00022364001	0,012	0,94018	0
GSPATT00003236001	1,111	0,00005	1	GSPATT00022373001	0,778	0,00583	0
GSPATT00003248001	-0,663	0,0009	0	GSPATT00022375001	0,335	0,05369	0
GSPATT00003253001	0,103	0,43872	0	GSPATT00022376001	0,282	0,36231	0
GSPATT00003279001	0,867	0,0006	0	GSPATT00022389001	0,267	0,0861	0
GSPATT00003280001	-0,04	0,80148	0	GSPATT00022398001	-0,303	0,11489	0
GSPATT00003283001	0,264	0,25078	0	GSPATT00022403001	0,733	0,00613	0
GSPATT00003292001	0,915	0,00467	0	GSPATT00022415001	0,64	0,00116	0
GSPATT00003309001	-0,973	0,0002	0	GSPATT00022426001	0,639	0,00648	0
GSPATT00003326001	-0,051	0,69934	0	GSPATT00022436001	-0,065	0,75967	0
GSPATT00003335001	-0,087	0,60586	0	GSPATT00022478001	-0,264	0,12121	0
GSPATT00003339001	0,266	0,15758	0	GSPATT00022479001	-0,83	0,0106	0
GSPATT00003344001	0,549	0,00644	0	GSPATT00022490001	0,953	0,00048	0
GSPATT00003348001	-0,128	0,5085	0	GSPATT00022560001	0,407	0,01304	0
GSPATT00003366001	0,829	0,00229	0	GSPATT00022581001	0,197	0,34705	0
GSPATT00003371001	0,268	0,11218	0	GSPATT00022585001	-0,137	0,38681	0
GSPATT00003407001	0,787	0,00042	0	GSPATT00022588001	0,519	0,01581	0
GSPATT00003425001	-0,517	0,01811	0	GSPATT00022625001	-0,631	0,00225	0
GSPATT00003444001	0,165	0,26508	0	GSPATT00022648001	1,169	0,00006	1
GSPATT00003486001	0,113	0,47243	0	GSPATT00022652001	0,216	0,20385	0
GSPATT00003501001	0,158	0,34108	0	GSPATT00022660001	0,792	0,00748	0
GSPATT00003507001	-1,565	0,00001	-1	GSPATT00022665001	0,156	0,24856	0
GSPATT00003528001	0,505	0,01812	0	GSPATT00022666001	1,059	0,00175	1
GSPATT00003535001	0,273	0,06967	0	GSPATT00022671001	0,453	0,06689	0
GSPATT00003572001	0,717	0,01034	0	GSPATT00022672001	0,392	0,0156	0
GSPATT00003620001	0,88	0,00103	0	GSPATT00022676001	0,956	0,00118	0
GSPATT00003647001	0,327	0,21564	0	GSPATT00022680001	0,88	0,01013	0
GSPATT00003651001	0,62	0,00494	0	GSPATT00022705001	0,511	0,00652	0
GSPATT00003657001	-0,746	0,00329	0	GSPATT00022722001	-0,149	0,46764	0
GSPATT00003674001	0,666	0,18862	0	GSPATT00022744001	0,355	0,09992	0
GSPATT00003681001	-0,103	0,4827	0	GSPATT00022747001	-0,042	0,84234	0
GSPATT00003682001	1,134	0,00047	1	GSPATT00022758001	-0,264	0,37366	0
GSPATT00003692001	0,249	0,08305	0	GSPATT00022760001	-0,332	0,06229	0
GSPATT00003699001	-0,208	0,19088	0	GSPATT00022772001	0,587	0,00225	0
GSPATT00003701001	-1,324	0,00003	-1	GSPATT00022794001	-0,642	0,00449	0
GSPATT00003711001	-0,057	0,78591	0	GSPATT00022804001	0,571	0,02711	0
GSPATT00003719001	-2,965	0	-1	GSPATT00022805001	0,721	0,00384	0
GSPATT00003722001	-0,214	0,14285	0	GSPATT00022813001	-0,287	0,25917	0
GSPATT00003724001	-1,06	0,00023	-1	GSPATT00022862001	0,196	0,23874	0
GSPATT00003735001	0,938	0,00036	0	GSPATT00022923001	0,087	0,54996	0
GSPATT00003784001	-0,762	0,00155	0	GSPATT00022960001	-0,158	0,24845	0
GSPATT00003800001	0,707	0,00325	0	GSPATT00022982001	0,79	0,04014	0
GSPATT00003805001	-0,458	0,01837	0	GSPATT00022998001	0,587	0,0075	0
GSPATT00003808001	-1,655	0,00001	-1	GSPATT00023007001	0,391	0,03401	0
GSPATT00003833001	0,314	0,13153	0	GSPATT00023011001	0,775	0,00132	0
GSPATT00003834001	0,118	0,39846	0	GSPATT00023034001	-0,277	0,07151	0
GSPATT00003866001	0,17	0,22326	0	GSPATT00023053001	0,174	0,19072	0
GSPATT00003883001	1,07	0,00005	1	GSPATT00023054001	0,175	0,21904	0
GSPATT00003889001	1,021	0,00011	1	GSPATT00023076001	0,772	0,00846	0
GSPATT00003892001	-0,172	0,33783	0	GSPATT00023086001	0,21	0,20719	0
GSPATT00003937001	-0,141	0,58292	0	GSPATT00023095001	-0,105	0,42062	0
GSPATT00003941001	-0,015	0,90753	0	GSPATT00023101001	0,252	0,21939	0
GSPATT00003946001	0,029	0,8323	0	GSPATT00023116001	0,243	0,2874	0
GSPATT00003963001	0,533	0,01029	0	GSPATT00023168001	-0,346	0,10457	0
GSPATT00003987001	0,889	0,00018	0	GSPATT00023209001	0,505	0,02572	0
GSPATT00004011001	0,459	0,0181	0	GSPATT00023226001	-0,09	0,6793	0
GSPATT00004041001	-2,104	0,00001	-1	GSPATT00023238001	-0,52	0,00932	0
GSPATT00004054001	0,449	0,01063	0	GSPATT00023249001	0,903	0,00029	0
GSPATT00004057001	-0,473	0,03309	0	GSPATT00023269001	-0,248	0,34866	0
GSPATT00004059001	0,026	0,88876	0	GSPATT00023282001	-0,358	0,04566	0

GSPATT00004060001	0,495	0,02651	0	GSPATT00023287001	-0,508	0,01292	0
GSPATT00004091001	0,299	0,07171	0	GSPATT00023291001	-0,368	0,10871	0
GSPATT00004095001	-0,095	0,61368	0	GSPATT00023309001	-0,41	0,01263	0
GSPATT00004114001	0,269	0,09228	0	GSPATT00023311001	0,38	0,03477	0
GSPATT00004132001	-0,103	0,69251	0	GSPATT00023324001	0,902	0,00067	0
GSPATT00004152001	0,206	0,29595	0	GSPATT00023325001	0,851	0,00031	0
GSPATT00004156001	0,259	0,28686	0	GSPATT00023332001	0,059	0,7739	0
GSPATT00004164001	0,47	0,0111	0	GSPATT00023391001	0,729	0,00128	0
GSPATT00004205001	-0,31	0,17947	0	GSPATT00023409001	-0,413	0,17182	0
GSPATT00004210001	0,416	0,03288	0	GSPATT00023417001	0,386	0,08109	0
GSPATT00004223001	0,612	0,00738	0	GSPATT00023422001	-0,559	0,02664	0
GSPATT00004225001	0,089	0,74057	0	GSPATT00023426001	-0,016	0,92617	0
GSPATT00004229001	-0,224	0,29312	0	GSPATT00023427001	0,645	0,027	0
GSPATT00004247001	0,159	0,57122	0	GSPATT00023429001	0,253	0,18096	0
GSPATT00004268001	1,307	0,00011	1	GSPATT00023436001	0,755	0,04632	0
GSPATT00004273001	-0,717	0,00174	0	GSPATT00023438001	0,035	0,83414	0
GSPATT00004274001	-1,143	0,00023	-1	GSPATT00023469001	-0,011	0,95408	0
GSPATT00004298001	0,13	0,50582	0	GSPATT00023488001	1,084	0,00116	1
GSPATT00004311001	0,568	0,00775	0	GSPATT00023514001	0,507	0,00757	0
GSPATT00004321001	0,151	0,6363	0	GSPATT00023531001	0,22	0,46511	0
GSPATT00004366001	0,068	0,69186	0	GSPATT00023540001	0,273	0,10052	0
GSPATT00004368001	0,323	0,16554	0	GSPATT00023549001	-1,224	0,00167	-1
GSPATT00004374001	1,795	0	1	GSPATT00023569001	-0,979	0,00048	0
GSPATT00004376001	0,343	0,08104	0	GSPATT00023571001	-0,22	0,33947	0
GSPATT00004396001	0,315	0,11278	0	GSPATT00023576001	-0,089	0,51115	0
GSPATT00004405001	0,153	0,39785	0	GSPATT00023605001	-0,146	0,31583	0
GSPATT00004412001	-1,59	0,00001	-1	GSPATT00023613001	0,192	0,1796	0
GSPATT00004440001	-0,239	0,16013	0	GSPATT00023618001	-0,03	0,8305	0
GSPATT00004442001	-0,201	0,48124	0	GSPATT00023627001	0,203	0,23093	0
GSPATT00004447001	0,383	0,05161	0	GSPATT00023631001	0,239	0,10083	0
GSPATT00004464001	-0,315	0,08048	0	GSPATT00023632001	0,122	0,36366	0
GSPATT00004480001	0,37	0,07342	0	GSPATT00023650001	0,252	0,19222	0
GSPATT00004496001	0,955	0,00187	0	GSPATT00023676001	0,145	0,31782	0
GSPATT00004514001	0,476	0,02673	0	GSPATT00023690001	-1,078	0,0043	-1
GSPATT00004528001	0,66	0,00397	0	GSPATT00023719001	-0,472	0,04584	0
GSPATT00004559001	0,711	0,00505	0	GSPATT00023728001	0,102	0,45091	0
GSPATT00004570001	0,195	0,22266	0	GSPATT00023729001	0,5	0,071	0
GSPATT00004575001	-0,004	0,97788	0	GSPATT00023735001	0,573	0,00261	0
GSPATT00004580001	1,242	0,00014	1	GSPATT00023790001	-0,309	0,06093	0
GSPATT00004590001	0,499	0,01576	0	GSPATT00023792001	0,035	0,82314	0
GSPATT00004592001	-0,308	0,04588	0	GSPATT00023807001	-0,378	0,04035	0
GSPATT00004598001	0,48	0,02633	0	GSPATT00023823001	-0,725	0,00571	0
GSPATT00004600001	0,083	0,57831	0	GSPATT00023843001	0,248	0,16073	0
GSPATT00004601001	1,962	0,0001	1	GSPATT00023861001	1,352	0,00004	1
GSPATT00004610001	0,945	0,00014	0	GSPATT00023871001	-0,181	0,31269	0
GSPATT00004630001	-0,933	0,00013	0	GSPATT00023901001	0,661	0,00244	0
GSPATT00004644001	-2,245	0,00001	-1	GSPATT00023907001	0,749	0,00073	0
GSPATT00004648001	0,147	0,30806	0	GSPATT00023914001	-0,777	0,0023	0
GSPATT00004661001	-0,088	0,65447	0	GSPATT00023937001	-0,496	0,07124	0
GSPATT00004664001	0,401	0,16244	0	GSPATT00023947001	0,21	0,19097	0
GSPATT00004681001	-0,284	0,11177	0	GSPATT00023951001	0,462	0,01336	0
GSPATT00004693001	0,654	0,00623	0	GSPATT00023962001	-0,49	0,01746	0
GSPATT00004708001	-0,936	0,00106	0	GSPATT00023981001	0,056	0,75794	0
GSPATT00004713001	-0,302	0,08002	0	GSPATT00024010001	0,28	0,11337	0
GSPATT00004716001	-0,265	0,1283	0	GSPATT00024035001	0,75	0,00345	0
GSPATT00004725001	-0,044	0,79332	0	GSPATT00024046001	0,488	0,06203	0
GSPATT00004760001	0,235	0,3263	0	GSPATT00024078001	0,879	0,0027	0
GSPATT00004761001	0,808	0,01968	0	GSPATT00024080001	0,962	0,00084	0
GSPATT00004764001	-0,15	0,28228	0	GSPATT00024093001	0,589	0,00369	0
GSPATT00004777001	1,749	0,00006	1	GSPATT00024120001	0,414	0,05081	0
GSPATT00004785001	0,782	0,00521	0	GSPATT00024158001	0,676	0,00939	0
GSPATT00004807001	0,124	0,60039	0	GSPATT00024181001	0,193	0,36401	0
GSPATT00004813001	0,679	0,01555	0	GSPATT00024182001	-0,819	0,01549	0
GSPATT00004832001	-0,277	0,07921	0	GSPATT00024183001	-0,176	0,34939	0
GSPATT00004834001	0,789	0,00098	0	GSPATT00024185001	1,039	0,00005	1
GSPATT00004846001	2,635	0	1	GSPATT00024203001	0,349	0,11648	0
GSPATT00004860001	-0,019	0,91882	0	GSPATT00024205001	0,66	0,00203	0

GSPATT00004865001	-0,645	0,01547	0	GSPATT00024206001	-0,177	0,2138	0
GSPATT00004869001	0,682	0,00693	0	GSPATT00024209001	1,188	0,00003	1
GSPATT00004874001	-0,141	0,55683	0	GSPATT00024218001	-0,321	0,04683	0
GSPATT00004905001	0,24	0,28818	0	GSPATT00024233001	0,949	0,0003	0
GSPATT00004906001	0,041	0,80854	0	GSPATT00024234001	0,863	0,00016	0
GSPATT00004925001	-0,765	0,01316	0	GSPATT00024244001	0,136	0,47521	0
GSPATT00004954001	-0,94	0,02171	0	GSPATT00024253001	0,327	0,12765	0
GSPATT00004968001	0,709	0,00121	0	GSPATT00024274001	0,022	0,87189	0
GSPATT00004976001	-0,023	0,86985	0	GSPATT00024309001	-0,157	0,28831	0
GSPATT00004988001	-0,207	0,1401	0	GSPATT00024335001	-0,34	0,04551	0
GSPATT00004998001	0,877	0,0004	0	GSPATT00024337001	0,437	0,02363	0
GSPATT00005003001	0,289	0,06439	0	GSPATT00024348001	-0,075	0,56089	0
GSPATT00005007001	0,56	0,00815	0	GSPATT00024349001	0,096	0,597	0
GSPATT00005009001	0,54	0,00742	0	GSPATT00024352001	0,614	0,00859	0
GSPATT00005029001	0,213	0,14145	0	GSPATT00024357001	0,148	0,45854	0
GSPATT00005077001	0,271	0,1489	0	GSPATT00024377001	1,043	0,00342	1
GSPATT00005093001	0,879	0,00087	0	GSPATT00024390001	-0,282	0,13976	0
GSPATT00005095001	0,782	0,00596	0	GSPATT00024398001	0,796	0,01758	0
GSPATT00005098001	0,976	0,00298	0	GSPATT00024407001	-0,816	0,004	0
GSPATT00005100001	-0,27	0,06839	0	GSPATT00024410001	0,428	0,02489	0
GSPATT00005117001	0,136	0,40124	0	GSPATT00024418001	0,221	0,13987	0
GSPATT00005120001	0,8	0,00223	0	GSPATT00024424001	-0,29	0,0846	0
GSPATT00005130001	-0,006	0,97738	0	GSPATT00024429001	0,278	0,07692	0
GSPATT00005209001	1,585	0,00011	1	GSPATT00024448001	-0,278	0,17393	0
GSPATT00005214001	-0,055	0,72902	0	GSPATT00024450001	-0,469	0,00704	0
GSPATT00005235001	-0,322	0,0632	0	GSPATT00024451001	0,393	0,03105	0
GSPATT00005243001	1,145	0,0018	1	GSPATT00024474001	-0,231	0,34278	0
GSPATT00005245001	0,407	0,07978	0	GSPATT00024479001	-0,903	0,00303	0
GSPATT00005247001	-0,102	0,43462	0	GSPATT00024481001	0,435	0,1006	0
GSPATT00005248001	-1,692	0,00037	-1	GSPATT00024485001	-0,462	0,15535	0
GSPATT00005267001	1,049	0,00042	1	GSPATT00024498001	0,272	0,35558	0
GSPATT00005269001	0,265	0,06576	0	GSPATT00024499001	0,56	0,00549	0
GSPATT00005271001	-0,185	0,35608	0	GSPATT00024504001	-0,557	0,06748	0
GSPATT00005274001	0,558	0,00601	0	GSPATT00024512001	1,298	0,00001	1
GSPATT00005284001	0,206	0,14532	0	GSPATT00024514001	0,748	0,01065	0
GSPATT00005295001	0,7	0,00631	0	GSPATT00024536001	0,01	0,96698	0
GSPATT00005301001	0,612	0,02995	0	GSPATT00024539001	-2,028	0	-1
GSPATT00005326001	1,016	0,00028	1	GSPATT00024546001	-0,401	0,04144	0
GSPATT00005329001	0,523	0,01314	0	GSPATT00024564001	0,433	0,05933	0
GSPATT00005343001	-0,049	0,73244	0	GSPATT00024565001	1,115	0,00008	1
GSPATT00005359001	-0,071	0,70866	0	GSPATT00024566001	0,629	0,00565	0
GSPATT00005382001	0,315	0,08827	0	GSPATT00024583001	0,315	0,15638	0
GSPATT00005394001	-2,476	0	-1	GSPATT00024591001	0,498	0,00429	0
GSPATT00005416001	-0,283	0,0906	0	GSPATT00024598001	-0,675	0,01914	0
GSPATT00005424001	-2,387	0	-1	GSPATT00024618001	0,097	0,49798	0
GSPATT00005443001	1,201	0,01436	1	GSPATT00024644001	0,654	0,00896	0
GSPATT00005458001	-0,786	0,00076	0	GSPATT00024673001	-0,186	0,23745	0
GSPATT00005479001	0,453	0,01604	0	GSPATT00024691001	1,052	0,00127	1
GSPATT00005498001	0,586	0,00534	0	GSPATT00024731001	0,285	0,24363	0
GSPATT00005504001	-0,323	0,07461	0	GSPATT00024737001	0,421	0,14567	0
GSPATT00005531001	0,024	0,88617	0	GSPATT00024766001	0,352	0,11206	0
GSPATT00005533001	-0,809	0,00171	0	GSPATT00024788001	0,153	0,34111	0
GSPATT00005536001	0,887	0,00254	0	GSPATT00024845001	0,312	0,05216	0
GSPATT00005555001	0,146	0,36762	0	GSPATT00024859001	1,158	0,00272	1
GSPATT00005556001	0,344	0,07104	0	GSPATT00024862001	0,232	0,28545	0
GSPATT00005557001	0,873	0,00334	0	GSPATT00024885001	-1,562	0,00004	-1
GSPATT00005568001	0,44	0,03873	0	GSPATT00024889001	0,107	0,58905	0
GSPATT00005581001	0,156	0,32591	0	GSPATT00024895001	-0,166	0,43815	0
GSPATT00005587001	0,343	0,06896	0	GSPATT00024913001	-0,542	0,01406	0
GSPATT00005611001	0,102	0,61679	0	GSPATT00024916001	1,07	0,00006	1
GSPATT00005647001	-0,353	0,0216	0	GSPATT00024917001	1,027	0,0002	1
GSPATT00005692001	-0,035	0,79461	0	GSPATT00024942001	-0,918	0,00043	0
GSPATT00005703001	0,426	0,05301	0	GSPATT00024976001	0,84	0,00119	0
GSPATT00005726001	0,372	0,0779	0	GSPATT00024990001	0,807	0,0027	0
GSPATT00005733001	0,017	0,92434	0	GSPATT00025030001	-0,48	0,01979	0
GSPATT00005734001	0,285	0,09036	0	GSPATT00025035001	1,087	0,00028	1
GSPATT00005738001	0,978	0,00023	0	GSPATT00025044001	0,229	0,26111	0

GSPATT00005752001	1,537	0,00004	1	GSPATT00025071001	-0,063	0,7592	0
GSPATT00005757001	0,742	0,00204	0	GSPATT00025076001	0,189	0,30656	0
GSPATT00005760001	-0,591	0,07206	0	GSPATT00025088001	0,087	0,7199	0
GSPATT00005782001	0,537	0,00527	0	GSPATT00025089001	0,396	0,07801	0
GSPATT00005804001	-2,483	0,00045	-1	GSPATT00025098001	0,473	0,02724	0
GSPATT00005811001	0,498	0,03959	0	GSPATT00025106001	0,702	0,01194	0
GSPATT00005814001	-0,041	0,82667	0	GSPATT00025128001	0,313	0,11606	0
GSPATT00005845001	0,153	0,32908	0	GSPATT00025133001	0,871	0,00255	0
GSPATT00005861001	0,66	0,00103	0	GSPATT00025144001	1,216	0,00018	1
GSPATT00005868001	0,142	0,49582	0	GSPATT00025176001	0,858	0,01028	0
GSPATT00005881001	0,784	0,01296	0	GSPATT00025177001	0,141	0,61521	0
GSPATT00005902001	1,463	0,00021	1	GSPATT00025184001	0,545	0,03088	0
GSPATT00005904001	0,174	0,28531	0	GSPATT00025243001	1,014	0,00008	1
GSPATT00005911001	0,788	0,00064	0	GSPATT00025254001	0,909	0,00035	0
GSPATT00005943001	1,376	0,00047	1	GSPATT00025257001	0,582	0,02537	0
GSPATT00005960001	1,544	0,00007	1	GSPATT00025262001	-0,617	0,0118	0
GSPATT00005995001	0,249	0,17873	0	GSPATT00025273001	1,277	0,00074	1
GSPATT00006007001	0,509	0,01301	0	GSPATT00025287001	-2,647	0	-1
GSPATT00006011001	0,426	0,04714	0	GSPATT00025348001	0,917	0,00082	0
GSPATT00006016001	0,78	0,00524	0	GSPATT00025382001	-0,664	0,00239	0
GSPATT00006030001	0,381	0,05068	0	GSPATT00025431001	0,45	0,04878	0
GSPATT00006033001	0,654	0,0075	0	GSPATT00025443001	0,397	0,05816	0
GSPATT00006057001	-0,917	0,00035	0	GSPATT00025447001	0,033	0,85164	0
GSPATT00006063001	-0,097	0,55356	0	GSPATT00025450001	0,01	0,9566	0
GSPATT00006069001	0,895	0,00022	0	GSPATT00025459001	0,443	0,0356	0
GSPATT00006075001	0,152	0,28079	0	GSPATT00025471001	-1,355	0,00017	-1
GSPATT00006080001	0,359	0,11534	0	GSPATT00025472001	0,638	0,01873	0
GSPATT00006094001	0,938	0,00214	0	GSPATT00025473001	1,303	0,00076	1
GSPATT00006125001	0,447	0,0539	0	GSPATT00025476001	1,287	0,00012	1
GSPATT00006174001	-1,189	0,00097	-1	GSPATT00025481001	-0,394	0,02039	0
GSPATT00006199001	-0,122	0,43015	0	GSPATT00025487001	0,527	0,00492	0
GSPATT00006217001	0,043	0,84452	0	GSPATT00025488001	0,117	0,44687	0
GSPATT00006226001	-0,062	0,65778	0	GSPATT00025542001	0,154	0,32238	0
GSPATT00006253001	-0,435	0,0265	0	GSPATT00025564001	0,335	0,03391	0
GSPATT00006272001	0,621	0,03899	0	GSPATT00025565001	0,102	0,44835	0
GSPATT00006280001	0,726	0,00083	0	GSPATT00025585001	0,189	0,32528	0
GSPATT00006296001	0,129	0,52836	0	GSPATT00025594001	-3,032	0	-1
GSPATT00006297001	0,413	0,01659	0	GSPATT00025621001	0,549	0,02246	0
GSPATT00006313001	0,809	0,01243	0	GSPATT00025624001	1,872	0,00011	1
GSPATT00006314001	0,607	0,00237	0	GSPATT00025626001	0,186	0,22873	0
GSPATT00006315001	0,211	0,21775	0	GSPATT00025634001	-0,572	0,0037	0
GSPATT00006331001	0,84	0,00169	0	GSPATT00025641001	-0,136	0,41303	0
GSPATT00006334001	-0,044	0,80576	0	GSPATT00025658001	0,311	0,08053	0
GSPATT00006378001	0,677	0,00317	0	GSPATT00025660001	0,423	0,01588	0
GSPATT00006379001	0,429	0,03699	0	GSPATT00025674001	-0,467	0,00665	0
GSPATT00006390001	-3,443	0	-1	GSPATT00025677001	0,628	0,0033	0
GSPATT00006408001	-0,255	0,28163	0	GSPATT00025683001	1,103	0,00903	1
GSPATT00006415001	-0,218	0,29617	0	GSPATT00025688001	0,986	0,00101	0
GSPATT00006446001	-0,06	0,78799	0	GSPATT00025714001	-0,285	0,11387	0
GSPATT00006468001	1,644	0,00007	1	GSPATT00025724001	-0,118	0,54796	0
GSPATT00006472001	-0,821	0,00056	0	GSPATT00025742001	0,095	0,63776	0
GSPATT00006519001	1,503	0,00026	1	GSPATT00025754001	1,693	0	1
GSPATT00006522001	0,067	0,61368	0	GSPATT00025774001	-3,012	0,00001	-1
GSPATT00006526001	0,784	0,00476	0	GSPATT00025790001	0,722	0,01137	0
GSPATT00006544001	0,253	0,22418	0	GSPATT00025829001	-0,303	0,13502	0
GSPATT00006575001	-0,075	0,7515	0	GSPATT00025830001	-0,219	0,47777	0
GSPATT00006576001	0,119	0,43904	0	GSPATT00025833001	0,318	0,12793	0
GSPATT00006580001	0,511	0,15256	0	GSPATT00025835001	0,577	0,00646	0
GSPATT00006581001	-0,025	0,86132	0	GSPATT00025843001	-0,234	0,1386	0
GSPATT00006592001	0,443	0,14222	0	GSPATT00025874001	0,078	0,61912	0
GSPATT00006594001	2,357	0	1	GSPATT00025891001	0,036	0,78724	0
GSPATT00006615001	0,212	0,32587	0	GSPATT00025892001	0,813	0,00112	0
GSPATT00006642001	1,159	0,00005	1	GSPATT00025893001	0,184	0,26595	0
GSPATT00006653001	0,808	0,00058	0	GSPATT00025925001	-2,389	0,00001	-1
GSPATT00006659001	-0,504	0,0224	0	GSPATT00025938001	0,244	0,21258	0
GSPATT00006666001	-0,748	0,0059	0	GSPATT00025977001	0,266	0,20896	0
GSPATT00006681001	1,225	0,00002	1	GSPATT00025982001	0,105	0,52355	0

GSPATT00006690001	0,625	0,00374	0	GSPATT00025983001	0,713	0,0069	0
GSPATT00006702001	1,003	0,00049	1	GSPATT00025987001	0,777	0,00304	0
GSPATT00006707001	0,096	0,55283	0	GSPATT00025999001	0,214	0,18023	0
GSPATT00006718001	-1,206	0,0008	-1	GSPATT00026028001	-0,597	0,02174	0
GSPATT00006721001	0,259	0,28365	0	GSPATT00026044001	1,043	0,00011	1
GSPATT00006725001	1,26	0,00008	1	GSPATT00026048001	0,131	0,45105	0
GSPATT00006735001	1,208	0,00017	1	GSPATT00026052001	0,22	0,26869	0
GSPATT00006746001	0,845	0,00197	0	GSPATT00026100001	-0,727	0,05157	0
GSPATT00006769001	0,213	0,32339	0	GSPATT00026117001	0,711	0,00252	0
GSPATT00006775001	0,248	0,09259	0	GSPATT00026130001	0,041	0,84926	0
GSPATT00006782001	0,148	0,27067	0	GSPATT00026158001	-0,019	0,89138	0
GSPATT00006852001	0,834	0,00426	0	GSPATT00026165001	1,329	0,00001	1
GSPATT00006853001	0,531	0,04301	0	GSPATT00026166001	0,972	0,00364	0
GSPATT00006854001	0,961	0,00034	0	GSPATT00026167001	0,538	0,00916	0
GSPATT00006864001	0,6	0,00924	0	GSPATT00026169001	0,593	0,0211	0
GSPATT00006875001	0,375	0,03661	0	GSPATT00026182001	0,405	0,01463	0
GSPATT00006884001	0,278	0,14598	0	GSPATT00026208001	-0,305	0,05561	0
GSPATT00006904001	0,29	0,11816	0	GSPATT00026214001	0,126	0,51347	0
GSPATT00006927001	0,501	0,00669	0	GSPATT00026231001	-0,721	0,00236	0
GSPATT00006928001	0,334	0,0274	0	GSPATT00026239001	0,204	0,24495	0
GSPATT00006948001	0,353	0,03894	0	GSPATT00026269001	-0,827	0,00039	0
GSPATT00006949001	0,568	0,03228	0	GSPATT00026274001	0,081	0,55749	0
GSPATT00006953001	0,189	0,35123	0	GSPATT00026292001	0,175	0,24434	0
GSPATT00006973001	-1,451	0,00032	-1	GSPATT00026323001	-0,937	0,00078	0
GSPATT00006974001	0,738	0,04036	0	GSPATT00026336001	-0,538	0,01977	0
GSPATT00006977001	-2,575	0	-1	GSPATT00026341001	0,56	0,00614	0
GSPATT00007015001	-0,04	0,8039	0	GSPATT00026346001	-0,833	0,00425	0
GSPATT00007020001	0,699	0,0179	0	GSPATT00026360001	1,582	0,00001	1
GSPATT00007023001	0,031	0,83742	0	GSPATT00026362001	0,039	0,7821	0
GSPATT00007028001	1,888	0,00035	1	GSPATT00026366001	0,255	0,08585	0
GSPATT00007036001	-1,628	0,00205	-1	GSPATT00026371001	0,968	0,00219	0
GSPATT00007037001	0,21	0,21843	0	GSPATT00026382001	-0,122	0,48679	0
GSPATT00007044001	-0,041	0,75732	0	GSPATT00026384001	0,13	0,49373	0
GSPATT00007068001	-0,215	0,22845	0	GSPATT00026401001	0,404	0,01576	0
GSPATT00007081001	-0,453	0,09098	0	GSPATT00026410001	-1,354	0,00004	-1
GSPATT00007084001	1,343	0,0001	1	GSPATT00026424001	-0,195	0,41235	0
GSPATT00007085001	0,898	0,00027	0	GSPATT00026435001	0,978	0,0003	0
GSPATT00007086001	-0,397	0,12088	0	GSPATT00026456001	-0,112	0,49348	0
GSPATT00007091001	1,465	0,00001	1	GSPATT00026471001	0,689	0,0725	0
GSPATT00007092001	0,27	0,19194	0	GSPATT00026484001	0,101	0,56577	0
GSPATT00007095001	0,892	0,00182	0	GSPATT00026498001	0,273	0,11056	0
GSPATT00007110001	0,57	0,00537	0	GSPATT00026501001	0,573	0,02563	0
GSPATT00007113001	0,035	0,79386	0	GSPATT00026505001	-2,575	0	-1
GSPATT00007130001	0,976	0,0003	0	GSPATT00026528001	0,335	0,1504	0
GSPATT00007151001	0,29	0,08359	0	GSPATT00026538001	0,133	0,33705	0
GSPATT00007162001	-0,046	0,76877	0	GSPATT00026558001	-0,121	0,43484	0
GSPATT00007168001	2,307	0	1	GSPATT00026582001	-0,61	0,00172	0
GSPATT00007170001	0,256	0,39221	0	GSPATT00026597001	-0,036	0,87291	0
GSPATT00007176001	0,545	0,0517	0	GSPATT00026598001	-0,409	0,02421	0
GSPATT00007193001	-0,121	0,39214	0	GSPATT00026607001	0,518	0,00768	0
GSPATT00007196001	0,471	0,05051	0	GSPATT00026611001	0,416	0,09592	0
GSPATT00007204001	0,658	0,0038	0	GSPATT00026620001	-0,023	0,89953	0
GSPATT00007206001	-0,243	0,15376	0	GSPATT00026642001	0,375	0,12827	0
GSPATT00007221001	-0,047	0,73213	0	GSPATT00026656001	0,014	0,92729	0
GSPATT00007228001	-0,424	0,07621	0	GSPATT00026672001	0,656	0,00939	0
GSPATT00007288001	-0,479	0,02516	0	GSPATT00026673001	1,475	0,00074	1
GSPATT00007295001	1,711	0	1	GSPATT00026685001	-0,162	0,49003	0
GSPATT00007306001	0,194	0,49349	0	GSPATT00026711001	-1,004	0,00014	-1
GSPATT00007307001	0,539	0,00329	0	GSPATT00026728001	0,421	0,03621	0
GSPATT00007337001	0,377	0,05996	0	GSPATT00026733001	-0,183	0,2048	0
GSPATT00007341001	-0,138	0,33222	0	GSPATT00026736001	0,014	0,9335	0
GSPATT00007343001	0,329	0,28753	0	GSPATT00026737001	-0,589	0,01656	0
GSPATT00007357001	-0,154	0,51852	0	GSPATT00026742001	1,681	0,00062	1
GSPATT00007369001	-0,3	0,09662	0	GSPATT00026751001	0,217	0,23484	0
GSPATT00007385001	0,031	0,87634	0	GSPATT00026762001	0,081	0,72109	0
GSPATT00007396001	0,747	0,00516	0	GSPATT00026768001	1,3	0,0016	1
GSPATT00007424001	0,679	0,0528	0	GSPATT00026770001	0,781	0,00036	0

GSPATT00007458001	-0,178	0,41913	0	GSPATT00026784001	0,447	0,02348	0
GSPATT00007469001	0,74	0,00278	0	GSPATT00026817001	-0,512	0,01273	0
GSPATT00007491001	0,204	0,26477	0	GSPATT00026853001	-0,021	0,90542	0
GSPATT00007506001	3,323	0	1	GSPATT00026855001	-0,189	0,23205	0
GSPATT00007552001	-0,289	0,16464	0	GSPATT00026879001	0,638	0,0089	0
GSPATT00007569001	-0,059	0,72729	0	GSPATT00026882001	0,229	0,14149	0
GSPATT00007633001	0,585	0,00705	0	GSPATT00026883001	0,746	0,01254	0
GSPATT00007636001	0,951	0,00013	0	GSPATT00026903001	0,385	0,04693	0
GSPATT00007651001	1,374	0,00001	1	GSPATT00026918001	0,426	0,01186	0
GSPATT00007652001	0,604	0,00281	0	GSPATT00026931001	0,327	0,03994	0
GSPATT00007655001	1,115	0,00032	1	GSPATT00026977001	0,418	0,01985	0
GSPATT00007660001	-2,547	0	-1	GSPATT00027082001	-0,14	0,42006	0
GSPATT00007679001	-0,046	0,77776	0	GSPATT00027084001	-2,917	0	-1
GSPATT00007689001	0,756	0,0049	0	GSPATT00027094001	-0,238	0,23457	0
GSPATT00007699001	0,698	0,00069	0	GSPATT00027100001	0,499	0,06172	0
GSPATT00007733001	-0,021	0,908	0	GSPATT00027115001	-0,008	0,97395	0
GSPATT00007750001	0,245	0,21356	0	GSPATT00027119001	0,768	0,00101	0
GSPATT00007792001	0,379	0,04772	0	GSPATT00027122001	-0,502	0,01341	0
GSPATT00007794001	-1,257	0,00002	-1	GSPATT00027138001	0,18	0,19509	0
GSPATT00007797001	0,346	0,07773	0	GSPATT00027147001	-0,082	0,6483	0
GSPATT00007801001	0,358	0,05841	0	GSPATT00027149001	0,147	0,32384	0
GSPATT00007808001	0,931	0,00109	0	GSPATT00027156001	0,039	0,76929	0
GSPATT00007826001	0,485	0,01651	0	GSPATT00027189001	0,049	0,79446	0
GSPATT00007852001	0,616	0,00193	0	GSPATT00027203001	0,063	0,64969	0
GSPATT00007857001	1,41	0,00014	1	GSPATT00027205001	-0,221	0,24032	0
GSPATT00007860001	0,07	0,62078	0	GSPATT00027222001	0,074	0,59482	0
GSPATT00007861001	0,494	0,03195	0	GSPATT00027241001	0,321	0,05768	0
GSPATT00007899001	0,019	0,90526	0	GSPATT00027257001	0,806	0,00465	0
GSPATT00007911001	-0,929	0,02523	0	GSPATT00027270001	0,745	0,00887	0
GSPATT00007917001	0,096	0,67711	0	GSPATT00027312001	-1,048	0,00047	-1
GSPATT00007919001	0,035	0,87791	0	GSPATT00027324001	0,003	0,98698	0
GSPATT00007922001	0,835	0,01888	0	GSPATT00027355001	-0,308	0,10498	0
GSPATT00007930001	-0,448	0,05141	0	GSPATT00027372001	1,25	0,00004	1
GSPATT00007946001	1,164	0,00002	1	GSPATT00027401001	0,365	0,07066	0
GSPATT00007947001	0,256	0,17572	0	GSPATT00027404001	-0,378	0,02671	0
GSPATT00007949001	1,323	0,00004	1	GSPATT00027408001	0,032	0,82286	0
GSPATT00007953001	0,179	0,38295	0	GSPATT00027410001	0,523	0,0081	0
GSPATT00007957001	0,151	0,34529	0	GSPATT00027442001	-0,097	0,65217	0
GSPATT00007961001	-0,294	0,05705	0	GSPATT00027453001	-0,38	0,0322	0
GSPATT00007964001	-2,161	0,00001	-1	GSPATT00027465001	-1,097	0,00007	-1
GSPATT00007966001	0,728	0,03307	0	GSPATT00027470001	0,073	0,75047	0
GSPATT00007989001	0,044	0,84104	0	GSPATT00027479001	0,392	0,10569	0
GSPATT00007997001	0,533	0,00985	0	GSPATT00027483001	0,725	0,00101	0
GSPATT00007998001	0,219	0,19699	0	GSPATT00027492001	1,241	0,00093	1
GSPATT00008011001	0,371	0,20891	0	GSPATT00027506001	0,183	0,46769	0
GSPATT00008014001	0,284	0,11593	0	GSPATT00027520001	-0,336	0,16994	0
GSPATT00008021001	-1,112	0,00013	-1	GSPATT00027535001	0,825	0,01036	0
GSPATT00008031001	-0,45	0,02946	0	GSPATT00027546001	-0,383	0,03735	0
GSPATT00008071001	0,691	0,00225	0	GSPATT00027549001	0,322	0,09184	0
GSPATT00008074001	-0,457	0,03281	0	GSPATT00027607001	0,43	0,06985	0
GSPATT00008104001	1,297	0,00002	1	GSPATT00027613001	-0,299	0,09538	0
GSPATT00008134001	0,932	0,00055	0	GSPATT00027617001	-0,374	0,02379	0
GSPATT00008150001	0,063	0,64187	0	GSPATT00027623001	0,439	0,01037	0
GSPATT00008151001	0,2	0,21222	0	GSPATT00027637001	0,397	0,02895	0
GSPATT00008165001	0,565	0,00488	0	GSPATT00027641001	1,114	0,00049	1
GSPATT00008178001	0,755	0,00688	0	GSPATT00027648001	-0,304	0,04309	0
GSPATT00008179001	-0,238	0,18876	0	GSPATT00027649001	-0,55	0,02463	0
GSPATT00008186001	-0,324	0,05436	0	GSPATT00027665001	0,667	0,04913	0
GSPATT00008216001	0,518	0,06627	0	GSPATT00027672001	-0,668	0,01828	0
GSPATT00008228001	1,643	0,00002	1	GSPATT00027677001	0,078	0,65055	0
GSPATT00008231001	-0,275	0,07499	0	GSPATT00027685001	0,812	0,00085	0
GSPATT00008235001	-0,069	0,63056	0	GSPATT00027690001	0,646	0,00592	0
GSPATT00008243001	0,87	0,00126	0	GSPATT00027700001	0,396	0,14503	0
GSPATT00008313001	-3,997	0	-1	GSPATT00027727001	0,419	0,06587	0
GSPATT00008318001	0,769	0,02343	0	GSPATT00027731001	-0,101	0,74111	0
GSPATT00008324001	0,184	0,37151	0	GSPATT00027740001	0,305	0,04752	0
GSPATT00008331001	0,617	0,11575	0	GSPATT00027741001	0,59	0,00929	0

GSPATT00008341001	0,816	0,00047	0	GSPATT00027763001	0,328	0,03342	0
GSPATT00008356001	0,219	0,14265	0	GSPATT00027770001	0,647	0,00238	0
GSPATT00008371001	0,43	0,01968	0	GSPATT00027787001	0,369	0,04914	0
GSPATT00008373001	0,504	0,0091	0	GSPATT00027796001	-1,461	0,0001	-1
GSPATT00008385001	0,841	0,01833	0	GSPATT00027807001	-2	0	-1
GSPATT00008408001	-0,744	0,00043	0	GSPATT00027838001	0,488	0,03197	0
GSPATT00008410001	0,894	0,00015	0	GSPATT00027852001	-1,233	0,00015	-1
GSPATT00008418001	-0,272	0,07464	0	GSPATT00027857001	0,522	0,0062	0
GSPATT00008426001	-0,189	0,19536	0	GSPATT00027864001	0,379	0,03557	0
GSPATT00008447001	-0,614	0,00381	0	GSPATT00027870001	-0,002	0,9924	0
GSPATT00008453001	0,032	0,86006	0	GSPATT00027898001	0,118	0,48905	0
GSPATT00008463001	1,266	0,00003	1	GSPATT00027906001	0,065	0,67546	0
GSPATT00008472001	0,504	0,00647	0	GSPATT00027913001	0,404	0,09183	0
GSPATT00008481001	0,364	0,12254	0	GSPATT00027933001	-0,886	0,00125	0
GSPATT00008497001	0,172	0,39591	0	GSPATT00027938001	0,112	0,47766	0
GSPATT00008503001	0,019	0,92472	0	GSPATT00027978001	-0,102	0,58439	0
GSPATT00008527001	0,18	0,35417	0	GSPATT00027980001	-0,117	0,44254	0
GSPATT00008559001	-0,208	0,15887	0	GSPATT00028004001	0,144	0,48628	0
GSPATT00008573001	-0,003	0,98576	0	GSPATT00028006001	0,033	0,84856	0
GSPATT00008596001	-1,074	0,00006	-1	GSPATT00028029001	1,144	0,00006	1
GSPATT00008599001	0,085	0,53896	0	GSPATT00028033001	0,014	0,94972	0
GSPATT00008602001	0,371	0,02113	0	GSPATT00028035001	1,108	0,00038	1
GSPATT00008611001	0,235	0,3889	0	GSPATT00028058001	-1,581	0,00253	-1
GSPATT00008619001	-0,021	0,87621	0	GSPATT00028068001	0,819	0,00613	0
GSPATT00008634001	0,014	0,92595	0	GSPATT00028082001	0,851	0,00023	0
GSPATT00008639001	-0,663	0,00927	0	GSPATT00028088001	0,595	0,00642	0
GSPATT00008645001	0,599	0,05259	0	GSPATT00028112001	0,016	0,92503	0
GSPATT00008738001	0,821	0,00201	0	GSPATT00028130001	0,382	0,0628	0
GSPATT00008749001	-0,136	0,49358	0	GSPATT00028137001	-0,328	0,07792	0
GSPATT00008764001	0,257	0,19576	0	GSPATT00028156001	0,053	0,70232	0
GSPATT00008778001	0,342	0,04202	0	GSPATT00028162001	0,316	0,14219	0
GSPATT00008793001	0,729	0,0008	0	GSPATT00028174001	-0,017	0,90512	0
GSPATT00008798001	-0,09	0,60109	0	GSPATT00028181001	0,307	0,0498	0
GSPATT00008805001	0,579	0,00784	0	GSPATT00028199001	1,181	0,00015	1
GSPATT00008830001	0,301	0,18862	0	GSPATT00028210001	-0,239	0,19183	0
GSPATT00008831001	0,097	0,54219	0	GSPATT00028223001	0,071	0,6272	0
GSPATT00008850001	0,887	0,00048	0	GSPATT00028224001	-0,142	0,45435	0
GSPATT00008866001	0,383	0,07319	0	GSPATT00028226001	-0,224	0,18937	0
GSPATT00008880001	-0,665	0,00165	0	GSPATT00028254001	0,074	0,68566	0
GSPATT00008893001	0,303	0,07004	0	GSPATT00028256001	1,144	0,00003	1
GSPATT00008897001	0,334	0,19577	0	GSPATT00028260001	0,201	0,33982	0
GSPATT00008901001	-1,237	0,00007	-1	GSPATT00028261001	0,648	0,00753	0
GSPATT00008924001	0,154	0,39308	0	GSPATT00028264001	-0,912	0,00051	0
GSPATT00008962001	-1,856	0	-1	GSPATT00028265001	-0,664	0,00186	0
GSPATT00008973001	-1,225	0,00169	-1	GSPATT00028266001	-0,643	0,01703	0
GSPATT00008975001	0,694	0,00205	0	GSPATT00028267001	1,612	0,00001	1
GSPATT00008986001	0,293	0,16667	0	GSPATT00028269001	0,88	0,00108	0
GSPATT00009000001	0,215	0,29281	0	GSPATT00028305001	0,264	0,18486	0
GSPATT00009001001	0,558	0,04588	0	GSPATT00028307001	-0,082	0,66338	0
GSPATT00009009001	-0,53	0,00588	0	GSPATT00028312001	0,636	0,00684	0
GSPATT00009013001	1,033	0,00013	1	GSPATT00028334001	-0,21	0,36834	0
GSPATT00009076001	0,364	0,0251	0	GSPATT00028347001	0,779	0,00108	0
GSPATT00009086001	0,586	0,00945	0	GSPATT00028368001	0,41	0,03475	0
GSPATT00009091001	-2,067	0,00001	-1	GSPATT00028374001	0,68	0,00629	0
GSPATT00009102001	0,179	0,23283	0	GSPATT00028387001	-0,687	0,00975	0
GSPATT00009110001	0,013	0,95996	0	GSPATT00028388001	0,698	0,06661	0
GSPATT00009123001	-0,164	0,60057	0	GSPATT00028405001	0,115	0,6059	0
GSPATT00009138001	1,521	0,00028	1	GSPATT00028413001	0,051	0,72041	0
GSPATT00009146001	-0,481	0,05125	0	GSPATT00028458001	-1,195	0,00003	-1
GSPATT00009149001	0,025	0,87197	0	GSPATT00028463001	0,348	0,16251	0
GSPATT00009152001	0,046	0,81853	0	GSPATT00028465001	-0,227	0,14925	0
GSPATT00009155001	-0,238	0,12312	0	GSPATT00028502001	0,619	0,01367	0
GSPATT00009172001	-1,485	0,00015	-1	GSPATT00028521001	-0,347	0,06932	0
GSPATT00009173001	0,244	0,23213	0	GSPATT00028522001	0,518	0,02311	0
GSPATT00009174001	0,724	0,00457	0	GSPATT00028523001	-0,194	0,30675	0
GSPATT00009176001	0,333	0,1816	0	GSPATT00028553001	-1,461	0,00001	-1
GSPATT00009191001	0,221	0,13878	0	GSPATT00028591001	-0,079	0,66532	0

GSPATT00009203001	0,022	0,87442	0	GSPATT00028640001	-0,009	0,94942	0
GSPATT00009207001	1,429	0,00002	1	GSPATT00028664001	1,165	0,00037	1
GSPATT00009211001	1,961	0	1	GSPATT00028690001	0,455	0,03584	0
GSPATT00009267001	-1,116	0,00261	-1	GSPATT00028736001	0,809	0,00541	0
GSPATT00009271001	-0,931	0,00059	0	GSPATT00028737001	1,259	0,00014	1
GSPATT00009291001	0,067	0,70438	0	GSPATT00028739001	0,281	0,07289	0
GSPATT00009324001	0,293	0,0582	0	GSPATT00028760001	-0,016	0,95354	0
GSPATT00009344001	-0,02	0,89901	0	GSPATT00028761001	-0,584	0,00278	0
GSPATT00009346001	0,505	0,00751	0	GSPATT00028762001	0,71	0,00514	0
GSPATT00009355001	-0,135	0,39547	0	GSPATT00028795001	-0,564	0,00507	0
GSPATT00009389001	0,729	0,00072	0	GSPATT00028814001	0,718	0,00439	0
GSPATT00009411001	0,367	0,14277	0	GSPATT00028854001	0,383	0,0558	0
GSPATT00009414001	1,265	0,00079	1	GSPATT00028862001	0,155	0,44362	0
GSPATT00009427001	0,213	0,16056	0	GSPATT00028879001	-0,101	0,52631	0
GSPATT00009428001	0,129	0,62268	0	GSPATT00028902001	0,115	0,49398	0
GSPATT00009429001	-0,821	0,00035	0	GSPATT00028903001	0,487	0,0098	0
GSPATT00009449001	0,122	0,41636	0	GSPATT00028907001	0,31	0,07543	0
GSPATT00009461001	-1,129	0,00293	-1	GSPATT00028913001	0,627	0,00447	0
GSPATT00009466001	0,566	0,00692	0	GSPATT00028920001	0,862	0,00104	0
GSPATT00009502001	-0,497	0,00886	0	GSPATT00028924001	0,504	0,00498	0
GSPATT00009512001	0,817	0,00579	0	GSPATT00028949001	0,858	0,01717	0
GSPATT00009529001	-0,262	0,0797	0	GSPATT00028981001	0,411	0,07642	0
GSPATT00009531001	0,045	0,73938	0	GSPATT00029004001	-0,194	0,28836	0
GSPATT00009538001	-0,422	0,05567	0	GSPATT00029007001	0,785	0,00492	0
GSPATT00009539001	-0,64	0,00235	0	GSPATT00029023001	0,516	0,03057	0
GSPATT00009546001	0,44	0,01605	0	GSPATT00029035001	-0,891	0,00058	0
GSPATT00009552001	-0,641	0,01566	0	GSPATT00029041001	0,57	0,00776	0
GSPATT00009559001	0,264	0,18251	0	GSPATT00029053001	-0,652	0,01479	0
GSPATT00009580001	0,2	0,21252	0	GSPATT00029067001	-0,083	0,53645	0
GSPATT00009588001	0,994	0,04353	0	GSPATT00029101001	-0,869	0,00393	0
GSPATT00009601001	-0,457	0,11601	0	GSPATT00029164001	0,564	0,12006	0
GSPATT00009602001	0,93	0,0008	0	GSPATT00029175001	-0,791	0,00029	0
GSPATT00009607001	0,735	0,01843	0	GSPATT00029187001	0,682	0,01155	0
GSPATT00009626001	0,037	0,77284	0	GSPATT00029196001	2,188	0,00004	1
GSPATT00009643001	0,405	0,01362	0	GSPATT00029198001	0,416	0,04007	0
GSPATT00009645001	0,973	0,00151	0	GSPATT00029217001	0,222	0,14787	0
GSPATT00009646001	0,589	0,00229	0	GSPATT00029249001	-0,351	0,02712	0
GSPATT00009693001	0,347	0,0372	0	GSPATT00029273001	-2,046	0,00011	-1
GSPATT00009703001	-2,787	0	-1	GSPATT00029295001	1,454	0,00404	1
GSPATT00009718001	-0,819	0,00093	0	GSPATT00029341001	0,262	0,14262	0
GSPATT00009748001	0,253	0,1151	0	GSPATT00029366001	0,262	0,1057	0
GSPATT00009760001	-2,631	0	-1	GSPATT00029406001	0,843	0,00341	0
GSPATT00009772001	-0,026	0,84037	0	GSPATT00029408001	0,85	0,0034	0
GSPATT00009782001	-0,119	0,55451	0	GSPATT00029420001	1,305	0,00003	1
GSPATT00009828001	0,419	0,03475	0	GSPATT00029443001	-0,601	0,00289	0
GSPATT00009848001	-0,228	0,27077	0	GSPATT00029444001	1,26	0,00076	1
GSPATT00009873001	1,405	0,00002	1	GSPATT00029470001	0,093	0,49861	0
GSPATT00009883001	0,58	0,00174	0	GSPATT00029472001	0,184	0,20615	0
GSPATT00009888001	0,679	0,00449	0	GSPATT00029475001	0,432	0,06467	0
GSPATT00009892001	-0,164	0,50097	0	GSPATT00029530001	0,081	0,70204	0
GSPATT00009932001	-0,127	0,37952	0	GSPATT00029552001	0,56	0,01662	0
GSPATT00009937001	0,505	0,12425	0	GSPATT00029556001	-0,933	0,00515	0
GSPATT00009951001	-0,279	0,21687	0	GSPATT00029558001	1,707	0,00001	1
GSPATT00009964001	0,607	0,03286	0	GSPATT00029574001	-1,108	0,00039	-1
GSPATT00009969001	0,018	0,89309	0	GSPATT00029583001	0,198	0,35094	0
GSPATT00009980001	0,361	0,01933	0	GSPATT00029601001	1,502	0,00004	1
GSPATT00009997001	0,66	0,0026	0	GSPATT00029614001	-0,616	0,00563	0
GSPATT00010022001	-0,69	0,00171	0	GSPATT00029618001	0,584	0,00422	0
GSPATT00010023001	0,56	0,00691	0	GSPATT00029627001	-0,199	0,15139	0
GSPATT00010038001	0,125	0,38308	0	GSPATT00029631001	0,75	0,00323	0
GSPATT00010040001	-0,369	0,07302	0	GSPATT00029668001	-0,047	0,74205	0
GSPATT00010043001	0,211	0,26138	0	GSPATT00029687001	-0,415	0,0173	0
GSPATT00010044001	0,443	0,07115	0	GSPATT00029713001	0,817	0,00169	0
GSPATT00010047001	0,376	0,04978	0	GSPATT00029723001	0,372	0,11982	0
GSPATT00010069001	0,096	0,49349	0	GSPATT00029731001	0,613	0,0095	0
GSPATT00010072001	0,597	0,04414	0	GSPATT00029755001	0,726	0,00287	0
GSPATT00010088001	0,474	0,01739	0	GSPATT00029761001	0,264	0,06412	0

GSPATT00010089001	-0,02	0,89788	0	GSPATT00029771001	-0,737	0,0067	0
GSPATT00010120001	0,439	0,05835	0	GSPATT00029778001	-0,608	0,00307	0
GSPATT00010125001	0,294	0,08285	0	GSPATT00029822001	0,665	0,00641	0
GSPATT00010132001	1,036	0,00021	1	GSPATT00029823001	-0,53	0,02834	0
GSPATT00010165001	1,057	0,05472	0	GSPATT00029824001	0,784	0,05706	0
GSPATT00010186001	-0,625	0,00879	0	GSPATT00029844001	0,11	0,52769	0
GSPATT00010187001	0,077	0,70848	0	GSPATT00029848001	0,411	0,08951	0
GSPATT00010189001	0,274	0,09078	0	GSPATT00029901001	0,748	0,00121	0
GSPATT00010191001	-0,38	0,03269	0	GSPATT00029929001	1,561	0,00012	1
GSPATT00010192001	-0,341	0,04434	0	GSPATT00029968001	0,19	0,2579	0
GSPATT00010201001	-1,112	0,00403	-1	GSPATT00029971001	-0,01	0,95787	0
GSPATT00010217001	0,055	0,68694	0	GSPATT00030001001	-0,993	0,04787	0
GSPATT00010238001	0,721	0,00063	0	GSPATT00030006001	0,333	0,17202	0
GSPATT00010240001	1,641	0,00002	1	GSPATT00030011001	0,244	0,31877	0
GSPATT00010241001	-0,059	0,67929	0	GSPATT00030033001	-0,25	0,39908	0
GSPATT00010256001	0,667	0,16041	0	GSPATT00030052001	-0,257	0,11428	0
GSPATT00010264001	1,126	0,00022	1	GSPATT00030056001	0,096	0,60981	0
GSPATT00010267001	0,363	0,04122	0	GSPATT00030064001	1,558	0,00015	1
GSPATT00010305001	0,583	0,02695	0	GSPATT00030135001	0,579	0,06809	0
GSPATT00010342001	-0,027	0,88113	0	GSPATT00030142001	0,973	0,0012	0
GSPATT00010360001	0,009	0,9481	0	GSPATT00030146001	-0,644	0,00333	0
GSPATT00010361001	1,344	0,00002	1	GSPATT00030175001	-0,442	0,01694	0
GSPATT00010434001	0,716	0,00843	0	GSPATT00030183001	0,09	0,72161	0
GSPATT00010455001	2,068	0,00001	1	GSPATT00030220001	-0,307	0,04731	0
GSPATT00010465001	0,224	0,16678	0	GSPATT00030230001	0,126	0,43629	0
GSPATT00010478001	0,755	0,0015	0	GSPATT00030249001	1,907	0	1
GSPATT00010489001	0,856	0,01707	0	GSPATT00030250001	0,03	0,86055	0
GSPATT00010520001	1,675	0,00001	1	GSPATT00030251001	0,536	0,02526	0
GSPATT00010535001	0,291	0,15153	0	GSPATT00030268001	0,167	0,24397	0
GSPATT00010550001	-0,166	0,3917	0	GSPATT00030295001	0,775	0,00158	0
GSPATT00010557001	0,305	0,0402	0	GSPATT00030300001	0,307	0,11242	0
GSPATT00010582001	-0,301	0,06581	0	GSPATT00030312001	0,159	0,34993	0
GSPATT00010591001	-0,327	0,06596	0	GSPATT00030338001	0,37	0,02763	0
GSPATT00010597001	0,042	0,75702	0	GSPATT00030339001	0,929	0,00014	0
GSPATT00010598001	0,186	0,32536	0	GSPATT00030340001	-0,629	0,00193	0
GSPATT00010599001	-0,541	0,00596	0	GSPATT00030347001	0,305	0,05631	0
GSPATT00010606001	-0,148	0,37763	0	GSPATT00030360001	1,11	0,00034	1
GSPATT00010622001	0,288	0,09844	0	GSPATT00030377001	0,608	0,03137	0
GSPATT00010631001	-0,002	0,99061	0	GSPATT00030395001	0,632	0,00363	0
GSPATT00010634001	-0,099	0,64063	0	GSPATT00030433001	0,649	0,01931	0
GSPATT00010668001	0,532	0,00424	0	GSPATT00030441001	-1,166	0,00076	-1
GSPATT00010700001	1,693	0	1	GSPATT00030461001	0,787	0,01769	0
GSPATT00010735001	0,406	0,06251	0	GSPATT00030462001	2,069	0	1
GSPATT00010794001	0,388	0,14585	0	GSPATT00030463001	0,18	0,23178	0
GSPATT00010803001	0,375	0,05126	0	GSPATT00030486001	-1,516	0,00003	-1
GSPATT00010807001	0,842	0,00505	0	GSPATT00030525001	0,296	0,14492	0
GSPATT00010834001	-0,748	0,00167	0	GSPATT00030537001	-0,36	0,14602	0
GSPATT00010852001	0,595	0,00207	0	GSPATT00030559001	1,24	0,00015	1
GSPATT00010915001	0,334	0,22897	0	GSPATT00030602001	0,266	0,22578	0
GSPATT00010926001	-0,295	0,08518	0	GSPATT00030614001	-0,182	0,32497	0
GSPATT00010927001	0,963	0,00093	0	GSPATT00030636001	0,66	0,0019	0
GSPATT00010930001	0,326	0,08283	0	GSPATT00030654001	-0,73	0,00085	0
GSPATT00010931001	0,755	0,00409	0	GSPATT00030656001	0,805	0,00115	0
GSPATT00010951001	-0,4	0,08547	0	GSPATT00030663001	0,245	0,35912	0
GSPATT00010956001	-0,78	0,00146	0	GSPATT00030666001	0,411	0,06139	0
GSPATT00010972001	-0,096	0,53691	0	GSPATT00030669001	0,057	0,6915	0
GSPATT00010982001	1,537	0,00037	1	GSPATT00030671001	-0,295	0,30814	0
GSPATT00010994001	-2,479	0	-1	GSPATT00030682001	-0,162	0,29594	0
GSPATT00011004001	0,073	0,58776	0	GSPATT00030687001	0,812	0,01438	0
GSPATT00011062001	-1,37	0,00003	-1	GSPATT00030688001	-0,02	0,90163	0
GSPATT00011094001	-0,248	0,11654	0	GSPATT00030698001	0,071	0,70437	0
GSPATT00011096001	0,27	0,14138	0	GSPATT00030708001	0,502	0,05646	0
GSPATT00011110001	0,439	0,02922	0	GSPATT00030723001	-0,18	0,43523	0
GSPATT00011111001	0,784	0,00245	0	GSPATT00030733001	-1,617	0,00013	-1
GSPATT00011112001	0,611	0,00704	0	GSPATT00030745001	-0,1	0,48372	0
GSPATT00011130001	0,933	0,00615	0	GSPATT00030749001	-0,359	0,06814	0
GSPATT00011132001	-1,165	0,00035	-1	GSPATT00030782001	0,235	0,17113	0

GSPATT00011155001	0,816	0,00288	0	GSPATT00030803001	1,123	0,0001	1
GSPATT00011167001	-0,396	0,02351	0	GSPATT00030806001	0,727	0,0015	0
GSPATT00011173001	-3,857	0	-1	GSPATT00030818001	-0,245	0,23455	0
GSPATT00011179001	0,334	0,11641	0	GSPATT00030826001	-2,147	0,00001	-1
GSPATT00011220001	0,315	0,15075	0	GSPATT00030848001	0,031	0,86428	0
GSPATT00011221001	1,038	0,00031	1	GSPATT00030851001	0,263	0,1287	0
GSPATT00011227001	0,852	0,00196	0	GSPATT00030853001	0,549	0,03624	0
GSPATT00011241001	0,626	0,02469	0	GSPATT00030855001	1,1	0,00013	1
GSPATT00011251001	0,282	0,09853	0	GSPATT00030860001	1,314	0,00014	1
GSPATT00011290001	1,791	0	1	GSPATT00030871001	-0,434	0,01131	0
GSPATT00011305001	-0,492	0,0392	0	GSPATT00030877001	-0,793	0,0011	0
GSPATT00011340001	0,056	0,74423	0	GSPATT00030894001	0,443	0,01543	0
GSPATT00011369001	1,426	0,00002	1	GSPATT00030895001	0,613	0,00519	0
GSPATT00011371001	-0,116	0,58125	0	GSPATT00030907001	0,89	0,00402	0
GSPATT00011372001	-0,323	0,11988	0	GSPATT00030912001	1,631	0,00007	1
GSPATT00011413001	0,314	0,1297	0	GSPATT00030921001	0,509	0,01961	0
GSPATT00011422001	-1,076	0,00614	-1	GSPATT00030945001	0,658	0,01733	0
GSPATT00011430001	0,323	0,07271	0	GSPATT00030956001	-0,545	0,00308	0
GSPATT00011436001	0,608	0,01574	0	GSPATT00030971001	0,707	0,00637	0
GSPATT00011440001	0,123	0,57717	0	GSPATT00030979001	0,011	0,94131	0
GSPATT00011454001	0,507	0,07282	0	GSPATT00030994001	0,373	0,06118	0
GSPATT00011455001	-0,483	0,15659	0	GSPATT00031006001	1,218	0,00004	1
GSPATT00011486001	-0,104	0,60315	0	GSPATT00031028001	-0,522	0,00869	0
GSPATT00011499001	-0,148	0,29042	0	GSPATT00031029001	-0,147	0,30646	0
GSPATT00011515001	0,879	0,00666	0	GSPATT00031066001	-3,25	0	-1
GSPATT00011546001	-0,929	0,00313	0	GSPATT00031082001	0,807	0,00177	0
GSPATT00011547001	0,964	0,00026	0	GSPATT00031097001	-0,034	0,81174	0
GSPATT00011564001	0,676	0,00108	0	GSPATT00031104001	-1,662	0,00021	-1
GSPATT00011565001	0,357	0,06272	0	GSPATT00031107001	0,915	0,00188	0
GSPATT00011566001	0,262	0,07738	0	GSPATT00031110001	1,016	0,0098	1
GSPATT00011580001	-0,02	0,90743	0	GSPATT00031155001	-0,356	0,05023	0
GSPATT00011584001	0,283	0,12359	0	GSPATT00031166001	0,279	0,06965	0
GSPATT00011620001	0,245	0,09049	0	GSPATT00031167001	0,272	0,08891	0
GSPATT00011622001	0,355	0,17874	0	GSPATT00031168001	0,836	0,01523	0
GSPATT00011666001	-0,488	0,00499	0	GSPATT00031178001	0,551	0,0218	0
GSPATT00011685001	-0,399	0,05343	0	GSPATT00031182001	-0,535	0,01622	0
GSPATT00011700001	-0,131	0,51052	0	GSPATT00031221001	-0,004	0,97942	0
GSPATT00011714001	0,124	0,49457	0	GSPATT00031235001	0,643	0,00762	0
GSPATT00011716001	0,632	0,00185	0	GSPATT00031280001	-0,052	0,69595	0
GSPATT00011729001	-0,066	0,66441	0	GSPATT00031285001	-0,236	0,21081	0
GSPATT00011731001	0,353	0,05227	0	GSPATT00031287001	0,155	0,35654	0
GSPATT00011735001	1,538	0,00053	1	GSPATT00031311001	0,32	0,0669	0
GSPATT00011739001	-0,466	0,04961	0	GSPATT00031336001	0,795	0,00047	0
GSPATT00011742001	1,365	0,00005	1	GSPATT00031426001	-1,033	0,00006	-1
GSPATT00011764001	0,296	0,13873	0	GSPATT00031441001	-0,048	0,82385	0
GSPATT00011816001	-0,319	0,13633	0	GSPATT00031454001	0,276	0,1339	0
GSPATT00011825001	-0,485	0,08169	0	GSPATT00031471001	-0,423	0,28841	0
GSPATT00011826001	1,565	0,00133	1	GSPATT00031487001	0,99	0,002	0
GSPATT00011840001	-0,798	0,00327	0	GSPATT00031493001	0,611	0,02344	0
GSPATT00011854001	0,553	0,02078	0	GSPATT00031509001	-0,798	0,00098	0
GSPATT00011856001	0,65	0,07665	0	GSPATT00031532001	1,665	0,00021	1
GSPATT00011857001	0,077	0,76623	0	GSPATT00031546001	0,565	0,00846	0
GSPATT00011864001	-0,091	0,49677	0	GSPATT00031569001	-0,125	0,54879	0
GSPATT00011887001	0,175	0,46958	0	GSPATT00031580001	1,114	0,00029	1
GSPATT00011897001	0,61	0,00299	0	GSPATT00031618001	0,323	0,2332	0
GSPATT00011903001	0,087	0,58179	0	GSPATT00031622001	-0,037	0,78942	0
GSPATT00011917001	0,417	0,15463	0	GSPATT00031623001	-0,002	0,9946	0
GSPATT00011929001	-1,564	0,00004	-1	GSPATT00031630001	1,142	0,00103	1
GSPATT00011952001	0,118	0,38041	0	GSPATT00031642001	-1,737	0,00014	-1
GSPATT00011968001	0,326	0,1663	0	GSPATT00031643001	-1,25	0,00034	-1
GSPATT00011981001	-0,032	0,83233	0	GSPATT00031645001	0,862	0,00026	0
GSPATT00011982001	0,265	0,10595	0	GSPATT00031653001	-0,225	0,14582	0
GSPATT00012043001	0,391	0,11161	0	GSPATT00031661001	0,548	0,02007	0
GSPATT00012044001	-0,04	0,80131	0	GSPATT00031665001	-0,001	0,99648	0
GSPATT00012047001	-0,114	0,39986	0	GSPATT00031682001	-1,39	0,00003	-1
GSPATT00012075001	0,5	0,02536	0	GSPATT00031698001	-0,689	0,03351	0
GSPATT00012141001	0,915	0,00115	0	GSPATT00031701001	-0,914	0,00444	0

GSPATT00012176001	0,545	0,02424	0	GSPATT00031704001	1,254	0,00029	1
GSPATT00012210001	-0,372	0,02535	0	GSPATT00031705001	1,81	0,00002	1
GSPATT00012216001	1,175	0,00014	1	GSPATT00031706001	-0,775	0,0011	0
GSPATT00012223001	0,404	0,08406	0	GSPATT00031738001	-1,034	0,00008	-1
GSPATT00012241001	0,159	0,5354	0	GSPATT00031739001	0,382	0,19043	0
GSPATT00012255001	0,104	0,64586	0	GSPATT00031740001	0,355	0,05225	0
GSPATT00012262001	-1,15	0,00006	-1	GSPATT00031754001	-0,461	0,03297	0
GSPATT00012269001	-0,185	0,39154	0	GSPATT00031756001	-0,463	0,03455	0
GSPATT00012278001	1,263	0,00039	1	GSPATT00031757001	0,716	0,03439	0
GSPATT00012282001	0,837	0,01461	0	GSPATT00031762001	-1,768	0,00003	-1
GSPATT00012286001	-0,79	0,00041	0	GSPATT00031768001	-0,037	0,82459	0
GSPATT00012322001	1,362	0,00005	1	GSPATT00031781001	-0,507	0,02156	0
GSPATT00012339001	1,278	0,00002	1	GSPATT00031782001	-0,24	0,42749	0
GSPATT00012348001	-1,134	0,00014	-1	GSPATT00031797001	-0,011	0,9401	0
GSPATT00012351001	0,299	0,06088	0	GSPATT00031802001	0,907	0,00053	0
GSPATT00012371001	-0,352	0,29514	0	GSPATT00031822001	0,463	0,05116	0
GSPATT00012372001	0,15	0,484	0	GSPATT00031828001	-0,487	0,01743	0
GSPATT00012374001	0,203	0,24495	0	GSPATT00031846001	-0,183	0,21789	0
GSPATT00012376001	0,959	0,00045	0	GSPATT00031865001	0,272	0,16899	0
GSPATT00012379001	0,369	0,07668	0	GSPATT00031924001	0,545	0,00883	0
GSPATT00012395001	0,809	0,0075	0	GSPATT00031948001	-0,37	0,18789	0
GSPATT00012396001	0,319	0,11515	0	GSPATT00031955001	-0,129	0,4888	0
GSPATT00012400001	0,852	0,00032	0	GSPATT00031957001	0,03	0,87201	0
GSPATT00012413001	0,837	0,00048	0	GSPATT00032006001	0,087	0,67206	0
GSPATT00012445001	0,429	0,03783	0	GSPATT00032021001	0,096	0,48485	0
GSPATT00012460001	0,238	0,14276	0	GSPATT00032022001	-0,54	0,01533	0
GSPATT00012470001	-0,034	0,86348	0	GSPATT00032029001	0,283	0,22374	0
GSPATT00012488001	-0,04	0,75807	0	GSPATT00032030001	1,339	0,00008	1
GSPATT00012520001	-2,03	0	-1	GSPATT00032037001	0,772	0,00865	0
GSPATT00012525001	0,13	0,44838	0	GSPATT00032092001	0,508	0,09947	0
GSPATT00012543001	0,86	0,00059	0	GSPATT00032094001	-2,851	0	-1
GSPATT00012570001	0,319	0,06017	0	GSPATT00032095001	0,112	0,41059	0
GSPATT00012610001	0,358	0,06157	0	GSPATT00032100001	0,509	0,00574	0
GSPATT00012628001	0,326	0,03065	0	GSPATT00032104001	-0,024	0,9428	0
GSPATT00012631001	0,442	0,02953	0	GSPATT00032110001	-1,089	0,00427	-1
GSPATT00012633001	0,282	0,10356	0	GSPATT00032111001	-1,233	0,00541	-1
GSPATT00012634001	0,634	0,00459	0	GSPATT00032114001	0,44	0,01977	0
GSPATT00012649001	-0,517	0,01272	0	GSPATT00032136001	-0,671	0,00209	0
GSPATT00012656001	0,231	0,16634	0	GSPATT00032166001	0,482	0,04639	0
GSPATT00012666001	0,427	0,02248	0	GSPATT00032169001	0,497	0,00936	0
GSPATT00012684001	-0,036	0,83675	0	GSPATT00032224001	0,067	0,66182	0
GSPATT00012692001	0,375	0,08064	0	GSPATT00032226001	0,466	0,04696	0
GSPATT00012702001	0,193	0,34721	0	GSPATT00032238001	0,364	0,06778	0
GSPATT00012722001	0,574	0,01609	0	GSPATT00032247001	-0,428	0,012	0
GSPATT00012732001	0,363	0,12773	0	GSPATT00032286001	0,05	0,80461	0
GSPATT00012735001	0,149	0,25751	0	GSPATT00032288001	0,859	0,00547	0
GSPATT00012742001	1,227	0,00011	1	GSPATT00032289001	-0,097	0,49171	0
GSPATT00012746001	0,143	0,51196	0	GSPATT00032301001	-0,947	0,00029	0
GSPATT00012753001	0,157	0,26935	0	GSPATT00032341001	0,829	0,00058	0
GSPATT00012754001	-0,119	0,5919	0	GSPATT00032372001	-0,241	0,09642	0
GSPATT00012755001	1,516	0,00005	1	GSPATT00032392001	0,313	0,05107	0
GSPATT00012756001	0,835	0,00358	0	GSPATT00032421001	-1,221	0,00022	-1
GSPATT00012822001	0,123	0,4374	0	GSPATT00032429001	0,493	0,03216	0
GSPATT00012835001	0,596	0,00563	0	GSPATT00032432001	-0,292	0,20952	0
GSPATT00012838001	0,001	0,99699	0	GSPATT00032442001	0,198	0,2398	0
GSPATT00012852001	1,275	0,00003	1	GSPATT00032444001	-0,967	0,00044	0
GSPATT00012853001	0,823	0,00197	0	GSPATT00032458001	0,703	0,02409	0
GSPATT00012859001	-0,198	0,15153	0	GSPATT00032459001	0,275	0,11268	0
GSPATT00012868001	0,163	0,31281	0	GSPATT00032460001	-0,526	0,00918	0
GSPATT00012875001	0,348	0,03129	0	GSPATT00032522001	-0,127	0,45119	0
GSPATT00012878001	0,169	0,34624	0	GSPATT00032523001	0,812	0,00089	0
GSPATT00012914001	0,157	0,26322	0	GSPATT00032532001	0,103	0,45294	0
GSPATT00012916001	-2,396	0	-1	GSPATT00032533001	-0,17	0,53723	0
GSPATT00012931001	0,812	0,00089	0	GSPATT00032559001	0,124	0,37615	0
GSPATT00012968001	0,154	0,45108	0	GSPATT00032564001	1,204	0,00066	1
GSPATT00012973001	-0,403	0,04733	0	GSPATT00032574001	1,413	0,00228	1
GSPATT00012989001	0,026	0,8519	0	GSPATT00032597001	0,884	0,00108	0

GSPATT00012998001	-0,105	0,43684	0
GSPATT00013005001	-0,158	0,4174	0
GSPATT00013045001	0,257	0,10688	0
GSPATT00013048001	0,033	0,83799	0
GSPATT00013062001	-0,119	0,40756	0
GSPATT00013078001	0,087	0,54212	0
GSPATT00013085001	1,595	0,00002	1
GSPATT00013094001	0,81	0,00674	0
GSPATT00013102001	0,769	0,00426	0
GSPATT00013110001	-1,076	0,00259	-1
GSPATT00013157001	0,235	0,17548	0
GSPATT00013159001	0,37	0,09003	0
GSPATT00013164001	-0,51	0,05354	0
GSPATT00013165001	1,021	0,00106	1
GSPATT00013173001	-0,295	0,08555	0
GSPATT00013174001	0,011	0,93101	0
GSPATT00013205001	0,52	0,00824	0
GSPATT00013243001	-0,027	0,84517	0
GSPATT00013253001	1,885	0,00001	1
GSPATT00013260001	0,645	0,00484	0
GSPATT00013263001	0,563	0,01505	0
GSPATT00013271001	0,175	0,21491	0
GSPATT00013272001	1,673	0,00001	1
GSPATT00013280001	-0,282	0,13129	0
GSPATT00013281001	0,128	0,50204	0
GSPATT00013308001	0,015	0,90603	0
GSPATT00013315001	-0,188	0,20496	0
GSPATT00013327001	-0,009	0,94794	0
GSPATT00013343001	0,267	0,15492	0
GSPATT00013356001	-0,722	0,00162	0
GSPATT00013360001	-0,83	0,00139	0
GSPATT00013364001	-0,11	0,50767	0
GSPATT00013383001	-1,314	0,0001	-1
GSPATT00013453001	0,604	0,02056	0
GSPATT00013470001	0,177	0,28718	0
GSPATT00013490001	0,322	0,07055	0
GSPATT00013494001	0,052	0,75665	0
GSPATT00013502001	-0,243	0,19365	0
GSPATT00013552001	0,741	0,00042	0
GSPATT00013561001	0,271	0,16205	0
GSPATT00013578001	1,991	0,00001	1
GSPATT00013652001	0,191	0,18598	0
GSPATT00013656001	0,171	0,2115	0
GSPATT00013680001	-0,238	0,41631	0
GSPATT00013693001	0,493	0,01038	0
GSPATT00013695001	0,474	0,01357	0
GSPATT00013702001	-0,192	0,32187	0
GSPATT00013754001	-3,054	0	-1
GSPATT00013770001	-0,187	0,4332	0
GSPATT00013783001	1,026	0,01651	1
GSPATT00013790001	0,074	0,5863	0
GSPATT00013801001	0,361	0,0755	0
GSPATT00013825001	-0,769	0,00205	0
GSPATT00013827001	0,481	0,00732	0
GSPATT00013828001	0,419	0,03295	0
GSPATT00013847001	0,139	0,40055	0
GSPATT00013852001	-0,368	0,03397	0
GSPATT00013855001	0,438	0,04631	0
GSPATT00013876001	1,319	0,00047	1
GSPATT00013891001	0,176	0,26584	0
GSPATT00013892001	-1,136	0,00035	-1
GSPATT00013898001	0,162	0,43243	0
GSPATT00013900001	-0,003	0,98838	0
GSPATT00013901001	0,247	0,35648	0
GSPATT00013913001	1,633	0,00001	1
GSPATT00013928001	0,8	0,00105	0
GSPATT00013961001	2,283	0,00001	1
GSPATT00032618001	0,457	0,06613	0
GSPATT00032619001	0,741	0,02826	0
GSPATT00032623001	-0,162	0,37167	0
GSPATT00032648001	0,509	0,00818	0
GSPATT00032649001	0,305	0,06511	0
GSPATT00032667001	1,812	0,00041	1
GSPATT00032670001	0,786	0,00243	0
GSPATT00032697001	0,477	0,0138	0
GSPATT00032706001	0,443	0,01419	0
GSPATT00032739001	0,096	0,63648	0
GSPATT00032755001	-0,448	0,01909	0
GSPATT00032763001	-0,489	0,02531	0
GSPATT00032783001	-0,567	0,00797	0
GSPATT00032787001	-0,864	0,00059	0
GSPATT00032791001	0,581	0,0063	0
GSPATT00032810001	0,795	0,00874	0
GSPATT00032894001	0,722	0,00361	0
GSPATT00032910001	1,39	0,00003	1
GSPATT00032920001	0,74	0,00144	0
GSPATT00032929001	0,137	0,64968	0
GSPATT00032930001	1,256	0,00033	1
GSPATT00032934001	0,256	0,2624	0
GSPATT00032957001	0,434	0,02449	0
GSPATT00032999001	0,68	0,00704	0
GSPATT00033009001	-0,785	0,00143	0
GSPATT00033016001	0,224	0,20102	0
GSPATT00033027001	0,378	0,03882	0
GSPATT00033067001	0,968	0,00046	0
GSPATT00033072001	0,525	0,02731	0
GSPATT00033076001	0,219	0,41915	0
GSPATT00033095001	-1,102	0,00064	-1
GSPATT00033106001	0,765	0,00138	0
GSPATT00033119001	-0,51	0,03205	0
GSPATT00033128001	-1,492	0,00008	-1
GSPATT00033139001	0,432	0,01158	0
GSPATT00033154001	1,026	0,00696	1
GSPATT00033161001	-0,302	0,08205	0
GSPATT00033165001	-0,046	0,8569	0
GSPATT00033167001	-0,34	0,05638	0
GSPATT00033198001	0,629	0,09101	0
GSPATT00033205001	0,673	0,00702	0
GSPATT00033223001	-0,423	0,03978	0
GSPATT00033237001	-0,283	0,16249	0
GSPATT00033250001	-0,453	0,0188	0
GSPATT00033258001	0,335	0,03868	0
GSPATT00033260001	-0,16	0,55088	0
GSPATT00033274001	0,713	0,00057	0
GSPATT00033288001	-0,334	0,19184	0
GSPATT00033306001	-0,043	0,85073	0
GSPATT00033333001	0,576	0,00241	0
GSPATT00033334001	0,4	0,0313	0
GSPATT00033358001	-0,167	0,26712	0
GSPATT00033399001	-0,126	0,62052	0
GSPATT00033466001	-1,107	0,00106	-1
GSPATT00033469001	0,884	0,00069	0
GSPATT00033477001	0,439	0,04845	0
GSPATT00033494001	0,481	0,13408	0
GSPATT00033495001	1,143	0,00006	1
GSPATT00033505001	0,875	0,00023	0
GSPATT00033512001	-3,055	0	-1
GSPATT00033523001	-1,294	0,00005	-1
GSPATT00033534001	0,523	0,02677	0
GSPATT00033537001	0,391	0,03807	0
GSPATT00033539001	0,246	0,40993	0
GSPATT00033568001	-0,204	0,16501	0
GSPATT00033573001	0,114	0,43633	0
GSPATT00033586001	0,277	0,16433	0

GSPATT00013977001	0,458	0,01806	0	GSPATT00033606001	-0,328	0,25723	0
GSPATT00013994001	1,578	0,00006	1	GSPATT00033617001	0,273	0,25396	0
GSPATT00014001001	0,554	0,00382	0	GSPATT00033636001	0,336	0,04963	0
GSPATT00014008001	0,723	0,00107	0	GSPATT00033646001	-0,077	0,64536	0
GSPATT00014044001	0,335	0,03981	0	GSPATT00033676001	0,609	0,03214	0
GSPATT00014052001	1,287	0,00418	1	GSPATT00033682001	-0,181	0,30005	0
GSPATT00014072001	0,024	0,90147	0	GSPATT00033690001	-0,633	0,00229	0
GSPATT00014093001	0,192	0,19217	0	GSPATT00033727001	-0,264	0,20849	0
GSPATT00014104001	0,566	0,03625	0	GSPATT00033743001	0,187	0,34652	0
GSPATT00014124001	-1,671	0,00007	-1	GSPATT00033764001	1,303	0,00004	1
GSPATT00014126001	-0,673	0,00116	0	GSPATT00033780001	1,385	0,00001	1
GSPATT00014132001	2,015	0	1	GSPATT00033786001	0,824	0,01346	0
GSPATT00014139001	0,906	0,02173	0	GSPATT00033798001	0,882	0,00143	0
GSPATT00014152001	0,095	0,60893	0	GSPATT00033801001	0,274	0,11977	0
GSPATT00014158001	1,1	0,00099	1	GSPATT00033810001	-0,178	0,21774	0
GSPATT00014190001	0,605	0,17114	0	GSPATT00033811001	-0,727	0,00103	0
GSPATT00014191001	-0,567	0,0055	0	GSPATT00033826001	0,54	0,01676	0
GSPATT00014196001	-0,265	0,26029	0	GSPATT00033832001	1,004	0,0005	1
GSPATT00014197001	0,509	0,02553	0	GSPATT00033839001	0,232	0,20924	0
GSPATT00014213001	-0,445	0,00944	0	GSPATT00033861001	0,201	0,23013	0
GSPATT00014216001	0,08	0,66083	0	GSPATT00033863001	0,989	0,00013	0
GSPATT00014218001	0,813	0,0031	0	GSPATT00033871001	0,751	0,00036	0
GSPATT00014219001	0,639	0,00371	0	GSPATT00033881001	0,29	0,19304	0
GSPATT00014235001	0,358	0,02401	0	GSPATT00033904001	0,322	0,04932	0
GSPATT00014239001	0,559	0,04551	0	GSPATT00033912001	0,771	0,0063	0
GSPATT00014299001	0,536	0,03039	0	GSPATT00033917001	0,213	0,23333	0
GSPATT00014310001	0,41	0,03511	0	GSPATT00033938001	1,074	0,00021	1
GSPATT00014391001	0,443	0,007	0	GSPATT00033979001	-0,385	0,04696	0
GSPATT00014403001	0,293	0,20293	0	GSPATT00033980001	0,087	0,60132	0
GSPATT00014411001	-0,004	0,97822	0	GSPATT00034005001	1,03	0,00044	1
GSPATT00014414001	0,567	0,00623	0	GSPATT00034018001	-1,92	0	-1
GSPATT00014433001	-0,31	0,10884	0	GSPATT00034024001	1,786	0,00003	1
GSPATT00014438001	0,123	0,39312	0	GSPATT00034063001	-0,183	0,27172	0
GSPATT00014454001	0,575	0,02293	0	GSPATT00034071001	0,388	0,02595	0
GSPATT00014461001	0,95	0,00019	0	GSPATT00034075001	-0,137	0,30288	0
GSPATT00014474001	-1,949	0	-1	GSPATT00034090001	0,737	0,00209	0
GSPATT00014478001	0,152	0,53003	0	GSPATT00034103001	-0,258	0,10215	0
GSPATT00014529001	1,182	0,00018	1	GSPATT00034104001	0,705	0,00489	0
GSPATT00014568001	-0,37	0,05496	0	GSPATT00034124001	-1,017	0,0022	-1
GSPATT00014587001	-0,019	0,88031	0	GSPATT00034141001	0,708	0,00161	0
GSPATT00014606001	0,487	0,04116	0	GSPATT00034156001	-0,057	0,72295	0
GSPATT00014629001	0,582	0,00301	0	GSPATT00034171001	0,663	0,00966	0
GSPATT00014641001	0,648	0,0036	0	GSPATT00034175001	-0,329	0,08396	0
GSPATT00014645001	0,006	0,97144	0	GSPATT00034216001	0,528	0,01503	0
GSPATT00014654001	0,521	0,03824	0	GSPATT00034230001	0,16	0,26608	0
GSPATT00014660001	0,098	0,64509	0	GSPATT00034233001	0,369	0,08874	0
GSPATT00014673001	-0,235	0,2283	0	GSPATT00034270001	1,032	0,00088	1
GSPATT00014674001	0,791	0,00595	0	GSPATT00034305001	0,927	0,0006	0
GSPATT00014676001	0,429	0,07671	0	GSPATT00034308001	-0,353	0,08333	0
GSPATT00014685001	0,887	0,0008	0	GSPATT00034314001	-1,355	0,00021	-1
GSPATT00014692001	-0,278	0,14785	0	GSPATT00034329001	1,249	0,00003	1
GSPATT00014705001	0,351	0,08889	0	GSPATT00034393001	-1,986	0,00002	-1
GSPATT00014745001	0,289	0,08436	0	GSPATT00034405001	-0,584	0,00941	0
GSPATT00014759001	1,203	0,00129	1	GSPATT00034420001	-0,659	0,00959	0
GSPATT00014793001	-0,201	0,47536	0	GSPATT00034421001	0,663	0,12721	0
GSPATT00014796001	1,387	0,00001	1	GSPATT00034447001	-0,601	0,07172	0
GSPATT00014810001	0,609	0,01875	0	GSPATT00034463001	0,197	0,40106	0
GSPATT00014822001	0,187	0,19063	0	GSPATT00034473001	0,036	0,77796	0
GSPATT00014841001	0,356	0,10216	0	GSPATT00034552001	1,257	0,00015	1
GSPATT00014872001	0,244	0,12625	0	GSPATT00034577001	-0,839	0,00058	0
GSPATT00014873001	0,194	0,50101	0	GSPATT00034596001	0,345	0,26159	0
GSPATT00014895001	0,711	0,00204	0	GSPATT00034603001	0,885	0,0034	0
GSPATT00014900001	0,37	0,05706	0	GSPATT00034615001	0,724	0,00057	0
GSPATT00014918001	-0,327	0,04948	0	GSPATT00034623001	0,426	0,02613	0
GSPATT00014943001	-0,826	0,00319	0	GSPATT00034631001	0,527	0,0108	0
GSPATT00014958001	-0,462	0,20687	0	GSPATT00034632001	-0,156	0,30353	0
GSPATT00014962001	-0,017	0,92935	0	GSPATT00034637001	0,89	0,00286	0

GSPATT00014964001	1,045	0,00154	1	GSPATT00034640001	1,246	0,00004	1
GSPATT00014966001	-0,912	0,0003	0	GSPATT00034647001	0,189	0,18304	0
GSPATT00014999001	0,415	0,07919	0	GSPATT00034648001	0,251	0,12628	0
GSPATT00015015001	0,687	0,00414	0	GSPATT00034657001	0,421	0,01754	0
GSPATT00015017001	-1,086	0,00027	-1	GSPATT00034667001	-0,213	0,27669	0
GSPATT00015018001	-0,08	0,70257	0	GSPATT00034770001	0,27	0,13463	0
GSPATT00015021001	-1,722	0,00006	-1	GSPATT00034806001	-0,248	0,17157	0
GSPATT00015033001	-0,23	0,21296	0	GSPATT00034819001	0,638	0,01374	0
GSPATT00015042001	-0,895	0,00041	0	GSPATT00034827001	-0,31	0,07036	0
GSPATT00015043001	0,268	0,46979	0	GSPATT00034860001	0,726	0,00775	0
GSPATT00015055001	0,271	0,36469	0	GSPATT00034862001	0,911	0,00088	0
GSPATT00015057001	1,019	0,00048	1	GSPATT00034863001	-2,801	0	-1
GSPATT00015058001	-0,19	0,26701	0	GSPATT00034885001	0,254	0,14736	0
GSPATT00015064001	0,376	0,08595	0	GSPATT00034888001	0,387	0,09919	0
GSPATT00015109001	-0,005	0,9692	0	GSPATT00034893001	0,489	0,0257	0
GSPATT00015151001	0,296	0,07883	0	GSPATT00034900001	0,251	0,34442	0
GSPATT00015169001	0,432	0,17161	0	GSPATT00034902001	-0,054	0,71793	0
GSPATT00015210001	1,87	0,00003	1	GSPATT00034911001	0,966	0,00178	0
GSPATT00015238001	1,322	0,00004	1	GSPATT00034927001	0,954	0,00128	0
GSPATT00015246001	0,577	0,07312	0	GSPATT00034954001	-0,254	0,17321	0
GSPATT00015257001	-0,315	0,32799	0	GSPATT00034968001	0,919	0,00041	0
GSPATT00015261001	0,509	0,06899	0	GSPATT00034975001	-0,694	0,00453	0
GSPATT00015299001	0,213	0,19618	0	GSPATT00034994001	0,228	0,11464	0
GSPATT00015301001	0,024	0,90708	0	GSPATT00034997001	0,566	0,00681	0
GSPATT00015311001	0,153	0,48604	0	GSPATT00035001001	0,72	0,00113	0
GSPATT00015312001	-0,675	0,00141	0	GSPATT00035014001	0,215	0,22573	0
GSPATT00015325001	0,179	0,42392	0	GSPATT00035015001	-0,804	0,00518	0
GSPATT00015335001	0,857	0,00098	0	GSPATT00035041001	0,7	0,01007	0
GSPATT00015344001	0,355	0,08801	0	GSPATT00035046001	-0,126	0,63021	0
GSPATT00015351001	0,593	0,00674	0	GSPATT00035048001	-0,034	0,8154	0
GSPATT00015356001	-0,33	0,11053	0	GSPATT00035049001	1,06	0,00012	1
GSPATT00015363001	-0,898	0,00028	0	GSPATT00035050001	1,458	0,0075	1
GSPATT00015373001	0,163	0,24261	0	GSPATT00035051001	1,15	0,00008	1
GSPATT00015374001	0,211	0,22091	0	GSPATT00035055001	-2,179	0	-1
GSPATT00015382001	1,396	0,00009	1	GSPATT00035058001	0,603	0,00388	0
GSPATT00015396001	0,853	0,02037	0	GSPATT00035098001	-0,426	0,01033	0
GSPATT00015413001	1,181	0,00207	1	GSPATT00035101001	0,274	0,13516	0
GSPATT00015428001	-0,166	0,38106	0	GSPATT00035109001	0,17	0,21947	0
GSPATT00015448001	1,213	0,00004	1	GSPATT00035113001	-3,146	0	-1
GSPATT00015474001	0,646	0,00966	0	GSPATT00035115001	0,815	0,01116	0
GSPATT00015475001	0,366	0,0282	0	GSPATT00035116001	0,615	0,00474	0
GSPATT00015479001	0,147	0,2715	0	GSPATT00035132001	0,541	0,03772	0
GSPATT00015588001	0,437	0,0437	0	GSPATT00035156001	0,909	0,02167	0
GSPATT00015597001	0,345	0,07945	0	GSPATT00035162001	0,462	0,02625	0
GSPATT00015625001	-1,091	0,00006	-1	GSPATT00035168001	-0,967	0,00127	0
GSPATT00015632001	1,616	0,00334	1	GSPATT00035170001	0,275	0,20192	0
GSPATT00015635001	0,054	0,76188	0	GSPATT00035186001	-0,111	0,58129	0
GSPATT00015640001	0,853	0,00185	0	GSPATT00035215001	-0,514	0,05916	0
GSPATT00015666001	-0,623	0,00761	0	GSPATT00035219001	0,385	0,08349	0
GSPATT00015671001	-0,147	0,30793	0	GSPATT00035221001	1,144	0,00247	1
GSPATT00015678001	0,121	0,52246	0	GSPATT00035230001	0,202	0,239	0
GSPATT00015687001	0,716	0,00115	0	GSPATT00035231001	0,708	0,00161	0
GSPATT00015690001	0,723	0,00416	0	GSPATT00035242001	0,289	0,32037	0
GSPATT00015693001	-0,397	0,09858	0	GSPATT00035265001	-0,98	0,00136	0
GSPATT00015702001	0,703	0,00191	0	GSPATT00035273001	0,212	0,15905	0
GSPATT00015703001	0,498	0,0388	0	GSPATT00035283001	0,586	0,00587	0
GSPATT00015729001	0,223	0,18041	0	GSPATT00035344001	1,515	0,00002	1
GSPATT00015736001	0,804	0,00049	0	GSPATT00035345001	0,075	0,59803	0
GSPATT00015750001	1,122	0,00018	1	GSPATT00035369001	1,788	0	1
GSPATT00015756001	0,261	0,13282	0	GSPATT00035370001	0,774	0,00252	0
GSPATT00015783001	0,994	0,00013	0	GSPATT00035400001	0,193	0,51965	0
GSPATT00015804001	-0,028	0,89234	0	GSPATT00035435001	0,252	0,14704	0
GSPATT00015821001	-0,236	0,16594	0	GSPATT00035439001	0,245	0,18477	0
GSPATT00015826001	-1,025	0,00242	-1	GSPATT00035512001	0,145	0,51374	0
GSPATT00015830001	2,01	0,00006	1	GSPATT00035537001	-0,316	0,13551	0
GSPATT00015840001	-0,032	0,85759	0	GSPATT00035553001	0,19	0,42969	0
GSPATT00015852001	0,793	0,01264	0	GSPATT00035558001	0,435	0,06307	0

GSPATT00015854001	-0,969	0,00154	0	GSPATT00035563001	0,506	0,20376	0
GSPATT00015864001	0,541	0,09018	0	GSPATT00035570001	0,438	0,14386	0
GSPATT00015877001	-0,291	0,21366	0	GSPATT00035586001	0,216	0,24577	0
GSPATT00015881001	0,435	0,06228	0	GSPATT00035588001	1,375	0,00072	1
GSPATT00015890001	0,521	0,0327	0	GSPATT00035590001	-0,098	0,61368	0
GSPATT00015896001	0,187	0,19576	0	GSPATT00035613001	0,93	0,00031	0
GSPATT00015906001	-0,376	0,07667	0	GSPATT00035630001	0,599	0,00235	0
GSPATT00015913001	-3,714	0	-1	GSPATT00035632001	1,647	0,00001	1
GSPATT00015946001	-0,176	0,47867	0	GSPATT00035666001	0,319	0,04691	0
GSPATT00015989001	0,756	0,0358	0	GSPATT00035684001	0,079	0,61168	0
GSPATT00015997001	0,616	0,05511	0	GSPATT00035689001	-0,122	0,45197	0
GSPATT00015998001	0,057	0,70953	0	GSPATT00035711001	-0,148	0,32112	0
GSPATT00016007001	-1,006	0,00099	-1	GSPATT00035735001	-0,093	0,61289	0
GSPATT00016022001	0,297	0,04559	0	GSPATT00035742001	-0,345	0,03219	0
GSPATT00016031001	1,983	0	1	GSPATT00035743001	-0,119	0,44406	0
GSPATT00016054001	-0,06	0,68875	0	GSPATT00035748001	0,686	0,00459	0
GSPATT00016058001	0,403	0,0261	0	GSPATT00035791001	-0,365	0,03388	0
GSPATT00016068001	0,584	0,00865	0	GSPATT00035804001	1,246	0,00002	1
GSPATT00016075001	0,476	0,01419	0	GSPATT00035808001	-0,036	0,8091	0
GSPATT00016078001	-0,46	0,01198	0	GSPATT00035812001	0,025	0,89409	0
GSPATT00016099001	-0,376	0,0569	0	GSPATT00035830001	0,647	0,0186	0
GSPATT00016118001	0,681	0,00081	0	GSPATT00035834001	0,834	0,00201	0
GSPATT00016152001	0,24	0,11484	0	GSPATT00035841001	-0,305	0,05898	0
GSPATT00016163001	0,936	0,00075	0	GSPATT00035845001	0,903	0,0002	0
GSPATT00016186001	0,651	0,00328	0	GSPATT00035846001	-0,018	0,91105	0
GSPATT00016201001	0,264	0,12078	0	GSPATT00035878001	-0,682	0,01003	0
GSPATT00016202001	0,942	0,0002	0	GSPATT00035892001	0,871	0,00133	0
GSPATT00016224001	0,577	0,00541	0	GSPATT00035943001	1,226	0,00006	1
GSPATT00016246001	1,272	0,00554	1	GSPATT00035967001	-1,028	0,00178	-1
GSPATT00016252001	-1,875	0,00002	-1	GSPATT00035984001	0,033	0,81622	0
GSPATT00016286001	0,878	0,0129	0	GSPATT00035987001	0,061	0,629	0
GSPATT00016304001	0,003	0,98948	0	GSPATT00035988001	0,121	0,62093	0
GSPATT00016323001	0,645	0,02514	0	GSPATT00035996001	1,583	0,00009	1
GSPATT00016324001	0,417	0,15142	0	GSPATT00036001001	0,324	0,09202	0
GSPATT00016354001	1,499	0,00002	1	GSPATT00036020001	0,633	0,01487	0
GSPATT00016370001	0,574	0,02089	0	GSPATT00036021001	-0,784	0,08263	0
GSPATT00016374001	0,711	0,02701	0	GSPATT00036025001	-0,396	0,01622	0
GSPATT00016390001	-0,025	0,88764	0	GSPATT00036029001	0,388	0,03177	0
GSPATT00016393001	0,939	0,0007	0	GSPATT00036032001	-0,747	0,00511	0
GSPATT00016396001	-0,359	0,34319	0	GSPATT00036035001	1,064	0,00084	1
GSPATT00016433001	0,343	0,0523	0	GSPATT00036037001	0,29	0,15131	0
GSPATT00016451001	0,509	0,00351	0	GSPATT00036059001	-2,602	0,00001	-1
GSPATT00016461001	2,349	0	1	GSPATT00036080001	-0,995	0,00114	0
GSPATT00016467001	0,07	0,6374	0	GSPATT00036130001	1,008	0,00009	1
GSPATT00016473001	-0,231	0,19236	0	GSPATT00036133001	0,425	0,08763	0
GSPATT00016474001	-0,225	0,18313	0	GSPATT00036142001	0,347	0,06142	0
GSPATT00016477001	0,581	0,00731	0	GSPATT00036146001	-0,276	0,13155	0
GSPATT00016484001	0,021	0,92665	0	GSPATT00036182001	0,139	0,41765	0
GSPATT00016487001	0,393	0,10186	0	GSPATT00036198001	0,154	0,39188	0
GSPATT00016492001	1,714	0,00007	1	GSPATT00036212001	0,53	0,00883	0
GSPATT00016497001	0,83	0,00243	0	GSPATT00036217001	-0,481	0,07457	0
GSPATT00016499001	2,913	0	1	GSPATT00036238001	0,234	0,24062	0
GSPATT00016501001	0,408	0,05581	0	GSPATT00036250001	0,42	0,01334	0
GSPATT00016508001	-0,185	0,31276	0	GSPATT00036252001	-0,676	0,01753	0
GSPATT00016522001	1,486	0,00001	1	GSPATT00036259001	-0,145	0,42835	0
GSPATT00016536001	0,338	0,11356	0	GSPATT00036286001	-0,683	0,00156	0
GSPATT00016547001	-0,209	0,18719	0	GSPATT00036336001	0,222	0,12243	0
GSPATT00016555001	0,08	0,62165	0	GSPATT00036337001	0,687	0,00442	0
GSPATT00016572001	0,158	0,27397	0	GSPATT00036353001	0,737	0,00133	0
GSPATT00016579001	-0,042	0,75571	0	GSPATT00036372001	-0,154	0,42691	0
GSPATT00016580001	1,975	0	1	GSPATT00036394001	-0,422	0,06663	0
GSPATT00016581001	0,583	0,00498	0	GSPATT00036395001	0,718	0,00113	0
GSPATT00016584001	0,48	0,00759	0	GSPATT00036407001	0,356	0,04968	0
GSPATT00016598001	-0,315	0,17406	0	GSPATT00036409001	0,316	0,04048	0
GSPATT00016621001	-0,635	0,00167	0	GSPATT00036434001	0,218	0,17014	0
GSPATT00016625001	0,578	0,01915	0	GSPATT00036444001	-0,56	0,00414	0
GSPATT00016626001	-1,01	0,01795	-1	GSPATT00036445001	0,343	0,02548	0

GSPATT00016635001	1,276	0,00002	1	GSPATT00036459001	1,229	0,0001	1
GSPATT00016642001	0,83	0,00038	0	GSPATT00036469001	0,587	0,01017	0
GSPATT00016644001	-0,085	0,60496	0	GSPATT00036471001	0,051	0,77388	0
GSPATT00016649001	0,549	0,0028	0	GSPATT00036474001	0,054	0,83167	0
GSPATT00016655001	-0,315	0,1207	0	GSPATT00036479001	-0,35	0,11614	0
GSPATT00016684001	0,371	0,03571	0	GSPATT00036483001	-0,011	0,94317	0
GSPATT00016686001	-0,649	0,0057	0	GSPATT00036486001	-0,301	0,19234	0
GSPATT00016687001	-1,172	0,00022	-1	GSPATT00036520001	1,333	0,00049	1
GSPATT00016707001	0,035	0,81827	0	GSPATT00036521001	-1,453	0,00724	-1
GSPATT00016715001	-0,125	0,40578	0	GSPATT00036542001	2,353	0	1
GSPATT00016728001	0,796	0,00369	0	GSPATT00036543001	3,458	0	1
GSPATT00016739001	0,05	0,81698	0	GSPATT00036578001	0,012	0,94123	0
GSPATT00016753001	0,303	0,03871	0	GSPATT00036602001	-0,016	0,91827	0
GSPATT00016756001	0,551	0,01981	0	GSPATT00036604001	0,849	0,00077	0
GSPATT00016834001	-0,177	0,2236	0	GSPATT00036632001	0,404	0,0432	0
GSPATT00016841001	0,199	0,27702	0	GSPATT00036666001	-0,42	0,03381	0
GSPATT00016868001	-0,668	0,00534	0	GSPATT00036671001	-0,905	0,00646	0
GSPATT00016887001	-0,259	0,12658	0	GSPATT00036678001	0,014	0,9207	0
GSPATT00016901001	-0,918	0,00013	0	GSPATT00036688001	0,31	0,04238	0
GSPATT00016943001	-0,089	0,60758	0	GSPATT00036690001	0,725	0,00628	0
GSPATT00016964001	0,04	0,87452	0	GSPATT00036705001	1,156	0,00008	1
GSPATT00016974001	-0,017	0,9121	0	GSPATT00036706001	0,883	0,00746	0
GSPATT00016976001	1,509	0,00004	1	GSPATT00036730001	-0,04	0,79056	0
GSPATT00016985001	0,32	0,1398	0	GSPATT00036752001	0,349	0,08868	0
GSPATT00017038001	2	0	1	GSPATT00036770001	0,189	0,34256	0
GSPATT00017050001	0,473	0,00736	0	GSPATT00036776001	-0,3	0,06534	0
GSPATT00017063001	0,061	0,69591	0	GSPATT00036800001	-0,04	0,79823	0
GSPATT00017094001	0,37	0,12392	0	GSPATT00036806001	-0,03	0,88012	0
GSPATT00017107001	0,512	0,01311	0	GSPATT00036817001	-0,834	0,01188	0
GSPATT00017117001	1,483	0,00007	1	GSPATT00036862001	-1,618	0,00012	-1
GSPATT00017119001	0,567	0,00522	0	GSPATT00036876001	0,655	0,01693	0
GSPATT00017122001	-0,89	0,00112	0	GSPATT00036879001	0,685	0,00059	0
GSPATT00017148001	0,445	0,01161	0	GSPATT00036888001	0,452	0,00944	0
GSPATT00017157001	0,228	0,29231	0	GSPATT00036914001	0,754	0,10066	0
GSPATT00017193001	0,423	0,02362	0	GSPATT00036919001	-0,269	0,14769	0
GSPATT00017227001	0,713	0,01736	0	GSPATT00036925001	0,498	0,00721	0
GSPATT00017228001	-1,143	0,00016	-1	GSPATT00036954001	-0,592	0,0063	0
GSPATT00017248001	0,004	0,98061	0	GSPATT00036971001	-0,218	0,16152	0
GSPATT00017265001	0,224	0,27585	0	GSPATT00037000001	0,283	0,14601	0
GSPATT00017266001	0,228	0,22183	0	GSPATT00037053001	-0,135	0,29881	0
GSPATT00017287001	0,722	0,00261	0	GSPATT00037058001	0,084	0,66195	0
GSPATT00017341001	-0,483	0,07425	0	GSPATT00037066001	-0,414	0,01584	0
GSPATT00017350001	-0,237	0,16156	0	GSPATT00037088001	0,441	0,01862	0
GSPATT00017354001	-0,088	0,60425	0	GSPATT00037094001	-0,008	0,9585	0
GSPATT00017356001	1,623	0,00011	1	GSPATT00037099001	0,395	0,03767	0
GSPATT00017361001	0,54	0,0029	0	GSPATT00037123001	0,774	0,01287	0
GSPATT00017388001	-0,64	0,00112	0	GSPATT00037129001	0,656	0,00104	0
GSPATT00017455001	0,77	0,0007	0	GSPATT00037143001	-0,027	0,85661	0
GSPATT00017456001	0,739	0,00754	0	GSPATT00037145001	0,616	0,02922	0
GSPATT00017488001	0,346	0,03561	0	GSPATT00037148001	-0,358	0,09184	0
GSPATT00017492001	0,873	0,0003	0	GSPATT00037154001	-0,557	0,01543	0
GSPATT00017520001	-0,5	0,016	0	GSPATT00037166001	1	0,00065	0
GSPATT00017543001	1,645	0,00001	1	GSPATT00037168001	0,397	0,02983	0
GSPATT00017557001	0,172	0,32619	0	GSPATT00037172001	0,145	0,33707	0
GSPATT00017560001	1,038	0,00731	1	GSPATT00037175001	0,205	0,26106	0
GSPATT00017576001	1,079	0,00068	1	GSPATT00037211001	0,931	0,00079	0
GSPATT00017619001	-0,144	0,3289	0	GSPATT00037212001	0,689	0,00673	0
GSPATT00017630001	-0,188	0,2095	0	GSPATT00037226001	0,202	0,30966	0
GSPATT00017662001	0,243	0,10339	0	GSPATT00037232001	0,667	0,00174	0
GSPATT00017665001	0,463	0,01095	0	GSPATT00037285001	0,579	0,00719	0
GSPATT00017666001	0,164	0,29665	0	GSPATT00037291001	-2,613	0,00006	-1
GSPATT00017671001	1,215	0,00163	1	GSPATT00037311001	-0,268	0,09834	0
GSPATT00017672001	0,666	0,00486	0	GSPATT00037328001	0,772	0,00301	0
GSPATT00017676001	0,897	0,00014	0	GSPATT00037330001	0,474	0,01266	0
GSPATT00017680001	1,263	0,0002	1	GSPATT00037368001	1,031	0,0019	1
GSPATT00017681001	1,015	0,00199	1	GSPATT00037376001	0,236	0,20339	0
GSPATT00017692001	0,757	0,02894	0	GSPATT00037398001	0,097	0,63269	0

GSPATT00017710001	0,784	0,00357	0	GSPATT00037444001	-0,662	0,00643	0
GSPATT00017712001	-0,359	0,17781	0	GSPATT00037451001	0,414	0,01229	0
GSPATT00017715001	1,019	0,00017	1	GSPATT00037461001	0,206	0,15823	0
GSPATT00017746001	0,26	0,0958	0	GSPATT00037466001	0,211	0,25267	0
GSPATT00017766001	-1,364	0,00026	-1	GSPATT00037469001	0,099	0,53472	0
GSPATT00017771001	0,257	0,1542	0	GSPATT00037471001	0,104	0,58331	0
GSPATT00017791001	-1,005	0,00013	-1	GSPATT00037488001	1,394	0,00067	1
GSPATT00017820001	0,089	0,63426	0	GSPATT00037523001	0,723	0,01928	0
GSPATT00017833001	1,804	0,00001	1	GSPATT00037526001	0,897	0,00043	0
GSPATT00017845001	-0,337	0,04954	0	GSPATT00037566001	-0,05	0,76612	0
GSPATT00017866001	-0,949	0,00081	0	GSPATT00037597001	-0,102	0,55503	0
GSPATT00017868001	-0,188	0,4063	0	GSPATT00037660001	0,186	0,17268	0
GSPATT00017882001	-0,011	0,94767	0	GSPATT00037661001	0,066	0,71158	0
GSPATT00017902001	-0,048	0,73859	0	GSPATT00037688001	0,303	0,04893	0
GSPATT00017904001	0,196	0,20412	0	GSPATT00037699001	0,741	0,00075	0
GSPATT00017915001	0,763	0,00829	0	GSPATT00037711001	-0,444	0,05547	0
GSPATT00017926001	-0,085	0,54936	0	GSPATT00037715001	0,109	0,42522	0
GSPATT00017952001	0,822	0,00042	0	GSPATT00037722001	0,821	0,0005	0
GSPATT00017953001	0,93	0,00178	0	GSPATT00037727001	0,543	0,04234	0
GSPATT00017960001	-0,674	0,00313	0	GSPATT00037737001	1,364	0,0001	1
GSPATT00017963001	-0,275	0,06466	0	GSPATT00037754001	-0,856	0,0023	0
GSPATT00017966001	0,227	0,14352	0	GSPATT00037781001	-1,255	0,00102	-1
GSPATT00017972001	-0,131	0,38508	0	GSPATT00037784001	0,072	0,57853	0
GSPATT00017992001	-0,11	0,50579	0	GSPATT00037789001	2,068	0	1
GSPATT00018004001	0,463	0,03497	0	GSPATT00037792001	-0,369	0,18448	0
GSPATT00018048001	0,735	0,00074	0	GSPATT00037795001	0,362	0,01982	0
GSPATT00018049001	0,948	0,00013	0	GSPATT00037796001	1,094	0,0001	1
GSPATT00018051001	-0,539	0,05915	0	GSPATT00037798001	0,035	0,81328	0
GSPATT00018101001	0,898	0,00614	0	GSPATT00037799001	-0,61	0,0081	0
GSPATT00018127001	0,153	0,36392	0	GSPATT00037811001	0,21	0,2921	0
GSPATT00018132001	0,741	0,02921	0	GSPATT00037815001	0,925	0,00331	0
GSPATT00018133001	-0,081	0,66594	0	GSPATT00037818001	0,928	0,00072	0
GSPATT00018143001	0,268	0,2351	0	GSPATT00037856001	0,303	0,06934	0
GSPATT00018153001	-0,086	0,72806	0	GSPATT00037875001	-0,066	0,66305	0
GSPATT00018163001	0,342	0,02849	0	GSPATT00037876001	0,446	0,01048	0
GSPATT00018188001	0,221	0,21776	0	GSPATT00037938001	0,146	0,5361	0
GSPATT00018202001	0,66	0,01285	0	GSPATT00037939001	0,394	0,02006	0
GSPATT00018223001	-0,409	0,02988	0	GSPATT00037945001	-0,437	0,02189	0
GSPATT00018248001	-0,041	0,77438	0	GSPATT00037967001	-0,06	0,6776	0
GSPATT00018263001	0,814	0,00153	0	GSPATT00037978001	0,461	0,08571	0
GSPATT00018280001	0,316	0,0745	0	GSPATT00038025001	-0,104	0,48304	0
GSPATT00018290001	-0,359	0,04447	0	GSPATT00038027001	0,502	0,05619	0
GSPATT00018291001	0,141	0,45858	0	GSPATT00038035001	0,888	0,00025	0
GSPATT00018293001	0,169	0,30306	0	GSPATT00038045001	-0,396	0,05929	0
GSPATT00018330001	0,511	0,01	0	GSPATT00038090001	1,233	0,00044	1
GSPATT00018389001	-0,094	0,58557	0	GSPATT00038149001	0,258	0,07307	0
GSPATT00018393001	-0,203	0,16787	0	GSPATT00038155001	0,75	0,00768	0
GSPATT00018398001	0,481	0,09586	0	GSPATT00038156001	0,107	0,61347	0
GSPATT00018431001	0,56	0,04386	0	GSPATT00038162001	-0,205	0,24742	0
GSPATT00018438001	0,545	0,01322	0	GSPATT00038171001	-1,199	0,00032	-1
GSPATT00018449001	0,58	0,0723	0	GSPATT00038175001	-0,718	0,00151	0
GSPATT00018471001	-1,113	0,00027	-1	GSPATT00038196001	0,025	0,86468	0
GSPATT00018476001	0,992	0,00041	0	GSPATT00038198001	1,751	0,00019	1
GSPATT00018515001	0,206	0,16244	0	GSPATT00038202001	1,049	0,00014	1
GSPATT00018529001	0,92	0,01084	0	GSPATT00038203001	1,279	0,00007	1
GSPATT00018559001	0,48	0,01489	0	GSPATT00038212001	0,313	0,14446	0
GSPATT00018563001	0,407	0,05649	0	GSPATT00038214001	2,959	0	1
GSPATT00018600001	0,51	0,01239	0	GSPATT00038217001	1,138	0,00032	1
GSPATT00018603001	-0,883	0,002	0	GSPATT00038232001	-1,557	0,00002	-1
GSPATT00018608001	0,219	0,14898	0	GSPATT00038377001	1,241	0,00049	1
GSPATT00018634001	-0,253	0,0798	0	GSPATT00038439001	0,034	0,86173	0
GSPATT00018635001	-4,269	0	-1	GSPATT00038442001	0,573	0,00219	0
GSPATT00018645001	-0,232	0,15845	0	GSPATT00038453001	0,958	0,00056	0
GSPATT00018660001	-0,45	0,12247	0	GSPATT00038457001	0,034	0,80944	0
GSPATT00018666001	0,125	0,44695	0	GSPATT00038462001	0,25	0,17848	0
GSPATT00018671001	0,675	0,00088	0	GSPATT00038469001	1,122	0,00009	1
GSPATT00018681001	-0,683	0,00496	0	GSPATT00038489001	0,49	0,00846	0

GSPATT00018695001	-0,076	0,62948	0	GSPATT00038497001	0,218	0,14041	0
GSPATT00018702001	-0,494	0,02855	0	GSPATT00038500001	-0,057	0,70837	0
GSPATT00018706001	0,301	0,2524	0	GSPATT00038501001	0,115	0,39982	0
GSPATT00018710001	-0,217	0,14626	0	GSPATT00038509001	0,207	0,13138	0
GSPATT00018727001	-0,029	0,84109	0	GSPATT00038517001	0,388	0,06212	0
GSPATT00018728001	-0,429	0,04057	0	GSPATT00038525001	0,766	0,00172	0
GSPATT00018737001	0,112	0,42429	0	GSPATT00038527001	0,262	0,08764	0
GSPATT00018784001	0,107	0,56227	0	GSPATT00038565001	-0,503	0,00816	0
GSPATT00018806001	-0,089	0,50827	0	GSPATT00038594001	-0,531	0,01391	0
GSPATT00018878001	-0,526	0,01436	0	GSPATT00038759001	0,112	0,58404	0
GSPATT00018891001	-0,275	0,12204	0	GSPATT00038767001	0,906	0,00064	0
GSPATT00018918001	0,815	0,00108	0	GSPATT00038772001	0,189	0,25284	0
GSPATT00018920001	-0,177	0,49925	0	GSPATT00038778001	0,45	0,0114	0
GSPATT00018943001	-0,344	0,12601	0	GSPATT00038811001	0,163	0,23056	0
GSPATT00018950001	0,098	0,47385	0	GSPATT00038843001	0,394	0,08578	0
GSPATT00018952001	0,199	0,36354	0	GSPATT00038849001	0,132	0,34446	0
GSPATT00018955001	-1,697	0,00048	-1	GSPATT00038851001	-0,622	0,0036	0
GSPATT00018964001	0,792	0,02949	0	GSPATT00038853001	0,357	0,03403	0
GSPATT00018965001	0,578	0,00897	0	GSPATT00038854001	0,067	0,67394	0
GSPATT00018969001	-0,459	0,03222	0	GSPATT00038872001	-0,347	0,10873	0
GSPATT00018978001	1,009	0,00294	1	GSPATT00038895001	-2,984	0,00003	-1
GSPATT00019012001	0,241	0,25273	0	GSPATT00038896001	-0,151	0,65702	0
GSPATT00019047001	-0,313	0,12451	0	GSPATT00038914001	1,241	0,00033	1
GSPATT00019066001	0,738	0,00884	0	GSPATT00038932001	0,144	0,35304	0
GSPATT00019068001	0,352	0,08916	0	GSPATT00038938001	0,67	0,06631	0
GSPATT00019089001	-0,092	0,50555	0	GSPATT00039020001	1,009	0,00006	1
GSPATT00019098001	-0,396	0,01375	0	GSPATT00039058001	0,227	0,19888	0
GSPATT00019105001	-0,564	0,03875	0	GSPATT00039143001	0,942	0,00145	0
GSPATT00019121001	-0,238	0,13986	0	GSPATT00039147001	-0,456	0,10233	0
GSPATT00019140001	-0,119	0,39295	0	GSPATT00039260001	0,476	0,01736	0
GSPATT00019142001	0,119	0,67944	0	GSPATT00039305001	0,494	0,01965	0
GSPATT00019147001	1,334	0,00037	1	GSPATT00039325001	0,34	0,08666	0
GSPATT00019169001	-0,494	0,02586	0	GSPATT00039337001	0,067	0,62405	0
GSPATT00019217001	0,697	0,00662	0	GSPATT00039369001	-0,01	0,94511	0
GSPATT00019232001	1,248	0,0003	1	GSPATT00039396001	-0,215	0,15116	0
GSPATT00019271001	0,448	0,01195	0	GSPATT00039397001	0,806	0,00031	0
GSPATT00019272001	0,626	0,00167	0	GSPATT00039403001	1,013	0,00053	1
GSPATT00019281001	0,242	0,15235	0	GSPATT00039440001	-0,449	0,0779	0
GSPATT00019357001	-0,269	0,11687	0	GSPATT00039502001	-0,434	0,08958	0
GSPATT00019361001	1,018	0,00339	1	GSPATT00039519001	0,599	0,01071	0
GSPATT00019366001	0,029	0,8276	0	GSPATT00039578001	1,071	0,0029	1
GSPATT00019371001	-1,072	0,00005	-1	GSPATT00039596001	-0,065	0,63218	0
GSPATT00019421001	0,515	0,01534	0	GSPATT00039606001	-0,083	0,73346	0
GSPATT00019446001	0,948	0,01336	0	GSPATT00039607001	0,689	0,00107	0
GSPATT00019447001	1,328	0,00111	1	GSPATT00039617001	-0,125	0,38541	0
GSPATT00019448001	1,274	0,00003	1	GSPATT00039623001	-0,179	0,37866	0
GSPATT00019460001	0,582	0,01671	0	GSPATT00039672001	0,81	0,01218	0
GSPATT00019477001	-0,546	0,02587	0	GSPATT00039701001	0,824	0,06378	0
GSPATT00019493001	-0,409	0,16665	0	GSPATT00039733001	0,707	0,00233	0
GSPATT00019504001	0,507	0,03568	0	GSPATT00039825001	0,881	0,00181	0
GSPATT00019520001	1,19	0,00006	1	PTETT10500005001	0,689	0,01507	0
GSPATT00019546001	0,939	0,00501	0	PTETT10800002001	-0,131	0,51208	0

GO 0043412 macromolecule modification

ID	coeff.	p-value	signif.	ID	coeff.	p-value	signif.
GSPATT00000029001	-0,656	0,02257	0	GSPATT00019584001	0,197	0,24399	0
GSPATT00000047001	0,686	0,01107	0	GSPATT00019589001	-0,23	0,14791	0
GSPATT00000064001	-0,605	0,00365	0	GSPATT00019597001	0,628	0,00396	0
GSPATT00000091001	0,592	0,02302	0	GSPATT00019614001	-0,092	0,48818	0
GSPATT00000093001	0,319	0,15526	0	GSPATT00019621001	1,048	0,0001	1
GSPATT00000160001	-0,436	0,09808	0	GSPATT00019622001	0,562	0,00554	0
GSPATT00000190001	0,762	0,00321	0	GSPATT00019623001	0,397	0,02171	0
GSPATT00000248001	0,46	0,00681	0	GSPATT00019627001	0,267	0,13823	0
GSPATT00000282001	-0,179	0,30932	0	GSPATT00019634001	-0,335	0,03168	0
GSPATT00000295001	0,583	0,02578	0	GSPATT00019648001	0,266	0,10254	0
GSPATT00000324001	-0,092	0,63602	0	GSPATT00019665001	0,352	0,16137	0
GSPATT00000365001	0,81	0,00102	0	GSPATT00019672001	0,262	0,38511	0

GSPATT00000366001	0,639	0,01895	0	GSPATT00019684001	0,599	0,03897	0
GSPATT00000384001	0,653	0,02308	0	GSPATT00019701001	0,633	0,00548	0
GSPATT00000394001	-0,275	0,16617	0	GSPATT00019734001	0,538	0,01044	0
GSPATT00000415001	-0,571	0,00652	0	GSPATT00019758001	-0,857	0,00047	0
GSPATT00000434001	0,504	0,02973	0	GSPATT00019770001	0,159	0,3745	0
GSPATT00000482001	-0,044	0,82968	0	GSPATT00019793001	0,172	0,36914	0
GSPATT00000508001	-0,009	0,95625	0	GSPATT00019834001	0,855	0,00735	0
GSPATT00000542001	0,969	0,00094	0	GSPATT00019835001	0,65	0,03101	0
GSPATT00000559001	0,401	0,04709	0	GSPATT00019840001	0,546	0,02765	0
GSPATT00000560001	0,804	0,0092	0	GSPATT00019849001	1,004	0,00239	1
GSPATT00000594001	-1,714	0,00001	-1	GSPATT00019861001	0,155	0,42691	0
GSPATT00000596001	-1,334	0,00022	-1	GSPATT00019885001	1,059	0,00032	1
GSPATT00000599001	-0,212	0,13935	0	GSPATT00019888001	1,012	0,00029	1
GSPATT00000615001	0,04	0,80664	0	GSPATT00019897001	0,216	0,1244	0
GSPATT00000619001	0,52	0,00518	0	GSPATT00019898001	0,291	0,35823	0
GSPATT00000630001	0,066	0,80692	0	GSPATT00019899001	1,302	0,01819	1
GSPATT00000664001	0,473	0,02458	0	GSPATT00019918001	1,36	0,00661	1
GSPATT00000685001	0,283	0,12345	0	GSPATT00019930001	-0,02	0,89979	0
GSPATT00000704001	1,505	0,00001	1	GSPATT00019935001	0,306	0,30175	0
GSPATT00000760001	1,018	0,00234	1	GSPATT00019962001	0,101	0,63424	0
GSPATT00000783001	0,956	0,007	0	GSPATT00019970001	0,305	0,11022	0
GSPATT00000795001	0,73	0,00542	0	GSPATT00019974001	0,112	0,7056	0
GSPATT00000811001	0,048	0,81575	0	GSPATT00019988001	-0,077	0,59245	0
GSPATT00000817001	-0,289	0,11652	0	GSPATT00020006001	0,694	0,0227	0
GSPATT00000822001	0,006	0,96587	0	GSPATT00020016001	-0,284	0,16066	0
GSPATT00000824001	1,895	0,00001	1	GSPATT00020018001	0,567	0,00877	0
GSPATT00000926001	-0,44	0,07676	0	GSPATT00020040001	0,426	0,13971	0
GSPATT00000934001	0,7	0,00134	0	GSPATT00020051001	0,424	0,02871	0
GSPATT00000950001	-0,039	0,85119	0	GSPATT00020056001	-0,263	0,09627	0
GSPATT00000961001	1,244	0,00014	1	GSPATT00020061001	0,203	0,18858	0
GSPATT00000975001	0,767	0,00162	0	GSPATT00020081001	0,562	0,01428	0
GSPATT00000978001	0,54	0,00309	0	GSPATT00020083001	-0,007	0,96307	0
GSPATT00000985001	-0,053	0,78626	0	GSPATT00020106001	0,617	0,04707	0
GSPATT00000988001	0,476	0,00614	0	GSPATT00020133001	0,915	0,00052	0
GSPATT00001013001	0,18	0,3735	0	GSPATT00020136001	0,721	0,00761	0
GSPATT00001029001	0,351	0,04038	0	GSPATT00020148001	0,628	0,00191	0
GSPATT00001071001	-1,676	0,00003	-1	GSPATT00020151001	0,152	0,25786	0
GSPATT00001077001	-1,834	0	-1	GSPATT00020164001	0,049	0,79466	0
GSPATT00001082001	0,802	0,00363	0	GSPATT00020171001	0,019	0,89222	0
GSPATT00001085001	-0,102	0,55835	0	GSPATT00020175001	-0,408	0,07148	0
GSPATT00001088001	-1,002	0,00029	-1	GSPATT00020181001	-0,28	0,08622	0
GSPATT00001107001	1,346	0,00005	1	GSPATT00020183001	0,379	0,08436	0
GSPATT00001118001	0,362	0,12047	0	GSPATT00020206001	1,187	0,00008	1
GSPATT00001133001	-0,118	0,39808	0	GSPATT00020213001	-1,901	0	-1
GSPATT00001155001	-0,688	0,04688	0	GSPATT00020222001	0,022	0,8861	0
GSPATT00001162001	-0,208	0,17385	0	GSPATT00020225001	0,491	0,02758	0
GSPATT00001174001	-0,137	0,61349	0	GSPATT00020239001	-0,066	0,6879	0
GSPATT00001183001	-1,126	0,00625	-1	GSPATT00020280001	0,417	0,00934	0
GSPATT00001189001	-0,09	0,61561	0	GSPATT00020292001	0,983	0,00426	0
GSPATT00001201001	-1,126	0,00014	-1	GSPATT00020315001	0,421	0,02041	0
GSPATT00001205001	0,174	0,23944	0	GSPATT00020317001	1,39	0,00016	1
GSPATT00001207001	0,62	0,00301	0	GSPATT00020328001	0,132	0,45889	0
GSPATT00001216001	-0,859	0,0049	0	GSPATT00020354001	0,971	0,00377	0
GSPATT00001230001	0,185	0,20391	0	GSPATT00020357001	0,423	0,11371	0
GSPATT00001255001	-0,19	0,27269	0	GSPATT00020367001	0,66	0,05352	0
GSPATT00001260001	1,21	0,00035	1	GSPATT00020375001	0,159	0,26389	0
GSPATT00001268001	-0,542	0,00397	0	GSPATT00020379001	0,528	0,00684	0
GSPATT00001273001	0,742	0,00126	0	GSPATT00020390001	-1,113	0,00014	-1
GSPATT00001279001	0,193	0,33618	0	GSPATT00020397001	0,42	0,02881	0
GSPATT00001292001	-0,196	0,32171	0	GSPATT00020419001	-0,186	0,31238	0
GSPATT00001310001	0,604	0,00221	0	GSPATT00020450001	0,494	0,01321	0
GSPATT00001314001	0,32	0,07355	0	GSPATT00020501001	0,141	0,29138	0
GSPATT00001320001	0,724	0,00683	0	GSPATT00020577001	0,213	0,12758	0
GSPATT00001342001	0,712	0,00317	0	GSPATT00020591001	0,653	0,02277	0
GSPATT00001359001	0,036	0,83426	0	GSPATT00020594001	0,125	0,38102	0
GSPATT00001361001	0,243	0,21121	0	GSPATT00020603001	0,424	0,03461	0
GSPATT00001369001	0,226	0,16319	0	GSPATT00020607001	0,553	0,03187	0

GSPATT00001373001	0,174	0,38899	0	GSPATT00020634001	-0,891	0,00039	0
GSPATT00001413001	-0,893	0,00057	0	GSPATT00020656001	0,384	0,09254	0
GSPATT00001414001	0,294	0,08325	0	GSPATT00020671001	0,4	0,06339	0
GSPATT00001427001	0,007	0,96536	0	GSPATT00020675001	0,023	0,86196	0
GSPATT00001430001	0,427	0,01633	0	GSPATT00020696001	1,227	0,00012	1
GSPATT00001441001	-0,411	0,0844	0	GSPATT00020706001	-2,177	0,00005	-1
GSPATT00001463001	1,174	0,00247	1	GSPATT00020718001	0,498	0,0293	0
GSPATT00001467001	-0,474	0,02385	0	GSPATT00020723001	1,447	0,00002	1
GSPATT00001471001	0,442	0,16312	0	GSPATT00020732001	0,346	0,02628	0
GSPATT00001479001	0,193	0,3499	0	GSPATT00020736001	-0,014	0,93682	0
GSPATT00001494001	-0,073	0,59749	0	GSPATT00020738001	-0,47	0,01442	0
GSPATT00001504001	0,05	0,75727	0	GSPATT00020743001	0,121	0,41116	0
GSPATT00001508001	0,42	0,01888	0	GSPATT00020748001	0,638	0,0469	0
GSPATT00001527001	0,128	0,47689	0	GSPATT00020754001	0,328	0,15362	0
GSPATT00001554001	0,882	0,00038	0	GSPATT00020755001	1,124	0,00008	1
GSPATT00001563001	-0,431	0,02903	0	GSPATT00020757001	0,109	0,4162	0
GSPATT00001592001	1,698	0,00001	1	GSPATT00020787001	0,074	0,69474	0
GSPATT00001610001	0,834	0,00147	0	GSPATT00020798001	-0,359	0,06684	0
GSPATT00001642001	-1,843	0,00008	-1	GSPATT00020807001	0,453	0,03103	0
GSPATT00001650001	0,32	0,08832	0	GSPATT00020812001	0,207	0,32984	0
GSPATT00001662001	0,438	0,17856	0	GSPATT00020816001	-0,014	0,954	0
GSPATT00001682001	-2,067	0,00007	-1	GSPATT00020817001	0,81	0,00504	0
GSPATT00001698001	-0,612	0,00264	0	GSPATT00020820001	0,296	0,15516	0
GSPATT00001746001	1,163	0,00005	1	GSPATT00020837001	-2,103	0	-1
GSPATT00001753001	0,102	0,57872	0	GSPATT00020856001	0,607	0,00205	0
GSPATT00001779001	0,661	0,00281	0	GSPATT00020863001	1,067	0,00014	1
GSPATT00001789001	0,667	0,00963	0	GSPATT00020867001	-0,219	0,23135	0
GSPATT00001794001	-0,276	0,08218	0	GSPATT00020886001	0,561	0,00314	0
GSPATT00001840001	0,602	0,00734	0	GSPATT00020887001	0,231	0,17424	0
GSPATT00001845001	-1,549	0,00003	-1	GSPATT00020897001	0,202	0,34688	0
GSPATT00001852001	1,949	0	1	GSPATT00020921001	0,201	0,27627	0
GSPATT00001853001	1,087	0,00026	1	GSPATT00020942001	-0,24	0,19484	0
GSPATT00001854001	1,3	0,00007	1	GSPATT00021004001	-0,039	0,80561	0
GSPATT00001859001	0,31	0,07571	0	GSPATT00021006001	-0,917	0,00215	0
GSPATT00001864001	0,864	0,00071	0	GSPATT00021015001	-0,087	0,60216	0
GSPATT00001865001	0,55	0,00393	0	GSPATT00021056001	-0,152	0,53854	0
GSPATT00001866001	0,294	0,40339	0	GSPATT00021080001	-0,038	0,88109	0
GSPATT00001870001	0,431	0,01514	0	GSPATT00021083001	1,214	0,00004	1
GSPATT00001871001	0,839	0,00105	0	GSPATT00021086001	0,414	0,02053	0
GSPATT00001873001	2,117	0,00001	1	GSPATT00021094001	0,597	0,01141	0
GSPATT00001911001	-0,107	0,62983	0	GSPATT00021104001	1,014	0,00009	1
GSPATT00001920001	0,423	0,0194	0	GSPATT00021108001	0,03	0,85909	0
GSPATT00002000001	0,994	0,00542	0	GSPATT00021127001	0,409	0,03217	0
GSPATT00002008001	0,799	0,00219	0	GSPATT00021152001	0,566	0,00438	0
GSPATT00002011001	0,938	0,00081	0	GSPATT00021161001	0,405	0,05228	0
GSPATT00002013001	0,586	0,02656	0	GSPATT00021175001	0,819	0,00079	0
GSPATT00002014001	0,546	0,01206	0	GSPATT00021195001	1,22	0,0001	1
GSPATT00002063001	0,381	0,02118	0	GSPATT00021201001	0,446	0,02497	0
GSPATT00002065001	0,716	0,00677	0	GSPATT00021207001	0,704	0,01544	0
GSPATT00002068001	1,376	0,00007	1	GSPATT00021213001	-0,327	0,35092	0
GSPATT00002077001	-0,063	0,72874	0	GSPATT00021222001	-1,069	0,00073	-1
GSPATT00002091001	0,425	0,05442	0	GSPATT00021227001	0,331	0,04285	0
GSPATT00002093001	1,052	0,00126	1	GSPATT00021248001	-0,222	0,12494	0
GSPATT00002094001	-0,116	0,53723	0	GSPATT00021249001	-0,183	0,27756	0
GSPATT00002107001	-0,282	0,23525	0	GSPATT00021250001	0,181	0,35664	0
GSPATT00002108001	0,114	0,44232	0	GSPATT00021252001	2,647	0	1
GSPATT00002114001	0,469	0,04573	0	GSPATT00021256001	0,688	0,00291	0
GSPATT00002115001	0,601	0,02057	0	GSPATT00021258001	1,026	0,00012	1
GSPATT00002129001	1,065	0,00431	1	GSPATT00021270001	0,517	0,01282	0
GSPATT00002132001	0,587	0,01286	0	GSPATT00021271001	0,429	0,01704	0
GSPATT00002156001	0,933	0,0007	0	GSPATT00021273001	1,843	0	1
GSPATT00002182001	1,186	0,00008	1	GSPATT00021274001	1,19	0,00002	1
GSPATT00002186001	0,08	0,52966	0	GSPATT00021275001	1,322	0,00075	1
GSPATT00002191001	1,024	0,00219	1	GSPATT00021294001	-0,03	0,86443	0
GSPATT00002195001	-0,541	0,01959	0	GSPATT00021304001	-0,938	0,00125	0
GSPATT00002196001	-0,094	0,51362	0	GSPATT00021337001	1,12	0,00026	1
GSPATT00002202001	-0,633	0,00593	0	GSPATT00021346001	0,927	0,00175	0

GSPATT00002221001	0,238	0,09743	0	GSPATT00021382001	0,681	0,00268	0
GSPATT00002233001	0,107	0,42961	0	GSPATT00021397001	-0,341	0,04105	0
GSPATT00002241001	-0,266	0,23687	0	GSPATT00021403001	0,008	0,96802	0
GSPATT00002247001	0,656	0,00298	0	GSPATT00021407001	0,546	0,01042	0
GSPATT00002269001	-2,667	0,00001	-1	GSPATT00021410001	0,546	0,03562	0
GSPATT00002272001	0,482	0,00978	0	GSPATT00021416001	0,262	0,11135	0
GSPATT00002284001	0,447	0,0176	0	GSPATT00021423001	0,41	0,01616	0
GSPATT00002285001	0,846	0,02305	0	GSPATT00021473001	-0,067	0,62289	0
GSPATT00002295001	0,216	0,24021	0	GSPATT00021496001	-0,121	0,56931	0
GSPATT00002297001	0,322	0,10354	0	GSPATT00021511001	0,607	0,01408	0
GSPATT00002321001	0,196	0,2005	0	GSPATT00021522001	0,222	0,20683	0
GSPATT00002371001	1,279	0,00502	1	GSPATT00021531001	1,595	0	1
GSPATT00002396001	0,89	0,00446	0	GSPATT00021532001	0,469	0,01357	0
GSPATT00002397001	0,65	0,00105	0	GSPATT00021547001	0,33	0,05213	0
GSPATT00002398001	0,608	0,00382	0	GSPATT00021550001	0,69	0,00115	0
GSPATT00002407001	-0,226	0,24151	0	GSPATT00021566001	0,15	0,38869	0
GSPATT00002410001	0,268	0,0869	0	GSPATT00021592001	0,574	0,00334	0
GSPATT00002422001	0,51	0,00603	0	GSPATT00021605001	-0,285	0,07981	0
GSPATT00002424001	-0,521	0,01826	0	GSPATT00021623001	-0,006	0,97564	0
GSPATT00002429001	0,235	0,11218	0	GSPATT00021625001	-0,609	0,00663	0
GSPATT00002438001	0,165	0,29594	0	GSPATT00021626001	0,481	0,08266	0
GSPATT00002465001	0,13	0,62332	0	GSPATT00021637001	0,858	0,0012	0
GSPATT00002480001	1,183	0,00004	1	GSPATT00021639001	1,696	0,00085	1
GSPATT00002495001	0,174	0,40222	0	GSPATT00021643001	0,057	0,70009	0
GSPATT00002529001	1,935	0	1	GSPATT00021645001	0,312	0,06296	0
GSPATT00002550001	-0,042	0,75069	0	GSPATT00021666001	-0,927	0,0001	0
GSPATT00002553001	0,322	0,07357	0	GSPATT00021705001	0,593	0,01376	0
GSPATT00002557001	0,873	0,00059	0	GSPATT00021709001	1,146	0,00013	1
GSPATT00002575001	0,758	0,01395	0	GSPATT00021737001	2,607	0,00001	1
GSPATT00002585001	0,436	0,08223	0	GSPATT00021745001	-0,479	0,00827	0
GSPATT00002589001	0,394	0,03041	0	GSPATT00021748001	0,772	0,0086	0
GSPATT00002593001	1,076	0,0001	1	GSPATT00021749001	1,147	0,01618	1
GSPATT00002595001	0,321	0,0482	0	GSPATT00021752001	0,314	0,06862	0
GSPATT00002614001	-0,048	0,8212	0	GSPATT00021753001	0,418	0,07228	0
GSPATT00002618001	0,222	0,2786	0	GSPATT00021754001	0,243	0,14094	0
GSPATT00002633001	0,209	0,22321	0	GSPATT00021761001	0,639	0,00204	0
GSPATT00002634001	-0,559	0,00848	0	GSPATT00021763001	1,74	0,00004	1
GSPATT00002648001	-0,019	0,91239	0	GSPATT00021786001	0,144	0,35607	0
GSPATT00002670001	0,301	0,22471	0	GSPATT00021789001	1,156	0,00096	1
GSPATT00002680001	1,006	0,00039	1	GSPATT00021797001	0,664	0,0087	0
GSPATT00002682001	0,464	0,04413	0	GSPATT00021815001	0,094	0,7025	0
GSPATT00002705001	0,629	0,00714	0	GSPATT00021818001	0,452	0,0106	0
GSPATT00002720001	-1,253	0,00068	-1	GSPATT00021839001	-0,624	0,00196	0
GSPATT00002743001	-0,488	0,03306	0	GSPATT00021843001	-0,573	0,01408	0
GSPATT00002752001	0,495	0,06805	0	GSPATT00021861001	-2,393	0,00001	-1
GSPATT00002760001	0,788	0,00112	0	GSPATT00021876001	0,606	0,05246	0
GSPATT00002771001	0,859	0,0012	0	GSPATT00021880001	0,418	0,06147	0
GSPATT00002776001	-0,859	0,00025	0	GSPATT00021882001	-0,509	0,02829	0
GSPATT00002799001	-0,188	0,27826	0	GSPATT00021885001	0,508	0,01902	0
GSPATT00002813001	0,189	0,28818	0	GSPATT00021893001	1,556	0,00001	1
GSPATT00002822001	0,211	0,21934	0	GSPATT00021905001	0,176	0,26244	0
GSPATT00002824001	-0,251	0,09825	0	GSPATT00021907001	-0,742	0,0007	0
GSPATT00002833001	-0,306	0,26468	0	GSPATT00021930001	-0,548	0,007	0
GSPATT00002839001	-0,591	0,0576	0	GSPATT00021935001	0,444	0,0187	0
GSPATT00002846001	-0,271	0,31085	0	GSPATT00021944001	-1,181	0,00069	-1
GSPATT00002865001	-0,375	0,08216	0	GSPATT00021946001	0,249	0,20733	0
GSPATT00002890001	-1,023	0,00005	-1	GSPATT00021955001	1,15	0,00027	1
GSPATT00002908001	1,161	0,00015	1	GSPATT00021964001	0,822	0,01015	0
GSPATT00002917001	-0,703	0,00355	0	GSPATT00021971001	-0,255	0,12333	0
GSPATT00002933001	-2,484	0	-1	GSPATT00021978001	-0,527	0,00915	0
GSPATT00002937001	-0,313	0,07411	0	GSPATT00021986001	1,819	0,00001	1
GSPATT00002954001	-0,433	0,0165	0	GSPATT00022016001	0,205	0,15716	0
GSPATT00002960001	-0,423	0,06926	0	GSPATT00022037001	0,79	0,00181	0
GSPATT00002969001	-0,585	0,02587	0	GSPATT00022042001	0,34	0,05228	0
GSPATT00002999001	1,266	0,00041	1	GSPATT00022061001	-0,298	0,11655	0
GSPATT00003022001	0,072	0,67211	0	GSPATT00022128001	-0,88	0,0016	0
GSPATT00003037001	0,383	0,02776	0	GSPATT00022131001	-0,083	0,72879	0

GSPATT00003054001	0,076	0,56297	0	GSPATT00022141001	1,027	0,00014	1
GSPATT00003075001	0,629	0,02375	0	GSPATT00022151001	-0,741	0,00371	0
GSPATT00003077001	-0,196	0,20764	0	GSPATT00022154001	0,346	0,07096	0
GSPATT00003089001	-0,441	0,11477	0	GSPATT00022155001	0,511	0,00951	0
GSPATT00003090001	-0,149	0,32277	0	GSPATT00022167001	0,329	0,07157	0
GSPATT00003094001	0,49	0,01226	0	GSPATT00022172001	0,608	0,01725	0
GSPATT00003105001	1,596	0,00013	1	GSPATT00022183001	-0,244	0,23912	0
GSPATT00003123001	0,138	0,52706	0	GSPATT00022205001	0,366	0,11152	0
GSPATT00003132001	0,679	0,00156	0	GSPATT00022214001	0,659	0,00671	0
GSPATT00003154001	0,332	0,08672	0	GSPATT00022218001	-0,466	0,01328	0
GSPATT00003156001	0,696	0,00226	0	GSPATT00022236001	1,845	0,00007	1
GSPATT00003167001	0,084	0,63843	0	GSPATT00022240001	0,698	0,0019	0
GSPATT00003168001	1,73	0,00037	1	GSPATT00022249001	0,454	0,00814	0
GSPATT00003185001	0,267	0,31747	0	GSPATT00022251001	-0,514	0,08066	0
GSPATT00003191001	-0,442	0,0623	0	GSPATT00022256001	0,89	0,0088	0
GSPATT00003192001	-0,143	0,32291	0	GSPATT00022278001	0,113	0,6617	0
GSPATT00003194001	1,519	0,00002	1	GSPATT00022290001	1,407	0,00034	1
GSPATT00003198001	0,506	0,02601	0	GSPATT00022296001	1,798	0,00001	1
GSPATT00003200001	0,421	0,03094	0	GSPATT00022304001	0,901	0,00027	0
GSPATT00003205001	-0,564	0,00697	0	GSPATT00022316001	0,995	0,0013	0
GSPATT00003224001	-0,237	0,12454	0	GSPATT00022319001	-0,149	0,47401	0
GSPATT00003229001	0,103	0,49036	0	GSPATT00022364001	0,012	0,94018	0
GSPATT00003230001	0,337	0,26038	0	GSPATT00022373001	0,778	0,00583	0
GSPATT00003236001	1,111	0,00005	1	GSPATT00022375001	0,335	0,05369	0
GSPATT00003248001	-0,663	0,0009	0	GSPATT00022376001	0,282	0,36231	0
GSPATT00003253001	0,103	0,43872	0	GSPATT00022389001	0,267	0,0861	0
GSPATT00003279001	0,867	0,0006	0	GSPATT00022398001	-0,303	0,11489	0
GSPATT00003280001	-0,04	0,80148	0	GSPATT00022403001	0,733	0,00613	0
GSPATT00003283001	0,264	0,25078	0	GSPATT00022415001	0,64	0,00116	0
GSPATT00003292001	0,915	0,00467	0	GSPATT00022426001	0,639	0,00648	0
GSPATT00003309001	-0,973	0,0002	0	GSPATT00022436001	-0,065	0,75967	0
GSPATT00003326001	-0,051	0,69934	0	GSPATT00022478001	-0,264	0,12121	0
GSPATT00003335001	-0,087	0,60586	0	GSPATT00022479001	-0,83	0,0106	0
GSPATT00003339001	0,266	0,15758	0	GSPATT00022490001	0,953	0,00048	0
GSPATT00003344001	0,549	0,00644	0	GSPATT00022560001	0,407	0,01304	0
GSPATT00003348001	-0,128	0,5085	0	GSPATT00022581001	0,197	0,34705	0
GSPATT00003366001	0,829	0,00229	0	GSPATT00022585001	-0,137	0,38681	0
GSPATT00003371001	0,268	0,11218	0	GSPATT00022588001	0,519	0,01581	0
GSPATT00003407001	0,787	0,00042	0	GSPATT00022625001	-0,631	0,00225	0
GSPATT00003425001	-0,517	0,01811	0	GSPATT00022648001	1,169	0,00006	1
GSPATT00003444001	0,165	0,26508	0	GSPATT00022652001	0,216	0,20385	0
GSPATT00003486001	0,113	0,47243	0	GSPATT00022660001	0,792	0,00748	0
GSPATT00003501001	0,158	0,34108	0	GSPATT00022665001	0,156	0,24856	0
GSPATT00003507001	-1,565	0,00001	-1	GSPATT00022666001	1,059	0,00175	1
GSPATT00003528001	0,505	0,01812	0	GSPATT00022671001	0,453	0,06689	0
GSPATT00003535001	0,273	0,06967	0	GSPATT00022672001	0,392	0,0156	0
GSPATT00003572001	0,717	0,01034	0	GSPATT00022676001	0,956	0,00118	0
GSPATT00003620001	0,88	0,00103	0	GSPATT00022680001	0,88	0,01013	0
GSPATT00003647001	0,327	0,21564	0	GSPATT00022705001	0,511	0,00652	0
GSPATT00003651001	0,62	0,00494	0	GSPATT00022722001	-0,149	0,46764	0
GSPATT00003657001	-0,746	0,00329	0	GSPATT00022744001	0,355	0,09992	0
GSPATT00003674001	0,666	0,18862	0	GSPATT00022747001	-0,042	0,84234	0
GSPATT00003681001	-0,103	0,4827	0	GSPATT00022758001	-0,264	0,37366	0
GSPATT00003682001	1,134	0,00047	1	GSPATT00022760001	-0,332	0,06229	0
GSPATT00003692001	0,249	0,08305	0	GSPATT00022772001	0,587	0,00225	0
GSPATT00003699001	-0,208	0,19088	0	GSPATT00022794001	-0,642	0,00449	0
GSPATT00003701001	-1,324	0,00003	-1	GSPATT00022804001	0,571	0,02711	0
GSPATT00003711001	-0,057	0,78591	0	GSPATT00022805001	0,721	0,00384	0
GSPATT00003719001	-2,965	0	-1	GSPATT00022813001	-0,287	0,25917	0
GSPATT00003722001	-0,214	0,14285	0	GSPATT00022862001	0,196	0,23874	0
GSPATT00003724001	-1,06	0,00023	-1	GSPATT00022923001	0,087	0,54996	0
GSPATT00003735001	0,938	0,00036	0	GSPATT00022960001	-0,158	0,24845	0
GSPATT00003784001	-0,762	0,00155	0	GSPATT00022982001	0,79	0,04014	0
GSPATT00003800001	0,707	0,00325	0	GSPATT00022998001	0,587	0,0075	0
GSPATT00003805001	-0,458	0,01837	0	GSPATT00023007001	0,391	0,03401	0
GSPATT00003808001	-1,655	0,00001	-1	GSPATT00023011001	0,775	0,00132	0
GSPATT00003833001	0,314	0,13153	0	GSPATT00023034001	-0,277	0,07151	0

GSPATT00003834001	0,118	0,39846	0	GSPATT00023053001	0,174	0,19072	0
GSPATT00003866001	0,17	0,22326	0	GSPATT00023054001	0,175	0,21904	0
GSPATT00003883001	1,07	0,00005	1	GSPATT00023076001	0,772	0,00846	0
GSPATT00003889001	1,021	0,00011	1	GSPATT00023086001	0,21	0,20719	0
GSPATT00003892001	-0,172	0,33783	0	GSPATT00023095001	-0,105	0,42062	0
GSPATT00003937001	-0,141	0,58292	0	GSPATT00023101001	0,252	0,21939	0
GSPATT00003941001	-0,015	0,90753	0	GSPATT00023116001	0,243	0,2874	0
GSPATT00003946001	0,029	0,8323	0	GSPATT00023168001	-0,346	0,10457	0
GSPATT00003963001	0,533	0,01029	0	GSPATT00023209001	0,505	0,02572	0
GSPATT00003987001	0,889	0,00018	0	GSPATT00023226001	-0,09	0,6793	0
GSPATT00004011001	0,459	0,0181	0	GSPATT00023238001	-0,52	0,00932	0
GSPATT00004041001	-2,104	0,00001	-1	GSPATT00023249001	0,903	0,00029	0
GSPATT00004054001	0,449	0,01063	0	GSPATT00023269001	-0,248	0,34866	0
GSPATT00004057001	-0,473	0,03309	0	GSPATT00023282001	-0,358	0,04566	0
GSPATT00004059001	0,026	0,88876	0	GSPATT00023287001	-0,508	0,01292	0
GSPATT00004060001	0,495	0,02651	0	GSPATT00023291001	-0,368	0,10871	0
GSPATT00004091001	0,299	0,07171	0	GSPATT00023309001	-0,41	0,01263	0
GSPATT00004095001	-0,095	0,61368	0	GSPATT00023311001	0,38	0,03477	0
GSPATT00004114001	0,269	0,09228	0	GSPATT00023324001	0,902	0,00067	0
GSPATT00004132001	-0,103	0,69251	0	GSPATT00023325001	0,851	0,00031	0
GSPATT00004152001	0,206	0,29595	0	GSPATT00023332001	0,059	0,7739	0
GSPATT00004156001	0,259	0,28686	0	GSPATT00023391001	0,729	0,00128	0
GSPATT00004164001	0,47	0,0111	0	GSPATT00023409001	-0,413	0,17182	0
GSPATT00004205001	-0,31	0,17947	0	GSPATT00023417001	0,386	0,08109	0
GSPATT00004210001	0,416	0,03288	0	GSPATT00023422001	-0,559	0,02664	0
GSPATT00004223001	0,612	0,00738	0	GSPATT00023426001	-0,016	0,92617	0
GSPATT00004225001	0,089	0,74057	0	GSPATT00023427001	0,645	0,027	0
GSPATT00004229001	-0,224	0,29312	0	GSPATT00023429001	0,253	0,18096	0
GSPATT00004247001	0,159	0,57122	0	GSPATT00023436001	0,755	0,04632	0
GSPATT00004268001	1,307	0,00011	1	GSPATT00023438001	0,035	0,83414	0
GSPATT00004273001	-0,717	0,00174	0	GSPATT00023469001	-0,011	0,95408	0
GSPATT00004274001	-1,143	0,00023	-1	GSPATT00023488001	1,084	0,00116	1
GSPATT00004298001	0,13	0,50582	0	GSPATT00023514001	0,507	0,00757	0
GSPATT00004311001	0,568	0,00775	0	GSPATT00023531001	0,22	0,46511	0
GSPATT00004321001	0,151	0,6363	0	GSPATT00023540001	0,273	0,10052	0
GSPATT00004366001	0,068	0,69186	0	GSPATT00023549001	-1,224	0,00167	-1
GSPATT00004368001	0,323	0,16554	0	GSPATT00023569001	-0,979	0,00048	0
GSPATT00004374001	1,795	0	1	GSPATT00023571001	-0,22	0,33947	0
GSPATT00004376001	0,343	0,08104	0	GSPATT00023576001	-0,089	0,51115	0
GSPATT00004396001	0,315	0,11278	0	GSPATT00023605001	-0,146	0,31583	0
GSPATT00004405001	0,153	0,39785	0	GSPATT00023613001	0,192	0,1796	0
GSPATT00004412001	-1,59	0,00001	-1	GSPATT00023618001	-0,03	0,8305	0
GSPATT00004440001	-0,239	0,16013	0	GSPATT00023627001	0,203	0,23093	0
GSPATT00004442001	-0,201	0,48124	0	GSPATT00023631001	0,239	0,10083	0
GSPATT00004447001	0,383	0,05161	0	GSPATT00023632001	0,122	0,36366	0
GSPATT00004464001	-0,315	0,08048	0	GSPATT00023650001	0,252	0,19222	0
GSPATT00004480001	0,37	0,07342	0	GSPATT00023676001	0,145	0,31782	0
GSPATT00004496001	0,955	0,00187	0	GSPATT00023690001	-1,078	0,0043	-1
GSPATT00004514001	0,476	0,02673	0	GSPATT00023719001	-0,472	0,04584	0
GSPATT00004528001	0,66	0,00397	0	GSPATT00023728001	0,102	0,45091	0
GSPATT00004559001	0,711	0,00505	0	GSPATT00023729001	0,5	0,071	0
GSPATT00004570001	0,195	0,22266	0	GSPATT00023735001	0,573	0,00261	0
GSPATT00004575001	-0,004	0,97788	0	GSPATT00023790001	-0,309	0,06093	0
GSPATT00004580001	1,242	0,00014	1	GSPATT00023792001	0,035	0,82314	0
GSPATT00004590001	0,499	0,01576	0	GSPATT00023807001	-0,378	0,04035	0
GSPATT00004592001	-0,308	0,04588	0	GSPATT00023823001	-0,725	0,00571	0
GSPATT00004598001	0,48	0,02633	0	GSPATT00023843001	0,248	0,16073	0
GSPATT00004600001	0,083	0,57831	0	GSPATT00023861001	1,352	0,00004	1
GSPATT00004601001	1,962	0,0001	1	GSPATT00023871001	-0,181	0,31269	0
GSPATT00004610001	0,945	0,00014	0	GSPATT00023901001	0,661	0,00244	0
GSPATT00004630001	-0,933	0,00013	0	GSPATT00023907001	0,749	0,00073	0
GSPATT00004644001	-2,245	0,00001	-1	GSPATT00023914001	-0,777	0,0023	0
GSPATT00004648001	0,147	0,30806	0	GSPATT00023937001	-0,496	0,07124	0
GSPATT00004661001	-0,088	0,65447	0	GSPATT00023947001	0,21	0,19097	0
GSPATT00004664001	0,401	0,16244	0	GSPATT00023951001	0,462	0,01336	0
GSPATT00004681001	-0,284	0,11177	0	GSPATT00023962001	-0,49	0,01746	0
GSPATT00004693001	0,654	0,00623	0	GSPATT00023981001	0,056	0,75794	0

GSPATT00004708001	-0,936	0,00106	0	GSPATT00024010001	0,28	0,11337	0
GSPATT00004713001	-0,302	0,08002	0	GSPATT00024035001	0,75	0,00345	0
GSPATT00004716001	-0,265	0,1283	0	GSPATT00024046001	0,488	0,06203	0
GSPATT00004725001	-0,044	0,79332	0	GSPATT00024078001	0,879	0,0027	0
GSPATT00004760001	0,235	0,3263	0	GSPATT00024080001	0,962	0,00084	0
GSPATT00004761001	0,808	0,01968	0	GSPATT00024093001	0,589	0,00369	0
GSPATT00004764001	-0,15	0,28228	0	GSPATT00024120001	0,414	0,05081	0
GSPATT00004777001	1,749	0,00006	1	GSPATT00024158001	0,676	0,00939	0
GSPATT00004785001	0,782	0,00521	0	GSPATT00024181001	0,193	0,36401	0
GSPATT00004807001	0,124	0,60039	0	GSPATT00024182001	-0,819	0,01549	0
GSPATT00004813001	0,679	0,01555	0	GSPATT00024183001	-0,176	0,34939	0
GSPATT00004832001	-0,277	0,07921	0	GSPATT00024185001	1,039	0,00005	1
GSPATT00004834001	0,789	0,00098	0	GSPATT00024203001	0,349	0,11648	0
GSPATT00004846001	2,635	0	1	GSPATT00024205001	0,66	0,00203	0
GSPATT00004860001	-0,019	0,91882	0	GSPATT00024206001	-0,177	0,2138	0
GSPATT00004865001	-0,645	0,01547	0	GSPATT00024209001	1,188	0,00003	1
GSPATT00004869001	0,682	0,00693	0	GSPATT00024218001	-0,321	0,04683	0
GSPATT00004874001	-0,141	0,55683	0	GSPATT00024233001	0,949	0,0003	0
GSPATT00004905001	0,24	0,28818	0	GSPATT00024234001	0,863	0,00016	0
GSPATT00004906001	0,041	0,80854	0	GSPATT00024244001	0,136	0,47521	0
GSPATT00004925001	-0,765	0,01316	0	GSPATT00024253001	0,327	0,12765	0
GSPATT00004954001	-0,94	0,02171	0	GSPATT00024274001	0,022	0,87189	0
GSPATT00004968001	0,709	0,00121	0	GSPATT00024309001	-0,157	0,28831	0
GSPATT00004976001	-0,023	0,86985	0	GSPATT00024335001	-0,34	0,04551	0
GSPATT00004988001	-0,207	0,1401	0	GSPATT00024337001	0,437	0,02363	0
GSPATT00004998001	0,877	0,0004	0	GSPATT00024348001	-0,075	0,56089	0
GSPATT00005003001	0,289	0,06439	0	GSPATT00024349001	0,096	0,597	0
GSPATT00005007001	0,56	0,00815	0	GSPATT00024352001	0,614	0,00859	0
GSPATT00005009001	0,54	0,00742	0	GSPATT00024357001	0,148	0,45854	0
GSPATT00005029001	0,213	0,14145	0	GSPATT00024377001	1,043	0,00342	1
GSPATT00005077001	0,271	0,1489	0	GSPATT00024390001	-0,282	0,13976	0
GSPATT00005093001	0,879	0,00087	0	GSPATT00024398001	0,796	0,01758	0
GSPATT00005095001	0,782	0,00596	0	GSPATT00024407001	-0,816	0,004	0
GSPATT00005098001	0,976	0,00298	0	GSPATT00024410001	0,428	0,02489	0
GSPATT00005100001	-0,27	0,06839	0	GSPATT00024418001	0,221	0,13987	0
GSPATT00005117001	0,136	0,40124	0	GSPATT00024424001	-0,29	0,0846	0
GSPATT00005120001	0,8	0,00223	0	GSPATT00024429001	0,278	0,07692	0
GSPATT00005130001	-0,006	0,97738	0	GSPATT00024448001	-0,278	0,17393	0
GSPATT00005209001	1,585	0,00011	1	GSPATT00024450001	-0,469	0,00704	0
GSPATT00005214001	-0,055	0,72902	0	GSPATT00024451001	0,393	0,03105	0
GSPATT00005235001	-0,322	0,0632	0	GSPATT00024474001	-0,231	0,34278	0
GSPATT00005243001	1,145	0,0018	1	GSPATT00024479001	-0,903	0,00303	0
GSPATT00005245001	0,407	0,07978	0	GSPATT00024481001	0,435	0,1006	0
GSPATT00005247001	-0,102	0,43462	0	GSPATT00024485001	-0,462	0,15535	0
GSPATT00005248001	-1,692	0,00037	-1	GSPATT00024498001	0,272	0,35558	0
GSPATT00005267001	1,049	0,00042	1	GSPATT00024499001	0,56	0,00549	0
GSPATT00005269001	0,265	0,06576	0	GSPATT00024504001	-0,557	0,06748	0
GSPATT00005271001	-0,185	0,35608	0	GSPATT00024512001	1,298	0,00001	1
GSPATT00005274001	0,558	0,00601	0	GSPATT00024514001	0,748	0,01065	0
GSPATT00005284001	0,206	0,14532	0	GSPATT00024536001	0,01	0,96698	0
GSPATT00005295001	0,7	0,00631	0	GSPATT00024539001	-2,028	0	-1
GSPATT00005301001	0,612	0,02995	0	GSPATT00024546001	-0,401	0,04144	0
GSPATT00005326001	1,016	0,00028	1	GSPATT00024564001	0,433	0,05933	0
GSPATT00005329001	0,523	0,01314	0	GSPATT00024565001	1,115	0,00008	1
GSPATT00005343001	-0,049	0,73244	0	GSPATT00024566001	0,629	0,00565	0
GSPATT00005359001	-0,071	0,70866	0	GSPATT00024583001	0,315	0,15638	0
GSPATT00005382001	0,315	0,08827	0	GSPATT00024591001	0,498	0,00429	0
GSPATT00005394001	-2,476	0	-1	GSPATT00024598001	-0,675	0,01914	0
GSPATT00005416001	-0,283	0,0906	0	GSPATT00024618001	0,097	0,49798	0
GSPATT00005424001	-2,387	0	-1	GSPATT00024644001	0,654	0,00896	0
GSPATT00005443001	1,201	0,01436	1	GSPATT00024673001	-0,186	0,23745	0
GSPATT00005458001	-0,786	0,00076	0	GSPATT00024691001	1,052	0,00127	1
GSPATT00005479001	0,453	0,01604	0	GSPATT00024731001	0,285	0,24363	0
GSPATT00005498001	0,586	0,00534	0	GSPATT00024737001	0,421	0,14567	0
GSPATT00005504001	-0,323	0,07461	0	GSPATT00024766001	0,352	0,11206	0
GSPATT00005531001	0,024	0,88617	0	GSPATT00024788001	0,153	0,34111	0
GSPATT00005533001	-0,809	0,00171	0	GSPATT00024845001	0,312	0,05216	0

GSPATT00005536001	0,887	0,00254	0	GSPATT00024859001	1,158	0,00272	1
GSPATT00005555001	0,146	0,36762	0	GSPATT00024862001	0,232	0,28545	0
GSPATT00005556001	0,344	0,07104	0	GSPATT00024885001	-1,562	0,00004	-1
GSPATT00005557001	0,873	0,00334	0	GSPATT00024889001	0,107	0,58905	0
GSPATT00005568001	0,44	0,03873	0	GSPATT00024895001	-0,166	0,43815	0
GSPATT00005581001	0,156	0,32591	0	GSPATT00024913001	-0,542	0,01406	0
GSPATT00005587001	0,343	0,06896	0	GSPATT00024916001	1,07	0,00006	1
GSPATT00005611001	0,102	0,61679	0	GSPATT00024917001	1,027	0,0002	1
GSPATT00005647001	-0,353	0,0216	0	GSPATT00024942001	-0,918	0,00043	0
GSPATT00005692001	-0,035	0,79461	0	GSPATT00024976001	0,84	0,00119	0
GSPATT00005703001	0,426	0,05301	0	GSPATT00024990001	0,807	0,0027	0
GSPATT00005726001	0,372	0,0779	0	GSPATT00025030001	-0,48	0,01979	0
GSPATT00005733001	0,017	0,92434	0	GSPATT00025035001	1,087	0,00028	1
GSPATT00005734001	0,285	0,09036	0	GSPATT00025044001	0,229	0,26111	0
GSPATT00005738001	0,978	0,00023	0	GSPATT00025071001	-0,063	0,7592	0
GSPATT00005752001	1,537	0,00004	1	GSPATT00025076001	0,189	0,30656	0
GSPATT00005757001	0,742	0,00204	0	GSPATT00025088001	0,087	0,7199	0
GSPATT00005760001	-0,591	0,07206	0	GSPATT00025089001	0,396	0,07801	0
GSPATT00005782001	0,537	0,00527	0	GSPATT00025098001	0,473	0,02724	0
GSPATT00005804001	-2,483	0,00045	-1	GSPATT00025106001	0,702	0,01194	0
GSPATT00005811001	0,498	0,03959	0	GSPATT00025128001	0,313	0,11606	0
GSPATT00005814001	-0,041	0,82667	0	GSPATT00025133001	0,871	0,00255	0
GSPATT00005845001	0,153	0,32908	0	GSPATT00025144001	1,216	0,00018	1
GSPATT00005861001	0,66	0,00103	0	GSPATT00025176001	0,858	0,01028	0
GSPATT00005868001	0,142	0,49582	0	GSPATT00025177001	0,141	0,61521	0
GSPATT00005881001	0,784	0,01296	0	GSPATT00025184001	0,545	0,03088	0
GSPATT00005902001	1,463	0,00021	1	GSPATT00025243001	1,014	0,00008	1
GSPATT00005904001	0,174	0,28531	0	GSPATT00025254001	0,909	0,00035	0
GSPATT00005911001	0,788	0,00064	0	GSPATT00025257001	0,582	0,02537	0
GSPATT00005943001	1,376	0,00047	1	GSPATT00025262001	-0,617	0,0118	0
GSPATT00005960001	1,544	0,00007	1	GSPATT00025273001	1,277	0,00074	1
GSPATT00005995001	0,249	0,17873	0	GSPATT00025287001	-2,647	0	-1
GSPATT00006007001	0,509	0,01301	0	GSPATT00025348001	0,917	0,00082	0
GSPATT00006011001	0,426	0,04714	0	GSPATT00025382001	-0,664	0,00239	0
GSPATT00006016001	0,78	0,00524	0	GSPATT00025431001	0,45	0,04878	0
GSPATT00006030001	0,381	0,05068	0	GSPATT00025443001	0,397	0,05816	0
GSPATT00006033001	0,654	0,0075	0	GSPATT00025447001	0,033	0,85164	0
GSPATT00006057001	-0,917	0,00035	0	GSPATT00025450001	0,01	0,9566	0
GSPATT00006063001	-0,097	0,55356	0	GSPATT00025459001	0,443	0,0356	0
GSPATT00006069001	0,895	0,00022	0	GSPATT00025471001	-1,355	0,00017	-1
GSPATT00006075001	0,152	0,28079	0	GSPATT00025472001	0,638	0,01873	0
GSPATT00006080001	0,359	0,11534	0	GSPATT00025473001	1,303	0,00076	1
GSPATT00006094001	0,938	0,00214	0	GSPATT00025476001	1,287	0,00012	1
GSPATT00006125001	0,447	0,0539	0	GSPATT00025481001	-0,394	0,02039	0
GSPATT00006174001	-1,189	0,00097	-1	GSPATT00025487001	0,527	0,00492	0
GSPATT00006199001	-0,122	0,43015	0	GSPATT00025488001	0,117	0,44687	0
GSPATT00006217001	0,043	0,84452	0	GSPATT00025542001	0,154	0,32238	0
GSPATT00006226001	-0,062	0,65778	0	GSPATT00025564001	0,335	0,03391	0
GSPATT00006253001	-0,435	0,0265	0	GSPATT00025565001	0,102	0,44835	0
GSPATT00006272001	0,621	0,03899	0	GSPATT00025585001	0,189	0,32528	0
GSPATT00006280001	0,726	0,00083	0	GSPATT00025594001	-3,032	0	-1
GSPATT00006296001	0,129	0,52836	0	GSPATT00025621001	0,549	0,02246	0
GSPATT00006297001	0,413	0,01659	0	GSPATT00025624001	1,872	0,00011	1
GSPATT00006313001	0,809	0,01243	0	GSPATT00025626001	0,186	0,22873	0
GSPATT00006314001	0,607	0,00237	0	GSPATT00025634001	-0,572	0,0037	0
GSPATT00006315001	0,211	0,21775	0	GSPATT00025641001	-0,136	0,41303	0
GSPATT00006331001	0,84	0,00169	0	GSPATT00025658001	0,311	0,08053	0
GSPATT00006334001	-0,044	0,80576	0	GSPATT00025660001	0,423	0,01588	0
GSPATT00006378001	0,677	0,00317	0	GSPATT00025674001	-0,467	0,00665	0
GSPATT00006379001	0,429	0,03699	0	GSPATT00025677001	0,628	0,0033	0
GSPATT00006390001	-3,443	0	-1	GSPATT00025683001	1,103	0,00903	1
GSPATT00006408001	-0,255	0,28163	0	GSPATT00025688001	0,986	0,00101	0
GSPATT00006415001	-0,218	0,29617	0	GSPATT00025714001	-0,285	0,11387	0
GSPATT00006446001	-0,06	0,78799	0	GSPATT00025724001	-0,118	0,54796	0
GSPATT00006468001	1,644	0,00007	1	GSPATT00025742001	0,095	0,63776	0
GSPATT00006472001	-0,821	0,00056	0	GSPATT00025754001	1,693	0	1
GSPATT00006519001	1,503	0,00026	1	GSPATT00025774001	-3,012	0,00001	-1

GSPATT00006522001	0,067	0,61368	0	GSPATT00025790001	0,722	0,01137	0
GSPATT00006526001	0,784	0,00476	0	GSPATT00025829001	-0,303	0,13502	0
GSPATT00006544001	0,253	0,22418	0	GSPATT00025830001	-0,219	0,47777	0
GSPATT00006575001	-0,075	0,7515	0	GSPATT00025833001	0,318	0,12793	0
GSPATT00006576001	0,119	0,43904	0	GSPATT00025835001	0,577	0,00646	0
GSPATT00006580001	0,511	0,15256	0	GSPATT00025843001	-0,234	0,1386	0
GSPATT00006581001	-0,025	0,86132	0	GSPATT00025874001	0,078	0,61912	0
GSPATT00006592001	0,443	0,14222	0	GSPATT00025891001	0,036	0,78724	0
GSPATT00006594001	2,357	0	1	GSPATT00025892001	0,813	0,00112	0
GSPATT00006615001	0,212	0,32587	0	GSPATT00025893001	0,184	0,26595	0
GSPATT00006642001	1,159	0,00005	1	GSPATT00025925001	-2,389	0,00001	-1
GSPATT00006653001	0,808	0,00058	0	GSPATT00025938001	0,244	0,21258	0
GSPATT00006659001	-0,504	0,0224	0	GSPATT00025977001	0,266	0,20896	0
GSPATT00006666001	-0,748	0,0059	0	GSPATT00025982001	0,105	0,52355	0
GSPATT00006681001	1,225	0,00002	1	GSPATT00025983001	0,713	0,0069	0
GSPATT00006690001	0,625	0,00374	0	GSPATT00025987001	0,777	0,00304	0
GSPATT00006702001	1,003	0,00049	1	GSPATT00025999001	0,214	0,18023	0
GSPATT00006707001	0,096	0,55283	0	GSPATT00026028001	-0,597	0,02174	0
GSPATT00006718001	-1,206	0,0008	-1	GSPATT00026044001	1,043	0,00011	1
GSPATT00006721001	0,259	0,28365	0	GSPATT00026048001	0,131	0,45105	0
GSPATT00006725001	1,26	0,00008	1	GSPATT00026052001	0,22	0,26869	0
GSPATT00006735001	1,208	0,00017	1	GSPATT00026100001	-0,727	0,05157	0
GSPATT00006746001	0,845	0,00197	0	GSPATT00026117001	0,711	0,00252	0
GSPATT00006769001	0,213	0,32339	0	GSPATT00026130001	0,041	0,84926	0
GSPATT00006775001	0,248	0,09259	0	GSPATT00026158001	-0,019	0,89138	0
GSPATT00006782001	0,148	0,27067	0	GSPATT00026165001	1,329	0,00001	1
GSPATT00006852001	0,834	0,00426	0	GSPATT00026166001	0,972	0,00364	0
GSPATT00006853001	0,531	0,04301	0	GSPATT00026167001	0,538	0,00916	0
GSPATT00006854001	0,961	0,00034	0	GSPATT00026169001	0,593	0,0211	0
GSPATT00006864001	0,6	0,00924	0	GSPATT00026182001	0,405	0,01463	0
GSPATT00006875001	0,375	0,03661	0	GSPATT00026208001	-0,305	0,05561	0
GSPATT00006884001	0,278	0,14598	0	GSPATT00026214001	0,126	0,51347	0
GSPATT00006904001	0,29	0,11816	0	GSPATT00026231001	-0,721	0,00236	0
GSPATT00006927001	0,501	0,00669	0	GSPATT00026239001	0,204	0,24495	0
GSPATT00006928001	0,334	0,0274	0	GSPATT00026269001	-0,827	0,00039	0
GSPATT00006948001	0,353	0,03894	0	GSPATT00026274001	0,081	0,55749	0
GSPATT00006949001	0,568	0,03228	0	GSPATT00026292001	0,175	0,24434	0
GSPATT00006953001	0,189	0,35123	0	GSPATT00026323001	-0,937	0,00078	0
GSPATT00006973001	-1,451	0,00032	-1	GSPATT00026336001	-0,538	0,01977	0
GSPATT00006974001	0,738	0,04036	0	GSPATT00026341001	0,56	0,00614	0
GSPATT00006977001	-2,575	0	-1	GSPATT00026346001	-0,833	0,00425	0
GSPATT00007015001	-0,04	0,8039	0	GSPATT00026360001	1,582	0,00001	1
GSPATT00007020001	0,699	0,0179	0	GSPATT00026362001	0,039	0,7821	0
GSPATT00007023001	0,031	0,83742	0	GSPATT00026366001	0,255	0,08585	0
GSPATT00007028001	1,888	0,00035	1	GSPATT00026371001	0,968	0,00219	0
GSPATT00007036001	-1,628	0,00205	-1	GSPATT00026382001	-0,122	0,48679	0
GSPATT00007037001	0,21	0,21843	0	GSPATT00026384001	0,13	0,49373	0
GSPATT00007044001	-0,041	0,75732	0	GSPATT00026401001	0,404	0,01576	0
GSPATT00007068001	-0,215	0,22845	0	GSPATT00026410001	-1,354	0,00004	-1
GSPATT00007081001	-0,453	0,09098	0	GSPATT00026424001	-0,195	0,41235	0
GSPATT00007084001	1,343	0,0001	1	GSPATT00026435001	0,978	0,0003	0
GSPATT00007085001	0,898	0,00027	0	GSPATT00026456001	-0,112	0,49348	0
GSPATT00007086001	-0,397	0,12088	0	GSPATT00026471001	0,689	0,0725	0
GSPATT00007091001	1,465	0,00001	1	GSPATT00026484001	0,101	0,56577	0
GSPATT00007092001	0,27	0,19194	0	GSPATT00026498001	0,273	0,11056	0
GSPATT00007095001	0,892	0,00182	0	GSPATT00026501001	0,573	0,02563	0
GSPATT00007110001	0,57	0,00537	0	GSPATT00026505001	-2,575	0	-1
GSPATT00007113001	0,035	0,79386	0	GSPATT00026528001	0,335	0,1504	0
GSPATT00007130001	0,976	0,0003	0	GSPATT00026538001	0,133	0,33705	0
GSPATT00007151001	0,29	0,08359	0	GSPATT00026558001	-0,121	0,43484	0
GSPATT00007162001	-0,046	0,76877	0	GSPATT00026582001	-0,61	0,00172	0
GSPATT00007168001	2,307	0	1	GSPATT00026597001	-0,036	0,87291	0
GSPATT00007170001	0,256	0,39221	0	GSPATT00026598001	-0,409	0,02421	0
GSPATT00007176001	0,545	0,0517	0	GSPATT00026607001	0,518	0,00768	0
GSPATT00007193001	-0,121	0,39214	0	GSPATT00026611001	0,416	0,09592	0
GSPATT00007196001	0,471	0,05051	0	GSPATT00026620001	-0,023	0,89953	0
GSPATT00007204001	0,658	0,0038	0	GSPATT00026642001	0,375	0,12827	0

GSPATT00007206001	-0,243	0,15376	0	GSPATT00026656001	0,014	0,92729	0
GSPATT00007221001	-0,047	0,73213	0	GSPATT00026672001	0,656	0,00939	0
GSPATT00007228001	-0,424	0,07621	0	GSPATT00026673001	1,475	0,00074	1
GSPATT00007288001	-0,479	0,02516	0	GSPATT00026685001	-0,162	0,49003	0
GSPATT00007295001	1,711	0	1	GSPATT00026711001	-1,004	0,00014	-1
GSPATT00007306001	0,194	0,49349	0	GSPATT00026728001	0,421	0,03621	0
GSPATT00007307001	0,539	0,00329	0	GSPATT00026733001	-0,183	0,2048	0
GSPATT00007337001	0,377	0,05996	0	GSPATT00026736001	0,014	0,9335	0
GSPATT00007341001	-0,138	0,33222	0	GSPATT00026737001	-0,589	0,01656	0
GSPATT00007343001	0,329	0,28753	0	GSPATT00026742001	1,681	0,00062	1
GSPATT00007357001	-0,154	0,51852	0	GSPATT00026751001	0,217	0,23484	0
GSPATT00007369001	-0,3	0,09662	0	GSPATT00026762001	0,081	0,72109	0
GSPATT00007385001	0,031	0,87634	0	GSPATT00026768001	1,3	0,0016	1
GSPATT00007396001	0,747	0,00516	0	GSPATT00026770001	0,781	0,00036	0
GSPATT00007424001	0,679	0,0528	0	GSPATT00026784001	0,447	0,02348	0
GSPATT00007458001	-0,178	0,41913	0	GSPATT00026817001	-0,512	0,01273	0
GSPATT00007469001	0,74	0,00278	0	GSPATT00026853001	-0,021	0,90542	0
GSPATT00007491001	0,204	0,26477	0	GSPATT00026855001	-0,189	0,23205	0
GSPATT00007506001	3,323	0	1	GSPATT00026879001	0,638	0,0089	0
GSPATT00007552001	-0,289	0,16464	0	GSPATT00026882001	0,229	0,14149	0
GSPATT00007569001	-0,059	0,72729	0	GSPATT00026883001	0,746	0,01254	0
GSPATT00007633001	0,585	0,00705	0	GSPATT00026903001	0,385	0,04693	0
GSPATT00007636001	0,951	0,00013	0	GSPATT00026918001	0,426	0,01186	0
GSPATT00007651001	1,374	0,00001	1	GSPATT00026931001	0,327	0,03994	0
GSPATT00007652001	0,604	0,00281	0	GSPATT00026977001	0,418	0,01985	0
GSPATT00007655001	1,115	0,00032	1	GSPATT00027082001	-0,14	0,42006	0
GSPATT00007660001	-2,547	0	-1	GSPATT00027084001	-2,917	0	-1
GSPATT00007679001	-0,046	0,77776	0	GSPATT00027094001	-0,238	0,23457	0
GSPATT00007689001	0,756	0,0049	0	GSPATT00027100001	0,499	0,06172	0
GSPATT00007699001	0,698	0,00069	0	GSPATT00027115001	-0,008	0,97395	0
GSPATT00007733001	-0,021	0,908	0	GSPATT00027119001	0,768	0,00101	0
GSPATT00007750001	0,245	0,21356	0	GSPATT00027122001	-0,502	0,01341	0
GSPATT00007792001	0,379	0,04772	0	GSPATT00027138001	0,18	0,19509	0
GSPATT00007794001	-1,257	0,00002	-1	GSPATT00027147001	-0,082	0,6483	0
GSPATT00007797001	0,346	0,07773	0	GSPATT00027149001	0,147	0,32384	0
GSPATT00007801001	0,358	0,05841	0	GSPATT00027156001	0,039	0,76929	0
GSPATT00007808001	0,931	0,00109	0	GSPATT00027189001	0,049	0,79446	0
GSPATT00007826001	0,485	0,01651	0	GSPATT00027203001	0,063	0,64969	0
GSPATT00007852001	0,616	0,00193	0	GSPATT00027205001	-0,221	0,24032	0
GSPATT00007857001	1,41	0,00014	1	GSPATT00027222001	0,074	0,59482	0
GSPATT00007860001	0,07	0,62078	0	GSPATT00027241001	0,321	0,05768	0
GSPATT00007861001	0,494	0,03195	0	GSPATT00027257001	0,806	0,00465	0
GSPATT00007899001	0,019	0,90526	0	GSPATT00027270001	0,745	0,00887	0
GSPATT00007911001	-0,929	0,02523	0	GSPATT00027312001	-1,048	0,00047	-1
GSPATT00007917001	0,096	0,67711	0	GSPATT00027324001	0,003	0,98698	0
GSPATT00007919001	0,035	0,87791	0	GSPATT00027355001	-0,308	0,10498	0
GSPATT00007922001	0,835	0,01888	0	GSPATT00027372001	1,25	0,00004	1
GSPATT00007930001	-0,448	0,05141	0	GSPATT00027401001	0,365	0,07066	0
GSPATT00007946001	1,164	0,00002	1	GSPATT00027404001	-0,378	0,02671	0
GSPATT00007947001	0,256	0,17572	0	GSPATT00027408001	0,032	0,82286	0
GSPATT00007949001	1,323	0,00004	1	GSPATT00027410001	0,523	0,0081	0
GSPATT00007953001	0,179	0,38295	0	GSPATT00027442001	-0,097	0,65217	0
GSPATT00007957001	0,151	0,34529	0	GSPATT00027453001	-0,38	0,0322	0
GSPATT00007961001	-0,294	0,05705	0	GSPATT00027465001	-1,097	0,00007	-1
GSPATT00007964001	-2,161	0,00001	-1	GSPATT00027470001	0,073	0,75047	0
GSPATT00007966001	0,728	0,03307	0	GSPATT00027479001	0,392	0,10569	0
GSPATT00007989001	0,044	0,84104	0	GSPATT00027483001	0,725	0,00101	0
GSPATT00007997001	0,533	0,00985	0	GSPATT00027492001	1,241	0,00093	1
GSPATT00007998001	0,219	0,19699	0	GSPATT00027506001	0,183	0,46769	0
GSPATT00008011001	0,371	0,20891	0	GSPATT00027520001	-0,336	0,16994	0
GSPATT00008014001	0,284	0,11593	0	GSPATT00027535001	0,825	0,01036	0
GSPATT00008021001	-1,112	0,00013	-1	GSPATT00027546001	-0,383	0,03735	0
GSPATT00008031001	-0,45	0,02946	0	GSPATT00027549001	0,322	0,09184	0
GSPATT00008071001	0,691	0,00225	0	GSPATT00027607001	0,43	0,06985	0
GSPATT00008074001	-0,457	0,03281	0	GSPATT00027613001	-0,299	0,09538	0
GSPATT00008104001	1,297	0,00002	1	GSPATT00027617001	-0,374	0,02379	0
GSPATT00008134001	0,932	0,00055	0	GSPATT00027623001	0,439	0,01037	0

GSPATT00008150001	0,063	0,64187	0	GSPATT00027637001	0,397	0,02895	0
GSPATT00008151001	0,2	0,21222	0	GSPATT00027641001	1,114	0,00049	1
GSPATT00008165001	0,565	0,00488	0	GSPATT00027648001	-0,304	0,04309	0
GSPATT00008178001	0,755	0,00688	0	GSPATT00027649001	-0,55	0,02463	0
GSPATT00008179001	-0,238	0,18876	0	GSPATT00027665001	0,667	0,04913	0
GSPATT00008186001	-0,324	0,05436	0	GSPATT00027672001	-0,668	0,01828	0
GSPATT00008216001	0,518	0,06627	0	GSPATT00027677001	0,078	0,65055	0
GSPATT00008228001	1,643	0,00002	1	GSPATT00027685001	0,812	0,00085	0
GSPATT00008231001	-0,275	0,07499	0	GSPATT00027690001	0,646	0,00592	0
GSPATT00008235001	-0,069	0,63056	0	GSPATT00027700001	0,396	0,14503	0
GSPATT00008243001	0,87	0,00126	0	GSPATT00027727001	0,419	0,06587	0
GSPATT00008313001	-3,997	0	-1	GSPATT00027731001	-0,101	0,74111	0
GSPATT00008318001	0,769	0,02343	0	GSPATT00027740001	0,305	0,04752	0
GSPATT00008324001	0,184	0,37151	0	GSPATT00027741001	0,59	0,00929	0
GSPATT00008331001	0,617	0,11575	0	GSPATT00027763001	0,328	0,03342	0
GSPATT00008341001	0,816	0,00047	0	GSPATT00027770001	0,647	0,00238	0
GSPATT00008356001	0,219	0,14265	0	GSPATT00027787001	0,369	0,04914	0
GSPATT00008371001	0,43	0,01968	0	GSPATT00027796001	-1,461	0,0001	-1
GSPATT00008373001	0,504	0,0091	0	GSPATT00027807001	-2	0	-1
GSPATT00008385001	0,841	0,01833	0	GSPATT00027838001	0,488	0,03197	0
GSPATT00008408001	-0,744	0,00043	0	GSPATT00027852001	-1,233	0,00015	-1
GSPATT00008410001	0,894	0,00015	0	GSPATT00027857001	0,522	0,0062	0
GSPATT00008418001	-0,272	0,07464	0	GSPATT00027864001	0,379	0,03557	0
GSPATT00008426001	-0,189	0,19536	0	GSPATT00027870001	-0,002	0,9924	0
GSPATT00008447001	-0,614	0,00381	0	GSPATT00027898001	0,118	0,48905	0
GSPATT00008453001	0,032	0,86006	0	GSPATT00027906001	0,065	0,67546	0
GSPATT00008463001	1,266	0,00003	1	GSPATT00027913001	0,404	0,09183	0
GSPATT00008472001	0,504	0,00647	0	GSPATT00027933001	-0,886	0,00125	0
GSPATT00008481001	0,364	0,12254	0	GSPATT00027938001	0,112	0,47766	0
GSPATT00008497001	0,172	0,39591	0	GSPATT00027978001	-0,102	0,58439	0
GSPATT00008503001	0,019	0,92472	0	GSPATT00027980001	-0,117	0,44254	0
GSPATT00008527001	0,18	0,35417	0	GSPATT00028004001	0,144	0,48628	0
GSPATT00008559001	-0,208	0,15887	0	GSPATT00028006001	0,033	0,84856	0
GSPATT00008573001	-0,003	0,98576	0	GSPATT00028029001	1,144	0,00006	1
GSPATT00008596001	-1,074	0,00006	-1	GSPATT00028033001	0,014	0,94972	0
GSPATT00008599001	0,085	0,53896	0	GSPATT00028035001	1,108	0,00038	1
GSPATT00008602001	0,371	0,02113	0	GSPATT00028058001	-1,581	0,00253	-1
GSPATT00008611001	0,235	0,3889	0	GSPATT00028068001	0,819	0,00613	0
GSPATT00008619001	-0,021	0,87621	0	GSPATT00028082001	0,851	0,00023	0
GSPATT00008634001	0,014	0,92595	0	GSPATT00028088001	0,595	0,00642	0
GSPATT00008639001	-0,663	0,00927	0	GSPATT00028112001	0,016	0,92503	0
GSPATT00008645001	0,599	0,05259	0	GSPATT00028130001	0,382	0,0628	0
GSPATT00008738001	0,821	0,00201	0	GSPATT00028137001	-0,328	0,07792	0
GSPATT00008749001	-0,136	0,49358	0	GSPATT00028156001	0,053	0,70232	0
GSPATT00008764001	0,257	0,19576	0	GSPATT00028162001	0,316	0,14219	0
GSPATT00008778001	0,342	0,04202	0	GSPATT00028174001	-0,017	0,90512	0
GSPATT00008793001	0,729	0,0008	0	GSPATT00028181001	0,307	0,0498	0
GSPATT00008798001	-0,09	0,60109	0	GSPATT00028199001	1,181	0,00015	1
GSPATT00008805001	0,579	0,00784	0	GSPATT00028210001	-0,239	0,19183	0
GSPATT00008830001	0,301	0,18862	0	GSPATT00028223001	0,071	0,6272	0
GSPATT00008831001	0,097	0,54219	0	GSPATT00028224001	-0,142	0,45435	0
GSPATT00008850001	0,887	0,00048	0	GSPATT00028226001	-0,224	0,18937	0
GSPATT00008866001	0,383	0,07319	0	GSPATT00028254001	0,074	0,68566	0
GSPATT00008880001	-0,665	0,00165	0	GSPATT00028256001	1,144	0,00003	1
GSPATT00008893001	0,303	0,07004	0	GSPATT00028260001	0,201	0,33982	0
GSPATT00008897001	0,334	0,19577	0	GSPATT00028261001	0,648	0,00753	0
GSPATT00008901001	-1,237	0,00007	-1	GSPATT00028264001	-0,912	0,00051	0
GSPATT00008924001	0,154	0,39308	0	GSPATT00028265001	-0,664	0,00186	0
GSPATT00008962001	-1,856	0	-1	GSPATT00028266001	-0,643	0,01703	0
GSPATT00008973001	-1,225	0,00169	-1	GSPATT00028267001	1,612	0,00001	1
GSPATT00008975001	0,694	0,00205	0	GSPATT00028269001	0,88	0,00108	0
GSPATT00008986001	0,293	0,16667	0	GSPATT00028305001	0,264	0,18486	0
GSPATT00009000001	0,215	0,29281	0	GSPATT00028307001	-0,082	0,66338	0
GSPATT00009001001	0,558	0,04588	0	GSPATT00028312001	0,636	0,00684	0
GSPATT00009009001	-0,53	0,00588	0	GSPATT00028334001	-0,21	0,36834	0
GSPATT00009013001	1,033	0,00013	1	GSPATT00028347001	0,779	0,00108	0
GSPATT00009076001	0,364	0,0251	0	GSPATT00028368001	0,41	0,03475	0

GSPATT00009086001	0,586	0,00945	0	GSPATT00028374001	0,68	0,00629	0
GSPATT00009091001	-2,067	0,00001	-1	GSPATT00028387001	-0,687	0,00975	0
GSPATT00009102001	0,179	0,23283	0	GSPATT00028388001	0,698	0,06661	0
GSPATT00009110001	0,013	0,95996	0	GSPATT00028405001	0,115	0,6059	0
GSPATT00009123001	-0,164	0,60057	0	GSPATT00028413001	0,051	0,72041	0
GSPATT00009138001	1,521	0,00028	1	GSPATT00028458001	-1,195	0,00003	-1
GSPATT00009146001	-0,481	0,05125	0	GSPATT00028463001	0,348	0,16251	0
GSPATT00009149001	0,025	0,87197	0	GSPATT00028465001	-0,227	0,14925	0
GSPATT00009152001	0,046	0,81853	0	GSPATT00028502001	0,619	0,01367	0
GSPATT00009155001	-0,238	0,12312	0	GSPATT00028521001	-0,347	0,06932	0
GSPATT00009172001	-1,485	0,00015	-1	GSPATT00028522001	0,518	0,02311	0
GSPATT00009173001	0,244	0,23213	0	GSPATT00028523001	-0,194	0,30675	0
GSPATT00009174001	0,724	0,00457	0	GSPATT00028553001	-1,461	0,00001	-1
GSPATT00009176001	0,333	0,1816	0	GSPATT00028591001	-0,079	0,66532	0
GSPATT00009191001	0,221	0,13878	0	GSPATT00028640001	-0,009	0,94942	0
GSPATT00009203001	0,022	0,87442	0	GSPATT00028664001	1,165	0,00037	1
GSPATT00009207001	1,429	0,00002	1	GSPATT00028690001	0,455	0,03584	0
GSPATT00009211001	1,961	0	1	GSPATT00028736001	0,809	0,00541	0
GSPATT00009267001	-1,116	0,00261	-1	GSPATT00028737001	1,259	0,00014	1
GSPATT00009271001	-0,931	0,00059	0	GSPATT00028739001	0,281	0,07289	0
GSPATT00009291001	0,067	0,70438	0	GSPATT00028760001	-0,016	0,95354	0
GSPATT00009324001	0,293	0,0582	0	GSPATT00028761001	-0,584	0,00278	0
GSPATT00009344001	-0,02	0,89901	0	GSPATT00028762001	0,71	0,00514	0
GSPATT00009346001	0,505	0,00751	0	GSPATT00028795001	-0,564	0,00507	0
GSPATT00009355001	-0,135	0,39547	0	GSPATT00028814001	0,718	0,00439	0
GSPATT00009389001	0,729	0,00072	0	GSPATT00028854001	0,383	0,0558	0
GSPATT00009411001	0,367	0,14277	0	GSPATT00028862001	0,155	0,44362	0
GSPATT00009414001	1,265	0,00079	1	GSPATT00028879001	-0,101	0,52631	0
GSPATT00009427001	0,213	0,16056	0	GSPATT00028902001	0,115	0,49398	0
GSPATT00009428001	0,129	0,62268	0	GSPATT00028903001	0,487	0,0098	0
GSPATT00009429001	-0,821	0,00035	0	GSPATT00028907001	0,31	0,07543	0
GSPATT00009449001	0,122	0,41636	0	GSPATT00028913001	0,627	0,00447	0
GSPATT00009461001	-1,129	0,00293	-1	GSPATT00028920001	0,862	0,00104	0
GSPATT00009466001	0,566	0,00692	0	GSPATT00028924001	0,504	0,00498	0
GSPATT00009502001	-0,497	0,00886	0	GSPATT00028949001	0,858	0,01717	0
GSPATT00009512001	0,817	0,00579	0	GSPATT00028981001	0,411	0,07642	0
GSPATT00009529001	-0,262	0,0797	0	GSPATT00029004001	-0,194	0,28836	0
GSPATT00009531001	0,045	0,73938	0	GSPATT00029007001	0,785	0,00492	0
GSPATT00009538001	-0,422	0,05567	0	GSPATT00029023001	0,516	0,03057	0
GSPATT00009539001	-0,64	0,00235	0	GSPATT00029035001	-0,891	0,00058	0
GSPATT00009546001	0,44	0,01605	0	GSPATT00029041001	0,57	0,00776	0
GSPATT00009552001	-0,641	0,01566	0	GSPATT00029053001	-0,652	0,01479	0
GSPATT00009559001	0,264	0,18251	0	GSPATT00029067001	-0,083	0,53645	0
GSPATT00009580001	0,2	0,21252	0	GSPATT00029101001	-0,869	0,00393	0
GSPATT00009588001	0,994	0,04353	0	GSPATT00029164001	0,564	0,12006	0
GSPATT00009601001	-0,457	0,11601	0	GSPATT00029175001	-0,791	0,00029	0
GSPATT00009602001	0,93	0,0008	0	GSPATT00029187001	0,682	0,01155	0
GSPATT00009607001	0,735	0,01843	0	GSPATT00029196001	2,188	0,00004	1
GSPATT00009626001	0,037	0,77284	0	GSPATT00029198001	0,416	0,04007	0
GSPATT00009643001	0,405	0,01362	0	GSPATT00029217001	0,222	0,14787	0
GSPATT00009645001	0,973	0,00151	0	GSPATT00029249001	-0,351	0,02712	0
GSPATT00009646001	0,589	0,00229	0	GSPATT00029273001	-2,046	0,00011	-1
GSPATT00009693001	0,347	0,0372	0	GSPATT00029295001	1,454	0,00404	1
GSPATT00009703001	-2,787	0	-1	GSPATT00029341001	0,262	0,14262	0
GSPATT00009718001	-0,819	0,00093	0	GSPATT00029366001	0,262	0,1057	0
GSPATT00009748001	0,253	0,1151	0	GSPATT00029406001	0,843	0,00341	0
GSPATT00009760001	-2,631	0	-1	GSPATT00029408001	0,85	0,0034	0
GSPATT00009772001	-0,026	0,84037	0	GSPATT00029420001	1,305	0,00003	1
GSPATT00009782001	-0,119	0,55451	0	GSPATT00029443001	-0,601	0,00289	0
GSPATT00009828001	0,419	0,03475	0	GSPATT00029444001	1,26	0,00076	1
GSPATT00009848001	-0,228	0,27077	0	GSPATT00029470001	0,093	0,49861	0
GSPATT00009873001	1,405	0,00002	1	GSPATT00029472001	0,184	0,20615	0
GSPATT00009883001	0,58	0,00174	0	GSPATT00029475001	0,432	0,06467	0
GSPATT00009888001	0,679	0,00449	0	GSPATT00029530001	0,081	0,70204	0
GSPATT00009892001	-0,164	0,50097	0	GSPATT00029552001	0,56	0,01662	0
GSPATT00009932001	-0,127	0,37952	0	GSPATT00029556001	-0,933	0,00515	0
GSPATT00009937001	0,505	0,12425	0	GSPATT00029558001	1,707	0,00001	1

GSPATT00009951001	-0,279	0,21687	0	GSPATT00029574001	-1,108	0,00039	-1
GSPATT00009964001	0,607	0,03286	0	GSPATT00029583001	0,198	0,35094	0
GSPATT00009969001	0,018	0,89309	0	GSPATT00029601001	1,502	0,00004	1
GSPATT00009980001	0,361	0,01933	0	GSPATT00029614001	-0,616	0,00563	0
GSPATT00009997001	0,66	0,0026	0	GSPATT00029618001	0,584	0,00422	0
GSPATT00010022001	-0,69	0,00171	0	GSPATT00029627001	-0,199	0,15139	0
GSPATT00010023001	0,56	0,00691	0	GSPATT00029631001	0,75	0,00323	0
GSPATT00010038001	0,125	0,38308	0	GSPATT00029668001	-0,047	0,74205	0
GSPATT00010040001	-0,369	0,07302	0	GSPATT00029687001	-0,415	0,0173	0
GSPATT00010043001	0,211	0,26138	0	GSPATT00029713001	0,817	0,00169	0
GSPATT00010044001	0,443	0,07115	0	GSPATT00029723001	0,372	0,11982	0
GSPATT00010047001	0,376	0,04978	0	GSPATT00029731001	0,613	0,0095	0
GSPATT00010069001	0,096	0,49349	0	GSPATT00029755001	0,726	0,00287	0
GSPATT00010072001	0,597	0,04414	0	GSPATT00029761001	0,264	0,06412	0
GSPATT00010088001	0,474	0,01739	0	GSPATT00029771001	-0,737	0,0067	0
GSPATT00010089001	-0,02	0,89788	0	GSPATT00029778001	-0,608	0,00307	0
GSPATT00010120001	0,439	0,05835	0	GSPATT00029822001	0,665	0,00641	0
GSPATT00010125001	0,294	0,08285	0	GSPATT00029823001	-0,53	0,02834	0
GSPATT00010132001	1,036	0,00021	1	GSPATT00029824001	0,784	0,05706	0
GSPATT00010165001	1,057	0,05472	0	GSPATT00029844001	0,11	0,52769	0
GSPATT00010186001	-0,625	0,00879	0	GSPATT00029848001	0,411	0,08951	0
GSPATT00010187001	0,077	0,70848	0	GSPATT00029901001	0,748	0,00121	0
GSPATT00010189001	0,274	0,09078	0	GSPATT00029929001	1,561	0,00012	1
GSPATT00010191001	-0,38	0,03269	0	GSPATT00029968001	0,19	0,2579	0
GSPATT00010192001	-0,341	0,04434	0	GSPATT00029971001	-0,01	0,95787	0
GSPATT00010201001	-1,112	0,00403	-1	GSPATT00030001001	-0,993	0,04787	0
GSPATT00010217001	0,055	0,68694	0	GSPATT00030006001	0,333	0,17202	0
GSPATT00010238001	0,721	0,00063	0	GSPATT00030011001	0,244	0,31877	0
GSPATT00010240001	1,641	0,00002	1	GSPATT00030033001	-0,25	0,39908	0
GSPATT00010241001	-0,059	0,67929	0	GSPATT00030052001	-0,257	0,11428	0
GSPATT00010256001	0,667	0,16041	0	GSPATT00030056001	0,096	0,60981	0
GSPATT00010264001	1,126	0,00022	1	GSPATT00030064001	1,558	0,00015	1
GSPATT00010267001	0,363	0,04122	0	GSPATT00030135001	0,579	0,06809	0
GSPATT00010305001	0,583	0,02695	0	GSPATT00030142001	0,973	0,0012	0
GSPATT00010342001	-0,027	0,88113	0	GSPATT00030146001	-0,644	0,00333	0
GSPATT00010360001	0,009	0,9481	0	GSPATT00030175001	-0,442	0,01694	0
GSPATT00010361001	1,344	0,00002	1	GSPATT00030183001	0,09	0,72161	0
GSPATT00010434001	0,716	0,00843	0	GSPATT00030220001	-0,307	0,04731	0
GSPATT00010455001	2,068	0,00001	1	GSPATT00030230001	0,126	0,43629	0
GSPATT00010465001	0,224	0,16678	0	GSPATT00030249001	1,907	0	1
GSPATT00010478001	0,755	0,0015	0	GSPATT00030250001	0,03	0,86055	0
GSPATT00010489001	0,856	0,01707	0	GSPATT00030251001	0,536	0,02526	0
GSPATT00010520001	1,675	0,00001	1	GSPATT00030268001	0,167	0,24397	0
GSPATT00010535001	0,291	0,15153	0	GSPATT00030295001	0,775	0,00158	0
GSPATT00010550001	-0,166	0,3917	0	GSPATT00030300001	0,307	0,11242	0
GSPATT00010557001	0,305	0,0402	0	GSPATT00030312001	0,159	0,34993	0
GSPATT00010582001	-0,301	0,06581	0	GSPATT00030338001	0,37	0,02763	0
GSPATT00010591001	-0,327	0,06596	0	GSPATT00030339001	0,929	0,00014	0
GSPATT00010597001	0,042	0,75702	0	GSPATT00030340001	-0,629	0,00193	0
GSPATT00010598001	0,186	0,32536	0	GSPATT00030347001	0,305	0,05631	0
GSPATT00010599001	-0,541	0,00596	0	GSPATT00030360001	1,11	0,00034	1
GSPATT00010606001	-0,148	0,37763	0	GSPATT00030377001	0,608	0,03137	0
GSPATT00010622001	0,288	0,09844	0	GSPATT00030395001	0,632	0,00363	0
GSPATT00010631001	-0,002	0,99061	0	GSPATT00030433001	0,649	0,01931	0
GSPATT00010634001	-0,099	0,64063	0	GSPATT00030441001	-1,166	0,00076	-1
GSPATT00010640001	0,662	0,03303	0	GSPATT00030461001	0,787	0,01769	0
GSPATT00010668001	0,532	0,00424	0	GSPATT00030462001	2,069	0	1
GSPATT00010700001	1,693	0	1	GSPATT00030463001	0,18	0,23178	0
GSPATT00010735001	0,406	0,06251	0	GSPATT00030486001	-1,516	0,00003	-1
GSPATT00010794001	0,388	0,14585	0	GSPATT00030525001	0,296	0,14492	0
GSPATT00010803001	0,375	0,05126	0	GSPATT00030537001	-0,36	0,14602	0
GSPATT00010807001	0,842	0,00505	0	GSPATT00030559001	1,24	0,00015	1
GSPATT00010834001	-0,748	0,00167	0	GSPATT00030602001	0,266	0,22578	0
GSPATT00010852001	0,595	0,00207	0	GSPATT00030614001	-0,182	0,32497	0
GSPATT00010915001	0,334	0,22897	0	GSPATT00030636001	0,66	0,0019	0
GSPATT00010926001	-0,295	0,08518	0	GSPATT00030654001	-0,73	0,00085	0
GSPATT00010927001	0,963	0,00093	0	GSPATT00030656001	0,805	0,00115	0

GSPATT00010930001	0,326	0,08283	0	GSPATT00030663001	0,245	0,35912	0
GSPATT00010931001	0,755	0,00409	0	GSPATT00030666001	0,411	0,06139	0
GSPATT00010951001	-0,4	0,08547	0	GSPATT00030669001	0,057	0,6915	0
GSPATT00010956001	-0,78	0,00146	0	GSPATT00030671001	-0,295	0,30814	0
GSPATT00010972001	-0,096	0,53691	0	GSPATT00030682001	-0,162	0,29594	0
GSPATT00010982001	1,537	0,00037	1	GSPATT00030687001	0,812	0,01438	0
GSPATT00010994001	-2,479	0	-1	GSPATT00030688001	-0,02	0,90163	0
GSPATT00011004001	0,073	0,58776	0	GSPATT00030698001	0,071	0,70437	0
GSPATT00011062001	-1,37	0,00003	-1	GSPATT00030708001	0,502	0,05646	0
GSPATT00011094001	-0,248	0,11654	0	GSPATT00030723001	-0,18	0,43523	0
GSPATT00011096001	0,27	0,14138	0	GSPATT00030733001	-1,617	0,00013	-1
GSPATT00011110001	0,439	0,02922	0	GSPATT00030745001	-0,1	0,48372	0
GSPATT00011111001	0,784	0,00245	0	GSPATT00030749001	-0,359	0,06814	0
GSPATT00011112001	0,611	0,00704	0	GSPATT00030782001	0,235	0,17113	0
GSPATT00011130001	0,933	0,00615	0	GSPATT00030803001	1,123	0,0001	1
GSPATT00011132001	-1,165	0,00035	-1	GSPATT00030806001	0,727	0,0015	0
GSPATT00011155001	0,816	0,00288	0	GSPATT00030818001	-0,245	0,23455	0
GSPATT00011167001	-0,396	0,02351	0	GSPATT00030826001	-2,147	0,00001	-1
GSPATT00011173001	-3,857	0	-1	GSPATT00030848001	0,031	0,86428	0
GSPATT00011179001	0,334	0,11641	0	GSPATT00030851001	0,263	0,1287	0
GSPATT00011220001	0,315	0,15075	0	GSPATT00030853001	0,549	0,03624	0
GSPATT00011221001	1,038	0,00031	1	GSPATT00030855001	1,1	0,00013	1
GSPATT00011227001	0,852	0,00196	0	GSPATT00030860001	1,314	0,00014	1
GSPATT00011241001	0,626	0,02469	0	GSPATT00030871001	-0,434	0,01131	0
GSPATT00011251001	0,282	0,09853	0	GSPATT00030877001	-0,793	0,0011	0
GSPATT00011290001	1,791	0	1	GSPATT00030894001	0,443	0,01543	0
GSPATT00011305001	-0,492	0,0392	0	GSPATT00030895001	0,613	0,00519	0
GSPATT00011317001	0,249	0,25263	0	GSPATT00030907001	0,89	0,00402	0
GSPATT00011340001	0,056	0,74423	0	GSPATT00030912001	1,631	0,00007	1
GSPATT00011369001	1,426	0,00002	1	GSPATT00030921001	0,509	0,01961	0
GSPATT00011371001	-0,116	0,58125	0	GSPATT00030945001	0,658	0,01733	0
GSPATT00011372001	-0,323	0,11988	0	GSPATT00030956001	-0,545	0,00308	0
GSPATT00011413001	0,314	0,1297	0	GSPATT00030971001	0,707	0,00637	0
GSPATT00011422001	-1,076	0,00614	-1	GSPATT00030979001	0,011	0,94131	0
GSPATT00011430001	0,323	0,07271	0	GSPATT00030994001	0,373	0,06118	0
GSPATT00011436001	0,608	0,01574	0	GSPATT00031006001	1,218	0,00004	1
GSPATT00011440001	0,123	0,57717	0	GSPATT00031028001	-0,522	0,00869	0
GSPATT00011454001	0,507	0,07282	0	GSPATT00031029001	-0,147	0,30646	0
GSPATT00011455001	-0,483	0,15659	0	GSPATT00031066001	-3,25	0	-1
GSPATT00011486001	-0,104	0,60315	0	GSPATT00031082001	0,807	0,00177	0
GSPATT00011499001	-0,148	0,29042	0	GSPATT00031097001	-0,034	0,81174	0
GSPATT00011515001	0,879	0,00666	0	GSPATT00031104001	-1,662	0,00021	-1
GSPATT00011546001	-0,929	0,00313	0	GSPATT00031107001	0,915	0,00188	0
GSPATT00011547001	0,964	0,00026	0	GSPATT00031110001	1,016	0,0098	1
GSPATT00011564001	0,676	0,00108	0	GSPATT00031155001	-0,356	0,05023	0
GSPATT00011565001	0,357	0,06272	0	GSPATT00031166001	0,279	0,06965	0
GSPATT00011566001	0,262	0,07738	0	GSPATT00031167001	0,272	0,08891	0
GSPATT00011580001	-0,02	0,90743	0	GSPATT00031168001	0,836	0,01523	0
GSPATT00011584001	0,283	0,12359	0	GSPATT00031178001	0,551	0,0218	0
GSPATT00011620001	0,245	0,09049	0	GSPATT00031182001	-0,535	0,01622	0
GSPATT00011622001	0,355	0,17874	0	GSPATT00031221001	-0,004	0,97942	0
GSPATT00011666001	-0,488	0,00499	0	GSPATT00031235001	0,643	0,00762	0
GSPATT00011685001	-0,399	0,05343	0	GSPATT00031280001	-0,052	0,69595	0
GSPATT00011700001	-0,131	0,51052	0	GSPATT00031285001	-0,236	0,21081	0
GSPATT00011714001	0,124	0,49457	0	GSPATT00031287001	0,155	0,35654	0
GSPATT00011716001	0,632	0,00185	0	GSPATT00031311001	0,32	0,0669	0
GSPATT00011729001	-0,066	0,66441	0	GSPATT00031336001	0,795	0,00047	0
GSPATT00011731001	0,353	0,05227	0	GSPATT00031426001	-1,033	0,00006	-1
GSPATT00011735001	1,538	0,00053	1	GSPATT00031441001	-0,048	0,82385	0
GSPATT00011739001	-0,466	0,04961	0	GSPATT00031454001	0,276	0,1339	0
GSPATT00011742001	1,365	0,00005	1	GSPATT00031471001	-0,423	0,28841	0
GSPATT00011764001	0,296	0,13873	0	GSPATT00031487001	0,99	0,002	0
GSPATT00011816001	-0,319	0,13633	0	GSPATT00031493001	0,611	0,02344	0
GSPATT00011825001	-0,485	0,08169	0	GSPATT00031509001	-0,798	0,00098	0
GSPATT00011826001	1,565	0,00133	1	GSPATT00031532001	1,665	0,00021	1
GSPATT00011840001	-0,798	0,00327	0	GSPATT00031546001	0,565	0,00846	0
GSPATT00011854001	0,553	0,02078	0	GSPATT00031569001	-0,125	0,54879	0

GSPATT00011856001	0,65	0,07665	0	GSPATT00031580001	1,114	0,00029	1
GSPATT00011857001	0,077	0,76623	0	GSPATT00031618001	0,323	0,2332	0
GSPATT00011864001	-0,091	0,49677	0	GSPATT00031622001	-0,037	0,78942	0
GSPATT00011887001	0,175	0,46958	0	GSPATT00031623001	-0,002	0,9946	0
GSPATT00011897001	0,61	0,00299	0	GSPATT00031630001	1,142	0,00103	1
GSPATT00011903001	0,087	0,58179	0	GSPATT00031642001	-1,737	0,00014	-1
GSPATT00011917001	0,417	0,15463	0	GSPATT00031643001	-1,25	0,00034	-1
GSPATT00011929001	-1,564	0,00004	-1	GSPATT00031645001	0,862	0,00026	0
GSPATT00011952001	0,118	0,38041	0	GSPATT00031653001	-0,225	0,14582	0
GSPATT00011968001	0,326	0,1663	0	GSPATT00031661001	0,548	0,02007	0
GSPATT00011981001	-0,032	0,83233	0	GSPATT00031665001	-0,001	0,99648	0
GSPATT00011982001	0,265	0,10595	0	GSPATT00031682001	-1,39	0,00003	-1
GSPATT00012043001	0,391	0,11161	0	GSPATT00031698001	-0,689	0,03351	0
GSPATT00012044001	-0,04	0,80131	0	GSPATT00031701001	-0,914	0,00444	0
GSPATT00012047001	-0,114	0,39986	0	GSPATT00031704001	1,254	0,00029	1
GSPATT00012075001	0,5	0,02536	0	GSPATT00031705001	1,81	0,00002	1
GSPATT00012141001	0,915	0,00115	0	GSPATT00031706001	-0,775	0,0011	0
GSPATT00012176001	0,545	0,02424	0	GSPATT00031738001	-1,034	0,00008	-1
GSPATT00012210001	-0,372	0,02535	0	GSPATT00031739001	0,382	0,19043	0
GSPATT00012216001	1,175	0,00014	1	GSPATT00031740001	0,355	0,05225	0
GSPATT00012223001	0,404	0,08406	0	GSPATT00031754001	-0,461	0,03297	0
GSPATT00012241001	0,159	0,5354	0	GSPATT00031756001	-0,463	0,03455	0
GSPATT00012255001	0,104	0,64586	0	GSPATT00031757001	0,716	0,03439	0
GSPATT00012262001	-1,15	0,00006	-1	GSPATT00031762001	-1,768	0,00003	-1
GSPATT00012269001	-0,185	0,39154	0	GSPATT00031768001	-0,037	0,82459	0
GSPATT00012278001	1,263	0,00039	1	GSPATT00031781001	-0,507	0,02156	0
GSPATT00012282001	0,837	0,01461	0	GSPATT00031782001	-0,24	0,42749	0
GSPATT00012286001	-0,79	0,00041	0	GSPATT00031797001	-0,011	0,9401	0
GSPATT00012322001	1,362	0,00005	1	GSPATT00031802001	0,907	0,00053	0
GSPATT00012339001	1,278	0,00002	1	GSPATT00031822001	0,463	0,05116	0
GSPATT00012348001	-1,134	0,00014	-1	GSPATT00031828001	-0,487	0,01743	0
GSPATT00012351001	0,299	0,06088	0	GSPATT00031846001	-0,183	0,21789	0
GSPATT00012371001	-0,352	0,29514	0	GSPATT00031865001	0,272	0,16899	0
GSPATT00012372001	0,15	0,484	0	GSPATT00031924001	0,545	0,00883	0
GSPATT00012374001	0,203	0,24495	0	GSPATT00031948001	-0,37	0,18789	0
GSPATT00012376001	0,959	0,00045	0	GSPATT00031955001	-0,129	0,4888	0
GSPATT00012379001	0,369	0,07668	0	GSPATT00031957001	0,03	0,87201	0
GSPATT00012395001	0,809	0,0075	0	GSPATT00032006001	0,087	0,67206	0
GSPATT00012396001	0,319	0,11515	0	GSPATT00032021001	0,096	0,48485	0
GSPATT00012400001	0,852	0,00032	0	GSPATT00032022001	-0,54	0,01533	0
GSPATT00012413001	0,837	0,00048	0	GSPATT00032029001	0,283	0,22374	0
GSPATT00012445001	0,429	0,03783	0	GSPATT00032030001	1,339	0,00008	1
GSPATT00012460001	0,238	0,14276	0	GSPATT00032037001	0,772	0,00865	0
GSPATT00012470001	-0,034	0,86348	0	GSPATT00032092001	0,508	0,09947	0
GSPATT00012488001	-0,04	0,75807	0	GSPATT00032094001	-2,851	0	-1
GSPATT00012520001	-2,03	0	-1	GSPATT00032095001	0,112	0,41059	0
GSPATT00012525001	0,13	0,44838	0	GSPATT00032100001	0,509	0,00574	0
GSPATT00012543001	0,86	0,00059	0	GSPATT00032104001	-0,024	0,9428	0
GSPATT00012570001	0,319	0,06017	0	GSPATT00032110001	-1,089	0,00427	-1
GSPATT00012610001	0,358	0,06157	0	GSPATT00032111001	-1,233	0,00541	-1
GSPATT00012628001	0,326	0,03065	0	GSPATT00032114001	0,44	0,01977	0
GSPATT00012631001	0,442	0,02953	0	GSPATT00032136001	-0,671	0,00209	0
GSPATT00012633001	0,282	0,10356	0	GSPATT00032166001	0,482	0,04639	0
GSPATT00012634001	0,634	0,00459	0	GSPATT00032169001	0,497	0,00936	0
GSPATT00012649001	-0,517	0,01272	0	GSPATT00032224001	0,067	0,66182	0
GSPATT00012656001	0,231	0,16634	0	GSPATT00032226001	0,466	0,04696	0
GSPATT00012666001	0,427	0,02248	0	GSPATT00032238001	0,364	0,06778	0
GSPATT00012684001	-0,036	0,83675	0	GSPATT00032247001	-0,428	0,012	0
GSPATT00012692001	0,375	0,08064	0	GSPATT00032286001	0,05	0,80461	0
GSPATT00012702001	0,193	0,34721	0	GSPATT00032288001	0,859	0,00547	0
GSPATT00012722001	0,574	0,01609	0	GSPATT00032289001	-0,097	0,49171	0
GSPATT00012732001	0,363	0,12773	0	GSPATT00032301001	-0,947	0,00029	0
GSPATT00012735001	0,149	0,25751	0	GSPATT00032341001	0,829	0,00058	0
GSPATT00012742001	1,227	0,00011	1	GSPATT00032372001	-0,241	0,09642	0
GSPATT00012746001	0,143	0,51196	0	GSPATT00032392001	0,313	0,05107	0
GSPATT00012753001	0,157	0,26935	0	GSPATT00032421001	-1,221	0,00022	-1
GSPATT00012754001	-0,119	0,5919	0	GSPATT00032429001	0,493	0,03216	0

GSPATT00012755001	1,516	0,00005	1	GSPATT00032432001	-0,292	0,20952	0
GSPATT00012756001	0,835	0,00358	0	GSPATT00032442001	0,198	0,2398	0
GSPATT00012822001	0,123	0,4374	0	GSPATT00032444001	-0,967	0,00044	0
GSPATT00012835001	0,596	0,00563	0	GSPATT00032458001	0,703	0,02409	0
GSPATT00012838001	0,001	0,99699	0	GSPATT00032459001	0,275	0,11268	0
GSPATT00012852001	1,275	0,00003	1	GSPATT00032460001	-0,526	0,00918	0
GSPATT00012853001	0,823	0,00197	0	GSPATT00032522001	-0,127	0,45119	0
GSPATT00012859001	-0,198	0,15153	0	GSPATT00032523001	0,812	0,00089	0
GSPATT00012868001	0,163	0,31281	0	GSPATT00032532001	0,103	0,45294	0
GSPATT00012875001	0,348	0,03129	0	GSPATT00032533001	-0,17	0,53723	0
GSPATT00012878001	0,169	0,34624	0	GSPATT00032558001	0,527	0,00289	0
GSPATT00012914001	0,157	0,26322	0	GSPATT00032559001	0,124	0,37615	0
GSPATT00012916001	-2,396	0	-1	GSPATT00032564001	1,204	0,00066	1
GSPATT00012931001	0,812	0,00089	0	GSPATT00032574001	1,413	0,00228	1
GSPATT00012968001	0,154	0,45108	0	GSPATT00032597001	0,884	0,00108	0
GSPATT00012973001	-0,403	0,04733	0	GSPATT00032618001	0,457	0,06613	0
GSPATT00012989001	0,026	0,8519	0	GSPATT00032619001	0,741	0,02826	0
GSPATT00012998001	-0,105	0,43684	0	GSPATT00032623001	-0,162	0,37167	0
GSPATT00013005001	-0,158	0,4174	0	GSPATT00032648001	0,509	0,00818	0
GSPATT00013045001	0,257	0,10688	0	GSPATT00032649001	0,305	0,06511	0
GSPATT00013048001	0,033	0,83799	0	GSPATT00032667001	1,812	0,00041	1
GSPATT00013062001	-0,119	0,40756	0	GSPATT00032670001	0,786	0,00243	0
GSPATT00013078001	0,087	0,54212	0	GSPATT00032697001	0,477	0,0138	0
GSPATT00013085001	1,595	0,00002	1	GSPATT00032706001	0,443	0,01419	0
GSPATT00013094001	0,81	0,00674	0	GSPATT00032739001	0,096	0,63648	0
GSPATT00013102001	0,769	0,00426	0	GSPATT00032755001	-0,448	0,01909	0
GSPATT00013110001	-1,076	0,00259	-1	GSPATT00032763001	-0,489	0,02531	0
GSPATT00013157001	0,235	0,17548	0	GSPATT00032783001	-0,567	0,00797	0
GSPATT00013159001	0,37	0,09003	0	GSPATT00032787001	-0,864	0,00059	0
GSPATT00013164001	-0,51	0,05354	0	GSPATT00032791001	0,581	0,0063	0
GSPATT00013165001	1,021	0,00106	1	GSPATT00032810001	0,795	0,00874	0
GSPATT00013173001	-0,295	0,08555	0	GSPATT00032894001	0,722	0,00361	0
GSPATT00013174001	0,011	0,93101	0	GSPATT00032910001	1,39	0,00003	1
GSPATT00013205001	0,52	0,00824	0	GSPATT00032920001	0,74	0,00144	0
GSPATT00013243001	-0,027	0,84517	0	GSPATT00032929001	0,137	0,64968	0
GSPATT00013253001	1,885	0,00001	1	GSPATT00032930001	1,256	0,00033	1
GSPATT00013260001	0,645	0,00484	0	GSPATT00032934001	0,256	0,2624	0
GSPATT00013263001	0,563	0,01505	0	GSPATT00032957001	0,434	0,02449	0
GSPATT00013271001	0,175	0,21491	0	GSPATT00032999001	0,68	0,00704	0
GSPATT00013272001	1,673	0,00001	1	GSPATT00033009001	-0,785	0,00143	0
GSPATT00013280001	-0,282	0,13129	0	GSPATT00033016001	0,224	0,20102	0
GSPATT00013281001	0,128	0,50204	0	GSPATT00033027001	0,378	0,03882	0
GSPATT00013308001	0,015	0,90603	0	GSPATT00033067001	0,968	0,00046	0
GSPATT00013315001	-0,188	0,20496	0	GSPATT00033072001	0,525	0,02731	0
GSPATT00013327001	-0,009	0,94794	0	GSPATT00033076001	0,219	0,41915	0
GSPATT00013343001	0,267	0,15492	0	GSPATT00033095001	-1,102	0,00064	-1
GSPATT00013356001	-0,722	0,00162	0	GSPATT00033106001	0,765	0,00138	0
GSPATT00013360001	-0,83	0,00139	0	GSPATT00033119001	-0,51	0,03205	0
GSPATT00013364001	-0,11	0,50767	0	GSPATT00033128001	-1,492	0,00008	-1
GSPATT00013383001	-1,314	0,0001	-1	GSPATT00033139001	0,432	0,01158	0
GSPATT00013411001	0,401	0,06647	0	GSPATT00033154001	1,026	0,00696	1
GSPATT00013453001	0,604	0,02056	0	GSPATT00033161001	-0,302	0,08205	0
GSPATT00013470001	0,177	0,28718	0	GSPATT00033165001	-0,046	0,8569	0
GSPATT00013490001	0,322	0,07055	0	GSPATT00033167001	-0,34	0,05638	0
GSPATT00013494001	0,052	0,75665	0	GSPATT00033198001	0,629	0,09101	0
GSPATT00013502001	-0,243	0,19365	0	GSPATT00033205001	0,673	0,00702	0
GSPATT00013552001	0,741	0,00042	0	GSPATT00033223001	-0,423	0,03978	0
GSPATT00013561001	0,271	0,16205	0	GSPATT00033237001	-0,283	0,16249	0
GSPATT00013578001	1,991	0,00001	1	GSPATT00033250001	-0,453	0,0188	0
GSPATT00013652001	0,191	0,18598	0	GSPATT00033258001	0,335	0,03868	0
GSPATT00013656001	0,171	0,2115	0	GSPATT00033260001	-0,16	0,55088	0
GSPATT00013680001	-0,238	0,41631	0	GSPATT00033274001	0,713	0,00057	0
GSPATT00013693001	0,493	0,01038	0	GSPATT00033288001	-0,334	0,19184	0
GSPATT00013695001	0,474	0,01357	0	GSPATT00033306001	-0,043	0,85073	0
GSPATT00013702001	-0,192	0,32187	0	GSPATT00033333001	0,576	0,00241	0
GSPATT00013754001	-3,054	0	-1	GSPATT00033334001	0,4	0,0313	0
GSPATT00013770001	-0,187	0,4332	0	GSPATT00033358001	-0,167	0,26712	0

GSPATT00013783001	1,026	0,01651	1	GSPATT00033399001	-0,126	0,62052	0
GSPATT00013790001	0,074	0,5863	0	GSPATT00033466001	-1,107	0,00106	-1
GSPATT00013801001	0,361	0,0755	0	GSPATT00033469001	0,884	0,00069	0
GSPATT00013825001	-0,769	0,00205	0	GSPATT00033477001	0,439	0,04845	0
GSPATT00013827001	0,481	0,00732	0	GSPATT00033494001	0,481	0,13408	0
GSPATT00013828001	0,419	0,03295	0	GSPATT00033495001	1,143	0,00006	1
GSPATT00013847001	0,139	0,40055	0	GSPATT00033505001	0,875	0,00023	0
GSPATT00013852001	-0,368	0,03397	0	GSPATT00033512001	-3,055	0	-1
GSPATT00013855001	0,438	0,04631	0	GSPATT00033523001	-1,294	0,00005	-1
GSPATT00013876001	1,319	0,00047	1	GSPATT00033534001	0,523	0,02677	0
GSPATT00013891001	0,176	0,26584	0	GSPATT00033537001	0,391	0,03807	0
GSPATT00013892001	-1,136	0,00035	-1	GSPATT00033539001	0,246	0,40993	0
GSPATT00013898001	0,162	0,43243	0	GSPATT00033568001	-0,204	0,16501	0
GSPATT00013900001	-0,003	0,98838	0	GSPATT00033573001	0,114	0,43633	0
GSPATT00013901001	0,247	0,35648	0	GSPATT00033586001	0,277	0,16433	0
GSPATT00013913001	1,633	0,00001	1	GSPATT00033606001	-0,328	0,25723	0
GSPATT00013928001	0,8	0,00105	0	GSPATT00033617001	0,273	0,25396	0
GSPATT00013961001	2,283	0,00001	1	GSPATT00033636001	0,336	0,04963	0
GSPATT00013977001	0,458	0,01806	0	GSPATT00033646001	-0,077	0,64536	0
GSPATT00013994001	1,578	0,00006	1	GSPATT00033676001	0,609	0,03214	0
GSPATT00014001001	0,554	0,00382	0	GSPATT00033682001	-0,181	0,30005	0
GSPATT00014008001	0,723	0,00107	0	GSPATT00033690001	-0,633	0,00229	0
GSPATT00014044001	0,335	0,03981	0	GSPATT00033727001	-0,264	0,20849	0
GSPATT00014052001	1,287	0,00418	1	GSPATT00033743001	0,187	0,34652	0
GSPATT00014072001	0,024	0,90147	0	GSPATT00033764001	1,303	0,00004	1
GSPATT00014093001	0,192	0,19217	0	GSPATT00033780001	1,385	0,00001	1
GSPATT00014104001	0,566	0,03625	0	GSPATT00033786001	0,824	0,01346	0
GSPATT00014124001	-1,671	0,00007	-1	GSPATT00033798001	0,882	0,00143	0
GSPATT00014126001	-0,673	0,00116	0	GSPATT00033801001	0,274	0,11977	0
GSPATT00014132001	2,015	0	1	GSPATT00033810001	-0,178	0,21774	0
GSPATT00014139001	0,906	0,02173	0	GSPATT00033811001	-0,727	0,00103	0
GSPATT00014152001	0,095	0,60893	0	GSPATT00033826001	0,54	0,01676	0
GSPATT00014158001	1,1	0,00099	1	GSPATT00033832001	1,004	0,0005	1
GSPATT00014190001	0,605	0,17114	0	GSPATT00033839001	0,232	0,20924	0
GSPATT00014191001	-0,567	0,0055	0	GSPATT00033861001	0,201	0,23013	0
GSPATT00014196001	-0,265	0,26029	0	GSPATT00033863001	0,989	0,00013	0
GSPATT00014197001	0,509	0,02553	0	GSPATT00033871001	0,751	0,00036	0
GSPATT00014213001	-0,445	0,00944	0	GSPATT00033877001	0,579	0,00323	0
GSPATT00014216001	0,08	0,66083	0	GSPATT00033881001	0,29	0,19304	0
GSPATT00014218001	0,813	0,0031	0	GSPATT00033904001	0,322	0,04932	0
GSPATT00014219001	0,639	0,00371	0	GSPATT00033912001	0,771	0,0063	0
GSPATT00014235001	0,358	0,02401	0	GSPATT00033917001	0,213	0,23333	0
GSPATT00014239001	0,559	0,04551	0	GSPATT00033938001	1,074	0,00021	1
GSPATT00014299001	0,536	0,03039	0	GSPATT00033979001	-0,385	0,04696	0
GSPATT00014310001	0,41	0,03511	0	GSPATT00033980001	0,087	0,60132	0
GSPATT00014391001	0,443	0,007	0	GSPATT00034005001	1,03	0,00044	1
GSPATT00014403001	0,293	0,20293	0	GSPATT00034018001	-1,92	0	-1
GSPATT00014411001	-0,004	0,97822	0	GSPATT00034024001	1,786	0,00003	1
GSPATT00014414001	0,567	0,00623	0	GSPATT00034063001	-0,183	0,27172	0
GSPATT00014433001	-0,31	0,10884	0	GSPATT00034071001	0,388	0,02595	0
GSPATT00014438001	0,123	0,39312	0	GSPATT00034075001	-0,137	0,30288	0
GSPATT00014454001	0,575	0,02293	0	GSPATT00034090001	0,737	0,00209	0
GSPATT00014461001	0,95	0,00019	0	GSPATT00034103001	-0,258	0,10215	0
GSPATT00014474001	-1,949	0	-1	GSPATT00034104001	0,705	0,00489	0
GSPATT00014478001	0,152	0,53003	0	GSPATT00034124001	-1,017	0,0022	-1
GSPATT00014529001	1,182	0,00018	1	GSPATT00034141001	0,708	0,00161	0
GSPATT00014568001	-0,37	0,05496	0	GSPATT00034156001	-0,057	0,72295	0
GSPATT00014587001	-0,019	0,88031	0	GSPATT00034171001	0,663	0,00966	0
GSPATT00014606001	0,487	0,04116	0	GSPATT00034175001	-0,329	0,08396	0
GSPATT00014629001	0,582	0,00301	0	GSPATT00034216001	0,528	0,01503	0
GSPATT00014641001	0,648	0,0036	0	GSPATT00034230001	0,16	0,26608	0
GSPATT00014645001	0,006	0,97144	0	GSPATT00034233001	0,369	0,08874	0
GSPATT00014654001	0,521	0,03824	0	GSPATT00034270001	1,032	0,00088	1
GSPATT00014660001	0,098	0,64509	0	GSPATT00034305001	0,927	0,0006	0
GSPATT00014673001	-0,235	0,2283	0	GSPATT00034308001	-0,353	0,08333	0
GSPATT00014674001	0,791	0,00595	0	GSPATT00034314001	-1,355	0,00021	-1
GSPATT00014676001	0,429	0,07671	0	GSPATT00034329001	1,249	0,00003	1

GSPATT00014685001	0,887	0,0008	0	GSPATT00034393001	-1,986	0,00002	-1
GSPATT00014692001	-0,278	0,14785	0	GSPATT00034405001	-0,584	0,00941	0
GSPATT00014705001	0,351	0,08889	0	GSPATT00034420001	-0,659	0,00959	0
GSPATT00014745001	0,289	0,08436	0	GSPATT00034421001	0,663	0,12721	0
GSPATT00014759001	1,203	0,00129	1	GSPATT00034447001	-0,601	0,07172	0
GSPATT00014793001	-0,201	0,47536	0	GSPATT00034463001	0,197	0,40106	0
GSPATT00014796001	1,387	0,00001	1	GSPATT00034473001	0,036	0,77796	0
GSPATT00014810001	0,609	0,01875	0	GSPATT00034536001	-0,738	0,00189	0
GSPATT00014822001	0,187	0,19063	0	GSPATT00034552001	1,257	0,00015	1
GSPATT00014841001	0,356	0,10216	0	GSPATT00034577001	-0,839	0,00058	0
GSPATT00014872001	0,244	0,12625	0	GSPATT00034596001	0,345	0,26159	0
GSPATT00014873001	0,194	0,50101	0	GSPATT00034603001	0,885	0,0034	0
GSPATT00014895001	0,711	0,00204	0	GSPATT00034615001	0,724	0,00057	0
GSPATT00014900001	0,37	0,05706	0	GSPATT00034623001	0,426	0,02613	0
GSPATT00014918001	-0,327	0,04948	0	GSPATT00034631001	0,527	0,0108	0
GSPATT00014943001	-0,826	0,00319	0	GSPATT00034632001	-0,156	0,30353	0
GSPATT00014958001	-0,462	0,20687	0	GSPATT00034637001	0,89	0,00286	0
GSPATT00014962001	-0,017	0,92935	0	GSPATT00034640001	1,246	0,00004	1
GSPATT00014964001	1,045	0,00154	1	GSPATT00034647001	0,189	0,18304	0
GSPATT00014966001	-0,912	0,0003	0	GSPATT00034648001	0,251	0,12628	0
GSPATT00014999001	0,415	0,07919	0	GSPATT00034657001	0,421	0,01754	0
GSPATT00015015001	0,687	0,00414	0	GSPATT00034667001	-0,213	0,27669	0
GSPATT00015017001	-1,086	0,00027	-1	GSPATT00034770001	0,27	0,13463	0
GSPATT00015018001	-0,08	0,70257	0	GSPATT00034806001	-0,248	0,17157	0
GSPATT00015021001	-1,722	0,00006	-1	GSPATT00034819001	0,638	0,01374	0
GSPATT00015033001	-0,23	0,21296	0	GSPATT00034827001	-0,31	0,07036	0
GSPATT00015042001	-0,895	0,00041	0	GSPATT00034860001	0,726	0,00775	0
GSPATT00015043001	0,268	0,46979	0	GSPATT00034862001	0,911	0,00088	0
GSPATT00015055001	0,271	0,36469	0	GSPATT00034863001	-2,801	0	-1
GSPATT00015057001	1,019	0,00048	1	GSPATT00034885001	0,254	0,14736	0
GSPATT00015058001	-0,19	0,26701	0	GSPATT00034888001	0,387	0,09919	0
GSPATT00015064001	0,376	0,08595	0	GSPATT00034893001	0,489	0,0257	0
GSPATT00015109001	-0,005	0,9692	0	GSPATT00034900001	0,251	0,34442	0
GSPATT00015151001	0,296	0,07883	0	GSPATT00034902001	-0,054	0,71793	0
GSPATT00015169001	0,432	0,17161	0	GSPATT00034911001	0,966	0,00178	0
GSPATT00015210001	1,87	0,00003	1	GSPATT00034927001	0,954	0,00128	0
GSPATT00015238001	1,322	0,00004	1	GSPATT00034954001	-0,254	0,17321	0
GSPATT00015246001	0,577	0,07312	0	GSPATT00034968001	0,919	0,00041	0
GSPATT00015257001	-0,315	0,32799	0	GSPATT00034975001	-0,694	0,00453	0
GSPATT00015261001	0,509	0,06899	0	GSPATT00034994001	0,228	0,11464	0
GSPATT00015299001	0,213	0,19618	0	GSPATT00034997001	0,566	0,00681	0
GSPATT00015301001	0,024	0,90708	0	GSPATT00035001001	0,72	0,00113	0
GSPATT00015311001	0,153	0,48604	0	GSPATT00035014001	0,215	0,22573	0
GSPATT00015312001	-0,675	0,00141	0	GSPATT00035015001	-0,804	0,00518	0
GSPATT00015325001	0,179	0,42392	0	GSPATT00035041001	0,7	0,01007	0
GSPATT00015335001	0,857	0,00098	0	GSPATT00035046001	-0,126	0,63021	0
GSPATT00015344001	0,355	0,08801	0	GSPATT00035048001	-0,034	0,8154	0
GSPATT00015351001	0,593	0,00674	0	GSPATT00035049001	1,06	0,00012	1
GSPATT00015356001	-0,33	0,11053	0	GSPATT00035050001	1,458	0,0075	1
GSPATT00015363001	-0,898	0,00028	0	GSPATT00035051001	1,15	0,00008	1
GSPATT00015373001	0,163	0,24261	0	GSPATT00035055001	-2,179	0	-1
GSPATT00015374001	0,211	0,22091	0	GSPATT00035058001	0,603	0,00388	0
GSPATT00015382001	1,396	0,00009	1	GSPATT00035098001	-0,426	0,01033	0
GSPATT00015396001	0,853	0,02037	0	GSPATT00035101001	0,274	0,13516	0
GSPATT00015413001	1,181	0,00207	1	GSPATT00035109001	0,17	0,21947	0
GSPATT00015428001	-0,166	0,38106	0	GSPATT00035113001	-3,146	0	-1
GSPATT00015448001	1,213	0,00004	1	GSPATT00035115001	0,815	0,01116	0
GSPATT00015474001	0,646	0,00966	0	GSPATT00035116001	0,615	0,00474	0
GSPATT00015475001	0,366	0,0282	0	GSPATT00035132001	0,541	0,03772	0
GSPATT00015479001	0,147	0,2715	0	GSPATT00035156001	0,909	0,02167	0
GSPATT00015588001	0,437	0,0437	0	GSPATT00035162001	0,462	0,02625	0
GSPATT00015597001	0,345	0,07945	0	GSPATT00035168001	-0,967	0,00127	0
GSPATT00015625001	-1,091	0,00006	-1	GSPATT00035170001	0,275	0,20192	0
GSPATT00015632001	1,616	0,00334	1	GSPATT00035186001	-0,111	0,58129	0
GSPATT00015635001	0,054	0,76188	0	GSPATT00035215001	-0,514	0,05916	0
GSPATT00015640001	0,853	0,00185	0	GSPATT00035219001	0,385	0,08349	0
GSPATT00015666001	-0,623	0,00761	0	GSPATT00035221001	1,144	0,00247	1

GSPATT00015671001	-0,147	0,30793	0	GSPATT00035230001	0,202	0,239	0
GSPATT00015678001	0,121	0,52246	0	GSPATT00035231001	0,708	0,00161	0
GSPATT00015687001	0,716	0,00115	0	GSPATT00035242001	0,289	0,32037	0
GSPATT00015690001	0,723	0,00416	0	GSPATT00035265001	-0,98	0,00136	0
GSPATT00015693001	-0,397	0,09858	0	GSPATT00035273001	0,212	0,15905	0
GSPATT00015702001	0,703	0,00191	0	GSPATT00035283001	0,586	0,00587	0
GSPATT00015703001	0,498	0,0388	0	GSPATT00035344001	1,515	0,00002	1
GSPATT00015729001	0,223	0,18041	0	GSPATT00035345001	0,075	0,59803	0
GSPATT00015736001	0,804	0,00049	0	GSPATT00035369001	1,788	0	1
GSPATT00015750001	1,122	0,00018	1	GSPATT00035370001	0,774	0,00252	0
GSPATT00015756001	0,261	0,13282	0	GSPATT00035400001	0,193	0,51965	0
GSPATT00015783001	0,994	0,00013	0	GSPATT00035435001	0,252	0,14704	0
GSPATT00015804001	-0,028	0,89234	0	GSPATT00035439001	0,245	0,18477	0
GSPATT00015821001	-0,236	0,16594	0	GSPATT00035512001	0,145	0,51374	0
GSPATT00015826001	-1,025	0,00242	-1	GSPATT00035537001	-0,316	0,13551	0
GSPATT00015830001	2,01	0,00006	1	GSPATT00035553001	0,19	0,42969	0
GSPATT00015840001	-0,032	0,85759	0	GSPATT00035558001	0,435	0,06307	0
GSPATT00015852001	0,793	0,01264	0	GSPATT00035563001	0,506	0,20376	0
GSPATT00015854001	-0,969	0,00154	0	GSPATT00035570001	0,438	0,14386	0
GSPATT00015864001	0,541	0,09018	0	GSPATT00035586001	0,216	0,24577	0
GSPATT00015877001	-0,291	0,21366	0	GSPATT00035588001	1,375	0,00072	1
GSPATT00015881001	0,435	0,06228	0	GSPATT00035590001	-0,098	0,61368	0
GSPATT00015890001	0,521	0,0327	0	GSPATT00035613001	0,93	0,00031	0
GSPATT00015896001	0,187	0,19576	0	GSPATT00035630001	0,599	0,00235	0
GSPATT00015906001	-0,376	0,07667	0	GSPATT00035632001	1,647	0,00001	1
GSPATT00015913001	-3,714	0	-1	GSPATT00035666001	0,319	0,04691	0
GSPATT00015946001	-0,176	0,47867	0	GSPATT00035684001	0,079	0,61168	0
GSPATT00015989001	0,756	0,0358	0	GSPATT00035689001	-0,122	0,45197	0
GSPATT00015997001	0,616	0,05511	0	GSPATT00035711001	-0,148	0,32112	0
GSPATT00015998001	0,057	0,70953	0	GSPATT00035735001	-0,093	0,61289	0
GSPATT00016007001	-1,006	0,00099	-1	GSPATT00035742001	-0,345	0,03219	0
GSPATT00016022001	0,297	0,04559	0	GSPATT00035743001	-0,119	0,44406	0
GSPATT00016031001	1,983	0	1	GSPATT00035748001	0,686	0,00459	0
GSPATT00016054001	-0,06	0,68875	0	GSPATT00035791001	-0,365	0,03388	0
GSPATT00016058001	0,403	0,0261	0	GSPATT00035804001	1,246	0,00002	1
GSPATT00016068001	0,584	0,00865	0	GSPATT00035808001	-0,036	0,8091	0
GSPATT00016075001	0,476	0,01419	0	GSPATT00035812001	0,025	0,89409	0
GSPATT00016078001	-0,46	0,01198	0	GSPATT00035830001	0,647	0,0186	0
GSPATT00016099001	-0,376	0,0569	0	GSPATT00035834001	0,834	0,00201	0
GSPATT00016118001	0,681	0,00081	0	GSPATT00035841001	-0,305	0,05898	0
GSPATT00016152001	0,24	0,11484	0	GSPATT00035845001	0,903	0,0002	0
GSPATT00016163001	0,936	0,00075	0	GSPATT00035846001	-0,018	0,91105	0
GSPATT00016186001	0,651	0,00328	0	GSPATT00035878001	-0,682	0,01003	0
GSPATT00016201001	0,264	0,12078	0	GSPATT00035892001	0,871	0,00133	0
GSPATT00016202001	0,942	0,0002	0	GSPATT00035943001	1,226	0,00006	1
GSPATT00016224001	0,577	0,00541	0	GSPATT00035967001	-1,028	0,00178	-1
GSPATT00016246001	1,272	0,00554	1	GSPATT00035984001	0,033	0,81622	0
GSPATT00016252001	-1,875	0,00002	-1	GSPATT00035987001	0,061	0,629	0
GSPATT00016286001	0,878	0,0129	0	GSPATT00035988001	0,121	0,62093	0
GSPATT00016302001	-0,243	0,11044	0	GSPATT00035996001	1,583	0,00009	1
GSPATT00016304001	0,003	0,98948	0	GSPATT00036001001	0,324	0,09202	0
GSPATT00016323001	0,645	0,02514	0	GSPATT00036020001	0,633	0,01487	0
GSPATT00016324001	0,417	0,15142	0	GSPATT00036021001	-0,784	0,08263	0
GSPATT00016354001	1,499	0,00002	1	GSPATT00036025001	-0,396	0,01622	0
GSPATT00016370001	0,574	0,02089	0	GSPATT00036029001	0,388	0,03177	0
GSPATT00016374001	0,711	0,02701	0	GSPATT00036032001	-0,747	0,00511	0
GSPATT00016390001	-0,025	0,88764	0	GSPATT00036035001	1,064	0,00084	1
GSPATT00016393001	0,939	0,0007	0	GSPATT00036037001	0,29	0,15131	0
GSPATT00016396001	-0,359	0,34319	0	GSPATT00036059001	-2,602	0,00001	-1
GSPATT00016433001	0,343	0,0523	0	GSPATT00036080001	-0,995	0,00114	0
GSPATT00016451001	0,509	0,00351	0	GSPATT00036130001	1,008	0,00009	1
GSPATT00016461001	2,349	0	1	GSPATT00036133001	0,425	0,08763	0
GSPATT00016467001	0,07	0,6374	0	GSPATT00036142001	0,347	0,06142	0
GSPATT00016473001	-0,231	0,19236	0	GSPATT00036146001	-0,276	0,13155	0
GSPATT00016474001	-0,225	0,18313	0	GSPATT00036182001	0,139	0,41765	0
GSPATT00016477001	0,581	0,00731	0	GSPATT00036198001	0,154	0,39188	0
GSPATT00016484001	0,021	0,92665	0	GSPATT00036212001	0,53	0,00883	0

GSPATT00016487001	0,393	0,10186	0	GSPATT00036217001	-0,481	0,07457	0
GSPATT00016492001	1,714	0,00007	1	GSPATT00036238001	0,234	0,24062	0
GSPATT00016497001	0,83	0,00243	0	GSPATT00036250001	0,42	0,01334	0
GSPATT00016499001	2,913	0	1	GSPATT00036252001	-0,676	0,01753	0
GSPATT00016501001	0,408	0,05581	0	GSPATT00036259001	-0,145	0,42835	0
GSPATT00016508001	-0,185	0,31276	0	GSPATT00036286001	-0,683	0,00156	0
GSPATT00016522001	1,486	0,00001	1	GSPATT00036336001	0,222	0,12243	0
GSPATT00016536001	0,338	0,11356	0	GSPATT00036337001	0,687	0,00442	0
GSPATT00016547001	-0,209	0,18719	0	GSPATT00036353001	0,737	0,00133	0
GSPATT00016555001	0,08	0,62165	0	GSPATT00036372001	-0,154	0,42691	0
GSPATT00016572001	0,158	0,27397	0	GSPATT00036394001	-0,422	0,06663	0
GSPATT00016579001	-0,042	0,75571	0	GSPATT00036395001	0,718	0,00113	0
GSPATT00016580001	1,975	0	1	GSPATT00036407001	0,356	0,04968	0
GSPATT00016581001	0,583	0,00498	0	GSPATT00036409001	0,316	0,04048	0
GSPATT00016584001	0,48	0,00759	0	GSPATT00036434001	0,218	0,17014	0
GSPATT00016598001	-0,315	0,17406	0	GSPATT00036444001	-0,56	0,00414	0
GSPATT00016621001	-0,635	0,00167	0	GSPATT00036445001	0,343	0,02548	0
GSPATT00016625001	0,578	0,01915	0	GSPATT00036459001	1,229	0,0001	1
GSPATT00016626001	-1,01	0,01795	-1	GSPATT00036469001	0,587	0,01017	0
GSPATT00016635001	1,276	0,00002	1	GSPATT00036471001	0,051	0,77388	0
GSPATT00016642001	0,83	0,00038	0	GSPATT00036474001	0,054	0,83167	0
GSPATT00016644001	-0,085	0,60496	0	GSPATT00036479001	-0,35	0,11614	0
GSPATT00016649001	0,549	0,0028	0	GSPATT00036483001	-0,011	0,94317	0
GSPATT00016655001	-0,315	0,1207	0	GSPATT00036486001	-0,301	0,19234	0
GSPATT00016684001	0,371	0,03571	0	GSPATT00036520001	1,333	0,00049	1
GSPATT00016686001	-0,649	0,0057	0	GSPATT00036521001	-1,453	0,00724	-1
GSPATT00016687001	-1,172	0,00022	-1	GSPATT00036542001	2,353	0	1
GSPATT00016707001	0,035	0,81827	0	GSPATT00036543001	3,458	0	1
GSPATT00016715001	-0,125	0,40578	0	GSPATT00036578001	0,012	0,94123	0
GSPATT00016728001	0,796	0,00369	0	GSPATT00036602001	-0,016	0,91827	0
GSPATT00016739001	0,05	0,81698	0	GSPATT00036604001	0,849	0,00077	0
GSPATT00016753001	0,303	0,03871	0	GSPATT00036632001	0,404	0,0432	0
GSPATT00016756001	0,551	0,01981	0	GSPATT00036666001	-0,42	0,03381	0
GSPATT00016834001	-0,177	0,2236	0	GSPATT00036671001	-0,905	0,00646	0
GSPATT00016841001	0,199	0,27702	0	GSPATT00036678001	0,014	0,9207	0
GSPATT00016868001	-0,668	0,00534	0	GSPATT00036688001	0,31	0,04238	0
GSPATT00016887001	-0,259	0,12658	0	GSPATT00036690001	0,725	0,00628	0
GSPATT00016901001	-0,918	0,00013	0	GSPATT00036705001	1,156	0,00008	1
GSPATT00016943001	-0,089	0,60758	0	GSPATT00036706001	0,883	0,00746	0
GSPATT00016964001	0,04	0,87452	0	GSPATT00036730001	-0,04	0,79056	0
GSPATT00016974001	-0,017	0,9121	0	GSPATT00036752001	0,349	0,08868	0
GSPATT00016976001	1,509	0,00004	1	GSPATT00036770001	0,189	0,34256	0
GSPATT00016985001	0,32	0,1398	0	GSPATT00036776001	-0,3	0,06534	0
GSPATT00017038001	2	0	1	GSPATT00036800001	-0,04	0,79823	0
GSPATT00017050001	0,473	0,00736	0	GSPATT00036806001	-0,03	0,88012	0
GSPATT00017063001	0,061	0,69591	0	GSPATT00036817001	-0,834	0,01188	0
GSPATT00017094001	0,37	0,12392	0	GSPATT00036862001	-1,618	0,00012	-1
GSPATT00017107001	0,512	0,01311	0	GSPATT00036876001	0,655	0,01693	0
GSPATT00017117001	1,483	0,00007	1	GSPATT00036879001	0,685	0,00059	0
GSPATT00017119001	0,567	0,00522	0	GSPATT00036888001	0,452	0,00944	0
GSPATT00017122001	-0,89	0,00112	0	GSPATT00036914001	0,754	0,10066	0
GSPATT00017148001	0,445	0,01161	0	GSPATT00036919001	-0,269	0,14769	0
GSPATT00017157001	0,228	0,29231	0	GSPATT00036925001	0,498	0,00721	0
GSPATT00017193001	0,423	0,02362	0	GSPATT00036954001	-0,592	0,0063	0
GSPATT00017227001	0,713	0,01736	0	GSPATT00036971001	-0,218	0,16152	0
GSPATT00017228001	-1,143	0,00016	-1	GSPATT00037000001	0,283	0,14601	0
GSPATT00017248001	0,004	0,98061	0	GSPATT00037053001	-0,135	0,29881	0
GSPATT00017265001	0,224	0,27585	0	GSPATT00037058001	0,084	0,66195	0
GSPATT00017266001	0,228	0,22183	0	GSPATT00037066001	-0,414	0,01584	0
GSPATT00017287001	0,722	0,00261	0	GSPATT00037088001	0,441	0,01862	0
GSPATT00017341001	-0,483	0,07425	0	GSPATT00037094001	-0,008	0,9585	0
GSPATT00017350001	-0,237	0,16156	0	GSPATT00037099001	0,395	0,03767	0
GSPATT00017354001	-0,088	0,60425	0	GSPATT00037123001	0,774	0,01287	0
GSPATT00017356001	1,623	0,00011	1	GSPATT00037129001	0,656	0,00104	0
GSPATT00017361001	0,54	0,0029	0	GSPATT00037143001	-0,027	0,85661	0
GSPATT00017388001	-0,64	0,00112	0	GSPATT00037145001	0,616	0,02922	0
GSPATT00017455001	0,77	0,0007	0	GSPATT00037148001	-0,358	0,09184	0

GSPATT00017456001	0,739	0,00754	0	GSPATT00037154001	-0,557	0,01543	0
GSPATT00017488001	0,346	0,03561	0	GSPATT00037166001	1	0,00065	0
GSPATT00017492001	0,873	0,0003	0	GSPATT00037168001	0,397	0,02983	0
GSPATT00017520001	-0,5	0,016	0	GSPATT00037172001	0,145	0,33707	0
GSPATT00017543001	1,645	0,00001	1	GSPATT00037175001	0,205	0,26106	0
GSPATT00017557001	0,172	0,32619	0	GSPATT00037211001	0,931	0,00079	0
GSPATT00017560001	1,038	0,00731	1	GSPATT00037212001	0,689	0,00673	0
GSPATT00017576001	1,079	0,00068	1	GSPATT00037226001	0,202	0,30966	0
GSPATT00017619001	-0,144	0,3289	0	GSPATT00037232001	0,667	0,00174	0
GSPATT00017630001	-0,188	0,2095	0	GSPATT00037285001	0,579	0,00719	0
GSPATT00017662001	0,243	0,10339	0	GSPATT00037291001	-2,613	0,00006	-1
GSPATT00017665001	0,463	0,01095	0	GSPATT00037311001	-0,268	0,09834	0
GSPATT00017666001	0,164	0,29665	0	GSPATT00037328001	0,772	0,00301	0
GSPATT00017671001	1,215	0,00163	1	GSPATT00037330001	0,474	0,01266	0
GSPATT00017672001	0,666	0,00486	0	GSPATT00037368001	1,031	0,0019	1
GSPATT00017676001	0,897	0,00014	0	GSPATT00037376001	0,236	0,20339	0
GSPATT00017680001	1,263	0,0002	1	GSPATT00037398001	0,097	0,63269	0
GSPATT00017681001	1,015	0,00199	1	GSPATT00037444001	-0,662	0,00643	0
GSPATT00017692001	0,757	0,02894	0	GSPATT00037451001	0,414	0,01229	0
GSPATT00017710001	0,784	0,00357	0	GSPATT00037461001	0,206	0,15823	0
GSPATT00017712001	-0,359	0,17781	0	GSPATT00037466001	0,211	0,25267	0
GSPATT00017715001	1,019	0,00017	1	GSPATT00037469001	0,099	0,53472	0
GSPATT00017746001	0,26	0,0958	0	GSPATT00037471001	0,104	0,58331	0
GSPATT00017766001	-1,364	0,00026	-1	GSPATT00037488001	1,394	0,00067	1
GSPATT00017771001	0,257	0,1542	0	GSPATT00037523001	0,723	0,01928	0
GSPATT00017791001	-1,005	0,00013	-1	GSPATT00037526001	0,897	0,00043	0
GSPATT00017820001	0,089	0,63426	0	GSPATT00037566001	-0,05	0,76612	0
GSPATT00017833001	1,804	0,00001	1	GSPATT00037597001	-0,102	0,55503	0
GSPATT00017845001	-0,337	0,04954	0	GSPATT00037660001	0,186	0,17268	0
GSPATT00017866001	-0,949	0,00081	0	GSPATT00037661001	0,066	0,71158	0
GSPATT00017868001	-0,188	0,4063	0	GSPATT00037688001	0,303	0,04893	0
GSPATT00017882001	-0,011	0,94767	0	GSPATT00037699001	0,741	0,00075	0
GSPATT00017902001	-0,048	0,73859	0	GSPATT00037711001	-0,444	0,05547	0
GSPATT00017904001	0,196	0,20412	0	GSPATT00037715001	0,109	0,42522	0
GSPATT00017915001	0,763	0,00829	0	GSPATT00037722001	0,821	0,0005	0
GSPATT00017926001	-0,085	0,54936	0	GSPATT00037727001	0,543	0,04234	0
GSPATT00017952001	0,822	0,00042	0	GSPATT00037737001	1,364	0,0001	1
GSPATT00017953001	0,93	0,00178	0	GSPATT00037754001	-0,856	0,0023	0
GSPATT00017960001	-0,674	0,00313	0	GSPATT00037781001	-1,255	0,00102	-1
GSPATT00017963001	-0,275	0,06466	0	GSPATT00037784001	0,072	0,57853	0
GSPATT00017966001	0,227	0,14352	0	GSPATT00037789001	2,068	0	1
GSPATT00017972001	-0,131	0,38508	0	GSPATT00037792001	-0,369	0,18448	0
GSPATT00017992001	-0,11	0,50579	0	GSPATT00037795001	0,362	0,01982	0
GSPATT00018004001	0,463	0,03497	0	GSPATT00037796001	1,094	0,0001	1
GSPATT00018048001	0,735	0,00074	0	GSPATT00037798001	0,035	0,81328	0
GSPATT00018049001	0,948	0,00013	0	GSPATT00037799001	-0,61	0,0081	0
GSPATT00018051001	-0,539	0,05915	0	GSPATT00037811001	0,21	0,2921	0
GSPATT00018101001	0,898	0,00614	0	GSPATT00037815001	0,925	0,00331	0
GSPATT00018127001	0,153	0,36392	0	GSPATT00037818001	0,928	0,00072	0
GSPATT00018132001	0,741	0,02921	0	GSPATT00037856001	0,303	0,06934	0
GSPATT00018133001	-0,081	0,66594	0	GSPATT00037875001	-0,066	0,66305	0
GSPATT00018143001	0,268	0,2351	0	GSPATT00037876001	0,446	0,01048	0
GSPATT00018153001	-0,086	0,72806	0	GSPATT00037938001	0,146	0,5361	0
GSPATT00018163001	0,342	0,02849	0	GSPATT00037939001	0,394	0,02006	0
GSPATT00018188001	0,221	0,21776	0	GSPATT00037945001	-0,437	0,02189	0
GSPATT00018202001	0,66	0,01285	0	GSPATT00037967001	-0,06	0,6776	0
GSPATT00018223001	-0,409	0,02988	0	GSPATT00037978001	0,461	0,08571	0
GSPATT00018248001	-0,041	0,77438	0	GSPATT00038025001	-0,104	0,48304	0
GSPATT00018263001	0,814	0,00153	0	GSPATT00038027001	0,502	0,05619	0
GSPATT00018280001	0,316	0,0745	0	GSPATT00038035001	0,888	0,00025	0
GSPATT00018290001	-0,359	0,04447	0	GSPATT00038045001	-0,396	0,05929	0
GSPATT00018291001	0,141	0,45858	0	GSPATT00038090001	1,233	0,00044	1
GSPATT00018293001	0,169	0,30306	0	GSPATT00038149001	0,258	0,07307	0
GSPATT00018330001	0,511	0,01	0	GSPATT00038155001	0,75	0,00768	0
GSPATT00018389001	-0,094	0,58557	0	GSPATT00038156001	0,107	0,61347	0
GSPATT00018393001	-0,203	0,16787	0	GSPATT00038162001	-0,205	0,24742	0
GSPATT00018398001	0,481	0,09586	0	GSPATT00038171001	-1,199	0,00032	-1

GSPATT00018431001	0,56	0,04386	0
GSPATT00018438001	0,545	0,01322	0
GSPATT00018449001	0,58	0,0723	0
GSPATT00018471001	-1,113	0,00027	-1
GSPATT00018476001	0,992	0,00041	0
GSPATT00018515001	0,206	0,16244	0
GSPATT00018529001	0,92	0,01084	0
GSPATT00018559001	0,48	0,01489	0
GSPATT00018563001	0,407	0,05649	0
GSPATT00018600001	0,51	0,01239	0
GSPATT00018603001	-0,883	0,002	0
GSPATT00018608001	0,219	0,14898	0
GSPATT00018634001	-0,253	0,0798	0
GSPATT00018635001	-4,269	0	-1
GSPATT00018645001	-0,232	0,15845	0
GSPATT00018660001	-0,45	0,12247	0
GSPATT00018666001	0,125	0,44695	0
GSPATT00018671001	0,675	0,00088	0
GSPATT00018681001	-0,683	0,00496	0
GSPATT00018695001	-0,076	0,62948	0
GSPATT00018702001	-0,494	0,02855	0
GSPATT00018706001	0,301	0,2524	0
GSPATT00018710001	-0,217	0,14626	0
GSPATT00018727001	-0,029	0,84109	0
GSPATT00018728001	-0,429	0,04057	0
GSPATT00018737001	0,112	0,42429	0
GSPATT00018784001	0,107	0,56227	0
GSPATT00018806001	-0,089	0,50827	0
GSPATT00018878001	-0,526	0,01436	0
GSPATT00018891001	-0,275	0,12204	0
GSPATT00018918001	0,815	0,00108	0
GSPATT00018920001	-0,177	0,49925	0
GSPATT00018943001	-0,344	0,12601	0
GSPATT00018950001	0,098	0,47385	0
GSPATT00018952001	0,199	0,36354	0
GSPATT00018955001	-1,697	0,00048	-1
GSPATT00018964001	0,792	0,02949	0
GSPATT00018965001	0,578	0,00897	0
GSPATT00018969001	-0,459	0,03222	0
GSPATT00018978001	1,009	0,00294	1
GSPATT00019012001	0,241	0,25273	0
GSPATT00019047001	-0,313	0,12451	0
GSPATT00019066001	0,738	0,00884	0
GSPATT00019068001	0,352	0,08916	0
GSPATT00019089001	-0,092	0,50555	0
GSPATT00019098001	-0,396	0,01375	0
GSPATT00019105001	-0,564	0,03875	0
GSPATT00019121001	-0,238	0,13986	0
GSPATT00019140001	-0,119	0,39295	0
GSPATT00019142001	0,119	0,67944	0
GSPATT00019147001	1,334	0,00037	1
GSPATT00019169001	-0,494	0,02586	0
GSPATT00019217001	0,697	0,00662	0
GSPATT00019232001	1,248	0,0003	1
GSPATT00019271001	0,448	0,01195	0
GSPATT00019272001	0,626	0,00167	0
GSPATT00019281001	0,242	0,15235	0
GSPATT00019357001	-0,269	0,11687	0
GSPATT00019361001	1,018	0,00339	1
GSPATT00019366001	0,029	0,8276	0
GSPATT00019371001	-1,072	0,00005	-1
GSPATT00019421001	0,515	0,01534	0
GSPATT00019446001	0,948	0,01336	0
GSPATT00019447001	1,328	0,00111	1
GSPATT00019448001	1,274	0,00003	1
GSPATT00019460001	0,582	0,01671	0
GSPATT00019477001	-0,546	0,02587	0
GSPATT00038175001	-0,718	0,00151	0
GSPATT00038196001	0,025	0,86468	0
GSPATT00038198001	1,751	0,00019	1
GSPATT00038202001	1,049	0,00014	1
GSPATT00038203001	1,279	0,00007	1
GSPATT00038212001	0,313	0,14446	0
GSPATT00038214001	2,959	0	1
GSPATT00038217001	1,138	0,00032	1
GSPATT00038232001	-1,557	0,00002	-1
GSPATT00038377001	1,241	0,00049	1
GSPATT00038378001	0,101	0,52246	0
GSPATT00038439001	0,034	0,86173	0
GSPATT00038442001	0,573	0,00219	0
GSPATT00038453001	0,958	0,00056	0
GSPATT00038457001	0,034	0,80944	0
GSPATT00038462001	0,25	0,17848	0
GSPATT00038469001	1,122	0,00009	1
GSPATT00038489001	0,49	0,00846	0
GSPATT00038497001	0,218	0,14041	0
GSPATT00038500001	-0,057	0,70837	0
GSPATT00038501001	0,115	0,39982	0
GSPATT00038509001	0,207	0,13138	0
GSPATT00038517001	0,388	0,06212	0
GSPATT00038525001	0,766	0,00172	0
GSPATT00038527001	0,262	0,08764	0
GSPATT00038565001	-0,503	0,00816	0
GSPATT00038594001	-0,531	0,01391	0
GSPATT00038759001	0,112	0,58404	0
GSPATT00038767001	0,906	0,00064	0
GSPATT00038772001	0,189	0,25284	0
GSPATT00038778001	0,45	0,0114	0
GSPATT00038811001	0,163	0,23056	0
GSPATT00038843001	0,394	0,08578	0
GSPATT00038849001	0,132	0,34446	0
GSPATT00038851001	-0,622	0,0036	0
GSPATT00038853001	0,357	0,03403	0
GSPATT00038854001	0,067	0,67394	0
GSPATT00038872001	-0,347	0,10873	0
GSPATT00038895001	-2,984	0,00003	-1
GSPATT00038896001	-0,151	0,65702	0
GSPATT00038914001	1,241	0,00033	1
GSPATT00038932001	0,144	0,35304	0
GSPATT00038938001	0,67	0,06631	0
GSPATT00039020001	1,009	0,00006	1
GSPATT00039058001	0,227	0,19888	0
GSPATT00039143001	0,942	0,00145	0
GSPATT00039147001	-0,456	0,10233	0
GSPATT00039260001	0,476	0,01736	0
GSPATT00039305001	0,494	0,01965	0
GSPATT00039325001	0,34	0,08666	0
GSPATT00039337001	0,067	0,62405	0
GSPATT00039369001	-0,01	0,94511	0
GSPATT00039396001	-0,215	0,15116	0
GSPATT00039397001	0,806	0,00031	0
GSPATT00039403001	1,013	0,00053	1
GSPATT00039440001	-0,449	0,0779	0
GSPATT00039502001	-0,434	0,08958	0
GSPATT00039519001	0,599	0,01071	0
GSPATT00039578001	1,071	0,0029	1
GSPATT00039596001	-0,065	0,63218	0
GSPATT00039606001	-0,083	0,73346	0
GSPATT00039607001	0,689	0,00107	0
GSPATT00039617001	-0,125	0,38541	0
GSPATT00039623001	-0,179	0,37866	0
GSPATT00039672001	0,81	0,01218	0
GSPATT00039701001	0,824	0,06378	0
GSPATT00039733001	0,707	0,00233	0

GSPATT00019493001	-0,409	0,16665	0	GSPATT00039825001	0,881	0,00181	0
GSPATT00019504001	0,507	0,03568	0	PTETT10500005001	0,689	0,01507	0
GSPATT00019520001	1,19	0,00006	1	PTETT10800002001	-0,131	0,51208	0
GSPATT00019546001	0,939	0,00501	0				
GSPATT00019578001	0,845	0,00108	0				

GO 0010468 regulation of gene expression

ID	coeff.	p-value	signif.	ID	coeff.	p-value	signif.
GSPATT00000430001	0,805	0,00385	0	GSPATT00022877001	0,756	0,0172	0
GSPATT00000588001	0,624	0,07319	0	GSPATT00022959001	1,261	0,00015	1
GSPATT00000689001	0,677	0,00237	0	GSPATT00022980001	0,614	0,0183	0
GSPATT00000920001	0,161	0,28196	0	GSPATT00023028001	-1,34	0,00001	-1
GSPATT00001199001	-0,4	0,07351	0	GSPATT00023176001	-0,179	0,37643	0
GSPATT00001224001	-0,004	0,97956	0	GSPATT00023182001	-0,408	0,11065	0
GSPATT00001226001	-1,729	0,00239	-1	GSPATT00023254001	-0,021	0,93274	0
GSPATT00001250001	-0,495	0,01962	0	GSPATT00023524001	-1,299	0,00004	-1
GSPATT00001289001	-0,134	0,36262	0	GSPATT00023575001	0,339	0,04028	0
GSPATT00001308001	0,452	0,0481	0	GSPATT00023645001	0,103	0,51728	0
GSPATT00001370001	0,219	0,1963	0	GSPATT00023667001	-0,421	0,04545	0
GSPATT00001516001	-0,138	0,34571	0	GSPATT00023883001	0,351	0,06871	0
GSPATT00001648001	-2,455	0	-1	GSPATT00023911001	0,332	0,02952	0
GSPATT00001669001	-0,037	0,78045	0	GSPATT00023915001	0,418	0,02957	0
GSPATT00001741001	0,068	0,70586	0	GSPATT00023926001	-0,1	0,57657	0
GSPATT00001797001	1,121	0,00009	1	GSPATT00023954001	0,22	0,22413	0
GSPATT00001802001	0,393	0,09075	0	GSPATT00024076001	0,966	0,0008	0
GSPATT00002052001	0,333	0,07203	0	GSPATT00024217001	0,867	0,00079	0
GSPATT00002095001	-0,036	0,82513	0	GSPATT00024226001	0,274	0,23486	0
GSPATT00002350001	0,323	0,12471	0	GSPATT00024229001	0,534	0,01445	0
GSPATT00002485001	0,016	0,91286	0	GSPATT00024361001	0,391	0,03397	0
GSPATT00002539001	0,748	0,00281	0	GSPATT00024488001	1,929	0,00003	1
GSPATT00002540001	0,687	0,01064	0	GSPATT00024803001	0,179	0,50259	0
GSPATT00002669001	0,347	0,04238	0	GSPATT00024824001	0,224	0,46418	0
GSPATT00002803001	-2,664	0,00001	-1	GSPATT00025402001	0,193	0,32529	0
GSPATT00002812001	0,982	0,00418	0	GSPATT00025403001	0,485	0,02971	0
GSPATT00002827001	0,039	0,80706	0	GSPATT00025404001	0,055	0,75224	0
GSPATT00003125001	0,192	0,20404	0	GSPATT00025409001	0,695	0,00746	0
GSPATT00003201001	0,867	0,00568	0	GSPATT00025434001	2,076	0	1
GSPATT00003330001	-0,65	0,01363	0	GSPATT00025445001	-0,258	0,18447	0
GSPATT00003870001	0,905	0,00024	0	GSPATT00025446001	-0,233	0,22419	0
GSPATT00004067001	-0,031	0,89029	0	GSPATT00025620001	-2,222	0,00008	-1
GSPATT00004178001	2,09	0	1	GSPATT00025776001	-0,141	0,50398	0
GSPATT00004478001	0,866	0,00048	0	GSPATT00025782001	-0,406	0,10809	0
GSPATT00004486001	0,093	0,52562	0	GSPATT00025897001	0,722	0,0068	0
GSPATT00004700001	-0,512	0,01026	0	GSPATT00026015001	-0,01	0,93699	0
GSPATT00004901001	0,489	0,04492	0	GSPATT00026018001	-0,126	0,54405	0
GSPATT00005000001	0,053	0,6952	0	GSPATT00026020001	0,065	0,63486	0
GSPATT00005055001	0,621	0,00372	0	GSPATT00026022001	0,577	0,00715	0
GSPATT00005348001	0,239	0,13893	0	GSPATT00026174001	1,148	0,00285	1
GSPATT00005401001	-0,375	0,06585	0	GSPATT00026199001	0,423	0,07113	0
GSPATT00005545001	0,196	0,25185	0	GSPATT00026202001	0,132	0,69157	0
GSPATT00005550001	0,219	0,21448	0	GSPATT00026205001	0,41	0,02359	0
GSPATT00005596001	0,673	0,0013	0	GSPATT00026207001	0,226	0,19413	0
GSPATT00005614001	0,401	0,02429	0	GSPATT00026603001	-0,864	0,04702	0
GSPATT00005787001	0,145	0,47364	0	GSPATT00026647001	-0,065	0,84197	0
GSPATT00005934001	0,631	0,00222	0	GSPATT00026808001	0,645	0,00101	0
GSPATT00005951001	-0,034	0,81726	0	GSPATT00026945001	0,882	0,00018	0
GSPATT00006105001	0,219	0,41076	0	GSPATT00026980001	-0,696	0,02691	0
GSPATT00006175001	0,509	0,1113	0	GSPATT00027002001	-0,598	0,02814	0
GSPATT00006267001	0,048	0,78023	0	GSPATT00027420001	0,675	0,00918	0
GSPATT00006443001	-0,296	0,26707	0	GSPATT00027510001	-0,41	0,01592	0
GSPATT00006660001	0,042	0,75014	0	GSPATT00027628001	0,752	0,00108	0
GSPATT00006812001	0,247	0,08762	0	GSPATT00027658001	0,158	0,28469	0
GSPATT00006969001	0,405	0,11317	0	GSPATT00027759001	0,752	0,01625	0
GSPATT00007043001	1,432	0,00004	1	GSPATT00027831001	1,076	0,00008	1
GSPATT00007243001	0,258	0,11177	0	GSPATT00027877001	0,758	0,00106	0
GSPATT00007291001	0,158	0,28251	0	GSPATT00027992001	-0,104	0,53897	0
GSPATT00007671001	-1,431	0,00067	-1	GSPATT00028020001	1,049	0,00011	1

GSPATT00007744001	0,304	0,08781	0	GSPATT00028026001	0,735	0,00115	0
GSPATT00007760001	0,871	0,00045	0	GSPATT00028027001	0,692	0,0099	0
GSPATT00007786001	0,238	0,12785	0	GSPATT00028113001	1,049	0,00192	1
GSPATT00007975001	0,396	0,04129	0	GSPATT00028126001	0,574	0,00662	0
GSPATT00008161001	2,401	0	1	GSPATT00028127001	1,666	0	1
GSPATT00008218001	1,001	0,00037	1	GSPATT00028128001	0,2	0,18528	0
GSPATT00008294001	1,862	0,00001	1	GSPATT00028146001	-0,056	0,71186	0
GSPATT00008295001	1,733	0,00021	1	GSPATT00028152001	0,312	0,18325	0
GSPATT00008401001	0,039	0,78242	0	GSPATT00028176001	-0,076	0,57	0
GSPATT00008512001	0,357	0,06391	0	GSPATT00028177001	0,461	0,01662	0
GSPATT00008733001	0,565	0,00234	0	GSPATT00028178001	0,347	0,04773	0
GSPATT00009399001	0,067	0,85641	0	GSPATT00028270001	0,562	0,00252	0
GSPATT00009400001	0,557	0,05258	0	GSPATT00028315001	0,054	0,68657	0
GSPATT00009524001	0,752	0,00072	0	GSPATT00028385001	-0,459	0,01665	0
GSPATT00009566001	1,203	0,00013	1	GSPATT00028411001	0,765	0,0418	0
GSPATT00009598001	0,527	0,00529	0	GSPATT00028455001	-0,011	0,95158	0
GSPATT00009745001	-1,536	0,00133	-1	GSPATT00028472001	0,036	0,85105	0
GSPATT00009876001	1,619	0	1	GSPATT00028479001	-0,028	0,85373	0
GSPATT00010127001	0,208	0,30607	0	GSPATT00028515001	0,356	0,07284	0
GSPATT00010129001	0,259	0,2078	0	GSPATT00028560001	-3,376	0	-1
GSPATT00010348001	-0,197	0,26965	0	GSPATT00028618001	-0,022	0,89648	0
GSPATT00010352001	3,057	0	1	GSPATT00028637001	0,235	0,34077	0
GSPATT00010353001	2,543	0,00003	1	GSPATT00028675001	0,851	0,00028	0
GSPATT00010440001	0,011	0,93285	0	GSPATT00028827001	-0,477	0,07177	0
GSPATT00010447001	0,824	0,00576	0	GSPATT00028847001	-1,597	0	-1
GSPATT00010448001	0,386	0,1129	0	GSPATT00028894001	-0,533	0,00646	0
GSPATT00010615001	0,827	0,00035	0	GSPATT00028954001	-0,724	0,00164	0
GSPATT00010741001	0,219	0,21126	0	GSPATT00029100001	-0,062	0,79269	0
GSPATT00010751001	0,385	0,03146	0	GSPATT00029158001	-0,005	0,97596	0
GSPATT00010783001	-0,256	0,31112	0	GSPATT00029173001	0,138	0,34159	0
GSPATT00010839001	0,892	0,01367	0	GSPATT00029363001	1,21	0,00015	1
GSPATT00010846001	0,562	0,04885	0	GSPATT00029377001	-0,243	0,26515	0
GSPATT00010987001	0,663	0,00641	0	GSPATT00029412001	-0,481	0,0793	0
GSPATT00011049001	-0,006	0,96222	0	GSPATT00029428001	0,483	0,01379	0
GSPATT00011076001	0,02	0,8917	0	GSPATT00029430001	-0,356	0,0534	0
GSPATT00011252001	-0,246	0,20466	0	GSPATT00029431001	0,251	0,16889	0
GSPATT00011275001	0,242	0,1838	0	GSPATT00029433001	0,663	0,01729	0
GSPATT00011347001	1,768	0,00005	1	GSPATT00029484001	0,665	0,00137	0
GSPATT00011459001	-2,57	0,00001	-1	GSPATT00029678001	-0,109	0,69058	0
GSPATT00011676001	0,136	0,30806	0	GSPATT00029739001	0,81	0,00323	0
GSPATT00011836001	0,971	0,00458	0	GSPATT00029750001	0,907	0,00106	0
GSPATT00011851001	0,261	0,28467	0	GSPATT00029805001	-0,134	0,51026	0
GSPATT00011969001	0,052	0,69884	0	GSPATT00029910001	0,873	0,00886	0
GSPATT00011991001	1,115	0,00161	1	GSPATT00030000001	0,107	0,49189	0
GSPATT00012070001	-0,627	0,00801	0	GSPATT00030118001	-0,924	0,0029	0
GSPATT00012266001	-2,957	0	-1	GSPATT00030165001	0,947	0,01145	0
GSPATT00012442001	0,124	0,39221	0	GSPATT00030244001	0,934	0,01354	0
GSPATT00012508001	0,826	0,00263	0	GSPATT00030274001	-0,248	0,24086	0
GSPATT00012593001	-0,057	0,67906	0	GSPATT00030420001	0,009	0,94162	0
GSPATT00012678001	-0,029	0,84403	0	GSPATT00030846001	-3,851	0	-1
GSPATT00012787001	0,562	0,00378	0	GSPATT00030941001	-0,143	0,34817	0
GSPATT00012913001	0,495	0,01652	0	GSPATT00030952001	-0,016	0,91391	0
GSPATT00012939001	1,318	0,00003	1	GSPATT00030964001	-0,044	0,77814	0
GSPATT00013032001	0,379	0,02191	0	GSPATT00031118001	1,385	0,00029	1
GSPATT00013033001	1,336	0,00061	1	GSPATT00031128001	-1,486	0,00002	-1
GSPATT00013220001	0,04	0,8336	0	GSPATT00031161001	2,492	0	1
GSPATT00013437001	-0,031	0,86058	0	GSPATT00031203001	-1,066	0,00004	-1
GSPATT00013468001	-0,118	0,51732	0	GSPATT00031278001	0,903	0,00128	0
GSPATT00013568001	1,448	0,00037	1	GSPATT00031485001	0,609	0,00227	0
GSPATT00013649001	-1,31	0,0001	-1	GSPATT00031486001	1,149	0,00221	1
GSPATT00013672001	0,039	0,79017	0	GSPATT00031487001	0,99	0,002	0
GSPATT00013773001	0,064	0,6282	0	GSPATT00031581001	0,409	0,01976	0
GSPATT00013907001	-0,24	0,3218	0	GSPATT00031582001	0,488	0,01159	0
GSPATT00013911001	-1,098	0,01747	-1	GSPATT00031589001	1,453	0,0009	1
GSPATT00013941001	0,431	0,03011	0	GSPATT00031612001	2,01	0,00001	1
GSPATT00013959001	1,407	0,0001	1	GSPATT00031620001	0,143	0,55437	0
GSPATT00014056001	1,151	0,00013	1	GSPATT00031621001	0,411	0,10532	0

GSPATT00014088001	0,517	0,03193	0	GSPATT00031772001	0,207	0,28805	0
GSPATT00014099001	0,788	0,00354	0	GSPATT00031815001	-0,045	0,73539	0
GSPATT00014100001	0,628	0,00216	0	GSPATT00031825001	0,037	0,77311	0
GSPATT00014133001	0,571	0,02431	0	GSPATT00031857001	0,006	0,96804	0
GSPATT00014202001	0,571	0,00343	0	GSPATT00031882001	0,046	0,76052	0
GSPATT00014234001	-0,019	0,94089	0	GSPATT00031904001	0,321	0,04964	0
GSPATT00014246001	0,51	0,01388	0	GSPATT00031946001	0,212	0,1392	0
GSPATT00014247001	0,275	0,31033	0	GSPATT00032049001	1,415	0,00003	1
GSPATT00014253001	-0,142	0,35399	0	GSPATT00032099001	0,878	0,00102	0
GSPATT00014329001	1,236	0,00008	1	GSPATT00032123001	2,246	0	1
GSPATT00014335001	-0,025	0,84538	0	GSPATT00032126001	3,068	0	1
GSPATT00014350001	-0,099	0,44714	0	GSPATT00032130001	1,691	0	1
GSPATT00014466001	0,607	0,0211	0	GSPATT00032201001	-0,256	0,07664	0
GSPATT00014523001	1,029	0,0076	1	GSPATT00032282001	-0,934	0,03839	0
GSPATT00014525001	1,28	0,0002	1	GSPATT00032351001	-0,087	0,60276	0
GSPATT00014567001	0,89	0,00055	0	GSPATT00032466001	-1,497	0,00001	-1
GSPATT00014588001	0,16	0,39805	0	GSPATT00032626001	1,023	0,02188	0
GSPATT00014766001	0,379	0,03636	0	GSPATT00032680001	1,174	0,00017	1
GSPATT00015032001	0,07	0,58712	0	GSPATT00032704001	0,794	0,00033	0
GSPATT00015069001	-1,155	0,0039	-1	GSPATT00032773001	0,48	0,00834	0
GSPATT00015079001	-0,239	0,10595	0	GSPATT00032797001	0,422	0,03104	0
GSPATT00015088001	0,299	0,05094	0	GSPATT00033038001	0,44	0,14681	0
GSPATT00015178001	2,216	0	1	GSPATT00033064001	0,437	0,10599	0
GSPATT00015260001	0,046	0,77054	0	GSPATT00033175001	-0,453	0,02416	0
GSPATT00015397001	0,341	0,07634	0	GSPATT00033184001	-1,616	0,00013	-1
GSPATT00015419001	0,131	0,44139	0	GSPATT00033235001	0,994	0,00473	0
GSPATT00015675001	-1,594	0,00001	-1	GSPATT00033329001	-0,267	0,19365	0
GSPATT00015795001	1,253	0,00003	1	GSPATT00033602001	1,733	0,00201	1
GSPATT00015930001	0,506	0,01359	0	GSPATT00033686001	0,218	0,13899	0
GSPATT00015932001	2,844	0,00001	1	GSPATT00033691001	0,637	0,0021	0
GSPATT00015933001	1,267	0,00003	1	GSPATT00033745001	0,089	0,5511	0
GSPATT00015958001	0,57	0,02514	0	GSPATT00033788001	0,71	0,00785	0
GSPATT00015959001	0,551	0,00394	0	GSPATT00033809001	1,165	0,00013	1
GSPATT00015960001	0,246	0,39617	0	GSPATT00033830001	0,158	0,29138	0
GSPATT00016014001	0,361	0,04847	0	GSPATT00033879001	0,892	0,00128	0
GSPATT00016098001	0,648	0,00105	0	GSPATT00033973001	-2,579	0	-1
GSPATT00016151001	1,122	0,00008	1	GSPATT00034183001	0,407	0,0172	0
GSPATT00016327001	0,649	0,00694	0	GSPATT00034304001	1,194	0,00003	1
GSPATT00016622001	0,521	0,02121	0	GSPATT00034349001	0,099	0,50982	0
GSPATT00016692001	0,905	0,00153	0	GSPATT00034411001	0,328	0,04486	0
GSPATT00016729001	0,287	0,09802	0	GSPATT00034412001	0,065	0,65671	0
GSPATT00016813001	0,916	0,00072	0	GSPATT00034501001	-0,264	0,06932	0
GSPATT00016940001	-2,525	0,00002	-1	GSPATT00034507001	-1,757	0,002	-1
GSPATT00017137001	0,492	0,02473	0	GSPATT00034530001	-0,757	0,00217	0
GSPATT00017253001	0,817	0,00077	0	GSPATT00034557001	0,064	0,6691	0
GSPATT00017322001	1,521	0,00009	1	GSPATT00034730001	0,714	0,00248	0
GSPATT00017458001	-0,593	0,00288	0	GSPATT00034731001	1,134	0,00029	1
GSPATT00017522001	0,536	0,05133	0	GSPATT00034783001	-1,519	0,00003	-1
GSPATT00017939001	0,564	0,01365	0	GSPATT00034881001	0,311	0,04131	0
GSPATT00018120001	0,405	0,03579	0	GSPATT00034924001	1,499	0,00094	1
GSPATT00018176001	0,286	0,13868	0	GSPATT00034945001	2,202	0	1
GSPATT00018221001	-1,731	0,00009	-1	GSPATT00034972001	0,359	0,02095	0
GSPATT00018285001	0,607	0,03673	0	GSPATT00035053001	0,554	0,0164	0
GSPATT00018286001	0,547	0,01699	0	GSPATT00035082001	0,013	0,93508	0
GSPATT00018299001	2,302	0	1	GSPATT00035090001	1,242	0,01803	1
GSPATT00018324001	0,468	0,06312	0	GSPATT00035110001	0,776	0,02913	0
GSPATT00018367001	0,012	0,96303	0	GSPATT00035153001	0,205	0,2602	0
GSPATT00018619001	0,353	0,20611	0	GSPATT00035445001	0,383	0,06155	0
GSPATT00018741001	0,659	0,00151	0	GSPATT00035491001	-2,066	0,00002	-1
GSPATT00018742001	0,353	0,04011	0	GSPATT00035536001	0,264	0,25914	0
GSPATT00018752001	2,824	0	1	GSPATT00035599001	0,523	0,00967	0
GSPATT00018758001	2,149	0	1	GSPATT00035600001	0,306	0,04161	0
GSPATT00018780001	0,691	0,01868	0	GSPATT00035603001	0,808	0,00069	0
GSPATT00018829001	0,329	0,23489	0	GSPATT00035664001	-1,285	0,00108	-1
GSPATT00019184001	0,032	0,83613	0	GSPATT00035672001	0,924	0,00022	0
GSPATT00019189001	-0,291	0,12085	0	GSPATT00035681001	0,628	0,00278	0
GSPATT00019250001	0,656	0,00171	0	GSPATT00035758001	-1,297	0,00005	-1

GSPATT00019251001	-0,139	0,2928	0	GSPATT00035913001	-0,255	0,30484	0
GSPATT00019254001	0,218	0,2943	0	GSPATT00036011001	0,418	0,0183	0
GSPATT00019389001	0,172	0,21789	0	GSPATT00036096001	0,118	0,59195	0
GSPATT00019417001	-0,087	0,59287	0	GSPATT00036105001	0,475	0,01933	0
GSPATT00019603001	0,551	0,01253	0	GSPATT00036127001	0,777	0,00038	0
GSPATT00019876001	-0,036	0,9177	0	GSPATT00036267001	1,177	0,00036	1
GSPATT00020053001	-0,207	0,22189	0	GSPATT00036475001	-0,585	0,0059	0
GSPATT00020054001	0,28	0,14998	0	GSPATT00036487001	-0,386	0,05882	0
GSPATT00020186001	-0,459	0,06575	0	GSPATT00036711001	0,158	0,40877	0
GSPATT00020286001	-1,05	0,00021	-1	GSPATT00036812001	0,669	0,00387	0
GSPATT00020353001	0,488	0,03007	0	GSPATT00036815001	-0,206	0,27728	0
GSPATT00020355001	0,399	0,03212	0	GSPATT00036909001	-0,089	0,69971	0
GSPATT00020442001	0,565	0,009	0	GSPATT00036976001	0,082	0,61383	0
GSPATT00020780001	-0,921	0,00026	0	GSPATT00037113001	-0,126	0,51661	0
GSPATT00020881001	0,497	0,0253	0	GSPATT00037118001	0,536	0,01752	0
GSPATT00020934001	0,337	0,02604	0	GSPATT00037119001	1,348	0,00024	1
GSPATT00020968001	1,383	0,00001	1	GSPATT00037130001	-0,147	0,41686	0
GSPATT00021053001	0,674	0,02023	0	GSPATT00037160001	1,034	0,00084	1
GSPATT00021078001	0,339	0,1892	0	GSPATT00037555001	1,057	0,0001	1
GSPATT00021123001	-1,327	0,00005	-1	GSPATT00037556001	0,583	0,00547	0
GSPATT00021196001	-0,237	0,09382	0	GSPATT00037585001	1,309	0,00003	1
GSPATT00021225001	0,909	0,00125	0	GSPATT00037590001	0,899	0,00058	0
GSPATT00021341001	0,726	0,00168	0	GSPATT00037672001	1,122	0,00011	1
GSPATT00021345001	0,792	0,00065	0	GSPATT00037716001	0,113	0,41615	0
GSPATT00021421001	0,26	0,09503	0	GSPATT00037729001	0,252	0,07882	0
GSPATT00021453001	0,568	0,01115	0	GSPATT00037730001	0,087	0,55556	0
GSPATT00021463001	0,187	0,32414	0	GSPATT00037899001	1,357	0,00003	1
GSPATT00021514001	-0,385	0,03271	0	GSPATT00037921001	0,469	0,01243	0
GSPATT00021518001	-1,039	0,00036	-1	GSPATT00037923001	0,63	0,00481	0
GSPATT00021521001	0,151	0,3119	0	GSPATT00037924001	0,253	0,13338	0
GSPATT00021537001	0,203	0,36082	0	GSPATT00037953001	0,708	0,00099	0
GSPATT00021553001	0,151	0,41981	0	GSPATT00038018001	-0,128	0,53853	0
GSPATT00021724001	0,203	0,34898	0	GSPATT00038150001	0,261	0,13523	0
GSPATT00021829001	0,857	0,00244	0	GSPATT00038173001	0,82	0,03887	0
GSPATT00021996001	0,704	0,01954	0	GSPATT00038174001	0,838	0,12108	0
GSPATT00022065001	0,429	0,02791	0	GSPATT00038238001	-0,208	0,38852	0
GSPATT00022194001	0,25	0,11301	0	GSPATT00038245001	0,014	0,92514	0
GSPATT00022292001	0,557	0,00245	0	GSPATT00038695001	-0,518	0,01381	0
GSPATT00022293001	0,059	0,75238	0	GSPATT00039053001	0,586	0,00254	0
GSPATT00022405001	-0,359	0,09691	0	GSPATT00039200001	0,402	0,0233	0
GSPATT00022582001	0,485	0,00767	0	GSPATT00039341001	0,375	0,02393	0
GSPATT00022714001	-0,242	0,34305	0	GSPATT00039393001	-0,004	0,98646	0
GSPATT00022781001	0,543	0,00457	0				
GSPATT00022792001	0,634	0,00443	0				

GO 000-6351/-6355/-51252 GSPATTregulation of) transcription, DNA-dependent/regulation of RNA metabolic process

ID	coeff.	p-value	signif.	ID	coeff.	p-value	signif.
GSPATT00000430001	0,805	0,00385	0	GSPATT00022877001	0,756	0,0172	0
GSPATT00000588001	0,624	0,07319	0	GSPATT00022959001	1,261	0,00015	1
GSPATT00000689001	0,677	0,00237	0	GSPATT00022980001	0,614	0,0183	0
GSPATT00000920001	0,161	0,28196	0	GSPATT00023028001	-1,34	0,00001	-1
GSPATT00001199001	-0,4	0,07351	0	GSPATT00023176001	-0,179	0,37643	0
GSPATT00001224001	-0,004	0,97956	0	GSPATT00023182001	-0,408	0,11065	0
GSPATT00001226001	-1,729	0,00239	-1	GSPATT00023254001	-0,021	0,93274	0
GSPATT00001250001	-0,495	0,01962	0	GSPATT00023524001	-1,299	0,00004	-1
GSPATT00001308001	0,452	0,0481	0	GSPATT00023575001	0,339	0,04028	0
GSPATT00001370001	0,219	0,1963	0	GSPATT00023645001	0,103	0,51728	0
GSPATT00001516001	-0,138	0,34571	0	GSPATT00023667001	-0,421	0,04545	0
GSPATT00001648001	-2,455	0	-1	GSPATT00023883001	0,351	0,06871	0
GSPATT00001669001	-0,037	0,78045	0	GSPATT00023911001	0,332	0,02952	0
GSPATT00001741001	0,068	0,70586	0	GSPATT00023915001	0,418	0,02957	0
GSPATT00001802001	0,393	0,09075	0	GSPATT00023926001	-0,1	0,57657	0
GSPATT00002052001	0,333	0,07203	0	GSPATT00023954001	0,22	0,22413	0
GSPATT00002095001	-0,036	0,82513	0	GSPATT00024076001	0,966	0,0008	0
GSPATT00002350001	0,323	0,12471	0	GSPATT00024217001	0,867	0,00079	0
GSPATT00002485001	0,016	0,91286	0	GSPATT00024226001	0,274	0,23486	0
GSPATT00002539001	0,748	0,00281	0	GSPATT00024229001	0,534	0,01445	0

GSPATT00002540001	0,687	0,01064	0	GSPATT00024361001	0,391	0,03397	0
GSPATT00002669001	0,347	0,04238	0	GSPATT00024488001	1,929	0,00003	1
GSPATT00002803001	-2,664	0,00001	-1	GSPATT00024803001	0,179	0,50259	0
GSPATT00002812001	0,982	0,00418	0	GSPATT00024824001	0,224	0,46418	0
GSPATT00002827001	0,039	0,80706	0	GSPATT00025402001	0,193	0,32529	0
GSPATT00003125001	0,192	0,20404	0	GSPATT00025403001	0,485	0,02971	0
GSPATT00003201001	0,867	0,00568	0	GSPATT00025404001	0,055	0,75224	0
GSPATT00003330001	-0,65	0,01363	0	GSPATT00025409001	0,695	0,00746	0
GSPATT00003870001	0,905	0,00024	0	GSPATT00025434001	2,076	0	1
GSPATT00004067001	-0,031	0,89029	0	GSPATT00025445001	-0,258	0,18447	0
GSPATT00004178001	2,09	0	1	GSPATT00025446001	-0,233	0,22419	0
GSPATT00004478001	0,866	0,00048	0	GSPATT00025620001	-2,222	0,00008	-1
GSPATT00004486001	0,093	0,52562	0	GSPATT00025776001	-0,141	0,50398	0
GSPATT00004700001	-0,512	0,01026	0	GSPATT00025782001	-0,406	0,10809	0
GSPATT00004901001	0,489	0,04492	0	GSPATT00025897001	0,722	0,0068	0
GSPATT00005000001	0,053	0,6952	0	GSPATT00026015001	-0,01	0,93699	0
GSPATT00005055001	0,621	0,00372	0	GSPATT00026018001	-0,126	0,54405	0
GSPATT00005348001	0,239	0,13893	0	GSPATT00026020001	0,065	0,63486	0
GSPATT00005401001	-0,375	0,06585	0	GSPATT00026022001	0,577	0,00715	0
GSPATT00005545001	0,196	0,25185	0	GSPATT00026174001	1,148	0,00285	1
GSPATT00005550001	0,219	0,21448	0	GSPATT00026199001	0,423	0,07113	0
GSPATT00005596001	0,673	0,0013	0	GSPATT00026202001	0,132	0,69157	0
GSPATT00005614001	0,401	0,02429	0	GSPATT00026205001	0,41	0,02359	0
GSPATT00005787001	0,145	0,47364	0	GSPATT00026207001	0,226	0,19413	0
GSPATT00005934001	0,631	0,00222	0	GSPATT00026603001	-0,864	0,04702	0
GSPATT00005951001	-0,034	0,81726	0	GSPATT00026647001	-0,065	0,84197	0
GSPATT00006105001	0,219	0,41076	0	GSPATT00026808001	0,645	0,00101	0
GSPATT00006175001	0,509	0,1113	0	GSPATT00026945001	0,882	0,00018	0
GSPATT00006267001	0,048	0,78023	0	GSPATT00027002001	-0,598	0,02814	0
GSPATT00006443001	-0,296	0,26707	0	GSPATT00027420001	0,675	0,00918	0
GSPATT00006660001	0,042	0,75014	0	GSPATT00027510001	-0,41	0,01592	0
GSPATT00006812001	0,247	0,08762	0	GSPATT00027628001	0,752	0,00108	0
GSPATT00006969001	0,405	0,11317	0	GSPATT00027658001	0,158	0,28469	0
GSPATT00007043001	1,432	0,00004	1	GSPATT00027759001	0,752	0,01625	0
GSPATT00007243001	0,258	0,11177	0	GSPATT00027831001	1,076	0,00008	1
GSPATT00007291001	0,158	0,28251	0	GSPATT00027877001	0,758	0,00106	0
GSPATT00007671001	-1,431	0,00067	-1	GSPATT00027992001	-0,104	0,53897	0
GSPATT00007744001	0,304	0,08781	0	GSPATT00028020001	1,049	0,00011	1
GSPATT00007760001	0,871	0,00045	0	GSPATT00028026001	0,735	0,00115	0
GSPATT00007786001	0,238	0,12785	0	GSPATT00028027001	0,692	0,0099	0
GSPATT00007975001	0,396	0,04129	0	GSPATT00028113001	1,049	0,00192	1
GSPATT00008161001	2,401	0	1	GSPATT00028126001	0,574	0,00662	0
GSPATT00008218001	1,001	0,00037	1	GSPATT00028127001	1,666	0	1
GSPATT00008294001	1,862	0,00001	1	GSPATT00028128001	0,2	0,18528	0
GSPATT00008295001	1,733	0,00021	1	GSPATT00028146001	-0,056	0,71186	0
GSPATT00008401001	0,039	0,78242	0	GSPATT00028152001	0,312	0,18325	0
GSPATT00008512001	0,357	0,06391	0	GSPATT00028176001	-0,076	0,57	0
GSPATT00008733001	0,565	0,00234	0	GSPATT00028177001	0,461	0,01662	0
GSPATT00009399001	0,067	0,85641	0	GSPATT00028178001	0,347	0,04773	0
GSPATT00009400001	0,557	0,05258	0	GSPATT00028270001	0,562	0,00252	0
GSPATT00009524001	0,752	0,00072	0	GSPATT00028315001	0,054	0,68657	0
GSPATT00009566001	1,203	0,00013	1	GSPATT00028385001	-0,459	0,01665	0
GSPATT00009598001	0,527	0,00529	0	GSPATT00028411001	0,765	0,0418	0
GSPATT00009745001	-1,536	0,00133	-1	GSPATT00028455001	-0,011	0,95158	0
GSPATT00009876001	1,619	0	1	GSPATT00028472001	0,036	0,85105	0
GSPATT00010127001	0,208	0,30607	0	GSPATT00028479001	-0,028	0,85373	0
GSPATT00010129001	0,259	0,2078	0	GSPATT00028515001	0,356	0,07284	0
GSPATT00010348001	-0,197	0,26965	0	GSPATT00028560001	-3,376	0	-1
GSPATT00010352001	3,057	0	1	GSPATT00028618001	-0,022	0,89648	0
GSPATT00010353001	2,543	0,00003	1	GSPATT00028675001	0,851	0,00028	0
GSPATT00010440001	0,011	0,93285	0	GSPATT00028827001	-0,477	0,07177	0
GSPATT00010447001	0,824	0,00576	0	GSPATT00028847001	-1,597	0	-1
GSPATT00010448001	0,386	0,1129	0	GSPATT00028954001	-0,724	0,00164	0
GSPATT00010615001	0,827	0,00035	0	GSPATT00029100001	-0,062	0,79269	0
GSPATT00010741001	0,219	0,21126	0	GSPATT00029158001	-0,005	0,97596	0
GSPATT00010751001	0,385	0,03146	0	GSPATT00029173001	0,138	0,34159	0
GSPATT00010783001	-0,256	0,31112	0	GSPATT00029363001	1,21	0,00015	1

GSPATT00010839001	0,892	0,01367	0
GSPATT00010846001	0,562	0,04885	0
GSPATT00010987001	0,663	0,00641	0
GSPATT00011049001	-0,006	0,96222	0
GSPATT00011076001	0,02	0,8917	0
GSPATT00011252001	-0,246	0,20466	0
GSPATT00011275001	0,242	0,1838	0
GSPATT00011347001	1,768	0,00005	1
GSPATT00011459001	-2,57	0,00001	-1
GSPATT00011676001	0,136	0,30806	0
GSPATT00011836001	0,971	0,00458	0
GSPATT00011851001	0,261	0,28467	0
GSPATT00011969001	0,052	0,69884	0
GSPATT00011991001	1,115	0,00161	1
GSPATT00012070001	-0,627	0,00801	0
GSPATT00012266001	-2,957	0	-1
GSPATT00012442001	0,124	0,39221	0
GSPATT00012508001	0,826	0,00263	0
GSPATT00012593001	-0,057	0,67906	0
GSPATT00012678001	-0,029	0,84403	0
GSPATT00012787001	0,562	0,00378	0
GSPATT00012913001	0,495	0,01652	0
GSPATT00012939001	1,318	0,00003	1
GSPATT00013032001	0,379	0,02191	0
GSPATT00013033001	1,336	0,00061	1
GSPATT00013220001	0,04	0,8336	0
GSPATT00013437001	-0,031	0,86058	0
GSPATT00013468001	-0,118	0,51732	0
GSPATT00013568001	1,448	0,00037	1
GSPATT00013649001	-1,31	0,0001	-1
GSPATT00013672001	0,039	0,79017	0
GSPATT00013773001	0,064	0,6282	0
GSPATT00013907001	-0,24	0,3218	0
GSPATT00013911001	-1,098	0,01747	-1
GSPATT00013941001	0,431	0,03011	0
GSPATT00013959001	1,407	0,0001	1
GSPATT00014056001	1,151	0,00013	1
GSPATT00014088001	0,517	0,03193	0
GSPATT00014099001	0,788	0,00354	0
GSPATT00014100001	0,628	0,00216	0
GSPATT00014133001	0,571	0,02431	0
GSPATT00014202001	0,571	0,00343	0
GSPATT00014234001	-0,019	0,94089	0
GSPATT00014246001	0,51	0,01388	0
GSPATT00014247001	0,275	0,31033	0
GSPATT00014253001	-0,142	0,35399	0
GSPATT00014329001	1,236	0,00008	1
GSPATT00014335001	-0,025	0,84538	0
GSPATT00014350001	-0,099	0,44714	0
GSPATT00014466001	0,607	0,0211	0
GSPATT00014523001	1,029	0,0076	1
GSPATT00014525001	1,28	0,0002	1
GSPATT00014567001	0,89	0,00055	0
GSPATT00014588001	0,16	0,39805	0
GSPATT00014766001	0,379	0,03636	0
GSPATT00015032001	0,07	0,58712	0
GSPATT00015069001	-1,155	0,0039	-1
GSPATT00015079001	-0,239	0,10595	0
GSPATT00015088001	0,299	0,05094	0
GSPATT00015178001	2,216	0	1
GSPATT00015260001	0,046	0,77054	0
GSPATT00015397001	0,341	0,07634	0
GSPATT00015419001	0,131	0,44139	0
GSPATT00015675001	-1,594	0,00001	-1
GSPATT00015795001	1,253	0,00003	1
GSPATT00015930001	0,506	0,01359	0
GSPATT00015932001	2,844	0,00001	1

GSPATT00029377001	-0,243	0,26515	0
GSPATT00029412001	-0,481	0,0793	0
GSPATT00029428001	0,483	0,01379	0
GSPATT00029430001	-0,356	0,0534	0
GSPATT00029431001	0,251	0,16889	0
GSPATT00029433001	0,663	0,01729	0
GSPATT00029484001	0,665	0,00137	0
GSPATT00029678001	-0,109	0,69058	0
GSPATT00029739001	0,81	0,00323	0
GSPATT00029750001	0,907	0,00106	0
GSPATT00029805001	-0,134	0,51026	0
GSPATT00029910001	0,873	0,00886	0
GSPATT00030000001	0,107	0,49189	0
GSPATT00030118001	-0,924	0,0029	0
GSPATT00030165001	0,947	0,01145	0
GSPATT00030244001	0,934	0,01354	0
GSPATT00030274001	-0,248	0,24086	0
GSPATT00030420001	0,009	0,94162	0
GSPATT00030846001	-3,851	0	-1
GSPATT00030941001	-0,143	0,34817	0
GSPATT00030952001	-0,016	0,91391	0
GSPATT00030964001	-0,044	0,77814	0
GSPATT00031118001	1,385	0,00029	1
GSPATT00031128001	-1,486	0,00002	-1
GSPATT00031161001	2,492	0	1
GSPATT00031203001	-1,066	0,00004	-1
GSPATT00031278001	0,903	0,00128	0
GSPATT00031485001	0,609	0,00227	0
GSPATT00031486001	1,149	0,00221	1
GSPATT00031487001	0,99	0,002	0
GSPATT00031581001	0,409	0,01976	0
GSPATT00031582001	0,488	0,01159	0
GSPATT00031589001	1,453	0,0009	1
GSPATT00031612001	2,01	0,00001	1
GSPATT00031620001	0,143	0,55437	0
GSPATT00031621001	0,411	0,10532	0
GSPATT00031772001	0,207	0,28805	0
GSPATT00031815001	-0,045	0,73539	0
GSPATT00031825001	0,037	0,77311	0
GSPATT00031857001	0,006	0,96804	0
GSPATT00031882001	0,046	0,76052	0
GSPATT00031904001	0,321	0,04964	0
GSPATT00031946001	0,212	0,1392	0
GSPATT00032049001	1,415	0,00003	1
GSPATT00032099001	0,878	0,00102	0
GSPATT00032123001	2,246	0	1
GSPATT00032126001	3,068	0	1
GSPATT00032130001	1,691	0	1
GSPATT00032201001	-0,256	0,07664	0
GSPATT00032282001	-0,934	0,03839	0
GSPATT00032351001	-0,087	0,60276	0
GSPATT00032466001	-1,497	0,00001	-1
GSPATT00032626001	1,023	0,02188	0
GSPATT00032680001	1,174	0,00017	1
GSPATT00032704001	0,794	0,00033	0
GSPATT00032773001	0,48	0,00834	0
GSPATT00032797001	0,422	0,03104	0
GSPATT00033038001	0,44	0,14681	0
GSPATT00033064001	0,437	0,10599	0
GSPATT00033175001	-0,453	0,02416	0
GSPATT00033184001	-1,616	0,00013	-1
GSPATT00033235001	0,994	0,00473	0
GSPATT00033329001	-0,267	0,19365	0
GSPATT00033602001	1,733	0,00201	1
GSPATT00033686001	0,218	0,13899	0
GSPATT00033691001	0,637	0,0021	0
GSPATT00033745001	0,089	0,5511	0

GSPATT00015933001	1,267	0,00003	1	GSPATT00033788001	0,71	0,00785	0
GSPATT00015958001	0,57	0,02514	0	GSPATT00033809001	1,165	0,00013	1
GSPATT00015959001	0,551	0,00394	0	GSPATT00033830001	0,158	0,29138	0
GSPATT00015960001	0,246	0,39617	0	GSPATT00033879001	0,892	0,00128	0
GSPATT00016014001	0,361	0,04847	0	GSPATT00033973001	-2,579	0	-1
GSPATT00016098001	0,648	0,00105	0	GSPATT00034183001	0,407	0,0172	0
GSPATT00016151001	1,122	0,00008	1	GSPATT00034304001	1,194	0,00003	1
GSPATT00016327001	0,649	0,00694	0	GSPATT00034349001	0,099	0,50982	0
GSPATT00016622001	0,521	0,02121	0	GSPATT00034411001	0,328	0,04486	0
GSPATT00016692001	0,905	0,00153	0	GSPATT00034412001	0,065	0,65671	0
GSPATT00016729001	0,287	0,09802	0	GSPATT00034501001	-0,264	0,06932	0
GSPATT00016813001	0,916	0,00072	0	GSPATT00034507001	-1,757	0,002	-1
GSPATT00016940001	-2,525	0,00002	-1	GSPATT00034530001	-0,757	0,00217	0
GSPATT00017137001	0,492	0,02473	0	GSPATT00034557001	0,064	0,6691	0
GSPATT00017322001	1,521	0,00009	1	GSPATT00034730001	0,714	0,00248	0
GSPATT00017458001	-0,593	0,00288	0	GSPATT00034731001	1,134	0,00029	1
GSPATT00017522001	0,536	0,05133	0	GSPATT00034783001	-1,519	0,00003	-1
GSPATT00017939001	0,564	0,01365	0	GSPATT00034881001	0,311	0,04131	0
GSPATT00018120001	0,405	0,03579	0	GSPATT00034924001	1,499	0,00094	1
GSPATT00018176001	0,286	0,13868	0	GSPATT00034945001	2,202	0	1
GSPATT00018221001	-1,731	0,00009	-1	GSPATT00034972001	0,359	0,02095	0
GSPATT00018285001	0,607	0,03673	0	GSPATT00035053001	0,554	0,0164	0
GSPATT00018286001	0,547	0,01699	0	GSPATT00035082001	0,013	0,93508	0
GSPATT00018299001	2,302	0	1	GSPATT00035090001	1,242	0,01803	1
GSPATT00018324001	0,468	0,06312	0	GSPATT00035110001	0,776	0,02913	0
GSPATT00018367001	0,012	0,96303	0	GSPATT00035153001	0,205	0,2602	0
GSPATT00018619001	0,353	0,20611	0	GSPATT00035445001	0,383	0,06155	0
GSPATT00018741001	0,659	0,00151	0	GSPATT00035491001	-2,066	0,00002	-1
GSPATT00018742001	0,353	0,04011	0	GSPATT00035536001	0,264	0,25914	0
GSPATT00018752001	2,824	0	1	GSPATT00035599001	0,523	0,00967	0
GSPATT00018758001	2,149	0	1	GSPATT00035600001	0,306	0,04161	0
GSPATT00018780001	0,691	0,01868	0	GSPATT00035603001	0,808	0,00069	0
GSPATT00018829001	0,329	0,23489	0	GSPATT00035664001	-1,285	0,00108	-1
GSPATT00019184001	0,032	0,83613	0	GSPATT00035672001	0,924	0,00022	0
GSPATT00019189001	-0,291	0,12085	0	GSPATT00035681001	0,628	0,00278	0
GSPATT00019250001	0,656	0,00171	0	GSPATT00035758001	-1,297	0,00005	-1
GSPATT00019251001	-0,139	0,2928	0	GSPATT00035913001	-0,255	0,30484	0
GSPATT00019254001	0,218	0,2943	0	GSPATT00036011001	0,418	0,0183	0
GSPATT00019389001	0,172	0,21789	0	GSPATT00036096001	0,118	0,59195	0
GSPATT00019417001	-0,087	0,59287	0	GSPATT00036105001	0,475	0,01933	0
GSPATT00019603001	0,551	0,01253	0	GSPATT00036127001	0,777	0,00038	0
GSPATT00019876001	-0,036	0,9177	0	GSPATT00036267001	1,177	0,00036	1
GSPATT00020053001	-0,207	0,22189	0	GSPATT00036475001	-0,585	0,0059	0
GSPATT00020054001	0,28	0,14998	0	GSPATT00036487001	-0,386	0,05882	0
GSPATT00020186001	-0,459	0,06575	0	GSPATT00036711001	0,158	0,40877	0
GSPATT00020286001	-1,05	0,00021	-1	GSPATT00036812001	0,669	0,00387	0
GSPATT00020353001	0,488	0,03007	0	GSPATT00036815001	-0,206	0,27728	0
GSPATT00020355001	0,399	0,03212	0	GSPATT00036909001	-0,089	0,69971	0
GSPATT00020442001	0,565	0,009	0	GSPATT00036976001	0,082	0,61383	0
GSPATT00020780001	-0,921	0,00026	0	GSPATT00037113001	-0,126	0,51661	0
GSPATT00020881001	0,497	0,0253	0	GSPATT00037118001	0,536	0,01752	0
GSPATT00020934001	0,337	0,02604	0	GSPATT00037119001	1,348	0,00024	1
GSPATT00020968001	1,383	0,00001	1	GSPATT00037130001	-0,147	0,41686	0
GSPATT00021053001	0,674	0,02023	0	GSPATT00037160001	1,034	0,00084	1
GSPATT00021078001	0,339	0,1892	0	GSPATT00037555001	1,057	0,0001	1
GSPATT00021123001	-1,327	0,00005	-1	GSPATT00037556001	0,583	0,00547	0
GSPATT00021196001	-0,237	0,09382	0	GSPATT00037585001	1,309	0,00003	1
GSPATT00021225001	0,909	0,00125	0	GSPATT00037590001	0,899	0,00058	0
GSPATT00021341001	0,726	0,00168	0	GSPATT00037672001	1,122	0,00011	1
GSPATT00021345001	0,792	0,00065	0	GSPATT00037716001	0,113	0,41615	0
GSPATT00021421001	0,26	0,09503	0	GSPATT00037729001	0,252	0,07882	0
GSPATT00021453001	0,568	0,01115	0	GSPATT00037730001	0,087	0,55556	0
GSPATT00021463001	0,187	0,32414	0	GSPATT00037899001	1,357	0,00003	1
GSPATT00021514001	-0,385	0,03271	0	GSPATT00037921001	0,469	0,01243	0
GSPATT00021518001	-1,039	0,00036	-1	GSPATT00037923001	0,63	0,00481	0
GSPATT00021521001	0,151	0,3119	0	GSPATT00037924001	0,253	0,13338	0
GSPATT00021537001	0,203	0,36082	0	GSPATT00037953001	0,708	0,00099	0

GSPATT00021553001	0,151	0,41981	0
GSPATT00021724001	0,203	0,34898	0
GSPATT00021829001	0,857	0,00244	0
GSPATT00021996001	0,704	0,01954	0
GSPATT00022065001	0,429	0,02791	0
GSPATT00022194001	0,25	0,11301	0
GSPATT00022292001	0,557	0,00245	0
GSPATT00022293001	0,059	0,75238	0
GSPATT00022405001	-0,359	0,09691	0
GSPATT00022582001	0,485	0,00767	0
GSPATT00022714001	-0,242	0,34305	0
GSPATT00022781001	0,543	0,00457	0
GSPATT00022792001	0,634	0,00443	0

GSPATT00038018001	-0,128	0,53853	0
GSPATT00038150001	0,261	0,13523	0
GSPATT00038173001	0,82	0,03887	0
GSPATT00038174001	0,838	0,12108	0
GSPATT00038238001	-0,208	0,38852	0
GSPATT00038245001	0,014	0,92514	0
GSPATT00038695001	-0,518	0,01381	0
GSPATT00039053001	0,586	0,00254	0
GSPATT00039200001	0,402	0,0233	0
GSPATT00039341001	0,375	0,02393	0
GSPATT00039393001	-0,004	0,98646	0
